# Dual-controlled guest release from coordination cages

**DOI:** 10.1038/s42004-024-01128-z

**Published:** 2024-02-27

**Authors:** Yuqing Yao, Chengyuan Shao, Shuwei Wang, Qiufang Gong, Jia Liu, Hua Jiang, Ying Wang

**Affiliations:** College of Chemistry, Beijing Normal University, Beijing, 100875 P. R. China

**Keywords:** Molecular capsules, Coordination chemistry

## Abstract

Despite having significant applications in the construction of controlled delivery systems with high anti-interference capability, to our knowledge dual-controlled molecular release has not yet been achieved based on small molecular/supramolecular entities. Herein, we report a dual-controlled release system based on coordination cages, for which releasing the guest from the cage demands synchronously altering the coordinative metal cations and the solvent. The cages, **Hg**_**5**_**L**_**2**_ and **Ag**_**5**_**L**_**2**_, are constructed via coordination-driven self-assembly of a corannulene-based ligand. While **Hg**_**5**_**L**_**2**_ shows a solvent-independent guest encapsulation in all the studied solvents, **Ag**_**5**_**L**_**2**_ is able to encapsulate the guests in only some of the solvents, such as acetone-d_6_, but will liberate the encapsulated guests in 1,1,2,2-tetrachloroethane-d_2_. **Hg**_**5**_**L**_**2**_ and **Ag**_**5**_**L**_**2**_ are interconvertible. Thus, the release of guests from **Hg**_**5**_**L**_**2**_ in acetone-d_6_ can be achieved, but requires two separate operations, including metal substitutions and a change of the solvent. Dual-controlled systems as such could be useful in complicated molecular release process to avoid those undesired stimulus-responses.

## Abstract (web summary)

Dual-controlled molecular release is of importance in the construction of controlled delivery systems with high anti-interference capabilities, but has been underexplored in the context of guest release from supramolecular entities. Here, the authors report a coordination cage whereby changes in both the coordinative metal cation and the solvent system are needed to induce guest release.

## Introduction

Capabilities regarding the controlled release of chemical substances are crucial for many applications, including drug delivery^1^, gene transfection carriers^2^, controllable catalysis^3^, and stimuli-responsive functional materials^4^. In the past decades, various entities, from the macroscopic capsules^5^ to polymers^6^ and molecular assemblies^7,8^, have been designed as carriers for controlled releasing drugs or chemicals. Recently, attention has been devoted to the systems with complicated release functions^9^.

Supramolecular cages possess a rigid and isolated (fully or partially) three-dimensional inner space^10,11^. Though such a structural characteristic is greatly beneficial for molecular encapsulation and provides the cages special potentials in reactive intermediate storages^12,13^, catalysis^14^, purification^15,16^, etc., it gives, on the other hand, the controlled release of the encapsulated guests when required being quite challenging. Nonetheless, substantial progress has still been made. A widely used approach in this regard is the disassembly of the cage architectures^17–26^. Besides, competitive molecules (including solvents) were also used for expelling the guests included^27–31^. Other rational designs include denaturing the cages/guests for a significant decrease of the host–guest affinities by various stimuli, such as light^32–36^, metal-coordinations^37,38^, electrolyte^39^, transmetallation^40^, redox^41–43^, reactions^44,45^ and changes in pH^46–48^ or temperatures^43,49,50^. Despite these elegant studies demonstrated a high efficiency in release of guests, the releases themselves typically respond to only a single stimulus, leaving cage systems that can realize precisely controlled release in complicated situations unexplored to a large extent.

**Fig. 1:**
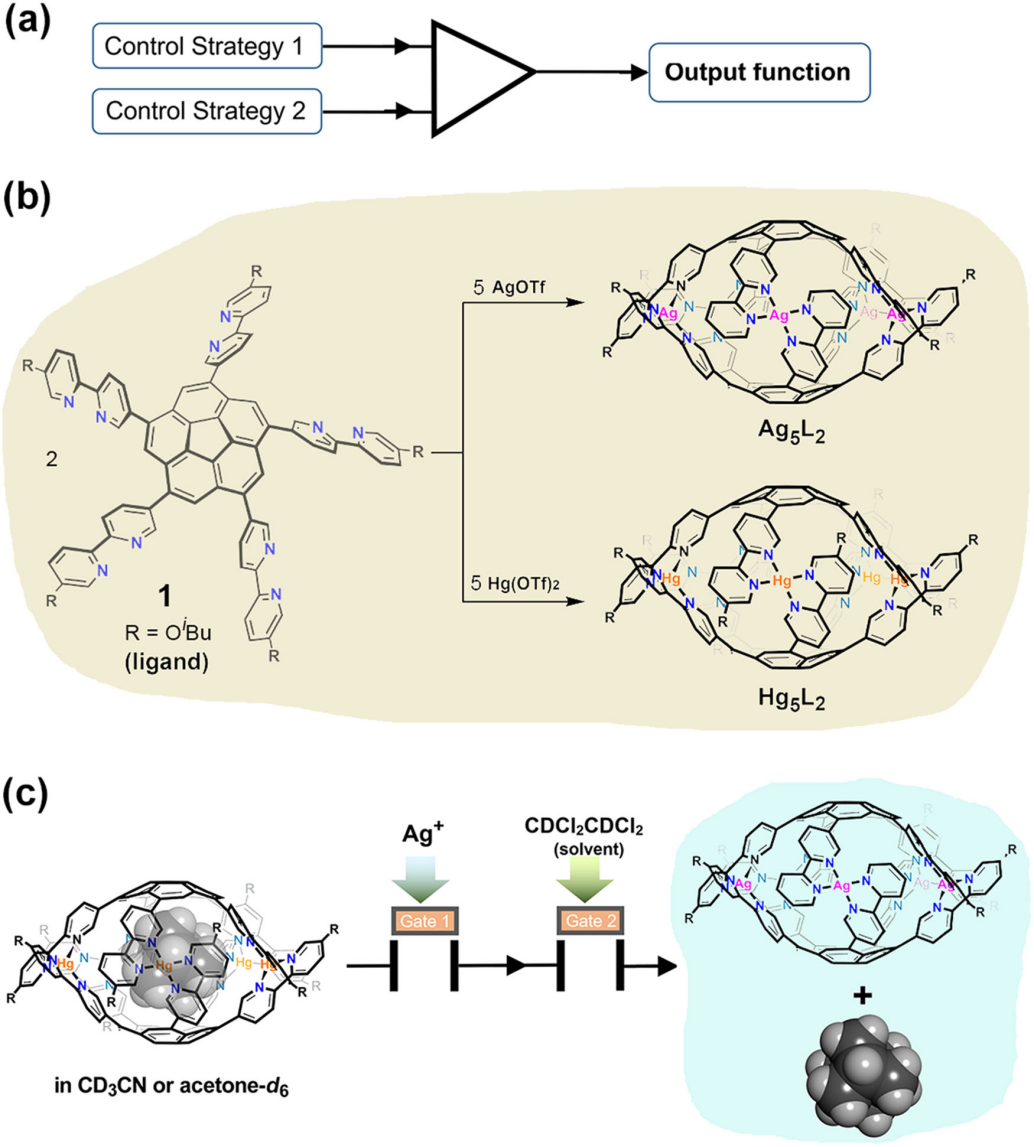
Overview of the dual-controlled guest release from cages. **a** Schematic representation of the dual control processes, in which two control strategies operate in concert to perform tasks. **b** The corannulene-based ligand **1** and the self-assembly to cages **Hg**_**5**_**L**_**2**_ and **Ag**_**5**_**L**_**2**_. The convex-*P,M,P* conformers of the cages are shown as examples. **c** Schematic representation of the dual-controlled guest release system studied herein, for which releasing the guest from **Hg**_**5**_**L**_**2**_ demands synchronous alteration of the coordinative metal cations and the solvent.

Dual control is a regulation that requires at least two separate control strategies operating in concert to perform a task (Fig. 1a). Dual control process has been widely used in high-risk areas of bank transaction^51^ and mechanical engineering^52,53^ to protect information or sensitive functions. In biology, it is adopted to generate a single, integrated response while information from several different sources is received simultaneously^54,55^. Theoretically, employment of such a dual-modality in controlled release would eliminate the uniqueness of the relevance of the release to a certain stimulus, reducing the correlation between the release and other functions relating to the particular stimulus, thus helping to construct anti-interference guest-release systems that are crucial for many applications of such systems in the future, including the drug delivery, gene transfection carriers and controllable catalysis. To date, a few dual-controlled systems have been constructed based on polymers/biopolymers^56,57^ and mesoporous nanoparticles^58–60^ for liberating drugs and chemicals^61,62^. Nevertheless, establishing such a function based on small molecular/supramolecular entities remains an unmet challenge.

Herein, we present, to the best of our knowledge, the first example of a dual-controlled guest release system based on coordination cages, using 1,3,5,7,9-penta(2,2’-bipyridin-5-yl)corannulene (**1**) as the ligand (Fig. 1b). We show that the complexation of **1** with Hg(II) and Ag(I) cations can produce two kinds of well-defined cage complexes, **Hg**_**5**_**L**_**2**_ and **Ag**_**5**_**L**_**2**_. Whereas the **Hg**_**5**_**L**_**2**_ cages show a solvent-independent guest encapsulation in the studied cases, the guest encapsulation and release from **Ag**_**5**_**L**_**2**_ are controlled by the solvents. These two kinds of cages are interconvertible through transmetallation, thus giving the guest release from **Hg**_**5**_**L**_**2**_ to be dually regulated by the metal cations and the solvents (Fig. 1c), while the liberation from **Ag**_**5**_**L**_**2**_ ones is solvent-gated only. Dual-controlled systems, as such, may find applications in complicated cargo release process^63^.

## Results and discussion

### Design and synthesis

Different from those cage systems undergoing significant structural and geometric changes upon stimuli^64^, the ones we attempted to obtain are supposed to change moderately in the cavity shape and volume upon the changes of complexed metal ions. Such features would allow the cages to alter their host capabilities moderately while concurrently maintaining the overall binging inclinations, thus facilitating fine-tuning of their guest-binding behaviors. In this regard, we were drawn to the molecular cages constructed by coordination-driven self-assembly with two high-symmetry building blocks^65^. Within such kind of cages, the metal components are limited in number and therefore function more like a simple linker rather than an important assembly organizer (as that in cages assembled from many small chelating ligands^66^). As a result, changing the coordinated metal cations would give rise to moderate changes in the size, shape, and other properties of the capsular inner space.

Corannulene is an excellent building block for constructing molecular cages due to the *C*_5v_ symmetry in its structure and the high reactivity^67^. In particular, corannulene possesses a curved π-surface and a dynamic, switchable molecular chirality in solution^68^, which can provide theoretically the corresponding cages a lot of intriguing properties that are in sharp contrast to those assembled via planar π-conjugated systems^67,69^. Nevertheless, the properties of corannulene-based molecular cages, including those in the aspect of host–guest interactions, had not been examined for quite a long time. We recently reported the first example of corannulene-based molecular cage^70,71^, constructed by coordination-driven self-assembly of 1,3,5,7,9-penta(pyridyl-3-yl)corannulene ligands and Ag^+^ cations. However, this cage is not suitable for the present purpose because the involved linear bidentate coordination is, to some extent, unfavorable to the modulation of the inner cavity due to the lack of diversity in the coordination pattern. We, therefore, envisioned substituting 2,2’-bipyridin-5-yl (bpy) groups for the pyridyl-3-yl ones on the ligand. Given the presence of various coordination geometries for metal cations with coordination numbers four or six, we expected that, by deliberate choice of metal species, overall the size/shape of the inner cavity could be tuned, thus providing an effective strategy towards controlling guest encapsulations. An *iso*-butoxy side chain was introduced on the tail of each bpy unit for enhanced solubility.

**Fig. 2:**
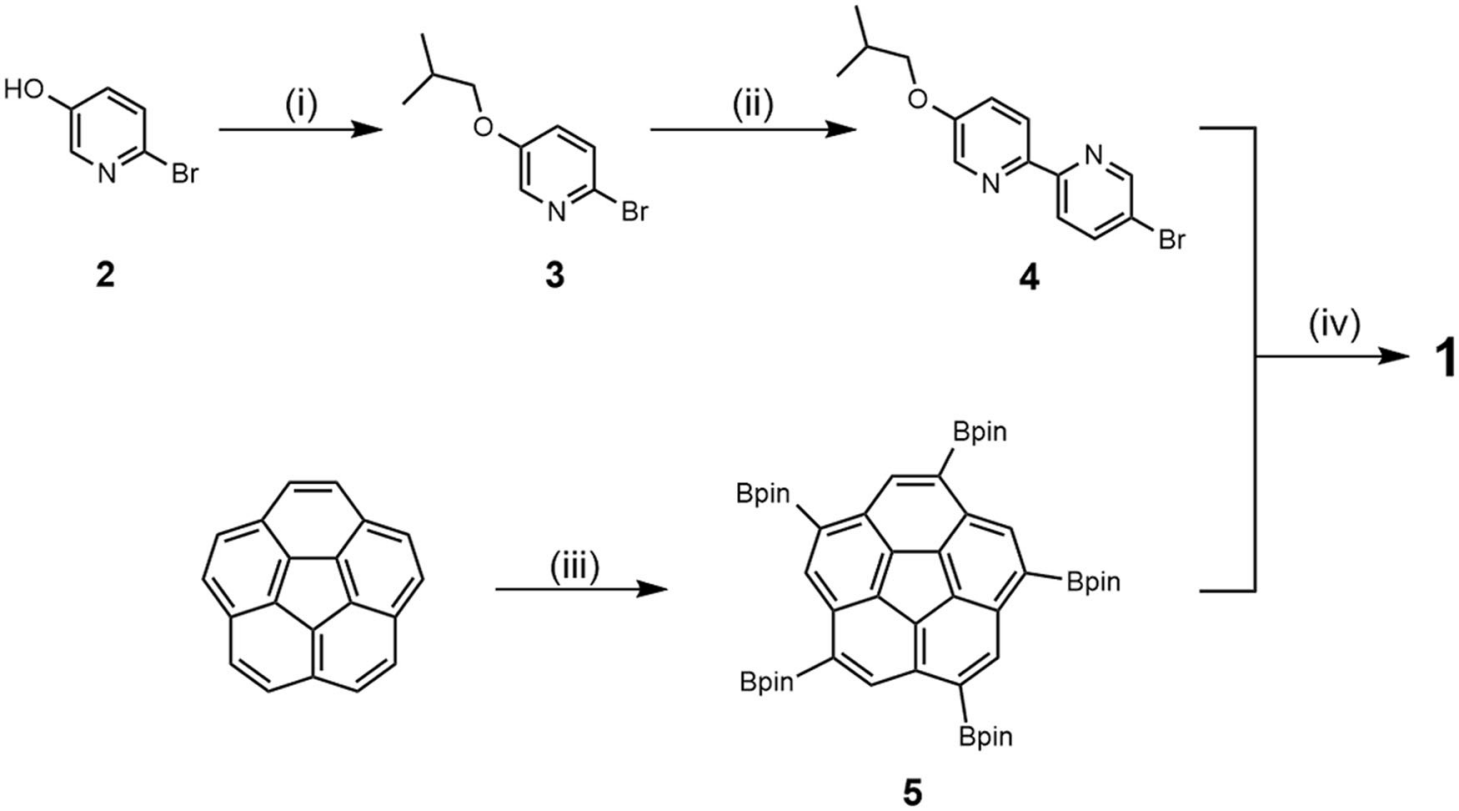
Synthesis of Ligand 1. Reagents and conditions: (i) 1-iodo-2-methylpropane, K_2_CO_3_, DMF, 60 °C, 8 h (50%); (ii) *n*-BuLi, ZnCl_2_, 2,5-dibromopyridine, Pd(PPh_3_)_4_, THF, reflux, 24 h (32%); (iii) [Ir(OMe)COD]_2_, 4,4’-dimethyl-2,2’-bipyridyl, B_2_pin_2_, potassium *t*-butoxide, THF (80%); (iv) Pd(PPh_3_)_4_, K_2_CO_3_, benzene/methanol/water, 100 °C, 4 d (68%).

Our synthetic approach to the bpy ligand is outlined in Fig. 2. Initially, *O*-alkylation of 2-bromo-5-*iso*-butoxypyridine (**2**) with alkyl iodide provided **3**. Treatment of **3** with *n*-BuLi and ZnCl_2_ generated the organozinc in situ, which then underwent Negishi cross-coupling reaction to give **4**. Finally, the Suzuki coupling of **4** with 1,3,5,7,9-pentakis(Bpin)corannulene (**5**) provided the target ligand **1** (Supplementary Methods 1 and 2; Supplementary Data). The structure of **1** was confirmed by nuclear magnetic resonance (NMR) and high-resolution mass spectra. (see Supplementary Figs. 14−17 and 30 in the Supplementary Information (SI)).

### Metal complexation and the formation of Ag_5_L_2_ and Hg_5_L_2_ cages

To explore the feasibility of access to the desired cages and to expand the pool of suitable metal cations, several kinds of metal ions with the potential to coordinate bpy to form four- or six-coordinate complexes^72^, including Ag^+^, Hg^2+^, Fe^3+^, Cu^+^, Mg^2+^, Ni^2+^, and Zn^2+^ ions, were first preliminarily screened. Mixing **1** with Fe(OTf)_3_, Ni(ClO_4_)_2_, Zn(OTf)_2_ or Zn(ClO_4_)_2_ at the ratio of 2:5 (mol/mol) in common solvents or solvents mixture gave no or extremely broad signals in the ^1^H NMR spectra, even after the samples were heated at elevated temperature (Supplementary Fig. S1). When the ligand was combined with Mg(OTf)_2_, no obvious changes were observed, indicative of the absence of coordination. With AgOTf, Hg(OTf)_2_ or Cu(CH_3_CN)_4_PF_6_ as the salt, in the solvent mixture of CD_3_CN/CDCl_3_, the ^1^H NMR spectrum showed a new set of intense signals (Supplementary Figs. 1 and 2), suggestive of a promising formation of well-defined cage complexes. However, the Cu^+^ cages tend to decompose under ambient conditions, as evidenced by the disappearance of its ^1^H NMR signals in three hours. The Ag^+^ and Hg^2+^ complexes are stable enough in solutions; we thus mainly focused on these two cations in the following studies.

It is noteworthy that while both Ag^+^ and Hg^2+^ cations possess the ability to form complexes of several different coordination numbers, they have a flexible coordinating sphere, thus having weak coordination geometry preferences and being able to tolerate, to some extent, the distortion from the ideal geometries^73,74^, which may contribute to the formation of stable molecular cages in the studied cases. Besides, Ag^+^ and Hg^2+^ complexes favor associative ligand exchanges. This character would promote the interconversion between different structures/configurations (if they exist) and greatly help the complexes to rapidly reach the most favourite ones.

**Fig. 3:**
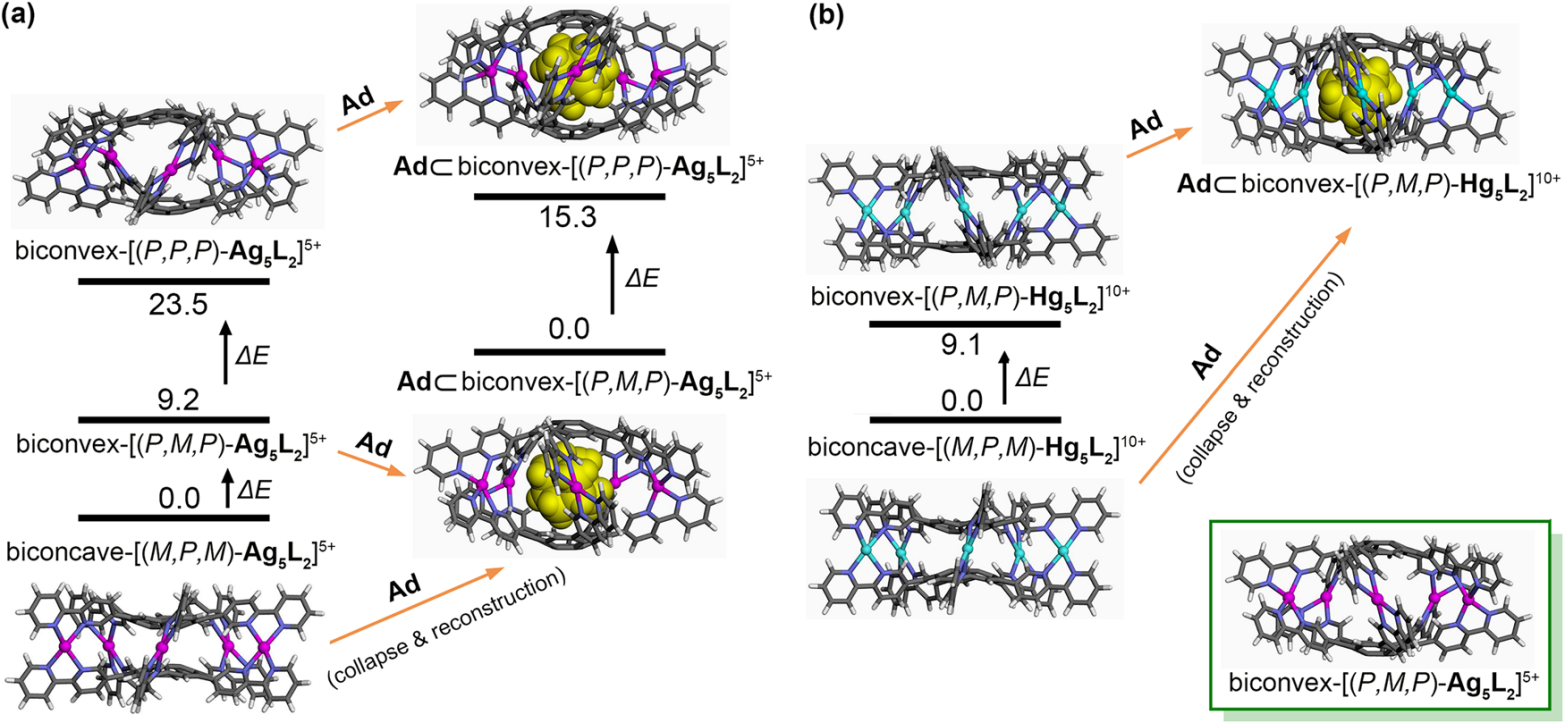
DFT (B3LYP-D3(BJ)//LANL2DZ/6-31 G(d)) energy-minimized structures. **a** Five species relating to the Ag^+^ cages, including biconvex-[(*P,P,P*)-**Ag**_**5**_**L**_**2**_]^5+^, biconvex-[(*P,M,P*)-**Ag**_**5**_**L**_**2**_]^5+^, biconcave-[(*M,P,M*)-**Ag**_**5**_**L**_**2**_]^5+^, **Ad**⊂biconvex-[(*P,M,P*)-**Ag**_**5**_**L**_**2**_]^5+^, and **Ad**⊂biconvex-[(*P,P,P*)-**Ag**_**5**_**L**_**2**_]^5+^. The inset (bottom right corner) shows a side-viewed structure of biconvex-[(*P,M,P*)-**Ag**_**5**_**L**_**2**_]^5+^. **b** Three species relating to the Hg^2+^ cages, including biconvex-[(*P,M,P*)-**Hg**_**5**_**L**_**2**_]^10+^, biconcave-[(*M,P,M*)-**Ag**_**5**_**L**_**2**_]^10+^ and **Ad**⊂biconvex-[(*P,M,P*)-**Hg**_**5**_**L**_**2**_]^10+^. For clarity, the enantiomers of these species are not listed, and **Ad** is represented as CPK sphere. The *iso*-butoxy side chains are replaced with hydrogen atoms to reduce the computational cost. The relative energy levels (black numbers) are provided in kcal mol^−1^.

Theoretically, the inherent chirality of corannulene causes the existence of four different stereo configurations (*P,M,P*/*M,P,M* and *P,P,P*/*M,M,M*)^70^ for the desired Ag^+^ and Hg^2+^ cages. In addition, due to the long bpy substituents as well as the possible bowl-to-bowl inversion of the corannulene moieties, the cages, on the whole, may adopt a clam-shell-like, biconvex structure, or a sunken, biconcave-lens-like geometry^75^. Therefore, totally eight stereoisomers (containing four pairs of enantiomers) are imaginable for each cage, including biconvex*-P,M,P*/*M,P,M* and biconvex*-P,P,P*/*M,M,M* as well as biconcave*-P,M,P*/*M,P,M* and biconcave*-P,P,P*/*M,M,M*. For better understanding of the relationships between different isomers, density functional theory (DFT) (B3LYP-D3(BJ)//LANL2DZ/6-31 G(d)) calculations^76^ were carried out. Careful examinations revealed the possible existence of three pairs of enantiomers for the isolated [Ag_5_**1**_2_]^5+^ cage, including biconvex*-P,M,P*/*M,P,M*, biconvex*-P,P,P*/*M,M,M* and biconcave*-P,M,P*/*M,P,M*; for [Hg_5_**1**_2_]^10+^, two pairs are obtained as minima, including biconvex*-P,M,P*/*M,P,M* and biconcave*-P,M,P*/*M,P,M* (Fig. 3, Supplementary Method 15 and Supplementary Tables 15–17, SI). The other stereoisomers do not represent any minima (local or global) on the corresponding potential energy surface. For examples, optimizations starting from biconcave-(*P,P,P*/*M,M,M*)-[Ag_5_**1**_2_]^5+^ and biconvex-(*P,P,P*/*M,M,M*)-[Hg_5_**1**^2^]^10+^ will give rapidly the geometries of biconvex-(*M,P,M*/*P,M,P*)-[Ag_5_**1**_2_]^5+^ and biconcave-(*M,P,M*/*P,M,P*)-[Hg_5_**1**_2_]^10+^, respectively.

The energy-minimized structures of all the obtained stereoisomers of [Ag_5_**1**_2_]^5+^ and [Hg_5_**1**_2_]^10+^ possess a helical, *D*_5_-symmetric geometry. Notably, the biconcave configuration is, to some extent, conducive to releasing the strain associated with the metal complexations. In energy, biconvex*-*[(*P,M,P*/*M,P,M*)-Ag_5_**1**_2_]^5+^ lies in ca. 14 kcal mol^−1^ lower than that of biconvex*-*[(*P,P,P*/*M,M,M*)-Ag_5_**1**_2_]^5+^, but is ca. 9 kcal mol^−1^ higher than that of biconcave-[(*P,M,P*/*M,P,M*)-Ag_5_**1**_2_]^5+^ (Fig. 3a). Similarly, for [Hg_5_**1**_2_]^10+^, the energy level of the biconvex*-P,M,P*/*M,P,M* conformers is ca. 9 kcal mol^−1^ higher than the biconcave*-P,M,P*/*M,P,M* ones (Fig. 3b). By the same token, biconvex-[(*P,M,P*/*M,P,M*)-Hg_5_**1**_2_]^10+^ is more flat (6.9 Å in height, Supplementary Table 15), compared to biconvex-[(*P,M,P*/*M,P,M*)-Ag_5_**1**_2_]^5+^ (height = 9.1 Å).

**Fig. 4:**
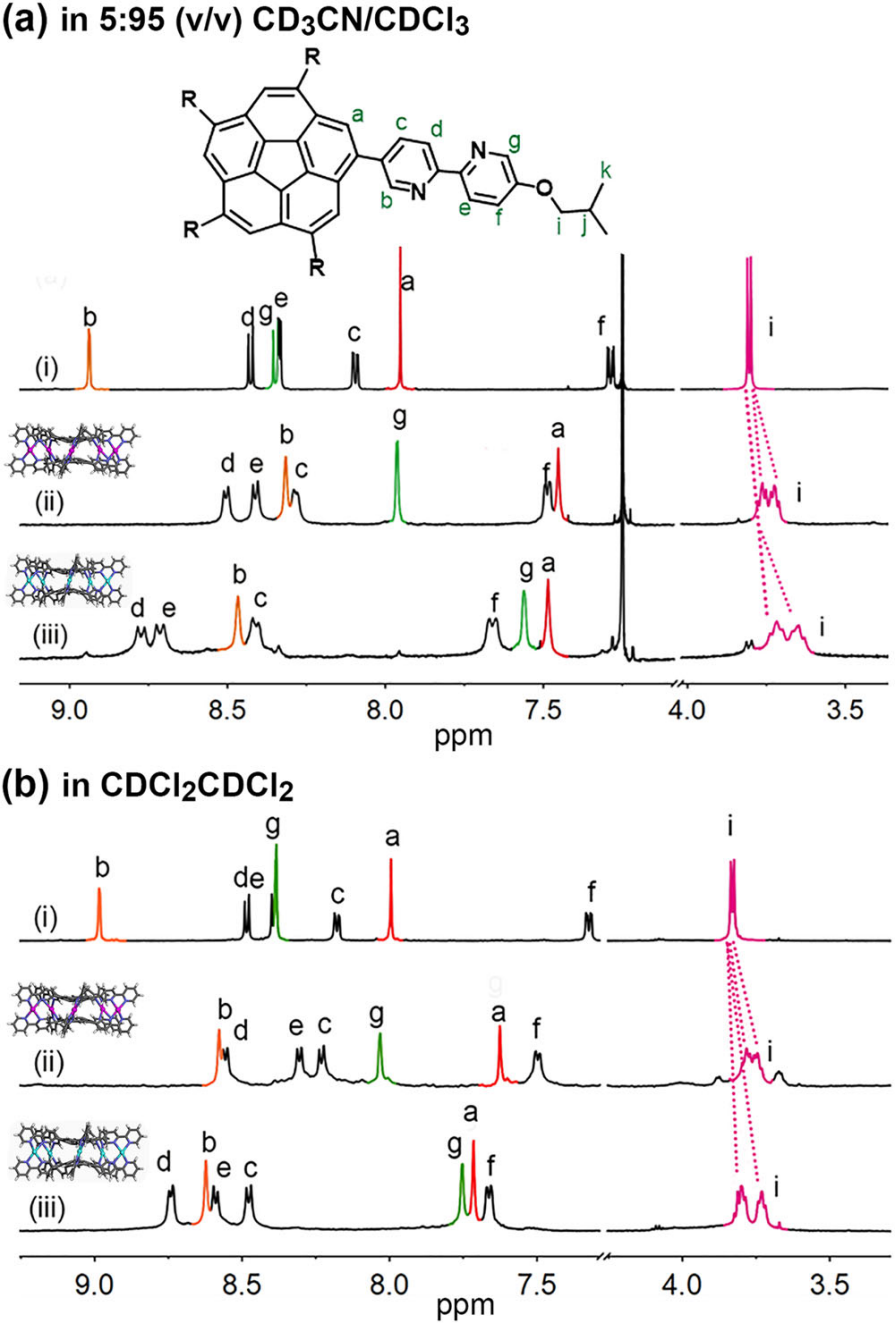
Coordination-driven self-assemblies of the Ag(I)/Hg(II) molecular cages. **a** ^1^H NMR spectra of (i) the ligand (**1**), (ii) [Ag_5_**1**_2_]∙[OTf]_5_ (**Ag**_**5**_**L**_**2**_) and (iii) [Hg_5_**1**_2_]∙[OTf]_10_ (**Hg**_**5**_**L**_**2**_) in 5:95 (v/v) CD_3_CN/CDCl_3_. **b** ^1^H NMR spectra of (i) the ligand (**1**), (ii) [Ag_5_**1**_2_]∙[OTf]_5_ (**Ag**_**5**_**L**_**2**_) and (iii) [Hg_5_**1**_2_]∙[OTf]_10_ (**Hg**_**5**_**L**_**2**_) in CDCl_2_CDCl_2._ The formations of cages give significant upfield shifts of those protons located in the inner cavity, including H_a_, H_b_ and H_g_, and a split of the H_i_ signals, compared to the ligand **1**.

To further confirm the formation of the cages, more experiments were carried out. ^1^H NMR titration of Ag(OTf) to **1** in 5:95 (v/v) CD_3_CN/CDCl_3_ clearly showed that, upon the addition of Ag^+^ cations, the signals of ligands gradually became weaker, and a new set of signals corresponding to the cages appeared (Fig. 4a and Supplementary Figs. 3 and 18, 19, SI). Based on the results of DFT calculations, it is reasonable to assign this new set of signals to the racemic [biconcave*-*(*P,M,P*)/(*M,P,M*)-Ag_5_**1**_2_]·[OTf]_5_. The complexation induced significant upfield shifts of the protons, which are supposed to be located in the inner cavity, such as *H*_a_, *H*_b_, and *H*_g_, as ascribed to the strong shielding of the cage (Fig. 4a). In particular, as an important indicator for the formation of cages, the methylene protons H_i_ on the *iso*butyl side chains split into two sets upon complexation, which results from the inequivalence between the proton directed inward and outward of the cage^70^. Additional evidence for the formation of cage complexes was also provided by the electrospray ionization—high-resolution mass spectrometry (ESI-HRMS) spectrum (Supplementary Fig. 31 and Supplementary Table 5), in which a series of prominent signals assignable to [Ag_m_**1**_2_·(OTf)_*n*_]^*m*–*n*^ (*m* ≤ 5) can be clearly observed. 2D diffusion ordered spectroscopy (DOSY) spectrum indicated the formation of a single product with a diffusion coefficient of *D* = (1.42 ± 0.01) × 10^−10^ m^2^ s^−1^, which is much smaller than that of the ligand in the same solvent (*D* = (3.76 ± 0.05) × 10^−10^ m^2^ s^−1^) (Supplementary Method 7, Supplementary Figs. 24−27 and Supplementary Table 4).

Except 5:95 (v/v) CD_3_CN/CDCl_3_ (dielectric constant, *ε* ≈ 6.4), other three kinds of solvents, including acetone-*d*_6_ (*ε* = 21), CD_3_CN (*ε* = 37), and 1,1,2,2-tetrachloroethane-*d*_2_ (CDCl_2_CDCl_2_, *ε* = 8.5) were also investigated. The **Ag**_**5**_**L**_**2**_ cages exhibited well-resolved ^1^H NMR spectra in acetone-*d*_6_ and CDCl_2_CDCl_2_ (Fig. 4b and Supplementary Fig. 4d); nevertheless, in CD_3_CN, broadened, overlapped signals were observed, which is probably due to the aggregation of the cages in this solvent (Supplementary Fig. 4a).

For the construction of the Hg^2+^ cages, initially, we found that the titration of Hg(OTf)_2_ to **1** gave very messy spectra. Nevertheless, adding 2.5 equiv. of the Hg^2+^ cations in a whole produced a distinct new set of signals, which can be assigned to biconcave*-*[(*P,M,P*/*M,P,M*)-Hg_5_**1**_2_]·[OTf]_10_ according to the results of DTF calculations, in all the cases of 5:95 (v/v) CD_3_CN/CDCl_3_, CD_3_CN, acetone-*d*_6_, and CDCl_2_CDCl_2_ (Fig. 4 and Supplementary Figs. 53 and 54). The formations of the Hg^2+^ cage are well confirmed by the split of *H*_i_ signal in the ^1^H NMR spectra as well as the corresponding HRMS (Supplementary Fig. 93 and Supplementary Table 12) and 2D NMR spectra (Supplementary Figs. 83–86 and 91).

### Solvent-dependent guest encapsulation and release from Ag_5_L_2_

Since no valuable results can be obtained from our isothermal titration calorimetry (ITC) experiments, the binding behaviour of the cages was investigated by ^1^H NMR spectroscopy at 298 K. Considering the concave surface of corannulene, three kinds of molecules, including pseudo-spherical adamantane (**Ad**) and two of its derivatives, namely, 1-adamantanemethanol (**Ad-MeOH**) and 1-adamantanecarboxylic acid (**Ad-COOH**), was chosen as the guests in the studies.

The guests competed successfully (but laboriously) for the inner space of **Ag**_**5**_**L**_**2**_ with the solvent molecules in 5:95 (v/v) CD_3_CN/CDCl_3_. For example, titration of **Ad** to **Ag**_**5**_**L**_**2**_ produced an obvious attenuation of the signals of the cages and, meanwhile, the appearance and gradual enhancement of a new set of signals corresponding to the stable host–guest complexes (Supplementary Method 4 and Supplementary Fig. 5). A 1:1 encapsulation is strongly suggested by the ESI-HRMS of the cage-guest complexes (Supplementary Fig. 32 and Supplementary Table 5); and the DTF calculations showed that the cage can accommodate only one guest molecule as well (Fig. 3a and Supplementary Table 16). ^1^H NMR titration of **Ad-MeOH** or **Ad-COOH** gave very similar results (Supplementary Figs. 6, 7, 33, 34 and Supplementary Table 5). Notably, the DFT calculations predicted **Ad**⊂[biconvex-(*P,M,P*)/(*M,P,M*)-**Ag**_**5**_**L**_**2**_]^5+^ complexes of 15 kcal mol^−1^ lower in energy in the gas phase (Fig. 3a), compared to that of **Ad**⊂[biconvex-(*P,P,P*)/(*M,M,M*)-**Ag**_**5**_**L**_**2**_]^5+^ ones, and a cage collapse for biconcave-[(*P,M,P*/*M,P,M*)-Ag_5_**1**_2_]^5+^ with **Ad** included due to their too small cavities, such that the cages might adopt biconvex-*P,M,P*/*M,P,M* conformations upon the guest complexations.

**Fig. 5:**
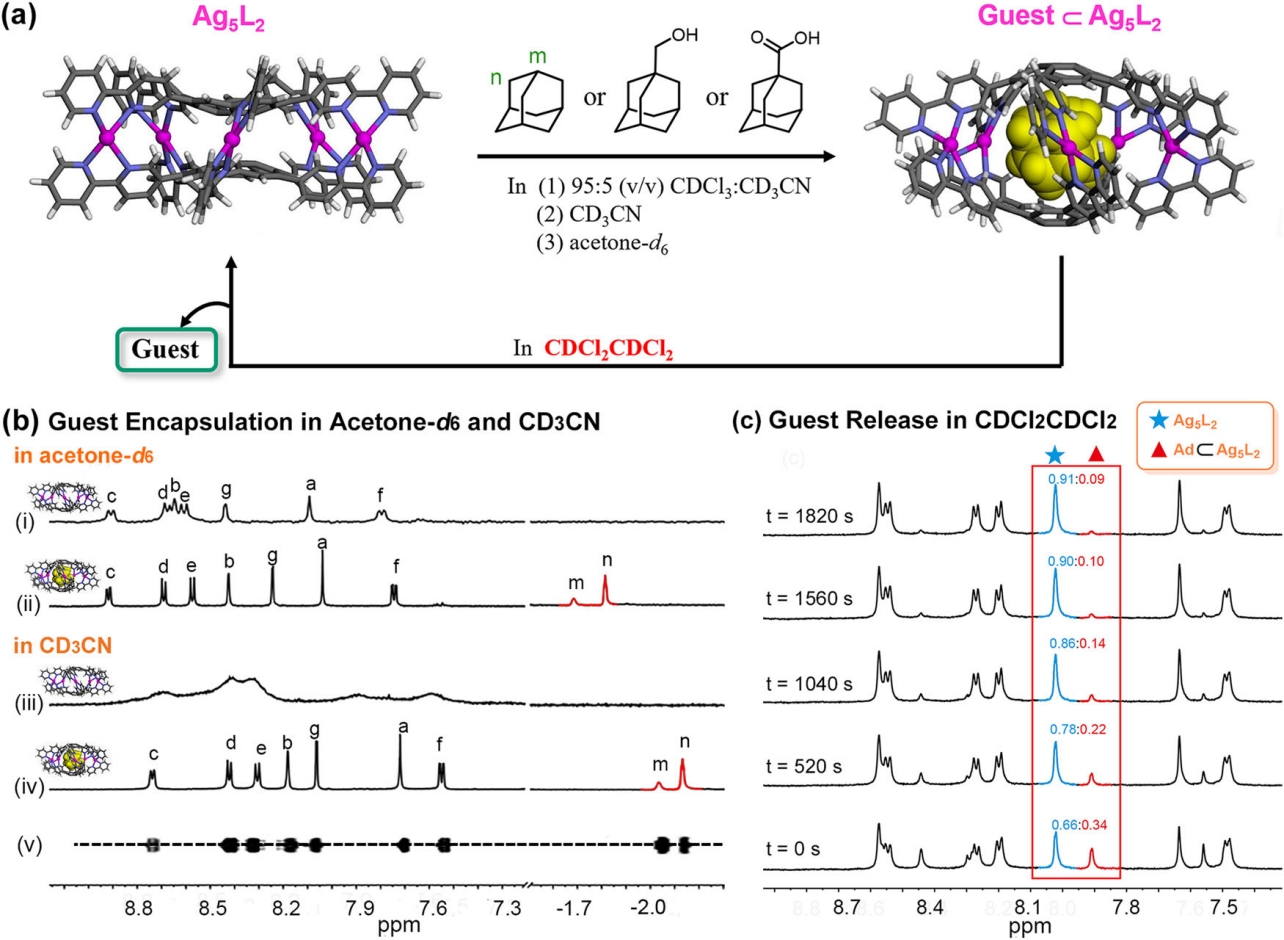
Solvent-dependent guest encapsulation and release from Ag_5_L_2_ cages. **a** A schematic representation of the host–guest chemistry of **Ag**_**5**_**L**_**2**_ in different solvents or solvent mixture. **Ag**_**5**_**L**_**2**_ can encapsulate the studied guests in 5:95 (v/v) CD_3_CN/CDCl_3_, CD_3_CN and acetone-*d*_6_. However, in CDCl_2_CDCl_2_, the included guests are gradually released from the host–guest complexes. **b** ^1^H NMR spectrum (600 MHz, 298 K, 1 mM) of (i) **Ag**_**5**_**L**_**2**_ and (ii) **Ad**⊂**Ag**_**5**_**L**_**2**_ in acetone-*d*_6_ as well as that of (iii) **Ag**_**5**_**L**_**2**_, (iv) **Ad**⊂**Ag**_**5**_**L**_**2**_ in CD_3_CN. The DOSY spectrum (v) of **Ad**⊂**Ag**_**5**_**L**_**2**_ in CD_3_CN showed that all signals from **Ad** with **Ag**_**5**_**L**_**2**_ were bound to diffuse at the same rate. **c**, Time-dependent ^1^H NMR spectra (600 MHz, 301 K) of the mixture of **Ag**_**5**_**L**_**2**_ and **Ad**⊂**Ag**_**5**_**L**_**2**_ in CDCl_2_CDCl_2._ For experimental details, see Supplementary Method 8, SI.

In acetone-*d*_6_, addition of the studied guests to **Ag**_**5**_**L**_**2**_ resulted in also the guest inclusions as observed in the ^1^H NMR spectra (Fig. 5b, Supplementary Method 5 and Supplementary Figs. 8−10). The signals of the host–guest complexes are pretty dispersed, thus being assignable, providing a good chance to explore the encapsulation behaviors. A closer examination of the ^1^H NMR spectra indicated that the complexation induced obvious changes in chemical shift for the protons that are supposed to be located in the inner cavity, i.e., H_a_ and H_b_, which is due to the σ−π interaction between the encapsulated guest and the host. The encapsulated guests experience a highly shielded nano-environment, thus showing one set of signals in the range of (–1.5–(–2.0)) ppm) (Fig. 5b). The encapsulation was unambiguously verified further by the 2D NOESY spectrum, which exhibited strong correlations between the protons of **Ad** and H_a_/H_c_ of the cages (Supplementary Figs. 20–23). Interestingly, in the ^1^H NMR spectra, the protons that are supposed to stay far from the encapsulated molecules, including H_e_, H_f_ and H_g_, also shifted to some extent, which is probably caused by a slight deformation of the cage upon guest encapsulations. In fact, the encapsulation-induced expansion was supported by the DFT calculations, which predicted a height of 10.0 Å for the energy-minimized structure of **Ad**⊂biconvex-[(*P,M,P*)/(*M,P,M*)-Ag_5_L_2_]^5+^ (Supplementary Tables 15, 16), a little larger than that of the free cages (ca. 9.1 Å).

**Ag**_**5**_**L**_**2**_ includes the studied guests in CD_3_CN as well. This is indicated by the appearance of a set of well-resolved signals in the ^1^H NMR titration experiments, despite the cage itself cannot show distinct signals in such a solvent (Fig. 5biii–iv, Supplementary Method 6 and Supplementary Figs. 11–13). In addition, the DOSY spectrum showed all signals from **Ag**_**5**_**L**_**2**_ and **Ad** diffusing at the same rate (Fig. 5bv and Supplementary Figs. 28, 29).

Though the ^1^H NMR signals of the cage-guest complexes in 5:95 (v/v) CD_3_CN/CDCl_3_ are not assignable due to the weak host–guest association (giving the coexist of **Ad**⊂**Ag**_**5**_**L**_**2**_ and **Ag**_**5**_**L**_**2**_) and the aggregation of the signals themselves, the molar ratio (*χ*) of **Ad**⊂**Ag**_**5**_**L**_**2**_ against **Ag**_**5**_**L**_**2**_ can still be derived from the integrations of the signals corresponding to the free cage and the cage-guest complex (Supplementary Methods 4 and Supplementary Table 1), which provided further the association constant (*K*_a_) of 23.4 ± 3.2 M^–1^, 21.7 ± 1.6 M^–1^ and 23.1 ± 6.5 M^–1^ for **Ad,** **Ad-MeOH**, and **Ad-COOH**, respectively (Table 1). The association constants in acetone-*d*_6_ and CD_3_CN can be determined in a similar manner (Supplementary Methods 5, 6 and Supplementary Tables 2, 3). Besides, compared to the case of 5:95 (v/v) CD_3_CN/CDCl_3_, the cage has ~3 orders of magnitude greater affinity for each guest in acetone-*d*_6_, and it is enhanced further when changing the solvent to CD_3_CN (Table 1). Nevertheless, in each particular solvent, the association constant is close to one other for different guests, as a comprehensive result of the interactions between the guests and the coordination cages as well as the solvents. Notably, overall, the guest-binding affinities are higher in more polar solvents. This could be first ascribed to a weaker competition from the solvent molecules for the guests with respect to host binding. Besides, in the cases of **Ad-COOH** and **Ad-OH**, looser ion-pairs between [Ag_5_**1**_2_]^5+^ and the counter anions (OTf^‒^) present in such situations, which is greatly conducive to the electrostatic interactions between the constituent metal cations and the included guests.

**Table 1:** Association constants (*K*_a_, M^−1^) and rate (*k*, s^−1^) of release of various guests from Ag_5_L_2_ in different solvents at 298 K

Guest	Encapsulation			Release
	CD_3_CN	95:5 (v/v) CDCl_3_:CD_3_CN	Acetone-*d*_6_	CDCl_2_CDCl_2_
	*K*_a_ (M^−1^)	*K*_a_ (M^−1^)	*K*_a_ (M^−1^)	*k* (s^−1^)^*a*^
	(1.2 ± 0.1) × 10^3^	23.4 ± 3.2	(2.1 ± 0.8) × 10^3^	5.1 × 10^−4^
	(1.4 ± 0.2) × 10^4^	21.7 ± 1.6	(6.8 ± 1.5) × 10^3^	2.0 × 10^−3^
	(8.9 ± 0.5) × 10^3^	22.3 ± 4.7	(9.1 ± 2.5) × 10^2^	4.4 × 10^−4^

^*a*^The kinetic data at 298 K are calculated by the linear extrapolation method^77^. For more parameters, see Tables S7, SI.

In contrast to those in the solvents mentioned above, **Ag**_**5**_**L**_**2**_ showed much different binding behavior in CDCl_2_CDCl_2_. In such a solvent, addition of the studied guests to **Ag**_**5**_**L**_**2**_ gave no changes in the ^1^H NMR spectrum. Moreover, when the samples of guest⊂**Ag**_**5**_**L**_**2**_ complexes prepared in CD_3_CN were dried under vacuum and then re-dissolved in CDCl_2_CDCl_2_, with increasing the standing time, at room temperature, the ^1^H NMR signals of the cage-guest complexes became (fast or gradually) weaker, along with the appearance of a new set of signals corresponding to the free cages, indicating that the encapsulated guests were expelled by the solvent molecules in such cases. The release processes were then monitored by ^1^H NMR spectroscopy at various temperatures, in which the range of temperature investigated (301−208 K, 292−279 K, and 288−274 K for **Ad,** **Ad-MeOH** and **Ad-COOH**, respectively) was carefully chosen to give the changes measurable at reasonable time scales (Supplementary Method 8 and Supplementary Figs. 35−52). First-order rate constants, *k*, for the release, were obtained by monitoring the decay of the signals of the cage-guest complexes and the thriving of the free cages (Supplementary Tables 8–10). Using the rate constants obtained, the enthalpic (Δ*H*^⧧^) and entropic (Δ*S*^⧧^) contributions to the transition state were calculated from Eyring plots (Supplementary Table 6). Other kinetic parameters at 298 K were next calculated using a linear extrapolation method (Supplementary Table 7)^77^.

The results show that the release rates in CDCl_2_CDCl_2_ follow the order of **Ad-MeOH** > **Ad** > **Ad-COOH**, but are basically of the same order of magnitude (Table 1). In all cases, the formation of activated complexes (the transition state) is an exothermic process (Supplementary Table 7). In the transition states, the inner cavity of the cage should be crowded with the studied guest and solvent molecules. The cage therefore needs energy to adjust itself to overcome the steric hindrance of the molecules inside. Compared to the other two kinds of guests, passing through the transition state in the case of **Ad-COOH** is less enthalpically but more entropically favored. This is probably due to the self-association of **Ad-COOH** in CDCl_2_CDCl_2_, which is a process associated with exotherm and an entropic reduction.

### Solvent-independent guest encapsulation in Hg_5_L_2_

With the data indicative of a solvent-controlled guest binding and release from cage **Ag**_**5**_**L**_**2**_ in hand, we next set out to study the binding behaviors of **Hg**_**5**_**L**_**2**_. Considering that the DFT calculations predicted a smaller height for the cavity of biconvex-[(*P,M,P*/*M,P,M*)-Hg_5_**1**_2_]^10+^ compared to biconvex-[(*P,M,P*/*M,P,M*)-Ag_5_**1**_2_]^5+^, we expected that the Hg^2+^ cage could bind the studied guests in a different manner.

DFT calculations provide a predictive insight into the encapsulation in **Hg**_**5**_**L**_**2**_, which showed that the inner cavity of biconcave*-P,M,P*/*M,P,M* are too small to accommodate **Ad** (inclusion of the guest will give a disassembly of the cage) so that only **Ad**⊂biconvex-[(*P,M,P*)/(*M,P,M*)-Hg_5_**1**_2_]^10+^ can be formed (Fig. 3b). The guest encapsulations lead to a larger deformation in the cage structure compared to that in the case of **Ag**_**5**_**L**_**2**_, particularly regarding the expansion of the inner cavity (from 6.9 Å for **Hg**_**5**_**L**_**2**_ to 9.5 Å for **Ad** ⊂**Hg**_**5**_**L**_**2**_ in height, compared to that from 9.1 Å for **Ag**_**5**_**L**_**2**_ to 10.0 Å for **Ad**⊂**Ag**_**5**_**L**_**2**_, Supplementary Table 15).

Experimentally, encapsulations of the guests with **Hg**_**5**_**L**_**2**_ are kinetically much harder than those in the cases of **Ag**_**5**_**L**_**2**_ as expected (Supplementary Method 9). In all the studied solvents, directly adding the studied guests to **Hg**_**5**_**L**_**2**_ at ambient temperature gave no changes in the ^1^H NMR spectra, suggestive of higher encapsulation energy barriers. We thus tried different procedures to achieve the encapsulations.

Overall, the encapsulation barriers in CD_3_CN and in acetone-*d*_6_ is apparently similar to each other. Two procedures (labeled **Procedure A** and **B**, Supplementary Method 10) were utilized to prepare the cage-guest complexes in such cases. In **Procedure A** (from Cages to Cage-guest Complexes), to the ligand in CD_3_CN (or acetone-*d*_6_), 2.5 equiv. of Hg^2+^ cations and the guests were added in sequence, and the obtained samples were sonicated for 30 min at ambient temperature. In **Procedure B** (One-Pot Construction), the mixtures of the ligand and the guest in CD_3_CN (or acetone-*d*_6_) were added 2.5 equiv. of Hg^2+^ cations, followed by a sonication at ambient temperature. Both procedures resulted in the same outcomes. In the ^1^H NMR spectra, the guest encapsulations gave rise to the disappearance of the cage signals and the presence of the new ones corresponding to the host–guest complexes (Supplementary Figs. 6b, 55–60, 87–90, 92, and 94 and Supplementary Table 13). Particularly, distinct resonances from the encapsulated guests in the inner cavity can be clearly observed in a high-field region of −1.0 ~−3.0 ppm.

In 5:95 (v/v) CD_3_CN/CDCl_3_, it is hard to produce the cage-guest complexes by sonicating the mixtures of the cage and the guests, or the mixtures of the ligand, Hg^2+^ cations and the guests, at ambient temperature. Nevertheless, by heating the mixtures of the cage and the guests at 50 °C for 2–3 days (**Procedure C**, Supplementary Method 11), the desired encapsulations can still be achieved (Supplementary Fig. 61). Alternatively, the complexes can be first prepared in CD_3_CN or acetone-*d*_6_, followed the replacement of the solvent with 5:95 (v/v) CD_3_CN/CDCl_3_ (**Procedure D**, Solvent-replacement Method, Supplementary Method 11 and Supplementary Figs. 62–64).

**Fig. 6:**
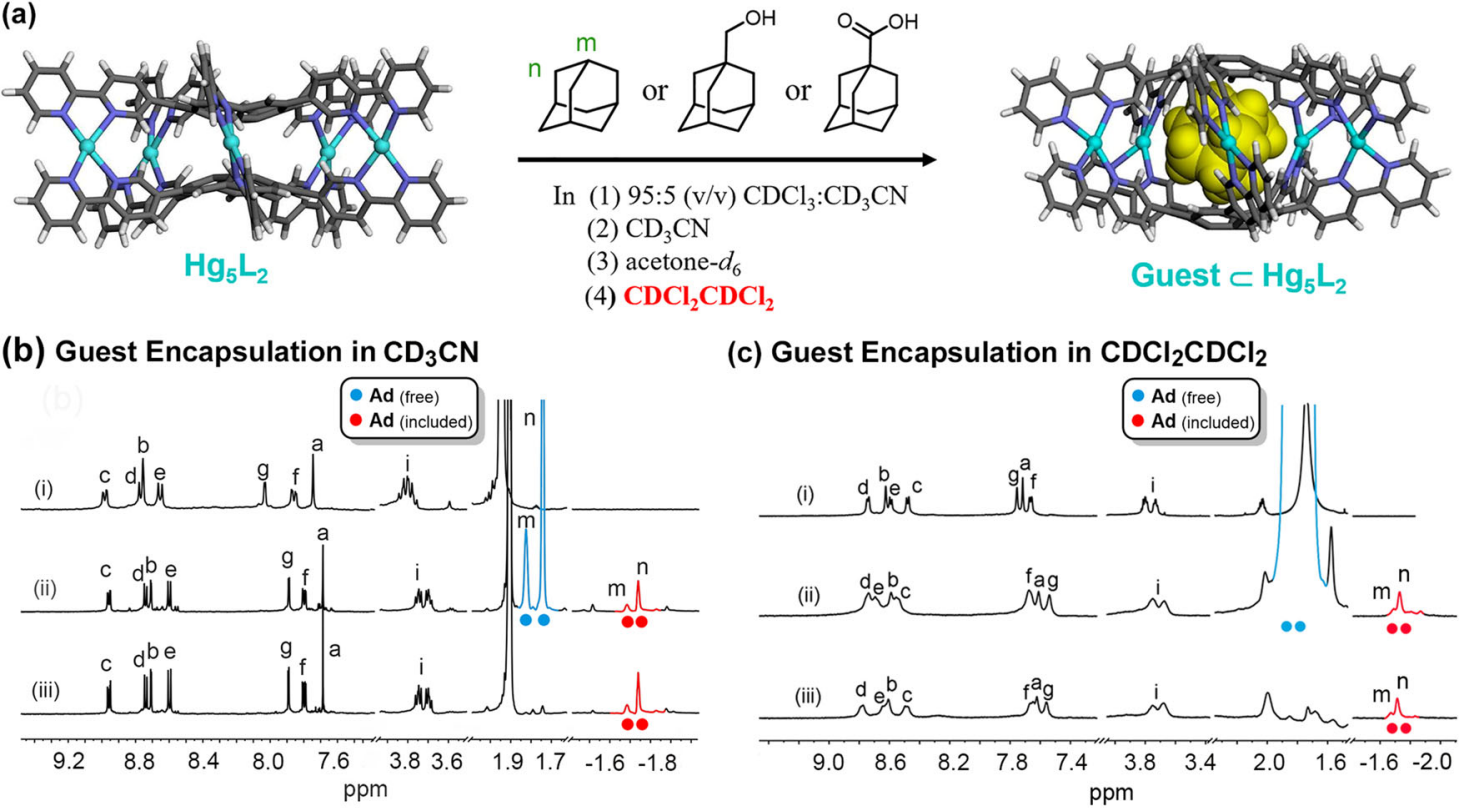
Solvent-independent guest encapsulation in the Ag_5_L_2_ cage. **a** Schematic representation of the host–guest chemistry of **Hg**_**5**_**L**_**2**_ in different solvents or solvent mixture. In all the studied solvent systems, including CDCl_2_CDCl_2_, the **Hg**_**5**_**L**_**2**_ cages can encapsulate the guests to form stable host–guest complexes. **b** ^1^H NMR spectra (600 MHz, 298 K, 1 mM, CD_3_CN) of (i) **Hg**_**5**_**L**_**2**_, (ii) **Ad**⊂**Hg**_**5**_**L**_**2**_ in the presence of 8 equiv. of **Ad** (prepared via **Procedure A**), and (iii) **Ad**⊂**Hg**_**5**_**L**_**2**_ in the absence of free **Ad** after the solution was stored at 298 K for 2 days. **c** ^1^H NMR spectra (600 MHz, 298 K, 1 mM CDCl_2_CDCl_2_) of (i) **Hg**_**5**_**L**_**2**_, (ii) **Ad**⊂**Hg**_**5**_**L**_**2**_ in the presence of 200 equiv. of **Ad** (prepared via **Procedure E**), and (iii) **Ad**⊂**Hg**_**5**_**L**_**2**_ in the absence of free **Ad** after the solution was kept at 298 K for 2 days. For experimental details, see Methods.

With CDCl_2_CDCl_2_ as the solvent, the encapsulations are even harder, which cannot be achieved via **Procedure C**. However, taking **Ad** as an example, when heating the mixtures of the cage and a large excess of the guest (200 equiv.) at 90 °C for 6 days, the cages underwent a complete transformation to the cage–guest complexes (**Procedure E**, Fig. 6c, Supplementary Method 12) (Supplementary Fig. 65). The same result can be obtained by heating the mixtures of the ligand, Hg^2+^ cations and the guests at 90 °C for 3 days (**Procedure F**, Supplementary Method 12) (Supplementary Figs. 66 and 67). Nevertheless, the simplest way to prepare the cage-guest complexes in such a situation still points to **Procedure D**, except that the solvent, CD_3_CN or acetone-*d*_6_, was finally replaced by CDCl_2_CDCl_2_ after the accomplishment of the guest encapsulations (Supplementary Method 11) (Supplementary Figs. 68−70).

We next assessed the stabilities of the **Hg**_**5**_**L**_**2**_-guest complexes in the studied solvents (or solvent mixture) at room temperature. In the investigations, the **Hg**_**5**_**L**_**2**_-guest complexes were first prepared in acetone-*d*_6_ according to **Procedure A** and then dried. Since a large amount of guest was used in the procedure, to get a clear observation on the possible release of the guests as well as to avoid the possible exchange of the guest molecules between inside and outside of the cage cavity, the free guests in the obtained solid mixtures were next removed completely by solvent-washing using cyclohexane. After the pure cage-guest complexes were re-dissolved in the studied solvents (or solvent mixture), decays of the complexes were monitored at 25 °C by ^1^H NMR spectroscopy for 2 days, and the stabilities of the complexes were assessed according to whether the signals of the free cages and the free guests reappear in the spectra (Methods, the main text).

The results show that, in all the studied solvents, particularly in the cases of CDCl_2_CDCl_2_, no included guest molecules were expelled from the cages (Fig. 6b, c Supplementary Table 11 and Supplementary Figs. 71–82). This is very interesting because such results are unambiguously indicative of a solvent-independent guest encapsulation for the Hg^2+^ cages in the studied cases (Fig. 6a).

### Ag_5_L_2_ ⇆ Hg_5_L_2_ conversions

**Fig. 7:**
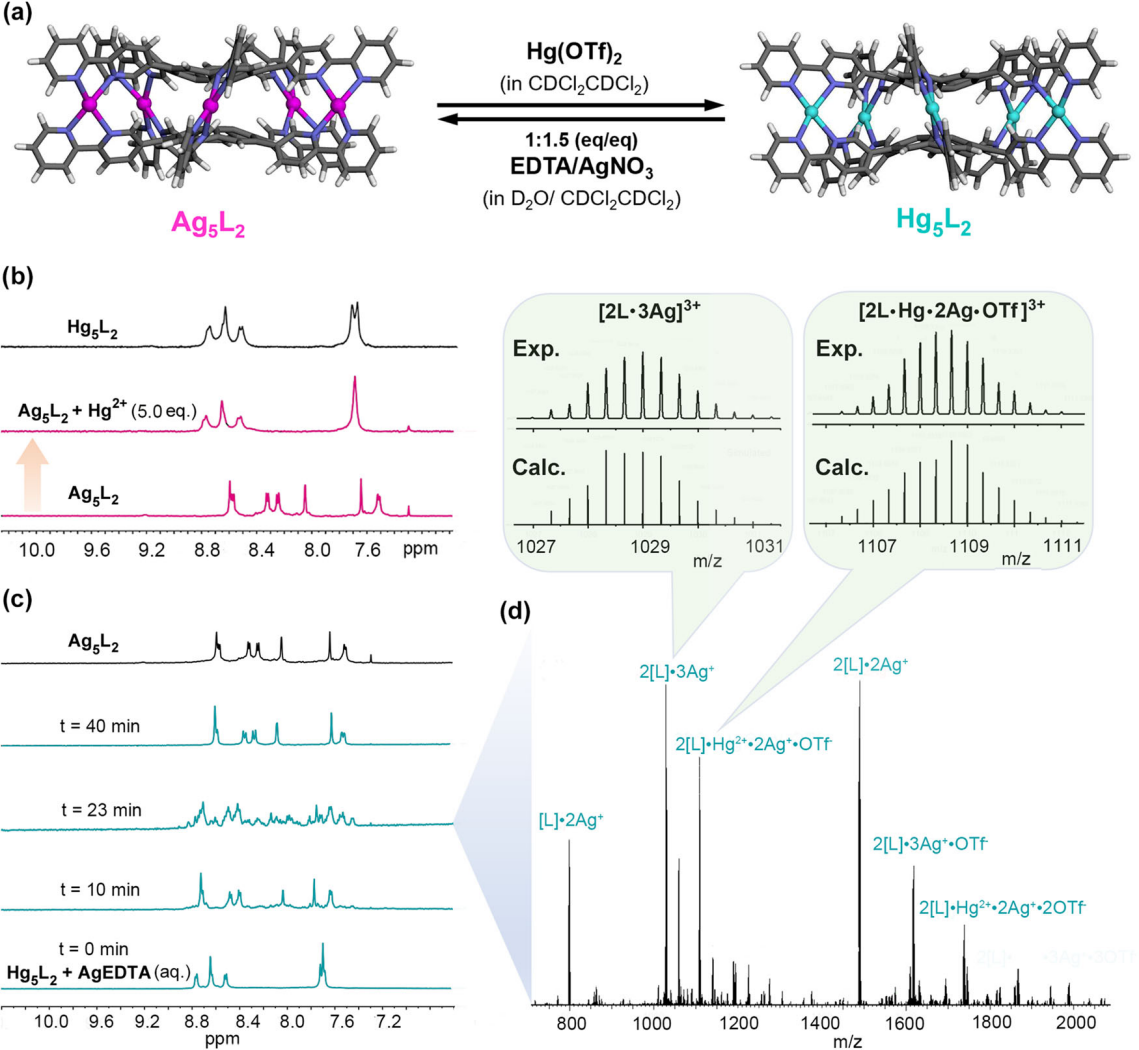
Interconversions between Ag_5_L_2_ and Hg_5_L_2_. **a** Schematic representation of the transmetallation-guided interconversions. **b** ^1^H NMR spectra (600 MHz, 298 K, CDCl_2_CDCl_2_) of **Ag**_**5**_**L**_**2**_ and **Ag**_**5**_**L**_**2**_ in the presence of 5.0 equiv. of Hg^2+^ cation. The latter produced a solution of **Hg**_**5**_**L**_**2**_ immediately due to a much stronger association strength with ligand **1** for the Hg^2+^ cations. For comparison, the spectrum of **Hg**_**5**_**L**_**2**_ in CDCl_2_CDCl_2_ is shown. **c** Stacked plots of the time-depended ^1^H NMR spectra (600 MHz, 298 K, CDCl_2_CDCl_2_) of the organic phase of the mixture of **Hg**_**5**_**L**_**2**_ in CDCl_2_CDCl_2_ and an aqueous solution of **AgEDTA** under vigorous stirring at 28 °C. The metal cation exchanges that occurred on the water-CDCl_2_CDCl_2_ interface gave rise to a formation of **Ag**_**5**_**L**_**2**_ finally. The spectrum of **Ag**_**5**_**L**_**2**_ in CDCl_2_CDCl_2_ is shown for comparison. **d** The corresponding ESI-MS spectrum of the sample at *t* = 23 min. The insets show the experimental and simulated isotopic clusters for two intermediate species, [2 L•3Ag]^3+^ and [2 L•Hg•3Ag•OTf]^3+^.

Transmetallations have been proven to be an effective way to achieve guest release from cages, as we mentioned above^40^. Given that the main difference in guest encapsulation between **Ag**_**5**_**L**_**2**_ and **Hg**_**5**_**L**_**2**_ is shown in CDCl_2_CDCl_2_, we then set about investigating the possibility of the interconversion of **Ag**_**5**_**L**_**2**_ **⇆ Hg**_**5**_**L**_**2**_ in CDCl_2_CDCl_2_ (Fig. 7a).

The strength of the association between Hg^2+^ cations and ligand **1** is much larger than that for Ag^+^ ones. Therefore, the conversion from **Ag**_**5**_**L**_**2**_ to **Hg**_**5**_**L**_**2**_ can be readily achieved by adding the Hg^2+^ cations to the solution of **Ag**_**5**_**L**_**2**_, as evidenced by the reappearance of **Hg**_**5**_**L**_**2**_ signals in the ^1^H NMR spectrum (Fig. 7b, Supplementary Method 14 and Supplementary Fig. 97). Comparatively, the conversion from **Hg**_**5**_**L**_**2**_ to **Ag**_**5**_**L**_**2**_ is much more complicated in methodology. Our attempts in this regard were baffled for a long time until we were drawn to the function of EDTA as the competitive chelating agent. EDTA can form very stable complexes with most metal cations. In particular, in water at 25 °C, the stability constant of Hg^2+^-EDTA (**HgEDTA**) complexes is ca. 14 orders of magnitude higher than that of Ag^+^-EDTA (**AgEDTA**) (log*K* = 21.6 and 7.2 in the case of Hg^2+^ and Ag^+^, respectively)^78^. We thus tried to use the Ag^+^ cations in **AgEDTA** to displace the Hg^2+^ ones in **Hg**_**5**_**L**_**2**_.

In our experiment, an aqueous solution of **AgEDTA** was first prepared by mixing Na_2_EDTA and 1.5 equiv. of AgNO_3_ in water. The solution was transferred to a vial containing a solution of **Hg**_**5**_**L**_**2**_ in CDCl_2_CDCl_2_ and the obtained bilayer mixture was vigorously stirred at 28 °C. To monitor the ion-exchange process, a series of samples taken regularly from the mixture and washed with water was characterized by ^1^H NMR spectroscopy (Supplementary Method 13). As shown in Fig. 7c and Supplementary Fig. 95, as expected, the metal cation-exchanges occurred on the water-CDCl_2_CDCl_2_ interface, giving rise to an immediate disappearance of the signals of **Hg**_**5**_**L**_**2**_ in the organic phase and, finally (ca. 40 min later), the only one set of district signals corresponding to **Ag**_**5**_**L**_**2**_. The exchanges process should be a non-synergistic one: A stepwise ion-exchange is strongly suggested by the loss of *C*_5_-symmetry for the cage in the ^1^H NMR spectrum (can be clearly observed at *t* = 23 and 27 min, Fig. 7c and Supplementary Fig. 95B); and the intermediates, in which the two types of metal ions (Ag^+^ and Hg^2+^) were coordinated to form heterometallic cages, were evidenced by the HR ESI-MS of the sample *t* = 23 min (Fig. 7d, Supplementary Fig. 96 and Supplementary Table 14), which showed a series of intense peaks assignable to mixed-metal cages with different stoichiometries for the composition of cooridnated Ag^+^ and Hg^2+^ cations.

### Metal-cation-and-solvent-gated guest release

**Fig. 8:**
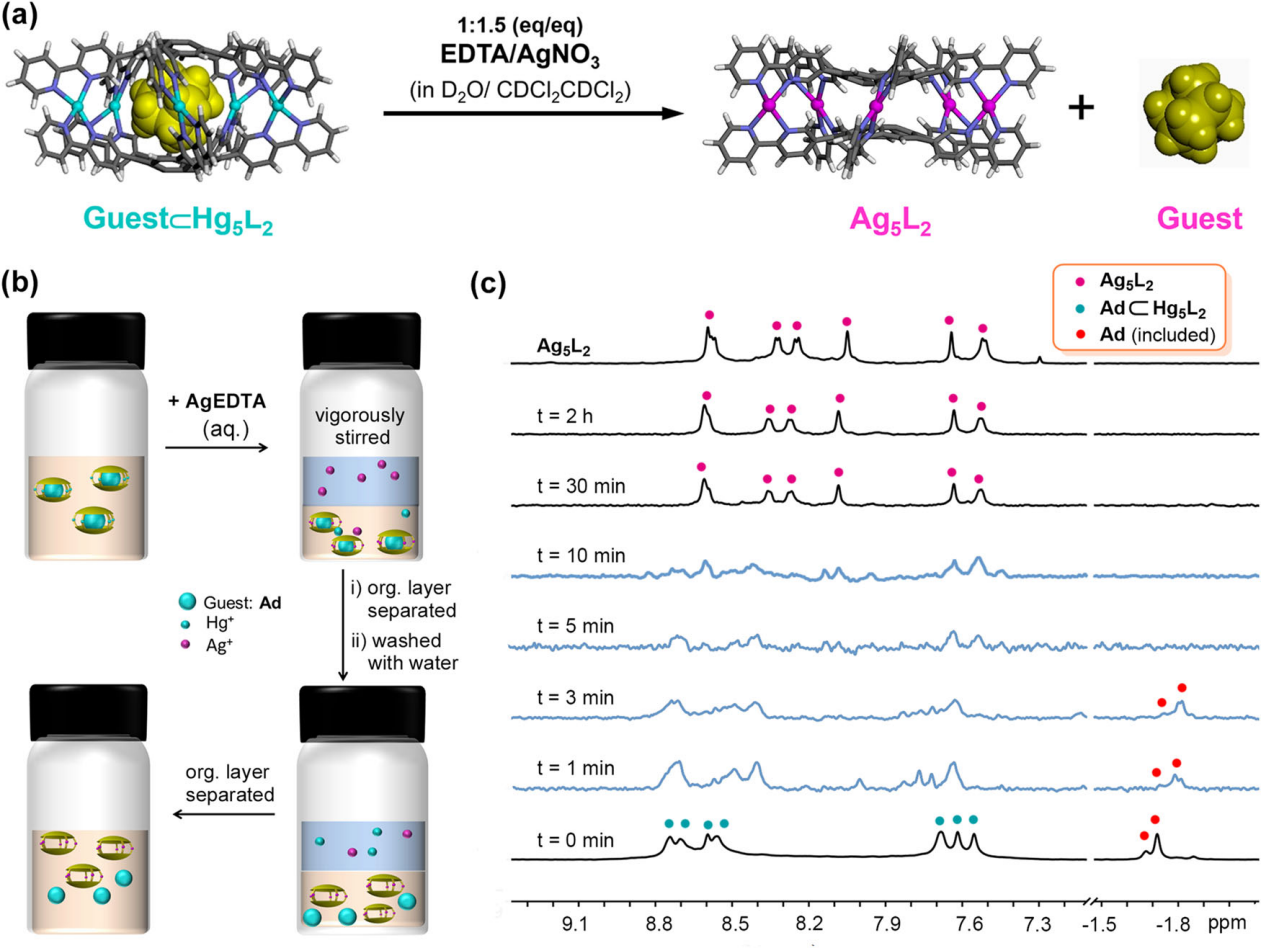
Metal-cation-and-solvent-controlled guest release from Hg_5_L_2_. **a** Schematic showing the metal cation-guided guest release from **Hg**_**5**_**L**_**2**_ in CDCl_2_CDCl_2_. **b** Cartoon representation of the protocol operation for releasing **Ad** from **Ad**⊂**Hg**_**5**_**L**_**2**_ complexes. Initially, an aqueous solution of **AgEDTA** was added to **Hg**_**5**_**L**_**2**_ in CDCl_2_CDCl_2_. The bilayer mixture was vigorously stirred at 28 °C. Samples were taken regularly from the mixture and the organic phase was separated and washed with water. The resulting solutions were further stored at 28 °C for 4 h before being characterized by ^1^H NMR spectroscopy which gave (**c**) a series of ^1^H NMR spectra (600 MHz, 298 K, CDCl_2_CDCl_2_) tracking the cage transformation and the guest release over time. The stirring time on the bilayer mixture of **Ad**⊂**Hg**_**5**_**L**_**2**_ and **AgEDTA** (aq.) is provided in minutes in Fig. 8c.

We finally explored the possibility of the conversion from **Hg**_**5**_**L**_**2**_ to **Ag**_**5**_**L**_**2**_ with the guests included (Fig. 8a). Based on the binding properties of **Ag**_**5**_**L**_**2**_, in CDCl_2_CDCl_2_, such conversion means theoretically a complete guest release from the cages after the obtained mixture is stored at room temperature for, at most, a couple of hours (Supplementary Table 6).

Experimentally, the samples of guest⊂**Hg**_**5**_**L**_**2**_ complexes in acetone-*d*_6_ were first prepared according to **Procedure A**. The free guests were removed, and the solvent was changed to CDCl_2_CDCl_2_, followed by the same transmetallation procedure (Fig. 8b) as that for the conversion from **Hg**_**5**_**L**_**2**_ to **Ag**_**5**_**L**_**2**_ in the absence of guests. A series of samples were taken regularly from the vigorously stirring mixture of **Hg**_**5**_**L**_**2**_ in CDCl_2_CDCl_2_ and aqueous **AgEDTA**. The organic-phase solutions of these samples were washed with water immediately, followed characterizations via ^1^H NMR spectroscopy (Methods, the main text).

Basically, two series of spectra were obtained in each case. The first series of spectra (Supplementary Figs. 98, 100, and 102) were recorded soon after the samples were sucked out of the bilayer mixture and washed with water, which was used to detect the species in the organic-phase solution of the stirring bilayer mixture; and another series of spectra (Fig. 8c, Supplementary Figs. 99, 101, and 103) were recorded after these samples were stored further at 28 °C for 4 h (to get fully released for the guests).

The results showed that the cation-exchanges indeed occurred in the vigorously stirring mixture to give guest⊂**Ag**_**5**_**L**_**2**_ complexes. However, we were surprised to observe that it was more difficult to liberate the included guests from **Ag**_**5**_**L**_**2**_ cages in the stirring bilayer mixtures (Supplementary Figs. 98, 100, and 102), compared to those in the cases of guest⊂**Ag**_**5**_**L**_**2**_ in pure CDCl_2_CDCl_2_ (Supplementary Table 6 and 7). Taking the case of **Ad** as an example, strong signals of the included **Ad** can still be observed in the ^1^H NMR spectrum even the mixture was stirred for 2 h (Supplementary Fig. 98A). This is probably due to the presence of **HgEDTA** in the systems, binding exohedrally to the silver cages thus hindering the release of the guests. Nevertheless, for the samples those were washed with water (**HgEDTA** removed) and then stored for 4 h, no signals of the included **Ad** were observable, as long as the mixture of **Ad**⊂**Hg**_**5**_**L**_**2**_/**AgEDTA** was stirred at 28 °C for no <5 min (*t* = 5–120 min, Fig. 8c and Supplementary Fig. 99), indicative of a complete **Ad** release from the silver cages in these cases. Notably, it seems that **Ad** can even be released from some heterometallic cages, given that, at *t* = 5 or 10 min, the Hg^2+^ cations in the cages have not been completely replaced by the Ag^+^ ones (Fig. 8c and Supplementary Fig. 99). Similar phenomena were also observed in the cases of **Ad-MeOH** and **Ad-COOH** (Supplementary Figs. 101 and 103). All these demonstrate that the release of the guests from **Hg**_**5**_**L**_**2**_ can be synergistically controlled by metal cations and solvents.

## Conclusion

In summary, we have demonstrated the synthesis, characterization and host–guest chemistry of two 1,3,5,7,9-penta(2,2’-bipyridin-5-yl)corannulene-based coordination cages, **Hg**_**5**_**L**_**2**_ and **Ag**_**5**_**L**_**2**_. Host–guest studies with the cages and three adamantane-based guests (**Ad,** **MeOH** and **Ad-COOH**) revealed that, while the former can encapsulate the guest molecules to form stable host–guest complexes in all four kinds of solvents (or solvent mixture), including acetone-*d*_6_, CD_3_CN, 5:95 (v/v) CD_3_CN/CDCl_3_ and CDCl_2_CDCl_2_, the latter shows a guest encapsulation capability in three kinds of the solvents and a guest-release behavior in CDCl_2_CDCl_2_. The two kinds of coordination cages are interconvertible. Therefore, to release the included guests from **Hg**_**5**_**L**_**2**_ in some solvents, such as acetone-*d*_6_, both the solvent and the metal cations have to be changed. This thus, in fact, represents a dual-controlled guest release system, performing the task only if there are the right metal cations and, at the same time, the right solvent. Such kind of anti-interference release systems may cooperate with a single-controlled guest release system, such as **Ag**_**5**_**L**_**2**_ in the studied case, to find applications in programmable synthesis, in which different reactants or catalysts are sequentially released under rational stimuli.

## Methods

### Stability studies on guest⊂Hg_5_L_2_ complexes in solutions

Three kinds of complexes, including **Ad**⊂[Hg_5_**1**_2_]·[OTf]_10_, **Ad-MeOH**⊂[Hg_5_**1**_2_]·[OTf]_10_, and **Ad-COOH**⊂[Hg_5_**1**_2_]·[OTf]_10_, and four different solvent systems, including (i) CD_3_CN, (ii) acetone-*d*_6_, (iii) 95:5 (v/v) CDCl_3_/CD_3_CN and (iv) CDCl_2_CDCl_2_, were investigated. The complexes in CD_3_CN and in acetone-*d*_6_ were prepared according to **Procedure A** (Supplementary Method 10), and those in 95:5 (v/v) CDCl_3_/CD_3_CN and in CDCl_2_CDCl_2_ were constructed via **Procedure D** (Supplementary Method 11). Since, in all the cases, the **Hg**_**5**_**L**_**2**_-guest complexes were prepared using much excess amount of guest compared to that of the cage, to get a clearer observation on the possible release of the guest from the cage as well as to avoid the possible change of the guest molecules between inside and outside of the cage cavity, after the cage-guest complexes were constructed, the free guest in the obtained mixture was first removed by solvent-washing. Then, decays of the complexes were monitored. Typical procedures for the construction of **Hg**_**5**_**L**_**2**_-guest complexes, the free-guest elimination and the stability studies are as follows.

(a) Construction of **Hg**_**5**_**L**_**2**_-guest complexes and free-guest elimination. To a sample of ligand **1** (2 mM, 0.5 mL) in acetone-*d*_6_, Hg(OTf)_2_ (200 mM, acetone-*d*_6_) was added to give a 1 mM **Hg**_**5**_**L**_**2**_ cage solution. To this solution, 8 equivalents of guest (50 mM, acetone-*d*_6_) was added. The mixture was sonicated at ambient temperature for 30 min, then evacuated under reduced pressure to dryness. The residue was re-dissolved in cyclohexane (4 ml) to give a turbid mixture. This mixture was sonicated at ambient temperature for 10 min and centrifuged, then the top homogenous solution was removed with a pipet. The solvent-washing procedure was repeated for total 5 times. Finally, the obtained solid was evacuated under high vacuum, and then re-dissolved into one of the studied solvents (or solvent mixture). ^1^H NMR spectra of the solids showed that all the free-guests were removed, as evidenced by the disappearance of the free-guest signals which typically showed at *δ* = 1.4 − 2.0 ppm (Supplementary Figs. 71A−79B and 80−82).

(b) Stability studies. The stability of the cage-guest complexes in different solvents (acetone-*d*_*6*_, CD_3_CN, 5:95(v/v) CD_3_CN/CDCl_3_ or CDCl_2_CDCl_2_) was measured by ^1^H NMR spectroscopy. Decays of the complexes can be assessed according to the ratio (based on signal integrals) of the cage-guest complexes to the free cages or guests that reappeared in the spectra. The complexes in the solution were monitored for two days. The obtained spectra are shown in Supplementary Figs. 71A−79B and 80−82, which shown that no guest molecules released from the cage cavity in all the cases. The results can be expressed as shown in Supplementary Table 11.

### Guest⊂Hg_5_L_2_→guest + Ag_5_L_2_ conversion

To ligand **1** (1 equiv), the guest (4 equiv), and Hg(OTf)_2_ (2.5 equiv) in a vial, solvent acetone was added, and the mixture was sonicated at ambient temperature for 30 min. The obtained solution was checked by ^1^H NMR spectroscopy at the end of the sonication to make sure that the host–guest complexes had completely formed. The solution was next transferred to a centrifuge tube, evacuated under reduced pressure to dryness. The solid residue was washed with cyclohexane (HPLC Grade). After the free guest has been confirmed to be completely removed by ^1^H NMR spectroscopy, the host–guest complexes were further dried under high-vacuum and then re-dissolved in CDCl_2_CDCl_2_ (4 ml) to give a 1 mM solution. The solution was transferred to a vial (I.D. 25 mm, 10 ml) with a magnetic stir bar (olive shape, diameter 9 mm, length 15 mm). To this solution, an aqueous solution of (1:1.5, equiv/equiv) Na_2_EDTA/AgNO_3_ (50 mM, 4 ml) was added. The mixture was vigorously stirred (950 rpm) at 28 °C. Samples were taken regularly from the mixture, and washed immediately with brine (×3) and pure water (×2), followed by drying with sodium sulfate. The series of samples were characterized by ^1^H NMR spectroscopy after been diluted twofold with CDCl_2_CDCl_2_.

Basically, two series of spectra were obtained for each case. The first series of spectra (Supplementary Figs. 98A, B, 100A, B and 102A, B) were recorded soon after the samples were sucked out of the bilayer mixture and washed with water, which was used to detect the species in the organic-phase solution of the stirring bilayer mixture; and another series of spectra (Fig. 8c, Supplementary Figs. 99A, B, 101A, B, and 103A, B) were recorded after these samples were stored further at 28 °C for 4 h (to get fully released for the guests). The obtained time-dependent ^1^H NMR spectra showed that Guest⊂[Hg_5_**1**_2_]·[OTf]_10_ has successfully turned into [Ag_5_**1**_2_]·[OTf]_10_ with the guests released at the end of the conversion.

### Supplementary information

Peer Review File

the Supplementary Information

Description of Additional Supplementary Files

Supplementary Data 1

## Data Availability

All data are included in this article, Supplementary Information and Supplementary Data (NMR spectra). The data are available from the corresponding author upon reasonable request.
