# Supplementary material for: Dual-controlled guest release from coordination cages
Source: Commun Chem. 2024 Feb 27;7:43. doi: 10.1038/s42004-024-01128-z (PMC10899651; doi:10.1038/s42004-024-01128-z)
Supplement: Supplementary file 2 — the Supplementary Information [file 42004_2024_1128_MOESM2_ESM.pdf]

Supplementary Information

Dual-controlled Guest Release from Coordination Cages

Yuqing Yao,† Chengyuan Shao,† Shuwei Wang, Qiufang Gong, Jia Liu, Hua Jiang,* and Ying Wang*

College of Chemistry, Beijing Normal University, Beijing 100875, China

†Equal contribution

Email: ywang1@bnu.edu.cn and jiangh@bnu.edu.cn

Supplementary Method 1. General Methods

Throughout the Supplementary Information a large number of chemical structures or chemical equilibriums have been used to schematically illustrate the labelling of the protons or the conversions between different cage species. Notably, unless indicated otherwise, these structures are used to show only the species of chemicals; no information about configuration/conformation is involved).

Anhydrous tetrahydrofuran (THF) was distilled over sodium benzophenone under argon. Other solvents were obtained from commercial sources. Per-deuterated solvents for NMR spectroscopy were obtained from Cambridge Isotope Laboratories and used as received. All the commercially available chemicals were obtained from either Alfa Aesar, Aldrich or Acros and used without further purification, unless indicated otherwise.

Analytical TLC was carried out using tapered silica plates with a preadsorbent zone. Crude compounds were purified by flash column chromatography, using flash grade silica gel and 0 – 20 psig pressure, or a Chromatotron centrifugal thin-layer chromatograph (Model 7924T, T-Squared Technology, Inc.), performed at ambient pressure.

Structural assignments were made with additional information from gCOSY, gHSQC, and gHMBC experiments. NMR spectra were obtained with a Bruker spectrometer or a JEOL Delta spectrometer (^{1}H, 400 and 600 MHz) using chloroform-*d* ($CDCl_3$), acetone-*d*$_6$ ($(CD_3)_2CO$), acetonitrile-*d*$_3$ (CD_3CN), 1,1,2,2-tetrachloroethane-*d*$_2$ ($CDCl_2CDCl_2$) or the mixture of chloroform-*d* and acetonitrile-*d*$_3$ as solvent. The chemical shift references were as follows: (^{1}H) chloroform, 7.26 ppm (chloroform-*d*), (^{13}C) chloroform-*d*, 77.00 ppm (chloroform-*d*); (^{1}H) acetone-*d*$_6$, 2.05 ppm (acetone-*d*$_5$), (^{13}C) acetone-*d*$_6$, 29.84 ppm and 206.26 ppm (acetone-*d*6); (^{1}H) acetonitrile-*d*$_2$, 1.94 ppm (acetonitrile-*d*$_3$), (^{13}C) acetonitrile-*d*$_3$, 118.26 ppm (acetonitrile-*d*$_3$); (^{1}H) 1,1,2,2-tetrachloroethane-*d*$_2$, 6.00 ppm (1,1,2,2-tetrachloroethane-*d*), (^{13}C) 1,1,2,2-tetrachloro-ethane-*d*$_2$, 73.8 ppm (1,1,2,2-tetrachloroethane-*d*$_2$). For solvent mixtures, TMS was used as the internal standard: (^{1}H) tetramethylsilane (TMS), 0.00 ppm (the mixture of chloroform-*d* and acetonitrile-*d*$_3$). Typical 1D FID was subjected to exponential multiplication with a line broadening exponent (LB) of 0.3 Hz (for ^{1}H) and 1.0 Hz (for ^{13}C). 2D NOESY was recorded with the mixing time of 300 ms. Mass spectra (ESI) were acquired on GCT spectrometer (Bruker Daltonics Inc).

Supplementary Method 2. General Methodology for the Synthesis of Ligand **1**

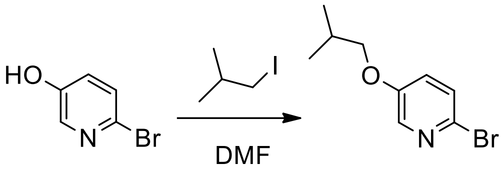

2-Bromo-5-*iso*-butoxypyridine (2).[S1] To a solution of 5-bromopyridin-2-ol (3.0 g, 17.24 mmol, 1.0 equiv) in DMF (15 mL), 1-iodo-2-methylpropane (2.2 mL, 18.96 mmol, 1.1 equiv) and K_2CO_3 (3.57 g, 25.86 mmol, 1.5 equiv) was added. The suspension mixture was stirred at 60 °C for overnight and then poured into pure water, then extracted with ethyl acetate. The organic layer was washed with brine for more than 5 times to remove the remained DMF, dried over sodium sulfate and concentrated in vacuo. The residue was purified on silica gel flash column chromatography (ethyl acetate/hexane, 1:15) to give the product **2** as pale yellow oil (1.98 g, yield: 50%). Mp. (under air): 122.0−123.5 °C. R_f = 0.4 (ethyl acetate/hexane, 1:20). ^{1}H NMR (400 MHz, $CDCl_3$): δ = 8.03 (dd, J = 9.3, 2.9 Hz, 1H), 7.35 (d, J = 8.7 Hz, 1H), 7.08 (dd, J = 8.7, 3.1 Hz, 1H), 3.74 (d, J = 6.5 Hz, 2H), 2.13 – 2.0 (m, 1H), 1.03 (d, J = 6.7 Hz, 6H). ^{13}C NMR (151 MHz): δ = 155.3, 137.7, 132.0, 128.2, 125.0, 75.3, 28.4, 19.2. IR (KBr, cm^{-1}): 2960.9, 2930.0, 2874.1, 1724.4, 1577.8, 1564.3, 1452.5, 1381.1, 1367.6, 1276.9, 1224.9, 1132.3, 1091.8, 1022.3, 821.7, 648.1, 418.6. TOF-HRMS-ESI: Calcd for $C_8H_{11}BrNO_2$ at $[M+H]^+$: 230.0181 (100%), 232.0160 (97.44%); Found: 230.0178 (100.0%, 1.3 ppm), 232.0156 (97.9%, 2.6 ppm).

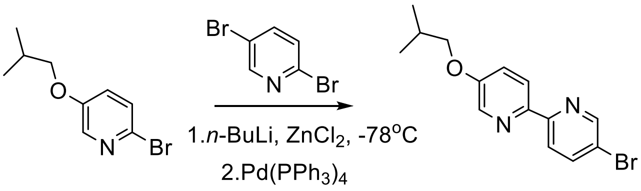

5-bromo-5'-*iso*butoxy-2,2'-bipyridine (3).[S2] To a solution of 2-bromo-5-*iso*butoxy-pyridine (1.15 g, 5 mmol, 1.0 equiv) in anhydrous THF (30 mL), a solution of *n*-BuLi (1.60 M in hexane, 3.2 mL, 5.5 mmol, 1.10 equiv) was added dropwise under argon at –78 °C in over 15 min. The reaction mixture was allowed to stir at –78 °C for 1 hour and then a solution of dry $ZnCl_2$ (0.82 g, 5.5 mmol, 1.10 equiv) in anhydrous THF (10 mL) was added *via* cannula. Upon warming to room temperature, the resulting aryl zincate solution was cannulated into a solution of 2,5-dibromopyridine (1.25 g,

5.25 mmol, 1.05 equiv) and $Pd(PPh_3)_4$ (35 mg, 0.15 mmol, 0.03 equiv) in anhydrous THF (20 mL). The reaction mixture was heated to reflux for 18 h and then allowed to cool to room temperature. The resulting white precipitate was filtered off, washed with THF, and dried under reduced pressure. The dry precipitate was then suspended in a mixture of CH_2Cl_2 and a basic aqueous solution of EDTA (basified using $NaHCO_3$) and stirred until all precipitate dissolved. The organic phase was separated, washed with brine and water, dried over $MgSO_4$ and concentrated. The residue was purified on silica gel flash column chromatography (ethyl acetate/hexane, 2:100) to give the target product (460 mg, 32%) as a white solid. Mp. (under air): 54.5−56.0 °C. R_f = 0.3 (ethyl acetate/hexane, 1:8). ^{1}H NMR (400 MHz, $CDCl_3$): δ = 8.67 (d, J = 2.3 Hz, 1H), 8.34 (d, J = 2.9 Hz, 1H), 8.29 (d, J = 8.8 Hz, 1H), 8.22 (d, J = 8.5 Hz, 1H), 7.89 (dd, J = 8.5, 2.3 Hz, 1H), 7.29 (dd, J = 8.8, 2.9 Hz, 1H), 3.83 (d, J = 6.5 Hz, 2H), 2.19 – 2.08 (m, 1H), 1.05 (t, J = 8.3 Hz, 6H). ^{13}C NMR (151 MHz): δ = 156.1, 154.7, 150.0, 147.7, 139.4, 137.6, 121.7, 121.7, 121.4, 120.1, 74.9, 28.4, 19.2. IR (KBr, cm^{-1}): 2957.0, 2924.2, 2872.1, 2852.8, 1564.3, 1547.0, 1450.5, 1365.7, 1282.7, 1257.6, 1230.6, 1136.1, 1024.2, 1003.0, 825.6, 731.1, 634.6. TOF-HRMS-ESI: Calcd for $C_{14}H_{16}N_2OBr$ at $[M+H]^+$: 307.0446 (100%), 309.0426 (97%), 304.0459 (17%), found: $[M+H]^+$: 307.0442 (100%, 0.5 ppm), 309.0422 (96%, 1.3 ppm), 310.0463 (32%, 1.3 ppm).

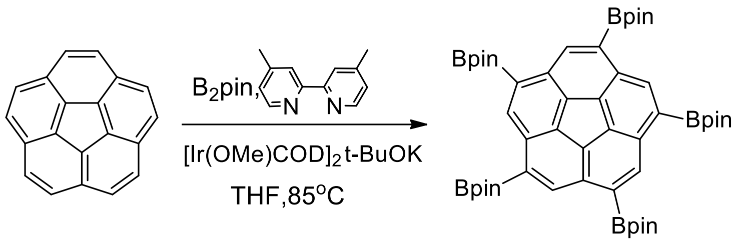

1,3,5,7,9-Pentakis(bpin)corannulene (4).[S3] To a 15 mL flame dried pressure vessel equipped with a rubber septum, $[Ir(OMe)COD]_2$ (53 mg, 0.8 mmol), 4,4'-dimethyl-2,2'-bipyridyl (30 mg, 0.16 mmol), B_2pin_2 (528 mg, 2.08 mmol), and potassium *t*-butoxide (5 mg, 0.04 mmol) was added. The vessel was charged with argon, followed the addition of anhydrous THF (0.4 mL) by syringe. The mixture in the vessel was heated at 50 °C for 10 min, and a dark brown-red solution formed. Under the flowing of argon, corannulene (100 mg, 0.4 mmol) was added to the vessel. The vessel was charged with argon for 3 times, and then the rubber septum was replaced with Teflon stopper in an argon bag. The mixture in the sealed vessel was stirred at 85 °C for 3 days, then cooled down to ambient temperature and immediately diluted with dichloromethane (~3 mL). The reaction was then

quenched by the dropwise addition of 10% HCl (~3 mL). The layers were separated, and the aqueous layer was thoroughly extracted with dichloromethane. The combined organic layers were washed with water (×1) and brine (×1), and dried over magnesium sulfate. The filtrate was concentrated to dryness on a rotavapor to give a dark red-brown oily semi-solid mixture. Methanol (15~20 mL) was added to the flask, and the mixture was sonicated for 5 min. The precipitate was collected by vacuum filtration with a small Buchner funnel, and the precipitate was washed with methanol (2 × 10 mL) to provide the target compound (282 mg, 80%). ^{1}H NMR (400 MHz, $CDCl_3$): δ = 8.97 (s, 5H), 1.46 (s, 60H). ^{13}C NMR (151 MHz): δ = 139.0, 137.0, 133.3, 83.8, 25.2. IR: 2976.3, 1922.3, 2850.9, 1628.0, 1448.6, 1369.5, 1298.2, 1267.3, 1141.9, 985.7, 854.5, 721.2, 657.8. (*Lit.*[S3] *^{1}H NMR (500 MHz, $CDCl_3$): δ = 8.98 (s, 5H), 1.46 (s, 60H); ^{13}C NMR (125 MHz, $CDCl_3$): δ = 138.9, 137.0, 133.3, 128.6 (broad) 83.8, 25.2.)*

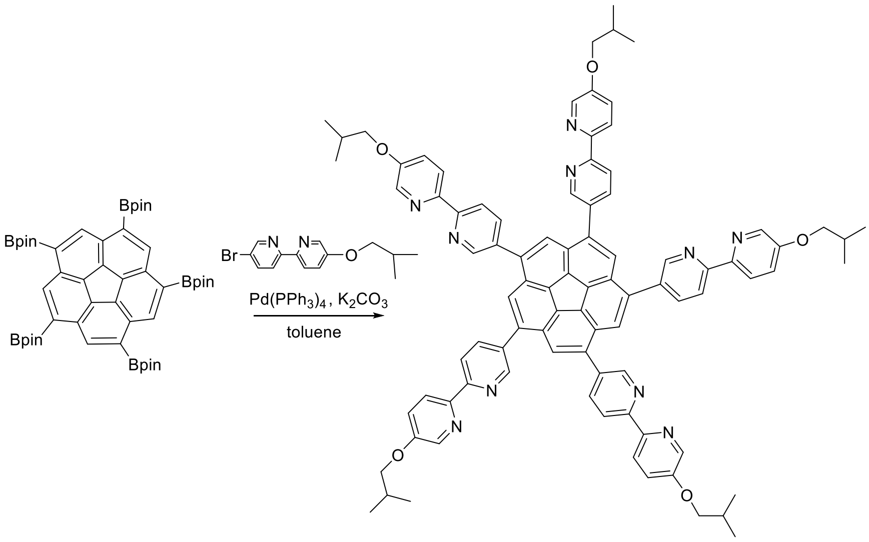

1,3,5,7,9-Pentakis(5-bromo-5'-isobutoxy-2,2'-bipyridine) corannulene (1).[S4] Degassed CH_3OH (2 ml) and H_2O (2 ml) was added to a solution of **3** (140mg,0.5 mmol), $Pd(PPh_3)_4$ (45 mg, 3 mol%), 1,3,5,7,9-pentakis(Bpin) corannulene (44 mg, 0.05mmol), sodium carbonate (138 mg, 2.3 mmol) in degassed toluene (5 ml). The mixture was heated at 95 °C for 3 days. The reaction was cooled to room temperature and extracted with dichloromethane (50 ml × 3) and dried under vacuum. The solid mixture was washed with hexane, CH_3OH and dichloromethane to obtain the target product as light-yellow solid (48 mg, 68%). Mp. (under air): 188.0−190.0 °C. R_f = 0.3 (dichloromethane/ CH_3OH /triethylamine, 50:1:1). ^{1}H NMR (400 MHz, $CDCl_3$): δ = 8.99 (t, J = 7.3 Hz, 1H), 8.48 (d, J = 8.2 Hz, 1H), 8.41 (d, J = 8.6 Hz, 2H), 8.14 (dd, J = 8.2, 2.0 Hz, 1H), 8.06 – 7.95 (s, 1H), 7.33 (dd, J = 8.8, 2.8 Hz, 1H), 3.83 (t, J = 14.2 Hz, 2H), 2.20 – 2.09 (m, 1H), 1.06 (t, J = 9.6 Hz, 6H). ^{13}C

NMR (100 MHz, $CDCl_3$): δ = 156.1, 155.7, 149.7, 148.2, 139.4, 138.0, 137.6, 136.1, 133.9, 129.4, 126.3, 122.0, 121.6, 120.5, 74.9, 28.4, 19.3. IR: 2958.9, 2924.2, 2872.1, 2854.8, 1585.6, 1574.0, 1464.0, 1435.1, 1390.7, 1263.4, 1222.9, 1020.4, 831.4, 408.9. TOF-HRMS-ESI: Calcd for $C_{90}H_{80}N_{10}O_5$ at $[M+H]^+$: 1381.6391 (100%), 1382.6425 (97%); found: 1381.6410 (84%,1.8 ppm), 1382.6436 (100%, 0.8 ppm).

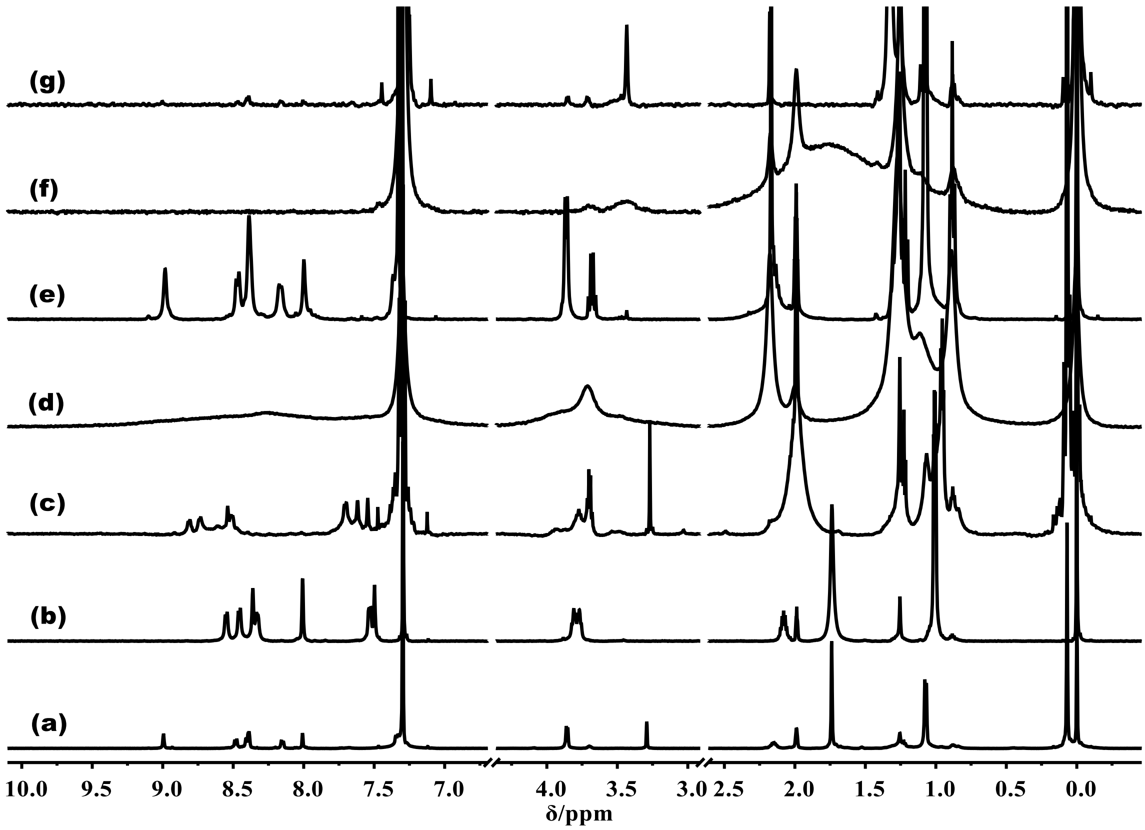

Supplementary Figure 1. ^{1}H NMR (600 MHz, 298 K) of ligand **1** (2.0 mM) in the presence of 2.5 equiv of (a) AgOTf; (b) $Hg(OTf)_2$; (c) $Fe(OTf)_2$; (e) $Mg(OTf)_2$; (f) $NiClO_4$ (g) $Zn(OTf)_2$ in 95:5 (v/v) $CDCl_3$/CD_3CN.

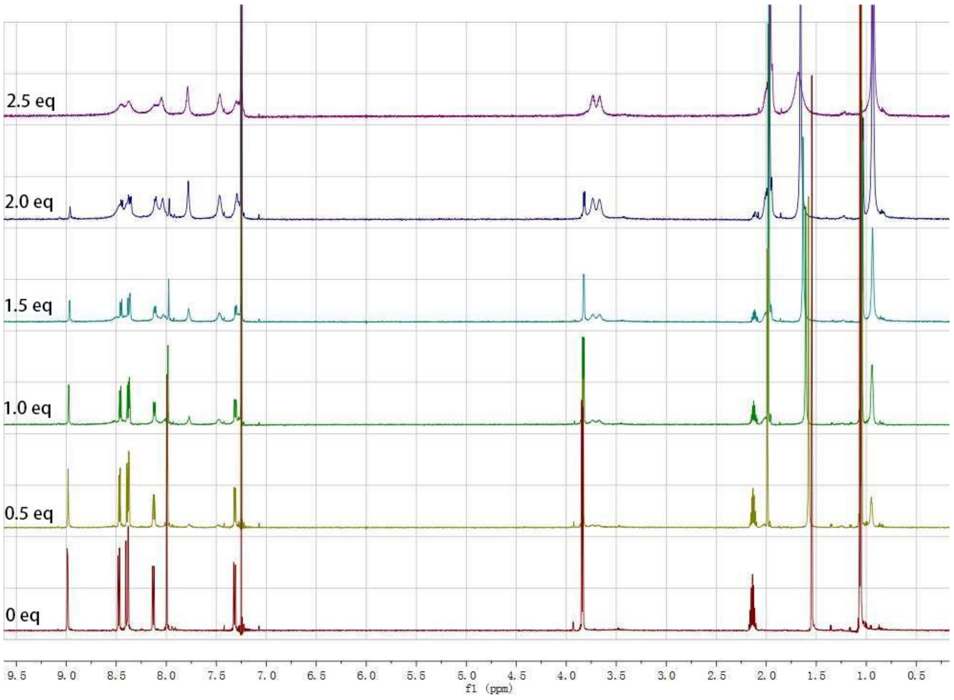

Supplementary Figure 2. ^{1}H NMR titration of **1** (2.0 mM, $CDCl_3$, 0.5 mL) with $Cu(CH_3CN)_4PF_6$ (50 mM, CD_3CN). The signals of the resultant complexes disappeared after the solutions were stored at room temperature for 3 h.

Supplementary Method 3. ^{1}H NMR Titration on Ligand **1** with Ag^+ Cations

The experiments were carried out by a gradual addition of a concentrated solution of silver triflate (AgOTf, 50 mM) in CD_3CN to the ligand **1** (2 mM, 0.5 mL) in $CDCl_3$. In this process, a serial of ^{1}H NMR spectra of the sample was recorded by a JEOL 600 MHz (^{1}H) spectrometer at 298 K.

The assignments of the ^{1}H NMR spectra of the ligand **1** were performed based on the corresponding ^{1}H−^{1}H COSY, ^{1}H−^{13}C HSQC, ^{1}H−^{13}C HMBC and ^{1}H−^{1}H NOESY experiments (see Supplementary Figures 14−17); and those for the complexes were assigned based on the corresponding ^{1}H−^{1}H COSY and ^{1}H−^{1}H NOESY spectra (see Supplementary Figures 18−19).

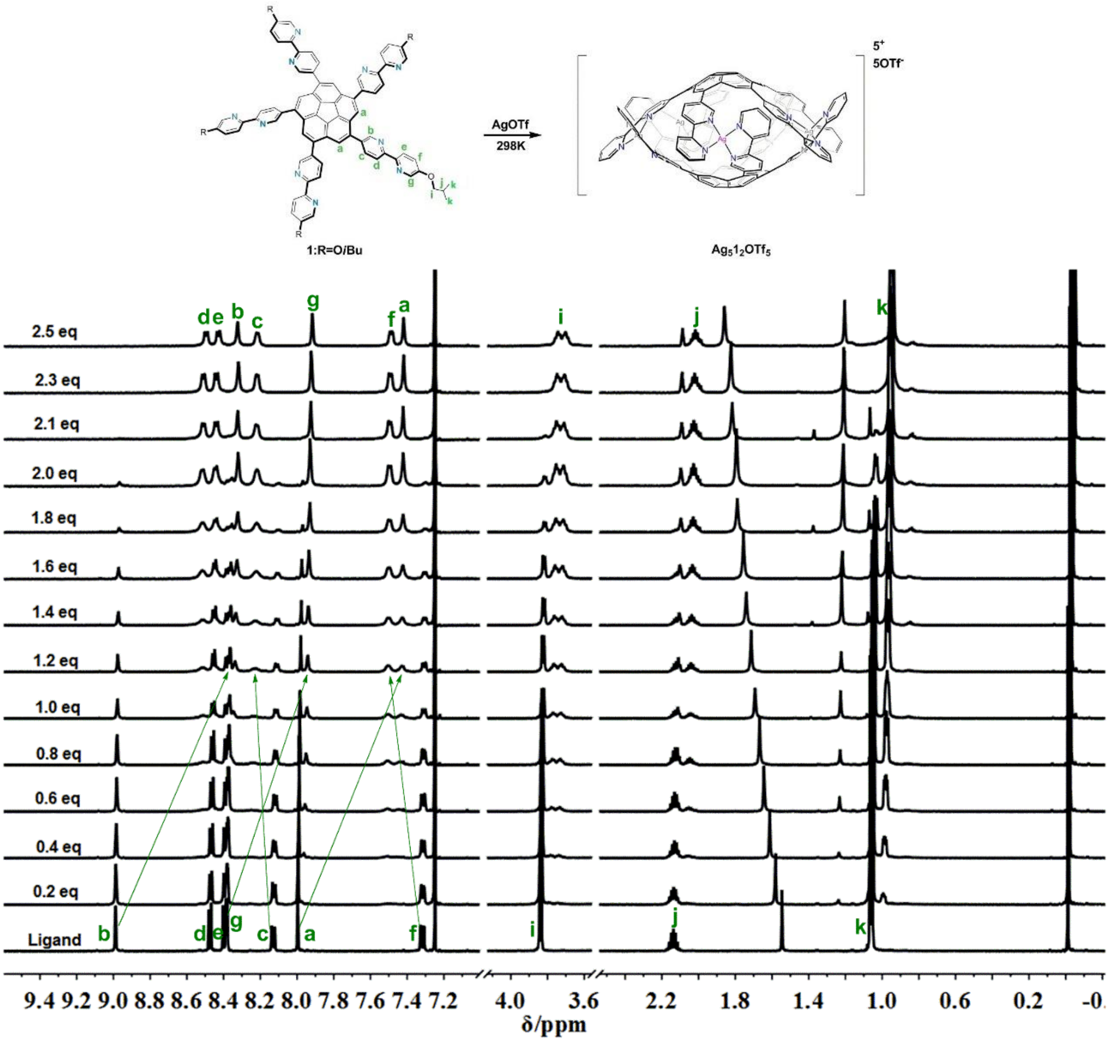

Supplementary Figure 3. ^{1}H NMR (600 MHz, 298 K) titration of the ligand **1** (2.0 mM) in $CDCl_3$ with AgOTf in CD_3CN (final 95:5 (v/v) $CDCl_3/CD_3CN$).

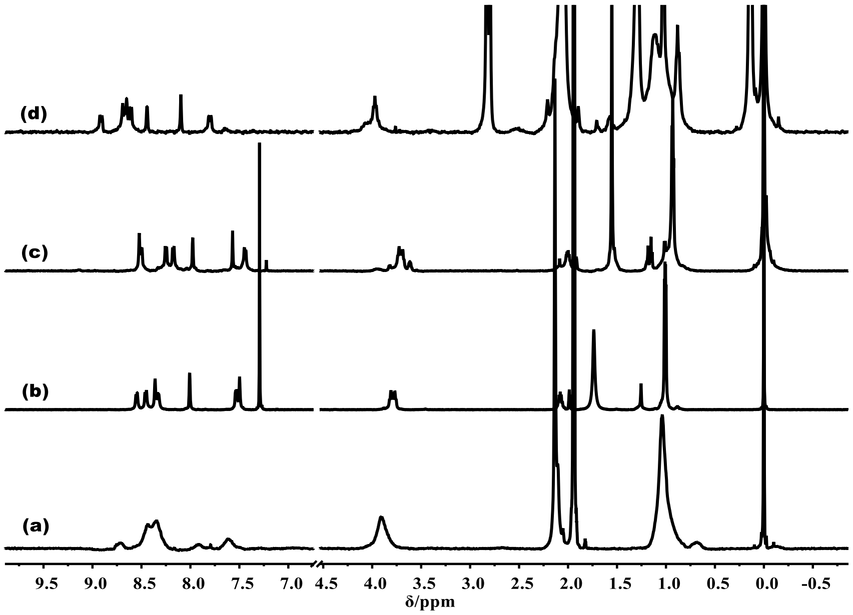

Supplementary Figure 4. ^{1}H NMR (600 MHz, 298 K) of $[Ag_5\mathbf{1}_2]\cdot[OTf]_5$ (**Ag_5L_2**) (1 mM) in (a) CD_3CN; (b) 95:5 (v/v) $CDCl_3/CD_3CN$; (c) $CDCl_2CDCl_2$; (d) acetone-d_6.

Supplementary Method 4. Encapsulation of **Ad** and its Derivatives in 95:5 (v/v) $CDCl_3/CD_3CN$

^{1}H NMR Titration Experiments. The cage samples were prepared by addition of a concentrated solution of silver triflate (AgOTf, 50 mM) in CD_3CN to ligand **1** (2 mM, 0.5 mL) in $CDCl_3$ to give 1 mM cage molecules in 95:5 (v/v) $CDCl_3/CD_3CN$. To this solution, a concentrated solution of guest in 95:5 (v/v) $CDCl_3/CD_3CN$ was gradually added. In this process, a serial of ^{1}H NMR spectra of the sample was recorded by a 600 MHz (^{1}H) spectrometer at 298 K. Three kinds of guest molecules, including adamantane (**Ad**), 1-admethanol (**Ad-MeOH**), and 1-adamantanecarboxylic acid (**Ad-COOH**) were investigated in the experiments.

Association constants (K_a). Determination of K_a were based on the corresponding ^{1}H NMR guest titration spectra, using Supplementary Eq. (S1) shown as below,

$$K_a = \frac{c_{capsule}}{c_{cage,free} \cdot [c_{G,free}]} = \frac{c_{capsule}}{c_{cage,free} \cdot (c_{G,\,total} - c_{capsule})} \qquad \text{(S1)}$$

where, $c_{capsule}$: concentration of the capsule with guest encapsulated;

$c_{cage,free}$: concentration of the free cage molecules in the solution;

$c_{G,free}$: concentration of the free guest molecules in the solution;

$c_{G,total}$: concentration calculated based on the accumulated amount of the guest added.

The concentration of the free cage and the capsule with guest encapsulated are obtained from

the initial concentration ($c_{cage,init}$ = 1 mM) of the cage and the molar ratio (χ) of $c_{capsule}$ against $c_{cage,free}$, calibrated with a coefficient (a) that represents the change in total volume (V_T) of the solution arising from the addition of guest solution (accumulated volume, $V_{G,\ accum}$), $a = V_T/(V_T + V_{G,\ accum})$.

$$c_{cage,free} = a\ C_{cage,init} \cdot \chi \qquad \text{(S2)}$$

$$c_{capsule} = a\ C_{cage,init} \cdot (1 - \chi) \qquad \text{(S3)}$$

wherein χ were derived from the ratio of the integration of the signals located at ca. *8.02 ppm* (corresponding to the free cage) against to that at ca. *7.90 ppm* (corresponding to the cage with guest encapsulated). To reduce the systematical errors, only parts of the spectra were used for the calculations. Summary of the obtained data are shown in Supplementary Table 1.

Supplementary Table 1. Summary of the Calculations on the Association Constant of $[Ag_5\mathbf{1}_2]\cdot[OTf]_5$ with **Ad**, **Ad-MeOH** and **Ad-COOH** in 95:5 (v/v) $CDCl_3/CD_3CN$.[a]

Guest	Equiv	χ [b]	$c_{capsule}$ (mM)	$c_{cage,free}$ (mM)	$c_{G,free}$ (mM)	K_a (M^{-1})	Mean (K_a) (M^{-1})	σ [c]
**Ad**	10	0.21	0.17	0.83	9.83	21.37	23.36	3.16
	20	0.43	0.30	0.70	19.70	21.83		
	30	0.65	0.39	0.61	29.61	21.95		
	40	0.89	0.47	0.53	39.53	22.52		
	50	1.11	0.53	0.47	49.47	22.44		
	60	1.31	0.57	0.43	59.43	22.04		
	70	1.53	0.60	0.40	69.40	22.05		
	80	1.85	0.65	0.35	79.35	23.31		
	90	1.99	0.67	0.33	89.33	22.28		
	100	2.45	0.71	0.29	99.29	24.68		
	110	3.55	0.78	0.22	109.22	32.50		
**Ad-MeOH**	10	0.24	0.19	0.81	9.81	24.47	21.67	1.58
	20	0.45	0.31	0.69	19.69	22.85		
	30	0.58	0.37	0.63	29.63	19.57		
	40	0.95	0.49	0.51	39.51	24.04		
	50	0.98	0.49	0.51	49.51	19.80		
	60	1.28	0.56	0.44	59.44	21.53		
	70	1.52	0.60	0.40	69.40	21.90		
	80	1.67	0.63	0.37	79.37	21.04		
	90	1.83	0.65	0.35	89.35	20.48		

	100	2.13	0.68	0.32	99.32	21.45		
	110	2.32	0.70	0.30	109.30	21.23		
**Ad-COOH**	20	0.6	0.38	0.63	19.63	30.57	22.30	4.68
	30	0.83	0.45	0.55	29.55	28.09		
	40	0.98	0.49	0.51	39.51	24.81		
	50	1.12	0.53	0.47	49.47	22.64		
	60	1.25	0.56	0.44	59.44	21.03		
	70	1.38	0.58	0.42	69.42	19.88		
	80	1.5	0.60	0.40	79.40	18.89		
	90	1.6	0.62	0.38	89.38	17.90		
	100	1.68	0.63	0.37	99.37	16.91		

[a] Calculations were carried out based on Supplementary Eq. S1. The raw data were derived from the ^{1}H NMR titration experiment shown in Supplementary Figures 5−7. [b] Molar ratio (χ) of c_{caps} against $c_{cage,free}$. [c] Standard deviation of the mean K_a.

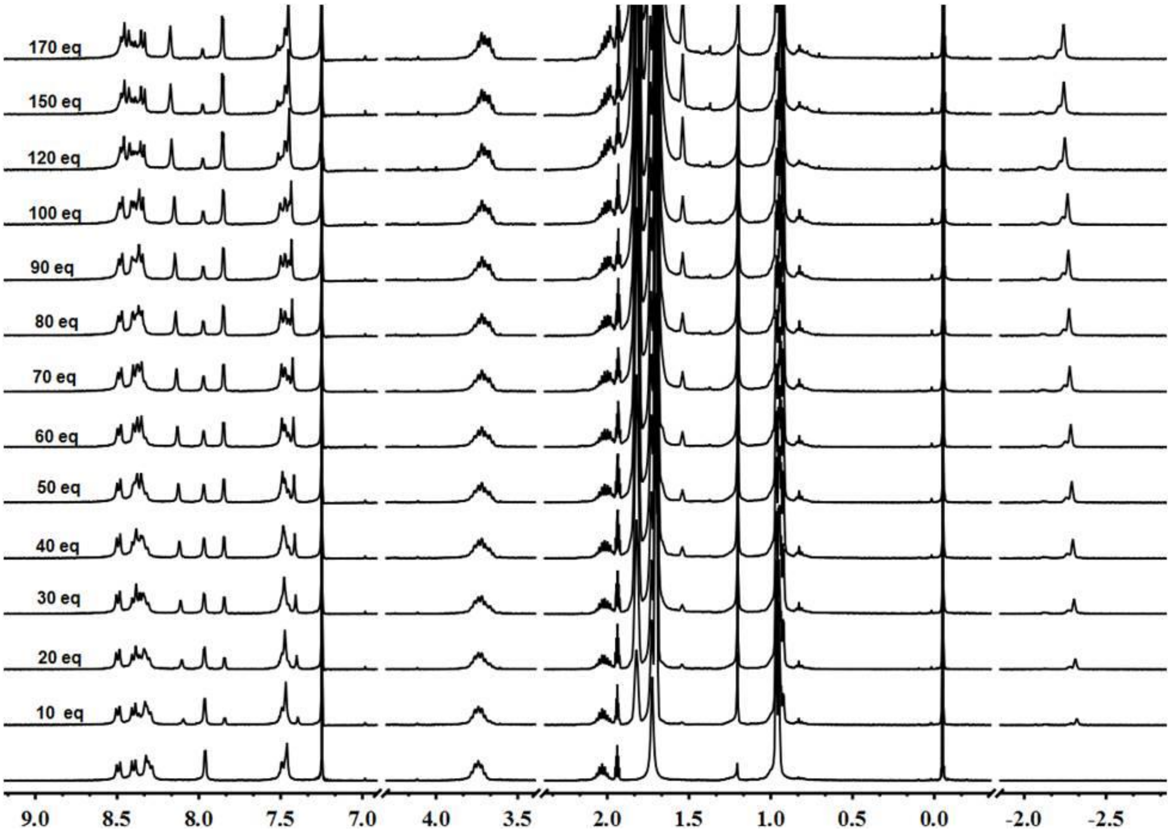

Supplementary Figure 5. Changes of ^{1}H NMR spectra (600 MHz, 298 K) of $[Ag_5\mathbf{1}_2]\cdot[OTf]_5$ (**Ag_5L_2**) (1 mM) in 95:5 (v/v) $CDCl_3/CD_3CN$ upon the addition of **Ad**.

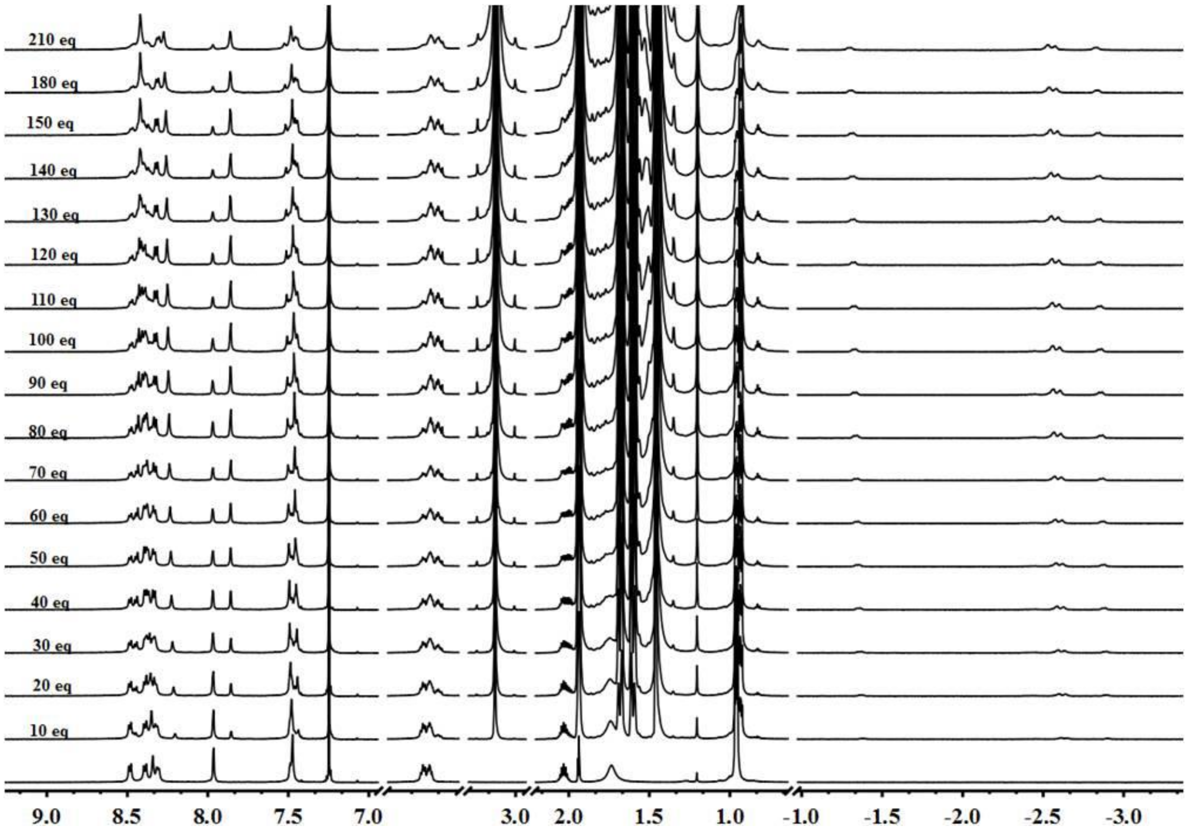

Supplementary Figure 6. Changes of ^{1}H NMR spectra (600 MHz, 298 K) of $[Ag_5\mathbf{1}_2]\cdot[OTf]_5$ (**Ag_5L_2**) (1 mM) in 95:5 (v/v) $CDCl_3/CD_3CN$ upon the addition of **Ad-MeOH**.

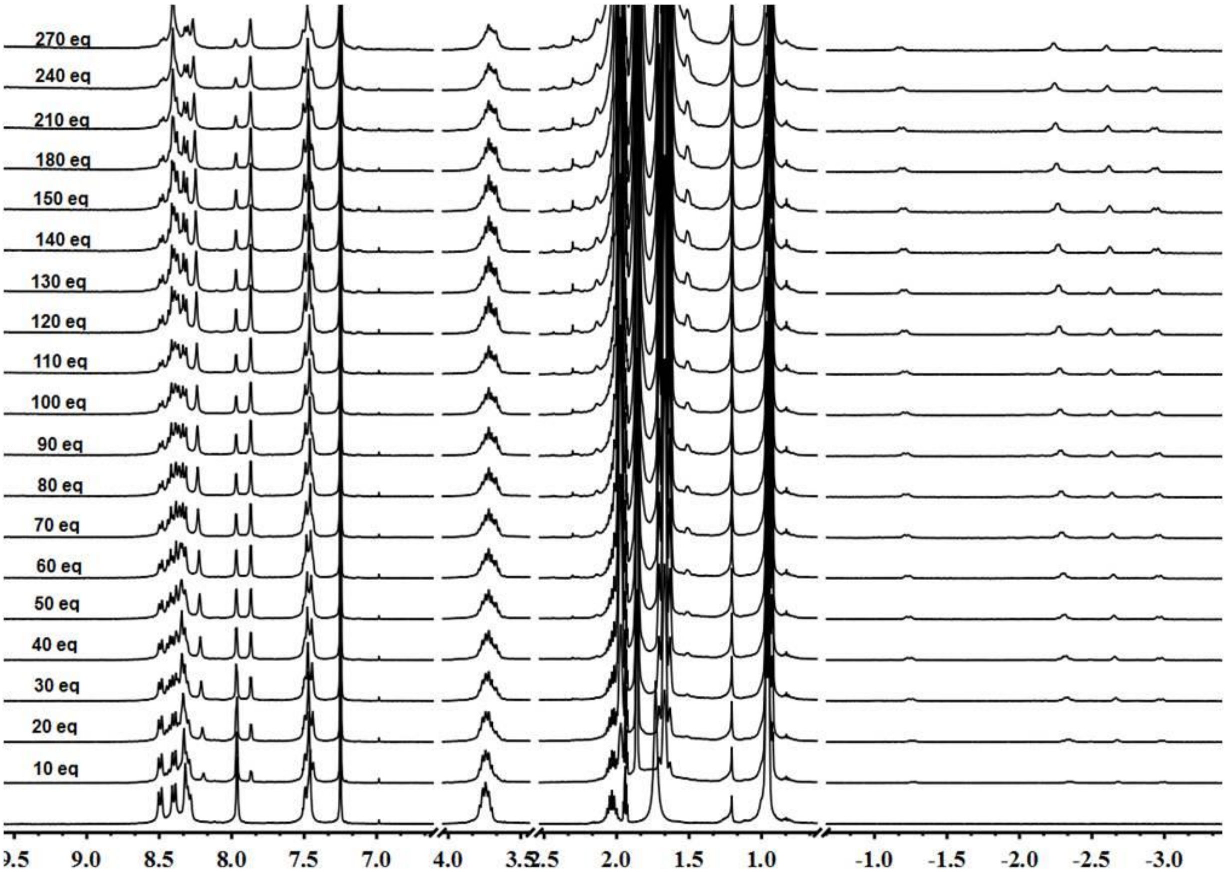

Supplementary Figure 7. Changes of ^{1}H NMR spectra (600 MHz, 298 K) of $[Ag_5\mathbf{1}_2]\cdot[OTf]_5$ (**Ag_5L_2**) (1 mM) in 95:5 (v/v) $CDCl_3/CD_3CN$ upon the addition of **Ad-COOH**.

Supplementary Method 5. Encapsulation of **Ad** and Its Derivatives in Acetone-d_6

^{1}H NMR Titration Experiments. Sample of $[Ag_5\mathbf{1}_2]\cdot[OTf]_5$ was prepared by mixing **1** with 2.5 equivalents of Ag(OTf), using 93:7 (v/v) $CDCl_3/CD_3CN$ as the solvent, followed by the evaporation of the solvent under reduced pressure and the redissolution of the residue into acetone-d_6. To the cage solution, a concentrated solution of the guest in acetone-d_6 was gradually added. In this process, a serial of ^{1}H NMR spectra of the sample was recorded by a 400 MHz (^{1}H) spectrometer at 298 K. **Ad**, **Ad-MeOH** and **Ad-COOH** were investigated as the guests in the experiments.

The ^{1}H NMR spectra of the guest-encapsulated capsule were assigned based on the signal patterns as well as the ^{1}H–^{1}H COSY, ^{1}H–^{13}C HSQC, ^{1}H–^{13}C HMBC and ^{1}H–^{1}H NOESY spectra of cage-guest complexes (see Supplementary Figures 20−23).

Association constants (K_a). K_a of the cage for the studied guests in acetone-d_6 were basically determined using Supplementary Eq. (S1) shown above. Summary of the obtained results are shown in Supplementary Table 2.

Supplementary Table 2. Summary of the Calculations on the Association Constant of $[Ag_5\mathbf{1}_2]\cdot[OTf]_5$ with **Ad**, **Ad-MeOH** and **Ad-COOH** in acetone-d_6.[a]

Guest	Equiv	χ [b]	$c_{capsule}$ (mM)	$c_{cage,free}$ (mM)	$c_{G,free}$ (mM)	K_a ($\times10^3$ M^{-1})	Mean (K_a) ($\times10^3$ M^{-1})	σ [c] ($\times10^3$ M^{-1})
**Ad**	1.0	0.51	0.17	0.33	0.33	1.54	2.1	0.78
	2.0	0.96	0.24	0.26	0.76	1.27		
	3.0	2.47	0.36	0.14	1.14	2.16		
	4.0	3.58	0.39	0.11	1.61	2.22		
	5.0	6.79	0.44	0.06	2.06	3.29		
**Ad-MeOH**	0.4	0.39	0.14	0.36	0.06	6.53	6.8	1.45
	0.6	0.69	0.20	0.30	0.10	7.20		
	0.8	0.73	0.21	0.29	0.19	3.86		
	1.0	1.55	0.30	0.20	0.20	7.91		
	1.2	2.13	0.34	0.16	0.26	8.20		
	1.4	2.22	0.34	0.16	0.36	6.25		
	1.6	3.10	0.38	0.12	0.42	7.35		
**Ad-COOH**	1.0	0.24	0.10	0.40	0.40	5.95	9.1	2.52
	2.0	0.57	0.18	0.32	0.82	6.96		
	3.0	1.25	0.28	0.22	1.22	10.23		
	4.0	1.95	0.33	0.17	1.67	11.68		
	5.0	2.33	0.35	0.15	2.15	10.84		

[a] Calculations were carried out based on Supplementary Eq. S1 listed in Page S9. The raw data were derived from the ^{1}H NMR titration experiment shown in Supplementary Figures 8−10. [b] Molar ratio (χ) of c_{caps} against $c_{cage,free}$. [c] Standard deviation of the mean K_a.

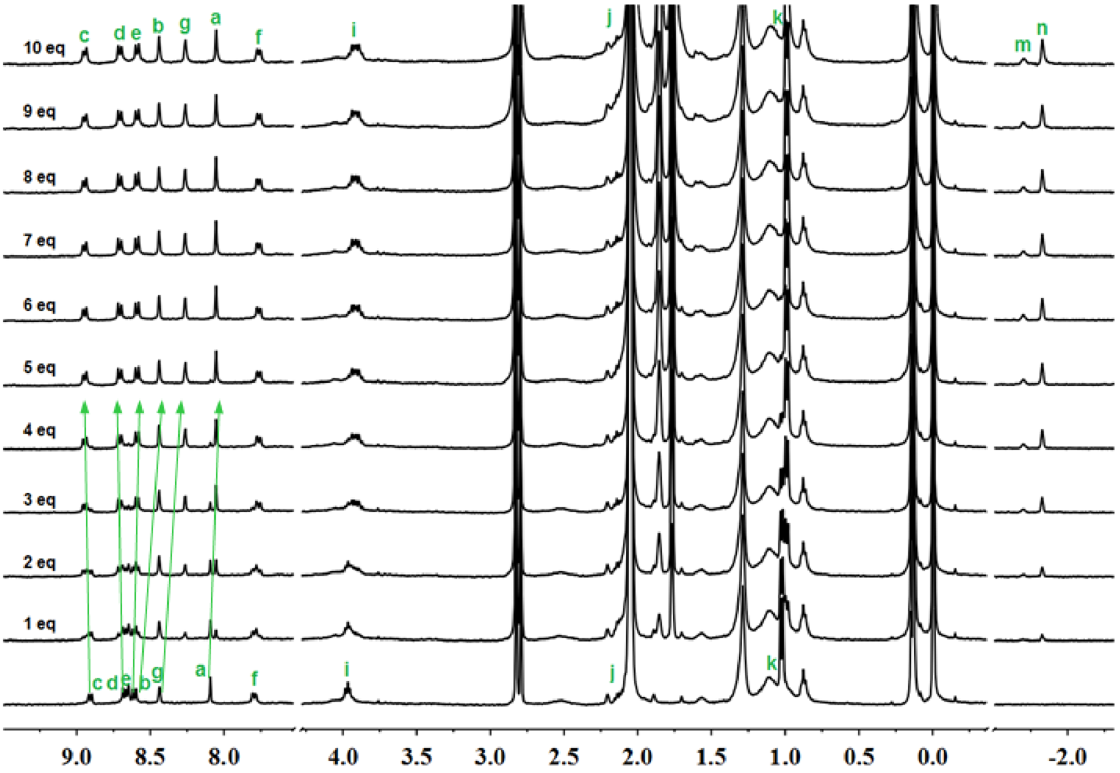

Supplementary Figure 8. Changes of ^{1}H NMR spectra (400 MHz, 298 K) of [Ag_5**1**$_2$]·[OTf]$_5$ (**Ag_5L_2**) (0.5 mM) in acetone-d_6 upon titration with **Ad**.

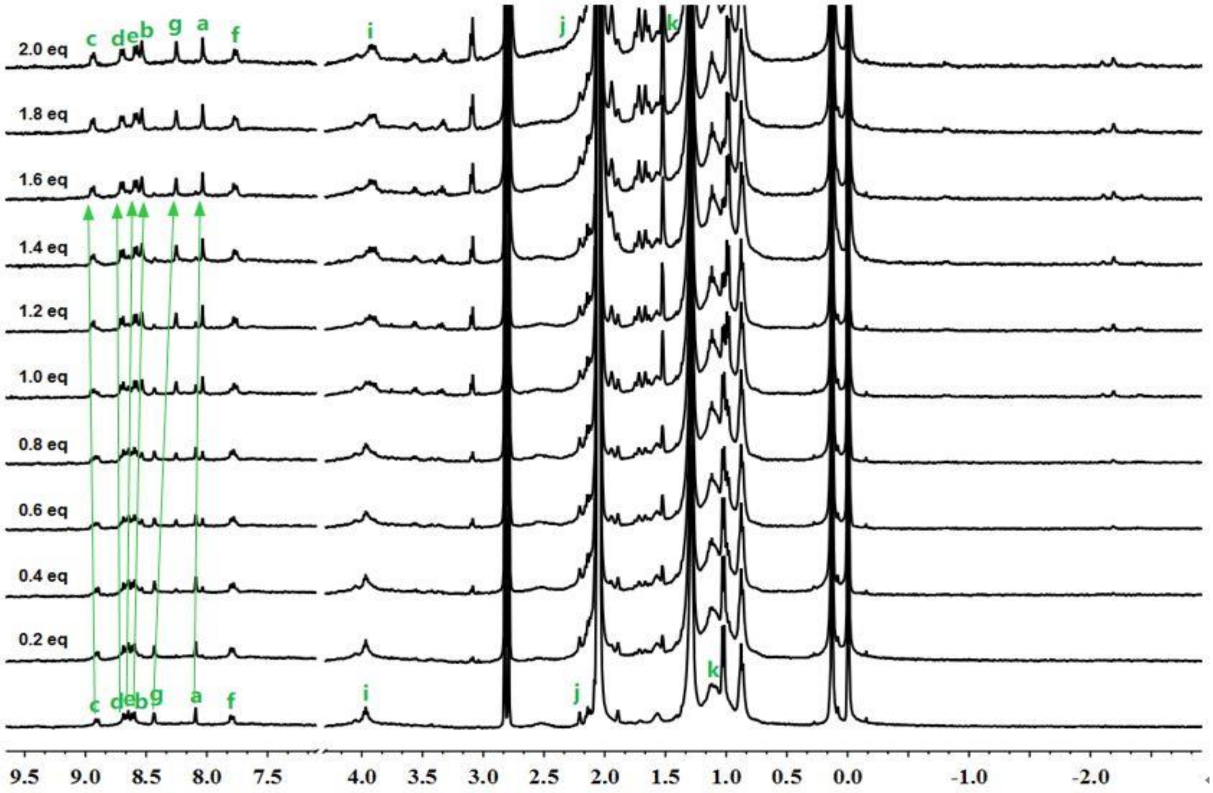

Supplementary Figure 9. Changes of ^{1}H NMR spectra (400 MHz, 298 K) of [Ag_5**1**$_2$]·[OTf]$_5$ (**Ag_5L_2**) (0.5 mM) in acetone-d_6 upon titration with **Ad-MeOH**.

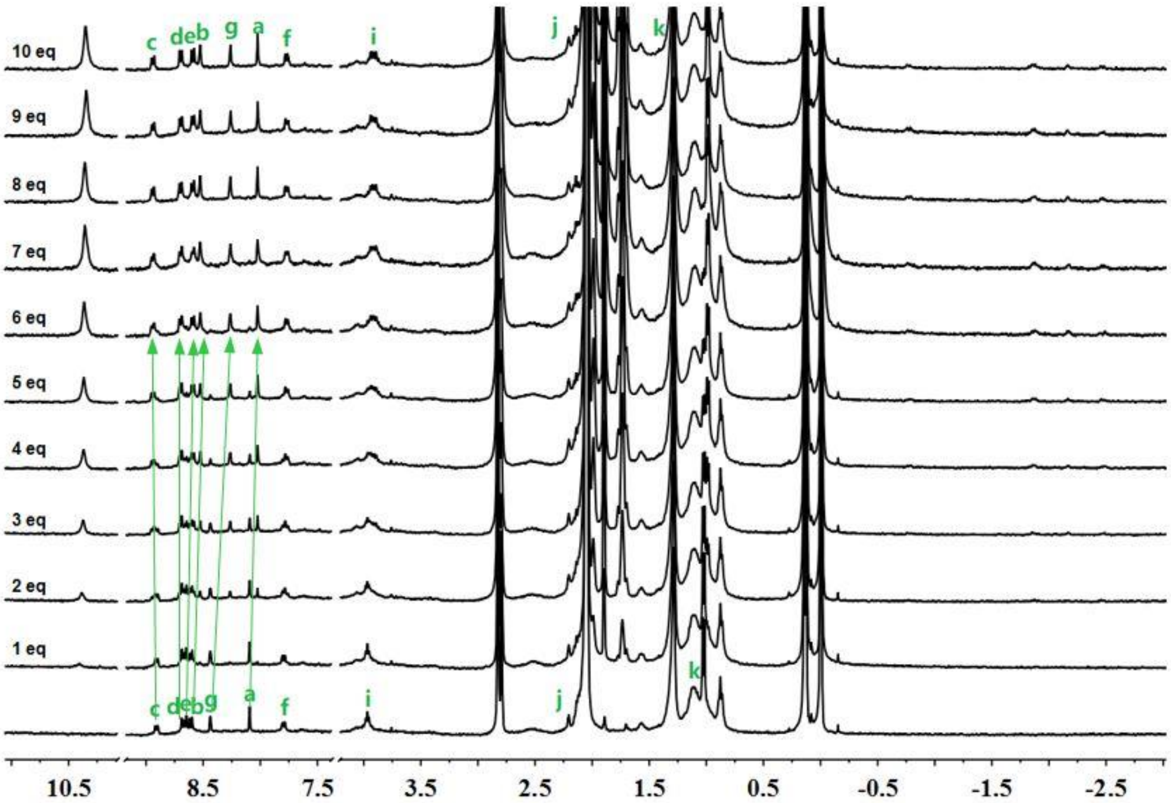

Supplementary Figure 10. Changes of ^{1}H NMR spectra (400 MHz, 298 K) of $[Ag_5\mathbf{1}_2]\cdot[OTf]_5$ ($\mathbf{Ag_5L_2}$) (0.5 mM) in acetone-d_6 upon titration with **Ad-COOH**.

Supplementary Method 6. Encapsulation of **Ad** and Its Derivatives in CD_3CN

^{1}H NMR Titration Experiments. Sample of $[Ag_5\mathbf{1}_2]\cdot[OTf]_5$ was prepared by mixing ligand **1** with 2.5 equivalents of Ag(OTf), using 93:7 (v/v) $CDCl_3/CD_3CN$ as the solvent, followed by the evaporation of the solvent under reduced pressure and the redissolution of the residue into CD_3CN. To the cage solution, a concentrated solution of guest in CD_3CN was gradually added. In this process, a serial of ^{1}H NMR spectra of the sample was recorded by a 600 MHz (^{1}H) spectrometer at 298 K. Guests **Ad**, **Ad-MeOH** and **Ad-COOH** were investigated in the experiments.

Association constants (K_a). K_a of the cage for the studied guests in CD_3CN were basically determined using Supplementary Eq. (S1) shown above.

Different from that in 95:5 (v/v) $CDCl_3/CD_3CN$, the free cages showed a ^{1}H NMR spectrum in CD_3CN with a very low resolution. Therefore, the concentration of the capsule with guest encapsulated ($c_{capsule}$) are determined by,

$$c_{capsule} = aC_{cage,init} \cdot \chi \cdot \frac{20}{m_G} \qquad \text{(S4)}$$

where $c_{capsule}$ is the concentration of the capsule with guest encapsulated, $c_{cage,init}$ the initial concentration of $[Ag_5\mathbf{1}_2]\cdot[OTf]_5$ (1 mM), χ the ratio of integral area of the signals located in range of (−1.0 ~ −3.0) ppm (corresponding to all protons of the guest) against to that of (3.5~4.0) ppm (corresponding to methylene protons of the side chain of both the free cage and the capsule with guest encapsulated), m_G the number of all the protons of the guest (i.e., m_G = 16 for Ad), a the coefficient associating to the dilution in concentration arising from the addition of guest solution, $a = V_T/(V_T + V_{G,\ accum})$. Concentrations for the free cage and the free guest are calculated based on $c_{capsule}$, $c_{cage,init}$, and the accumulated amount of the guest added.

Summary of the corresponding calculations are shown in Supplementary Table 3.

Supplementary Table 3. Summary of the Calculations on the Association Constant of $[Ag_5\mathbf{1}_2]\cdot[OTf]_5$ with **Ad**, **Ad-MeOH** and **Ad-COOH** in CD_3CN.[a]

Guest	Equiv	χ [b]	$c_{capsule}$ (mM)	$c_{cage,free}$ (mM)	$c_{G,free}$ (mM)	K_a ($\times10^4$ M^{-1})	Mean (K_a) ($\times10^4$ M^{-1})	σ [c] ($\times10^4$ M^{-1})
**Ad**	0.6	1.33	0.57	0.43	0.13	1.0	1.2	0.11
	0.8	1.78	0.64	0.36	0.16	1.1		
	1.0	2.85	0.74	0.26	0.26	1.1		
	1.2	4.56	0.82	0.18	0.38	1.2		
	1.4	6.69	0.87	0.13	0.53	1.3		
	1.6	9.00	0.90	0.10	0.70	1.3		
	1.8	11.50	0.92	0.08	0.88	1.3		
	2.0	13.29	0.93	0.07	1.07	1.2		
	2.4	19.00	0.95	0.05	1.45	1.3		
**Ad-MeOH**	0.6	1.13	0.53	0.47	0.07	1.6	1.4	0.14
	0.8	2.03	0.67	0.33	0.13	1.6		
	1.0	3.17	0.76	0.24	0.24	1.3		
	1.2	4.88	0.83	0.17	0.37	1.3		
	1.4	6.69	0.87	0.13	0.53	1.3		
	1.6	9.00	0.90	0.10	0.70	1.3		
	1.8	13.29	0.93	0.07	0.87	1.3		
	2.0	15.67	0.94	0.06	1.06	1.5		
	2.4	19.00	0.95	0.05	1.45	1.3		
**Ad-COOH**	0.6	1.04	0.51	0.49	0.11	9.5	8.9	0.53
	0.8	1.63	0.62	0.38	0.18	9.1		
	1.0	2.57	0.72	0.28	0.28	9.2		
	1.2	3.55	0.78	0.22	0.42	8.4		
	1.4	4.88	0.83	0.17	0.57	8.6		
	1.6	6.69	0.87	0.13	0.73	9.2		

1.8	8.09	0.89	0.11	0.91	8.9
2.0	10.11	0.91	0.09	1.09	9.3
2.4	11.50	0.92	0.08	1.48	7.8

[a] Calculations were carried out based on Supplementary Eq. S4 listed in Page S15. The raw data were derived from the ^{1}H NMR titration experiment shown in Supplementary Figures 11−13. [b] Molar ratio (χ) of c_{caps} against $c_{cage,free}$. [c] Standard deviation of the mean K_a.

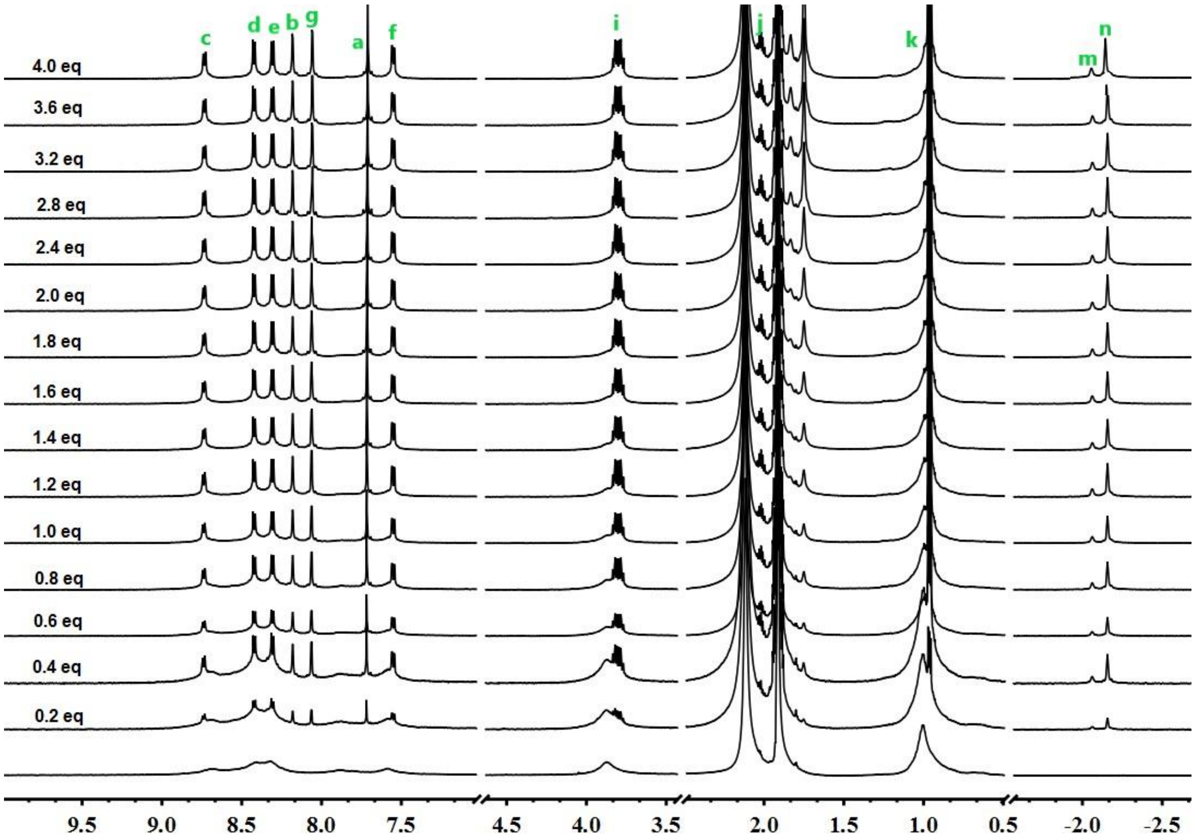

Supplementary Figure 11. Changes of ^{1}H NMR spectra (600 MHz, 298 K) of $[Ag_5\mathbf{1}_2]\cdot[OTf]_5$ ($\mathbf{Ag_5L_2}$) (1 mM) in CD_3CN upon titration with **Ad**.

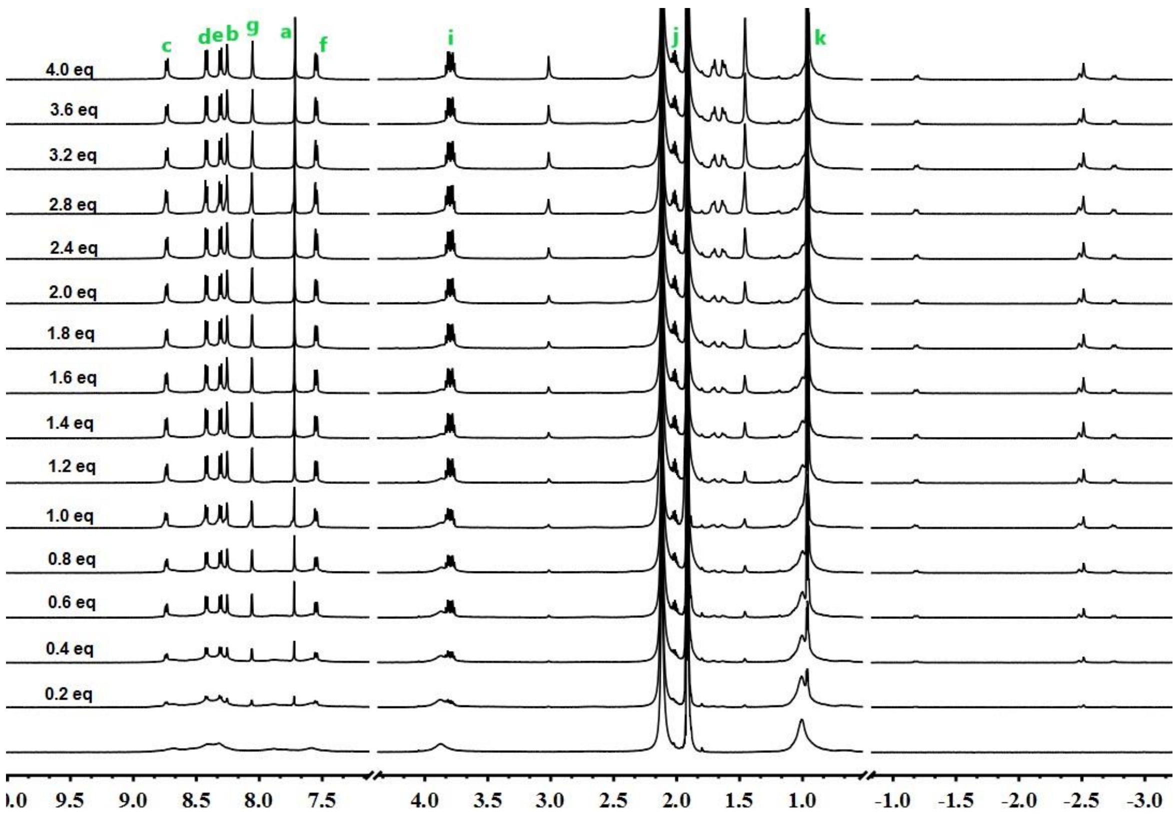

Supplementary Figure 12. Changes of ^{1}H NMR spectra (600 MHz, 298 K) of $[Ag_5\mathbf{1}_2]\cdot[OTf]_5$ (**Ag_5L_2**) (1 mM) in CD_3CN upon titration with **Ad-MeOH**.

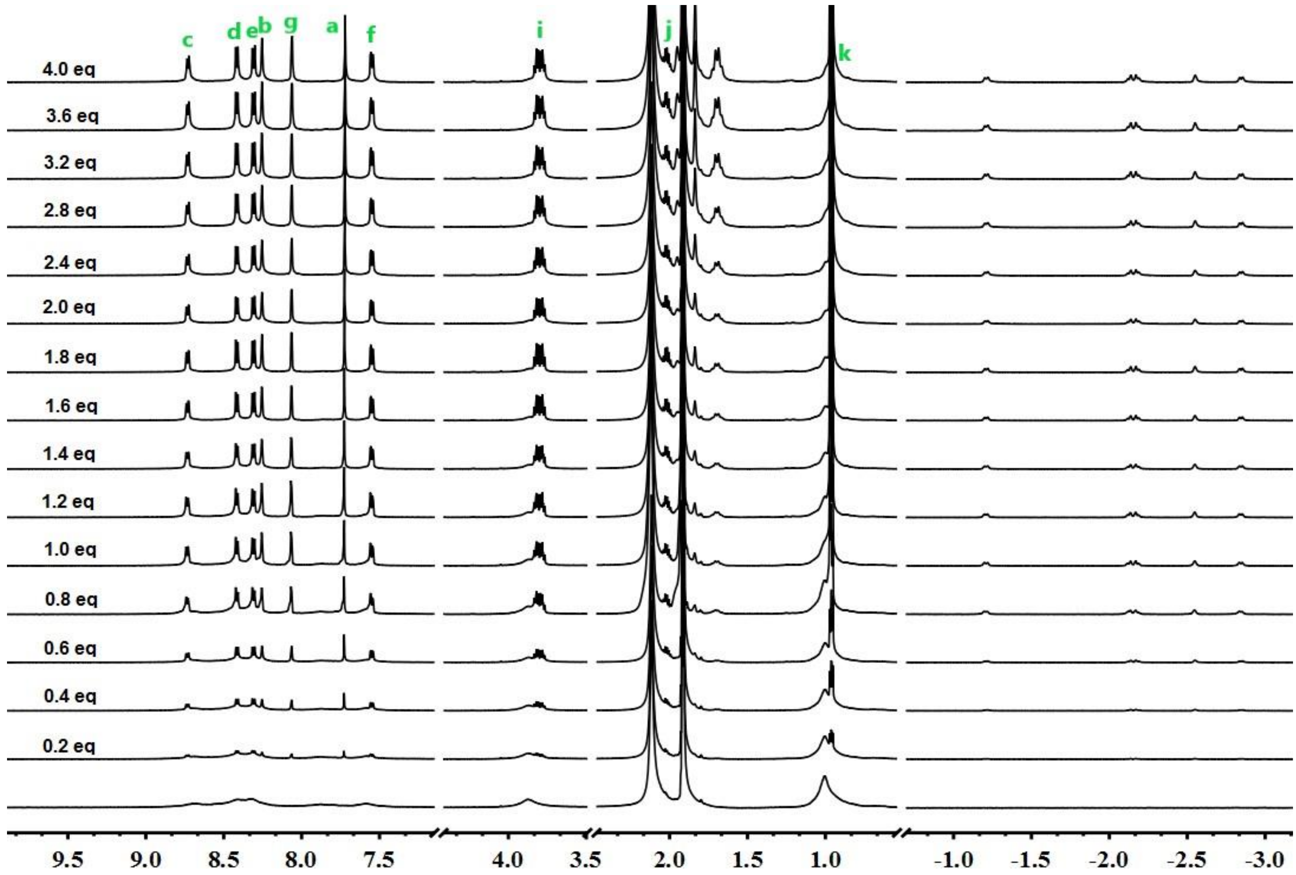

Supplementary Figure 13. Changes of ^{1}H NMR spectra (600 MHz, 298 K) of $[Ag_5\mathbf{1}_2]\cdot[OTf]_5$ (1 mM) in CD_3CN upon titration with **Ad-COOH**.

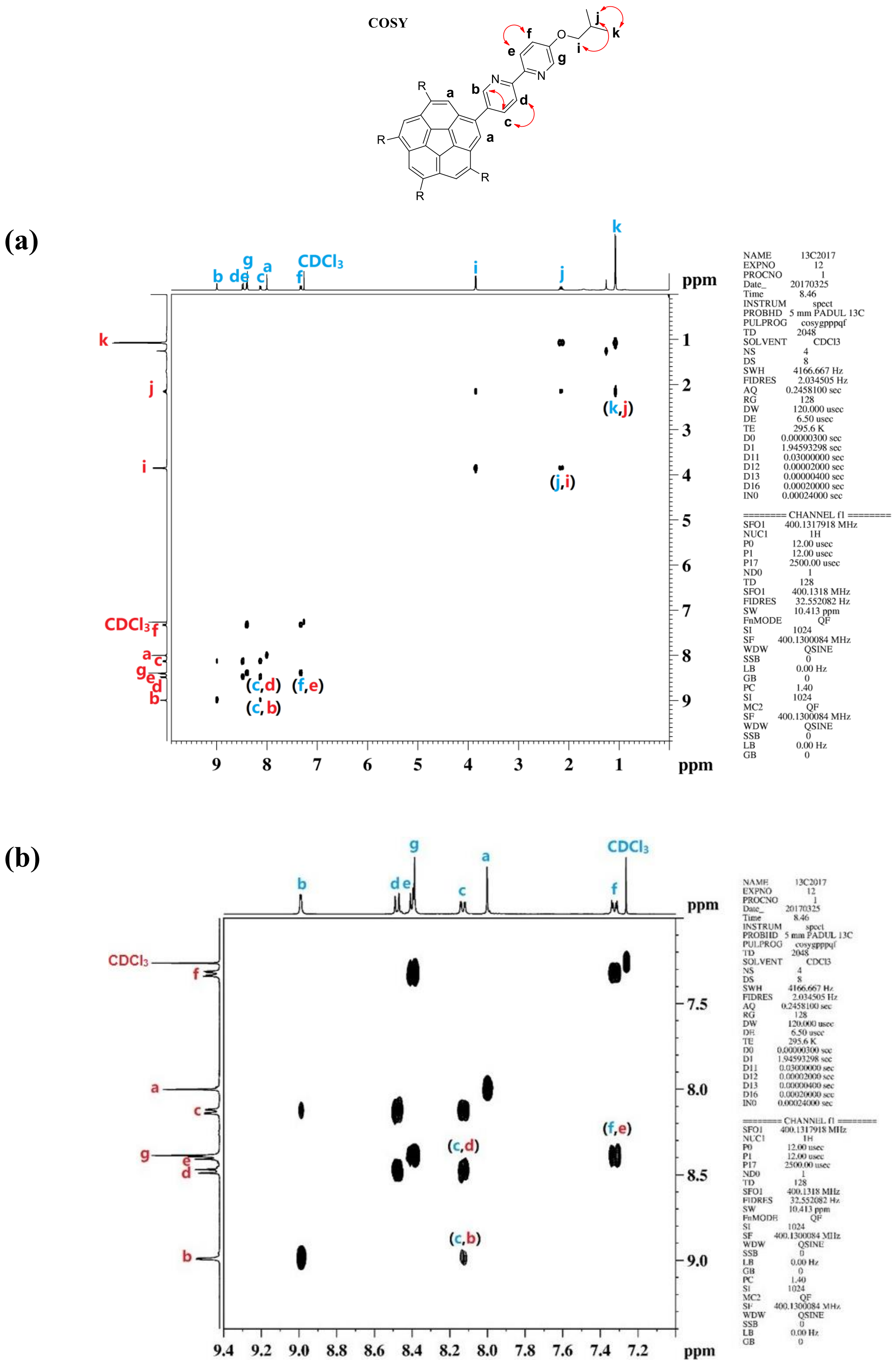

Supplementary Figure 14. **(a)** ^{1}H–^{1}H COSY spectrum (500 MHz, 298 K, 95:5 (v/v) $CDCl_3/CD_3CN$) of the ligand **1** (5 mM). **(b)** Zoom of the spectrum (a).

HSQC

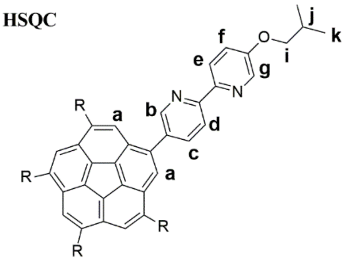

(a)

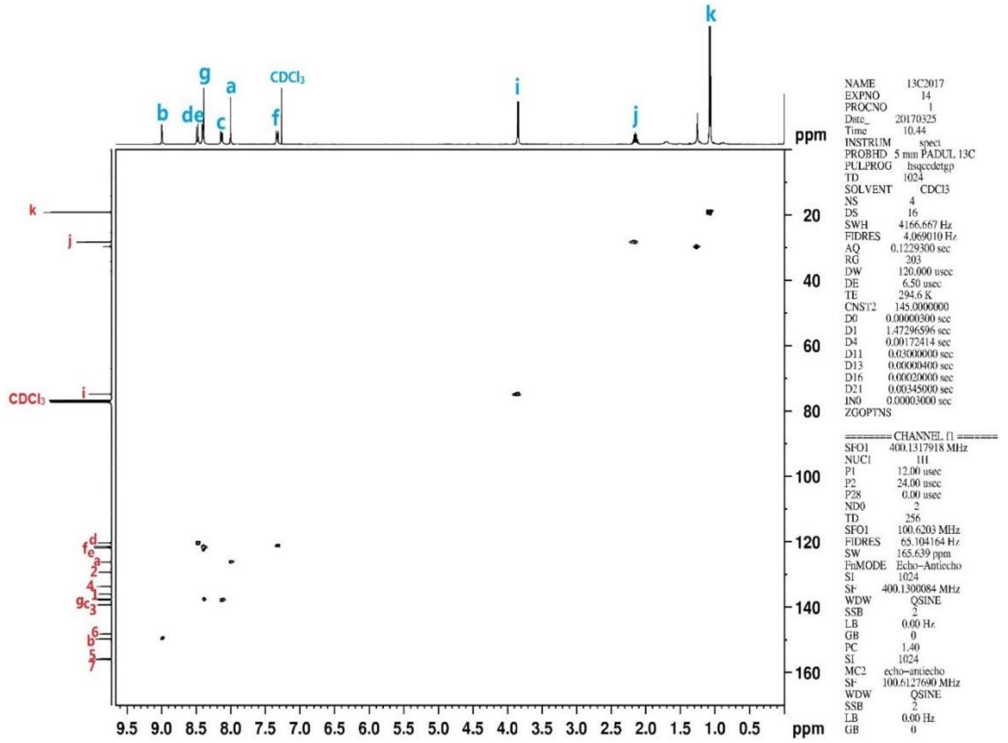

(b)

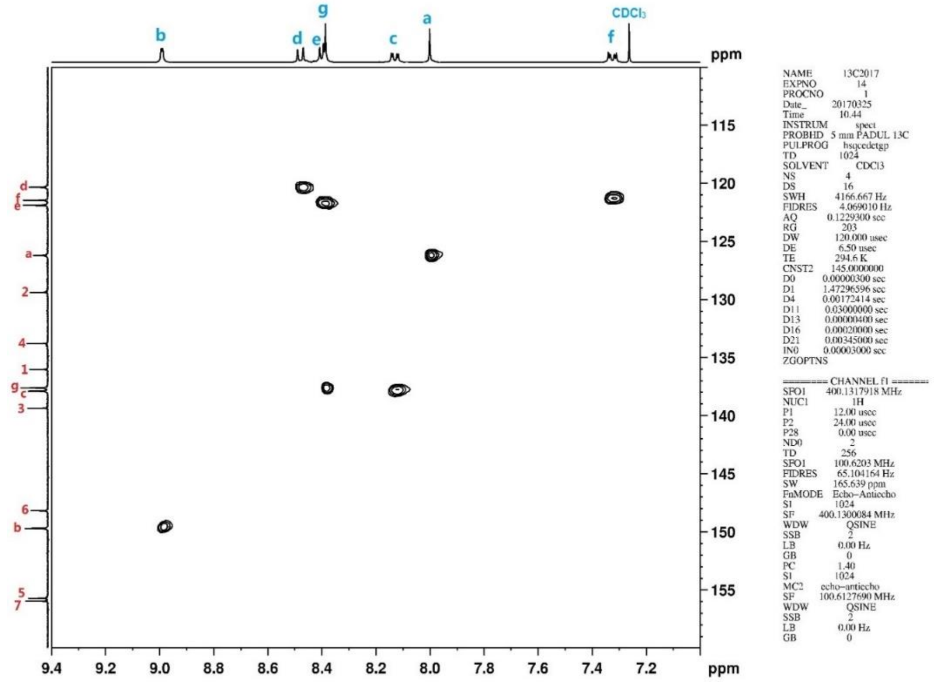

Supplementary Figure 15. (a) ^{1}H–^{13}C HSQC spectrum (500 MHz, 298 K, 95:5 (v/v) $CDCl_3/CD_3CN$) of ligand **1** (5 mM). **(b)** Zoom of spectrum (a).

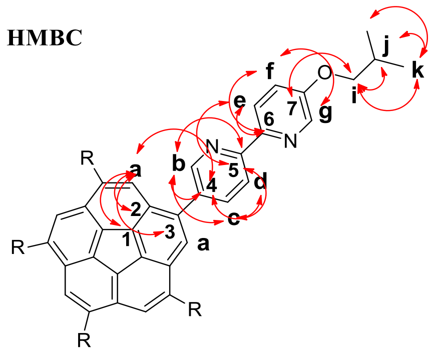

(a)

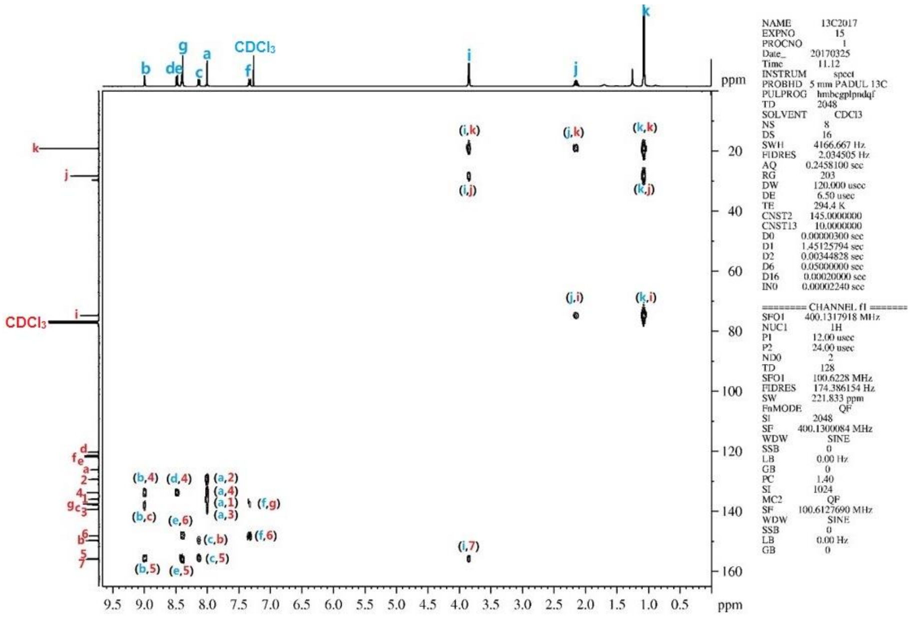

(b)

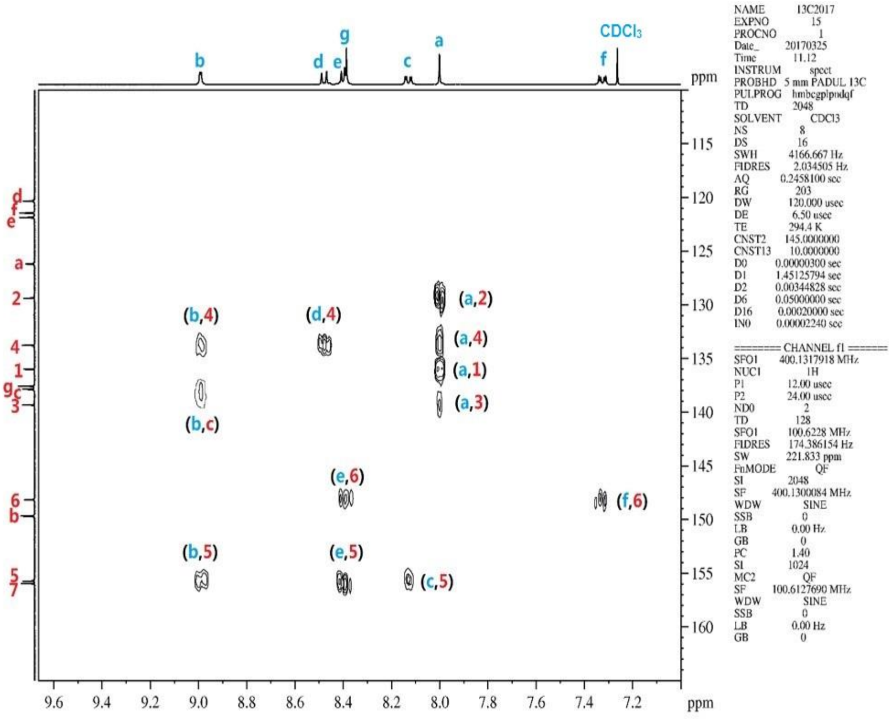

Supplementary Figure 16. **(a)** ^{1}H–^{13}C HMBC spectrum (500 MHz, 298K, 95:5 (v/v) $CDCl_3/CD_3CN$) of ligand **1** (5 mM). **(b)** Zoom of the spectrum (a).

(a)

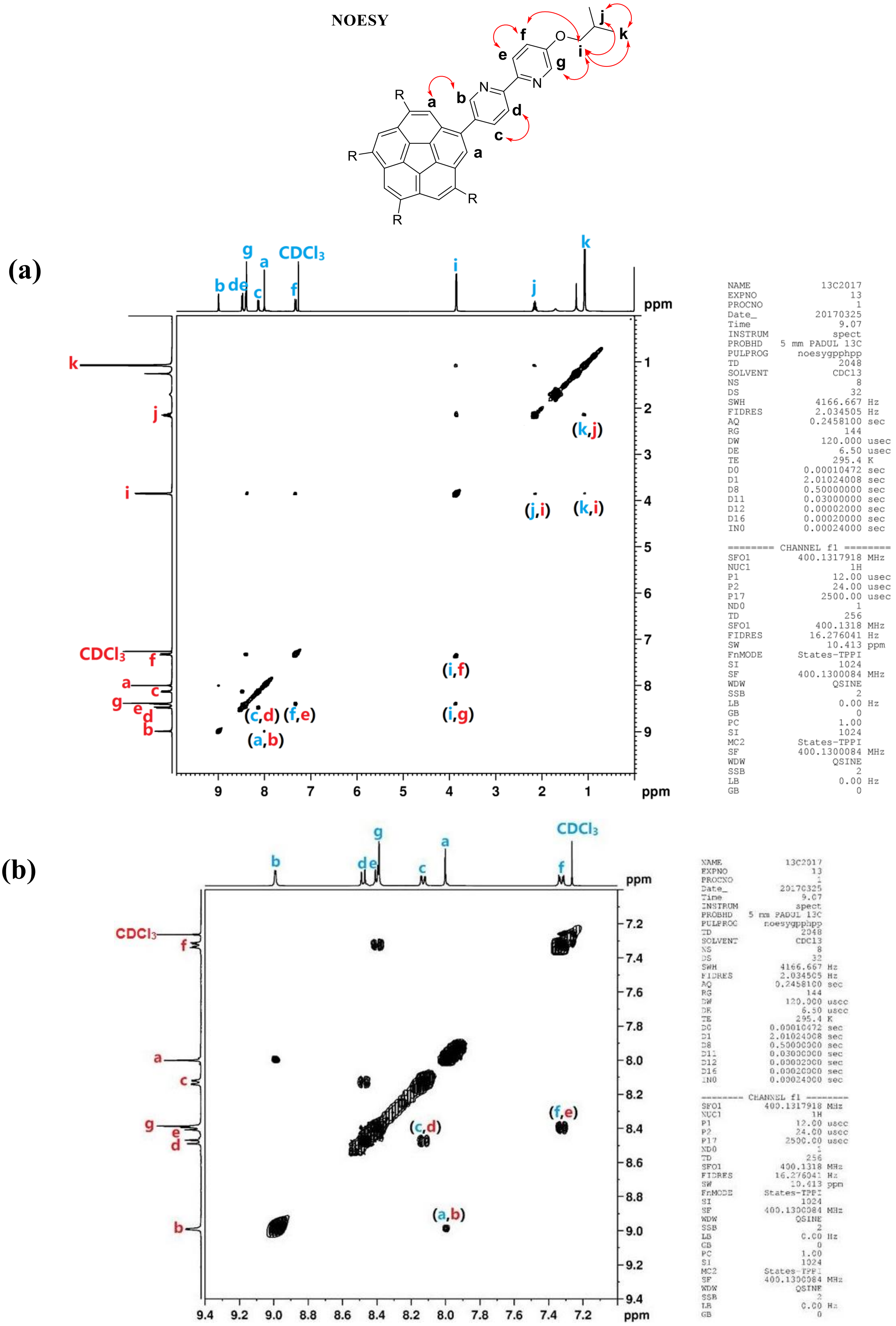

Supplementary Figure 17. (a) [1]H–[1]H NOESY spectrum (500 MHz, 298 K, 95:5 (v/v) $CDCl_3/CD_3CN$) of ligand **1** (5 mM). **(b)** Zoom of spectrum a.

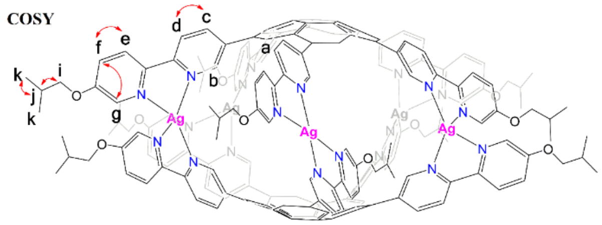

(a)

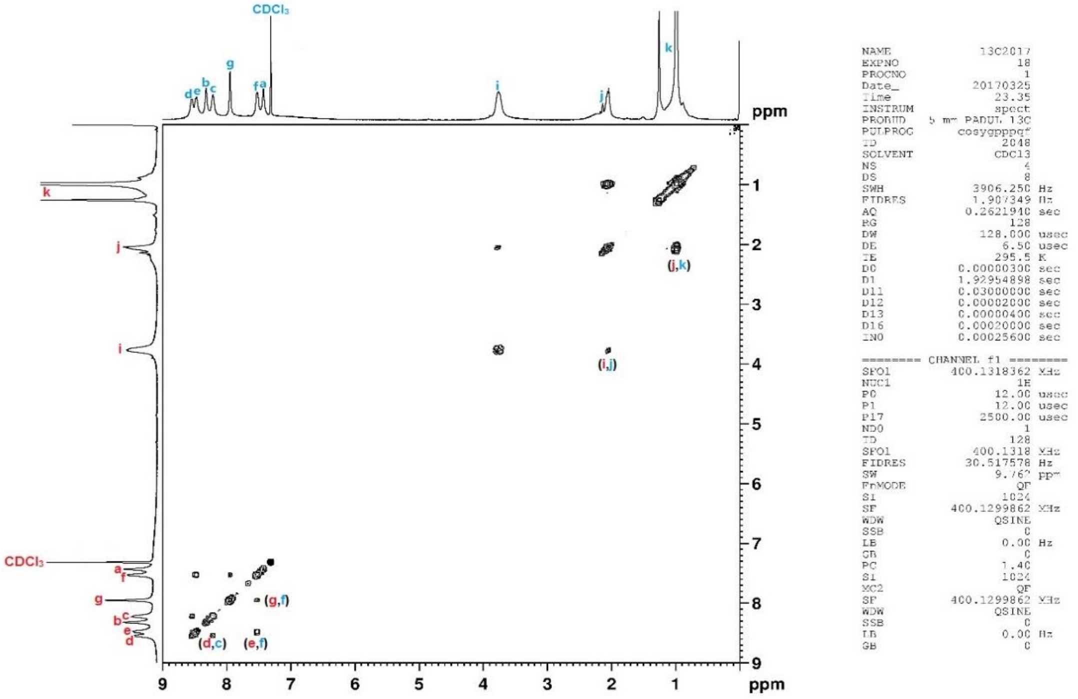

(b)

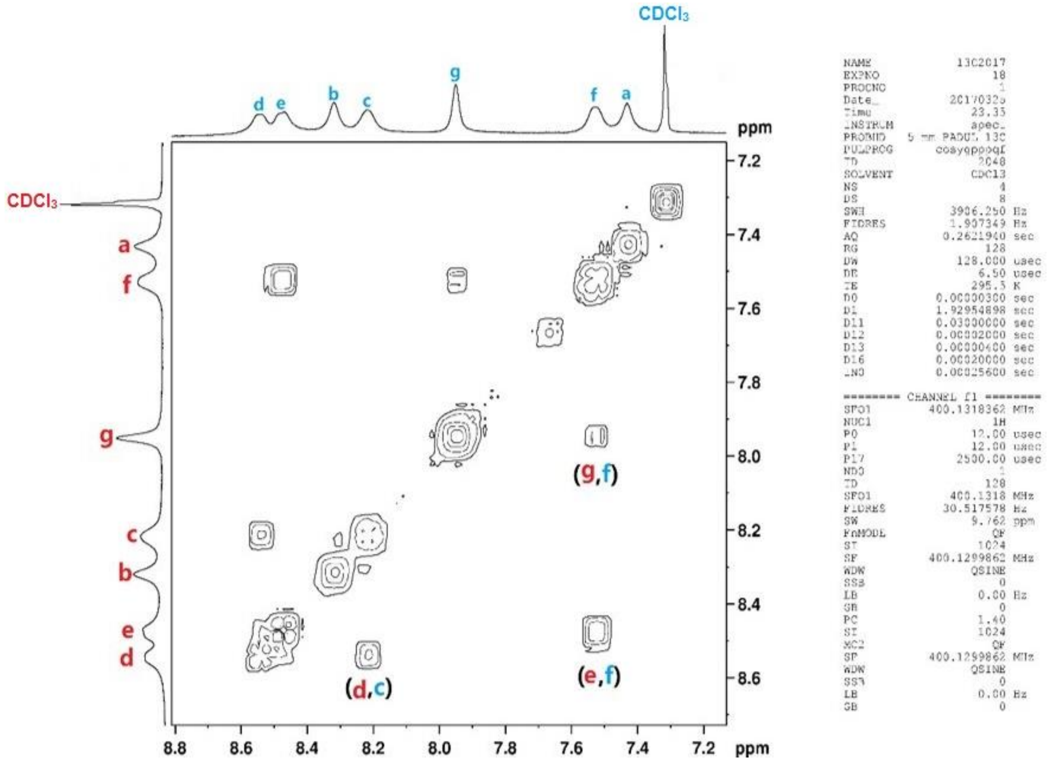

Supplementary Figure 18. **(a)** ^{1}H–^{1}H COSY spectrum (500 MHz, 298K, 95:5 (v/v) $CDCl_3/CD_3CN$) of $[Ag_5\mathbf{1}_2]\cdot[OTf]_5$ ($\mathbf{Ag_5L_2}$) (5 mM). **(b)** Zoom of the spectrum (a).

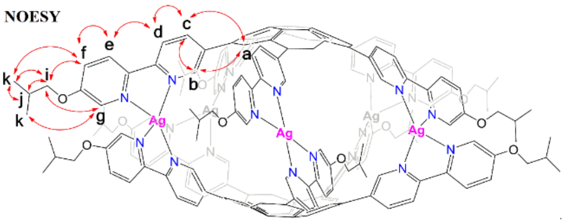

(a)

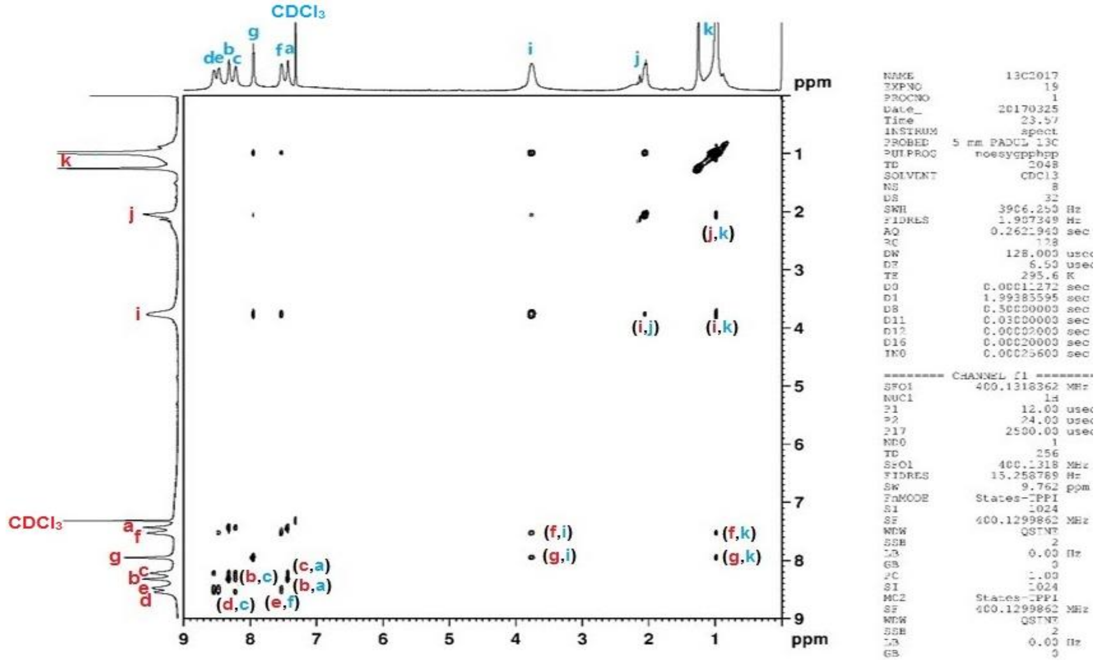

(b)

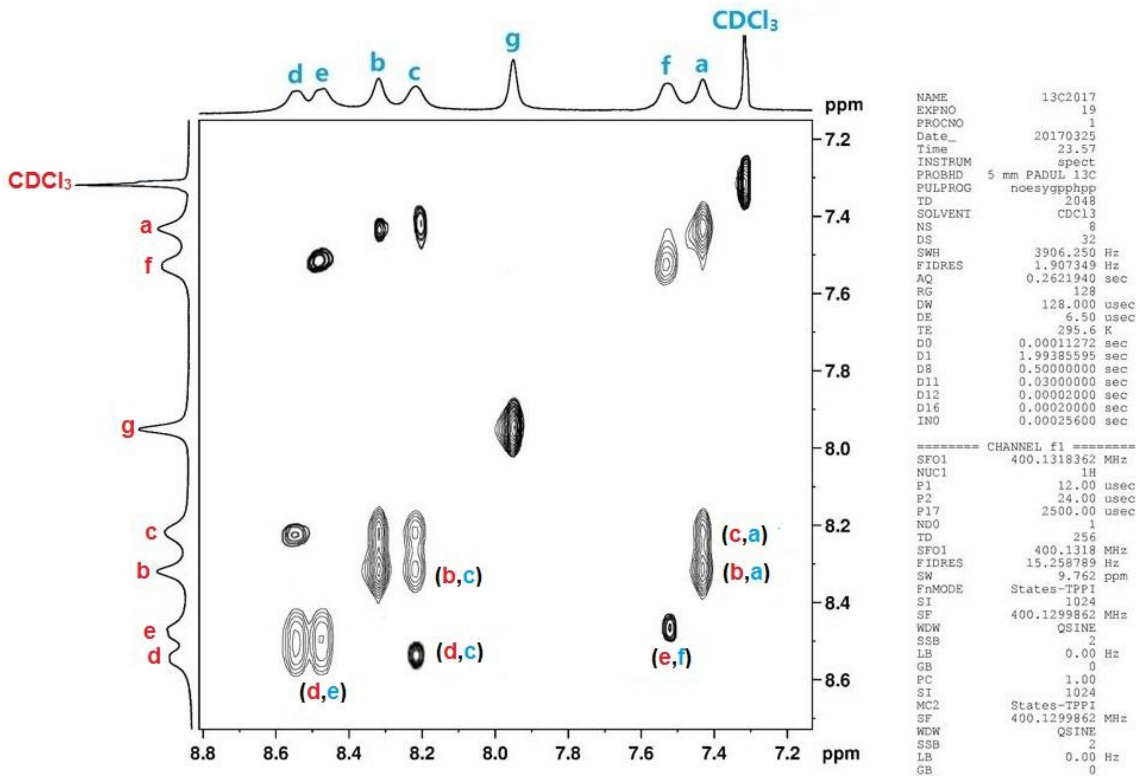

Supplementary Figure 19. **(a)** ^1H–^1H NOESY spectrum (500 MHz, 298K, 95:5 (v/v) $CDCl_3/CD_3CN$) of $[Ag_5\mathbf{1}_2]\cdot[OTf]_5$ (5 mM). **(b)** Zoom of the spectrum (a).

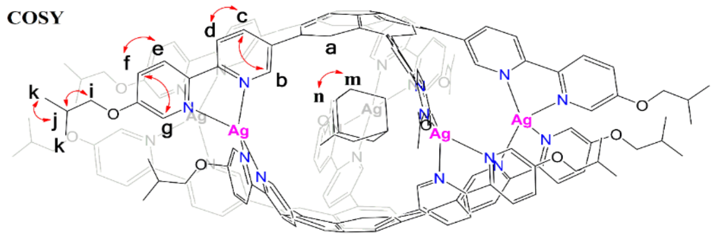

(a)

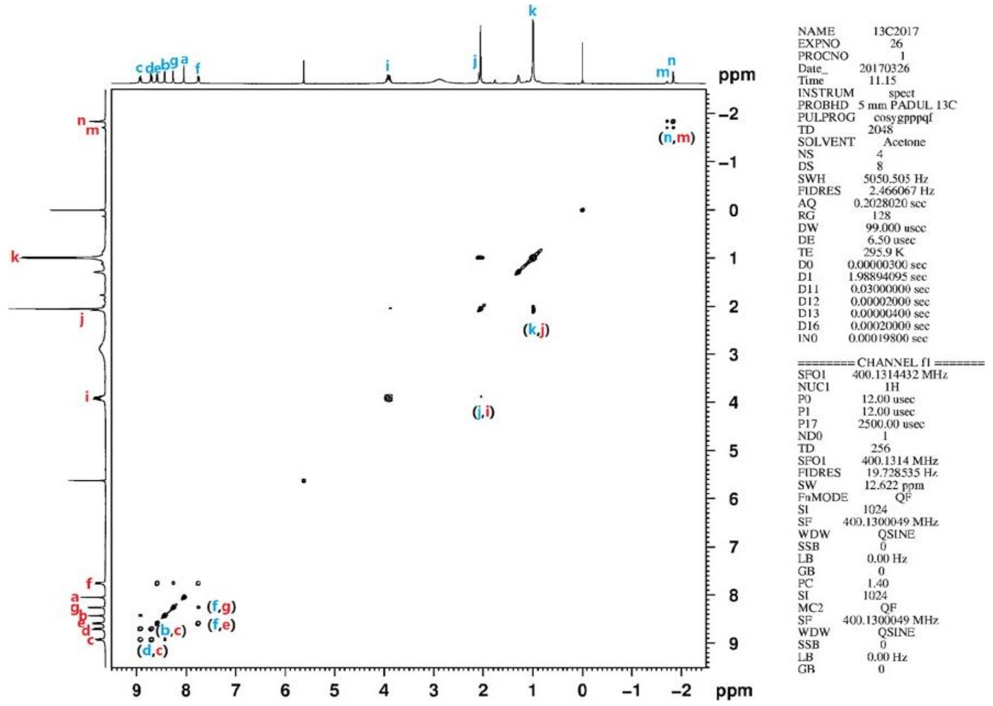

(b)

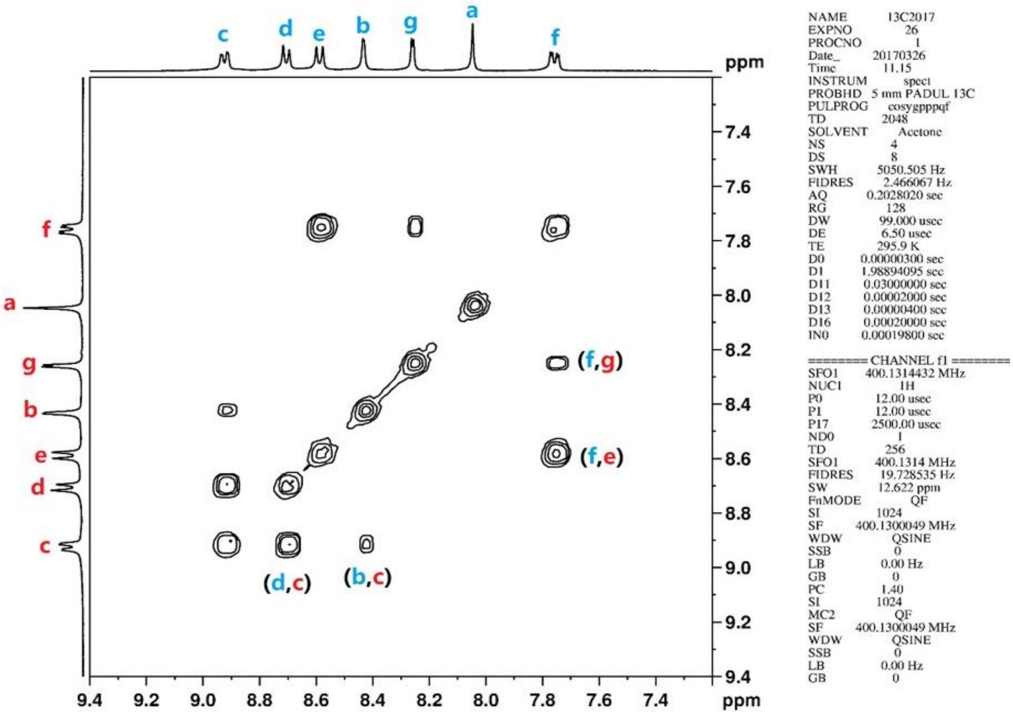

Supplementary Figure 20. **(a)** ^{1}H-^{1}H COSY (500 MHz, 298K, acetone-*d*$_6$) of **Ad**⊂[Ag_5**1**$_2$]·[OTf]$_5$ (5 mM). (b) Zoom of the spectrum (a).

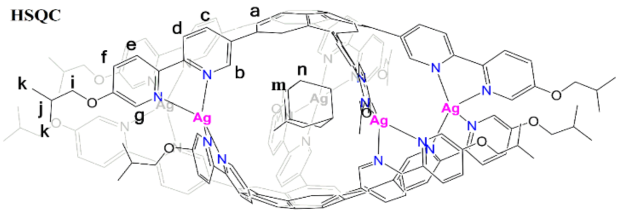

(a)

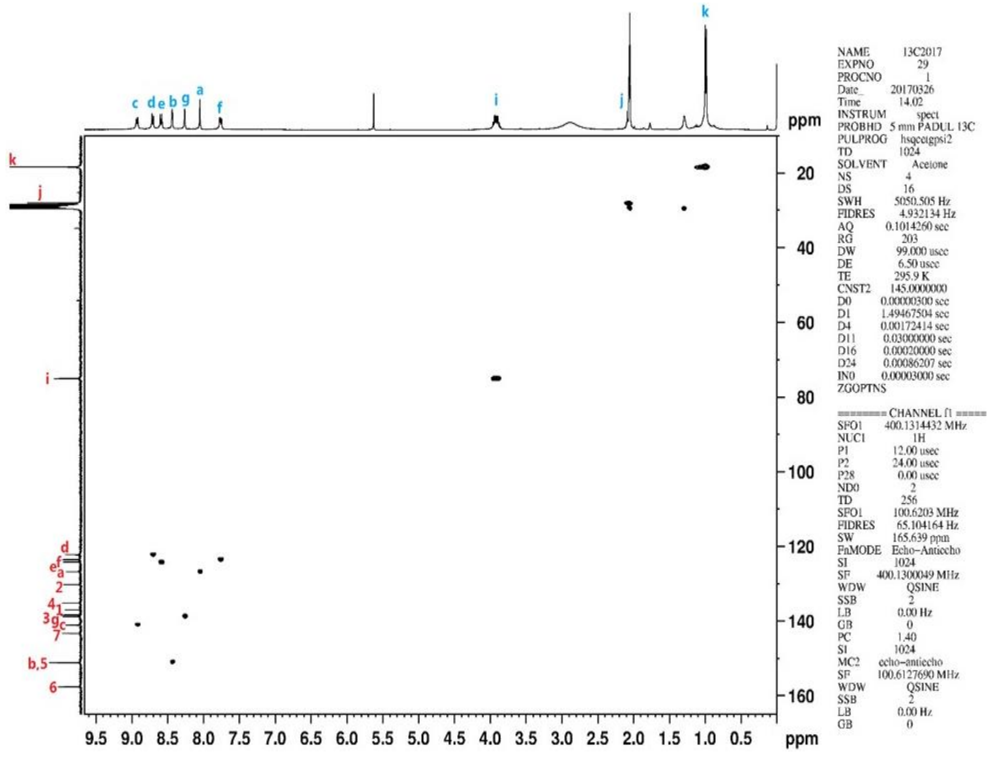

(b)

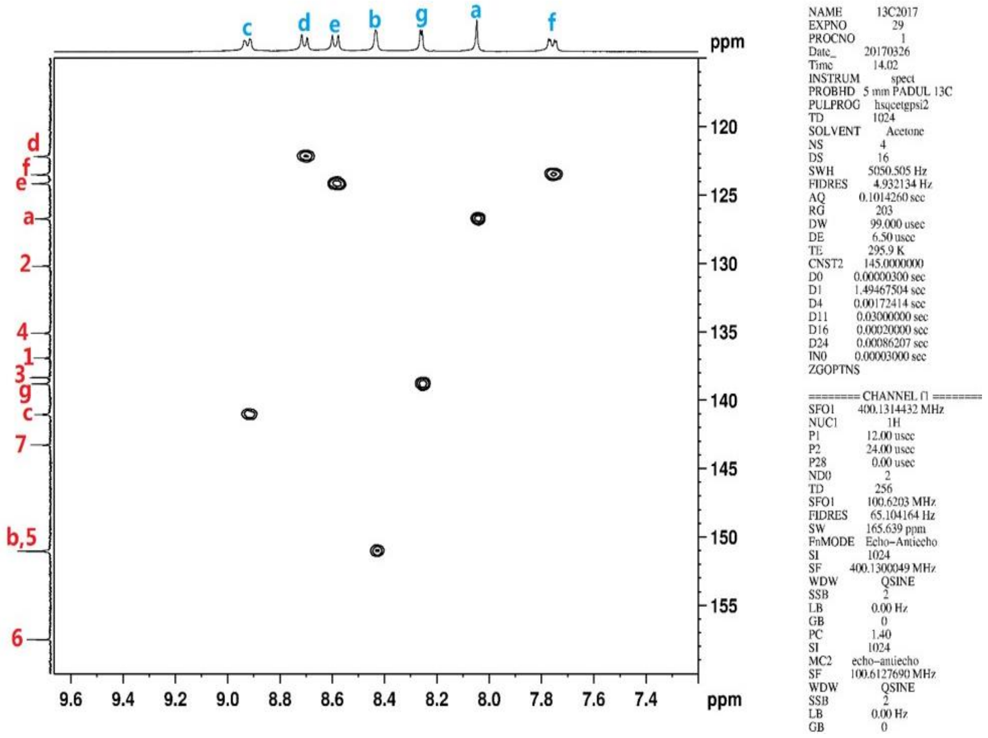

Supplementary Figure 21. **(a)** ^{1}H-^{13}C HSQC (500 MHz, 298K, acetone-d_6) of **Ad**⊂[Ag_5**1**$_2$]·[OTf]$_5$ (5 mM). **(b)** Zoom of the spectrum (a).

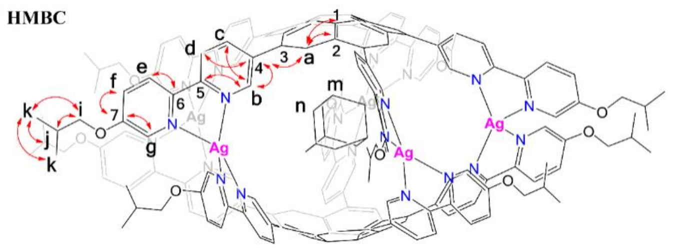

(a)

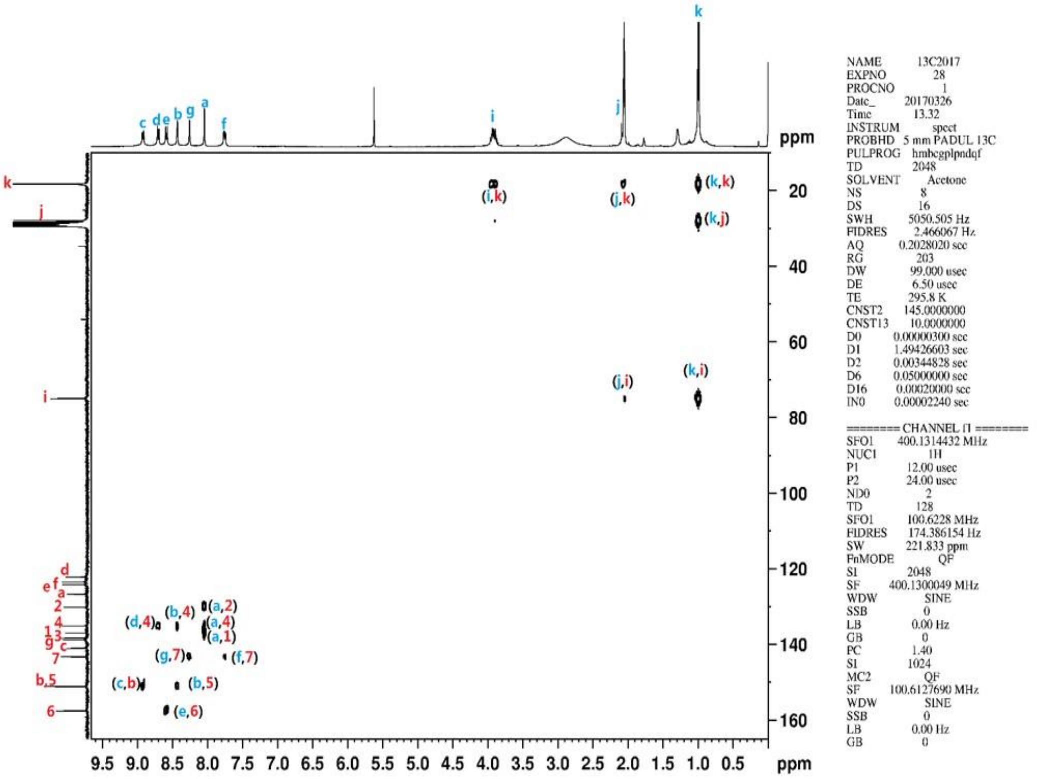

(b)

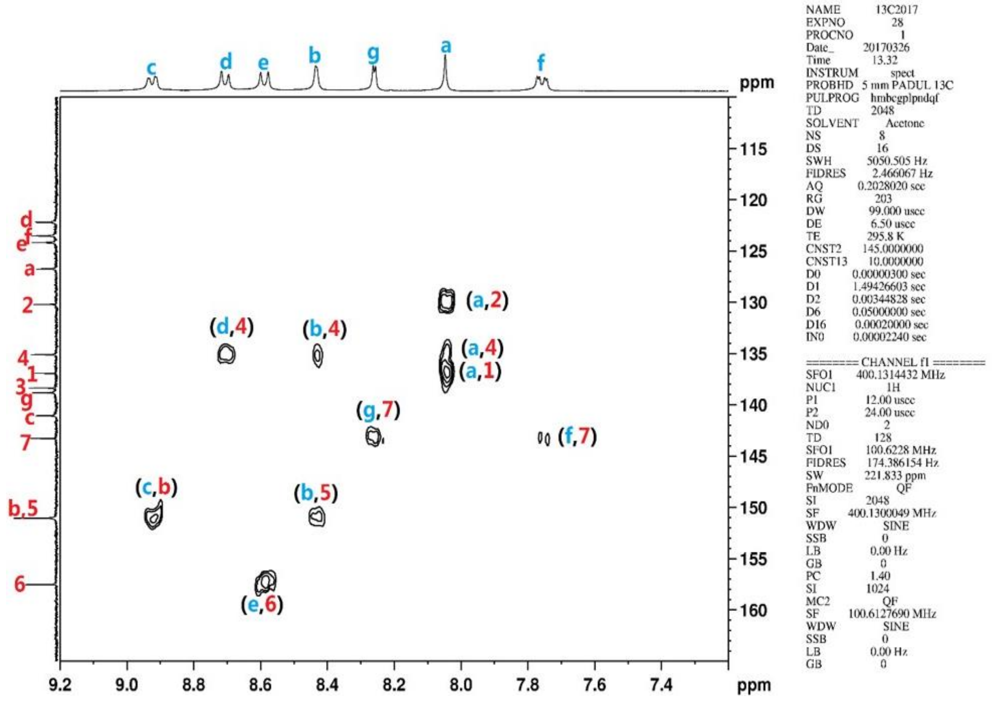

Supplementary Figure 22. **(a)** ^{1}H-^{13}C HMBC (500 MHz, 298K, acetone-d_6) of **Ad**⊂[Ag_5**1**$_2$]·[OTf]$_5$ (5 mM). **(b)** Zoom of the spectrum (a).

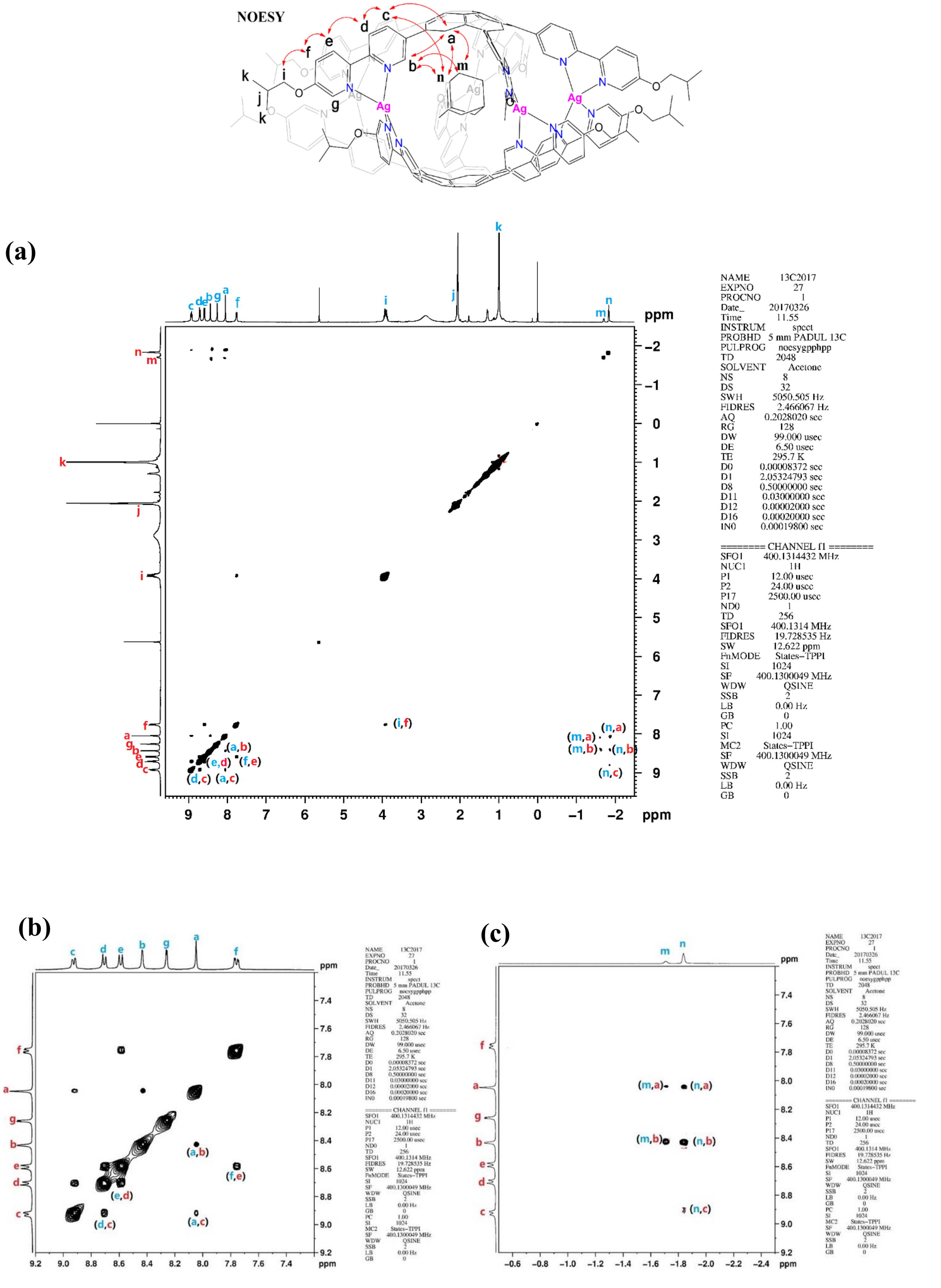

Supplementary Figure 23. **(a)** ^{1}H-^{1}H NOESY (500 MHz, 298K, acetone-d_6) of **Ad**⊂[Ag_5**1**$_2$]·[OTf]$_5$ (5 mM). **(b)** Zoom of the spectrum (a). **(c)** Zoom of the spectrum (a).

Supplementary Method 7. DOSY Experiments on Ligand **1**, $\mathbf{Ag_5L_2}$ and $\mathbf{Ad{\subset}Ag_5L_2}$

Sample of $[Ag_5\mathbf{1}_2]\cdot[OTf]_5$ was prepared by mixing ligand **1** with 2.5 equivalents of Ag(OTf), using 95:5 (v/v) $CDCl_3/CD_3CN$ as the solvent. Sample of $\mathbf{Ad}{\subset}[Ag_5\mathbf{1}_2]\cdot[OTf]_5$ was prepared by mixing $[Ag_5\mathbf{1}_2]\cdot[OTf]_5$ with 4 equivalent of **Ad**, using CD_3CN as the final solvent.

Diffusion NMR experiments were carried out on a Bruker Advance III (^1H, 500 MHz) instrument using triple resonance broad-band probe with ATM (5 mm PATBO BB-1H/19F/D Z-GRD) and a Diff30 pulsed field gradient amplifier. The 2D diffusion-ordered NMR (DOSY) spectra were recorded using the "stebpgp1s" pulse sequence, with the gradient strength varied linearly from 5% up to 95% in 16 steps. T1 was independently measured prior to running each DOSY experiment; and the spectra were typically recorded with a delay of 5 times greater than T1 between scans in order to guarantee sufficient relaxation of the magnetization. The obtained data were processed using the T1/T2 Relaxation procedure implemented in Topspin 3.5 software package, where each 2D column was nonlinear-fitted separately. Attenuations of three aromatic protons signals for the ligands and the cages (see Supplementary Table 4) were employed for the fitting. Diffusion constants were reported as mean of the values obtained from those three runs.

The obtained diffusion coefficients (***D***) are summarized in Supplementary Table 4. Examples for simulated diffusion decay curves are shown in Supplementary Figures 25、27 and 29.

Supplementary Table 4. Summary of Diffusion Coefficients (D, $m^2{\cdot}s^{-1}$) of Ligand **1** and $[Ag_5\mathbf{1}_2]\cdot[OTf]_5$ ($\mathbf{Ag_5L_2}$) in 95:5 (v/v) $CDCl_3/CD_3CN$ and $\mathbf{Ad}{\subset}[Ag_5\mathbf{1}_2]\cdot[OTf]_5$ ($\mathbf{Ad{\subset}Ag_5L_2}$) in CD_3CN at 298 K.[a]

Species	Data Sets and D	Run 1	Run 2	Run 3	Mean (D)	σ^b Mean(D)
Ligand **1**	Peak	H_c, 9.08-8.96 ppm	H_a, 8.47-8.37 ppm	H_b, 8.07-7.97 ppm		
	D ($\times10^{-10}$)	3.708	3.779	3.806	3.764	0.051
$[Ag_5\mathbf{1}_2]\cdot[OTf]_5$	Peak	H_e, 8.48 ppm	H_b, 8.23ppm	H_c, 7.98 ppm		
	D ($\times10^{-10}$)	1.438	1.421	1.417	1.425	0.011
$\mathbf{Ad}{\subset}[Ag_5\mathbf{1}_2]\cdot[OTf]_5$	Peak	H_C, 8.574 ppm	H_d, 8.465 ppm	H_g, 7.870 ppm		
	D ($\times10^{-10}$)	1.658	1.677	1.676	1.670	0.011

[a] The diffusion coefficients were derived from the DOSY experiments by using the T1/T2 Relaxation procedure implemented in Topspin 2.1 software package. The data sets employed for the fitting were denoted by the labels of the protons (the labels of the atoms are shown in Figures 14、18 and 21) or the chemical shift of the peaks. Throughout the table, the unit of diffusion coefficients, including D and the mean D, is $m^2{\cdot}s^{-1}$. [b] Standard derivation of the mean D.

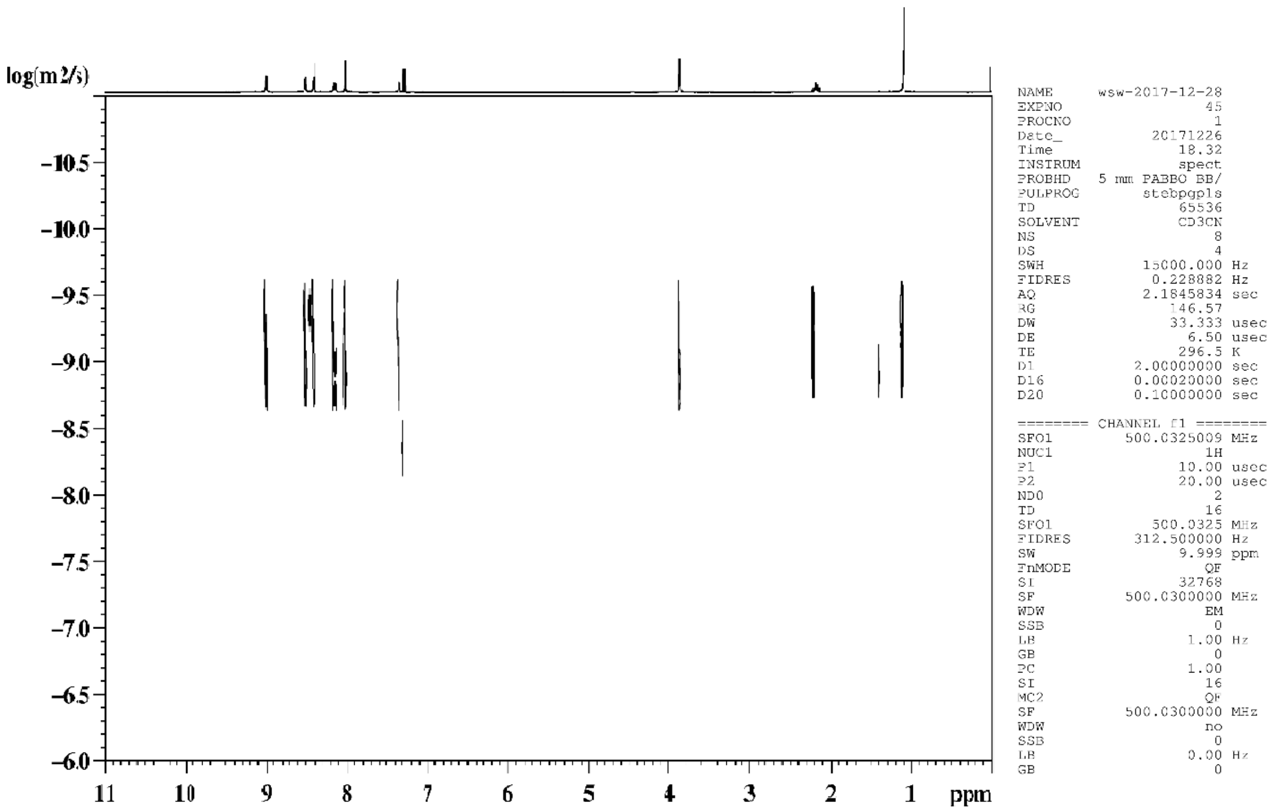

Supplementary Figure 24. Part of the DOSY spectrum (500 MHz, 298 K) of ligand **1** (10 mM) in 95:5(v/v) $CDCl_3/CD_3CN$.

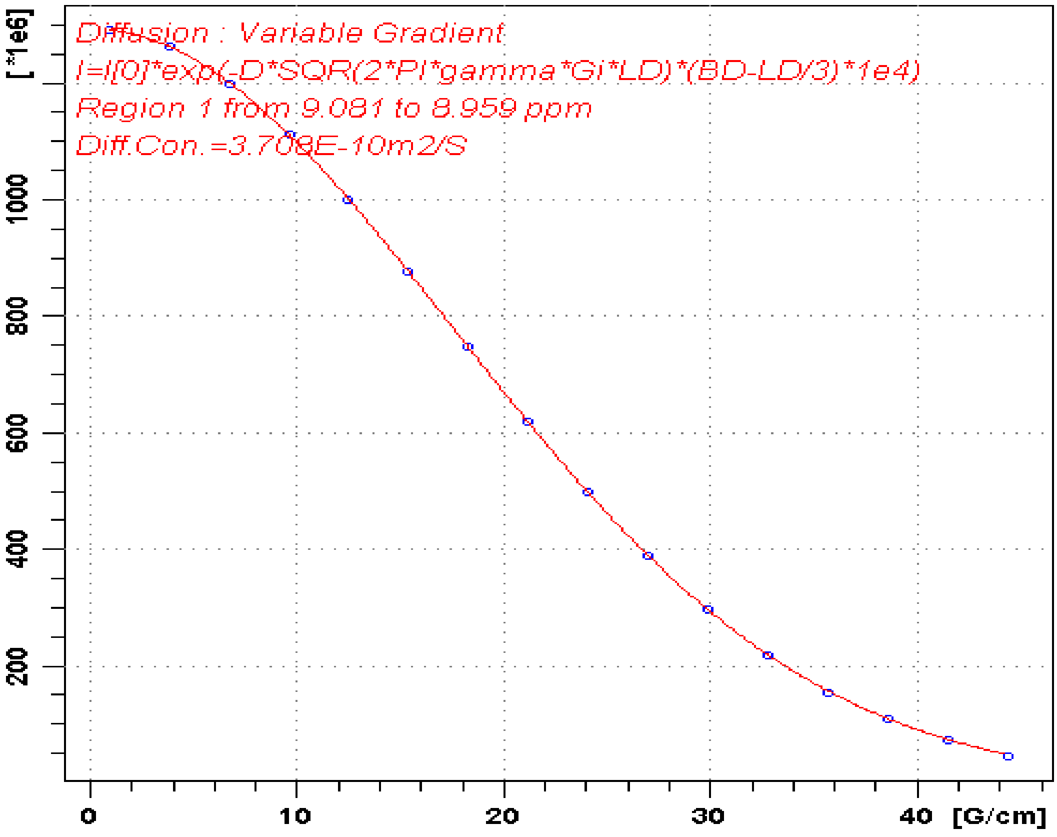

Supplementary Figure 25. Simulated diffusion decay curve for signal H_b (peak from 9.08 ppm to 8.96 ppm) on ligand **1**.

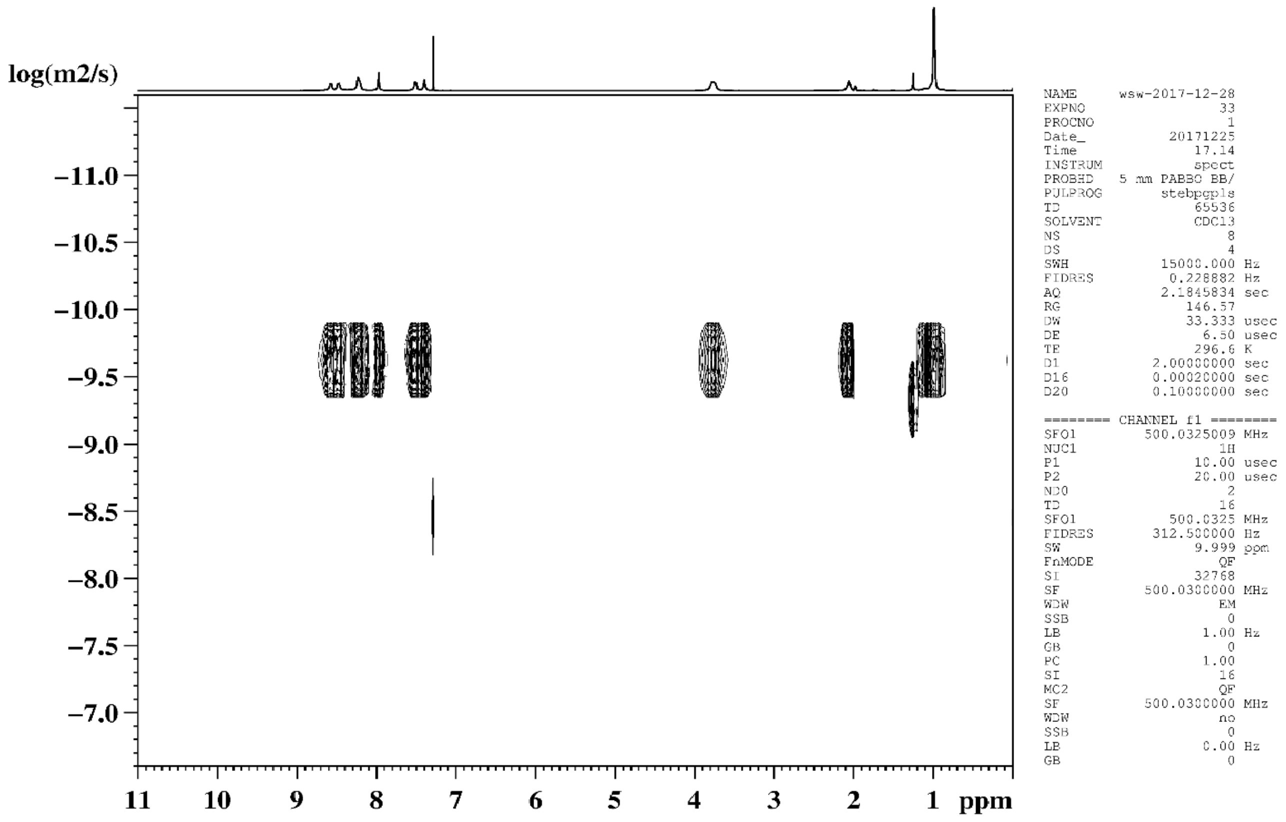

Supplementary Figure 26. Part of the DOSY spectrum (500 MHz, 298 K) of the molecular cage $[Ag_5\mathbf{1}_2]\cdot[OTf]_5$ (**Ag_5L_2**, 5 mM) in 95:5(v/v) $CDCl_3/CD_3CN$.

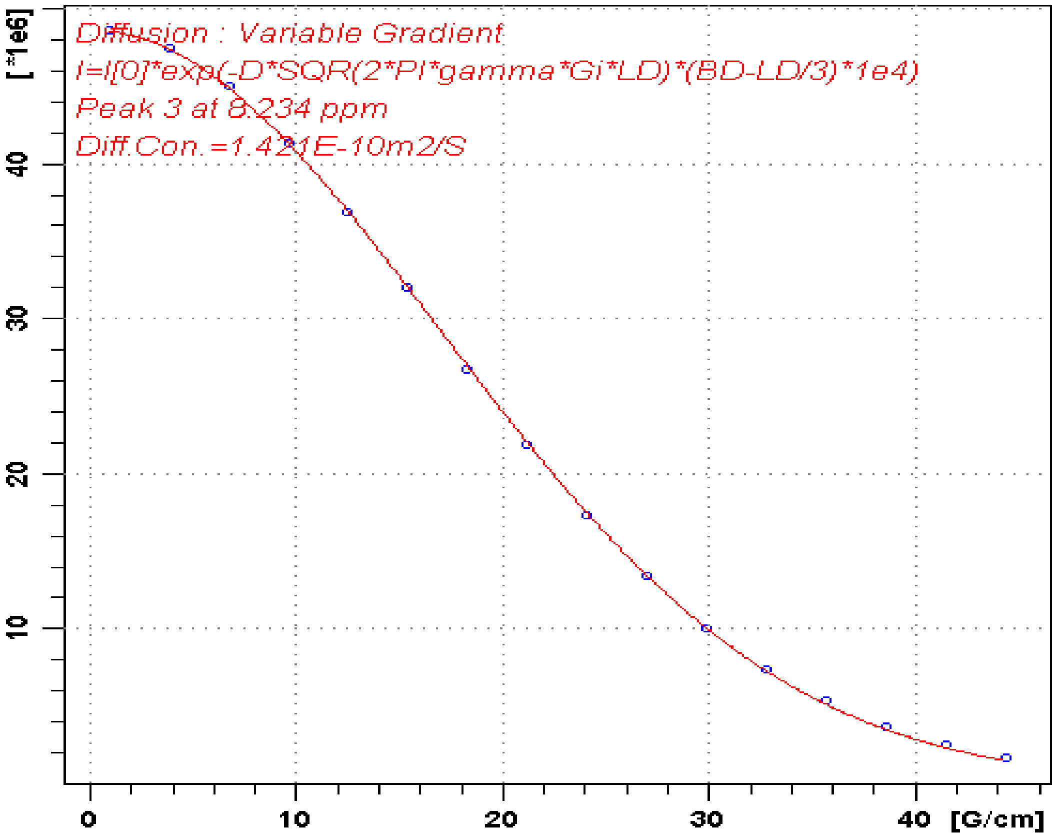

Supplementary Figure 27. Simulated diffusion decay curve for the signal H_b (peak at 8.23 ppm) on partially-complexed cages.

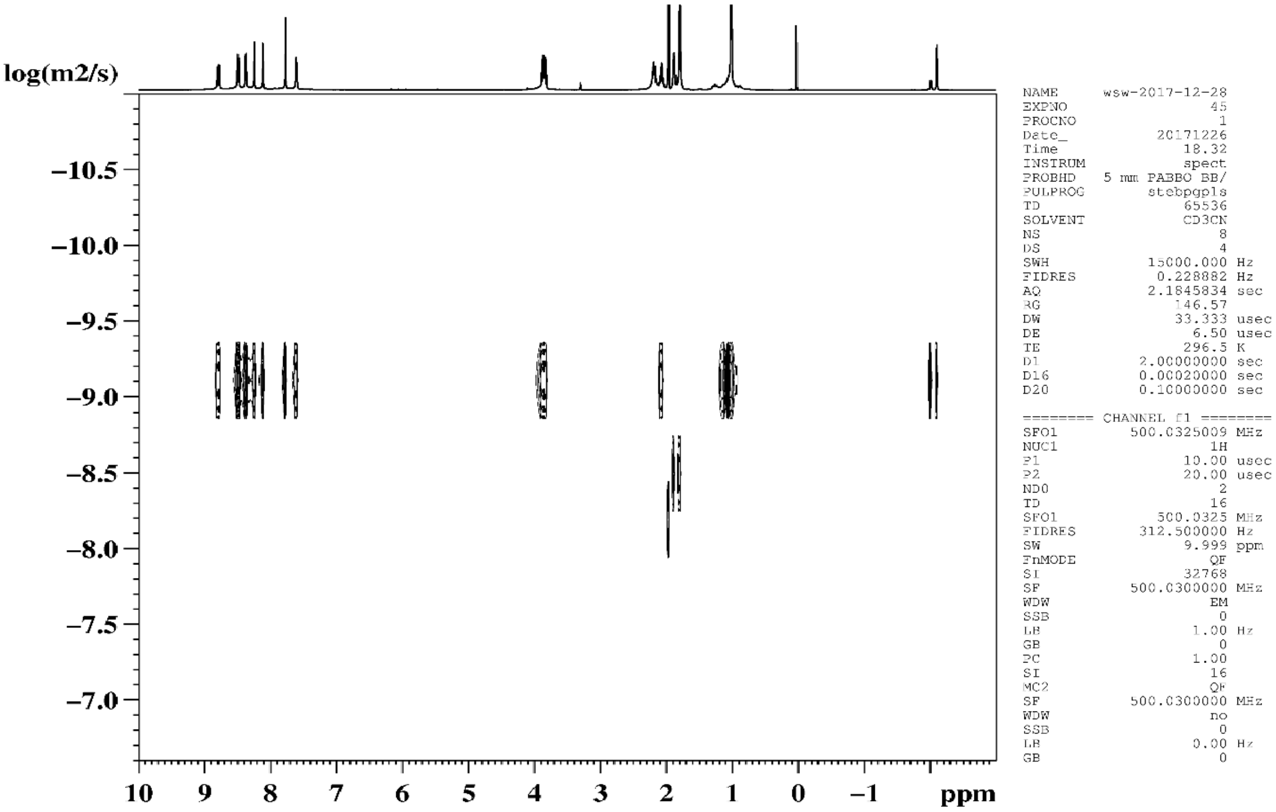

Supplementary Figure 28. Part of the DOSY spectrum (500 MHz, 298 K) of **Ad**⊂[Ag_5**1**$_2$]·$[OTf]_5$ (5 mM) in CD_3CN.

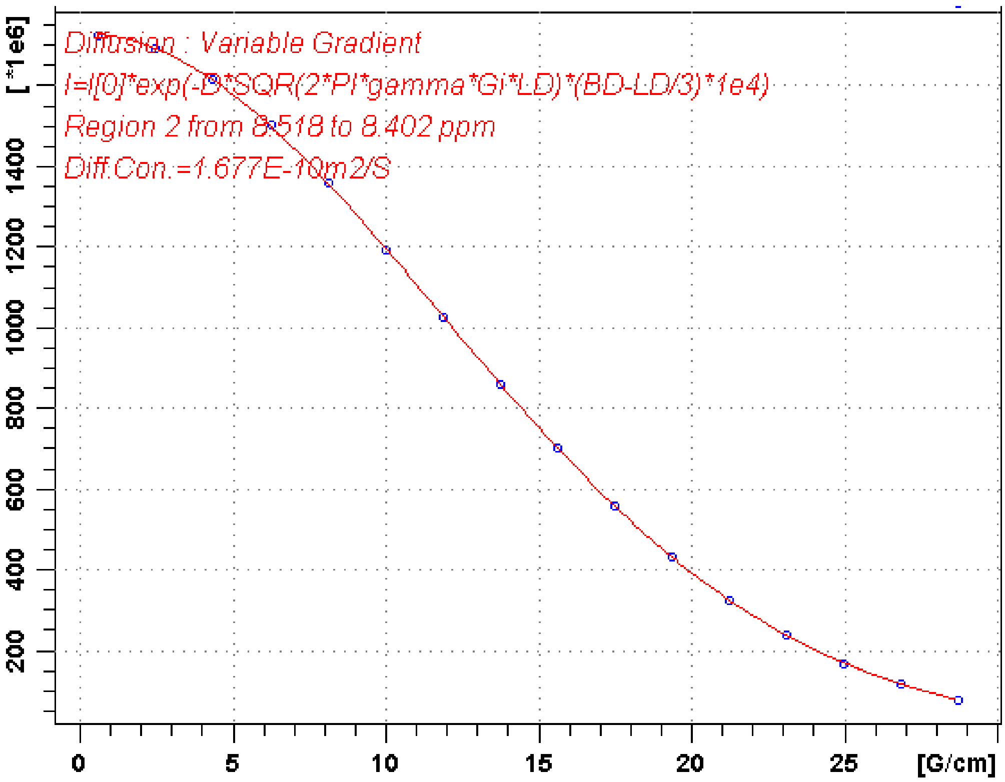

Supplementary Figure 29. Simulated diffusion decay curve for the signal H_d (peak at 8.47 ppm) of **Ad**⊂[Ag_5**1**$_2$]·$[OTf]_5$.

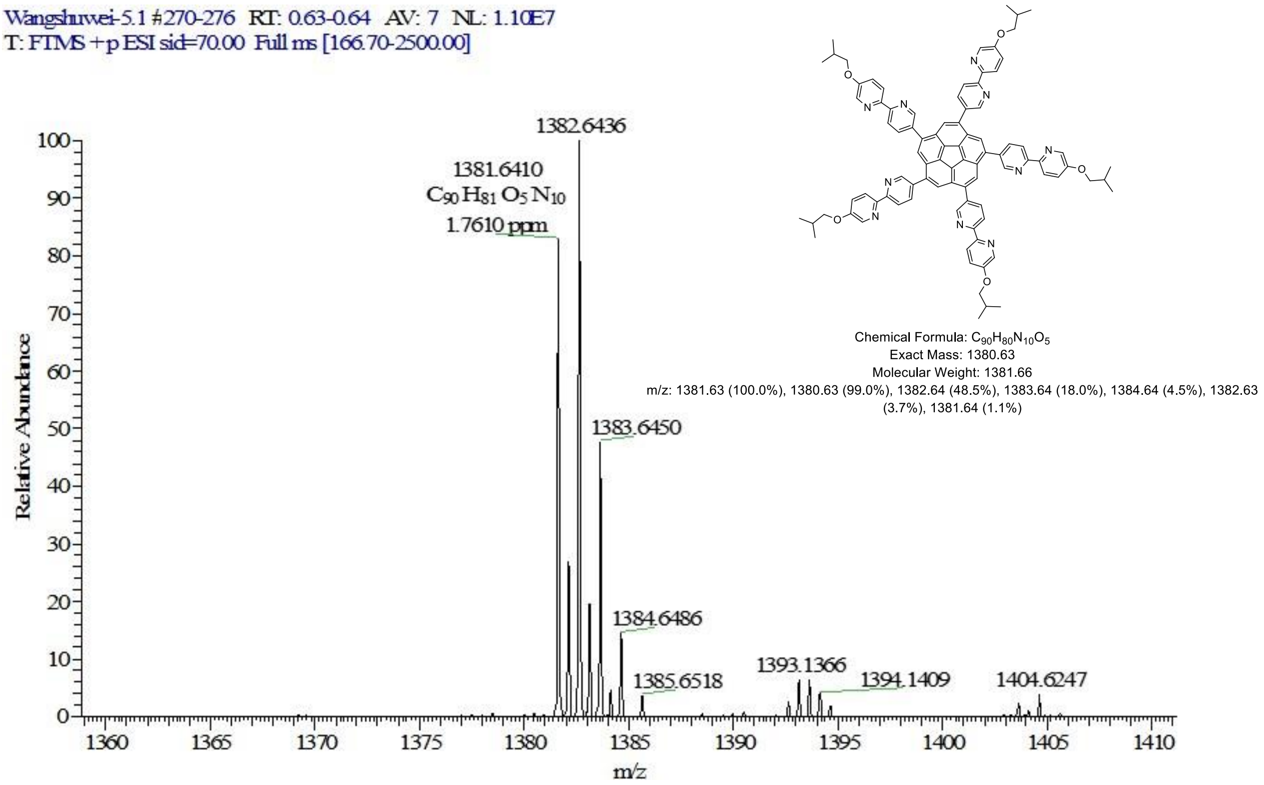

Supplementary Figure 30. ESI-MS spectra of the Ligand **1.**

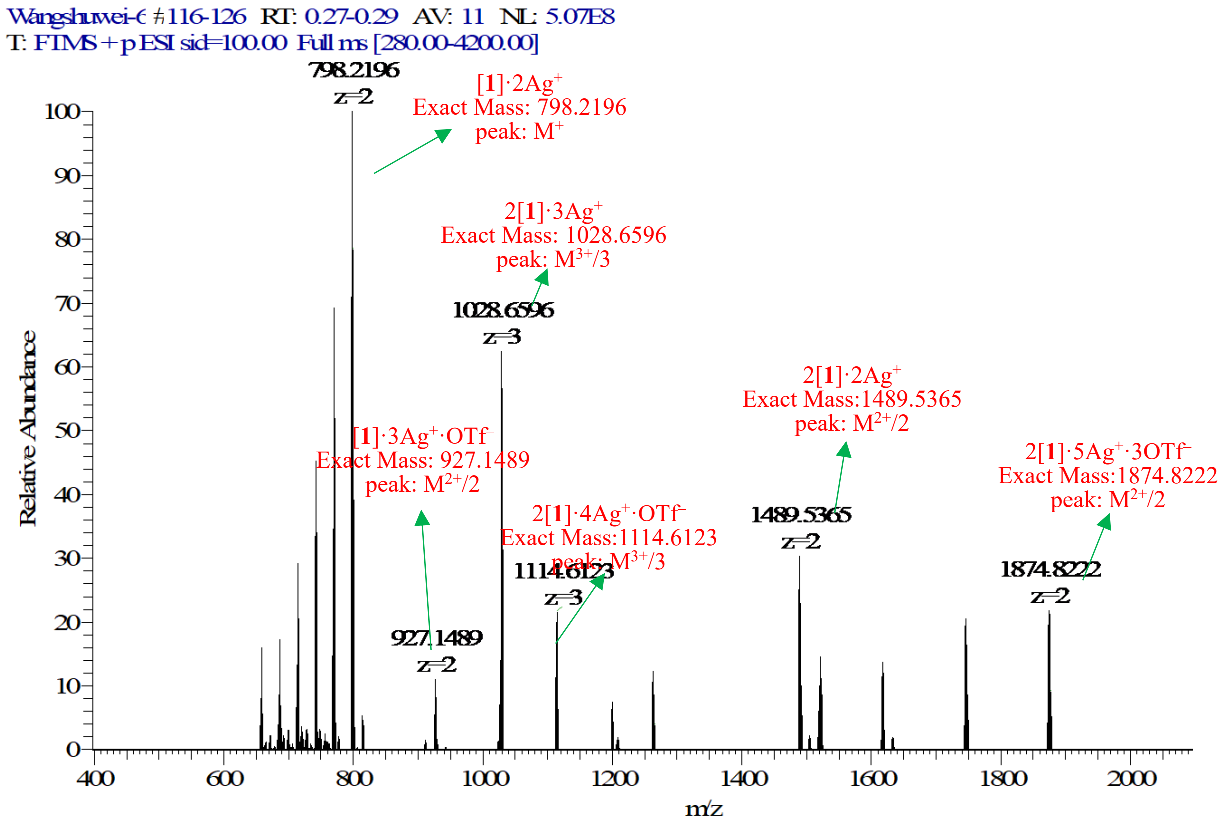

Supplementary Figure 31. ESI-MS spectra of the cage $[Ag_5\mathbf{1}_2]\cdot[OTf]_5$ ($\mathbf{Ag_5L_2}$). The experimental and simulated expanded isotopic clusters of some important species as well as the summary of the data are shown in Supplementary Table 5.

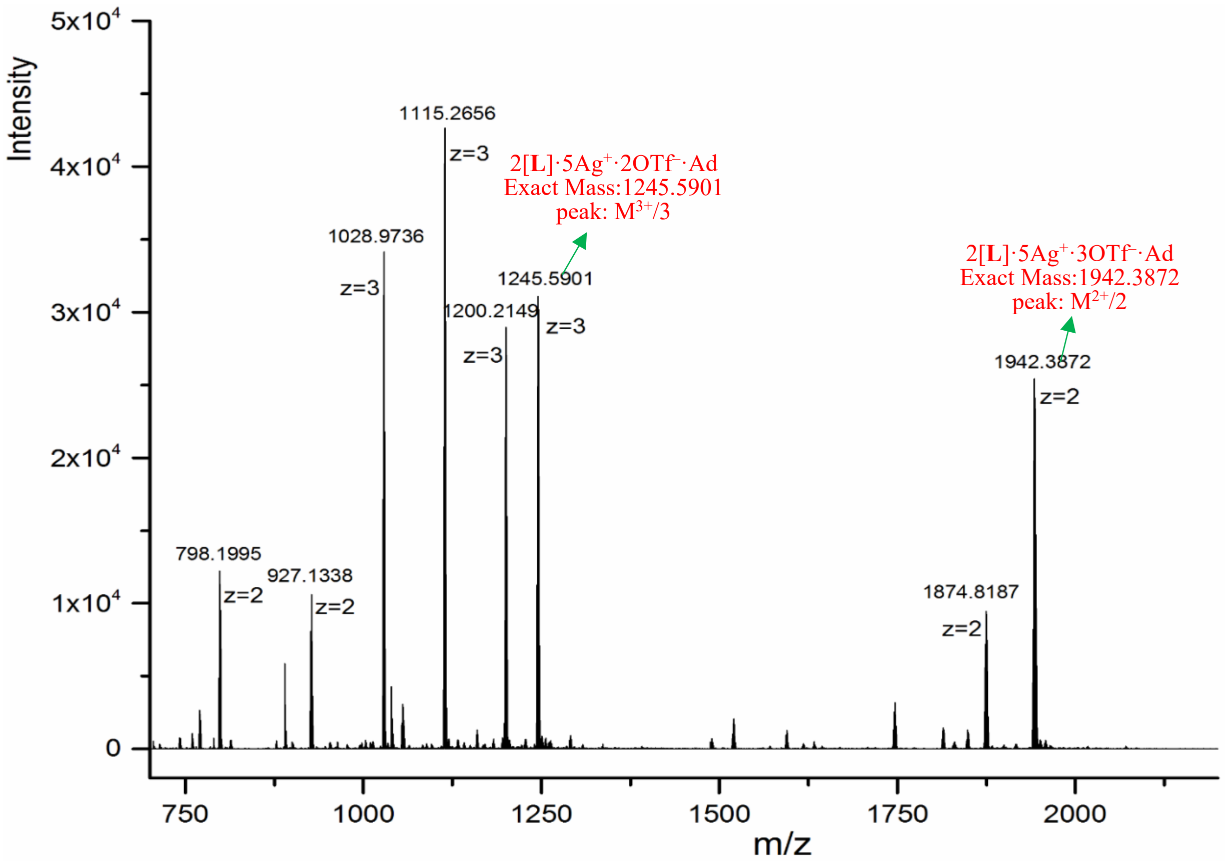

Supplementary Figure 32. ESI-MS spectra of **Ad**⊂[Ag$_5$**1**$_2$]·[OTf]$_5$ (**Ad⊂Ag$_5$L$_2$**). The experimental and simulated expanded isotopic clusters of some important species as well as the summary of the data are shown in Supplementary Table 5.

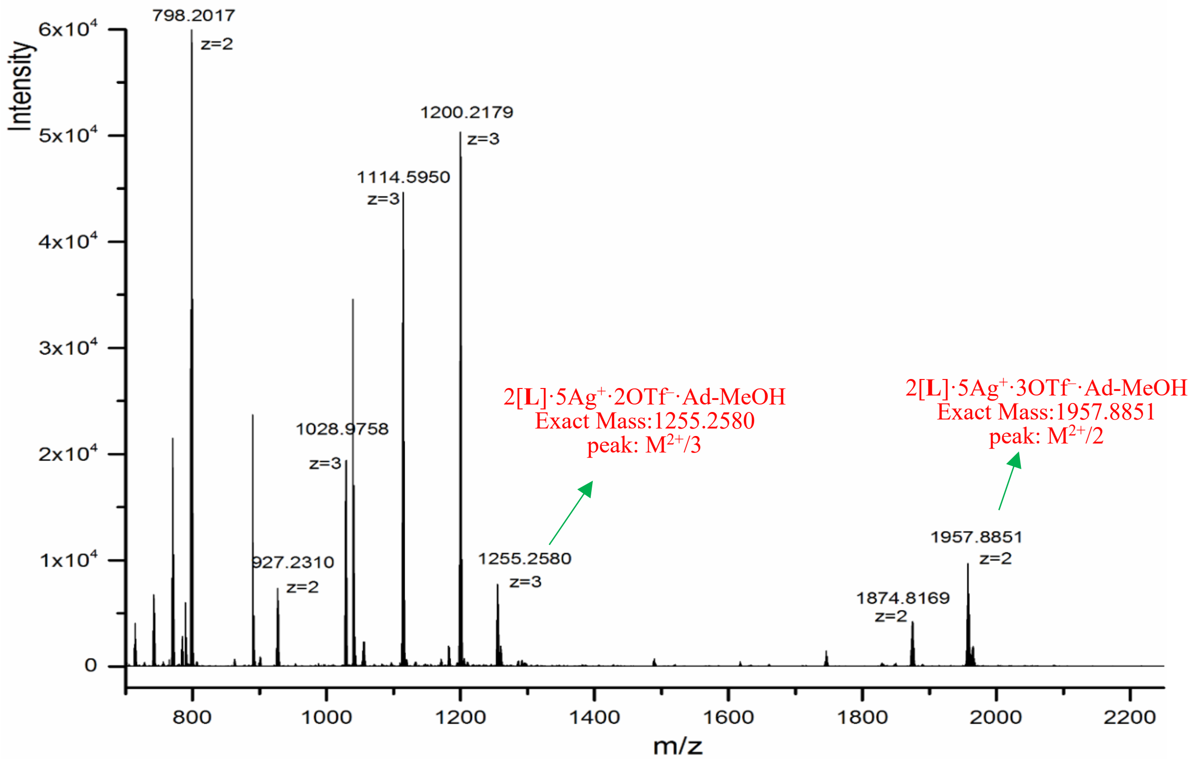

Supplementary Figure 33. ESI-MS spectrum of **Ad-MeOH**⊂[Ag$_5$**1**$_2$]·[OTf]$_5$. The experimental and simulated expanded isotopic clusters of some important species as well as the data are shown in Supplementary Table 5.

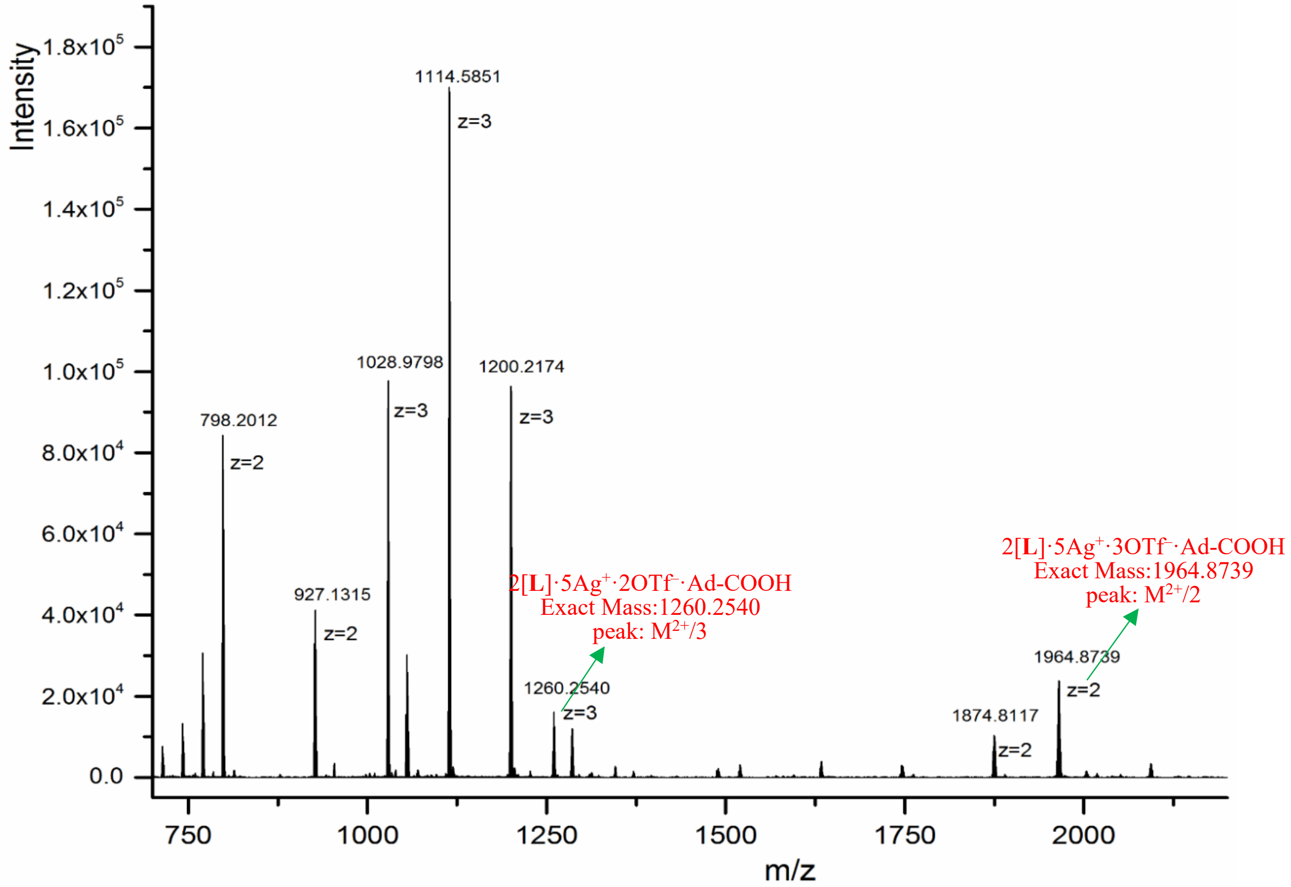

Supplementary Figure 34. ESI-MS spectrum of **Ad-COOH**⊂[Ag_5**1**$_2$]·[OTf]$_5$. The experimental and simulated expanded isotopic clusters of some important species as well as the data are shown in Supplementary Table 5.

Supplementary Table 5. Summary of the HRMS-ESI Data and the Expanded Isotopic Clusters for the Ag^+ Cage and its Complexes with Guests.

Species	Charge	Spectrum patterns	Chemical Formula	Data
2[**L**]·5Ag^+·3OTf^-	+2	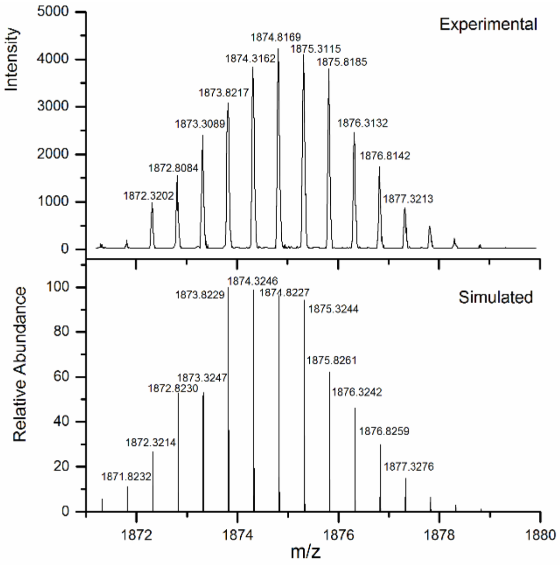	$C_{183}H_{160}N_{20}O_{19}F_9S_3Ag_5$ at M^{2+}	Found: 1874.8169 (100%, -5.8 ppm); 1875.3115 (96.9%, -6.9 ppm); 1874.3162 (90.7%, -4.5 ppm); 1875.8185 (89.7%, -4.1 ppm); 1873.8217 (72.5%, -0.6 ppm); 1876.3132 (58.2% -5.9 ppm); 1873.3089 (49.4%, -8.4 ppm); 1876.8142 (41.2%, -6.2 ppm).

2[**L**]·5Ag⁺·2OTf⁻	+3	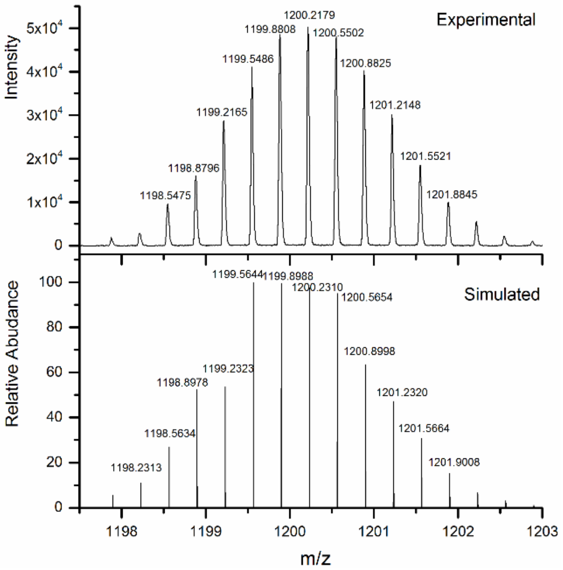	$C_{182}H_{160}N_{20}O_{16}F_6S_2Ag_5$ at M^{3+}	Found: 1200.2179 (100%, -10.9 ppm); 1199.8808 (96.5%, -15 ppm); 1200.5502 (95.4%, -12.7 ppm); 1199.5486 (81.5%, -13.2 ppm); 1200.8825 (80.0%, -14.4 ppm); 1201.2148 (59.9%, -14.3 ppm); 1199.2165 (57.1% -13.2 ppm); 1201.5521 (36.8%, -11.9 ppm); 1198.8796 (32.1%, -15.2 ppm).
2[**L**]·4Ag⁺·OTf⁻	+3	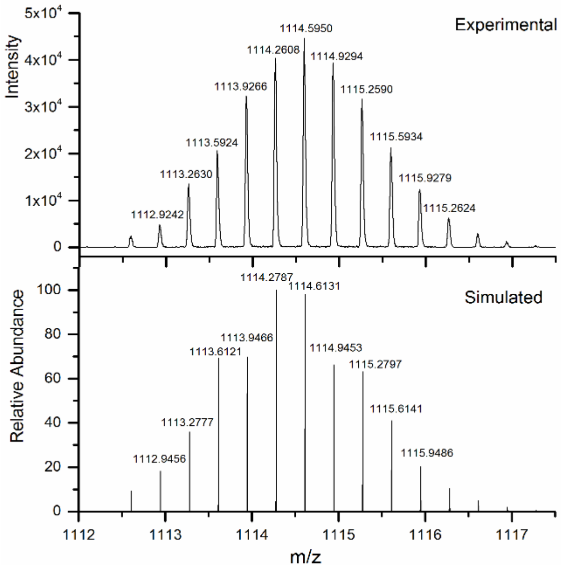	$C_{181}H_{160}N_{20}O_{13}F_3SAg_4$ at M^{3+}	Found: 1114.5950 (100%, -16.2 ppm); 1114.2608 (90.6%, -16.1 ppm); 1114.9294 (88.0%, -14.3 ppm); 1113.9266 (72.5%, -18.0ppm); 1115.2590 (70.9%, -18.6 ppm); 1115.5934 (47.5%, -18.6 ppm); 1113.5924 (46.3%, -17.7 ppm); 1113.2630 (30.3%, -13.2 ppm) ; 1115.9279 (27.8%, -18.5 ppm).
2[L]·3Ag⁺	+3	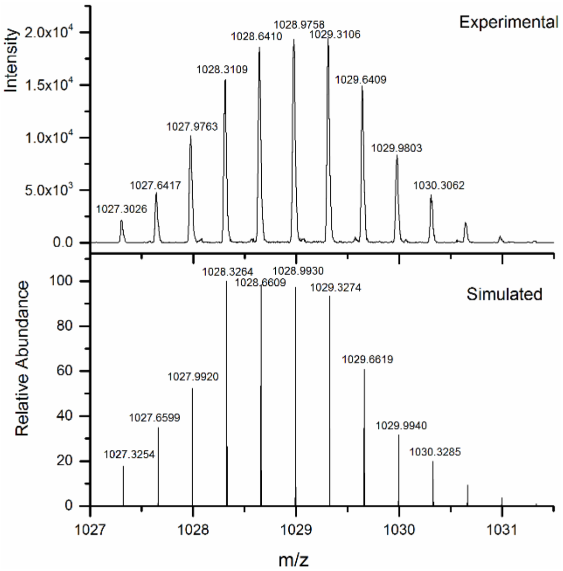	$C_{180}H_{160}N_{20}O_{10}Ag_3$ at M^{3+}	Found: 1029.3106 (100%, -16.3 ppm); 1028.9758 (99.5%, -16.7 ppm); 1028.6410 (95.7%, -19.3 ppm); 1028.3109 (80.0%, -15.1 ppm); 1029.6409 (76.7%, -20.4 ppm); 1027.9763 (52.2%, -15.3 ppm); 1029.9803 (42.9%, -13.3 ppm); 1027.6417 (24.6%, -17.7 ppm); 1030.3062 (23.5%, -21.6 ppm).

[**L**]·3Ag⁺·OTf⁻	+2	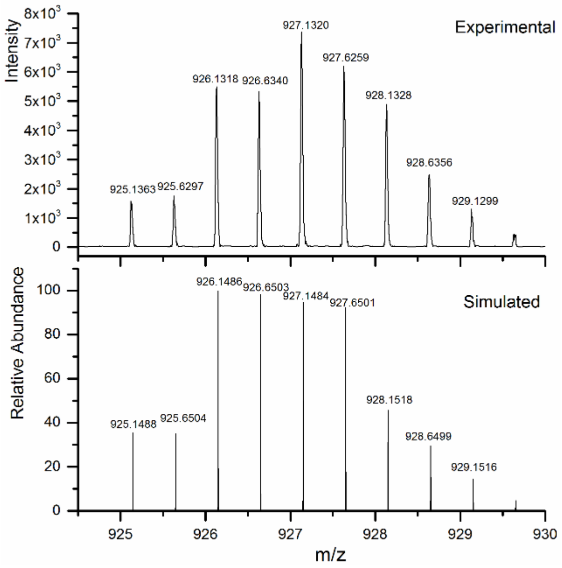	$C_{91}H_{80}N_{10}O_8F_3SAg_3$ at M^{2+}	Found: 927.1320 (100%, -17.7 ppm); 927.6259 (84.2%, -26.1 ppm); 926.1318 (74.8%, -18.1 ppm); 926.6340 (72.6%, -17.6 ppm); 928.1328 (66.5%, -20.5 ppm); 928.6356 (33.6%, -15.4 ppm); 925.6297 (23.9%, -22.4 ppm); 925.1363 (20.2%, -13.5 ppm); 929.1299 (17.8%, -23.4ppm).
[**L**]·2Ag⁺	+2	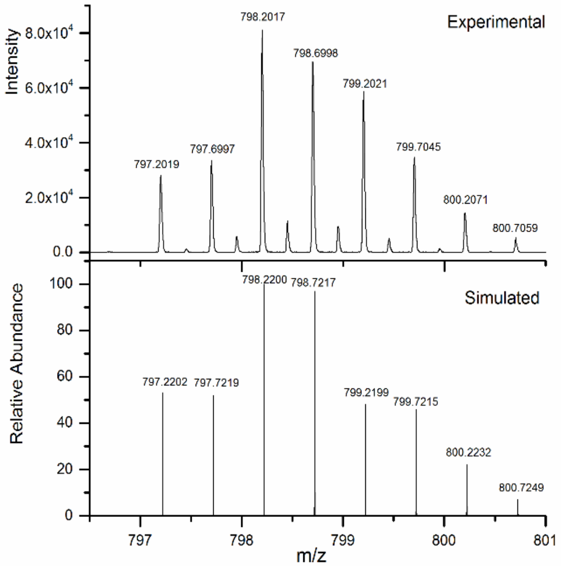	$C_{90}H_{80}N_{10}O_5Ag_2$ at M^{2+}	Found: 798.2017 (100%, -22.9 ppm); 798.6998 (85.8%, -27.4 ppm); 799.2021 (72.5%, -22.3 ppm); 799.7045 (42.7%, -21.2 ppm); 797.6997 (41.4%, -27.8 ppm); 797.2019 (34.6%, -22.9 ppm); 800.2071 (17.8%, -20.1 ppm); 800.7059 (6.5%, -23.7 ppm).
2[**L**] ·5Ag⁺·3OTf⁻· 1**Ad**	+2	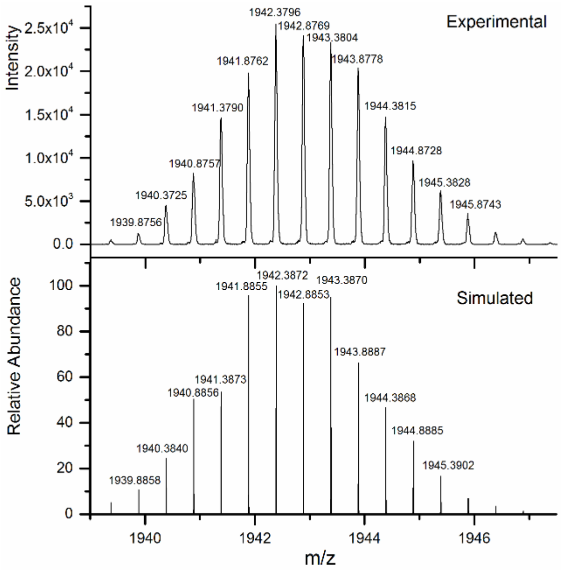	$C_{193}H_{176}N_{20}O_{19}F_9S_3Ag_5$ at M^{2+}	Found: 1942.3796 (100.0%, -3.9 ppm); 1942.8769 (94.8%, -4.3 ppm) ; 1943.3804 (91.8%, -3.4 ppm); 1943.8778 (80.1%, -5.6 ppm); 1941.8762 (77.8%, -4.8 ppm); 1944.3815 (57.8%, -2.7 ppm); 1941.3790 (57.5%, -4.3 ppm); 1944.8728 (38.0%, -8.1 ppm); 1940.8757 (32.3%, -5.1 ppm); 1945.3828 (24.4%, -3.8 ppm); 1940.3725 (17.9%, -5.9 ppm).

2[**L**] ·5Ag^+·2OTf⁻·1**Ad**	+3	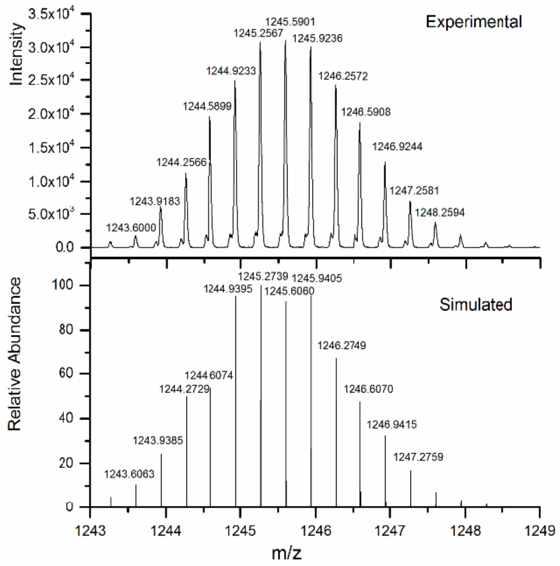	$C_{192}H_{176}N_{20}O_{16}F_6S_2Ag_5$ at M^{3+}	Found: 1245.5901 (100.0%, -12.7 ppm); 1245.2567 (99.1%, -13.8 ppm), 1245.9236 (97.0%, -13.6 ppm); 1244.9233 (80.1%, -13.0 ppm); 1246.2572 (78.2%, -14.2 ppm); 1244.5899 (63.2%, -14.1 ppm); 1246.5908 (60.3%, -13.0 ppm); 1246.9244 (41.4%, -13.7 ppm); 1244.2566 (36.3%, -13.1 ppm); 1247.2581 (22.9%, -14.3 ppm).
2[**L**] ·5Ag^+·3OTf⁻·1**Ad-MeOH**	+2	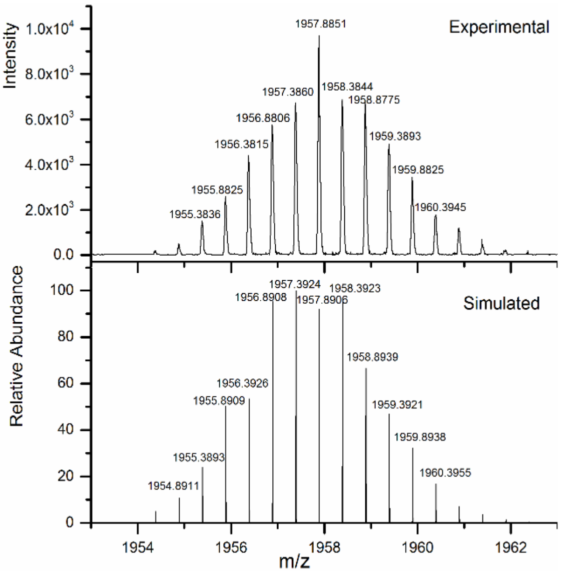	$C_{194}H_{178}N_{20}O_{20}F_9S_3Ag_5$ at M^{2+}	Found: 1957.8851 (100.0%, -2.8 ppm); 1958.3844 (70.7%, -4.0 ppm); 1958.8775 (70.5%, -8.4 ppm); 1957.3860 (69.4%, -3.3 ppm); 1956.8806 (59.2%, -5.2 ppm); 1959.3893 (50.5%, -1.4 ppm); 1956.3815 (43.5%, -5.7 ppm); 1959.8825 (35.4%, -5.8 ppm); 1955.8825 (26.6%, -4.3 ppm); 1960.3945 (18.6%, -0.5 ppm); 1955.3836 (15.6%, -2.9 ppm).
2[**L**] ·5Ag^+·2OTf⁻·1**Ad-MeOH**	+3	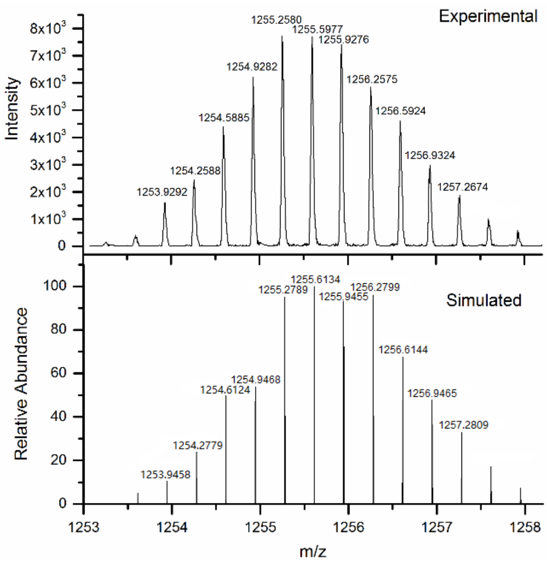	$C_{193}H_{178}N_{20}O_{17}F_6S_2Ag_5$ at M^{3+}	Found: 1255.2580 (100.0%, -16.6ppm); 1255.5977 (99.8%, -12.5 ppm); 1255.9276 (96.1%, -14.3 ppm); 1254.9282 (80.3%, -14.8 ppm); 1256.2575 (75.7%, -17.8 ppm); 1256.5924 (59.8%, -17.5 ppm); 1254.5885 (56.6%, -19.1 ppm); 1256.9324 (38.5%, -11.2 ppm); 1254.2588 (31.6%, -15.2 ppm); 1257.2674 (24.4%, -10.7 ppm); 1253.9292 (20.8%, -13.2 ppm).

2[**L**] ·5Ag^{+}·3OTf^{-}·<br>1**Ad-COOH**	+2	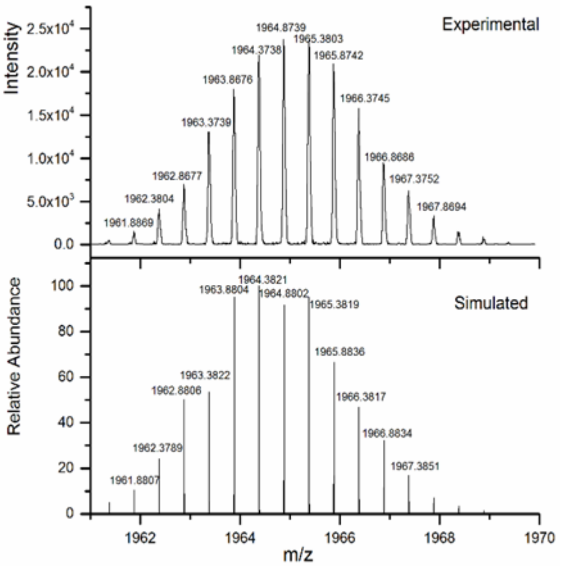	$C_{194}H_{176}N_{20}O_{21}F_9S_3Ag_5$ at M^{2+}	Found: 1964.8739 (100.0%, -3.2 ppm); 1965.3803 (99.8%, -0.8 ppm); 1964.3738 (92.4%, -4.2 ppm); 1965.8742 (88.1%, -4.8 ppm); 1963.8676 (75.6%, -6.5 ppm); 1966.3745 (66.3%, -3.7 ppm); 1963.3739 (54.9%, -4.2 ppm); 1966.8686 (39.8%, -7.5 ppm); 1962.8677 (29.5%, -6.6 ppm); 1967.3752 (26.0%, -5.0 ppm); 1962.3804 (17.3%, 0.8 ppm).
2[**L**] ·5Ag^{+}·2OTf^{-}-·<br>1**Ad-COOH**	+3	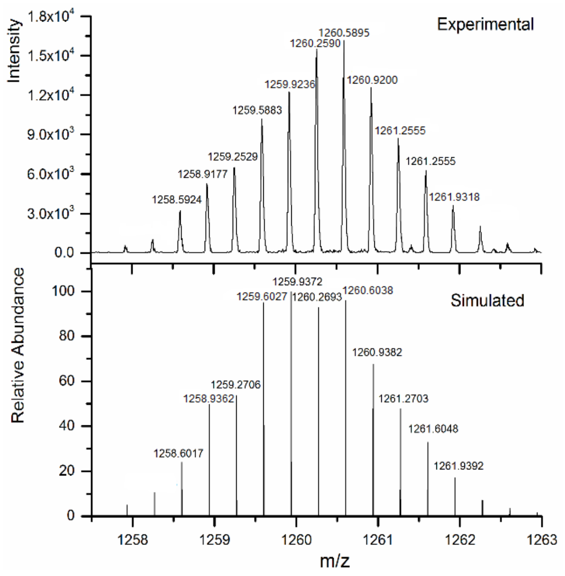	$C_{193}H_{176}N_{20}O_{18}F_6S_2Ag_5$ at M^{3+}	Found: 1260.5895 (100%, -11.3 ppm); 1260.2590 (93.6%, -8.2 ppm); 1260.9200 (78.0%, -14.4 ppm); 1259.9236 (75.7%, -10.8 ppm); 1259.5883 (63.2%, -11.4 ppm); 1261.2555 (54.0%, -11.7 ppm); 1259.2529 (40.3%, -14.0 ppm); 1258.9177 (32.8%, -14.7 ppm); 1258.5924 (19.9%, -7.4 ppm)); 1261.9318 (18.4%, -5.9 ppm).

Supplementary Method 8. Kinetic Studies on Guest Releases from **Ag$_5$L$_2$** in CDCl$_2$CDCl$_2$

^{1}H NMR Experiments. Samples of guest⊂[Ag$_5$**1**$_2$]·[OTf]$_5$ (guest⊂**Ag$_5$L$_2$**) were first prepared in CD$_3$CN according to the method described in Supplementary Method 3–5 in this Supplementary Information. The solutions were allowed to evaporate to dryness and evacuated under high vacuum. The residues were re-dissolved in 1,1,2,2-tetrachloroethane-d_2 (CDCl$_2$CDCl$_2$; m.p. –45 °C, b.p. 146 °C), and the NMR tubes were then positioned in the NMR cavity rapidly. After the samples were maintained at the stated temperature for 3~5 minutes, a series of ^{1}H NMR spectra were obtained. The obtained ^{1}H NMR spectra in the cases of **Ad**, **Ad-MeOH** and **Ad-COOH** are shown in Supplementary Figures 35–38, 41–44 and 47–50.

Release Kinetics. First-order release rate constants, k, are basically obtained by monitoring the decay of proton signal at ca. 7.9 ppm (corresponding to the guest-cage complexes) and the thriving of the signal at ca. 8.0 ppm (corresponding to the free cages). Exact release rates (k) are given by Supplementary Eq. S5,

$$\ln c_{\text{capsule},0} - \ln c_{\text{cage,prd}} = kt \tag{S5}$$

where $c_{\text{capsule},0}$ expresses the initial concentration of the capsule with guest encapsulated at the moment defined as $t = 0$, $c_{\text{cage,prd}}$ the analytical concentration of the free cage in the decay.

Using the rates of release obtained, the enthalpic ($\Delta H^{\ddagger}$) and entropic ($\Delta S^{\ddagger}$) contributions to the transition state were calculated based on Eyring equation (Supplementary Eq. S6)

$$\ln\frac{k}{T} = -\frac{\Delta H^{\ddagger}}{R}\cdot\frac{1}{T} + \ln\frac{k_{\text{B}}}{h} + \frac{\Delta S^{\ddagger}}{R} \tag{S6}$$

where k is the exchange rate constant, T the absolute temperature, $\Delta H^{\ddagger}$ enthalpy of activation, R the universal gas constant (1.9859 $\text{cal}\cdot\text{mol}^{-1}\cdot\text{K}^{-1}$), k_{B} the Boltzmann constant (3.2976×10^{-24} $\text{cal}\cdot\text{K}^{-1}$), h the Planck's constant (1.5827×10^{-34} $\text{cal}\cdot\text{s}$), $\Delta S^{\ddagger}$ the entropy of activation.

The free energy of activation ($\Delta G^{\ddagger}$) was determined from,

$$\Delta G^{\ddagger} = \Delta H^{\ddagger} - \text{T}\Delta S^{\ddagger} \tag{S7}$$

using the activation enthalpy ($\Delta H^{\ddagger}$) and the entropy ($\Delta S^{\ddagger}$) derived from the Eyring plots. The $\Delta G^{\ddagger}$ values at 298 K for the release systems were calculated. A linear extrapolation method was used for the calculations, based on an assumption that the enthalpy and the entropy terms remain constant over a wide temperature range.

Summaries of the obtained kinetic and the thermodynamic parameters are shown in Supplementary Table 6. Corresponding fits to the first order kinetics and the Eyring plots are shown in Supplementary Figures 39–40, 45–46 and 51–52. Original data derived from the time-dependent ^{1}H NMR spectra are summarized in Supplementary Tables 8–10.

In all the cases the Eyring plots displayed a high degree of linearity in the range of temperatures examined. Considering the time-dependent ^{1}H NMR experiments were carried out in different range of temperatures for different guest, for comparison, we also calculated/listed the free energy of activation ($\Delta G^{\ddagger}$) at 298 K for all the release processes, using a linear extrapolation method in the cases of **Ad-MeOH** and **Ad-COOH**. Those obtained $\Delta G^{\ddagger}$ values, as well as the enthalpy ($\Delta H^{\ddagger}$) and entropy ($\text{T}\Delta S^{\ddagger}$) contributions to the $\Delta G^{\ddagger}$, are summarized in Supplementary Table 7.

Supplementary Table 6. Summary of Release Kinetics for **Ad**, **Ad-MeOH** and **Ad-COOH** from the Silver Cages ([Ag_5**1**$_2$]·[OTf]$_5$) in $CDCl_2CDCl_2$.[a]

Guest	T (K)	k [b] (s^{-1})	$t_{1/2}$ [c] (s)	ln(k/T)	$\Delta G^{\ddagger}$ (kcal·mol^{-1})	$\Delta H^{\ddagger}$ (kcal·mol)$^{-1}$	$\Delta S^{\ddagger}$ (cal·mol^{-1}·K^{-1})
**Ad**	288	3.08×10^{-5}	2.25×10^{4}	-16.0510	22.71	45.21	78.16
	292	1.31×10^{-4}	5.29×10^{3}	-14.6178	22.39		
	297	3.32×10^{-4}	2.09×10^{3}	-13.7033	22.00		
	301	1.14×10^{-3}	6.08×10^{2}	-12.4850	21.69		
**Ad-MeOH**	279	3.08×10^{-5}	2.25×10^{4}	-16.0193	22.08	36.17	50.49
	284	8.52×10^{-5}	8.14×10^{3}	-15.0196	21.83		
	288	2.18×10^{-4}	3.18×10^{3}	-14.0919	21.62		
	292	5.90×10^{-4}	1.17×10^{3}	-13.1119	21.42		
**Ad-COOH**	274	4.00×10^{-5}	1.73×10^{4}	-15.7398	21.47	15.36	-22.31
	279	7.44×10^{-5}	9.32×10^{3}	-15.1373	21.58		
	284	1.22×10^{-4}	5.68×10^{3}	-14.6605	21.69		
	288	1.65×10^{-4}	4.20×10^{3}	-14.3725	21.78		

[a] Data were derived from the kinetic ^{1}H NMR studies. [b] Release rate at the given temperature. [c] Half-life for the release kinetics.

Supplementary Table 7. Calculated Activation Parameters for the Release of the Guest at 298 K.

Guest	k (s^{-1})	$\Delta G^{\ddagger}$ (kcal·mol^{-1})	$\Delta H^{\ddagger}$ (kcal·mol^{-1})	T$\Delta S^{\ddagger}$ (kcal·mol^{-1})
**Ad**	5.11×10^{-4}	21.92	45.21	23.29
**Ad-MeOH**	1.97×10^{-3}	21.12	36.17	15.05
**Ad-COOH**	4.42×10^{-4}	22.00	15.36	-6.65

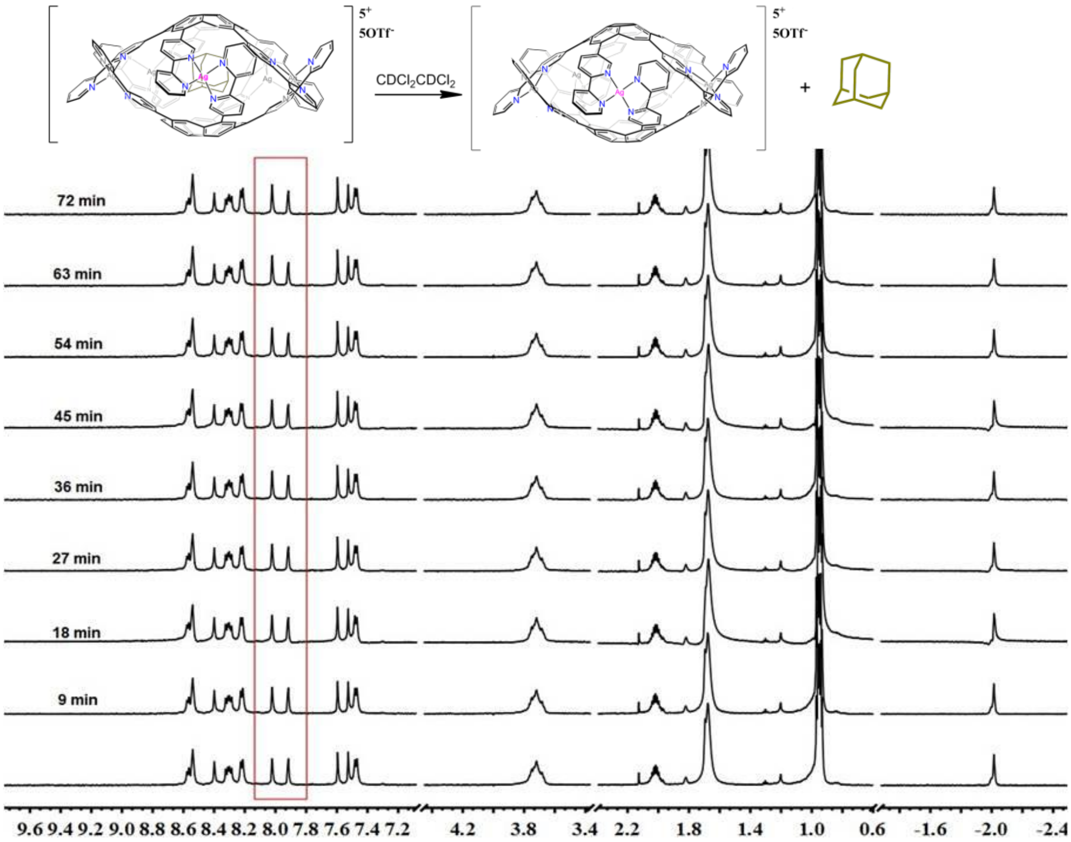

Supplementary Figure 35. Time-dependent ^{1}H NMR spectra (600 MHz, **288** K) of the mixture of $[Ag_5\mathbf{1}_2]\cdot[OTf]_5$ and **Ad**$\subset[Ag_5\mathbf{1}_2]\cdot[OTf]_5$ in $CDCl_2CDCl_2$.

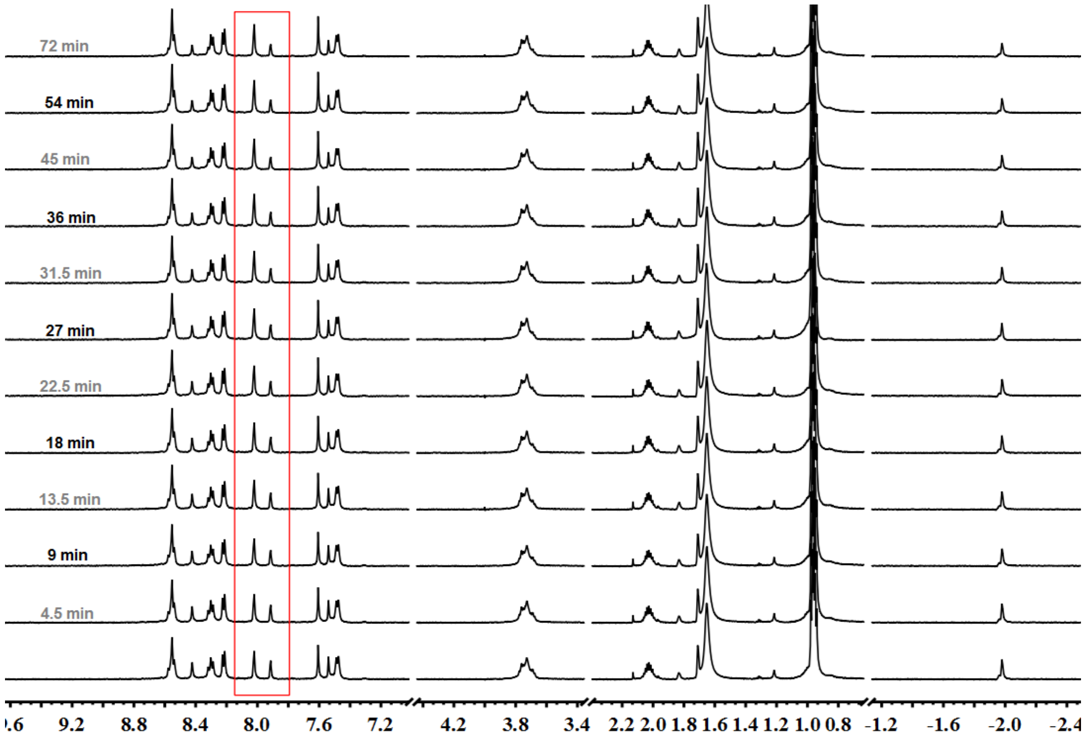

Supplementary Figure 36. Time-dependent ^{1}H NMR spectra (600 MHz, **292** K) of the mixture of $[Ag_5\mathbf{1}_2]\cdot[OTf]_5$ and **Ad**$\subset[Ag_5\mathbf{1}_2]\cdot[OTf]_5$ in $CDCl_2CDCl_2$.

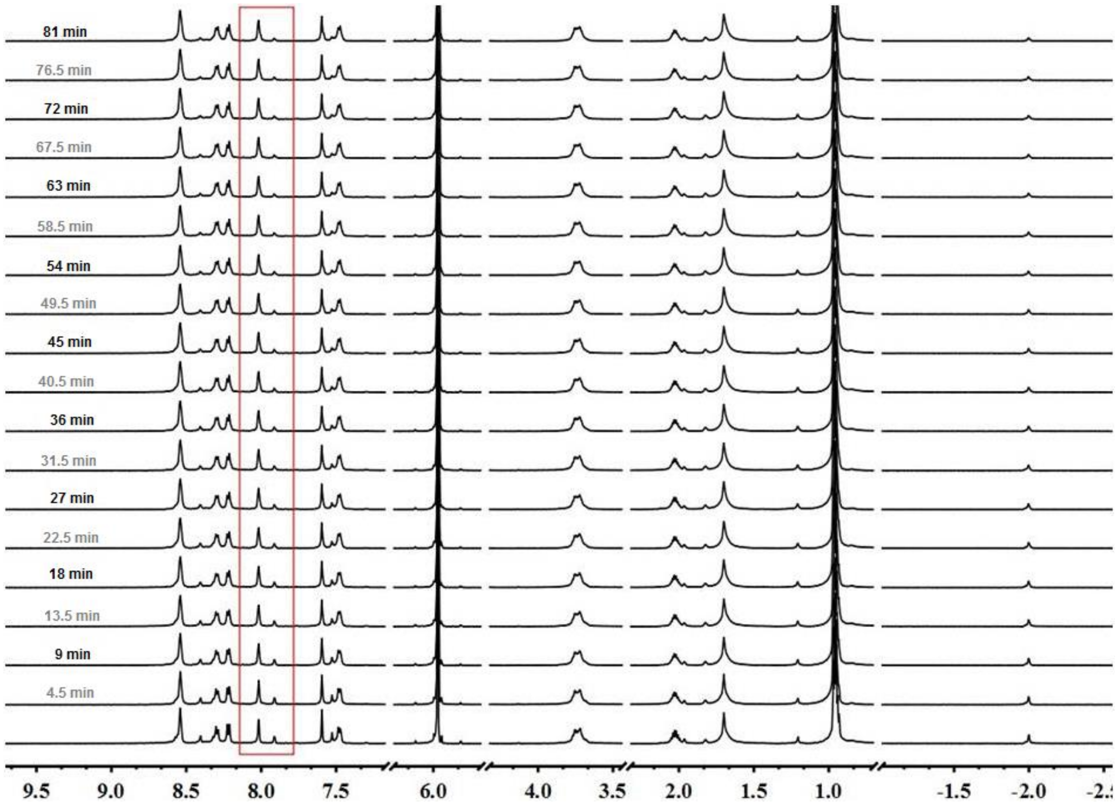

Supplementary Figure 37. Time-dependent ^{1}H NMR spectra (600 MHz, **297** K) of the mixture of $[Ag_5\mathbf{1}_2]\cdot[OTf]_5$ and $\mathbf{Ad}\subset[Ag_5\mathbf{1}_2]\cdot[OTf]_5$ in $CDCl_2CDCl_2$.

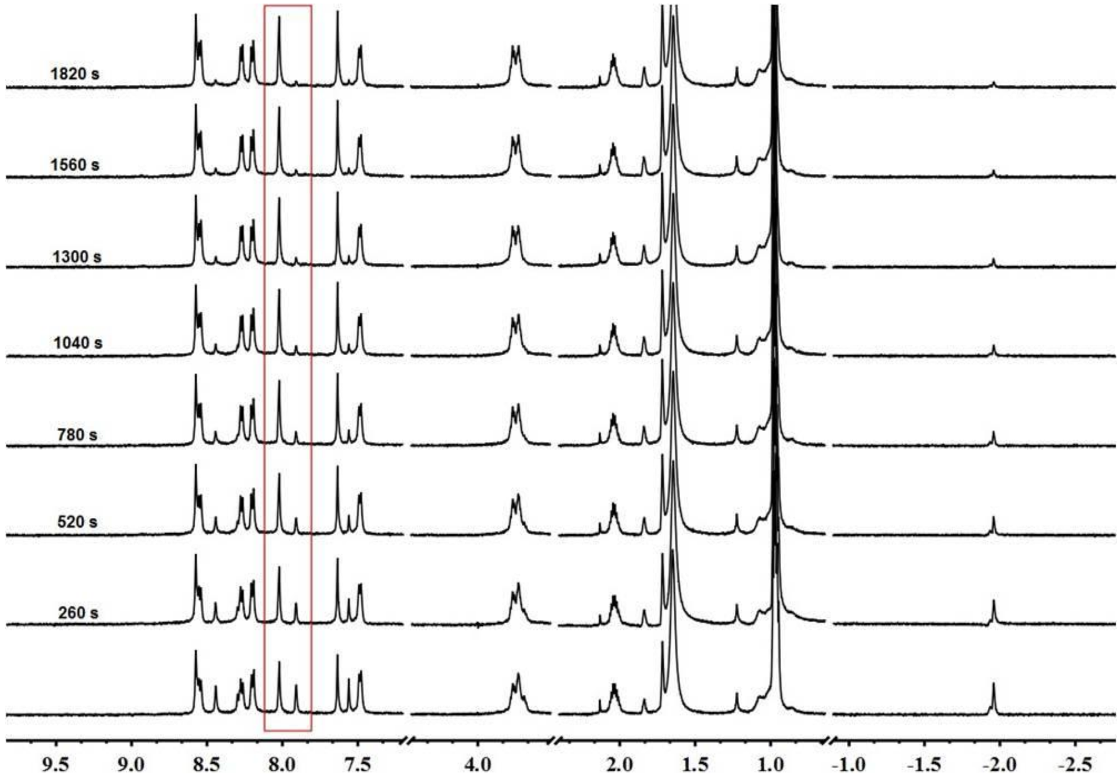

Supplementary Figure 38. Time-dependent ^{1}H NMR spectra (600 MHz, **301** K) of the mixture of $[Ag_5\mathbf{1}_2]\cdot[OTf]_5$ and $\mathbf{Ad}\subset[Ag_5\mathbf{1}_2]\cdot[OTf]_5$ in $CDCl_2CDCl_2$.

Supplementary Table 8. The calculations for **Ad** release kinetics in $CDCl_2CDCl_2$ at different temperatures: the concentration of the capsule with guest encapsulated ($c_{capsule}$), concentration of the free cage produced in the decay ($c_{cage,prd}$), and ln($c_{capsule}$) at different time. The data were derived from the time-dependent ^{1}H NMR spectra (Supplementary Figures 35–38).

288K	*t* (s)	0	528	1054	1574	2094	2612	3138	3664
	$c_{cage,prd}$	0.543	0.552	0.562	0.568	0.575	0.581	0.588	0.592
	$c_{capsule}$	0.457	0.448	0.438	0.432	0.425	0.419	0.412	0.408
	ln($c_{capsule}$)	-0.784	-0.804	-0.825	-0.840	-0.855	-0.871	-0.887	-0.896
292K	t (s)	0	260	520	780	1040	1300	1560	1820
	$c_{cage,prd}$	0.645	0.654	0.662	0.671	0.680	0.694	0.709	0.714
	$c_{capsule}$	0.355	0.346	0.338	0.329	0.320	0.306	0.291	0.286
	ln($c_{capsule}$)	-1.036	-1.060	-1.085	-1.112	-1.140	-1.186	-1.235	-1.253
297K	t (s)	0	260	520	780	1040	1300	1560	1820
	$c_{cage,prd}$	0.800	0.820	0.833	0.847	0.855	0.870	0.877	0.893
	$c_{capsule}$	0.200	0.180	0.167	0.153	0.145	0.130	0.123	0.107
	ln($c_{capsule}$)	-1.609	-1.713	-1.792	-1.880	-1.929	-2.037	-2.097	-2.234
301K	t (s)	0	260	520	780	1040	1300	1560	1820
	$c_{cage,prd}$	0.654	0.746	0.813	0.862	0.901	0.919	0.939	0.959
	$c_{capsule}$	0.346	0.254	0.187	0.138	0.099	0.081	0.061	0.041
	ln($c_{capsule}$)	-1.060	-1.371	-1.677	-1.981	-2.312	-2.515	-2.796	-3.189

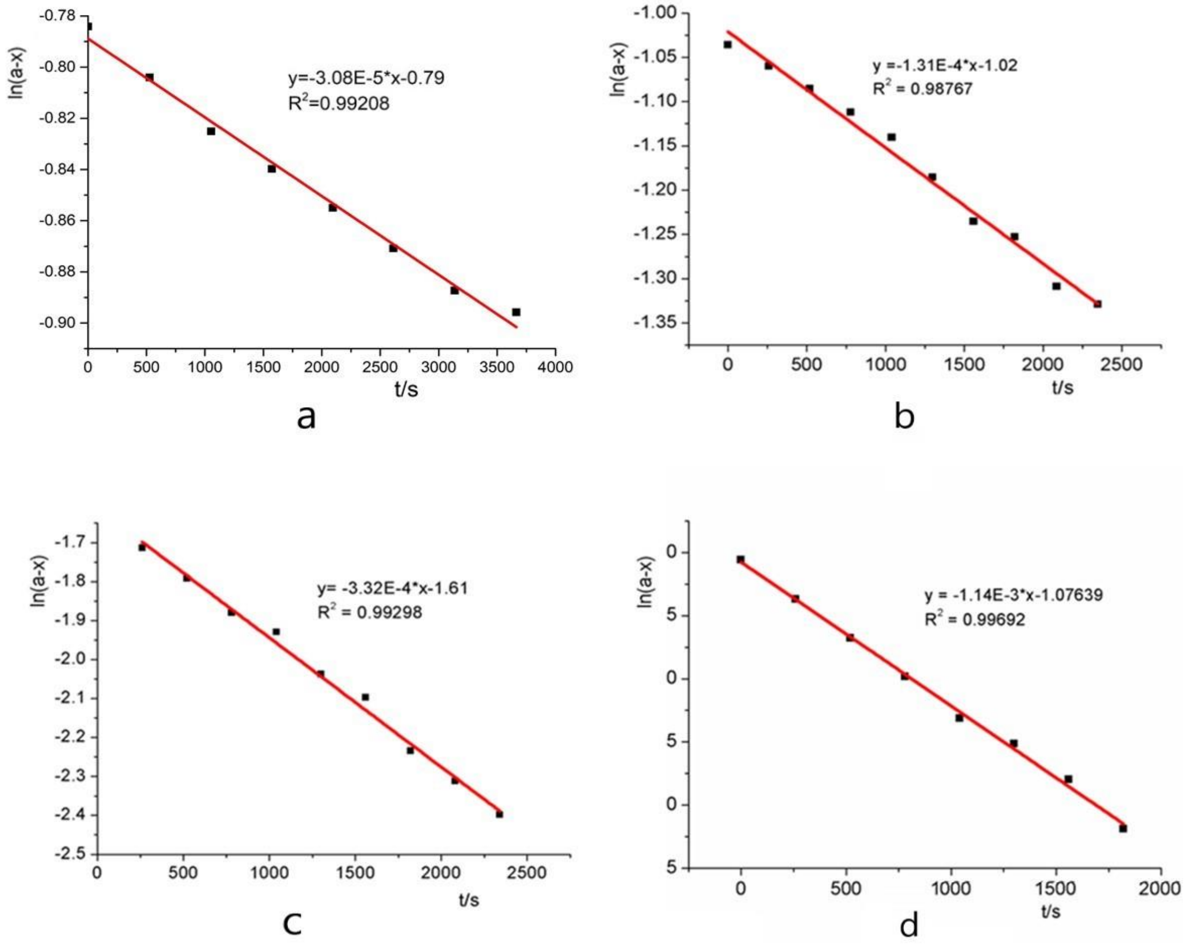

Supplementary Figure 39. Release kinetics for **Ad** from $[Ag_5\mathbf{1}_2]\cdot[OTf]_5$ in $CDCl_2CDCl_2$ at different temperature: fits to the first order kinetics as measures of relative concentration of **Ad**$\subset[Ag_5\mathbf{1}_2]\cdot[OTf]_5$. (a) 288K; (b) 292 K; (c) 297 K; (d) 301 K.

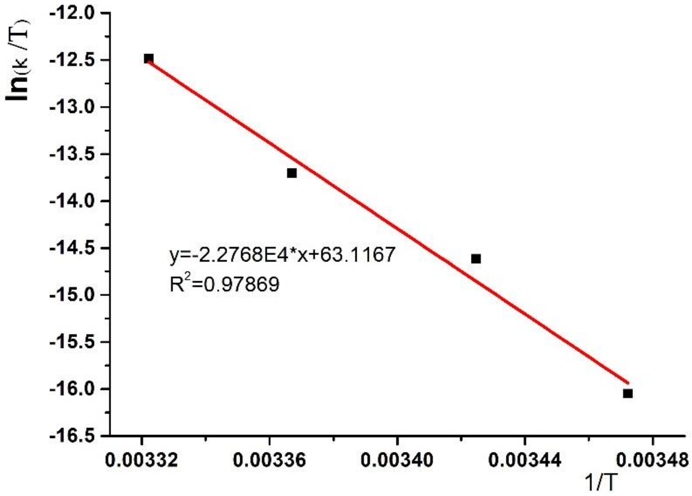

Supplementary Figure 40. Release kinetics for **Ad** from $[Ag_5\mathbf{1}_2]\cdot[OTf]_5$ in $CDCl_2CDCl_2$: numerical fit to the Eyring equations. The first order rate constant (k) were obtained from Supplementary Figure 39.

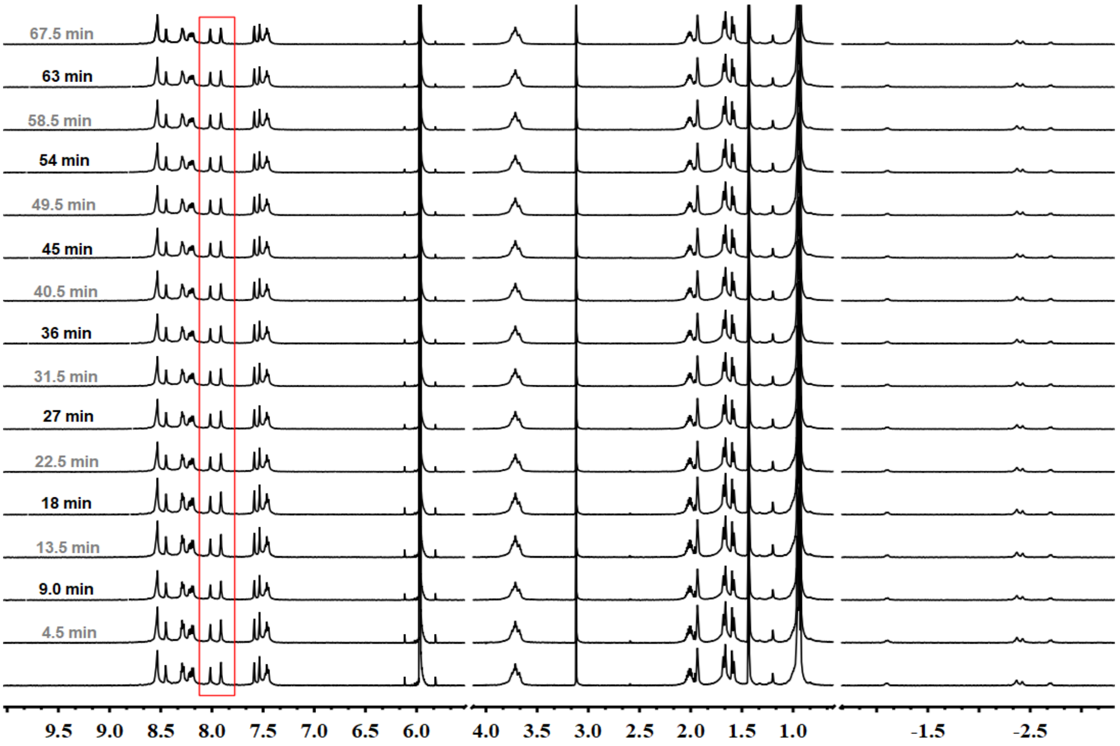

Supplementary Figure 41. Time-dependent ^{1}H NMR spectra (600 MHz, **279** K) of the mixture of $[Ag_5\mathbf{1}_2]\cdot[OTf]_5$ and **Ad-MeOH**$\subset[Ag_5\mathbf{1}_2]\cdot[OTf]_5$ in $CDCl_2CDCl_2$.

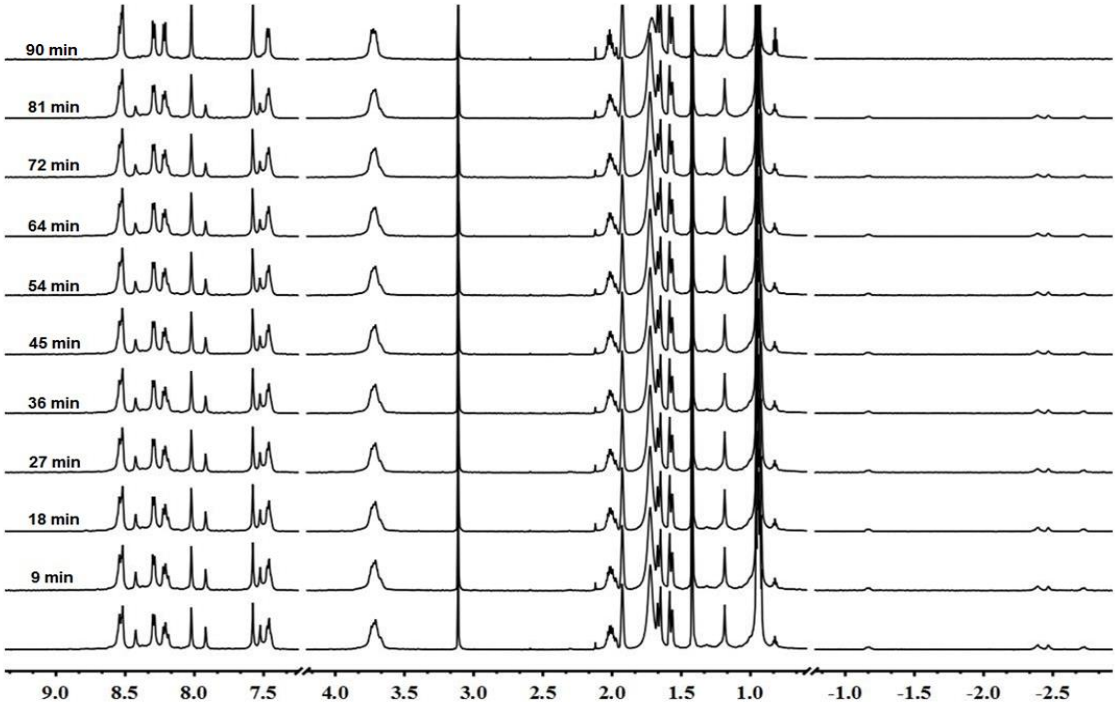

Supplementary Figure 42. Time-dependent ^{1}H NMR spectra (600 MHz, **284** K) of the mixture of $[Ag_5\mathbf{1}_2]\cdot[OTf]_5$ and **Ad-MeOH**$\subset[Ag_5\mathbf{1}_2]\cdot[OTf]_5$ in $CDCl_2CDCl_2$.

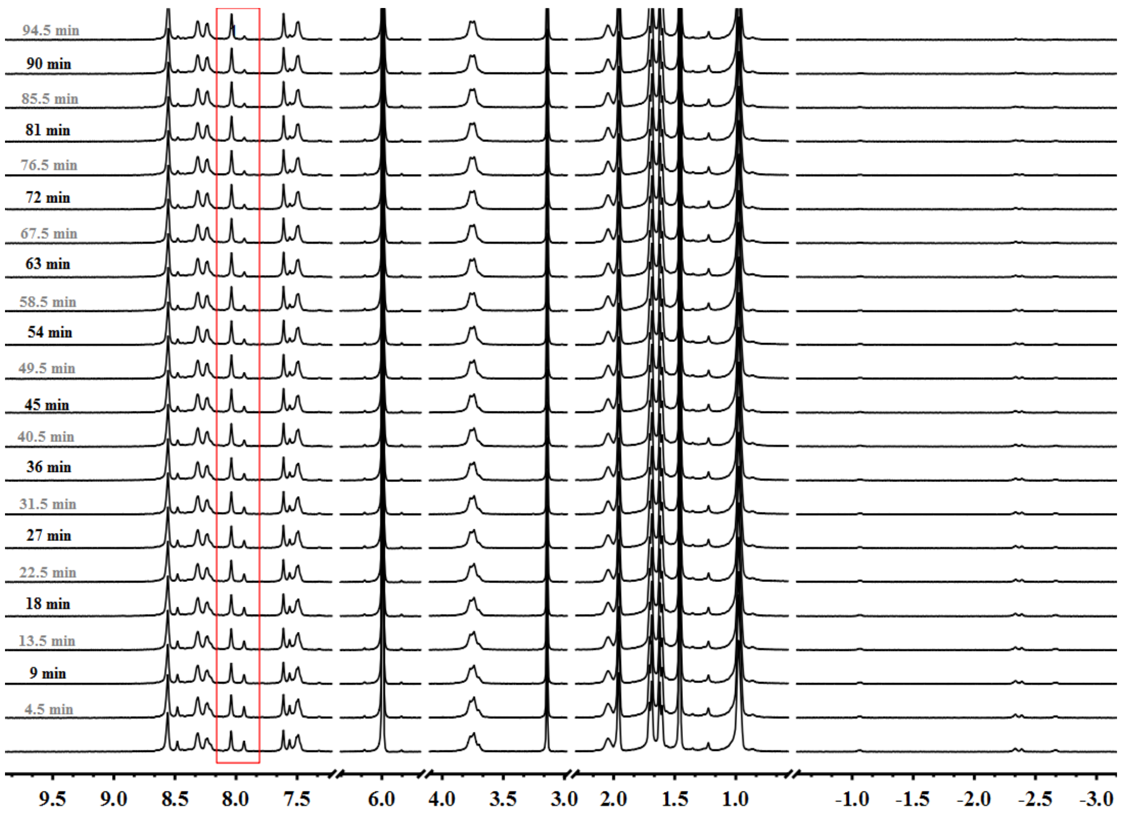

Supplementary Figure 43. Time-dependent ^{1}H NMR spectra (600 MHz, **288** K) of the mixture of $[Ag_5\mathbf{1}_2]\cdot[OTf]_5$ and **Ad-MeOH**$\subset[Ag_5\mathbf{1}_2]\cdot[OTf]_5$ in $CDCl_2CDCl_2$.

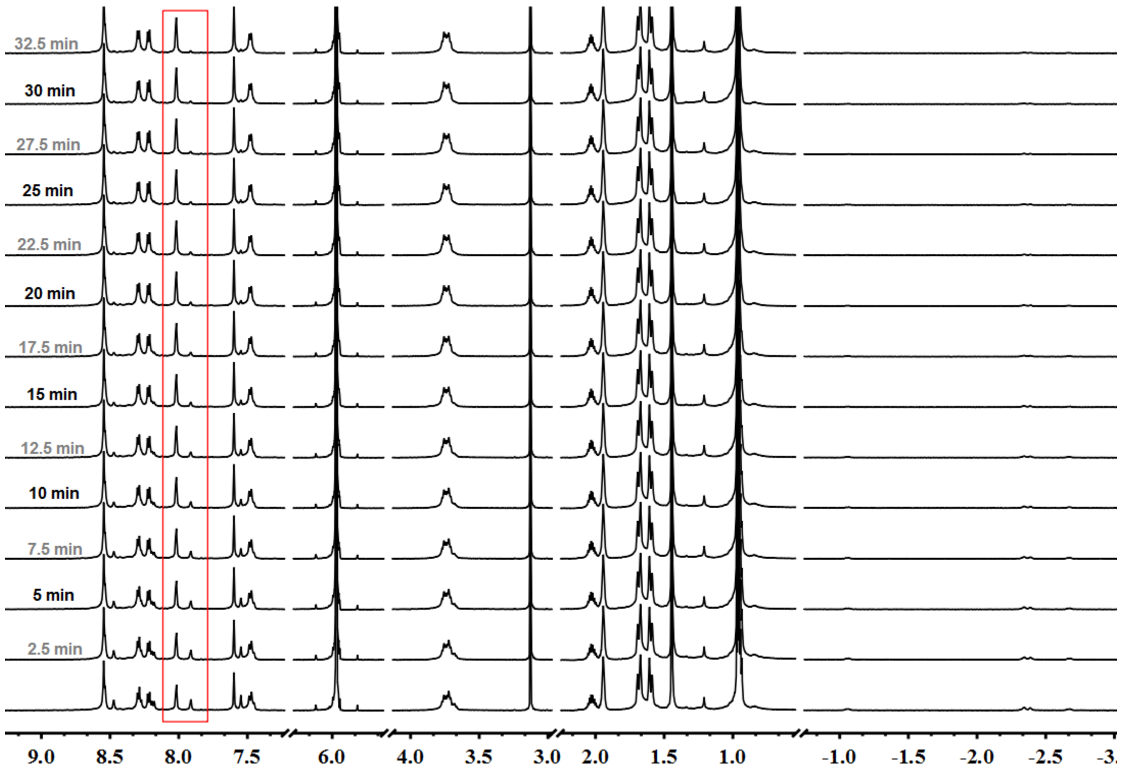

Supplementary Figure 44. Time-dependent ^{1}H NMR spectra (600 MHz, **292** K) of the mixture of $[Ag_5\mathbf{1}_2]\cdot[OTf]_5$ and **Ad-MeOH**$\subset[Ag_5\mathbf{1}_2]\cdot[OTf]_5$ in $CDCl_2CDCl_2$.

Supplementary Table 9. The calculations for **Ad-MeOH** release kinetics in $CDCl_2CDCl_2$ at different temperatures: the concentration of the capsule with guest encapsulated ($c_{capsule}$), concentration of the free cage produced in the decay ($c_{cage,prd}$), and ln($c_{capsule}$) at different time. The data were derived from the time-dependent ^{1}H NMR spectra (Supplementary Figures 41–44).

279K	t (s)	0	528	1054	1574	2094	2612	3138	3664
	$c_{cage,prd}$	0.543	0.552	0.562	0.568	0.575	0.581	0.588	0.592
	$c_{capsule}$	0.457	0.448	0.438	0.432	0.425	0.419	0.412	0.408
	ln($c_{capsule}$)	-0.784	-0.804	-0.825	-0.840	-0.855	-0.871	-0.887	-0.896
284K	t (s)	0	536	1068	1603	2139	2673	3207	3739
	$c_{cage,prd}$	0.654	0.671	0.690	0.704	0.714	0.725	0.741	0.752
	$c_{capsule}$	0.346	0.329	0.310	0.296	0.286	0.275	0.259	0.248
	ln($c_{capsule}$)	-1.060	-1.112	-1.170	-1.218	-1.253	-1.290	-1.350	-1.394
288K	t (s)	0	540	1080	1620	2160	2700	3240	3780
	$c_{cage,prd}$	0.680	0.704	0.735	0.763	0.787	0.813	0.833	0.855
	$c_{capsule}$	0.320	0.296	0.265	0.237	0.213	0.187	0.167	0.145
	ln($c_{capsule}$)	-1.140	-1.218	-1.329	-1.441	-1.548	-1.677	-1.792	-1.929
292K	t (s)	0	268	535	803	1070	1337	1604	1867
	$c_{cage,prd}$	0.694	0.741	0.775	0.806	0.833	0.862	0.877	0.901
	$c_{capsule}$	0.306	0.259	0.225	0.194	0.167	0.138	0.123	0.099
	ln($c_{capsule}$)	-1.186	-1.350	-1.493	-1.642	-1.792	-1.981	-2.097	-2.312

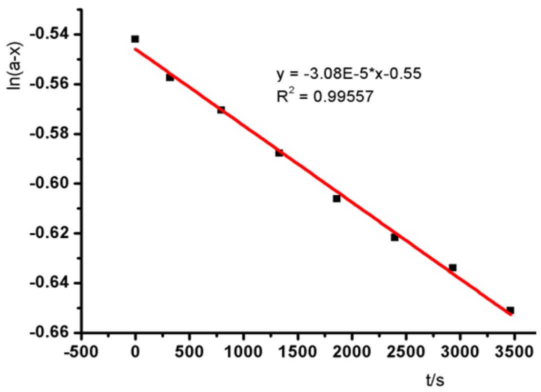

a

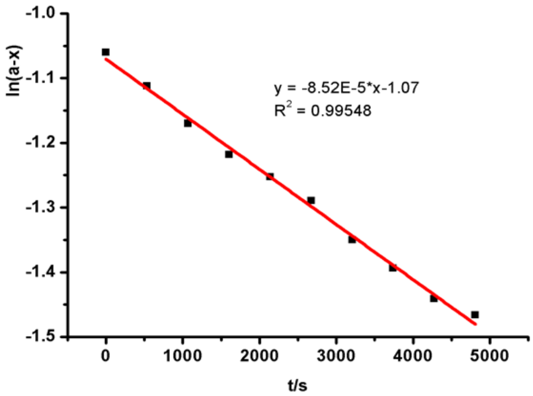

b

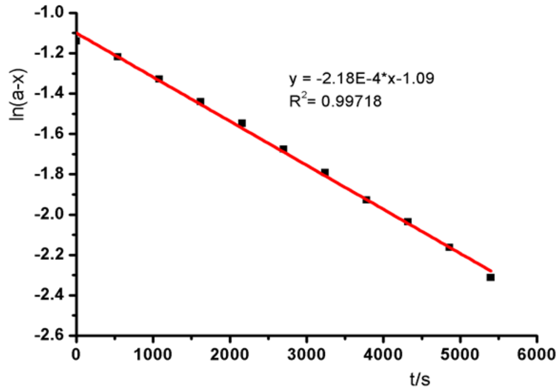

c

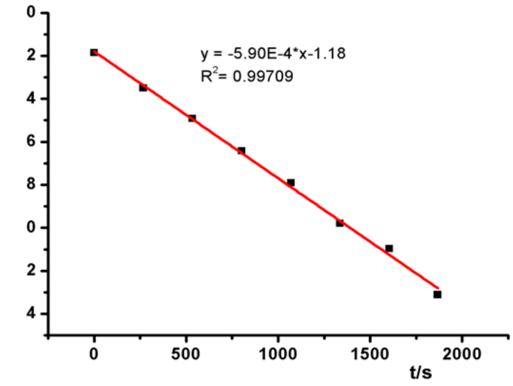

d

Supplementary Figure 45. Release kinetics for **Ad-MeOH** from $[Ag_5\mathbf{1}_2]\cdot[OTf]_5$ in $CDCl_2CDCl_2$ at different temperature: fits to the first order kinetics as measures of relative concentration of **Ad-MeOH**$\subset$ $[Ag_5\mathbf{1}_2]\cdot[OTf]_5$. (a) 279K; (b) 284 K; (c) 288 K; (d) 292 K.

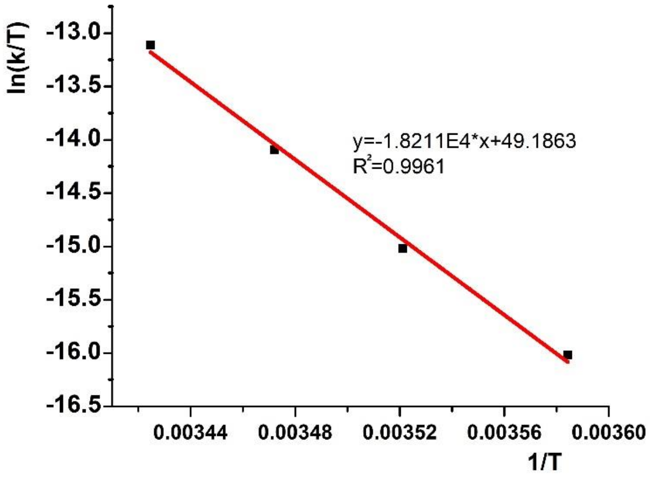

Supplementary Figure 46. Release kinetics for **Ad-MeOH** from $[Ag_5\mathbf{1}_2]\cdot[OTf]_5$ in $CDCl_2CDCl_2$: numerical fit to the Eyring equations. The first order rate constant (k) were obtained from Supplementary Figure 45.

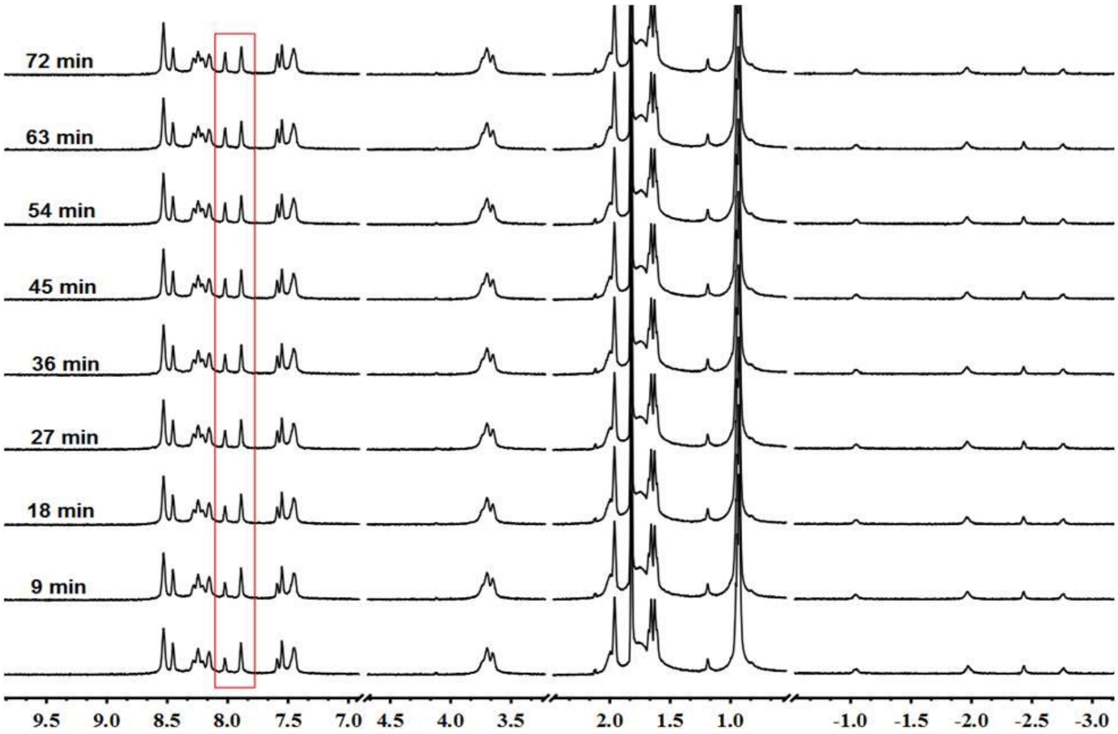

Supplementary Figure 47. Time-dependent ^{1}H NMR spectra (600 MHz, **274** K) of the mixture of $[Ag_5\mathbf{1}_2]\cdot[OTf]_5$ and **Ad-COOH**$\subset[Ag_5\mathbf{1}_2]\cdot[OTf]_5$ in $CDCl_2CDCl_2$.

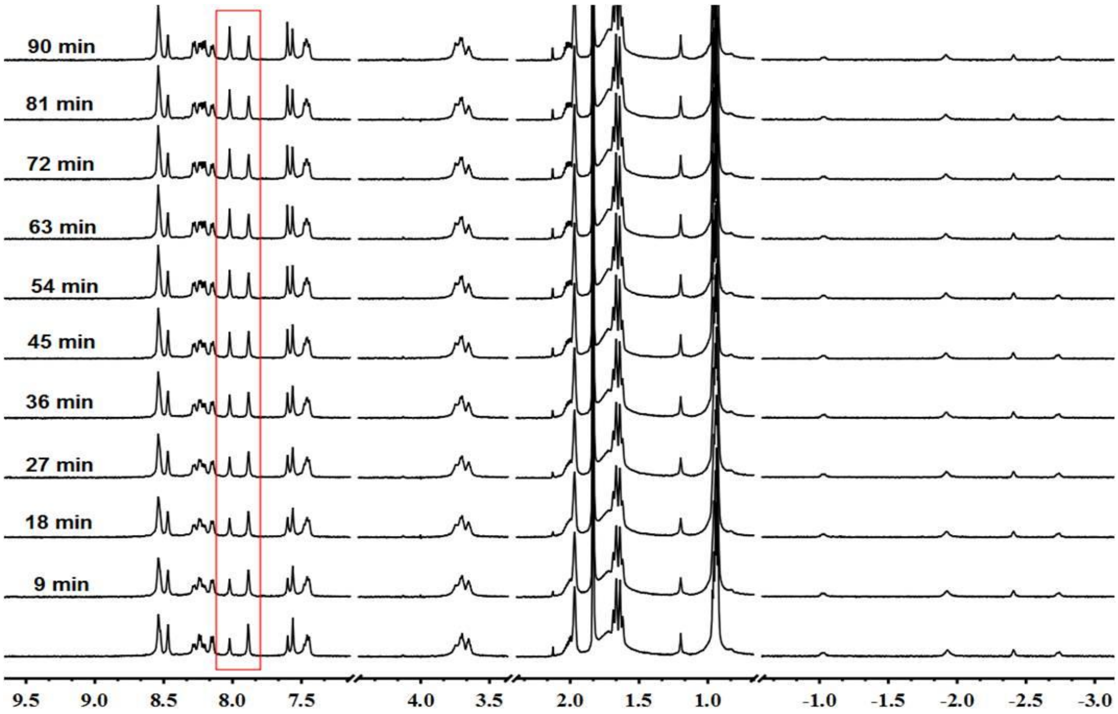

Supplementary Figure 48. Time-dependent ^{1}H NMR spectra (600 MHz, **279** K) of the mixture of $[Ag_5\mathbf{1}_2]\cdot[OTf]_5$ and **Ad-COOH**$\subset[Ag_5\mathbf{1}_2]\cdot[OTf]_5$ in $CDCl_2CDCl_2$.

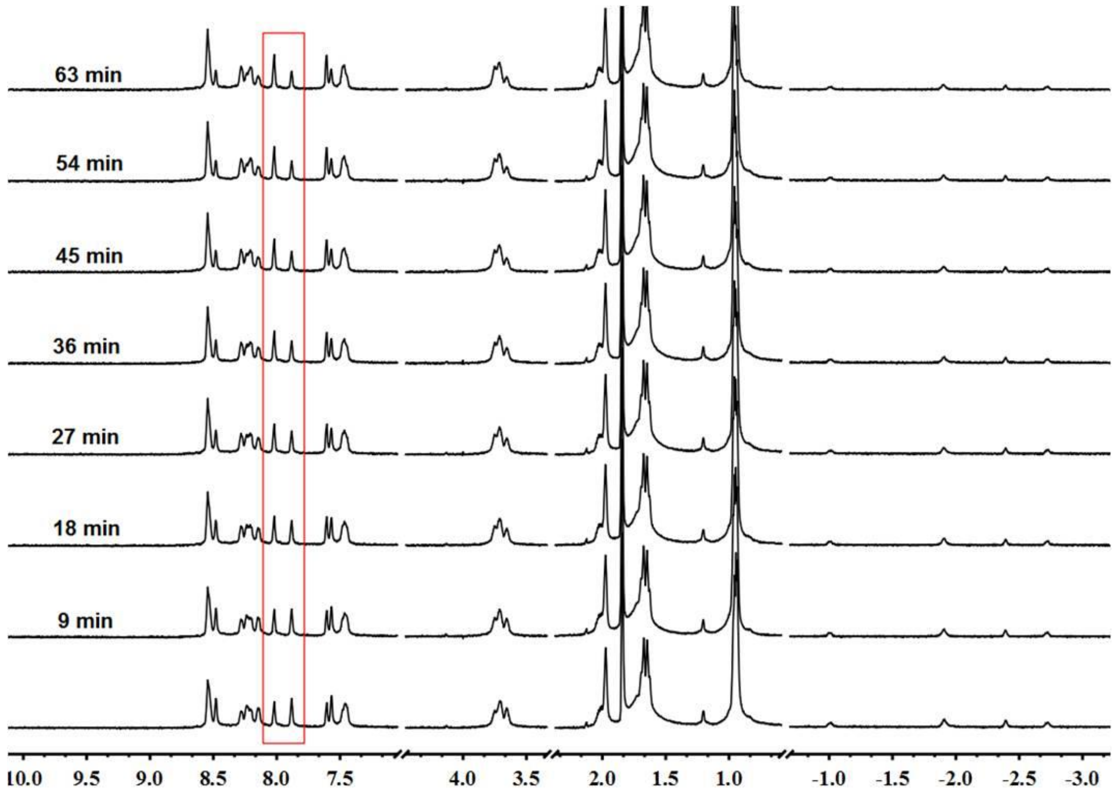

Supplementary Figure 49. Time-dependent ^{1}H NMR spectra (600 MHz, **284** K) of the mixture of [Ag_5**1**$_2$]·[OTf]$_5$ and **Ad-COOH**⊂[Ag_5**1**$_2$]·[OTf]$_5$ in $CDCl_2CDCl_2$.

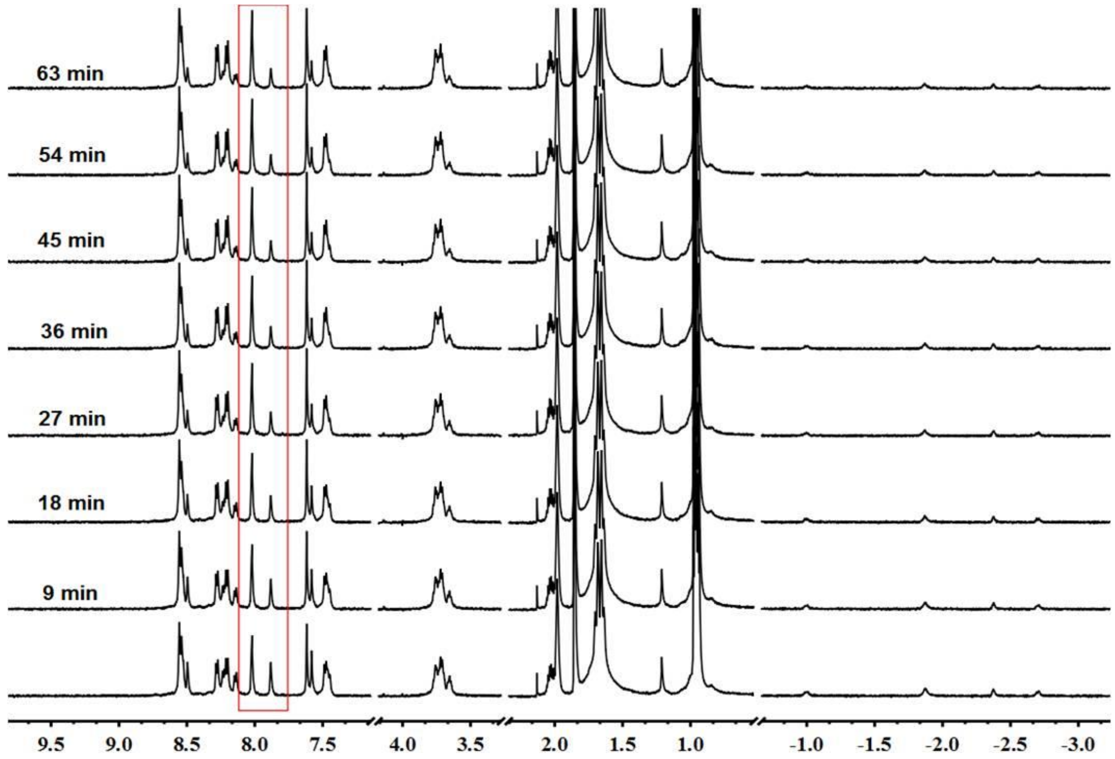

Supplementary Figure 50. Time-dependent ^{1}H NMR spectra (600 MHz, **288** K) of the mixture of [Ag_5**1**$_2$]·[OTf]$_5$ and **Ad-COOH**⊂[Ag_5**1**$_2$]·[OTf]$_5$ in $CDCl_2CDCl_2$.

Supplementary Table 10. The calculations for **Ad-COOH** release kinetics in $CDCl_2CDCl_2$ at different temperatures: the concentration of the capsule with guest encapsulated ($c_{capsule}$), concentration of the free cage produced in the decay ($c_{cage,prd}$), and ln($c_{capsule}$) at different time. The data were derived from the time-dependent ^{1}H NMR spectra (Supplementary Figures 47–50).

274 K	t (s)	0	804	1338	1874	2408	2976	3510	4044	4578
	$c_{cage,prd}$	0.298	0.328	0.341	0.355	0.360	0.380	0.394	0.407	0.410
	$c_{capsule}$	0.702	0.672	0.659	0.645	0.640	0.620	0.606	0.593	0.590
	ln($c_{capsule}$)	-0.353	-0.397	-0.417	-0.438	-0.446	-0.478	-0.500	-0.522	-0.527
279 K	t (s)	0	1070	1604	2138	2672	3470	4002	4536	5072
	$c_{cage,prd}$	0.663	0.615	0.588	0.565	0.535	0.515	0.490	0.479	0.454
	$c_{capsule}$	0.337	0.385	0.412	0.435	0.465	0.485	0.510	0.521	0.546
	ln($c_{capsule}$)	-0.411	-0.486	-0.530	-0.571	-0.626	-0.669	-0.714	-0.736	-0.791
284 K	t (s)	0	800	1314	1848	2382	2916	3448	-	-
	$c_{cage,prd}$	0.442	0.485	0.521	0.546	0.568	0.599	0.641	-	-
	$c_{capsule}$	0.558	0.515	0.479	0.454	0.432	0.401	0.359	-	-
	ln($c_{capsule}$)	-0.584	-0.664	-0.736	-0.791	-0.840	-0.913	-1.025	-	-
288 K	t (s)	0	508	1014	1520	2028	2534	3040	3546	4054
	$c_{cage,prd}$	0.410	0.452	0.493	0.518	0.562	0.599	0.633	0.667	0.699
	$c_{capsule}$	0.590	0.548	0.507	0.482	0.438	0.401	0.367	0.333	0.301
	ln($c_{capsule}$)	-0.425	-0.602	-0.678	-0.730	-0.825	-0.913	-1.002	-1.099	-0.202

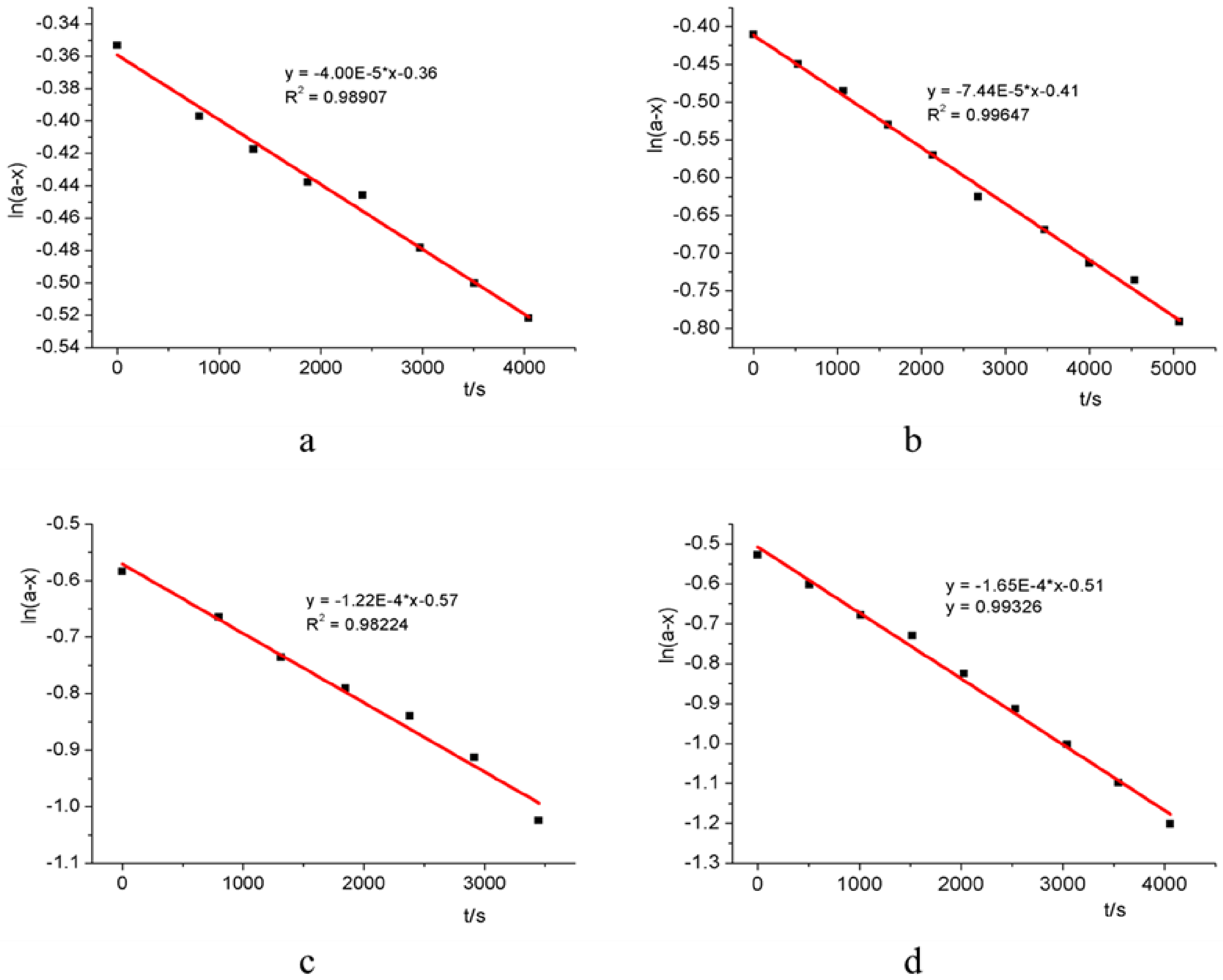

Supplementary Figure 51. Release kinetics for **Ad-COOH** from [Ag$_5$**1**$_2$]·[OTf]$_5$ in $CDCl_2CDCl_2$ at different temperature: fits to the first order kinetics as measures of relative concentration of **Ad-COOH**⊂ [Ag$_5$**1**$_2$]·[OTf]$_5$. (a) 274K; (b) 279 K; (c) 284 K; (d) 288 K.

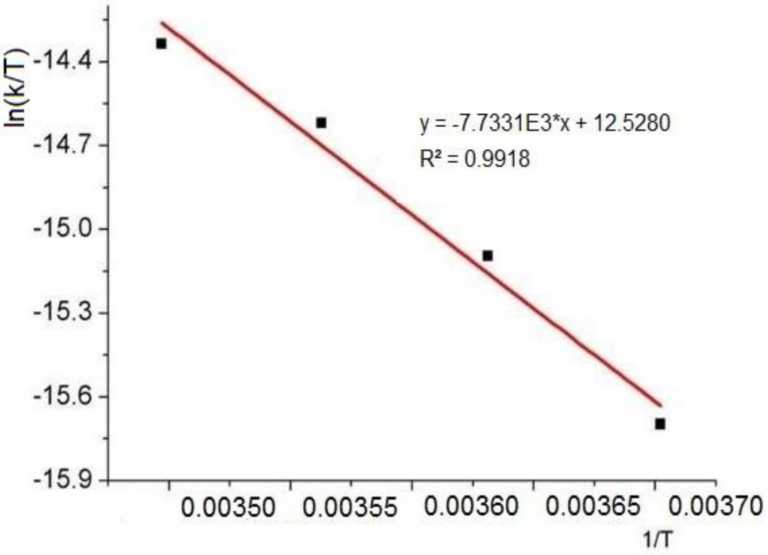

Supplementary Figure 52. Release kinetics for **Ad-COOH** from [Ag$_5$**1**$_2$]·[OTf]$_5$ in $CDCl_2CDCl_2$: numerical fit to the Eyring equations. The first order rate constant (*k*) were obtained from Supplementary Figure 51.

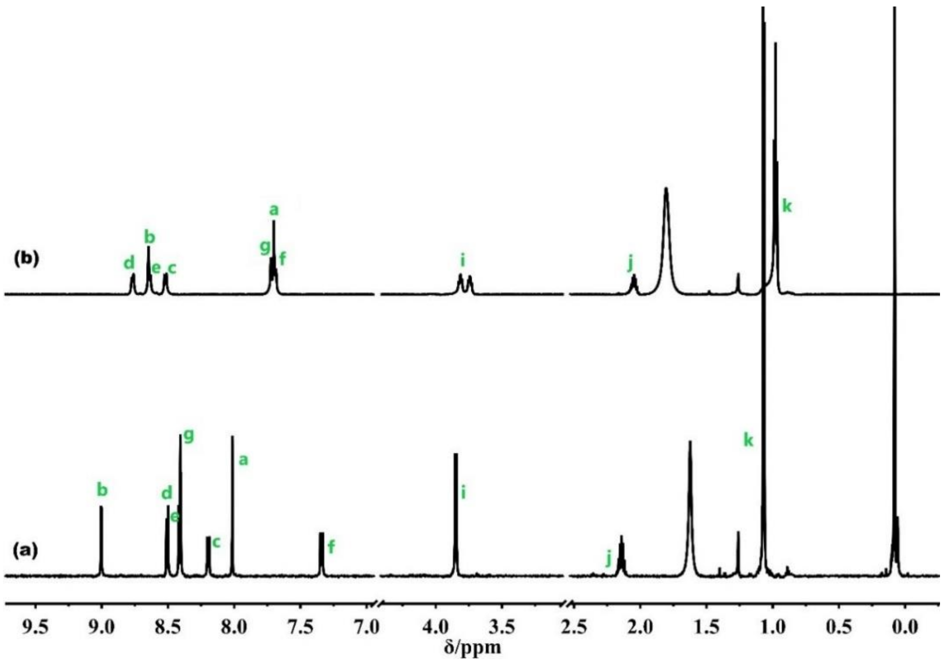

Supplementary Figure 53. ^{1}H NMR (600 MHz, 298 K, $CDCl_2CDCl_2$) spectrum of (a) Ligand **1**; (b) $[Hg_5\mathbf{1}_2]\cdot[OTf]_{10}$ (1 mM).

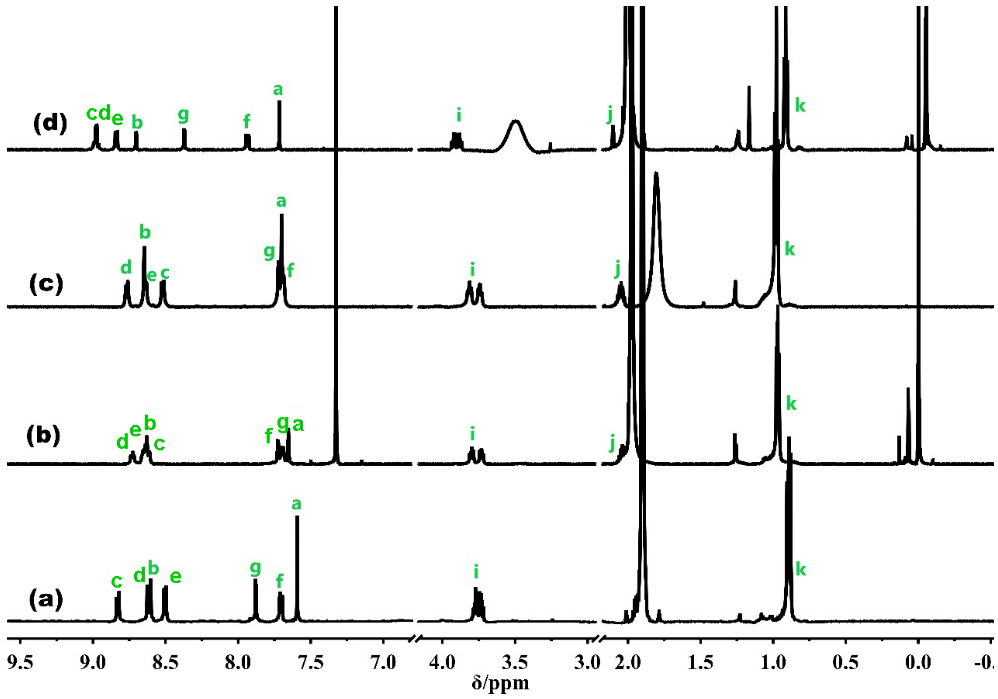

Supplementary Figure 54. ^{1}H NMR (600 MHz, 298 K) of $[Hg_5\mathbf{1}_2]\cdot[OTf]_{10}$ (1 mM) in (a) CD_3CN; (b) 95:5 (v/v) $CDCl_3/CD_3CN$; (c) $CDCl_2CDCl_2$; (d) acetone-d_6.

Supplementary Method 9. Studies on Encapsulation of **Ad** and its Derivatives in **Hg_5L_2** in Different Solvent Systems

Three kinds of guest molecules, including adamantane (**Ad**), 1-admethanol (**Ad-MeOH**), and 1-adamantanecarboxylic acid (**Ad-COOH**) were investigated in this study.

Encapsulation of the studied guests with **Hg_5L_2** is kinetically harder or much harder than that in the cases of **Ag_5L_2**. We thus tried different procedures to achieve it.

Supplementary Method 10. Guest Encapsulation in **Hg_5L_2** in CD_3CN and Acetone-*d_6*

For **Hg_5L_2**, the behavior of the guest encapsulation in CD_3CN and in acetone-*d_6* is pretty similar to each other. Two procedures (**Procedure A** and **B**, *vide infra*) were utilized to prepare the cage-guest complexes as well as to verify the encapsulation process, using CD_3CN or acetone-*d_6* as the solvent. These two procedures resulted in the same outcomes.

Procedure A (From Cages to Cage-guest Complexes): Totally 6 ~ 8 cage samples were prepared by addition of 2.5 equivalents of a concentrated solution of mercury triflate ($Hg(OTf)_2$, 200 mM) in CD_3CN (or acetone-*d_6*) to ligand **1** (2 mM, 0.5 mL) in CD_3CN (or acetone-*d_6*) to give 1 mM cage solutions. To each of these solutions, different equivalents of guest (50 mM in CD_3CN or acetone-*d_6*) was added, then the sample was sonicated for 30 minutes in an ultrasonic instrument. A series of ^1H NMR spectra of the obtained mixtures were recorded by a 600 MHz (^1H) spectrometer at 298 K.

Procedure B (One-Pot Construction): To a series of samples of ligand **1** (2 mM, 0.5 mL) and different equivalents of guest in CD_3CN (or acetone-*d_6*), 2.5 equivalents of $Hg(OTf)_2$ were added. The obtained mixtures were sonicated at ambient temperature for 30 minutes, then corresponding ^1H NMR spectra were recorded by a 600 MHz (^1H) spectrometer at 298 K.

Examples of the obtained ^1H NMR spectra are shown in Supplementary Figures 55−60. The ^1H NMR signals of the complexes were assigned based on the ^1H−^1H COSY, ^1H−^{13}C HSQC, ^1H−^{13}C HMBC and ^1H−^1H NOESY spectra of **Ad**-encapsulated capsules (see Supplementary Figures 87−90).

Examples of the ^{1}H NMR spectra of guest⊂**Hg$_5$L$_2$** prepared via **Procedure A** or **B**:

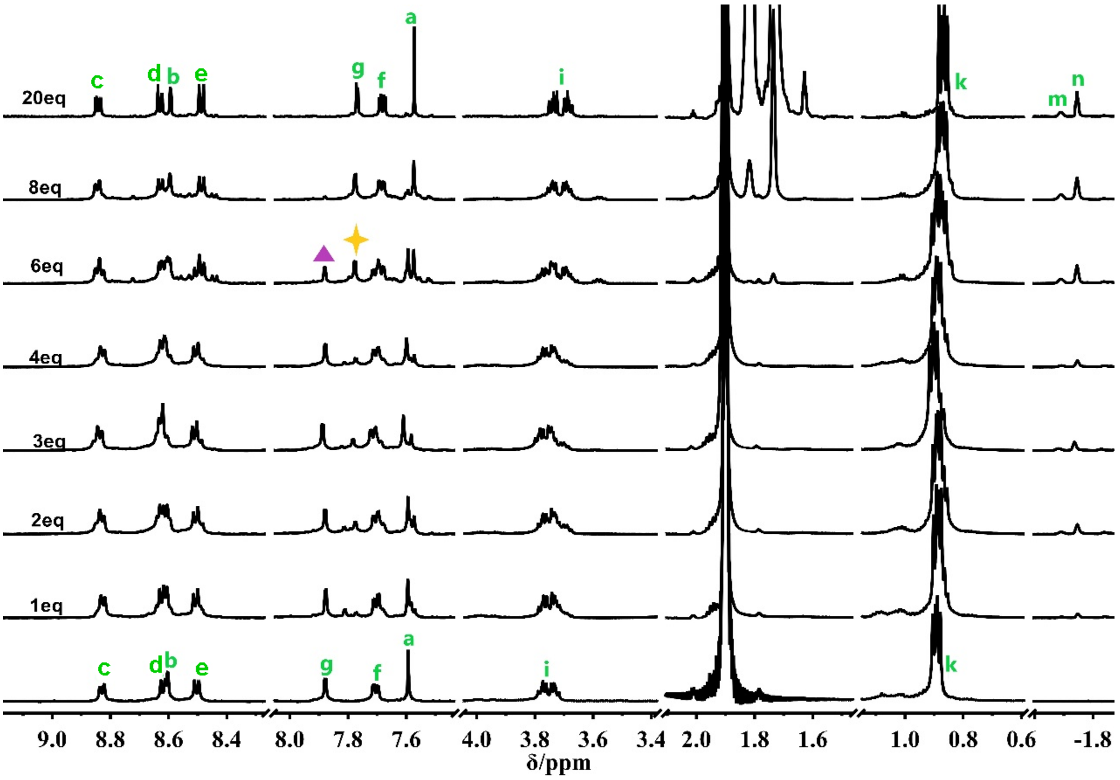

Supplementary Figure 55. Changes of ^{1}H NMR spectra (600 MHz, 298 K) of [Hg$_5$**1**$_2$]·[OTf]$_{10}$ (1 mM) in CD_3CN with different equivalents of **Ad**. ▲ =[Hg$_5$**1**$_2$]·[OTf]$_{10}$, ✦ = **Ad**⊂[Hg$_5$**1**$_2$]·[OTf]$_{10}$.

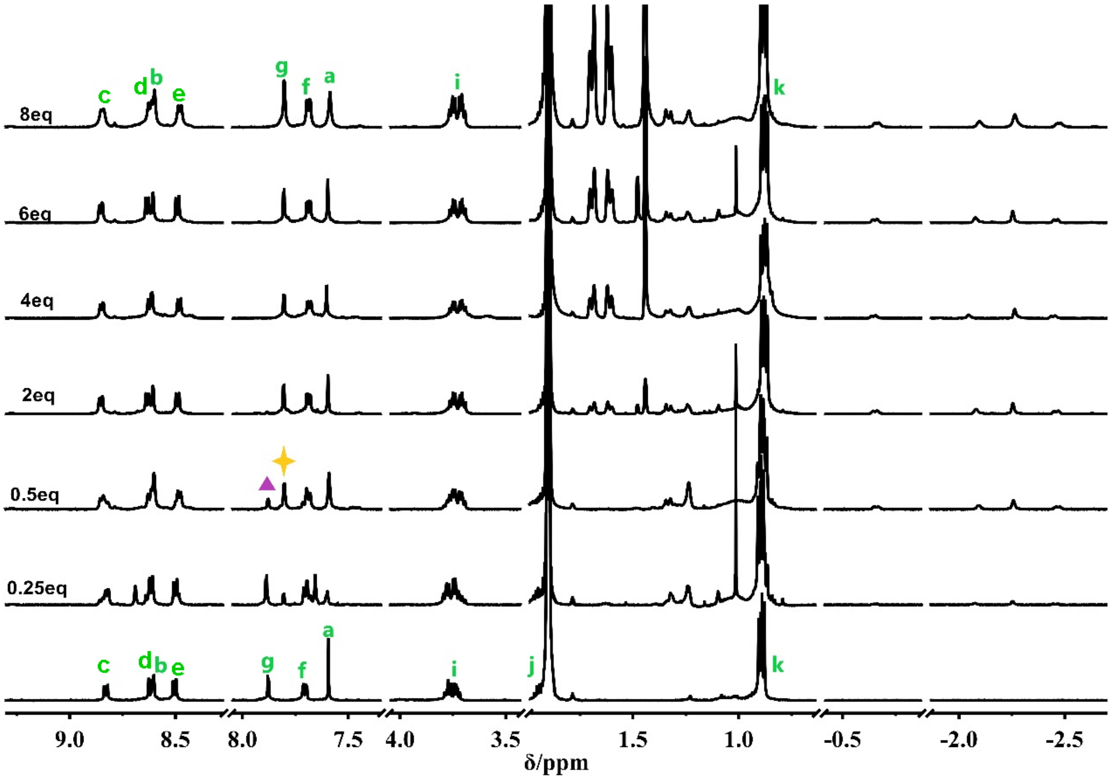

Supplementary Figure 56. Changes of ^{1}H NMR spectra (600 MHz, 298 K) of [Hg$_5$**1**$_2$]·[OTf]$_{10}$ (1 mM) in CD_3CN with different equivalents of **Ad-MeOH**. ▲ =[Hg$_5$**1**$_2$]·[OTf]$_{10}$, ✦ = **Ad-MeOH** ⊂[Hg$_5$**1**$_2$]·[OTf]$_{10}$.

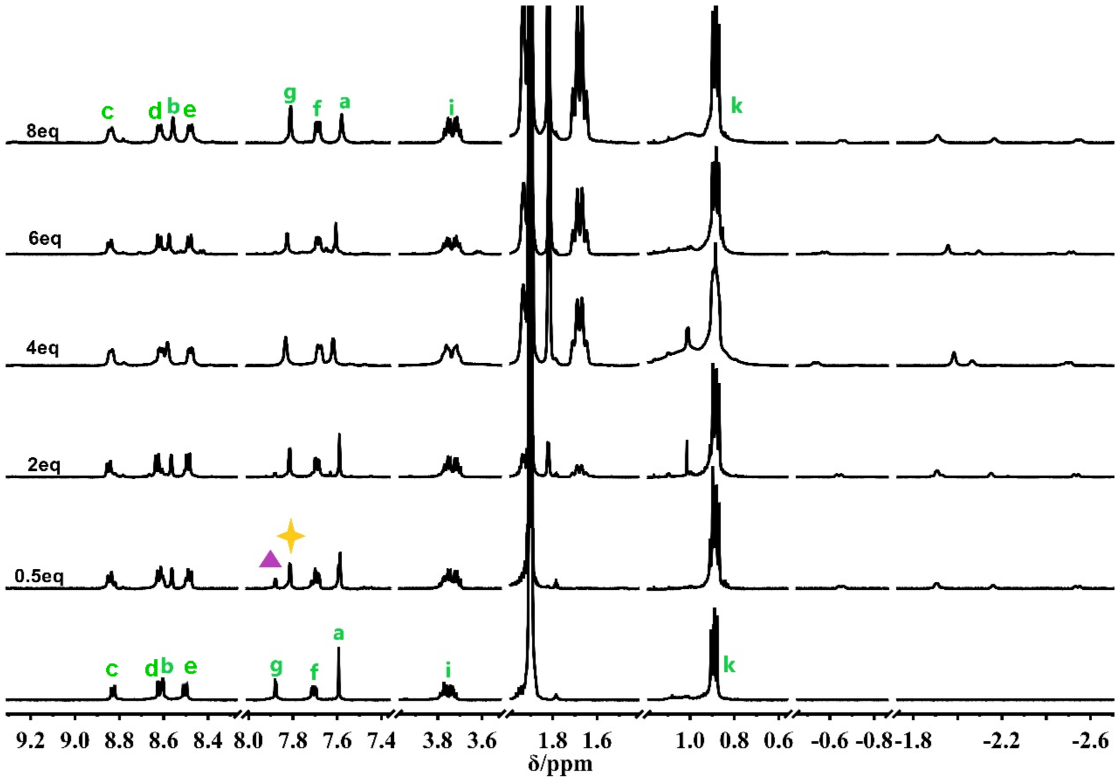

Supplementary Figure 57. Changes of ^{1}H NMR spectra (600 MHz, 298 K) of $[Hg_5\mathbf{1}_2]\cdot[OTf]_{10}$ (1 mM) in CD_3CN with different equivalents of **Ad-COOH**. ▲ $=[Hg_5\mathbf{1}_2]\cdot[OTf]_{10}$, ✦ = **Ad-COOH**$\subset[Hg_5\mathbf{1}_2]\cdot[OTf]_{10}$.

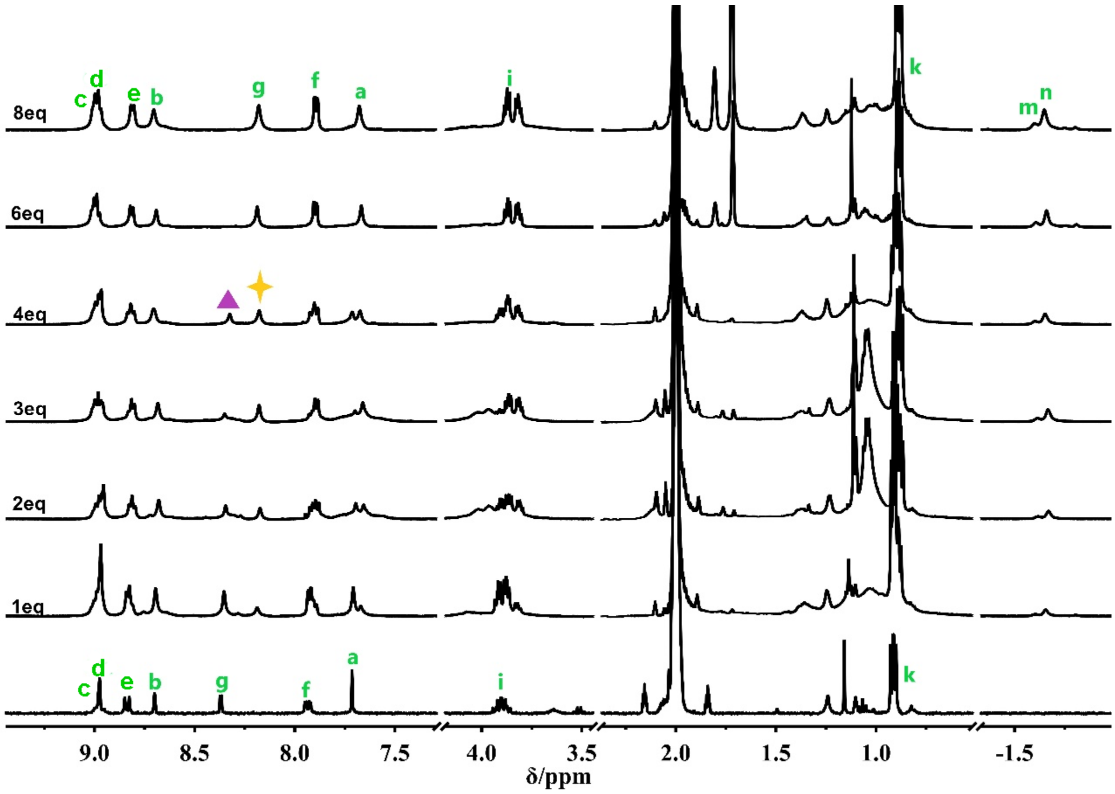

Supplementary Figure 58. Changes of ^{1}H NMR spectra (600 MHz, 298 K) of $[Hg_5\mathbf{1}_2]\cdot[OTf]_{10}$ (1 mM) in acetone-d_6 with different equivalents of **Ad**. ▲ $=[Hg_5\mathbf{1}_2]\cdot[OTf]_{10}$, ✦ = **Ad**$\subset[Hg_5\mathbf{1}_2]\cdot[OTf]_{10}$.

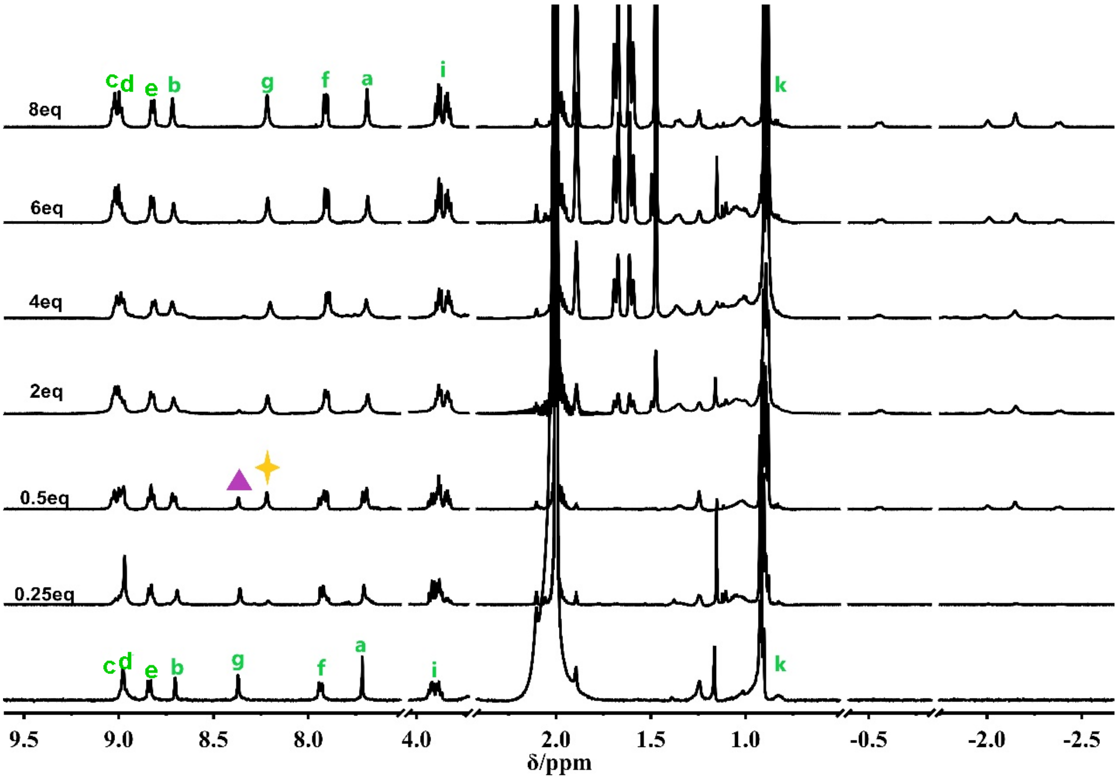

Supplementary Figure 59. Changes of ^{1}H NMR spectra (600 MHz, 298 K) of [Hg$_5$**1**$_2$]·[OTf]$_{10}$ (1 mM) in acetone-d_6 with different equivalents of **Ad-MeOH**. ▲ =[Hg$_5$**1**$_2$]·[OTf]$_{10}$, ✦ = **Ad-MeOH**⊂[Hg$_5$**1**$_2$]·[OTf]$_{10}$.

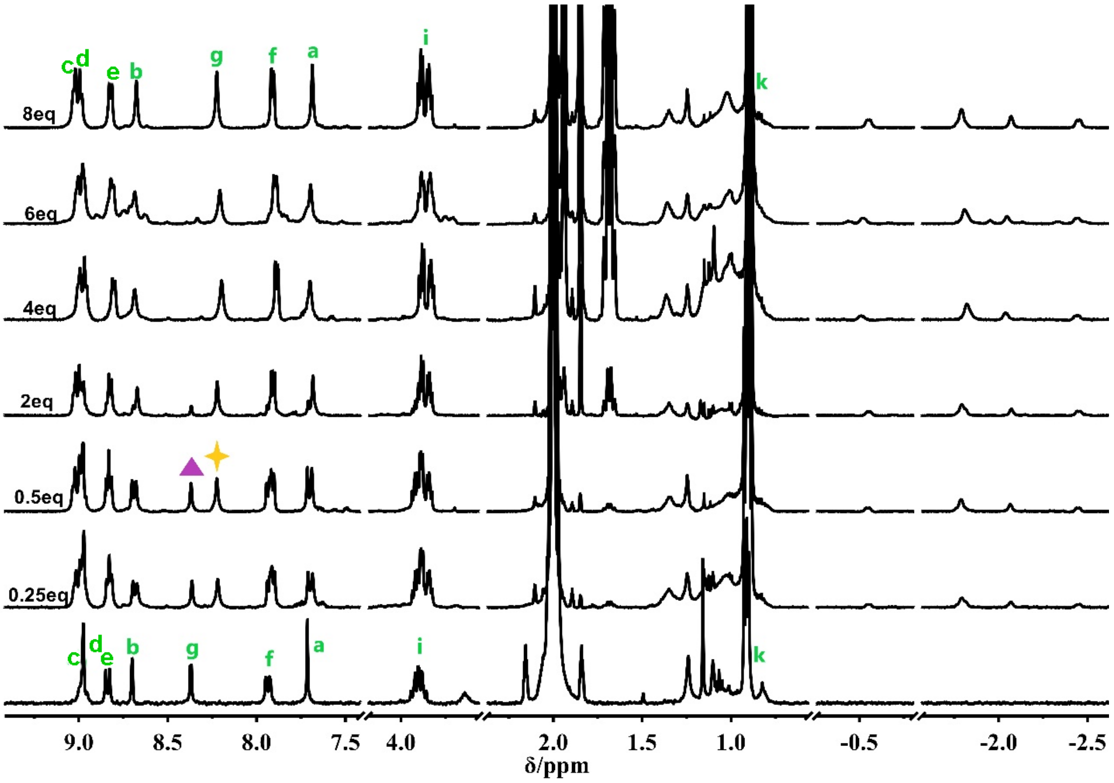

Supplementary Figure 60. Changes of ^{1}H NMR spectra (600 MHz, 298 K) of [Hg$_5$**1**$_2$]·[OTf]$_{10}$ (1 mM) in acetone-d_6 with different equivalents of **Ad-COOH**. ▲ =[Hg$_5$**1**$_2$]·[OTf]$_{10}$, ✦ = **Ad-COOH**⊂[Hg$_5$**1**$_2$]·[OTf]$_{10}$.

Supplementary Method 11. Guest Encapsulation in $\mathbf{Hg_5L_2}$ in 95:5 (v/v) $CDCl_3/CD_3CN$

Two procedures (**Procedure C** and **D**) were tried to achieve the guest encapsulation in 95:5 (v/v) $CDCl_3/CD_3CN$.

Procedure C (From Cages to Cage-guest Complexes): This procedure was tired with only **Ad** as the guest. To a sample of ligand **1** (2 mM, 0.5 mL) in 95:5 (v/v) $CDCl_3/CD_3CN$, mercury triflate ($Hg(OTf)_2$, 200 mM) in 95:5 (v/v) $CDCl_3/CD_3CN$ was added, giving a 1 mM $\mathbf{Hg_5L_2}$ cage solution. To the solution, 200 equivalents of **Ad** (50 mM, 95:5 (v/v) $CDCl_3/CD_3CN$) was added. The mixture was heated at 50 °C. The encapsulation process was monitored by ^{1}H NMR spectroscopy as shown in Supplementary Figure 61, which showed that saturation of the complexation could be achieved in 2~3 days.

Procedure D (Solvent-replacement Method): This procedure involves the preparation of the cage-guest complexes in CD_3CN or acetone-d_6 in advance, followed the replacement of CD_3CN or acetone-d_6 with 95:5 (v/v) $CDCl_3/CD_3CN$.

To a series of samples of ligand **1** (2 mM, 0.5 mL) in CD_3CN, 2.5 equivalents of $Hg(OTf)_2$ (200 mM, CD_3CN) was added, respectively. To each of the obtained solution, different equivalents of the guest (50 mM) was added. The mixtures were sonicated for 30 minutes at 298 K, then evacuated under reduced pressure. The residues were re-dissolved in 95:5 (v/v) $CDCl_3/CD_3CN$, and the corresponding ^{1}H NMR spectra was recorded at 298 K. The obtained spectra are shown in Supplementary Figures 62−64.

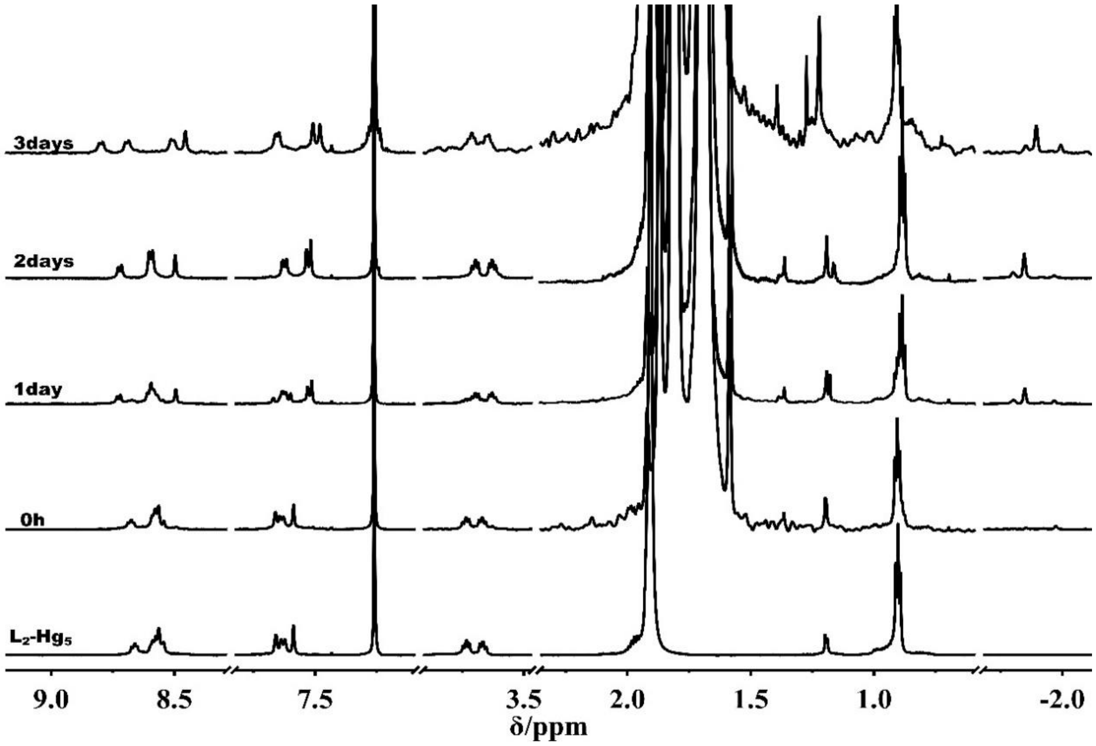

Supplementary Figure 61. Time-dependent ^{1}H NMR spectra (600 MHz, 298 K, 5:95 (v/v) $CD_3CN/CDCl_3$) of the mixture of $[Hg_5\mathbf{1}_2]\cdot[OTf]_{10}$ (1 mM) and **Ad** (200 mM) upon heating at 323 K.

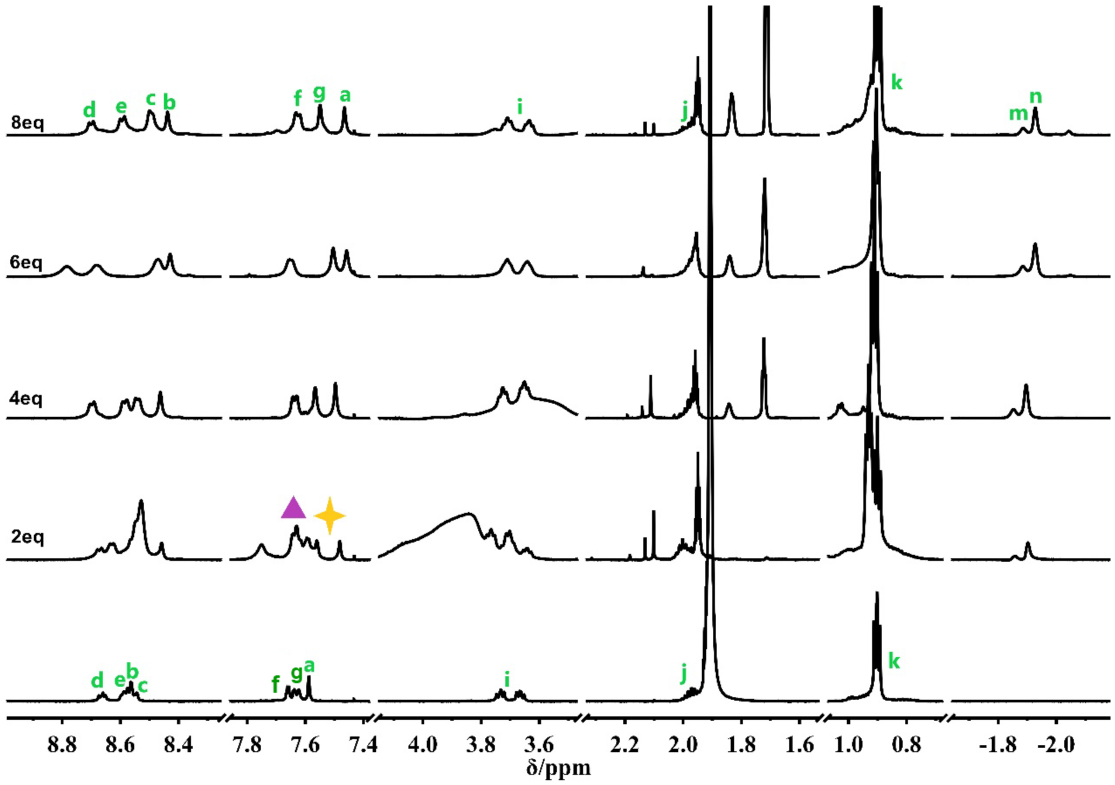

Supplementary Figure 62. Changes of ^{1}H NMR spectra (600 MHz, 298 K) of $[Hg_5\mathbf{1}_2]\cdot[OTf]_{10}$ (1 mM) in 95:5 (v/v) $CDCl_3/CD_3CN$ with different equivalents of **Ad**. ▲=$[Hg_5\mathbf{1}_2]\cdot[OTf]_{10}$, ✦= **Ad**$\subset[Hg_5\mathbf{1}_2]\cdot[OTf]_{10}$. Samples were prepared via **Procedure D**.

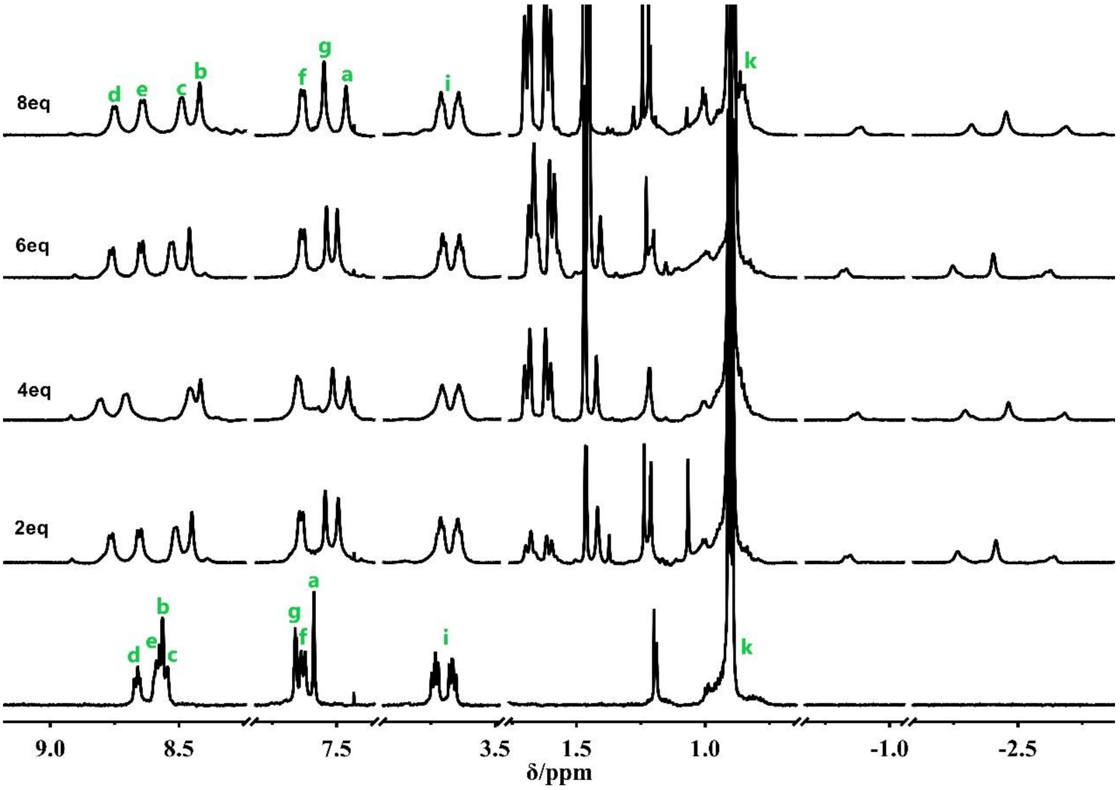

Supplementary Figure 63. Changes of ^{1}H NMR spectra (600 MHz, 298 K) of $[Hg_5\mathbf{1}_2]\cdot[OTf]_{10}$ (1 mM) in 95:5 (v/v) $CDCl_3/CD_3CN$ with different equivalents of **Ad-MeOH**. Samples were prepared via **Procedure D**.

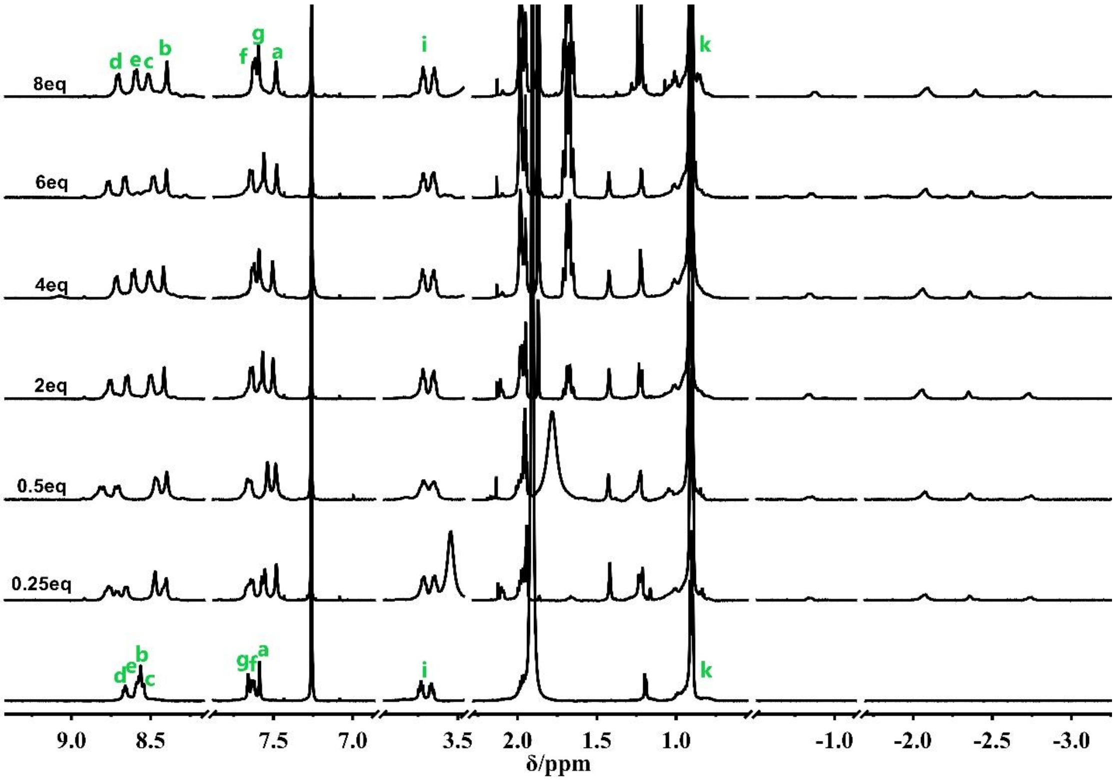

Supplementary Figure 64. Changes of ^{1}H NMR spectra (600 MHz, 298 K) of $[Hg_5\mathbf{1}_2]\cdot[OTf]_{10}$ (1 mM) in 95:5 (v/v) $CDCl_3/CD_3CN$ with different equivalents of **Ad-COOH**. Samples were prepared via **Procedure D**.

Supplementary Method 12. Guest Encapsulation in $\mathbf{Hg_5L_2}$ in $CDCl_2CDCl_2$

Three procedures (**Procedure E**, **F** and **D**) were tried to achieve the guest encapsulation in $CDCl_2CDCl_2$.

Procedure E (From Cages to Complexes): This procedure is as almost the same as that of **Procedure C** (Supplementary Method 11), except in which the $CDCl_2CDCl_2$ was used as the solvent and that the mixture of the cage and the guest was heated at 90 °C rather than 50 °C. **Ad** was tried as the only guest in this study. The encapsulation process was monitored by ^{1}H NMR spectroscopy. The obtained time-dependent ^{1}H NMR spectra are shown in Supplementary Figure 65, which showed that saturation of the complexation was achieved after the sample was heated for 6 days.

Procedure F (One-Pot Construction): This procedure is pretty close to that of **Procedure B** (Supplementary Method 10), except that $CDCl_2CDCl_2$ was used as the solvent and that, instead of being sonicated for 30 minutes, the mixture of the cage and the guest was heated at 90 °C. **Ad** was tired as the only guest in this study. The encapsulation process was monitored by ^{1}H NMR as shown in Supplementary Figure 66. The results showed that saturation of the complexation can be achieved in 3 days.

Supplementary Figure 67 shows this procedure produced the same outcome as that via **Procedure E**.

Procedure D (Solvent-replacement Method): Procedure D described in Supplementary Method 11 was used also to prepare the cage-guest complexes in $CDCl_2CDCl_2$. The obtained ^1H NMR spectra of the cage in the present of different amount of guest are shown in Supplementary Figures 68−70.

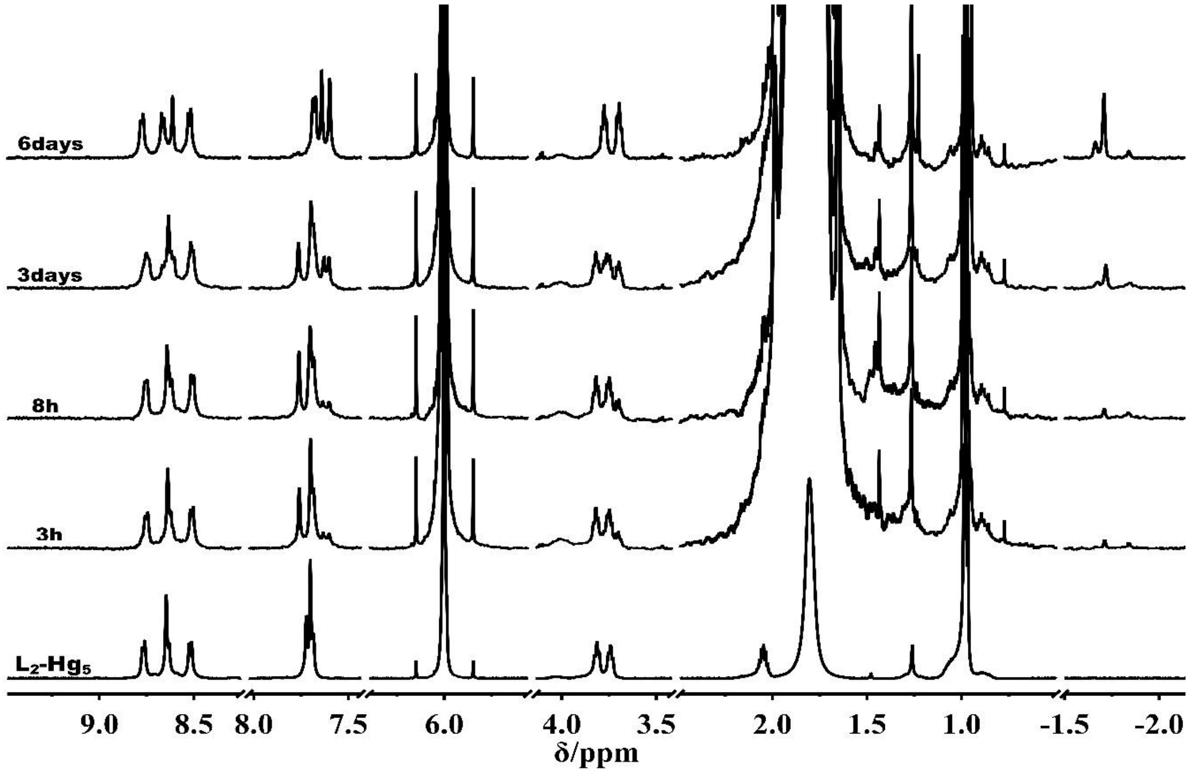

Supplementary Figure 65. Time-dependent ^1H NMR spectra (600 MHz, 298 K, $CDCl_2CDCl_2$) of the mixture of $[Hg_5\mathbf{1}_2]\cdot[OTf]_{10}$ (1 mM) and **Ad** (200 mM) upon heating at 90 °C.

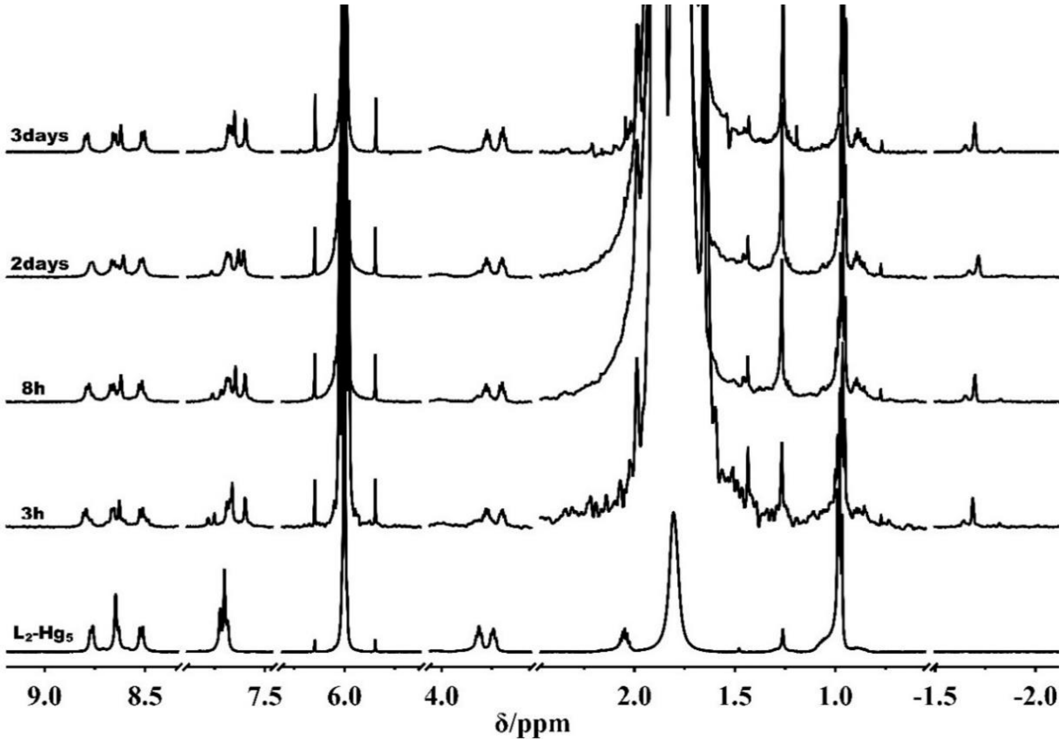

Supplementary Figure 66. Time-dependent ^1H NMR spectra (600 MHz, 298 K) of the mixture of ligand **1** (2 mM), $Hg(OTf)_2$ (5 mM) and **Ad** (200 mM) in $CDCl_2CDCl_2$ upon heating at 90 °C.

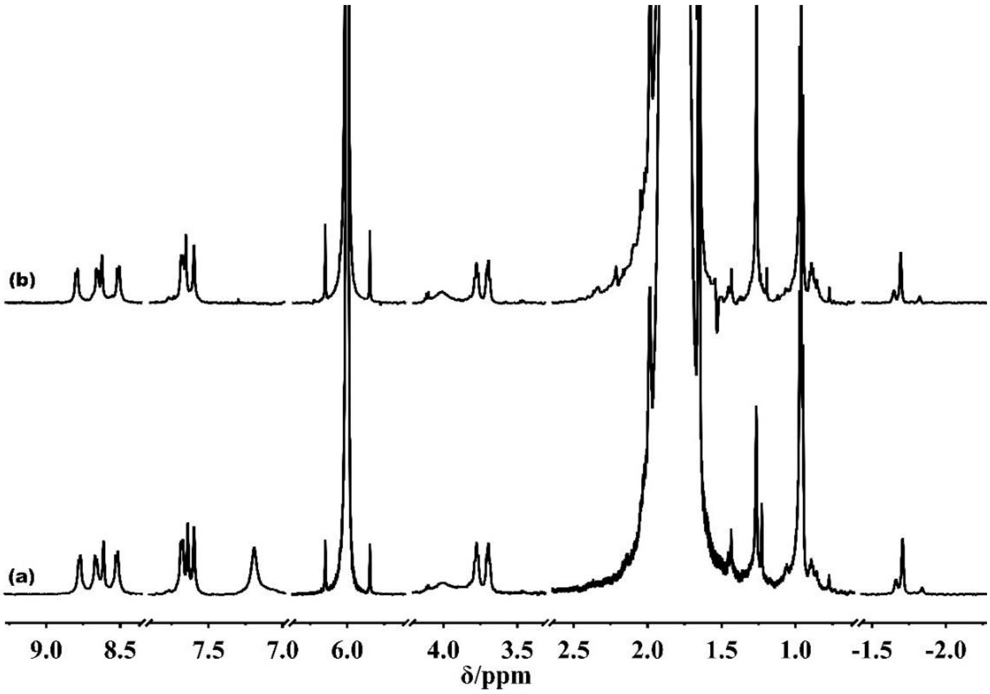

Supplementary Figure 67. Comparison of the ^{1}H NMR spectrum of **Ad**⊂[Hg_5**1**$_2$]·[OTf]$_{10}$ obtained from **Procedure E** and **Procedure F**, respectively, after the saturation of the complexation.

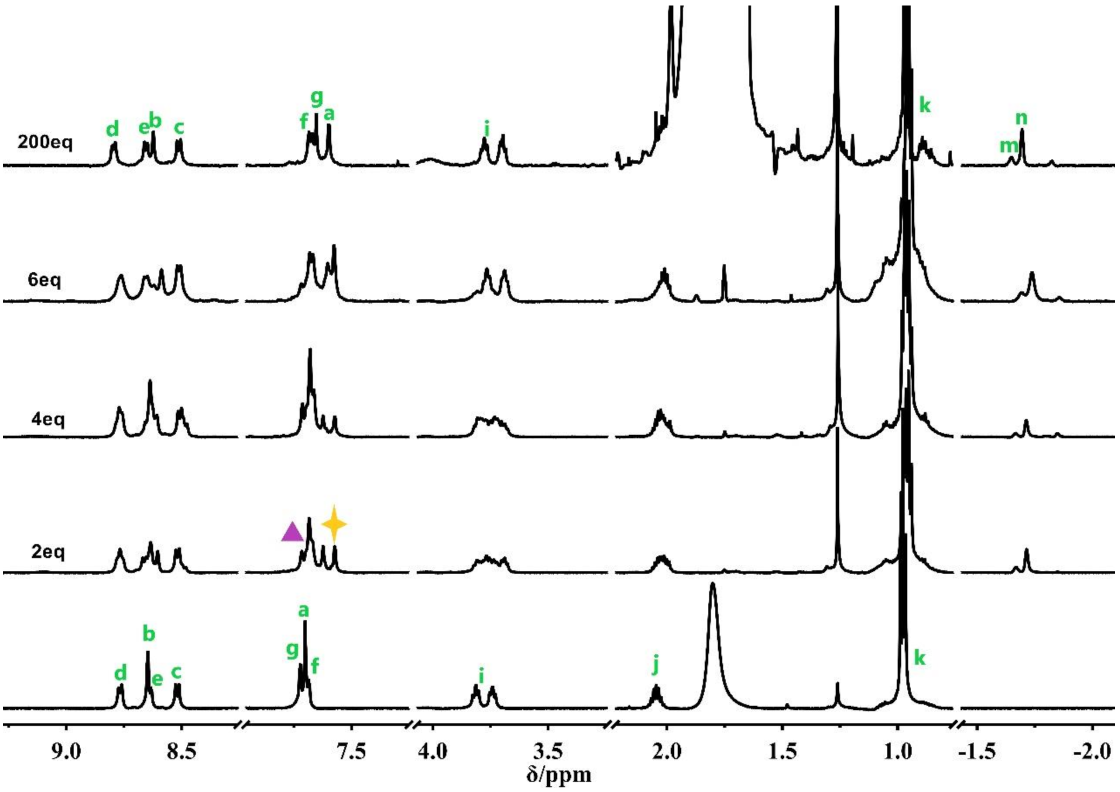

Supplementary Figure 68. Changes of ^{1}H NMR spectra (600 MHz, 298 K) of [Hg_5**1**$_2$]·[OTf]$_{10}$ (1 mM) in $CDCl_2CDCl_2$ with different equivalents of **Ad**. ▲ =[Hg_5**1**$_2$]·[OTf]$_{10}$, ✦ = **Ad**⊂[Hg_5**1**$_2$]·[OTf]$_{10}$. The cage-guest complexes were first prepared in acetone-*d*$_6$, followed an evaporation of the solvent and a redissolution in $CDCl_2CDCl_2$.

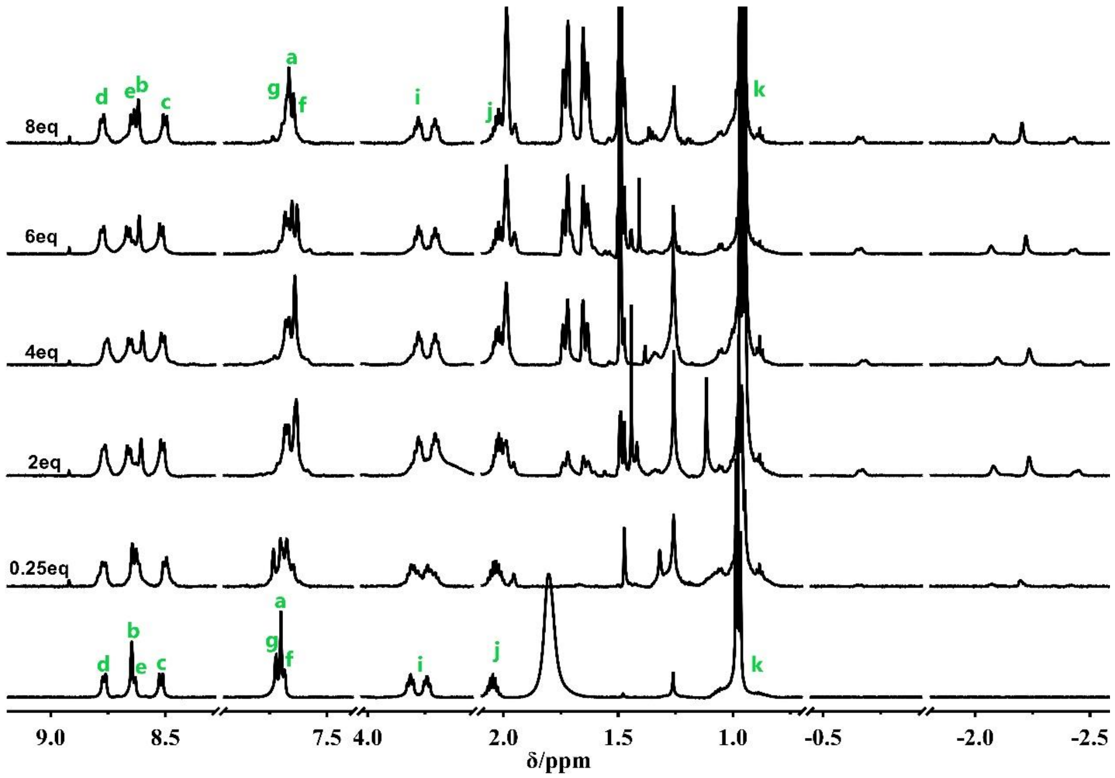

Supplementary Figure 69. Changes of ^{1}H NMR spectra (600 MHz, 298 K) of $[Hg_5\mathbf{1}_2]\cdot[OTf]_{10}$ (1 mM) in $CDCl_2CDCl_2$ with different equivalents of **Ad-MeOH**. The cage-guest complexes were first prepared in acetone-d_6, followed an evaporation of the solvent and a redissolution in $CDCl_2CDCl_2$.

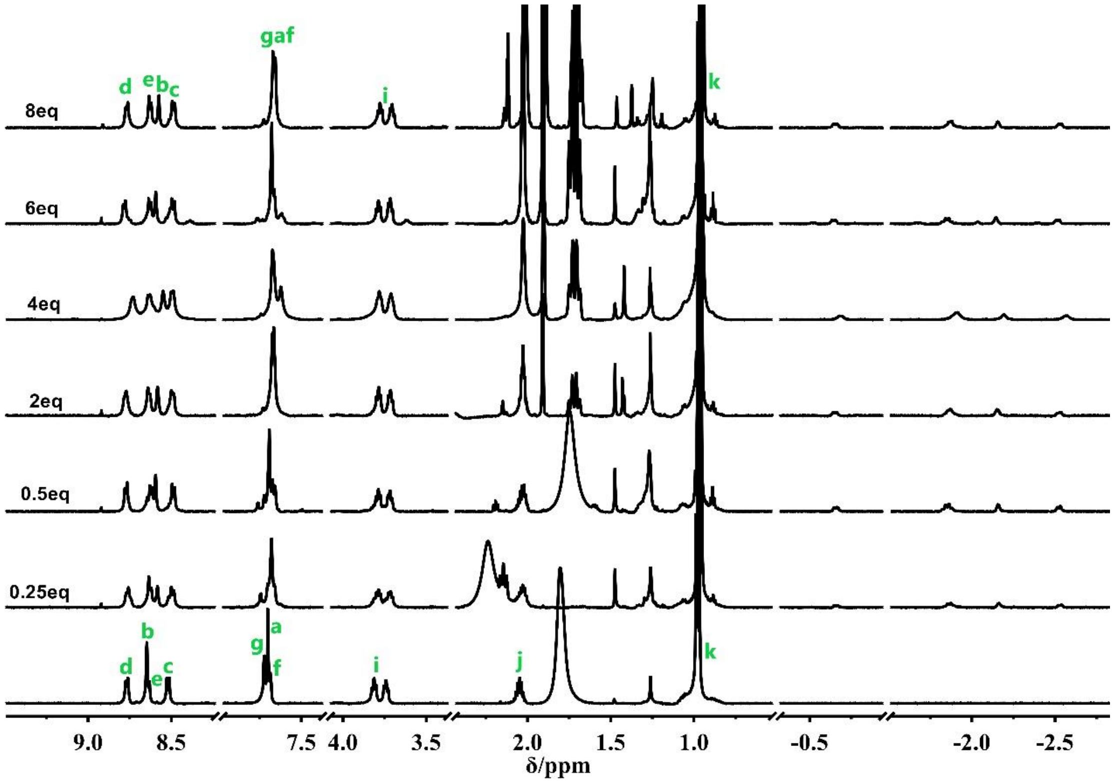

Supplementary Figure 70. Changes of ^{1}H NMR spectra (600 MHz, 298 K) of $[Hg_5\mathbf{1}_2]\cdot[OTf]_{10}$ (1 mM) in $CDCl_2CDCl_2$ with different equivalents of **Ad-COOH**. The cage-guest complexes were first prepared in acetone-d_6, followed an evaporation of the solvent and a redissolution in $CDCl_2CDCl_2$.

Supplementary Table 11. Summary of the Content of the Cage-Guest Complexes in Solutions: Before and After the Free-Guest Elimination As Well As After the Solution Was Kept at 298 K for 2 days.[a]

Guest Species	CD_3CN			5:95(v/v) $CD_3CN/CDCl_3$			acetone-d_6			$CDCl_2CDCl_2$		
	A[b]	B[c]	C[d]	A[b]	B[c]	C[d]	A[b]	B[c]	C[d]	A[b]	B[c]	C[d]
**Ad**	100%	100%	100%	100%	100%	100%	100%	100%	100%	100%	100%	100%
**Ad-MeOH**	100%	100%	100%	100%	100%	100%	100%	100%	100%	100%	100%	100%
**Ad-COOH**	100%	100%	100%	100%	100%	100%	100%	100%	100%	100%	100%	100%

[a] The contents expressed as the fraction of the cage-guest complexes in the mixture of the cage-guest complexes and the free cages that probably exist. [b] Before free-guest elimination. [c] After free-guest elimination. [c] After the complexes in the solution was kept at 298 K for 2 days.

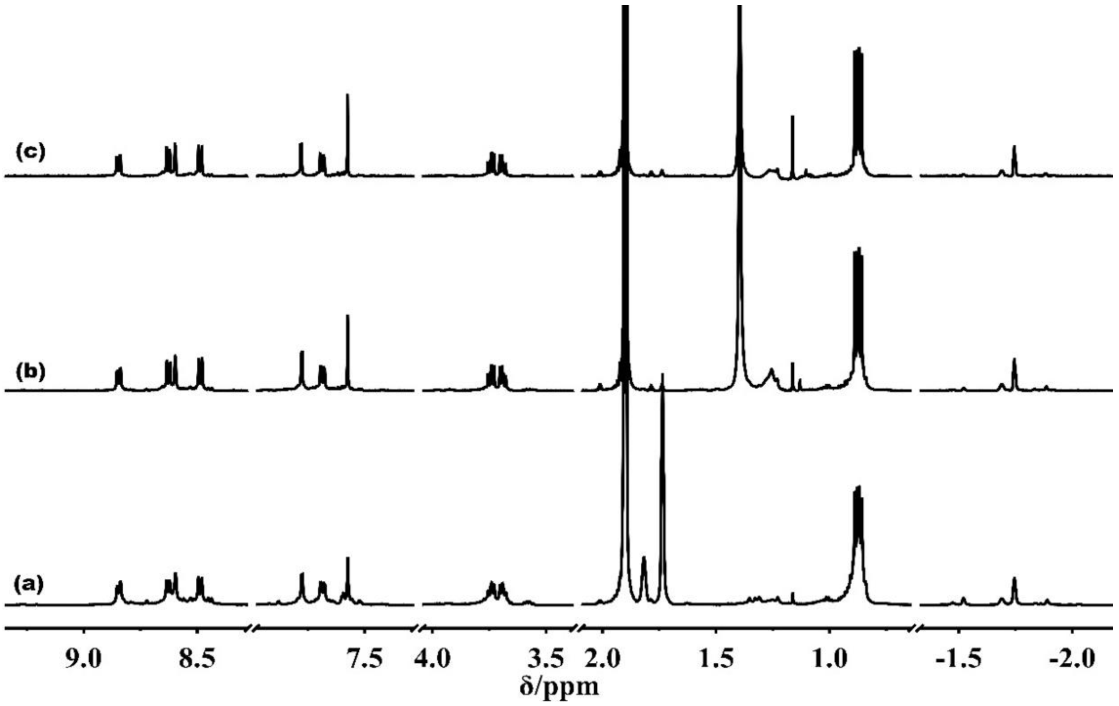

Supplementary Figure 71A. ^1H NMR spectra (600 MHz, 298 K, 1 mM, CD_3CN) of **Ad**⊂$[Hg_5\mathbf{1}_2]\cdot[OTf]_{10}$ in the cases of that: (a) in the presence of excess 7 equiv. of free **Ad** (before free-guest elimination), (b) in the absence of free **Ad** (after free-guest elimination) and (c) after the solution (b) was kept at 298 K for 2 days.

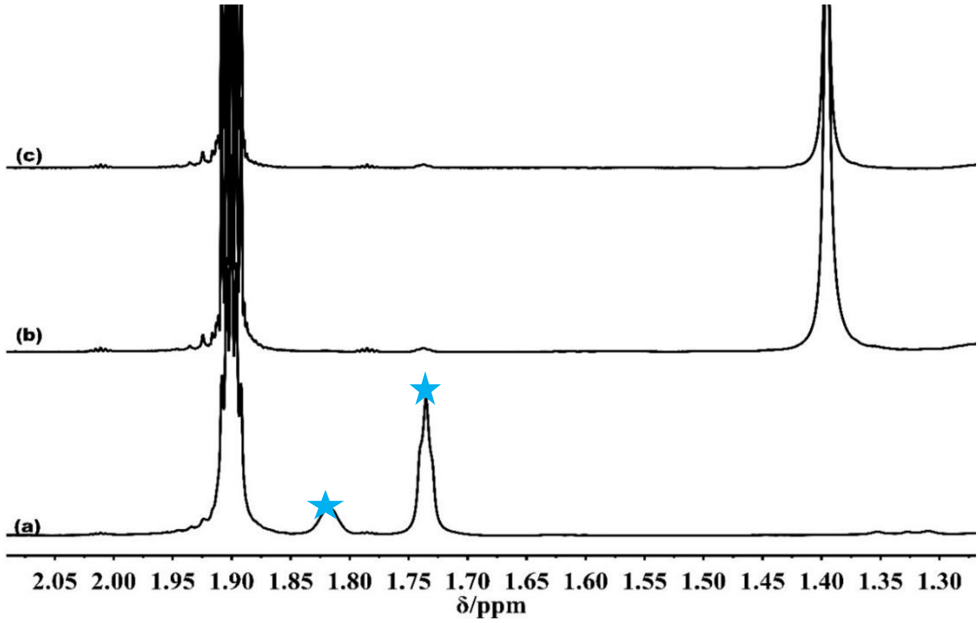

Supplementary Figure 71B. Zoom of the ^{1}H NMR spectra S71A. (a) in the presence of excess 7 equiv. of free **Ad** (before free-guest elimination); (b) in the absence of free **Ad** (after free-guest elimination); (c) after the solution (b) was kept at 298 K for 2 days. ★ = free **Ad**.

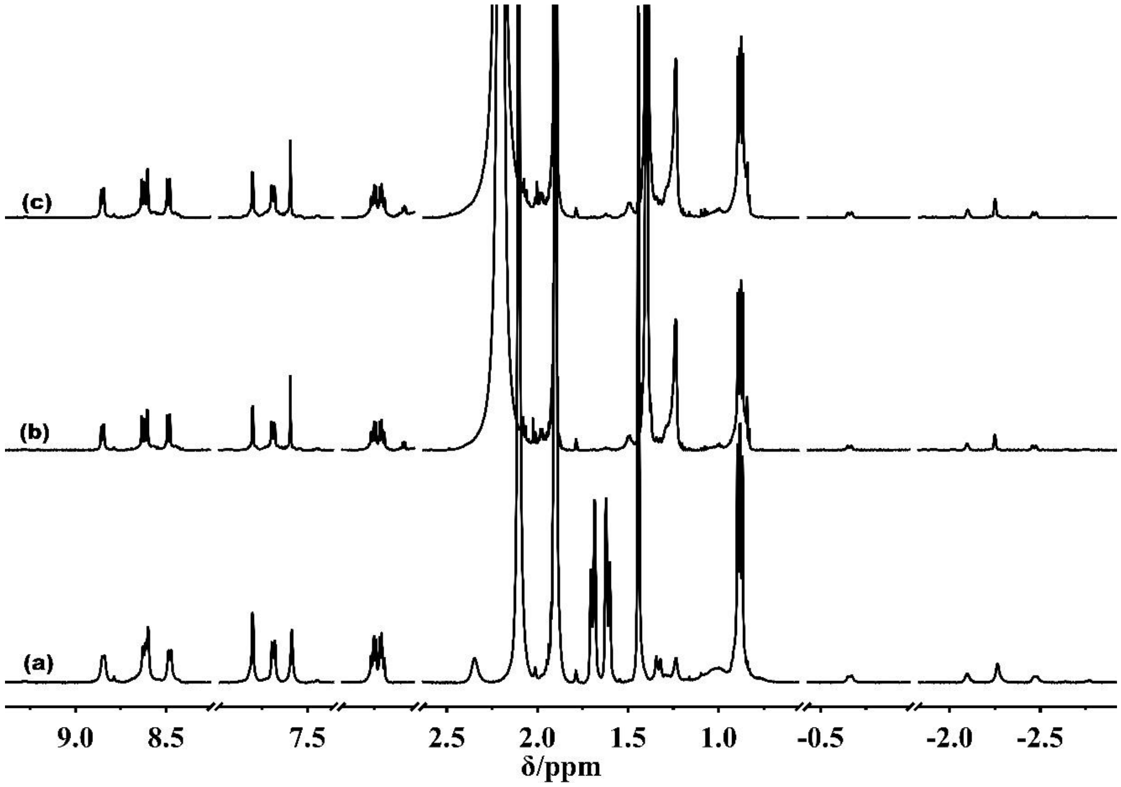

Supplementary Figure 72A. ^{1}H NMR spectra (600 MHz, 298 K, 1 mM, CD_3CN) of **Ad-MeOH**⊂$[Hg_5\mathbf{1}_2]\cdot[OTf]_{10}$ in the cases of that (a) in the presence of excess 7 equiv. of free **Ad-MeOH** (before free-guest elimination); (b) in the absence of free **Ad-MeOH** (after free-guest elimination); (c) after the solution (b) was kept at 298 K for 2 days.

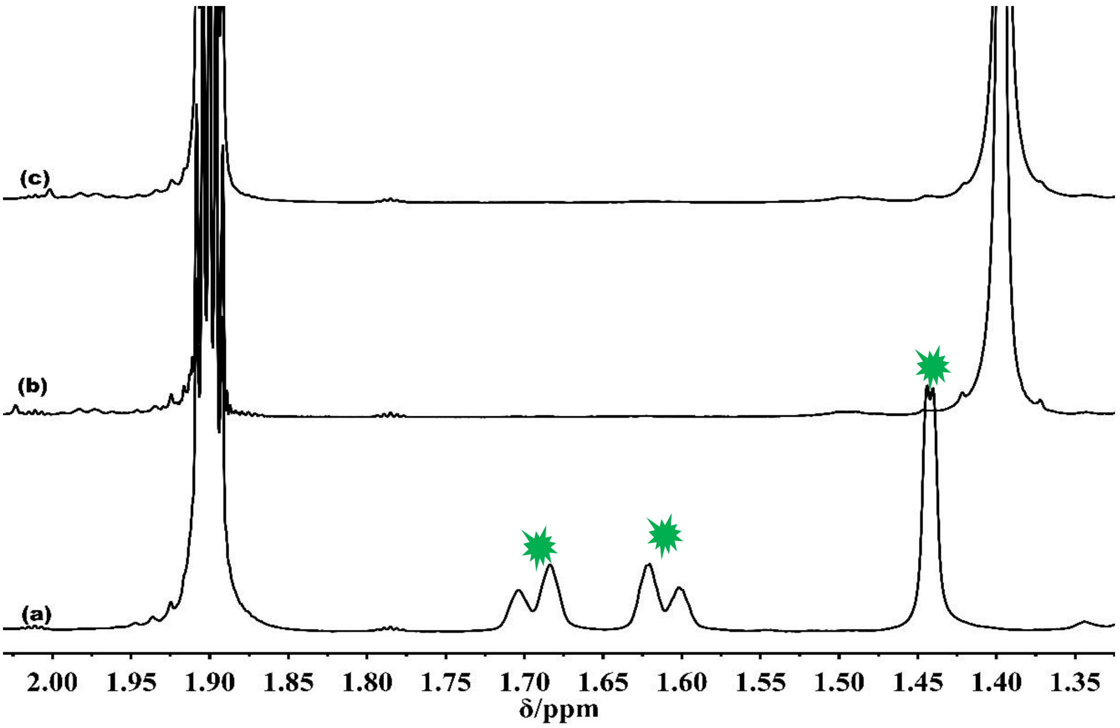

Supplementary Figure 72B. Zoom of the ^{1}H NMR spectra S72A. (a) in the presence of excess 7 equiv. of free **Ad-MeOH** (before free-guest elimination); (b) in the absence of free **Ad-MeOH** (after free-guest elimination); (c) after the solution (b) was kept at 298 K for 2 days. ✹ = free **Ad-MeOH**.

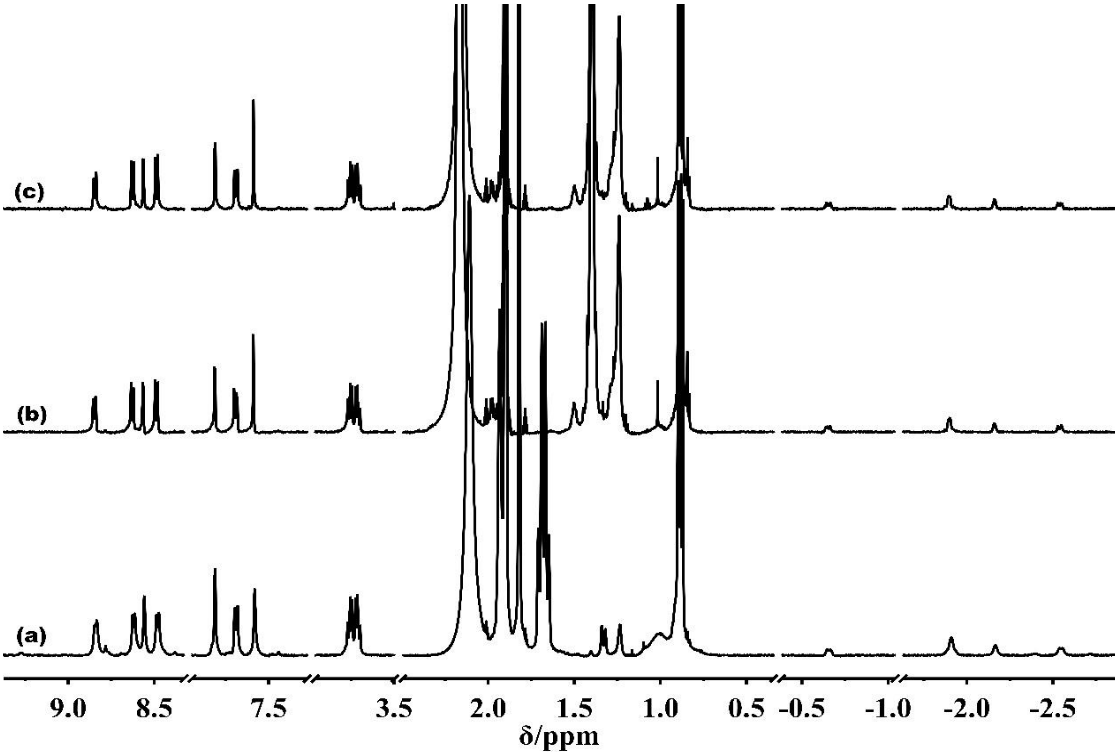

Supplementary Figure 73A. ^{1}H NMR spectra (600 MHz, 298 K, 1 mM, CD_3CN) of **Ad-COOH**$\subset[Hg_5\mathbf{1}_2]\cdot[OTf]_{10}$ in the cases of that (a) in the presence of excess 7 equiv. of free **Ad-COOH** (before free-guest elimination); (b) in the absence of free **Ad-COOH** (after free-guest elimination); (c) after the solution (b) was kept at 298 K for 2 days.

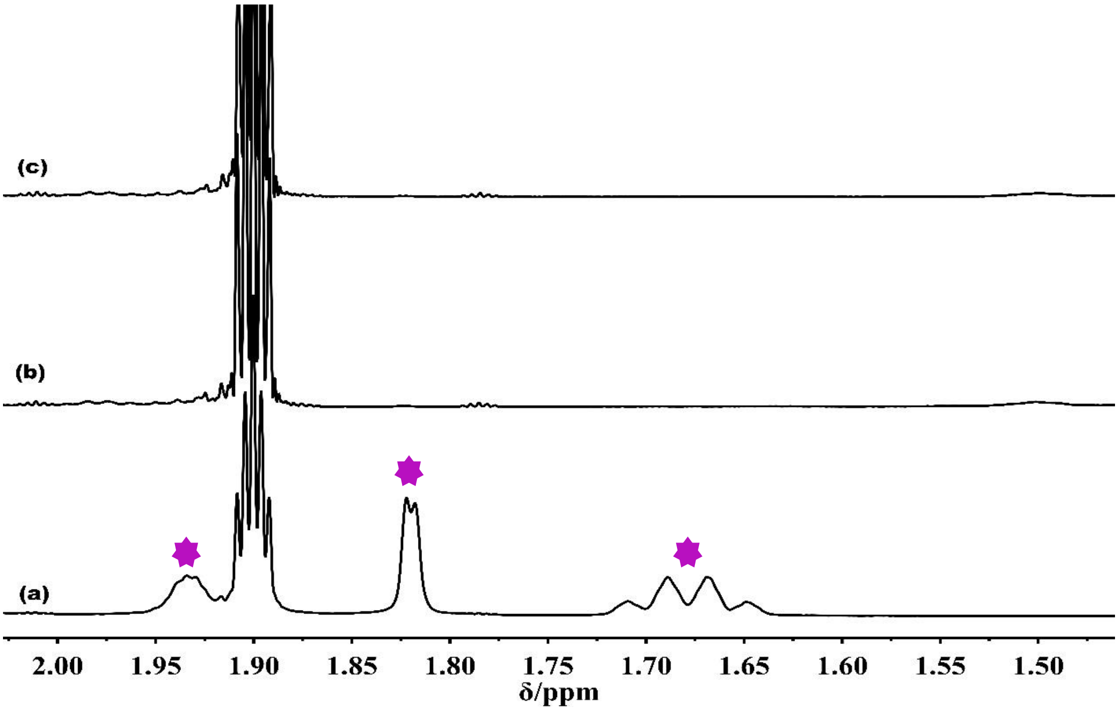

Supplementary Figure 73B. Zoom of the ^{1}H NMR spectra S73A. (a) in the presence of excess 7 equiv. of free **Ad-COOH** (before free-guest elimination); (b) in the absence of free **Ad-COOH** (after free-guest elimination); (c) after the solution (b) was kept at 298 K for 2 days. ✸= free **Ad-COOH**.

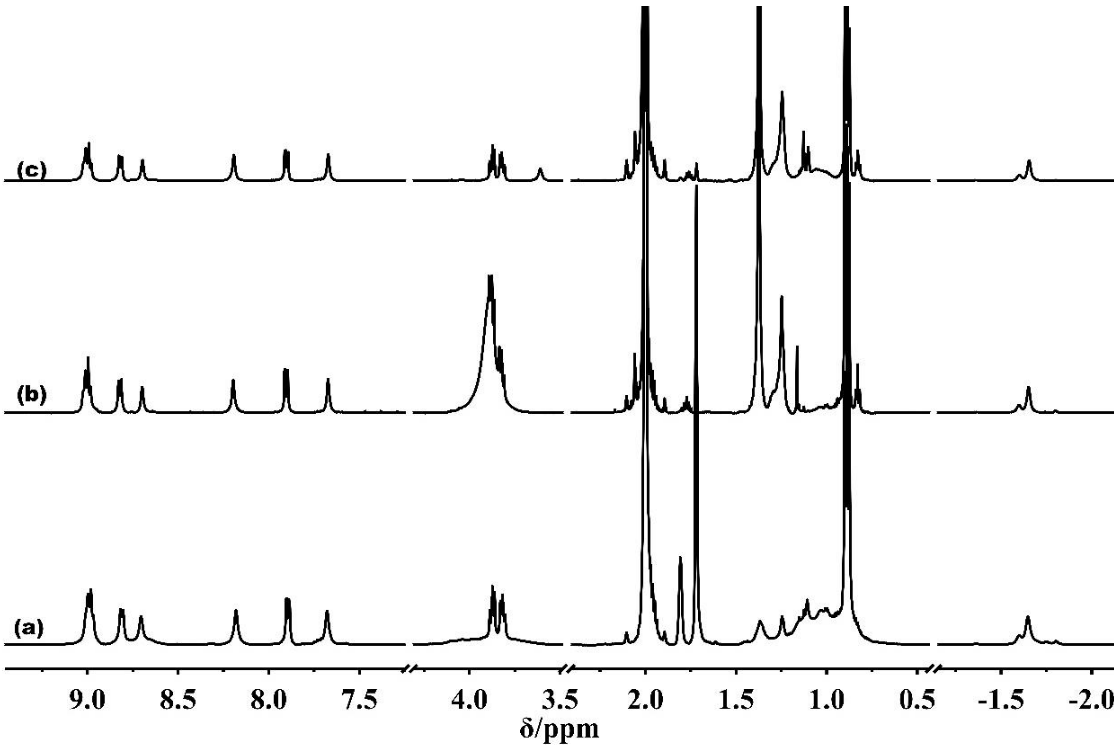

Supplementary Figure 74A. ^{1}H NMR spectra (600 MHz, 298 K, 1 mM, acetone-d_6) of **Ad**⊂[Hg_5**1**$_2$]·$[OTf]_{10}$ in the cases of: (a) in the presence of excess 7 equiv. of free **Ad** (before free-guest elimination); (b) in the absence of free **Ad** (after free-guest elimination); and (c) after the solution (b) was kept at 298 K for 2 days.

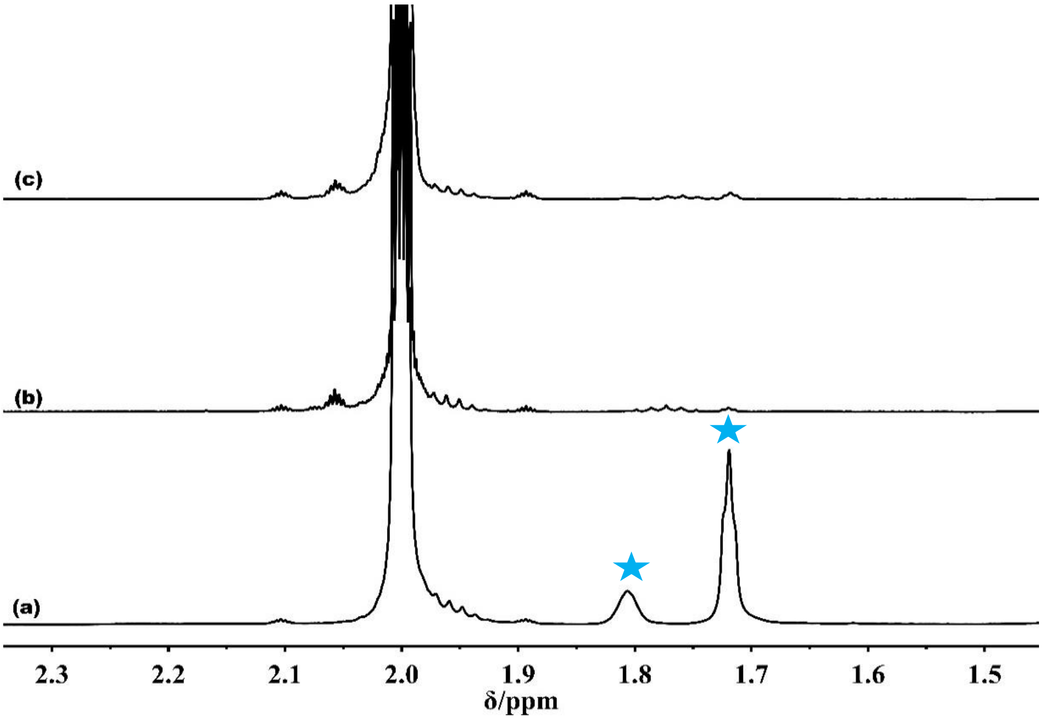

Supplementary Figure 74B. Zoom of the ^{1}H NMR spectra S74A. (a) in the presence of excess 7 equiv. of free **Ad** (before free-guest elimination); (b) in the absence of free **Ad** (after free-guest elimination); (c) after the solution (b) was kept at 298 K for 2 days. ★ = free **Ad**.

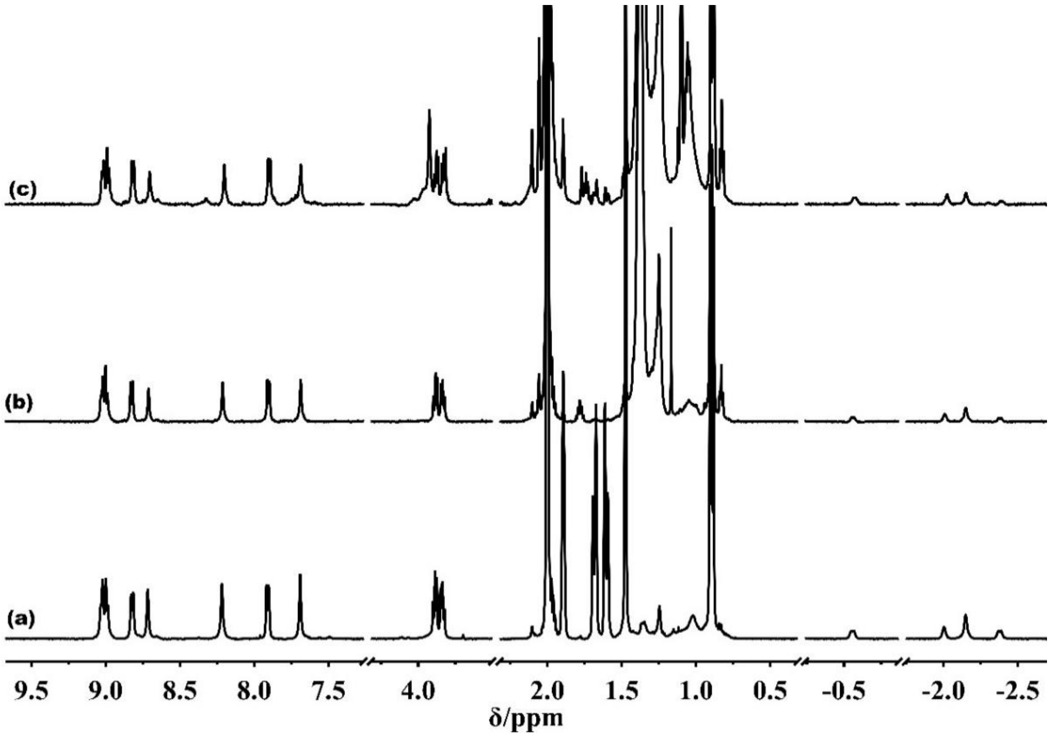

Supplementary Figure 75A. ^{1}H NMR spectra (600 MHz, 298 K, 1 mM, acetone-d_6) of **Ad-MeOH**$\subset$ [Hg$_5$**1**$_2$]·[OTf]$_{10}$ in the cases of that (a) in the presence of excess 7 equiv. of free **Ad-MeOH** (before free-guest elimination); (b) in the absence of free **Ad-MeOH** (after free-guest elimination); (c) after the solution (b) was kept at 298 K for 2 days.

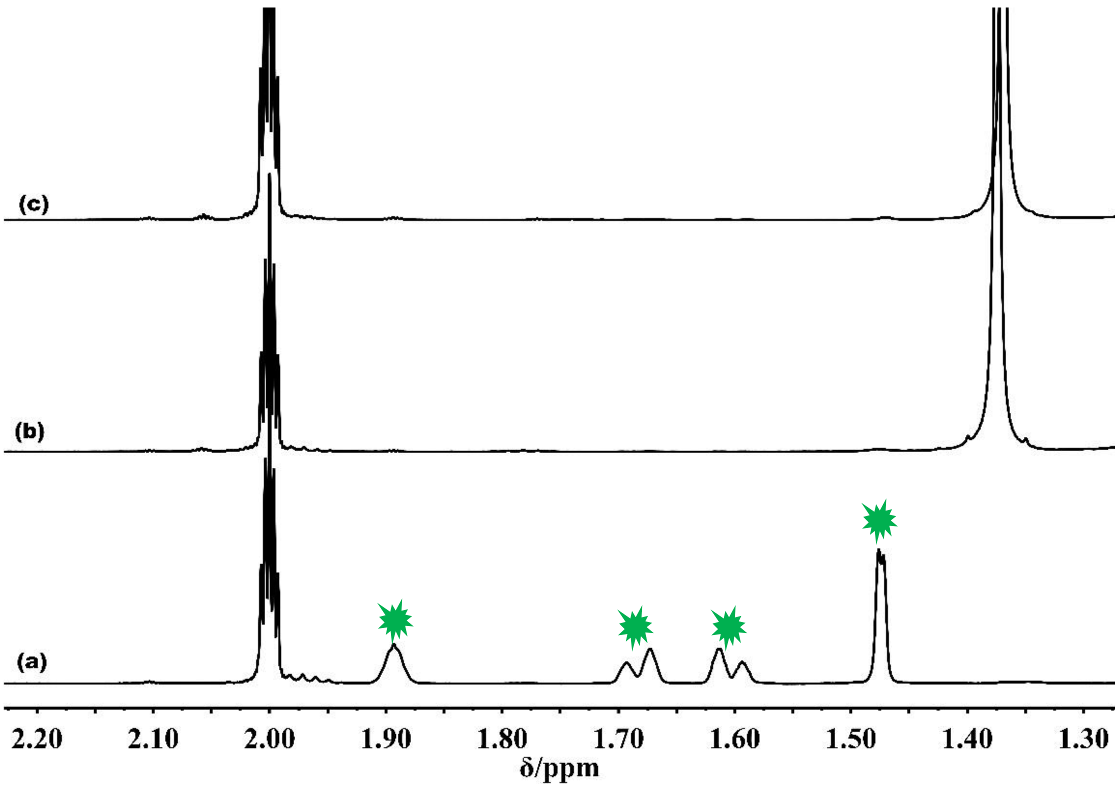

Supplementary Figure 75B. Zoom of the ^{1}H NMR spectra S75A. (a) in the presence of excess 7 equiv. of free **Ad-MeOH** (before free-guest elimination); (b) in the absence of free **Ad-MeOH** (after free-guest elimination); (c) after the solution (b) was kept at 298 K for 2 days. ✸= free **Ad-MeOH**.

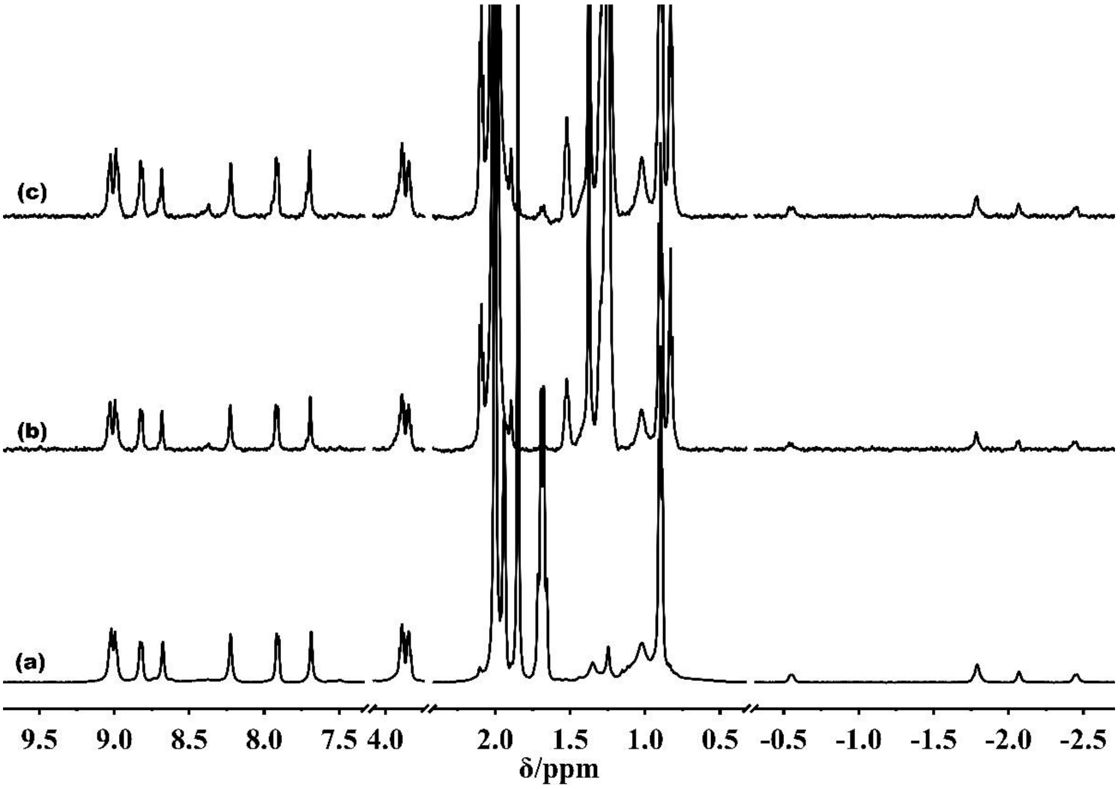

Supplementary Figure 76A. ^{1}H NMR spectra (600 MHz, 298 K, 1 mM, acetone-d_6) of **Ad-COOH**⊂ [Hg_5**1**$_2$]·[OTf]$_{10}$ in the cases of that (a) in the presence of excess 7 equiv. of free **Ad-COOH** (before free-guest elimination); (b) in the absence of free **Ad-COOH** (after free-guest elimination); (c) after the solution (b) was kept at 298 K for 2 days.

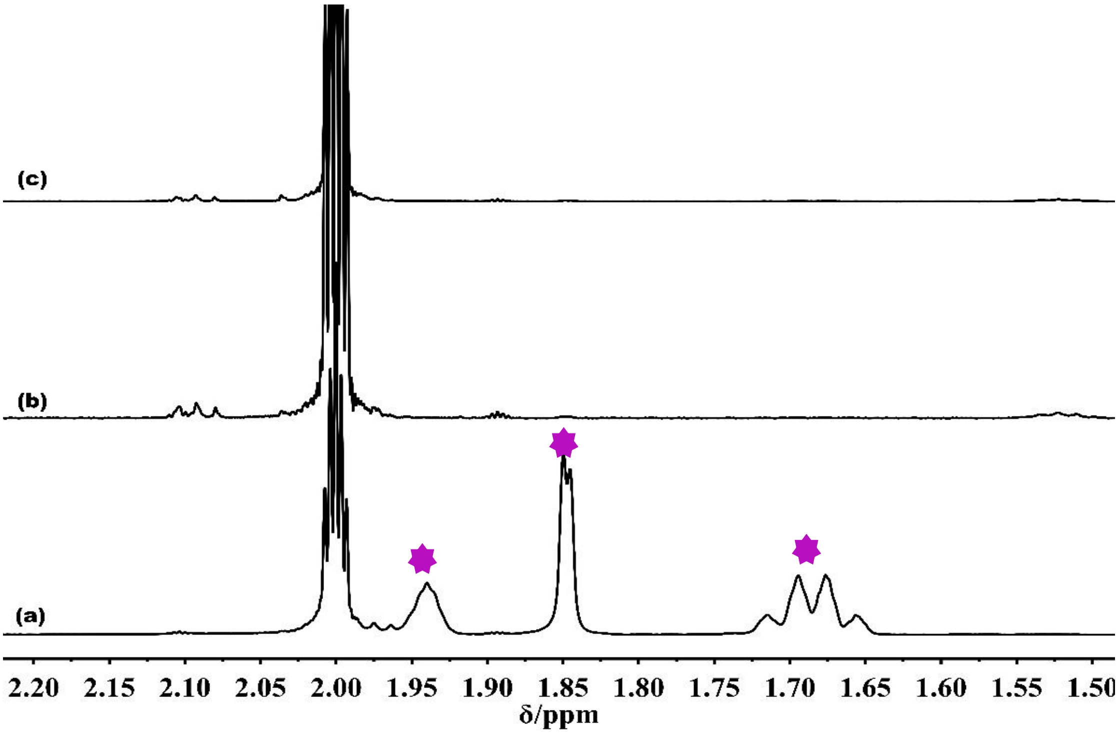

Supplementary Figure 76B. Zoom of the ^{1}H NMR spectra S76A. (a) in the presence of excess 7 equiv. of free **Ad-COOH** (before free-guest elimination); (b) in the absence of free **Ad-COOH** (after free-guest elimination); (c) after the solution (b) was kept at 298 K for 2 days. ✸ = free **Ad-COOH**.

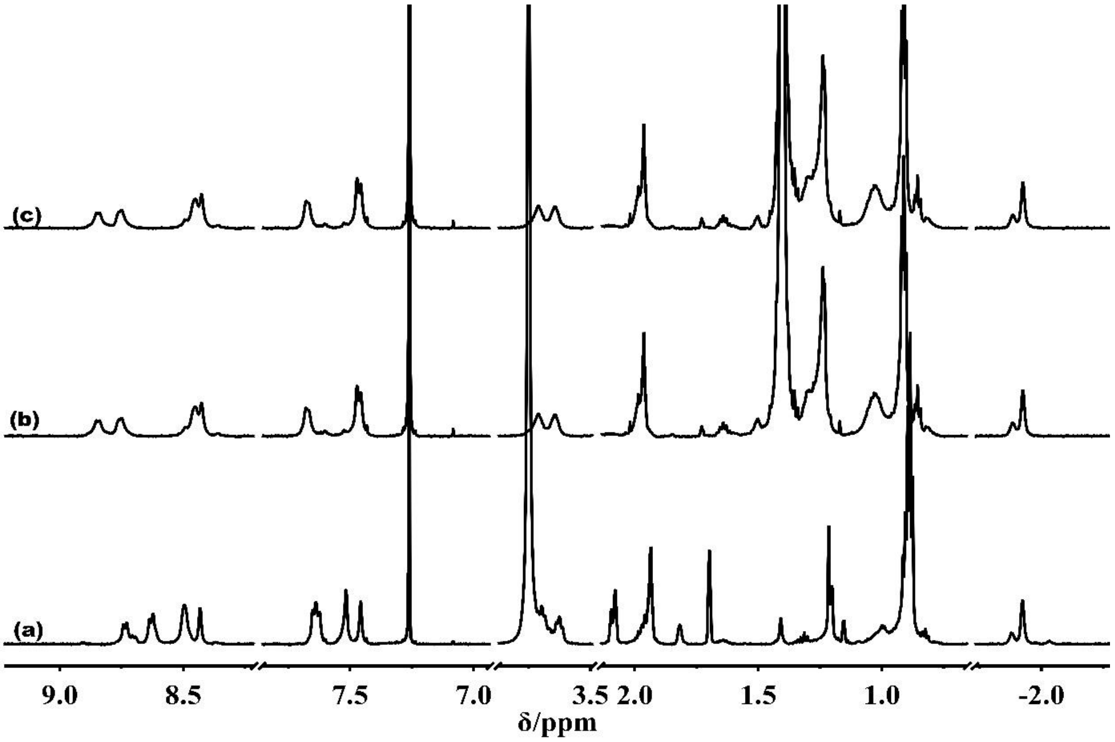

Supplementary Figure 77A. ^{1}H NMR spectra (600 MHz, 298 K, 1 mM, 5:95(v/v) $CD_3CN/CDCl_3$) of **Ad**⊂[Hg$_5$**1**$_2$]·[OTf]$_{10}$ in the cases of: (a) in the presence of excess 7 equiv. of free **Ad** (before free-guest elimination); (b) in the absence of free **Ad** (after free-guest elimination); and (c) after the solution (b) was kept at 298 K for 2 days.

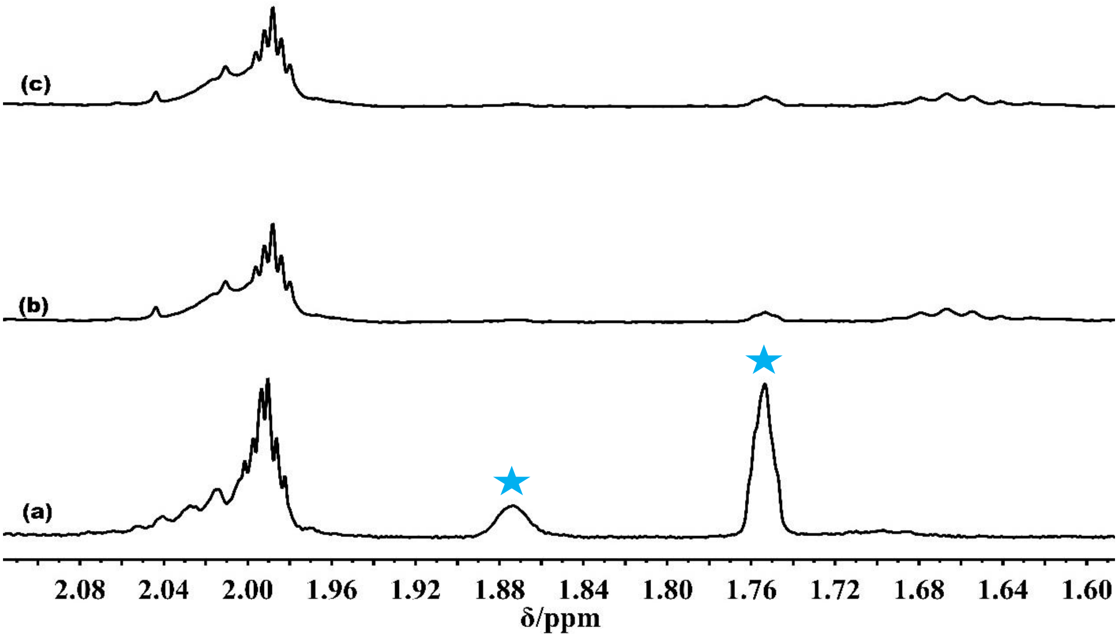

Supplementary Figure 77B. Zoom of the ^{1}H NMR spectra S77A. (a) in the presence of excess 7 equiv. of free **Ad** (before free-guest elimination); (b) in the absence of free **Ad** (after free-guest elimination); (c) after the solution (b) was kept at 298 K for 2 days. ★ = free **Ad**.

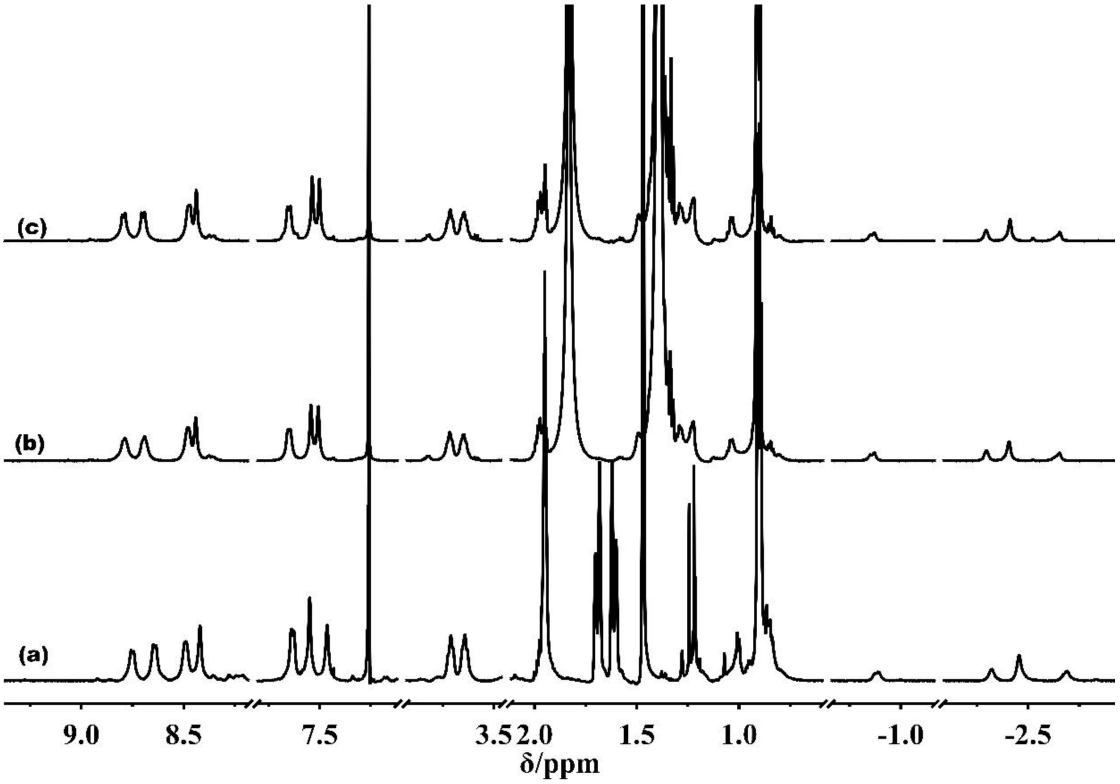

Supplementary Figure 78A. ^{1}H NMR spectra (600 MHz, 298 K, 1 mM, 5:95(v/v) $CD_3CN/CDCl_3$) of **Ad**⊂[Hg_5**1**$_2$]·[OTf]$_{10}$ in the cases of: (a) in the presence of excess 7 equiv. of free **Ad-MeOH** (before free-guest elimination); (b) in the absence of free **Ad-MeOH** (after free-guest elimination); and (c) after the solution (b) was kept at 298 K for 2 days.

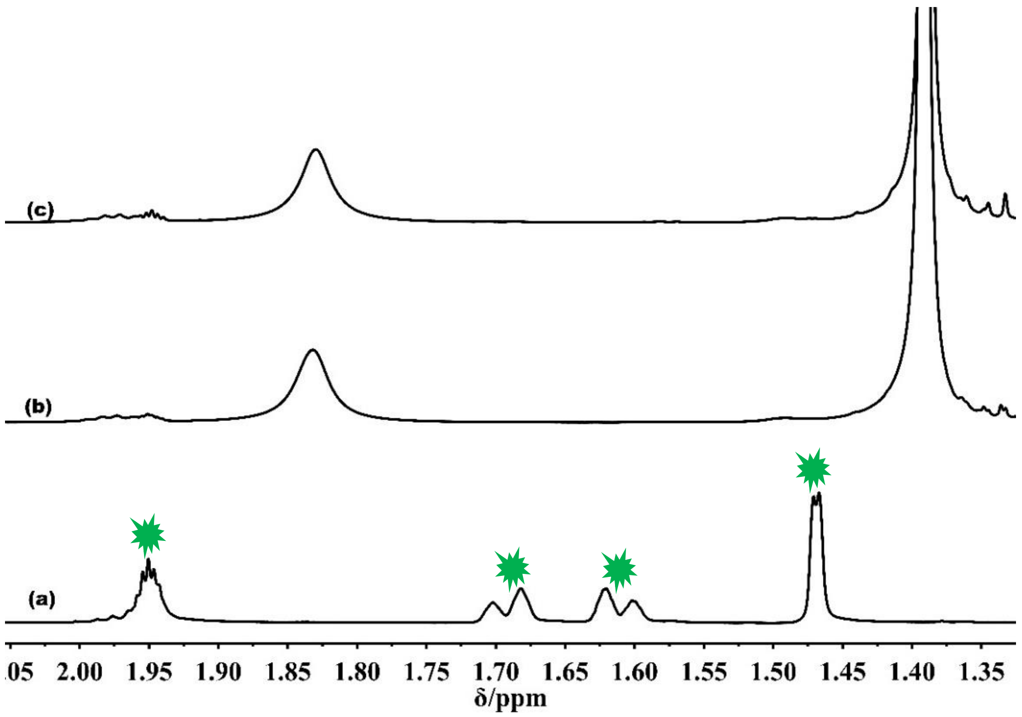

Supplementary Figure 78B. Zoom of the ^{1}H NMR spectra S78A. (a) in the presence of excess 7 equiv. of free **Ad-MeOH** (before free-guest elimination); (b) in the absence of free **Ad-MeOH** (after free-guest elimination); (c) after the solution (b) was kept at 298 K for 2 days. = free **Ad-MeOH**.

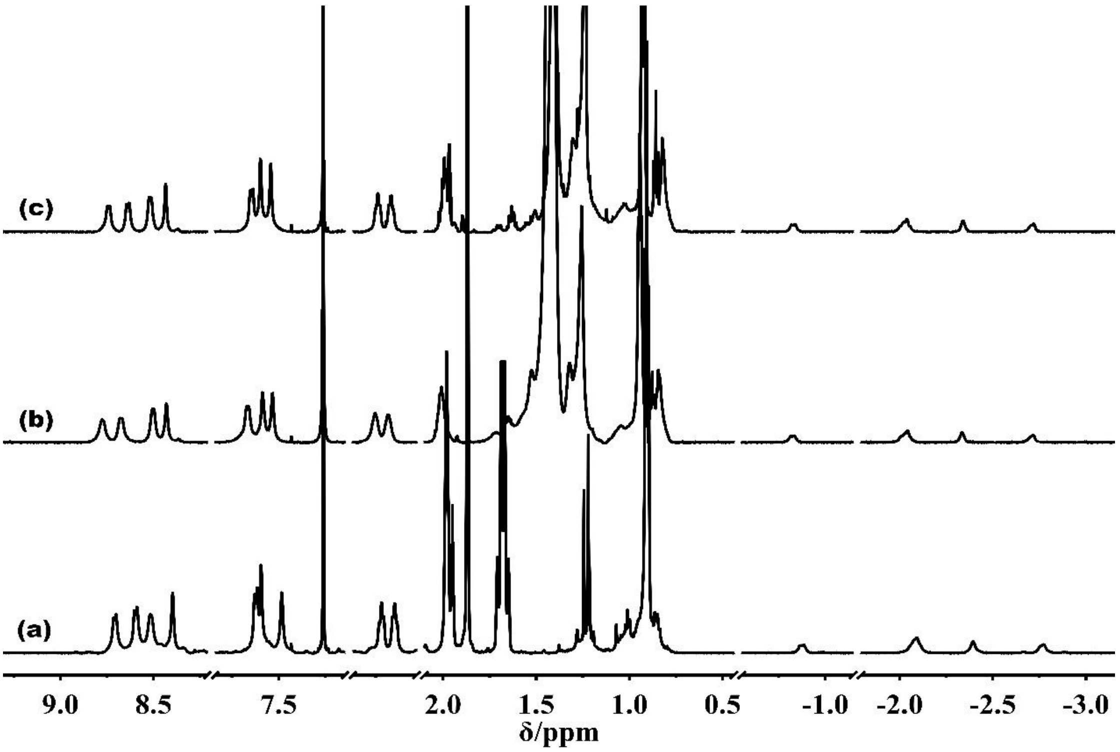

Supplementary Figure 79A. ^{1}H NMR spectra (600 MHz, 298 K, 1 mM, 5:95 (v/v) $CD_3CN/CDCl_3$) of **Ad-COOH**⊂$[Hg_5\mathbf{1}_2]\cdot[OTf]_{10}$ in the cases of: (a) in the presence of excess 7 equiv. of free **Ad** (before free-guest elimination); (b) in the absence of free **Ad-COOH** (after free-guest elimination); and (c) after the solution (b) was kept at 298 K for 2 days..

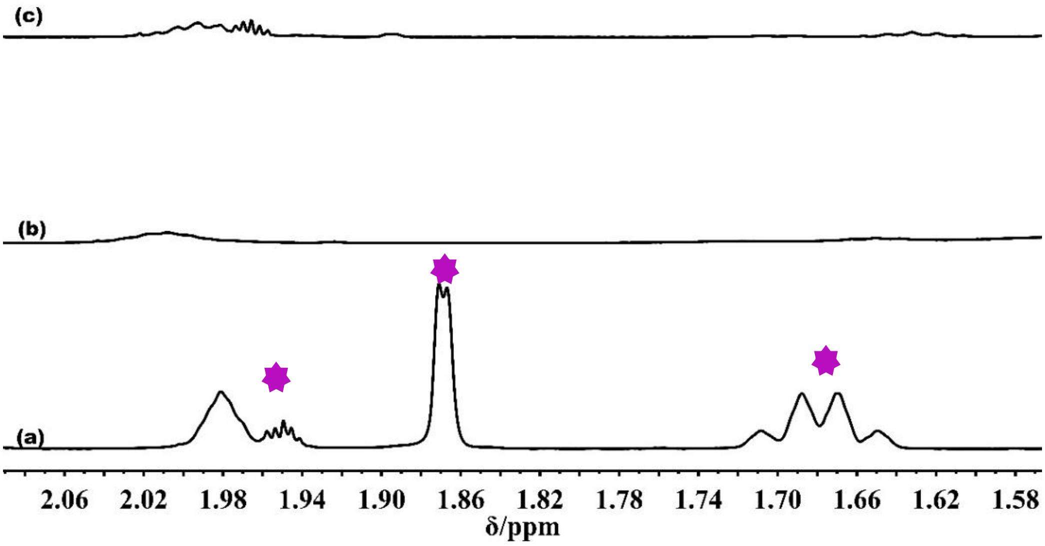

Supplementary Figure 79B. Zoom of the ^{1}H NMR spectra S79A. (a) in the presence of excess 7 equiv. of free **Ad-COOH** (before free-guest elimination); (b) in the absence of free **Ad-COOH** (after free-guest elimination); (c) after the solution (b) was kept at 298 K for 2 days. ✸= free **Ad-COOH**.

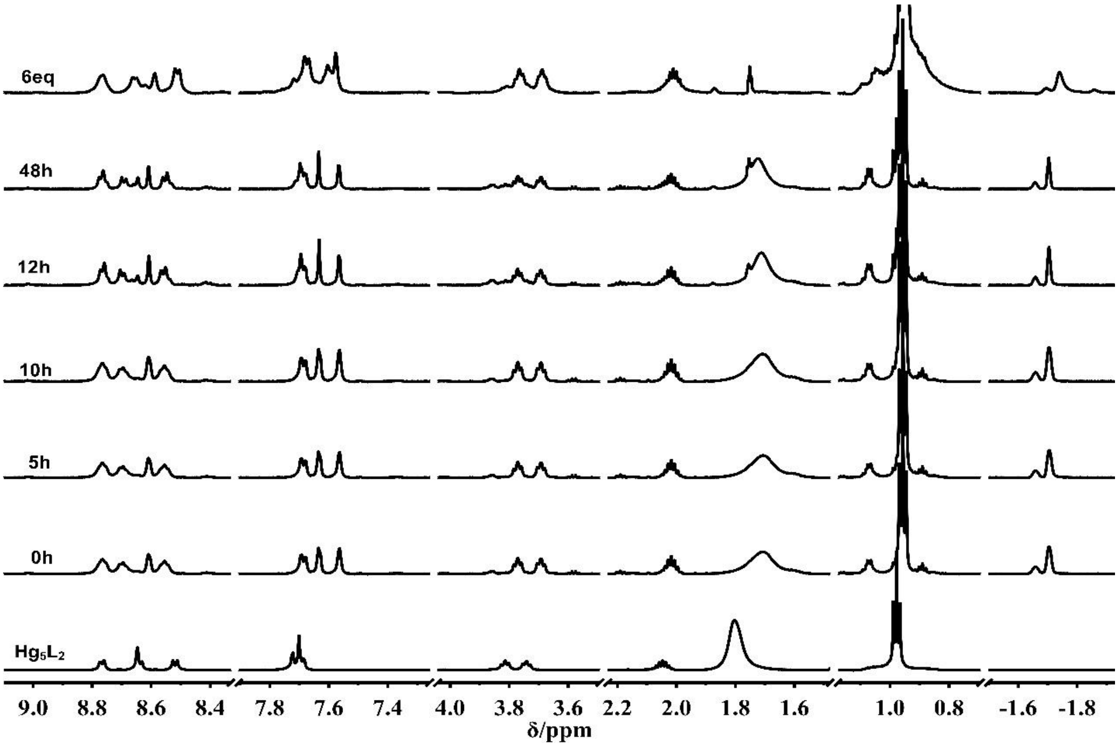

Supplementary Figure 80. Time-depended ^{1}H NMR spectra (600 MHz, 298 K, 1 mM, $CDCl_2CDCl_2$) of **Ad**⊂[Hg_5**1**$_2$]·[OTf]$_{10}$ in the absence of free **Ad**. ^{1}H NMR spectra of the free cages and the cage-guest complexes sample without free-guest elimination are also shown for comparison.

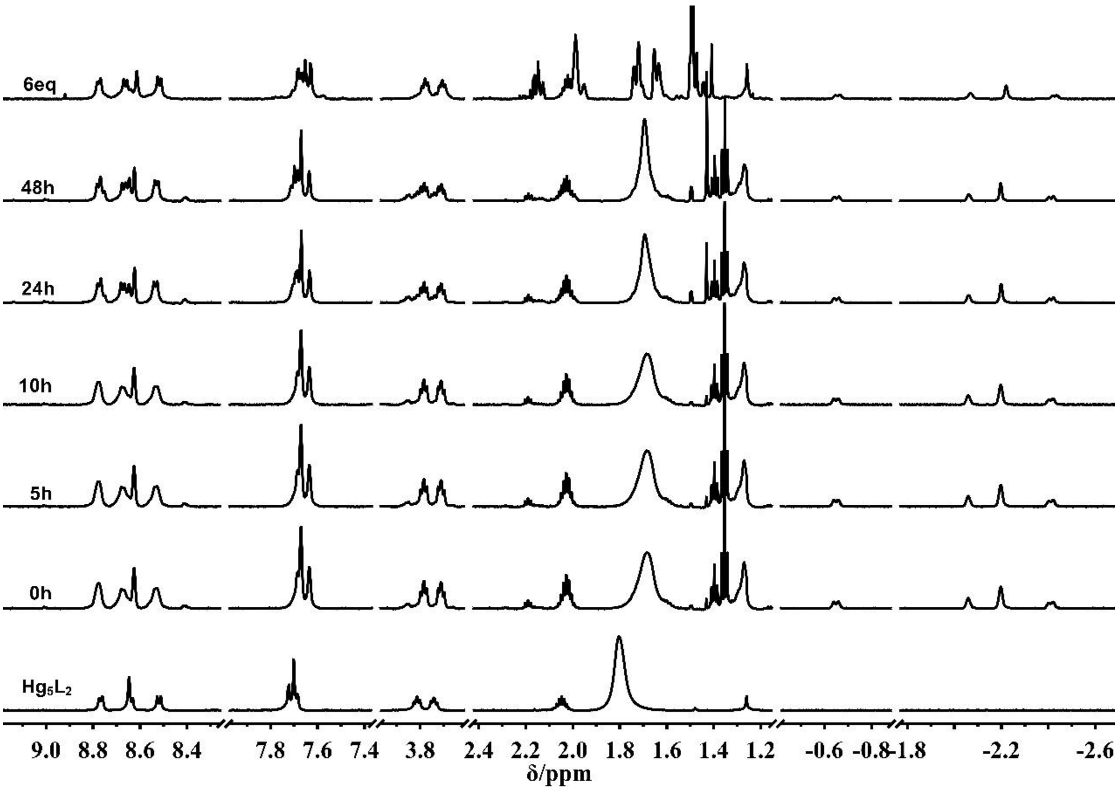

Supplementary Figure 81. Time-depended ^{1}H NMR spectra (600 MHz, 298 K, 1 mM, $CDCl_2CDCl_2$) of **Ad-MeOH**⊂[Hg_5**1**$_2$]·[OTf]$_{10}$ in the absence of free **Ad-MeOH**. ^{1}H NMR spectra of the free cages and the cage-guest complexes sample without free-guest elimination are also shown for comparison.

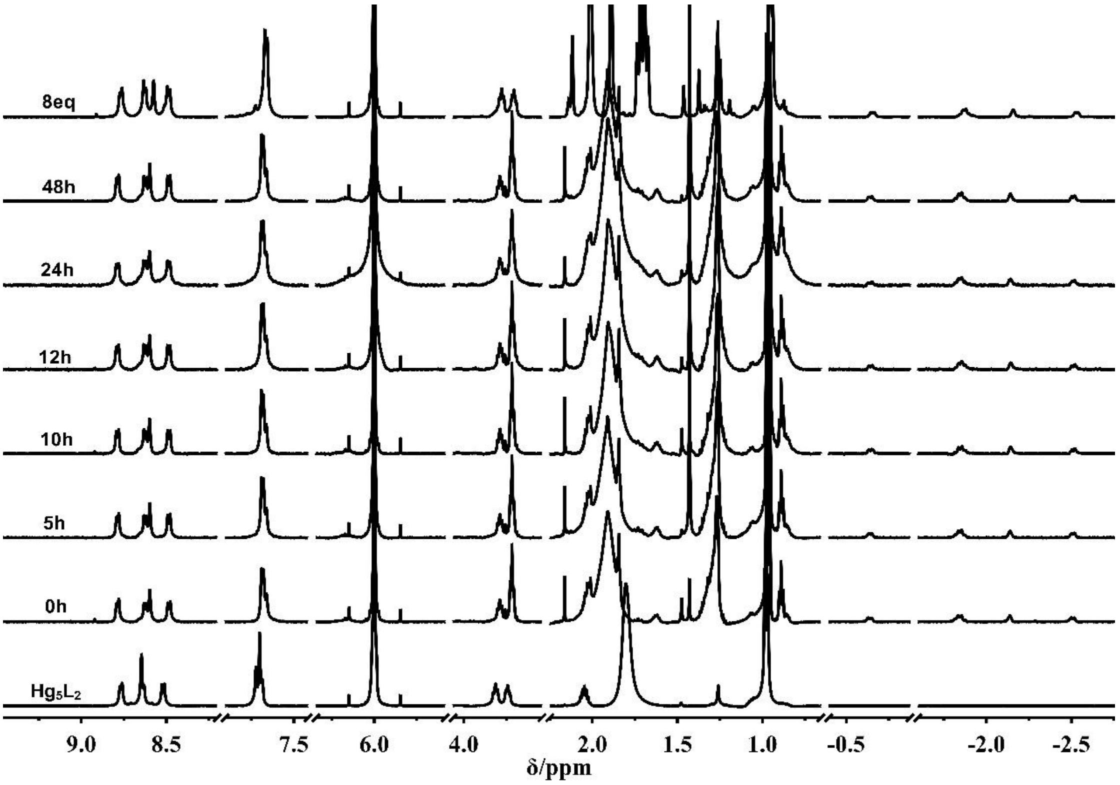

Supplementary Figure 82. Time-depended ^{1}H NMR spectra (600 MHz, 298 K, 1 mM, $CDCl_2CDCl_2$) of **Ad-COOH**⊂[Hg_5**1**$_2$]·[OTf]$_{10}$ in the absence of free **Ad-COOH**. ^{1}H NMR spectra of the free cages and the cage-guest complexes sample without free-guest elimination are also shown for comparison.

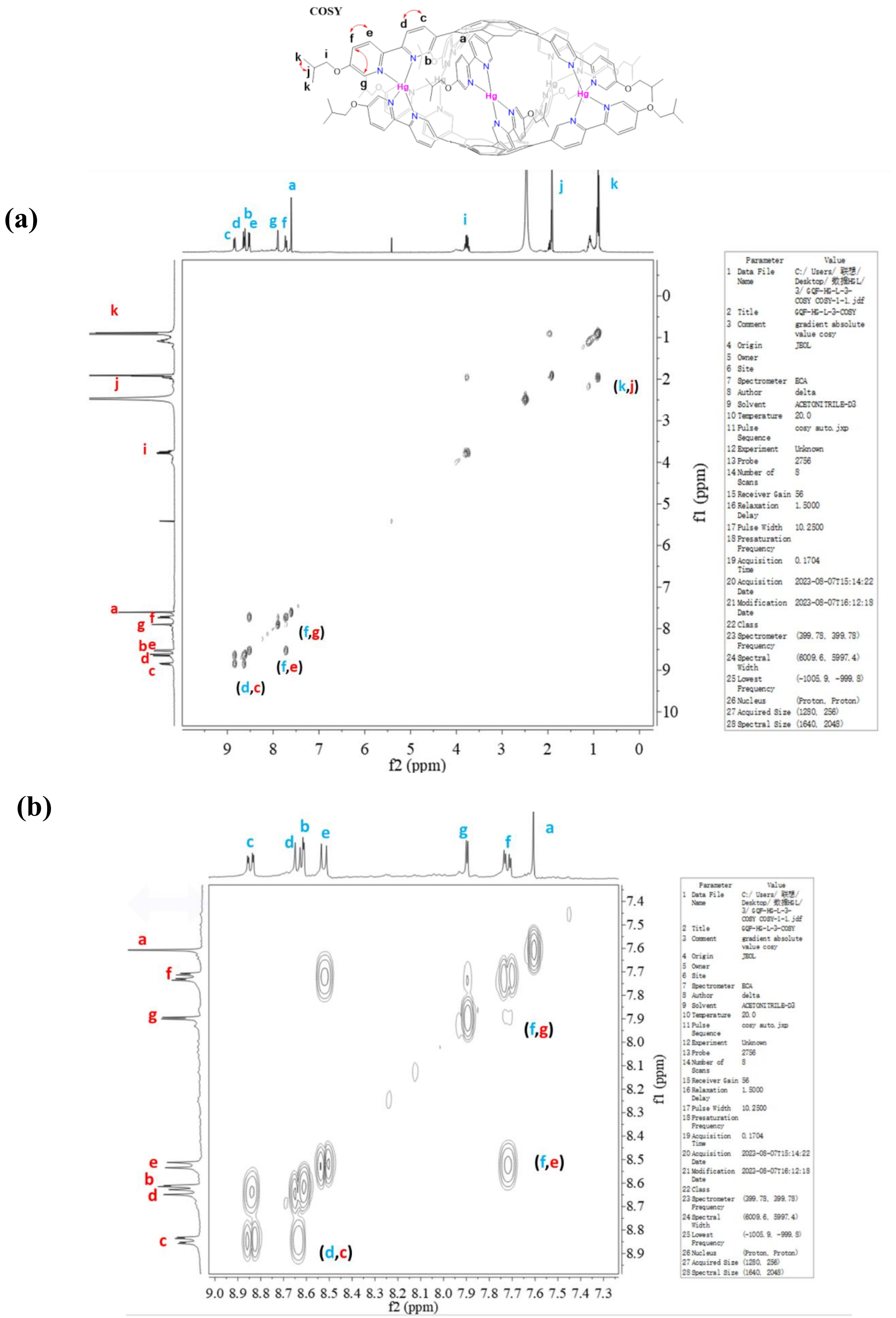

Supplementary Figure 83. **(a)** ^{1}H–^{1}H COSY spectrum (500 MHz, 298 K, 5mM, CD_3CN) of $[Hg_5\mathbf{1}_2]\cdot[OTf]_{10}$. **(b)** Zoom of the spectrum (a).

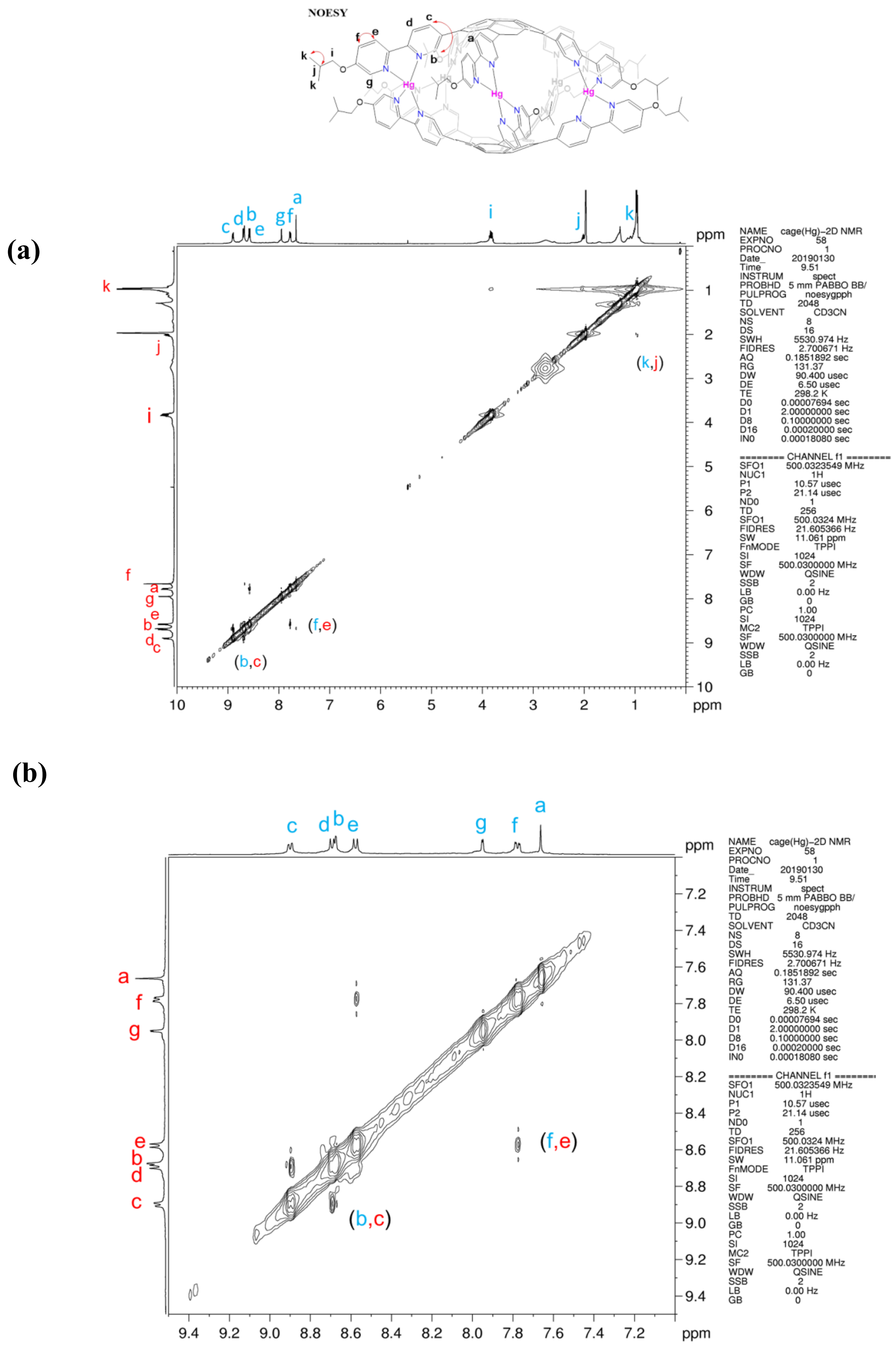

Supplementary Figure 84. (a) $^{1}H–^{1}H$ NOESY spectrum (500 MHz, 298 K, CD_3CN) of $[Hg_5\mathbf{1}_2]\cdot[OTf]_{10}$ (5 mM). (b) Zoom of spectrum a.

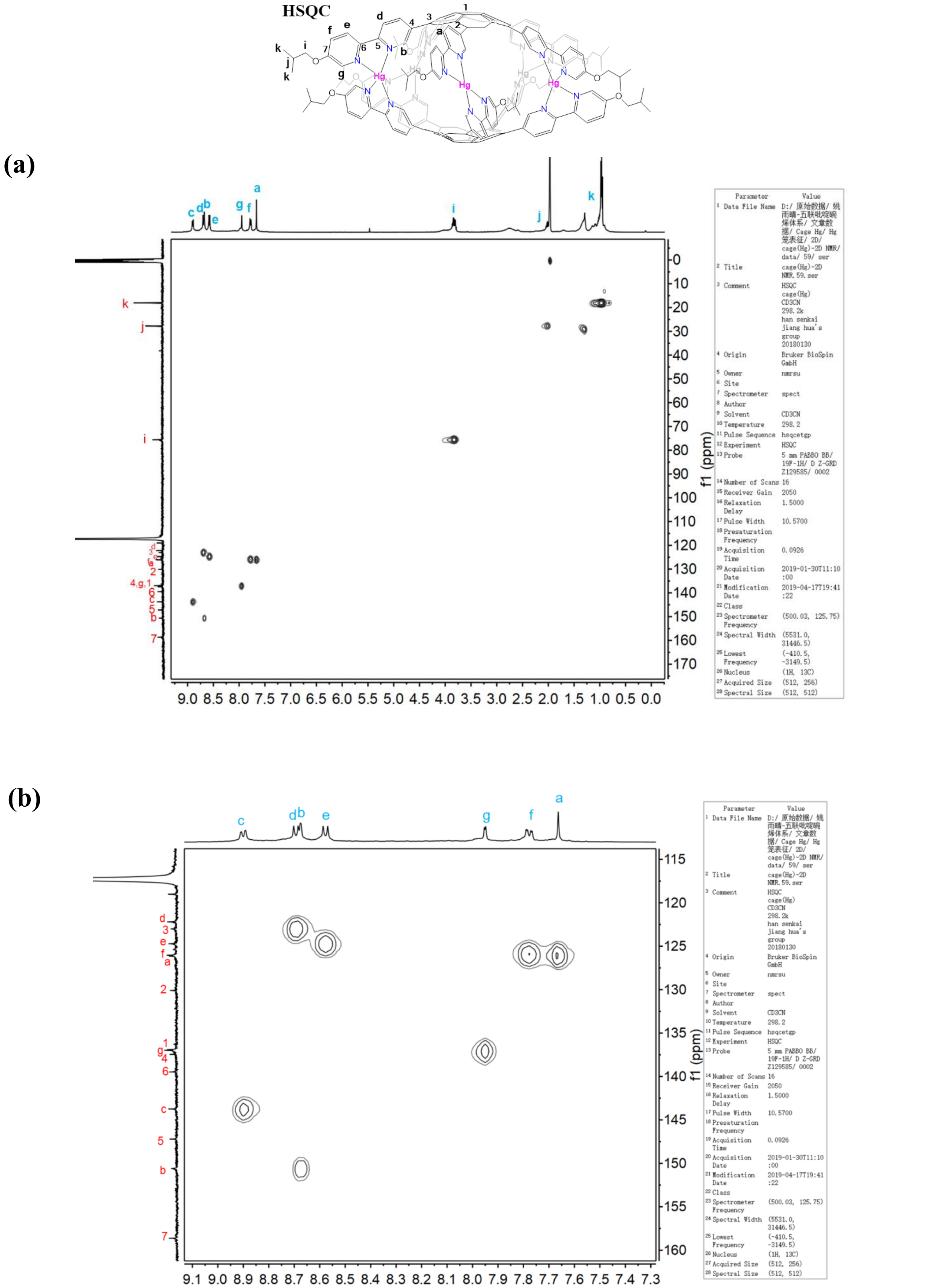

Supplementary Figure 85. (a) ^{1}H–^{13}C HSQC spectrum (500 MHz, 298 K, CD_3CN) of $[Hg_5\mathbf{1}_2]\cdot[OTf]_{10}$ (5 mM). (b) Zoom of spectrum (a).

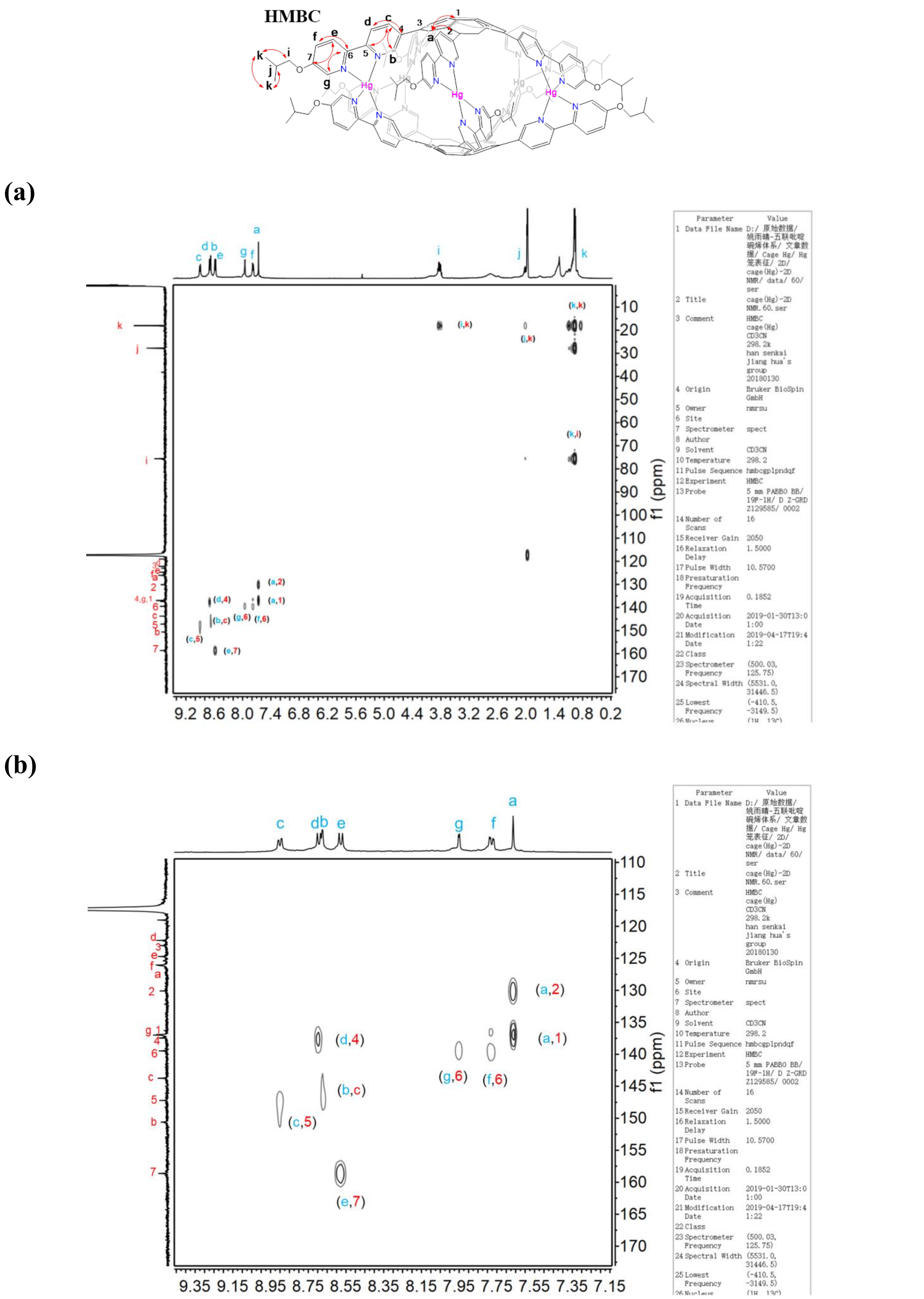

Supplementary Figure 86. (a) ^{1}H–^{13}C HMBC spectrum (500 MHz, 298 K, CD_3CN) of $[Hg_5\mathbf{1}_2]\cdot[OTf]_{10}$ (5 mM). (b) Zoom of spectrum (a).

COSY

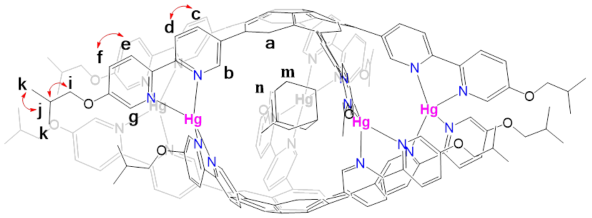

(a)

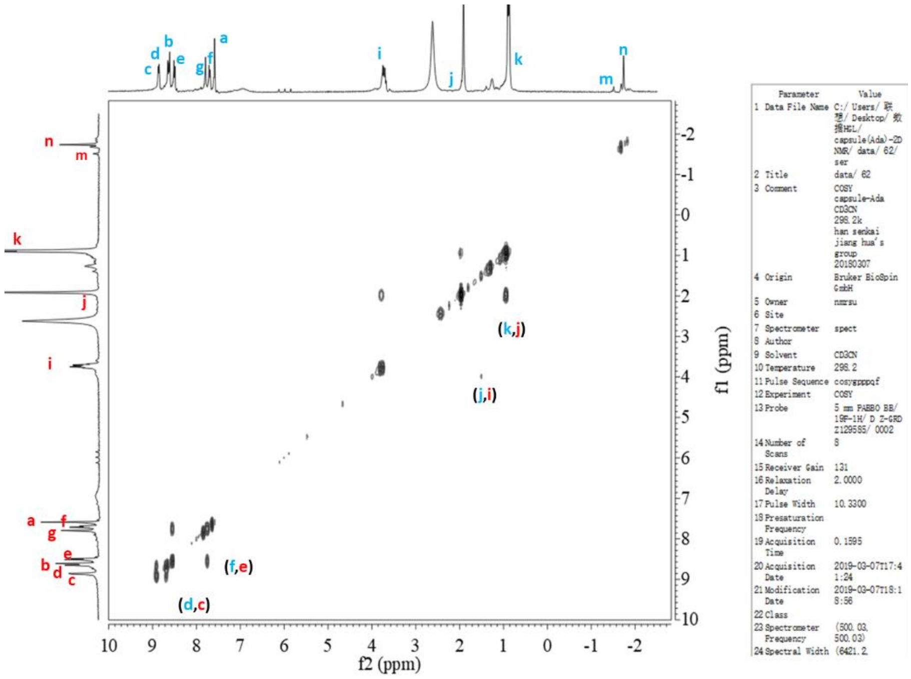

(b)

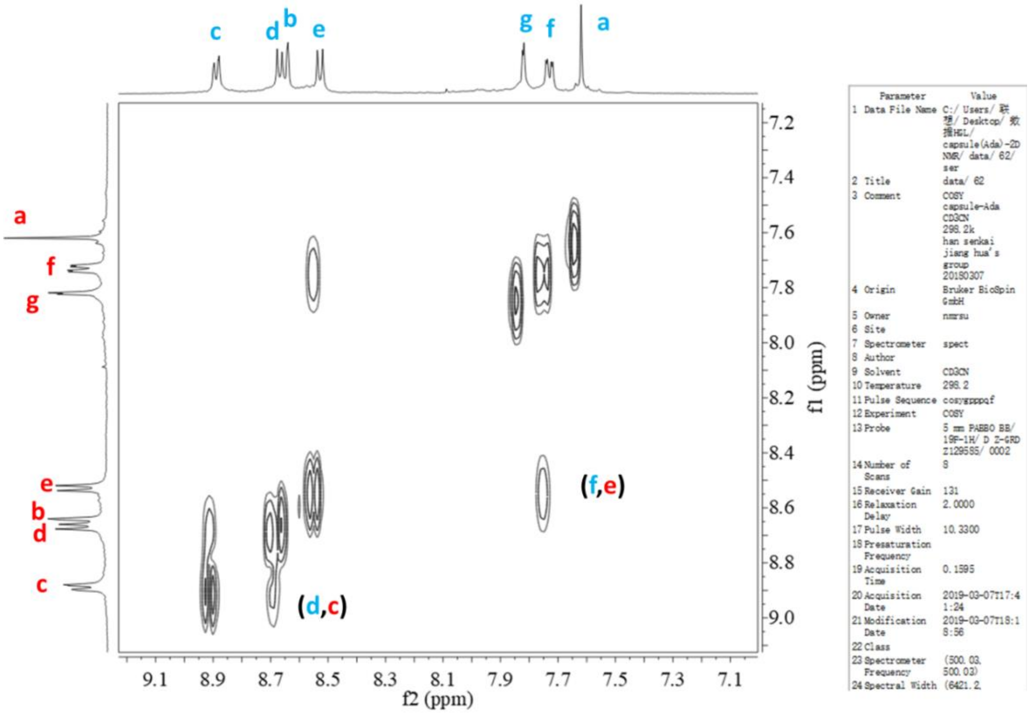

Supplementary Figure 87. (a) ^{1}H–^{1}H COSY spectrum (500 MHz, 298 K, 5mM, CD_3CN) of the **Ad**⊂ [Hg_5**1**$_2$]·[OTf]$_{10}$. **(b)** Zoom of the spectrum (a).

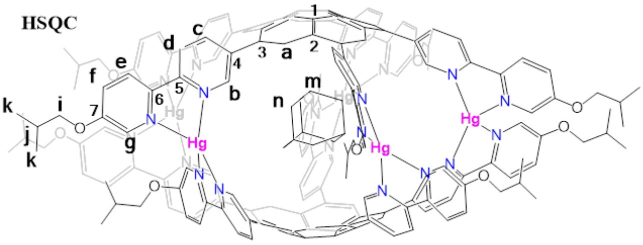

(a)

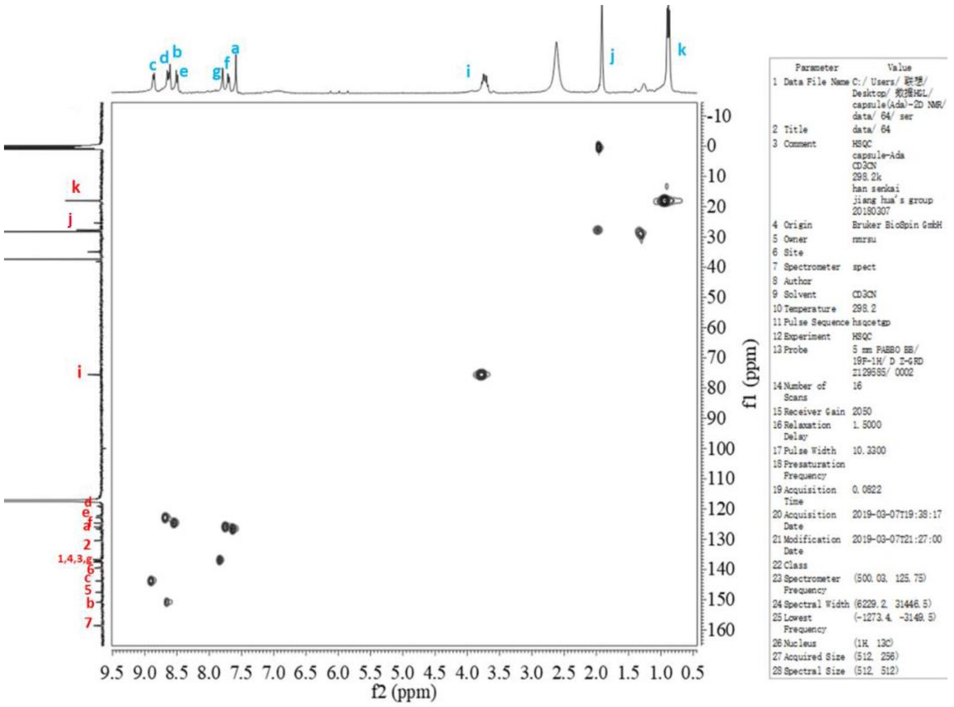

(b)

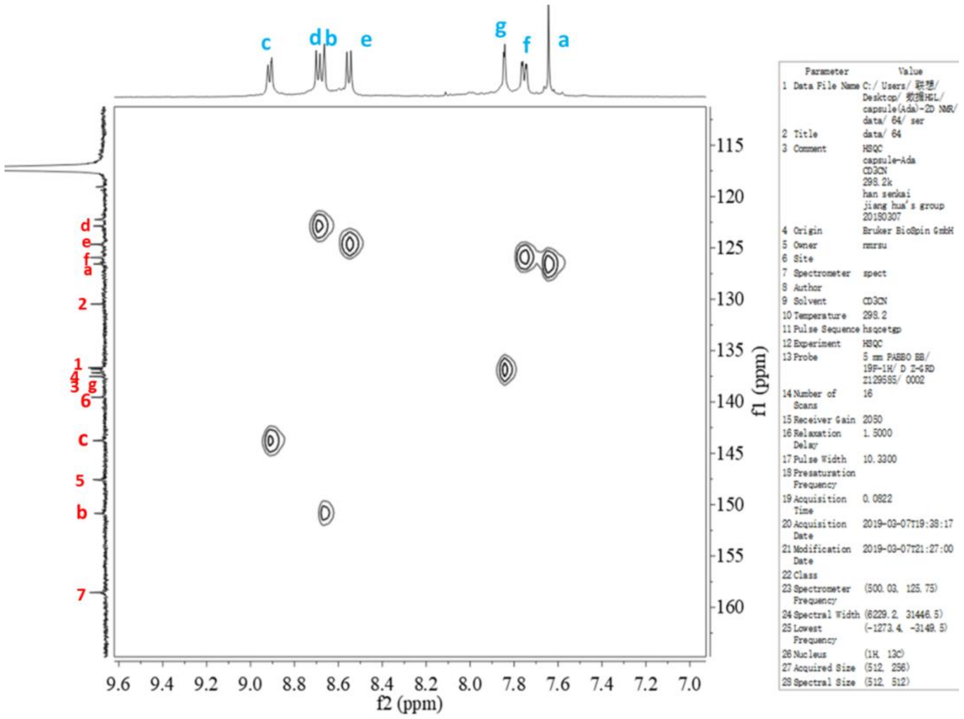

Supplementary Figure 88. (a) ^{1}H–^{13}C HSQC spectrum (500 MHz, 298 K, CD_3CN) of **Ad**⊂$[Hg_5\mathbf{1}_2]\cdot[OTf]_{10}$ (5 mM). (b) Zoom of spectrum (a).

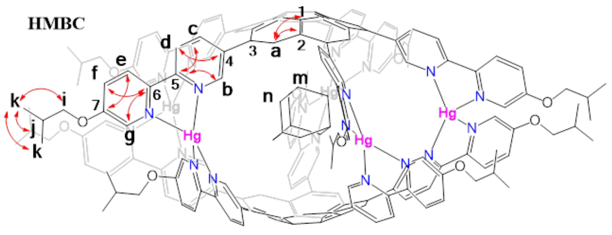

(a)

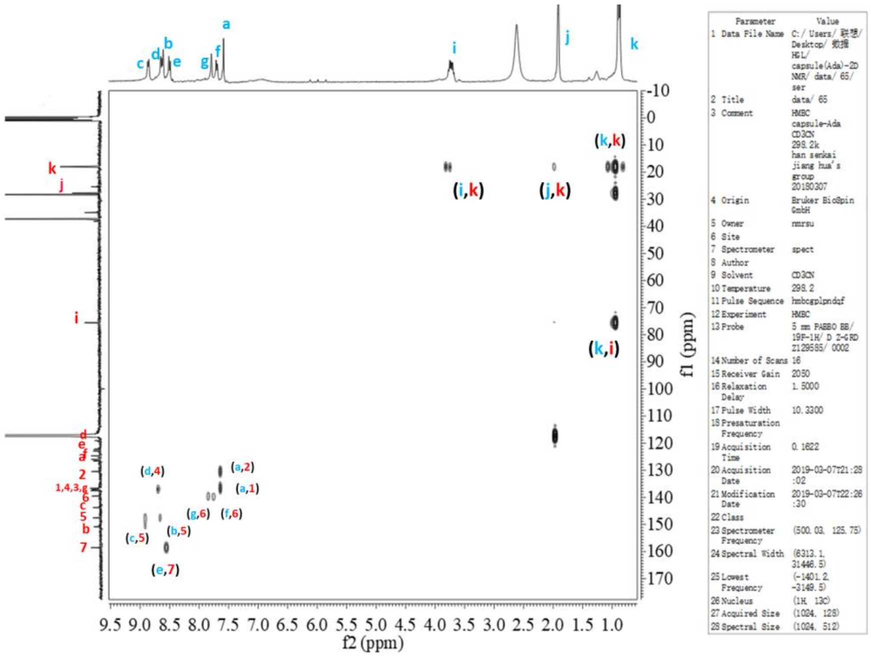

(b)

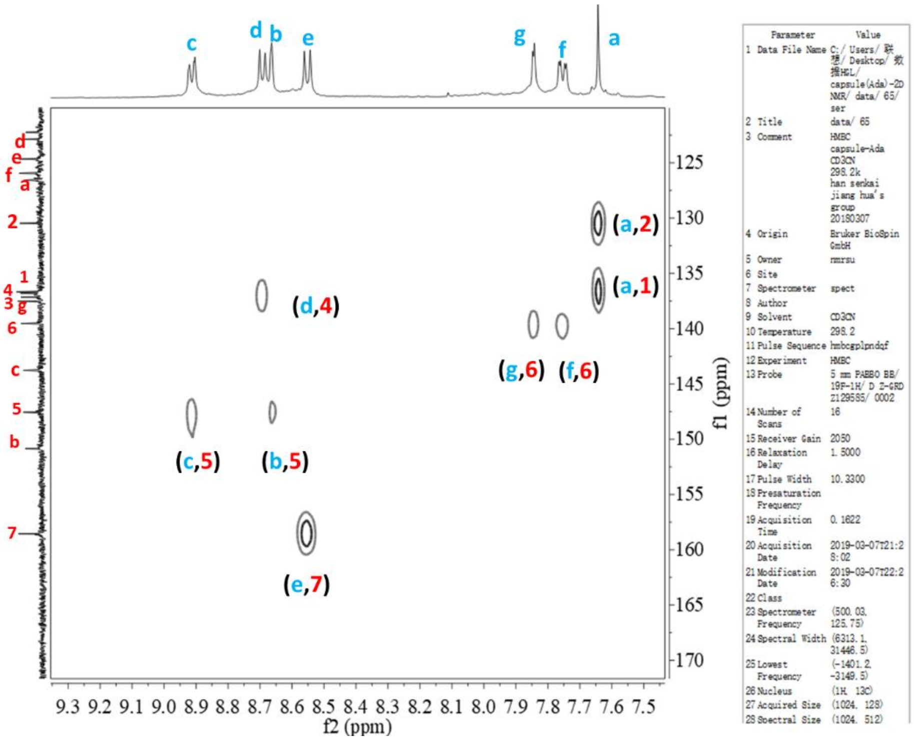

Supplementary Figure 89. ^1H–^{13}C HMBC spectrum (500 MHz, 298 K, CD_3CN) of **Ad**⊂$[Hg_5\mathbf{1}_2]\cdot[OTf]_{10}$ (5 mM). (b) Zoom of spectrum (a).

NOESY

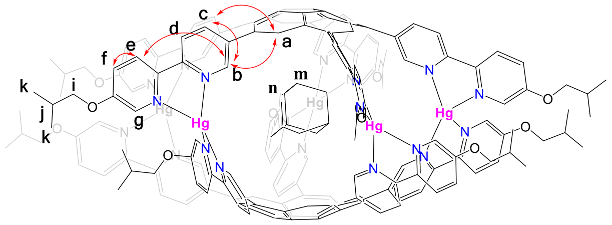

(a)

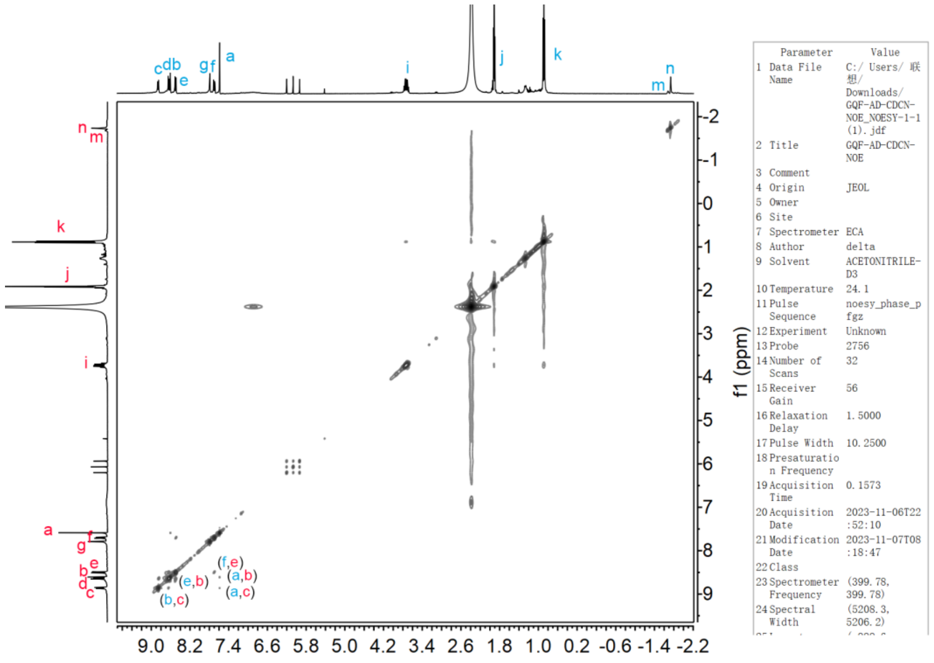

(b)

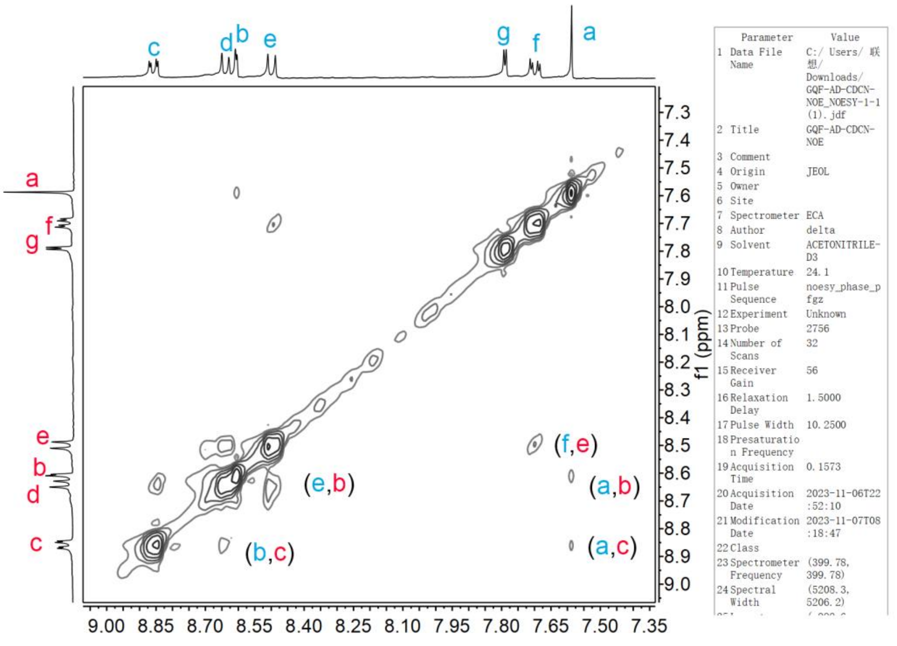

Supplementary Figure 90. ^1H–^1H NOESY spectrum (400 MHz, 298 K, CD_3CN) of **Ad**⊂$[Hg_5\mathbf{1}_2]\cdot[OTf]_{10}$ (5 mM). (b) Zoom of spectrum (a).

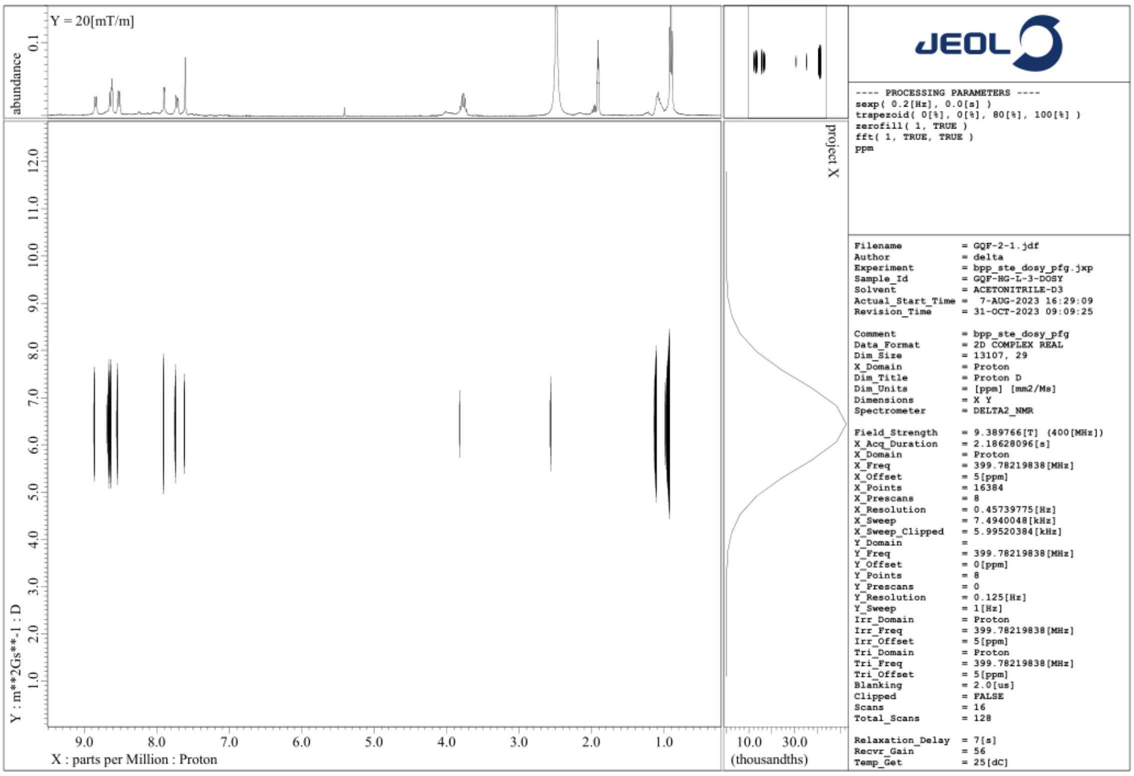

Supplementary Figure 91. Part of the DOSY spectrum (400 MHz, 298 K) of **$[Hg_51_2]\cdot[OTf]_{10}$** (5mM) in CD_3CN.

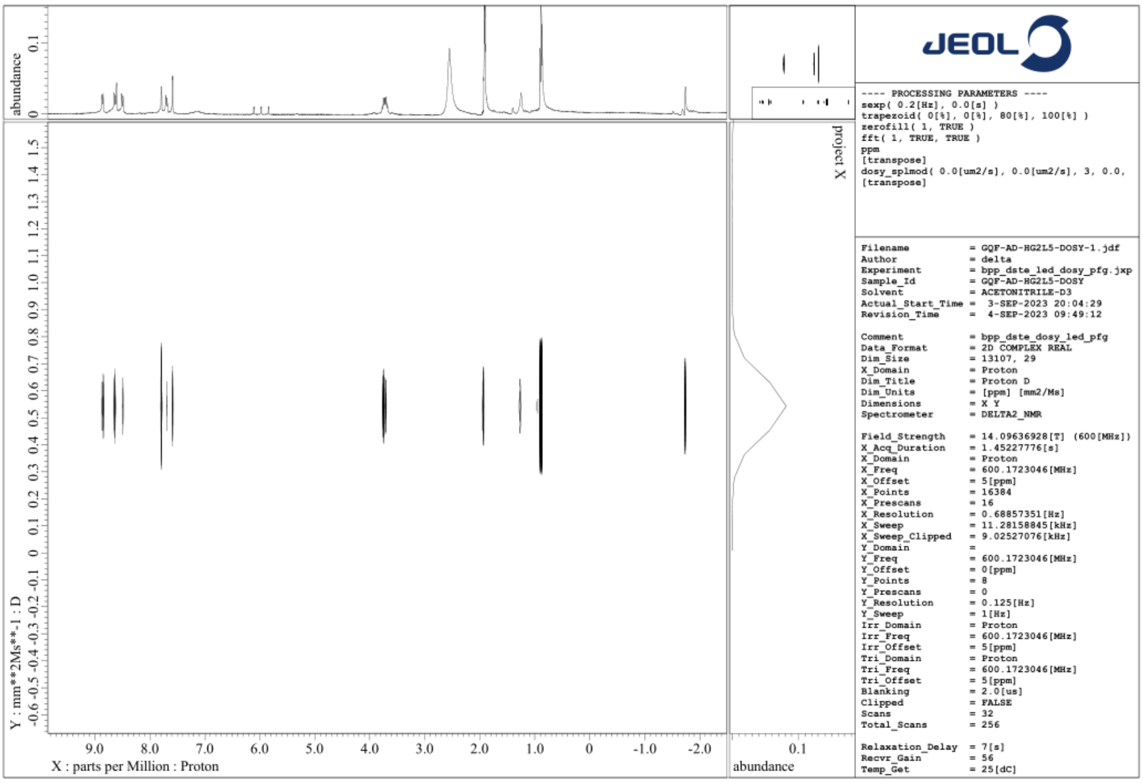

Supplementary Figure 92. Part of the DOSY spectrum (400 MHz, 298 K) of **Ad** ⊂ **$[Hg_51_2]\cdot[OTf]_{10}$** (5mM) in CD_3CN.

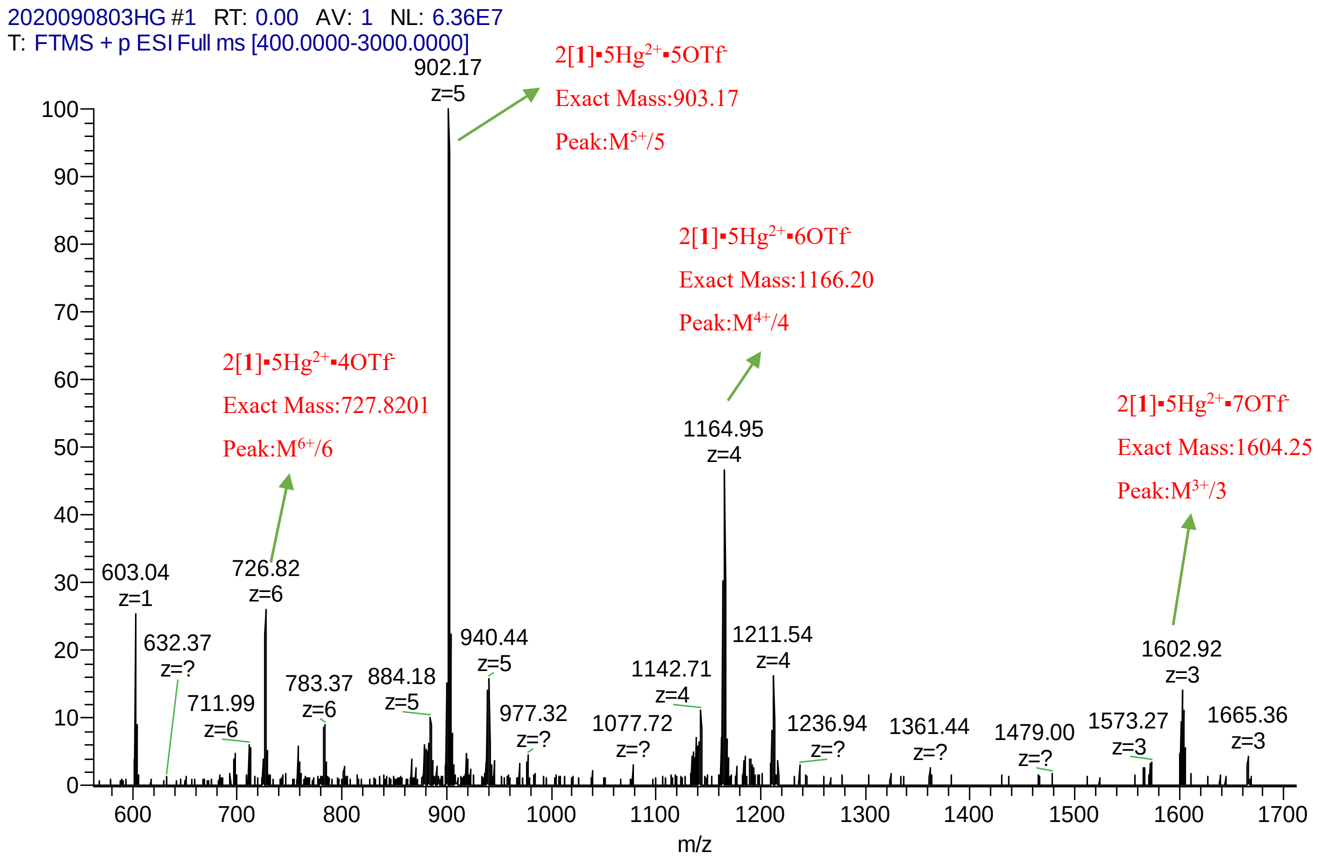

Supplementary Figure 93. ESI-MS spectrum of the cage $[Hg_5\mathbf{1}_2]\cdot[OTf]_5$. The experimental and simulated expanded isotopic clusters of some important species as well as the summary of the data are shown in Supplementary Table 12.

Supplementary Table 12. Summary of HRMS-ESI Data and the Expanded Isotopic Clusters for $[Hg_5\mathbf{1}_2]\cdot[OTf]_5$.

Species	Charge	Spectrum patterns	Chemical Formula	Data
2[**L**]·5Hg^{2+}·4OTf^-	+6	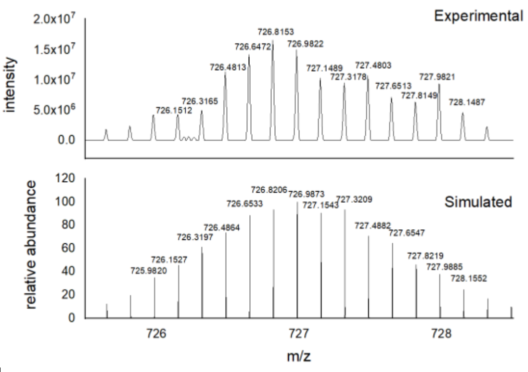	$C_{184}H_{160}N_{20}O_{22}F_{12}S_4$ Hg_5 at M^{6+}	Found: 726.8153 (100%, -7.3ppm); 726.9822 (90.3%, -7.0 ppm); 726.6472 (85.6%, -8.4ppm); 726.4813 (68.2%, -7.0ppm); 727.4803 (64.8%, -10.9 ppm); 727.1489 (62.4% -7.4ppm); 727.3178 (57.5%, -4.3 ppm); 727.9821 (56.8%, -8.8 ppm); 727.6513(43.0%, -4.7ppm); 727.8149(38.3%, -9.6ppm).

<table>
<tr><td>2[L]•5Hg²⁺•5OTf⁻</td><td>+5</td><td>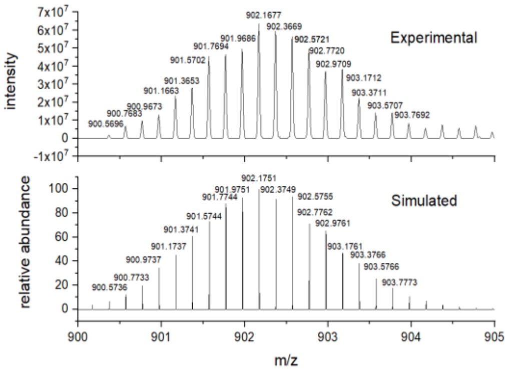</td><td>$C_{185}H_{160}N_{20}O_{25}F_{15}S_5$
Hg_5 at M^{5+}</td><td>Found: 902.1677 (100%, -8.2ppm);
902.3669 (93.8%, -8.9 ppm);
902.5721 (90.0%, -3.8 ppm);
901.9686 (78.0%, -7.2ppm);
902.7720(77.8%, -4.7ppm);
901.7694 (73.2%, -5.5 ppm);
901.5702 (71.7%, -4.7 ppm);
903.1712(60.5% ， -5.4ppm);
902.9709 (58.6%, -5.8 ppm).
901.3653(43.7%, -9.8ppm)
901.1663(37.0%, -8.2ppm)
903.3711(35.3%, -6.1ppm)
903.5707(22.0%, -6.5ppm)
900.9673(20.6%, -7.1ppm)</td></tr>
<tr><td>2[L]•5Hg²⁺•6OTf⁻</td><td>+4</td><td>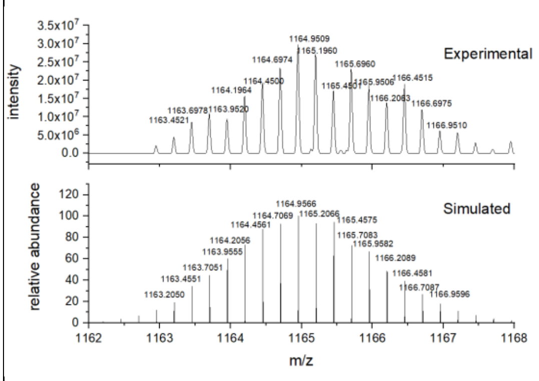</td><td>$C_{186}H_{160}N_{20}O_{28}F_{18}S_6$
Hg_5 at M^{4+}</td><td>Found: 1164.9509(100%, -4.9ppm);
1165.1960(91.4%, -9.1ppm);
1165.6960(77.8%，-10.6ppm);
1164.6974(78.9%, -8.2ppm);
1164.4500(64.8%, -5.2ppm);
1165.9506(62.9%, -6.5ppm);
1166.4515(63.6%, -5.7ppm);
1164.1964(52.9%, -7.9ppm);
1166.2063(45.3%, -2.2ppm);
1166.6975（40.1%，-9.6ppm）;
1163.6978(37.1%, -6.3ppm);
1163.9520(31.7%, -3.0ppm)</td></tr>
<tr><td>2[L]•5Hg²⁺•7OTf⁻</td><td>+3</td><td>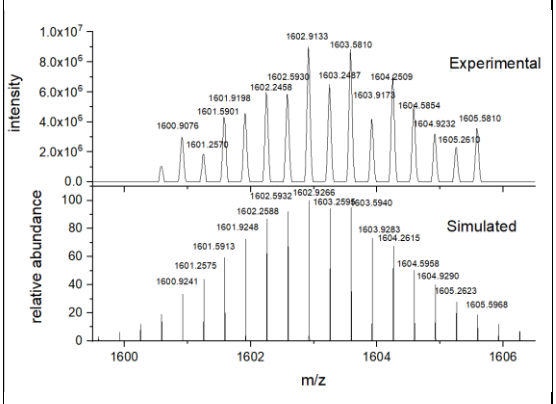</td><td>$C_{187}H_{160}N_{20}O_{31}F_{21}S_7$
Hg_5 at M^{3+}</td><td>Found: 1602.9133(100%, -8.3 ppm);
1603.5810 (98.3%, -8.1ppm);
1604.2509(79.5%, -6.6ppm);
1603.2487(72.1%, -6.7 ppm);
1602.2458 (66.4%, -8.1ppm);
1604.5854(57.3%, -6.5 ppm);
1601.9198 (50.7% ， -3.1 ppm);
1601.5901(46.6%, -0.7ppm)
1605.5810(37.6%, -9.8 ppm).
1604.9232(35.6%, -3.6ppm)</td></tr>
</table>

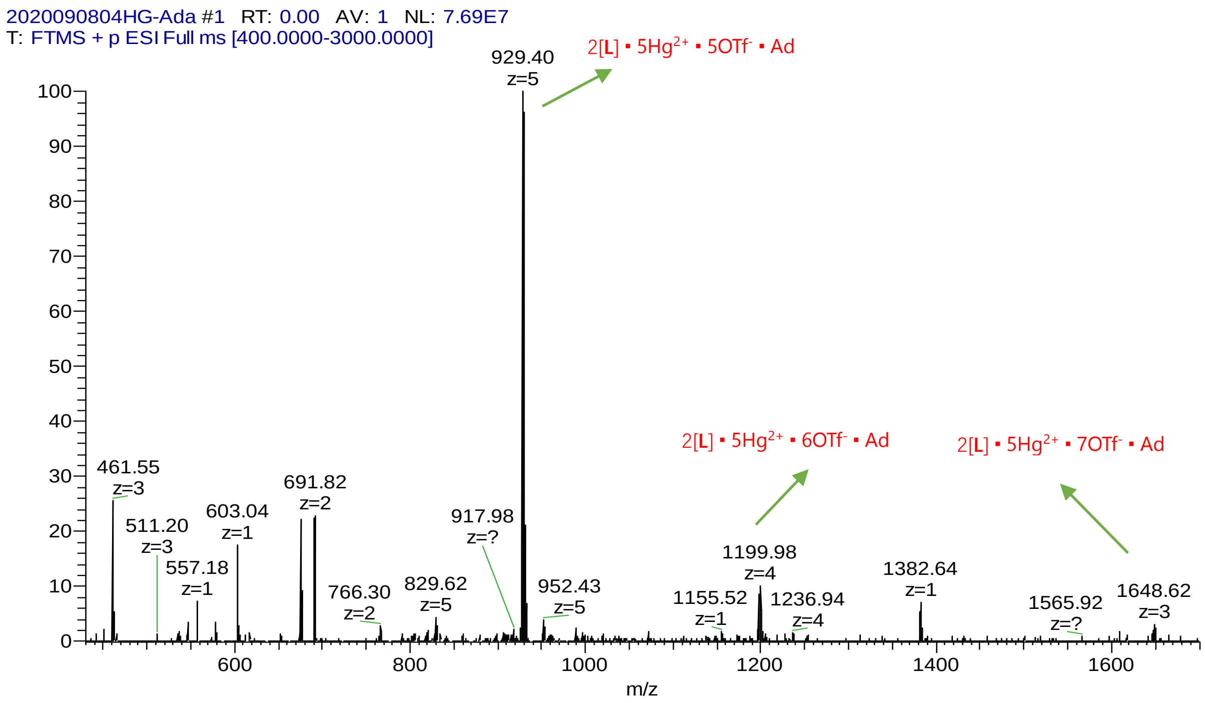

Supplementary Figure 94. ESI-MS spectrum of **Ad**⊂[Hg_5**1**$_2$]·$[OTf]_{10}$. The experimental and simulated expanded isotopic clusters of some important species as well as the summary of the data are shown in Supplementary Table 13.

Supplementary Table 13. Summary of the HRMS-ESI Data and the Expanded Isotopic Clusters for **Ad**⊂ [Hg_5**1**$_2$]·$[OTf]_{10}$.

Species	Charge	Spectrum patterns	Chemical Formula	Data
2[**L**]·$5Hg^{2+}$·$5OTf^-$·Ad	+5	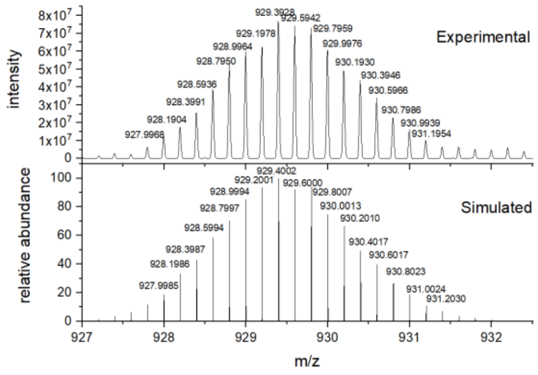	$C_{195}H_{176}N_{20}O_{25}F_{15}S_5$ Hg_5 at M^{5+}	Found: 929.3928 (100%, -8.0ppm); 929.7959 (94.8%, -5.2ppm); 929.5942 (96.2%，-6.2 ppm); 929.1978 (81.1%, -2.5ppm); 929.9976 (79.0%，-4.0ppm); 928.9964 (77.6%, -3.2ppm); 928.7950 (68.7%, -5.1ppm); 930.1930 (64.0%，-8.6 ppm); 930.3946 (56.9%, -7.6ppm); 928.5936 (50.3%, -6.2ppm); 930.5966 (44.0%, -5.5ppm); 928.3991 (32.9%, 0.4ppm); 930.7986 (29.3%, -4.0 ppm）; 928.1904 (21.7%, -8.8 ppm)

2[**L**]•5Hg^{2+}•6OTf^{-}•Ad	+4	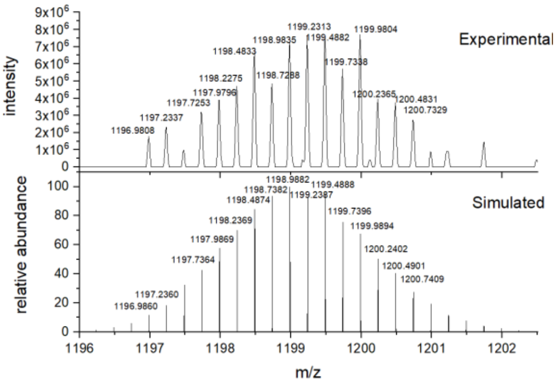	$C_{196}H_{176}N_{20}O_{28}F_{18}S_6$ Hg_5 at M^{4+}	Found:<br>1199.9804 (100%, -7.5ppm);<br>1199.2313 (98.6%%, -6.2ppm);<br>1199.4882 (96.9%, -0.5ppm);<br>1198.9835 (93.4%, -3.9ppm);<br>1198.4833 (86.3%， -3.4ppm);<br>1199.7338 (74.2%， -4.8 ppm);<br>1198.7288 (62.9%, -7.8ppm);<br>1198.2275 (60.9%, -7.8ppm);<br>1200.2365(51.3%， -3.1ppm);<br>1200.4831 (48.6%, -5.8ppm);<br>1197.9796 (50.8%, -6.1ppm);<br>1197.7253 (41.6%, -9.3ppm);<br>1200.7329 (35.6%， -6.7 ppm);<br>1197.2337 (30.3%, -1.9ppm);<br>1196.9808 (23.1%, -4.3ppm)
2[**L**]•5Hg^{2+}•7OTf^{-}•Ad	+3	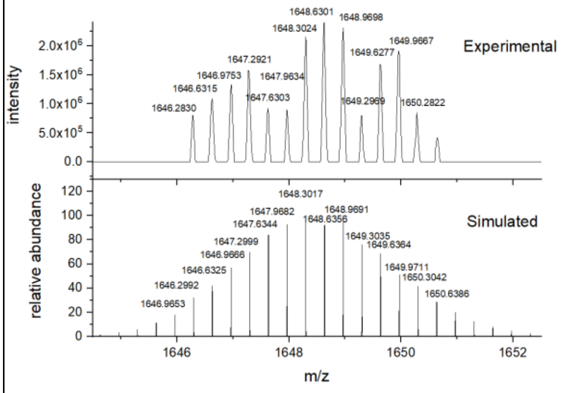	$C_{197}H_{176}N_{20}O_{31}F_{21}S_7$ Hg_5 at M^{3+}	Found: 1648.6301 (100%, -3.3ppm)；<br>1648.9698 (95.5%，0.4 ppm);<br>1648.3024 (88.9%, 0.4ppm);<br>1649.9667 (77.8%, -2.7ppm);<br>1649.6277 (69.7%， -5.3 ppm);<br>1647.2921 (65.2%， -4.7 ppm);<br>1646.9753 (55.2 %, 5.3ppm);<br>1646.6315 (42.5%, -0.6ppm); 1647.9634 (37.3%, -2.9ppm)；<br>1650.2822 (35.3%, -13.3ppm)<br>1646.2830 (33.6%, -9.8ppm);<br>1627.6303 (38.4%, -2.5ppm);<br>1649.2969 (33.2%， -4.0 ppm）；

Supplementary Method 13. Conversion From $\mathbf{Hg_5L_2}$ to $\mathbf{Ag_5L_2}$

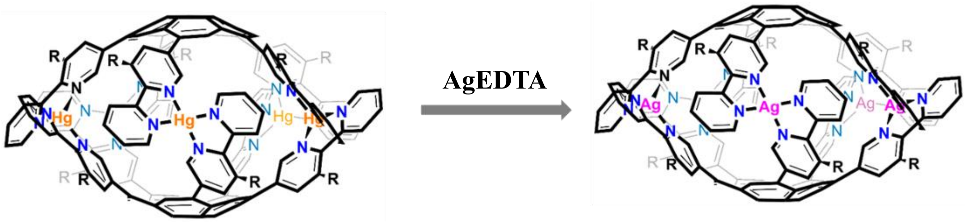

$\mathbf{Hg_5L_2}$ $\mathbf{Ag_5L_2}$

To a solution of disodium ethylenediaminetetraacetate (Na_2EDTA, 50 mM) in pure water, 1.50 equiv. of $AgNO_3$ was added to give an aqueous solution of AgEDTA. This aqueous solution (4 ml) was transferred to a vial (I.D. 25 mm, 10 ml) containing a solution of $\mathbf{Hg_5L_2}$ (0.5 mM, 4 ml) in $CDCl_2CDCl_2$ and a magnetic stir bar (olive shape, diameter 9 mm, length 15 mm) to give a bilayer mixture. The mixture was vigorously stirred (950 rpm) at 28 °C. To monitor the ion-exchange process, sample was taken regularly from the mixture. These samples were washed immediately with brine (×3) and pure water (×2), followed by drying with sodium sulfate. The series of samples were characterized by ^1H NMR spectroscopy. The obtained time-dependent ^1H NMR spectra (Supplementary Figures 95A and B) showed that cage $\mathbf{Hg_5L_2}$ successfully converted into $\mathbf{Ag_5L_2}$ in the presence of AgEDTA, through a multistep ion-exchange processes. The stepwise ion-exchange is strongly suggested by the loss of C_5-symmetry for the cage in the ^1H NMR spectrum (can be clearly observed at T = 23 and 27 min, Supplementary Figure 95B). The intermediates, naturally, in which the two types of metal ions (Ag^+ and Hg^{2+}) are coordinated to form a heterometallic cages, were evidenced by the HR ESI-MS of the sample T = 23 min (Figure 6d, Supplementary Figure 96 and Supplementary Table 14), which showed a series of intense peaks assignable to mixed-metal cages with different stoichiometries for the composition of the cooridnated Ag^+ and Hg^{2+} cations.

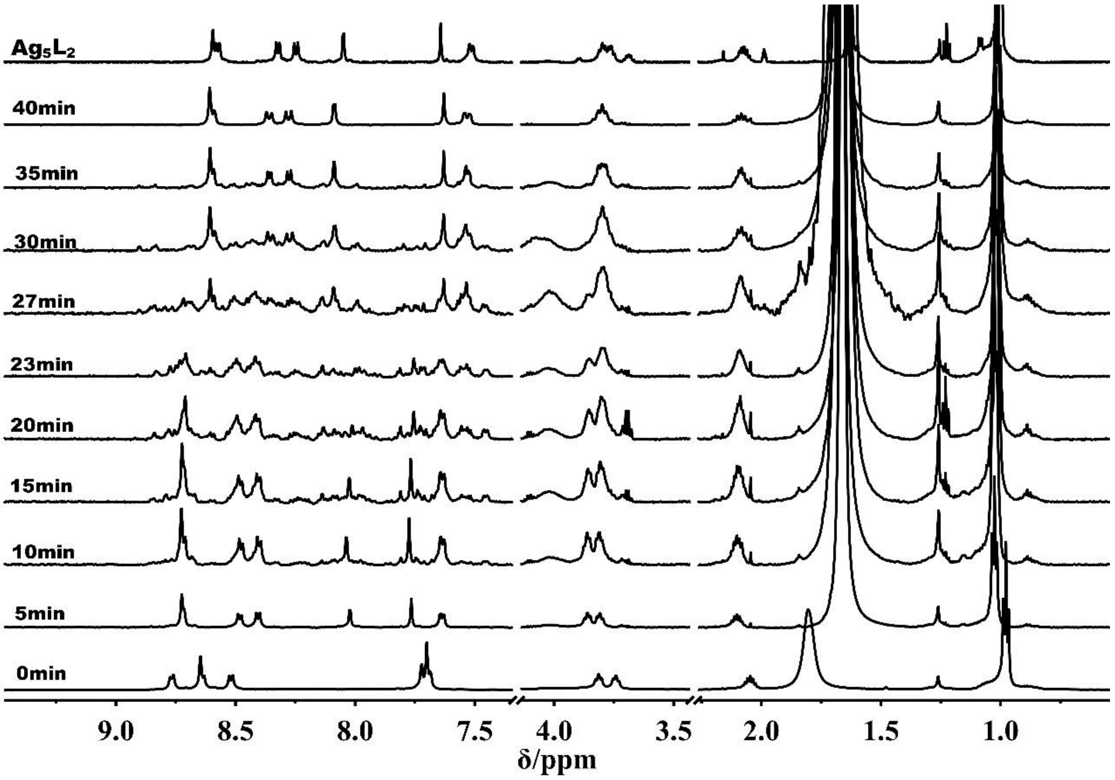

Supplementary Figure 95A. Time-depended ^{1}H NMR spectra (600 MHz, 298 K, $CDCl_2CDCl_2$) of the water-washed organic phase solution in the vigorously stirred bilayer mixture of $[Hg_5\mathbf{1}_2]\cdot[OTf]_{10}$ ($\mathbf{Hg_5L_2}$) in $CDCl_2CDCl_2$ and AgEDTA in water.

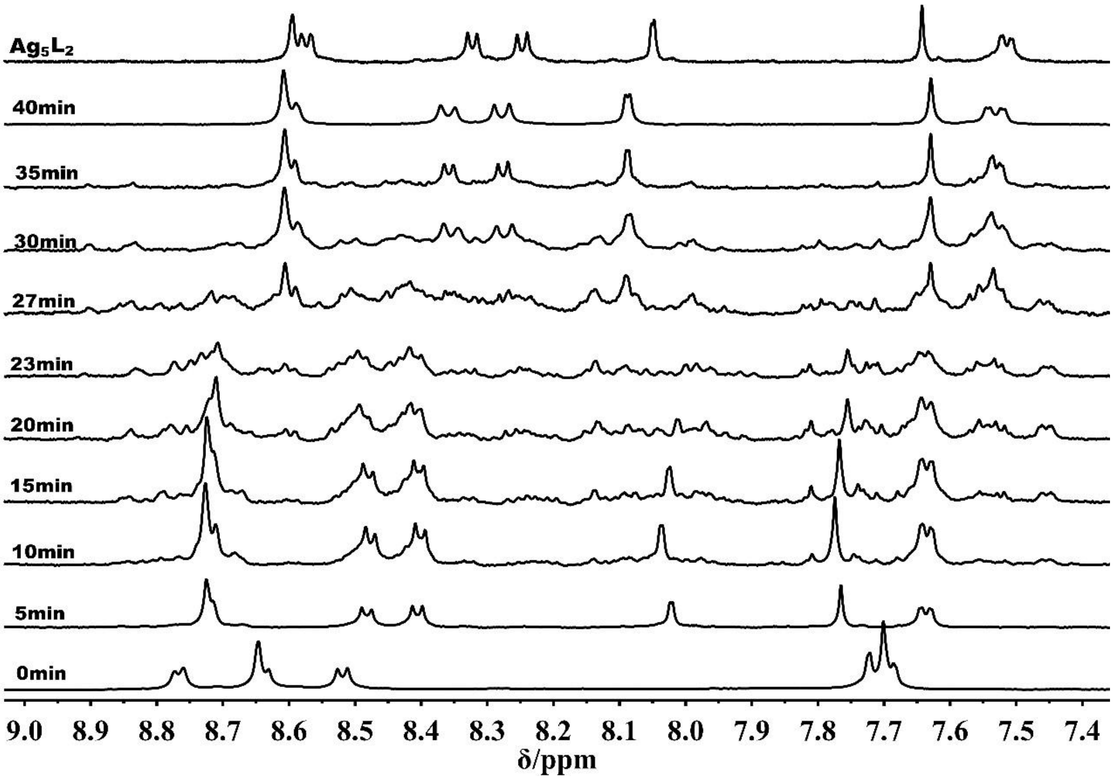

Supplementary Figure 95B. Zoom of the ^{1}H NMR spectra (600 MHz, 298 K, $CDCl_2CDCl_2$) shown in Supplementary Figure 95A, which shows that $[Hg_5\mathbf{1}_2]\cdot[OTf]_{10}$ was gradually converted to $[Ag_5\mathbf{1}_2]\cdot[OTf]_5$ ($\mathbf{Ag_5L_2}$) over time.

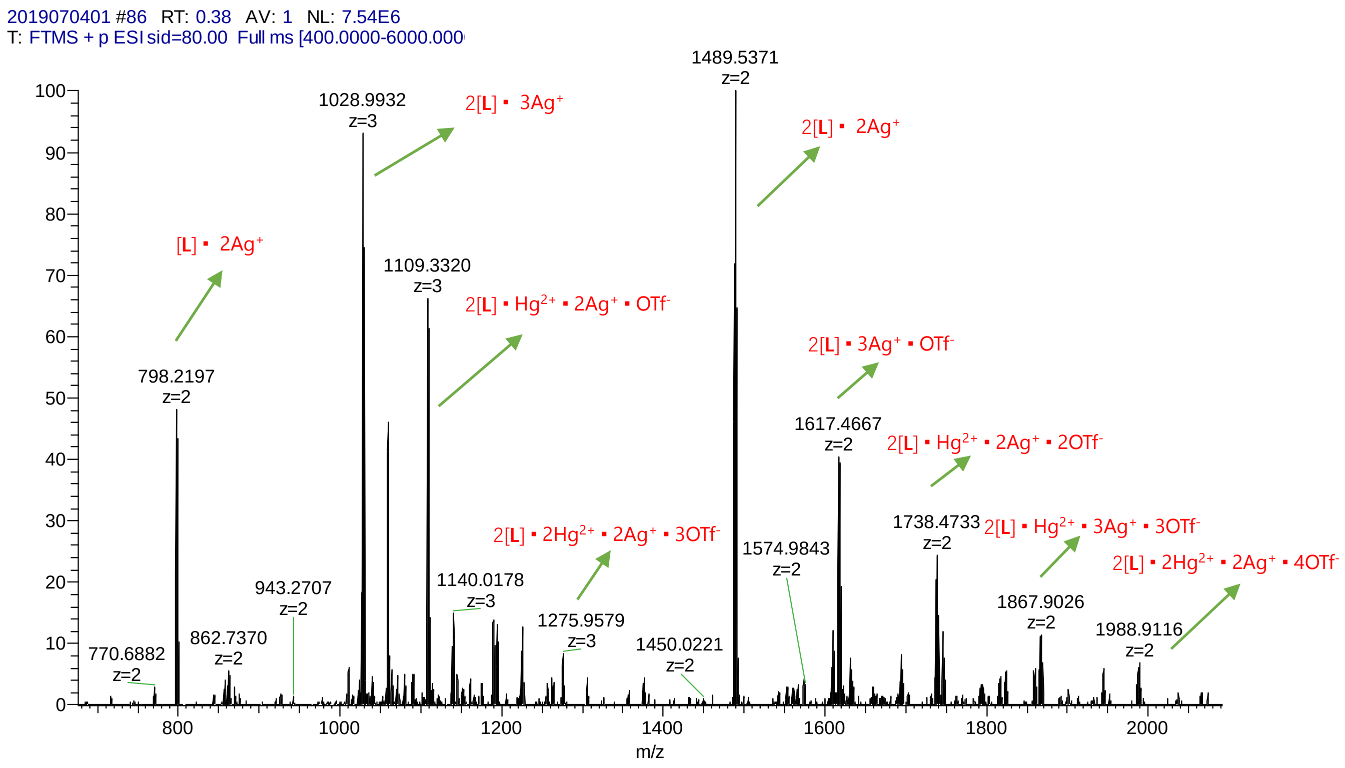

Supplementary Figure 96. ESI-MS spectrum of the sample at T = 23 min. The sample was taken from the vigorously stirred bilayer mixture of $[Hg_5\mathbf{1}_2]\cdot[OTf]_{10}$ ($\mathbf{Hg_5L_2}$) in $CDCl_2CDCl_2$ and AgEDTA in water, followed being washed immediately with water drying with sodium sulfate. The corresponding ^{1}H NMR spectrum is shown in Supplementary Figure 95. The experimental and simulated expanded isotopic clusters of some important species are shown in Supplementary Table 14.

Supplementary Table 14. Summary of the HRMS-ESI Data shown in Supplementary Figure 95 as well as the Expanded Isotopic Clusters.

Species	Charge	Spectrum patterns	Chemical Formula	Data
[**L**]·$2Ag^+$	+2	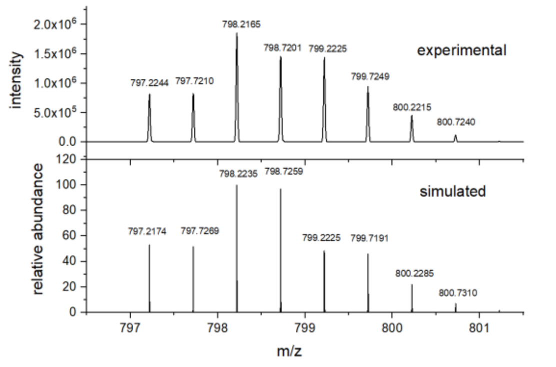	$C_{90}H_{80}N_{10}O_5Ag_2$ at M^{2+}	Found: 798.2165 (100%, -8.8 ppm); 798.7201 (78.6%, -7.3 ppm); 799.2225 (77.6%, 0 ppm); 799.7249 (51.1%, 7.3 ppm); 797.7210 (44.3%, -7.4 ppm); 797.2244 (44.1%, 8.8 ppm); 800.2215 (24.3%, -8.7 ppm); 800.7240 (6.6%, -8.7 ppm).

2[**L**]·3Ag^+	+3	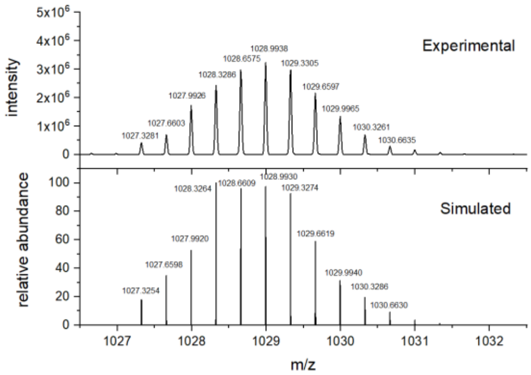	$C_{180}H_{160}N_{20}O_{10}Ag_3$ at M^{3+}	Found: 1028.9938 (100%, 0.8ppm); 1028.6575 (92.0%, -3.3 ppm); 1029.3305 (92.0%, 3.0 ppm); 1028.3286 (75.3%, 2.1 ppm); 1029.6597 (66.8%, -2.1 ppm); 1027.9926 (53.4%, 0.6 ppm); 1029.9965 (41.3% ， 2.4 ppm); 1030.3261 (21.5%, -2.4 ppm).
2[**L**]·Hg^{2+}·2Ag^+·OTf⁻	+3	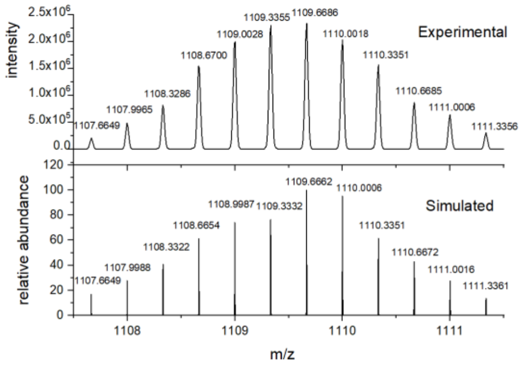	$C_{181}H_{160}N_{20}O_{13}F_3SAg_2Hg$ at M^{3+}	Found: 1109.6686 (100%, 2.2ppm); 1109.3355 (97.9%, 2.1ppm); 1110.0018 (86.6%, 1.1ppm); 1110.3351 (66.8%, 0 ppm); 1108.6700 (65.9%, 4.1 ppm); 1110.6685 (37.1%,1.2 ppm); 1108.3286 (34.7% ， -3.2 ppm); 1107.9965 (20.5%, -2.1 ppm) 1111.0006(12.8%, -0.9 ppm). 1111.3356 (12.8%, -0.4ppm) 1107.6649 (8.7%,0ppm)
2[**L**]·2Hg^{2+}·2Ag^+·3OTf⁻	+3	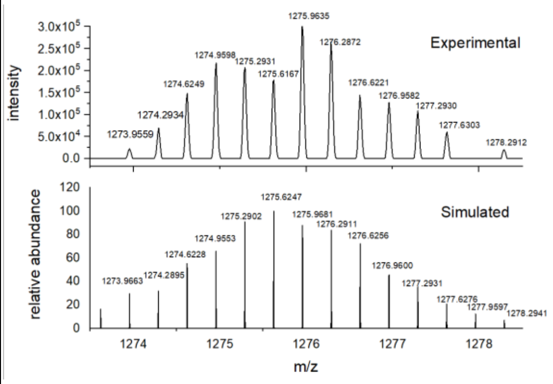	$C_{183}H_{160}N_{20}O_{19}F_9S_3Hg_2Ag_2$ at M^{3+}	Found: 1275.9635 (100%, -3.6 ppm); 1276.2872 (92.6%, -3.1ppm); 1274.9598 (76.1%, 3.5 ppm); 1275.2931 (72.4%, 2.3 ppm);1275.6167 (61.3%, -6.3ppm); 1274.6249 (51.8%,1.1.6 ppm); 1276.6221 (50.4%, -2.7 ppm); 1276.9582 (44.8%, -1.4 ppm) 1277.2930(38.0%, -0.08 ppm). 1277.6303 (21.3%, 2.1 ppm) 1274.2934 (24.2%, 3.1 ppm)

2[**L**]·2Ag^{+}	+2	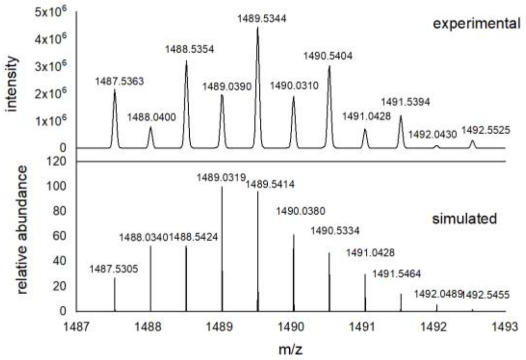	$C_{180}H_{160}N_{20}O_{10}Ag_2$ at M^{2+}	Found: 1489.5344 (100%, -4.7ppm); 1488.5354 (71.7%, -4.7ppm); 1490.5404 (67.7%,4.7ppm); 1487.5363(48.5%, 3.9 ppm);1489.0390 (43.7%,4.8ppm); 1490.0310 (45.3%,-4.7 ppm); 1491.5394 (27.8%, -4.7 ppm); 1488.0400 (19.0%, 4.0 ppm) 1491.0428(16.2%, 0 ppm).
2[**L**]·3Ag^{+}·OTf$^{–}$	+2	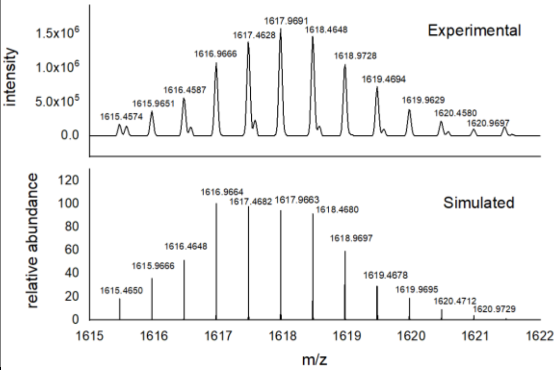	$C_{181}H_{160}N_{20}O_{13}F_3SAg_3$ at M^{2+}	Found: 1617.9691 (100%, 1.7ppm); 1618.4648 (93.3%, -2.0 ppm); 1617.4628 (87.8%, -3.3 ppm); 1618.9728 (66.6%, 1.9 ppm); 1619.4694 (46.0%, 1.0ppm); 1616.4587 (35.5%, -3.8 ppm); 1619.9629(24.5% ， -4.1 ppm); 1615.9651(23.3%,-1.0 ppm) 1620.4580 (13.5%, -8.1ppm); 1651.4574 (10.6%, -4.6ppm); 1620.9697(6.3%, -2.0 ppm)
2[**L**]·Hg^{2+}·2Ag^{+}·2OTf$^{–}$	+2	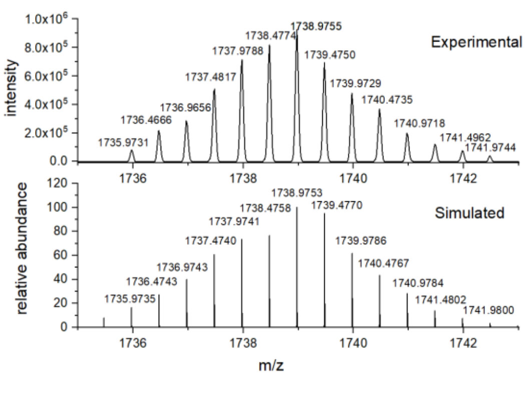	$C_{182}H_{160}N_{20}O_{16}F_6S_2HgAg_2$ at M^{2+}	Found: 1738.9755 (100%, 0.1 ppm); 1738.4774 (88.5%, 0.9 ppm); 1737.9788 (77.2%, 2.7 ppm); 1739.4750 (74.9%, -1.1 ppm); 1737.4817 (54.9%, 4.4 ppm); 1739.9729 (51.9%, -3.3ppm); 1740.4735 (39.8%, -1.8 ppm);1736.9656 (30.8%, -5.0ppm). 1740.9718 (21.5%, -3.8ppm); 1736.4666 (23.4%, -4.4 ppm)

2[**L**]·Hg^{2+}·$3Ag^{+}$·3OTf⁻	+2	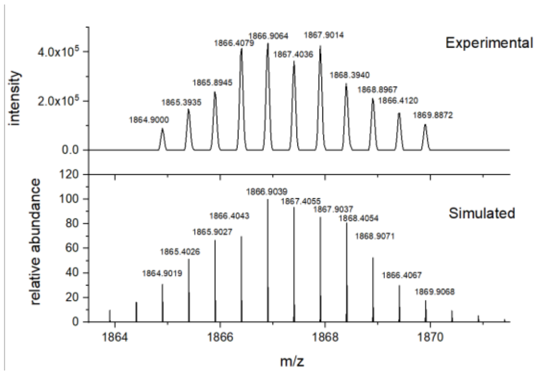	$C_{183}H_{160}N_{20}O_{19}F_9S_3Hg$ Ag_3 at M^{2+}	Found: 1866.9064 (100%, 1.3ppm); 1866.4079 (95.1%, 1.9ppm); 1867.9014 (97.5%, -1.2ppm); 1867.4036 (83.2%, -1.0 ppm); 1868.3940 (62.4%, -6.1ppm); 1865.8945 (54.6%, -4.4ppm); 1868.8967 (48.4%,-5.6 ppm) 1866.4120 (34.6%, 2.8ppm). 1865.3935 (38.4%, -4.9ppm) 1869.8872 (24%, -10.5ppm) 1864.9000(20.4%， -1.0ppm)

Supplementary Method 14. Conversion From **Ag_5L_2** to **Hg_5L_2**

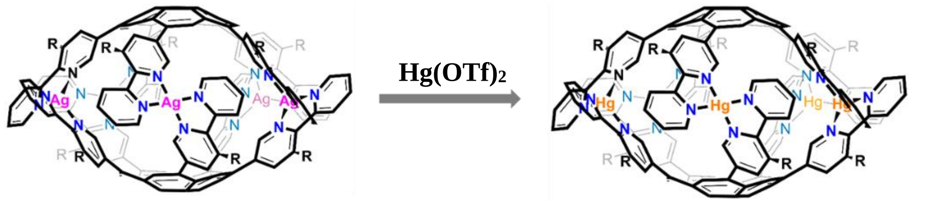

Ag_5L_2 **Hg_5L_2**

Conversion from **Ag_5L_2** to **Hg_5L_2** can be readily achieved by adding Hg^{2+} cations to the solution of **Ag_5L_2**. To $[Ag_5\mathbf{1}_2]\cdot[OTf]_5$ (1 mM) in $CDCl_2CDCl_2$, $Hg(OTf)_2$ (5.0 equiv., $CDCl_2CDCl_2$) was added. The mixture was shaken by hand for 3~5 minutes. ^1H NMR spectra of the sample before and after the conversion are shown in Supplementary Figure 97, in which the spectrum of the sample after the conversion is almost identical to the intrinsic one of $[Hg_5\mathbf{1}_2]\cdot[OTf]_5$ in $CDCl_2CDCl_2$ (Supplementary Figure 97 and Figure 6b in the main text).

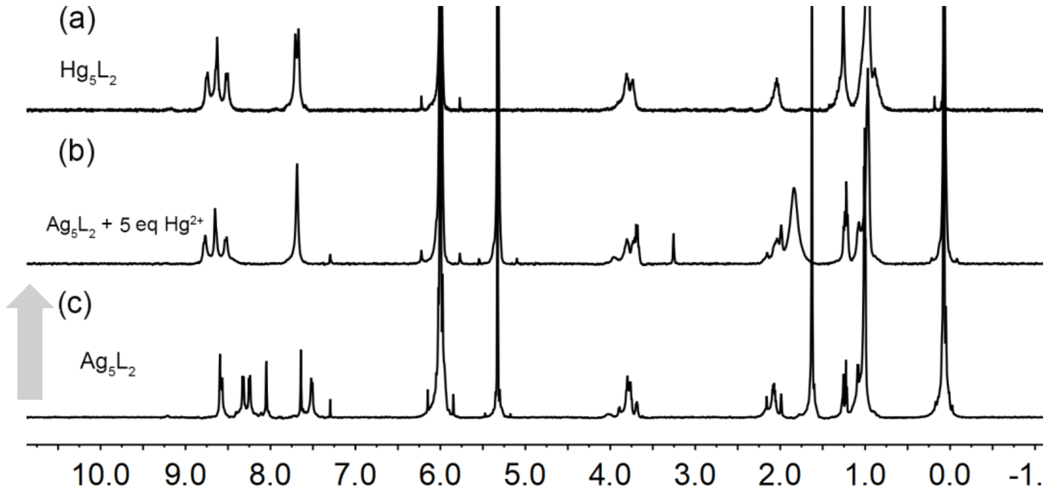

Supplementary Figure 97. ^1H NMR spectra (600 MHz, 298 K, $CDCl_2CDCl_2$) of (a) $[Hg_5\mathbf{1}_2]\cdot[OTf]_5$, (b) $[Ag_5\mathbf{1}_2]\cdot[OTf]_5$ in the presence of 5.0 equiv of $Hg(OTf)_2$, and (c) $[Ag_5\mathbf{1}_2]\cdot[OTf]_5$.

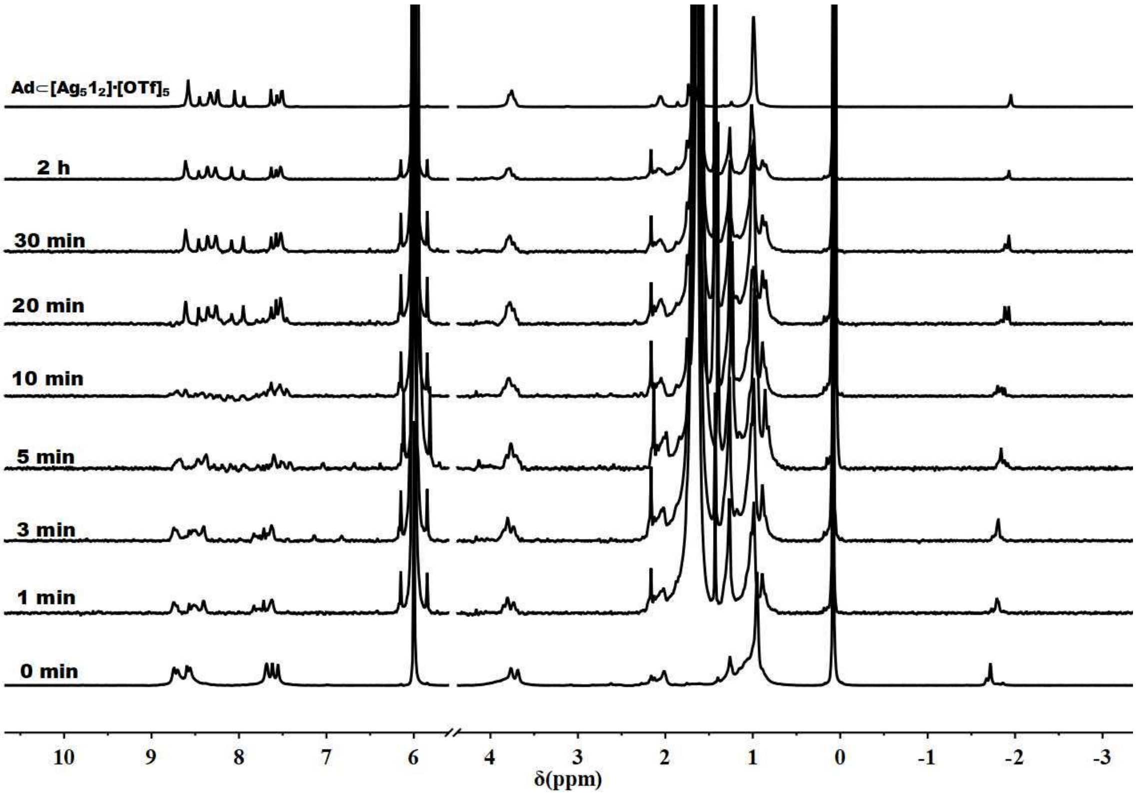

Supplementary Figure 98A. Time-depended ^{1}H NMR spectra (600 MHz, 298 K, $CDCl_2CDCl_2$) of the water-washed organic phase solution in the vigorously stirred bilayer mixture of **Ad**⊂[Hg_5**1**$_2$]·$[OTf]_{10}$ (**Ad⊂Hg_5L_2**) in $CDCl_2CDCl_2$ and AgEDTA in water. The spectra were recorded soon after the samples were sucked out of the bilayer mixture and washed with water. For comparison, ^{1}H NMR spectrum of **Ad**⊂[Ag_5**1**$_2$]·$[OTf]_5$ in $CDCl_2CDCl_2$ is shown.

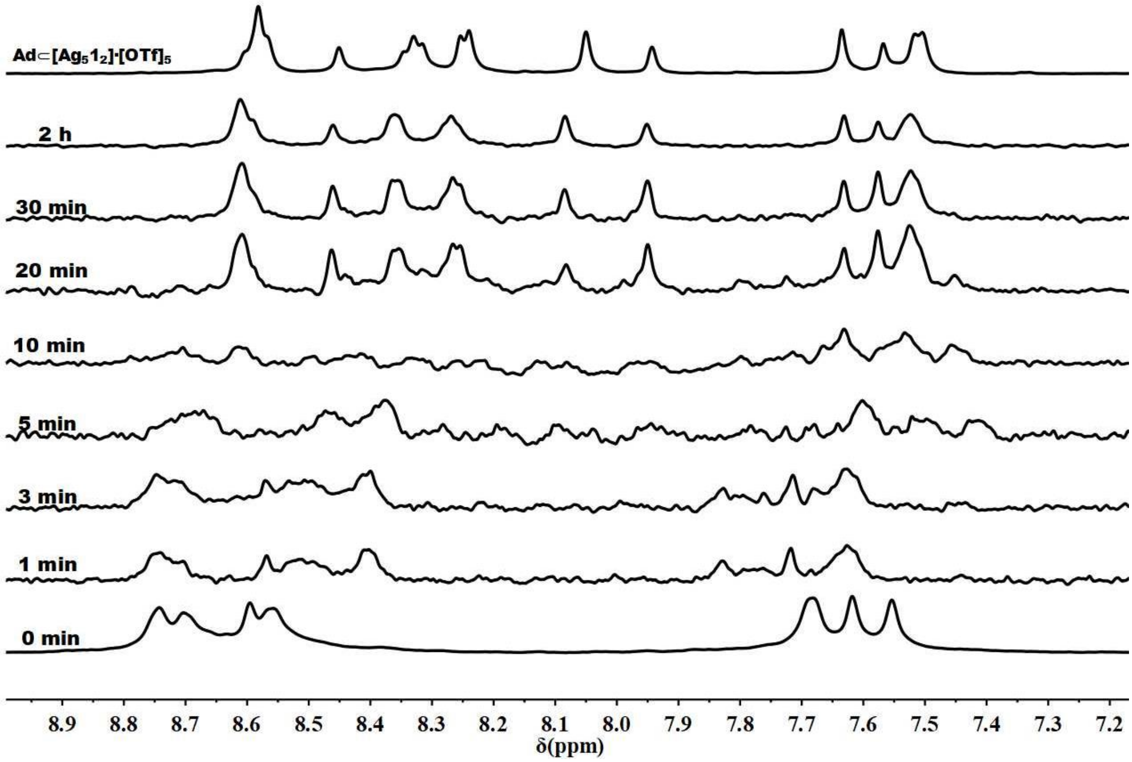

Supplementary Figure 98B. Zoom of the ^{1}H NMR spectra 97A, which shows that **Ad**⊂[Hg_5**1**$_2$]·$[OTf]_{10}$ is gradually converted to **Ad**⊂[Ag_5**1**$_2$]·$[OTf]_5$ over time.

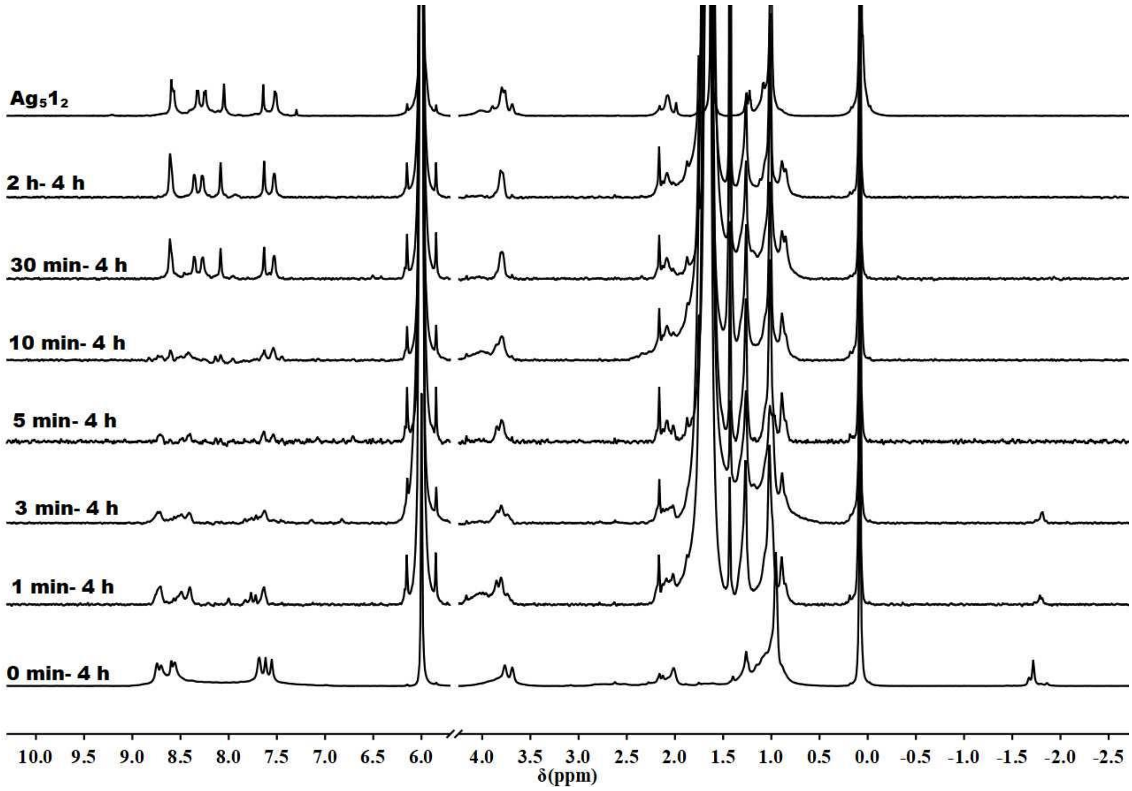

Supplementary Figure 99A. Time-depended ^{1}H NMR spectra (600 MHz, 298 K, $CDCl_2CDCl_2$) of the water-washed organic phase solution in the vigorously stirred bilayer mixture of **Ad**⊂[Hg_5**1**$_2$]·[OTf]$_{10}$ (**Ad⊂Hg$_5$L$_2$**) in $CDCl_2CDCl_2$ and AgEDTA in water. The spectra were recorded after the samples were sucked out of the bilayer mixture, washed with water and stored further at 28 °C for 4 hours. For comparison, ^{1}H NMR spectrum of [Ag_5**1**$_2$]·[OTf]$_5$ in $CDCl_2CDCl_2$ is shown.

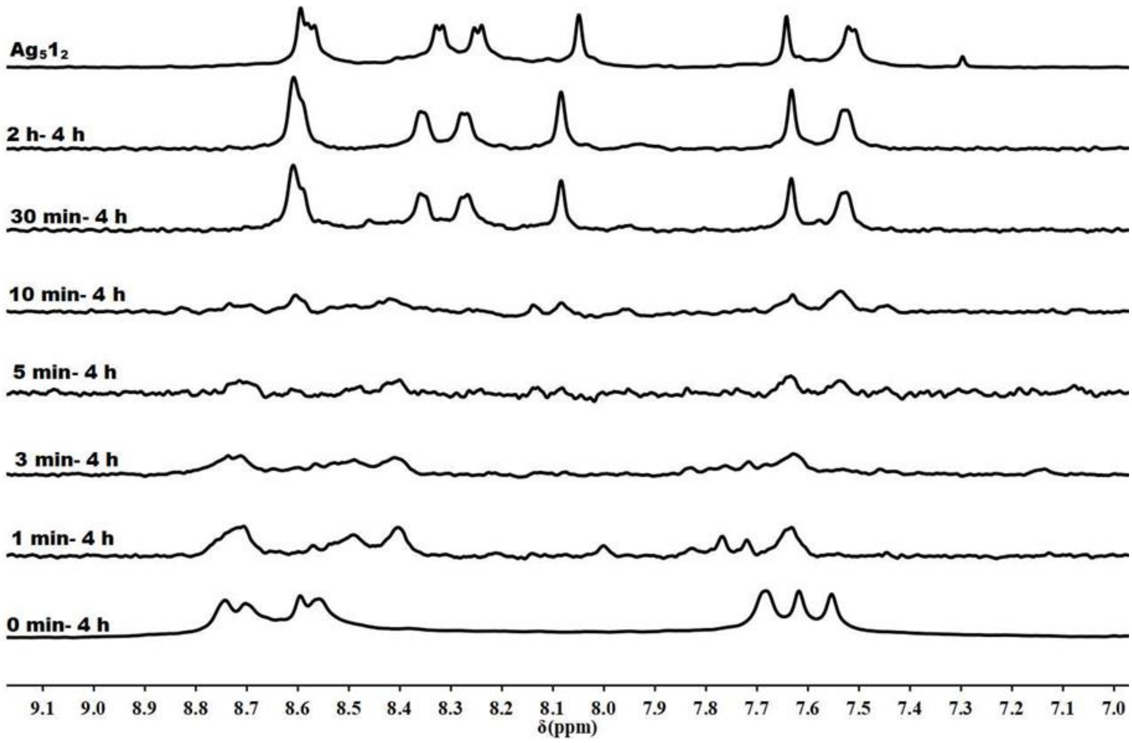

Supplementary Figure 99B. Zoom of the ^{1}H NMR spectra 98A, which shows that **Ad**⊂[Hg_5**1**$_2$]·[OTf]$_{10}$ (**Ad⊂Hg$_5$L$_2$**) was gradually converted to [Ag_5**1**$_2$]·[OTf]$_5$ (**Ag$_5$L$_2$**) with **Ad** released over time.

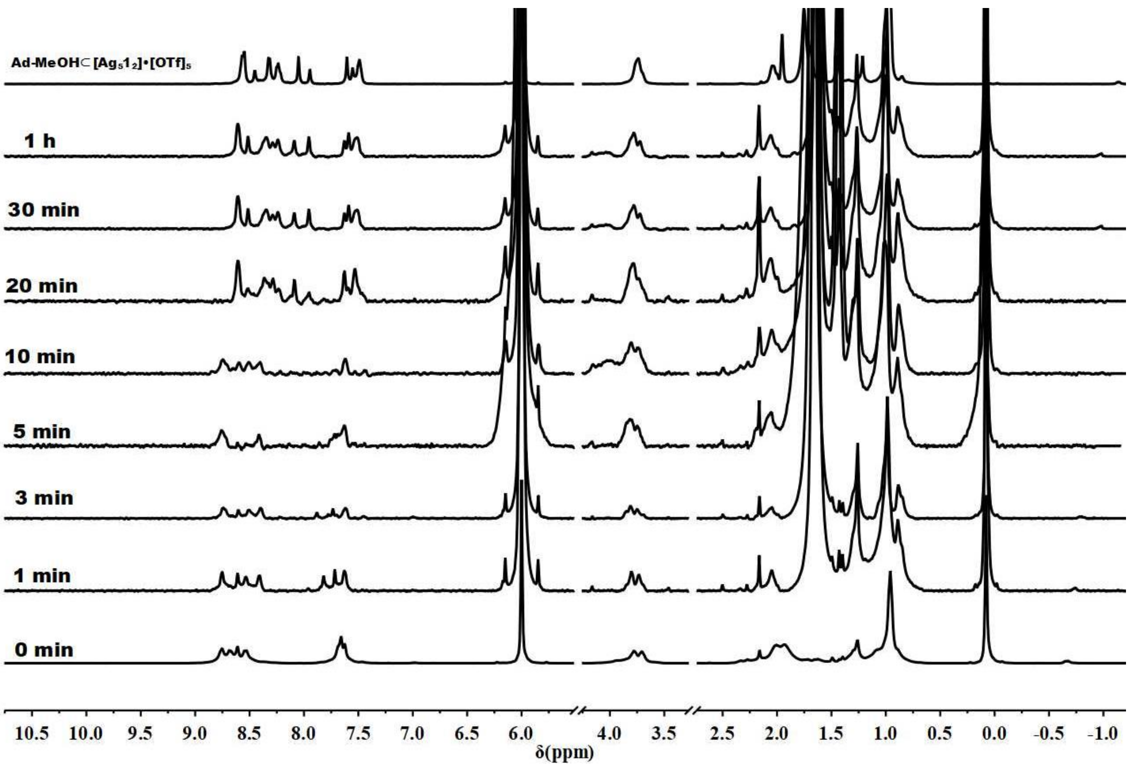

Supplementary Figure 100A. Time-depended ^{1}H NMR spectra (600 MHz, 298 K, $CDCl_2CDCl_2$) of the water-washed organic phase solution in the vigorously stirred bilayer mixture of **Ad-MeOH⊂** $[Hg_5\mathbf{1}_2]\cdot[OTf]_{10}$ (**Ad-MeOH⊂Hg$_5$L$_2$**) in $CDCl_2CDCl_2$ and AgEDTA in water. The spectra were recorded soon after the samples were sucked out of the bilayer mixture and washed with water. For comparison, ^{1}H NMR spectrum of **Ad-MOH**$\subset[Ag_5\mathbf{1}_2]\cdot[OTf]_5$ in $CDCl_2CDCl_2$ is shown.

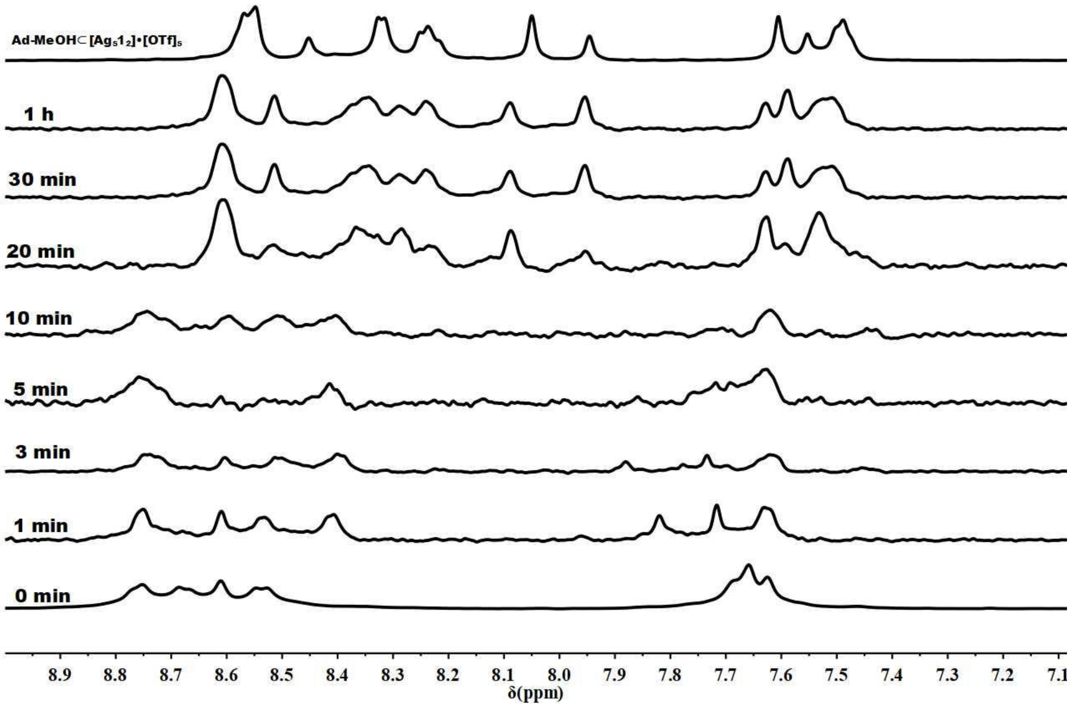

Supplementary Figure 100B. Zoom of the ^{1}H NMR spectra 99A, which shows that **Ad-MeOH**$\subset[Hg_5\mathbf{1}_2]\cdot[OTf]_{10}$ (**Ad-MeOH⊂Hg$_5$L$_2$**) was gradually converted to **Ad-MeOH**$\subset[Ag_5\mathbf{1}_2]\cdot[OTf]_5$ (**Ad-MeOH⊂Ag$_5$L$_2$**) over time.

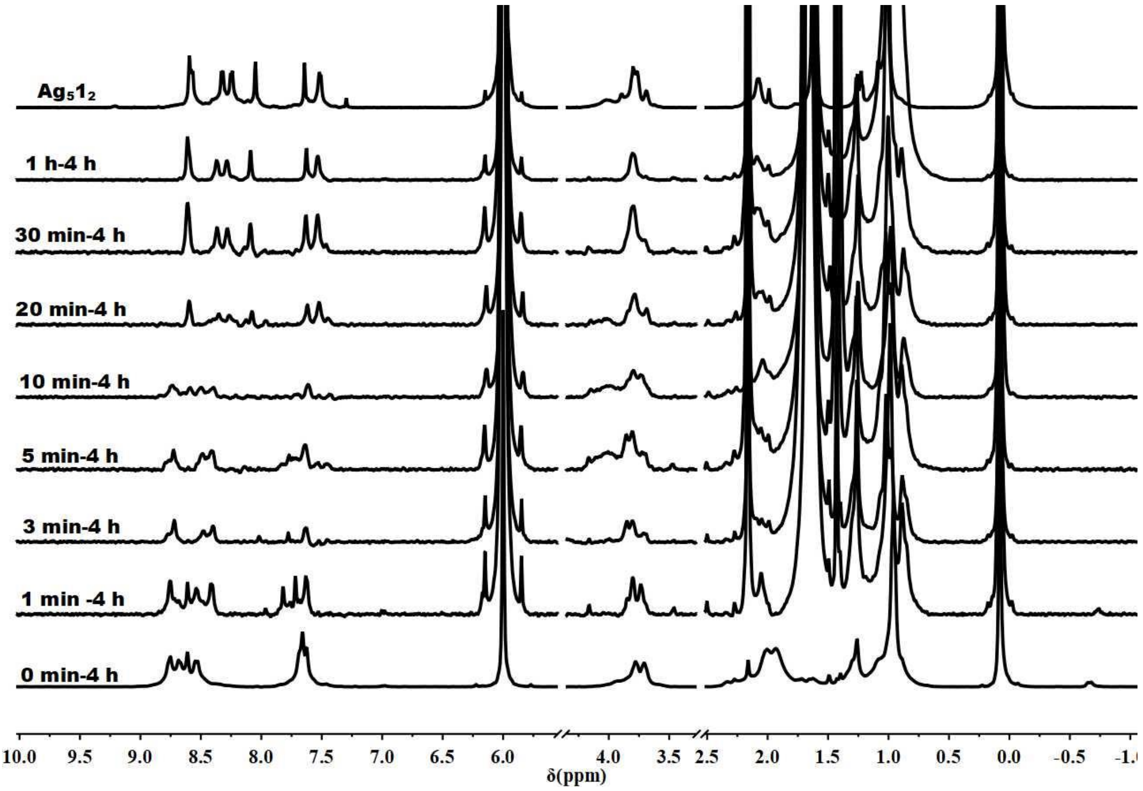

Supplementary Figure 101A. Time-depended ^{1}H NMR spectra (600 MHz, 298 K, $CDCl_2CDCl_2$) of the water-washed organic phase solution in the vigorously stirred bilayer mixture of **Ad-MeOH**$\subset$ $[Hg_5\mathbf{1}_2]\cdot[OTf]_{10}$ (**Ad-MeOH**$\subset$$\mathbf{Hg_5L_2}$) in $CDCl_2CDCl_2$ and AgEDTA in water. The spectra were recorded after the samples were sucked out of the bilayer mixture, washed with water and stored further at 28 °C for 4 hours. For comparison, ^{1}H NMR spectrum of $[Ag_5\mathbf{1}_2]\cdot[OTf]_5$ in $CDCl_2CDCl_2$ is shown.

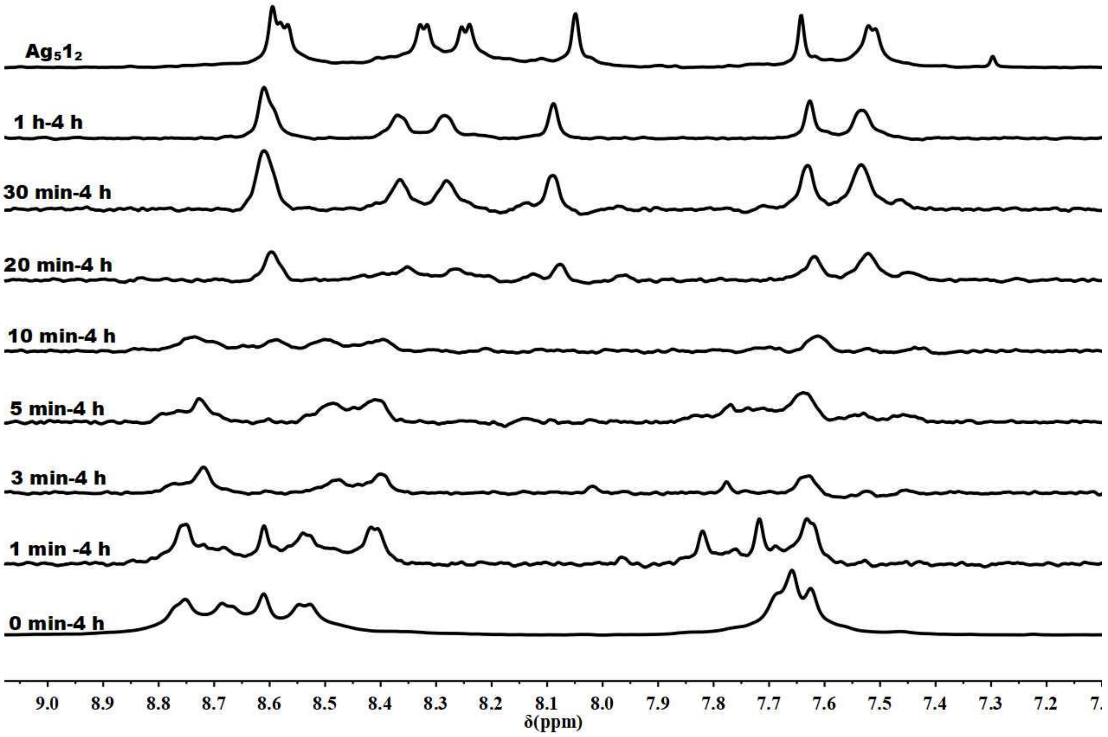

Supplementary Figure 101B. Zoom of ^{1}H NMR spectra 100A, which shows that **Ad-MeOH**$\subset$$[Hg_5\mathbf{1}_2]\cdot[OTf]_{10}$ (**Ad-MeOH**$\subset$$\mathbf{Hg_5L_2}$) was gradually converted to $[Ag_5\mathbf{1}_2]\cdot[OTf]_5$ ($\mathbf{Ag_5L_2}$) with **Ad-MeOH** released over time.

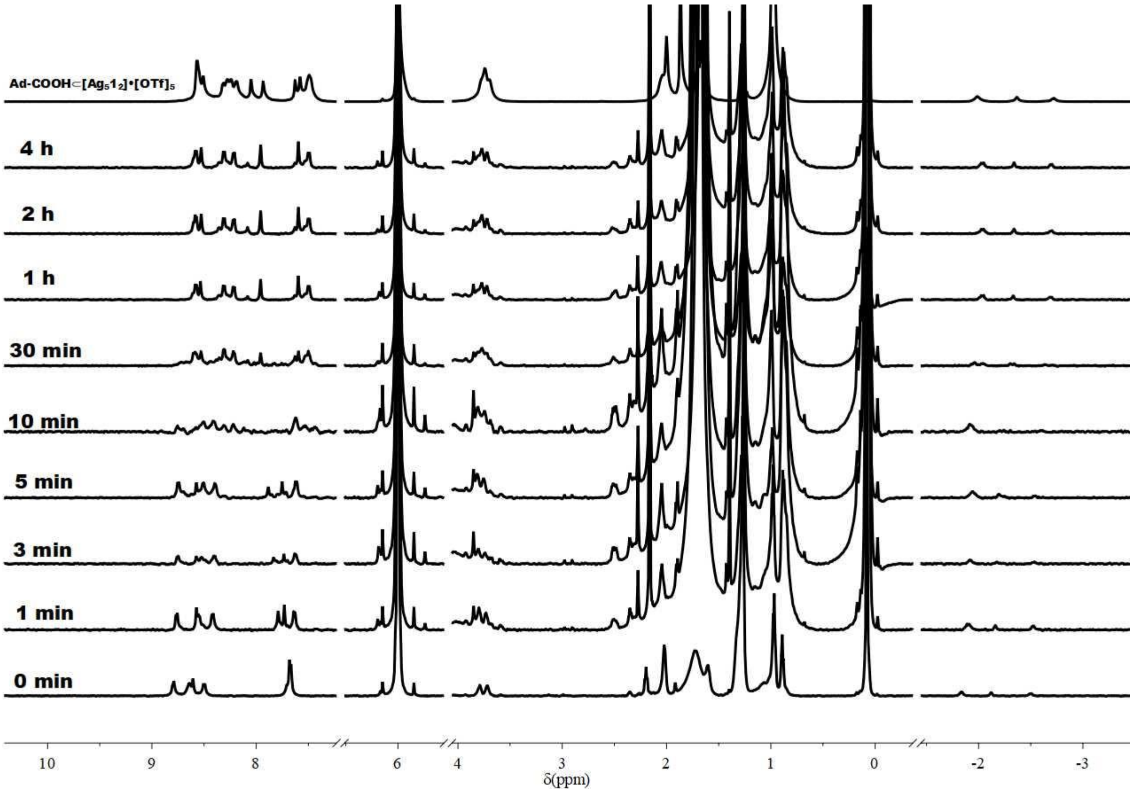

Supplementary Figure 102A. Time-depended ^{1}H NMR spectra (600 MHz, 298 K, $CDCl_2CDCl_2$) of the water-washed organic phase solution in the vigorously stirred bilayer mixture of **Ad-COOH⊂Hg$_5$L$_2$** in $CDCl_2CDCl_2$ and AgEDTA in water. The spectra were recorded soon after the samples were sucked out of the bilayer mixture and washed with water. For comparison, ^{1}H NMR spectrum of **Ad-COOH**⊂[Ag$_5$**1**$_2$]·[OTf]$_5$ in $CDCl_2CDCl_2$ is shown.

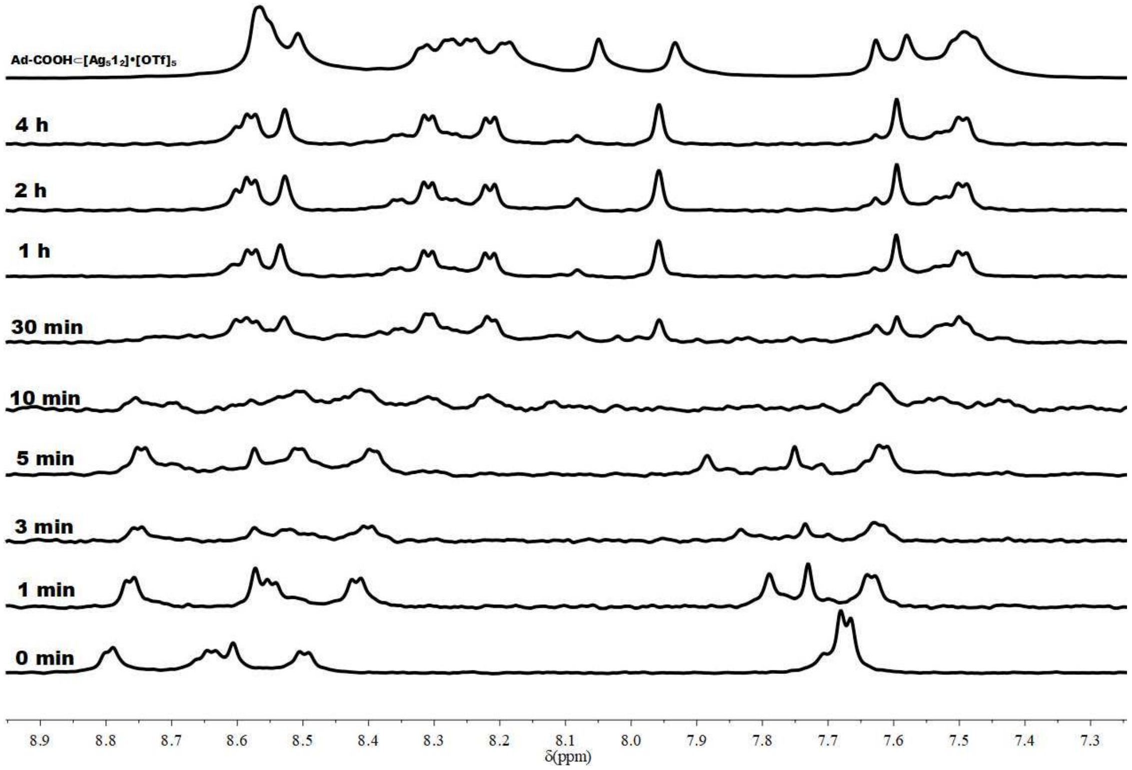

Supplementary Figure 102B. Zoom of ^{1}H NMR spectra 101A, which shows that **Ad-COOH**⊂[Hg$_5$**1**$_2$]·[OTf]$_{10}$ is gradually converted to **Ad-COOH**⊂[Ag$_5$**1**$_2$]·[OTf]$_5$ over time.

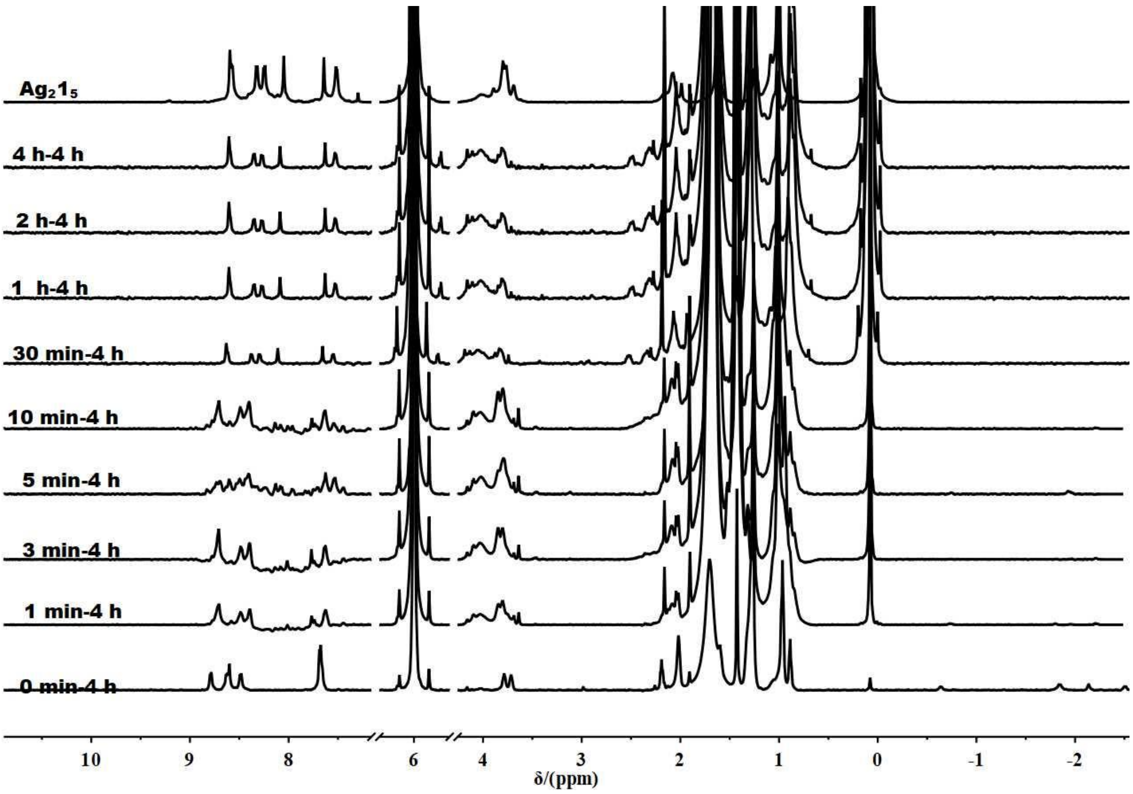

Supplementary Figure 103A. Time-depended ^{1}H NMR spectra (600 MHz, 298 K, $CDCl_2CDCl_2$) of the water-washed organic phase solution in the vigorously stirred bilayer mixture of **Ad-COOH**⊂ $[Hg_5\mathbf{1}_2]\cdot[OTf]_{10}$ (**Ad-COOH⊂Hg$_5$L$_2$**) in $CDCl_2CDCl_2$ and AgEDTA in water. The spectra were recorded after the samples were sucked out of the bilayer mixture, washed with water and stored further at 28 °C for 4 hours. For comparison, ^{1}H NMR spectrum of $[Ag_5\mathbf{1}_2]\cdot[OTf]_5$ in $CDCl_2CDCl_2$ is shown.

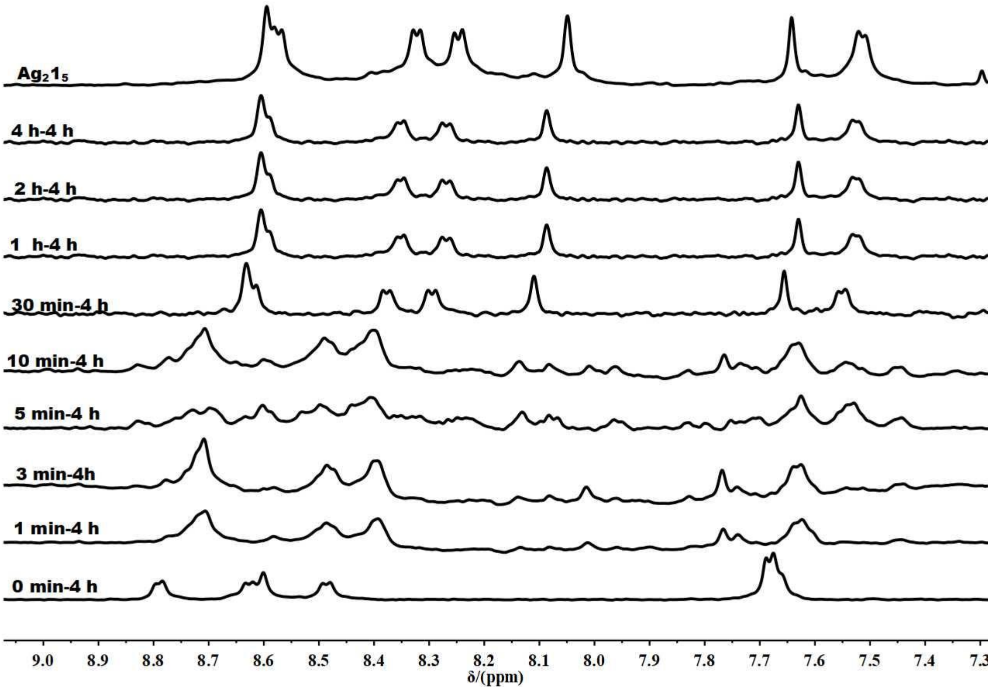

Supplementary Figure 103B. Zoom of ^{1}H NMR spectra 102A, which shows that **Ad-COOH**⊂$[Hg_5\mathbf{1}_2]\cdot[OTf]_{10}$ (**Ad-COOH⊂Hg$_5$L$_2$**) was gradually converted to $[Ag_5\mathbf{1}_2]\cdot[OTf]_5$ (**Ag$_5$L$_2$**) with **Ad-COOH** released over time.

Supplementary Method 15. Computational Details

Geometry optimizations were performed using the Gaussian 09 program package in Linux operating system. Optimized geometries for the cages and the cage-guest complexes in the gas phase were obtained by full system optimization at the DFT B3LYP//LANL2DZ/6-31G(d) levels of theory, using LANL2DZ pseudopotential for metal cations and 6-31G(d) basis set for the rest of the atoms.[S5] Dispersion was taken into account using Grimme's empirical dispersion correction (D3 version)[S6] with Becke–Johnson damping function[S7] (empiricaldispersion=gd3bj). Adamantane (**Ad**) was investigated as the example of the studied guests in the computations. To reduce the computational cost, we calculated simplified structures of the cages and the cage-guest complexes, in which all the *iso*-butoxy side chains are replaced with hydrogen atoms; besides, only the isolated states of the cages and cage-guest complexes (i.e., $[Ag_5\mathbf{1}_2]^{5+}$, $[Hg_5\mathbf{1}_2]^{10+}$, **Ad**⊂$[Ag_5\mathbf{1}_2]^{5+}$, **Ad**⊂$[Hg_5\mathbf{1}_2]^{10+}$), which could be regarded as the one when the cages/complexes are highly solvated, were involved investigated.

Cages $[Ag_5\mathbf{1}_2]^{5+}$and $[Hg_5\mathbf{1}_2]^{5+}$. For each kind of cages, totally eight structures (corresponding to four pairs of enantiomers), which include biconvex-*P,M,P*/*M,P,M*, biconvex-*P,P,P*/*M,M,M*, biconcave-*P,M,P*/*M,P,M* and biconcave-*P,P,P*/*M,M,M*, were considered. All the minima were obtained by full system optimization. Notably, some configurations, such as biconcave-(*P,P,P*/*M,M,M*) for $[Ag_5\mathbf{1}_2]^{5+}$, were proved to do not represent any minima (local or global) on the potential energy surfaces. The geometries and the corresponding energies as well as the structural parameters of the obtained three pairs of minima for cage $[Ag_5\mathbf{1}_2]^{5+}$, including [biconvex-(*P,M,P*/*M,P,M*)-$Ag_5\mathbf{1}_2]^{5+}$, [biconvex-(*P,P,P*/*M,M,M*)-$Ag_5\mathbf{1}_2]^{5+}$ and [biconcave -(*P,M,P*/*M,P,M*)-$Ag_5\mathbf{1}_2]^{5+}$, are summarized in Supplementary Tables 15 and 16; the ones for the $[Hg_5\mathbf{1}_2]^{10+}$ cages, including [biconvex-(*P,M,P*/*M,P,M*)-$Hg_5\mathbf{1}_2]^{10+}$ and [biconcave-(*M,P,M*/*P,M,P*) -$Hg_5\mathbf{1}_2]^{10+}$, are summarized in Supplementary Tables 15 and 16 as well.

Cage-guest complexes. The optimizations were carried out on the four stereoisomers of **Ad**⊂$[Ag_5\mathbf{1}_2]^{5+}$, including **Ad**⊂[biconvex-(*P,M,P*)-$Ag_5\mathbf{1}_2]^{5+}$, **Ad** ⊂ [biconvex-(*M,P,M*)-$Ag_5\mathbf{1}_2]^{5+}$, **Ad** ⊂ [biconvex-(*P,P,P*)-$Ag_5\mathbf{1}_2]^{5+}$ and **Ad** ⊂ [biconvex-(*M,M,M*)-$Ag_5\mathbf{1}_2]^{5+}$. Inclusion of **Ad** will give disassembly of [biconcave-(*P,M,P*/*M,P,M*)-$Ag_5\mathbf{1}_2]^{5+}$. In the cases of **Ad**⊂$[Hg_5\mathbf{1}_2]^{10+}$, DFT calculations showed that the inner cavity of biconcave-[(*P,M,P*/*M,P,M*)-$Hg_5\mathbf{1}_2]^{10+}$ are also too small to

include **Ad** so that only **Ad** ⊂ [biconvex-(*P*,*M*,*P*)-Hg_5**1**$_2$]$^{10+}$ and **Ad** ⊂ [biconvex-(*M*,*P*,*M*)-Hg_5**1**$_2$]$^{10+}$ are finally involved. The results relating to geometries, energies and structural parameters are summarized in Supplementary Tables 15−16.

Supplementary Table 15. The DFT//B3LYP//LANL2DZ/6-31G(d) calculations with Grimme's dispersion correction for the minima of isolated [Ag_5**L**$_2$]$^{5+}$ and [Hg_5**L**$_2$]$^{10+}$ as well as **Ad** ⊂ [Ag_5**L**$_2$]$^{5+}$ and **Ad** ⊂ [Hg_5**L**$_2$]$^{10+}$ in different conformers: energies (E°, hartree), relative energies (kcal mol^{-1}) and the important parameters for the geometries.[a,b]

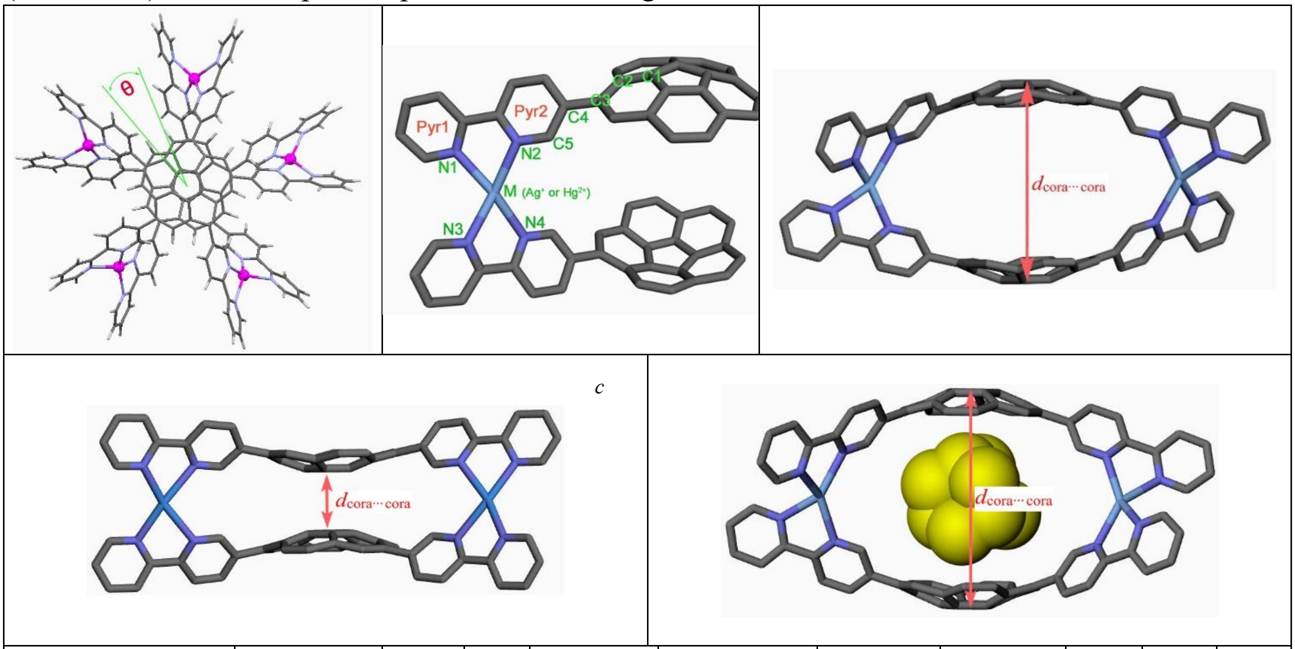

Geometry	E°	Rel. energies [d]	θ [e] (°)	Dihedral Angel (Pyr1/Pyr2) [f] (°)	Dihedral Angel (N1,N2,M)/(N3,N4,M) (°)	Dihedral Angel (C1-C2-C3-C4) (°)	Dihedral Angel (C3-C4-C5-N2) (°)	$d_{N1/N3\cdots M}$ [g] (Å)	$d_{N2/N4\cdots M}$ [g] (Å)	$d_{cora\cdots cora}$ [h] (Å)
[biconvex-(*P*,*M*,*P*)-Ag_5**L**$_2$]$^{5+}$	–7206.76111317	9.28	19.9	21.73	57.62	171.02	177.18	1.32	1.11	9.13
[biconvex-(*M*,*P*,*M*)-Ag_5**L**$_2$]$^{5+}$	–7206.76111555	9.28	19.3	21.70	57.60	-171.01	-177.08	1.31	1.11	9.13
[biconvex-(*P*,*P*,*P*)-Ag_5**L**$_2$]$^{5+}$	–7206.73833047	23.58	0.0	22.37	45.44	171.87	179.93	1.32	1.11	8.17
[biconvex-(*M*,*M*,*M*)-Ag_5**L**$_2$]$^{5+}$	–7206.73828392	23.61	0.0	22.35	45.47	-171.86	-179.92	1.47	1.08	8.18
[biconcave-(*M*,*P*,*M*)-Ag_5**L**$_2$]$^{5+}$	–7206.77578002	0.08	13.2	28.64	30.39	-170.05	173.32	2.35	2.47	3.17
[biconcave-(*P*,*M*,*P*)-Ag_5**L**$_2$]$^{5+}$	–7206.77590824	**0.00**	13.7	28.59	30.48	170.00	-173.28	2.35	2.46	3.18
[biconvex-(*P*,*M*,*P*)-Hg_5**L**$_2$]$^{10+}$	-6688.70701154	9.08	23.5	19.57	35.87	167.02	175.73	1.13	1.54	6.89
[biconvex-(*M*,*P*,*M*)-Hg_5**L**$_2$]$^{10+}$	-6688.70701149	9.08	23.0	19.57	35.87	-167.02	-175.72	1.13	1.54	6.89
[biconcave-(*M*,*P*,*M*)-Hg_5**L**$_2$]$^{10+}$	-6688.72145803	0.01	20.9	24.19	24.20	-170.44	171.10	1.17	1.58	3.28
[biconcave-(*P*,*M*,*P*)-Hg_5**L**$_2$]$^{10+}$	-6688.7214852	**0.00**	19.8	24.19	24.17	170.43	-171.10	1.17	1.58	3.28
**Ad**⊂[biconvex-(*P*,*M*,*P*)-Ag_5**L**$_2$]$^{5+}$	-7597.5875623	**0.00**	19.3	21.33	60.96	173.30	177.46	1.43	0.93	9.96
**Ad**⊂[biconvex-(*M*,*P*,*M*)-Ag_5**L**$_2$]$^{5+}$	-7597.58751968	0.03	20.1	21.37	60.98	169.27	176.88	1.43	0.93	9.97
**Ad**⊂[biconvex-(*P*,*P*,*P*)-Ag_5**L**$_2$]$^{5+}$	-7597.56315502	15.32	0.0	22.30	54.80	175.67	178.41	1.65	0.75	9.82

**Ad**⊂[biconvex-(*M*,*M*,*M*)-Ag_5**L**$_2$]$^{5+}$	-7597.56315188	15.32	0.0	22.30	54.80	-175.67	-178.41	1.65	0.75	9.82
**Ad**⊂[biconvex-(*P*,*M*,*P*)-Hg_5**L**$_2$]$^{10+}$	-7079.49943758	0.07	26.5	18.19	46.36	169.27	176.88	1.41	1.17	9.53
**Ad**⊂[biconvex-(*M*,*P*,*M*)-Hg_5**L**$_2$]$^{10+}$	-7079.4995547	**0.00**	26.1	18.05	46.11	-169.18	-176.81	1.41	1.17	9.53

[a] In the gas phase. [b] Models for elaboration of the structural parameters are shown at the top of the table. For clarity, some of the atoms were deleted. The two pyridyl rings that located at one pendant arm and coordinates with the same metal cation denotes "pyr1"and "pyr2", respectively, and Ag^+ and Hg^{2+} cations denote "M". Angles and distances show in the table are reported as means or ranges of all the related values obtained from the optimized structures. [c] In the cases of [(*P*,*P*,*P*)/(*M*,*M*,*M*)-Hg_5**1**$_2$]$^{10+}$. [d] Relative energy with relative to that of the most stable conformer. 1 Hartree = 627.5095 kcal mol^{-1}. [e] Twist angle between the two corannulene units. [f] The dihedral angle between the planes of two pyridyl rings that coordinates with the same silver cation. [g] The average distance between the mentioned nitrogen and the metal cation. [i] The distance between planes that the central pentagon of corannulene located.

Supplementary Table 16. Summary of the illustrations of the DFT optimized structures of the different isolated [Ag_5**1**$_2$]$^{5+}$ and [Hg_5**1**$_2$]$^{10+}$ cage conformers as well those of **Ad** ⊂ [Ag_5**L**$_2$]$^{5+}$ and **Ad** ⊂ [Hg_5**L**$_2$]$^{5+}$: top and side view, side view with partial groups omitted for clarity (in the cases of cage-guest complexes). Guest **Ad** in the host-guest complexes is represented as CPK sphere.

[biconvex-(*P*,*M*,*P*)-Ag_5**1**$_2$]$^{5+}$	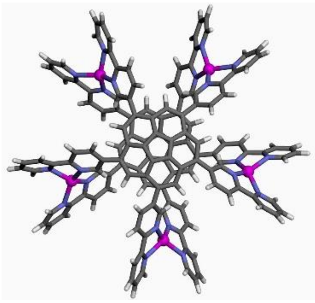	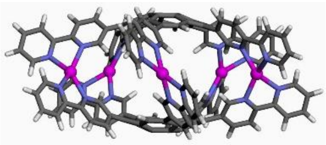	/
[biconvex-(*M*,*P*,*M*)-Ag_5**1**$_2$]$^{5+}$	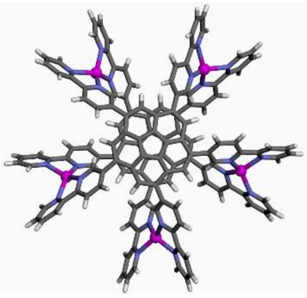	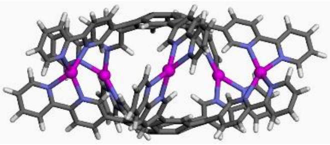	/
[biconvex-(*P*,*P*,*P*)-Ag_5**1**$_2$]$^{5+}$	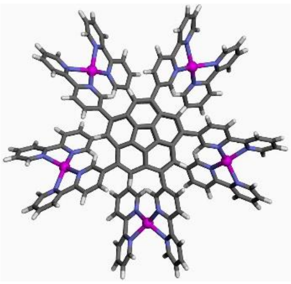	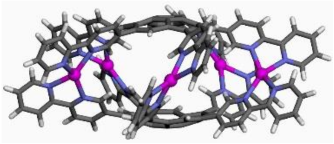	/
[biconvex-(*M*,*M*,*M*)-Ag_5**1**$_2$]$^{5+}$	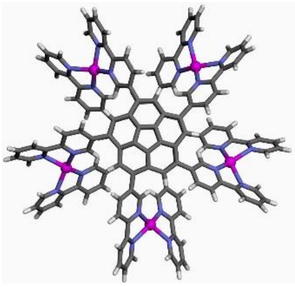	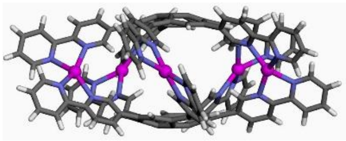	/

[biconcave-(*M*,*P*,*M*)-$Ag_5\mathbf{L}_2$]$^{5+}$	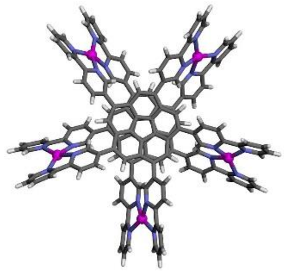	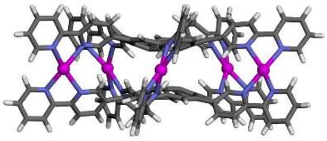	/
[biconcave-(*P*,*M*,*P*)-$Ag_5\mathbf{L}_2$]$^{5+}$	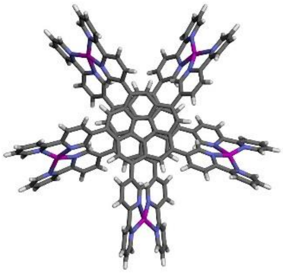	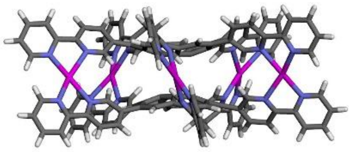	/
[biconvex-(*P*,*M*,*P*)-$Hg_5\mathbf{1}_2$]$^{10+}$	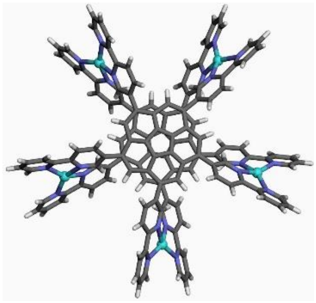	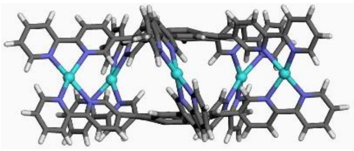	/
[biconvex-(*M*,*P*,*M*)-$Hg_5\mathbf{1}_2$]$^{10+}$	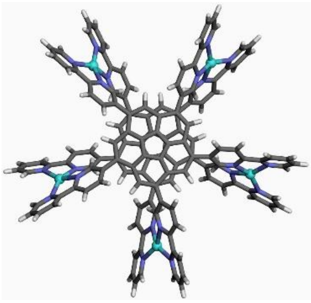	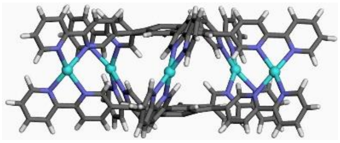	/
[biconcave-(*M*,*P*,*M*)-$Hg_5\mathbf{1}_2$]$^{10+}$	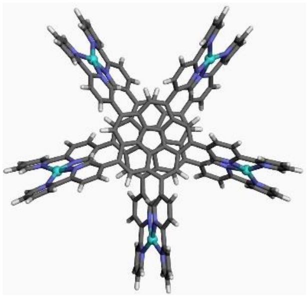	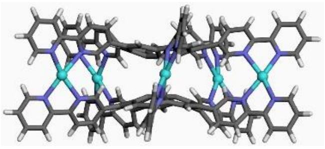	/
[biconcave-(*P*,*M*,*P*)-$Hg_5\mathbf{1}_2$]$^{10+}$	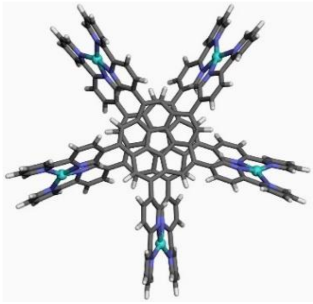	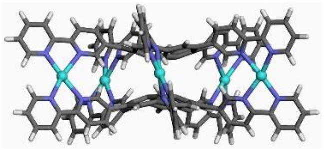	/

**Ad**⊂[biconvex-(*P*,*M*,*P*)-Ag$_5$**L**$_2$]$^{5+}$	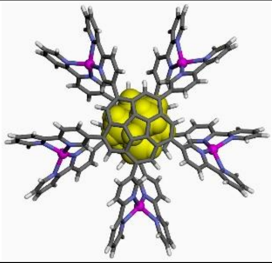	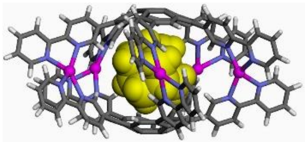	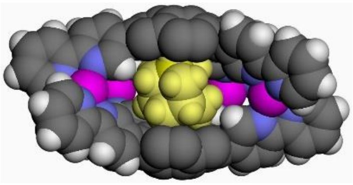
**Ad**⊂[biconvex-(*M*,*P*,*M*)-Ag$_5$**1**$_2$]$^{5+}$	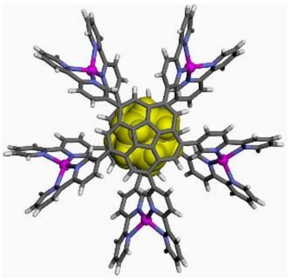	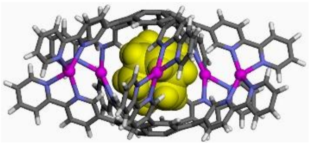	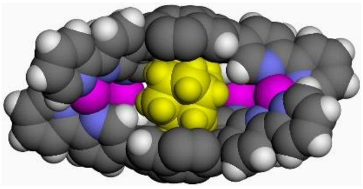
**Ad**⊂[biconvex-(*P*,*P*,*P*)-Ag$_5$**1**$_2$]$^{5+}$	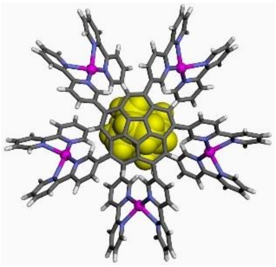	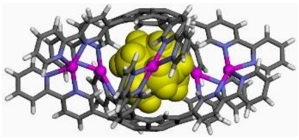	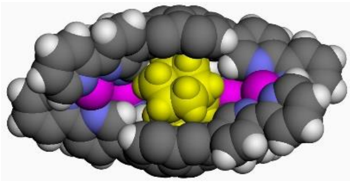
**Ad**⊂[biconvex-(*M*,*M*,*M*)-Ag$_5$**1**$_2$]$^{5+}$	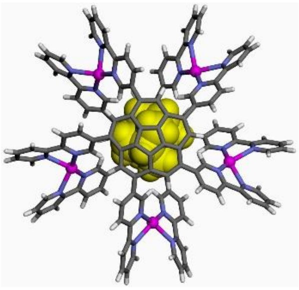	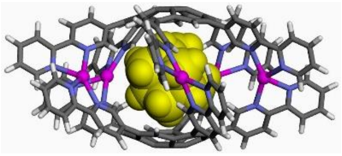	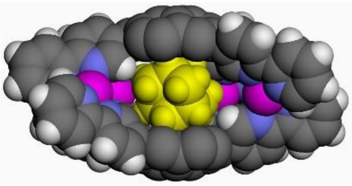
**Ad**⊂[biconvex-(*P*,*M*,*P*)-Hg$_5$**L**$_2$]$^{10+}$	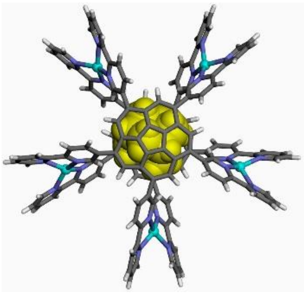	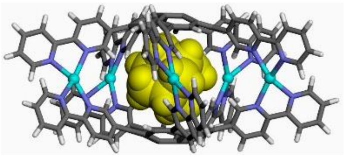	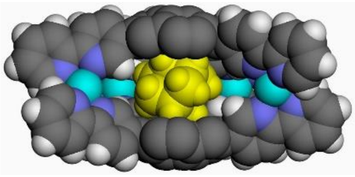
**Ad**⊂[biconvex-(*M*,*P*,*M*)-Hg$_5$**1**$_2$]$^{10+}$	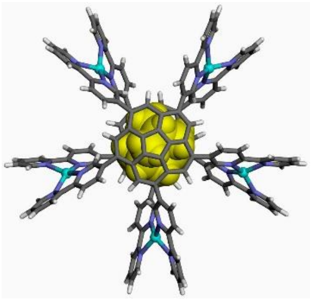	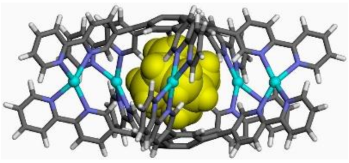	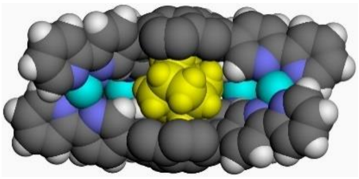

Supplementary Table 17. Optimized Geometries/Coordinate Outputs of DFT Calculations

[biconvex-(*P*,*M*,*P*)-$Ag_5\mathbf{L}_2]^{5+}$:

Center Number	Atomic Number	Atomic Type	Coordinates (Angstroms) X	Y	Z
1	47	0	6.191621	-4.621632	0.001344
2	47	0	-2.484952	-7.319331	-0.000780
3	47	0	-7.733782	0.098425	-0.000519
4	6	0	-3.171621	4.529719	-4.165986
5	6	0	-3.941345	5.640999	-3.850338
6	6	0	-3.561777	6.468736	-2.784048
7	7	0	-2.477619	6.168852	-2.038307
8	6	0	-2.021992	4.237485	-3.415359
9	6	0	-1.741812	5.096213	-2.343917
10	1	0	-3.444331	3.900917	-5.007762
11	1	0	-4.832727	5.859408	-4.425688
12	1	0	-0.888670	4.903382	-1.699218
13	6	0	5.228120	-1.801794	-4.168556
14	6	0	4.126104	-2.239317	-3.417185
15	6	0	4.403858	-3.096116	-2.343569
16	7	0	5.629572	-3.530501	-2.036410
17	6	0	6.503918	-2.247767	-3.851546
18	6	0	6.683167	-3.137866	-2.783015
19	1	0	5.079416	-1.134641	-5.011878
20	6	0	8.025022	-3.675558	-2.429889
21	6	0	-0.095111	-5.528615	-4.166732
22	6	0	-0.123633	-6.879736	-3.849422
23	6	0	-0.917117	-7.326277	-2.783029
24	7	0	-1.619791	-6.446490	-2.038821
25	6	0	-0.854677	-4.616565	-3.417251
26	6	0	-1.586122	-5.146485	-2.345748
27	1	0	0.495003	-5.180432	-5.008683
28	1	0	0.470435	-7.580183	-4.423662
29	1	0	-2.163723	-4.489090	-1.701610
30	6	0	-5.288400	-1.615752	-4.167924
31	6	0	-6.582812	-2.005312	-3.852238
32	6	0	-7.252413	-1.390512	-2.784607
33	7	0	-6.632433	-0.452495	-2.038079
34	6	0	-4.655084	-0.613816	-3.415912
35	6	0	-5.385150	-0.083236	-2.343792
36	1	0	-4.774979	-2.067877	-5.010738
37	1	0	-7.066109	-2.784704	-4.428655
38	6	0	-0.891804	-3.178509	-3.760109
39	6	0	-2.116368	-2.500887	-3.752511
40	6	0	-2.243245	-1.096092	-4.072133
41	6	0	-1.085312	-0.525525	-4.562319
42	6	0	0.349056	-2.471757	-4.073281
43	6	0	0.164335	-1.193976	-4.562933
44	6	0	1.186242	-0.212105	-4.562926
45	6	0	1.724165	-2.785255	-3.753014
46	6	0	2.747002	-1.830096	-3.760015
47	6	0	2.458477	-0.431549	-4.073251
48	6	0	0.568253	1.063288	-4.562276
49	6	0	1.170156	2.205190	-4.071954
50	6	0	2.589457	2.047248	-3.759055
51	6	0	3.181643	0.779190	-3.752637
52	6	0	-0.835641	0.869538	-4.561794
53	6	0	-1.735708	1.794395	-4.070912
54	6	0	-1.146994	3.095409	-3.757635
55	6	0	0.241967	3.267029	-3.751178
56	6	0	-3.298596	-0.134252	-3.758040
57	6	0	-3.032310	1.239655	-3.750228
58	1	0	-2.997890	-3.062811	-3.460398
59	1	0	4.225271	0.715220	-3.461235
60	1	0	0.625036	4.239808	-3.459444
61	1	0	-3.838889	1.904627	-3.458002
62	6	0	3.330279	4.415645	-4.166985
63	6	0	3.405736	3.231865	-3.416356
64	6	0	4.308218	3.229781	-2.344161
65	7	0	5.101824	4.260203	-2.037890
66	6	0	4.150267	5.490162	-3.850622
67	6	0	5.053780	5.384075	-2.783594
68	1	0	2.648920	4.481527	-5.009468
69	6	0	5.981171	6.493149	-2.430878
70	6	0	-4.330941	7.693200	-2.432663
71	1	0	3.600223	-3.440144	-1.698206
72	1	0	7.353575	-1.902002	-4.427593
73	6	0	-1.011812	-8.768698	-2.429618
74	6	0	-8.653893	-1.745858	-2.432616
75	1	0	1.986094	-3.797365	-3.461076
76	1	0	4.387129	2.358745	-1.699361
77	1	0	4.084192	6.405389	-4.426206
78	6	0	0.046046	5.531738	4.167010
79	6	0	0.051001	6.883492	3.851201
80	6	0	-0.728876	7.350341	2.783547
81	7	0	-1.451027	6.488730	2.036661
82	6	0	-0.734253	4.639364	3.415184
83	6	0	-1.449977	5.188010	2.342450
84	1	0	0.625713	5.168492	5.009886
85	1	0	0.660644	7.568727	4.427452
86	1	0	-2.042454	4.545744	1.696564
87	6	0	-3.287539	-4.444557	4.167146
88	6	0	-2.131538	-4.181354	3.415632
89	6	0	-1.874332	-5.046388	2.343505
90	7	0	-2.637692	-6.099658	2.037882
91	6	0	-4.085663	-5.535634	3.851611
92	6	0	-3.728497	-6.372173	2.784465
93	1	0	-3.543277	-3.809703	5.009707

[biconvex-(*M*,*P*,*M*)-$Ag_5\mathbf{L}_2]^{5+}$:

Center Number	Atomic Number	Atomic Type	Coordinates (Angstroms) X	Y	Z
1	47	0	-2.328088	7.367548	0.000211
2	47	0	6.297541	4.487394	0.001457
3	47	0	6.219777	-4.597776	-0.001330
4	6	0	-0.071058	-5.529545	4.164337
5	6	0	-0.095318	-6.881140	3.848764
6	6	0	-0.886940	-7.331370	2.782581
7	7	0	-1.592313	-6.454793	2.037190
8	6	0	-0.833677	-4.620889	3.413859
9	6	0	-1.563146	-5.154371	2.342814
10	1	0	0.517433	-5.178508	5.006254
11	1	0	0.500616	-7.579058	4.424118
12	1	0	-2.143689	-4.499782	1.698482
13	6	0	-3.189627	4.516868	4.170596
14	6	0	-2.040548	4.227837	3.417988
15	6	0	-1.766212	5.085693	2.344338
16	7	0	-2.507421	6.154435	2.037970
17	6	0	-3.964761	5.624207	3.854415
18	6	0	-3.591378	6.451202	2.785455
19	1	0	-3.457882	3.888801	5.014379
20	6	0	-4.367110	7.671080	2.432377
21	6	0	3.307991	4.430795	4.164501
22	6	0	4.122057	5.509836	3.848449
23	6	0	5.028084	5.407993	2.783191
24	7	0	5.083625	4.283776	2.038343
25	6	0	3.391151	3.246887	3.414886
26	6	0	4.295191	3.249244	2.343988
27	1	0	2.625351	4.493574	5.006198
28	1	0	4.049682	6.425296	4.422901
29	1	0	4.379676	2.378651	1.699305
30	6	0	5.236359	-1.778083	4.165359
31	6	0	6.514515	-2.218149	3.849868
32	6	0	6.698820	-3.108629	2.782573
33	7	0	5.647656	-3.507771	2.036012
34	6	0	4.137009	-2.221450	3.413421
35	6	0	4.419661	-3.078699	2.341436
36	1	0	5.083934	-1.111283	5.008301
37	1	0	7.362100	-1.867809	4.426164
38	6	0	2.580763	2.058258	3.757265
39	6	0	3.178692	0.792864	3.749524
40	6	0	2.461179	-0.421291	4.069670
41	6	0	1.188371	-0.207988	4.560627
42	6	0	1.160993	2.209500	4.071307
43	6	0	0.564662	1.064563	4.561434
44	6	0	-0.838330	0.864556	4.562099
45	6	0	0.227857	3.267242	3.751709
46	6	0	-1.160324	3.089592	3.759639
47	6	0	-1.742970	1.785929	4.072978
48	6	0	-1.081813	-0.531582	4.561437
49	6	0	-2.237557	-1.106789	4.071499
50	6	0	-3.297490	-0.149394	3.759307
51	6	0	-3.037327	1.225719	3.752997
52	6	0	0.170814	-1.194445	4.560485
53	6	0	0.360955	-2.470804	4.069338
54	6	0	-0.876871	-3.182873	3.756321
55	6	0	-2.104466	-2.510755	3.750355
56	6	0	2.755941	-1.818363	3.755578
57	6	0	1.737288	-2.777966	3.748164
58	1	0	4.222386	0.733810	3.457226
59	1	0	-3.847050	1.887447	3.462022
60	1	0	-2.983724	-3.076574	3.458920
61	1	0	2.003555	-3.788831	3.455752
62	6	0	-5.281797	-1.639894	4.166322
63	6	0	-4.651996	-0.634045	3.416588
64	6	0	-5.383815	-0.104205	2.345315
65	7	0	-6.629408	-0.477821	2.038378
66	6	0	-6.574665	-2.033808	3.849437
67	6	0	-7.246127	-1.419815	2.782538
68	1	0	-4.767220	-2.091421	5.008759
69	6	0	-8.645923	-1.779889	2.428111
70	6	0	-0.977506	-8.774483	2.430717
71	1	0	-0.913880	4.895411	1.697797
72	1	0	-4.855822	5.840004	4.431268
73	6	0	5.950275	6.521831	2.431775
74	6	0	8.043786	-3.639426	2.430634
75	1	0	0.606234	4.241877	3.459929
76	1	0	-4.940100	0.650537	1.701742
77	1	0	-7.055170	-2.816004	4.424403
78	6	0	-3.270295	-4.459619	-4.165387
79	6	0	-4.065157	-5.552990	-3.849644
80	6	0	-3.705425	-6.388228	-2.782382
81	7	0	-2.615490	-6.112225	-2.035756
82	6	0	-2.115147	-4.192632	-3.413845
83	6	0	-1.855405	-5.056645	-2.341424
84	1	0	-3.528047	-3.825437	-5.007840
85	1	0	-4.960706	-5.751632	-4.425686
86	1	0	-0.999138	-4.882426	-1.695538
87	6	0	5.264845	1.689156	-4.168941
88	6	0	4.173473	2.149281	-3.415507
89	6	0	4.470809	3.000234	-2.342481
90	7	0	5.705808	3.409648	-2.037929
91	6	0	6.550123	2.108861	-3.854447
92	6	0	6.749548	2.995430	-2.786570
93	1	0	5.100899	1.025236	-5.011997

94	6	0	-4.529308	-7.576163	2.432892
95	6	0	3.211722	-4.499370	4.169489
96	6	0	4.001194	-5.596300	3.852359
97	6	0	4.906485	-5.515162	2.784668
98	7	0	4.985765	-4.392556	2.039629
99	6	0	3.319622	-3.317900	3.419221
100	6	0	4.221601	-3.340396	2.346861
101	1	0	2.528963	-4.546748	5.012112
102	1	0	3.909795	-6.509590	4.427545
103	1	0	4.324801	-2.471576	1.702527
104	6	0	5.273116	1.666301	4.170894
105	6	0	6.560621	2.077272	3.853944
106	6	0	6.764049	2.961885	2.785197
107	7	0	5.721442	3.383612	2.039044
108	6	0	4.183424	2.133019	3.419057
109	6	0	4.484354	2.982544	2.345918
110	1	0	5.106413	1.003802	5.014506
111	1	0	7.400614	1.708647	4.429939
112	1	0	3.690538	3.348315	1.700357
113	6	0	2.535133	-2.111984	3.761511
114	6	0	3.160346	-0.859859	3.755046
115	6	0	2.468800	0.369524	4.074643
116	6	0	1.190874	0.183541	4.563494
117	6	0	1.111975	-2.232685	4.073552
118	6	0	0.539840	-1.075271	4.563096
119	6	0	-0.858539	-0.845015	4.561558
120	6	0	0.156722	-3.269984	3.752303
121	6	0	-1.227300	-3.062297	3.757911
122	6	0	-1.782091	-1.746365	4.070548
123	6	0	-1.071772	0.556034	4.561120
124	6	0	-2.214042	1.156287	4.069717
125	6	0	-3.294066	0.222242	3.755661
126	6	0	-3.063681	-1.158218	3.749067
127	6	0	0.194916	1.191732	4.562255
128	6	0	0.413231	2.463986	4.072059
129	6	0	-0.808505	3.202587	3.757426
130	6	0	-2.050260	2.557115	3.749360
131	6	0	2.793937	1.760007	3.761240
132	6	0	1.796249	2.741448	3.752771
133	1	0	4.205414	-0.823290	3.463966
134	1	0	-3.887251	-1.802170	3.457145
135	1	0	-2.916653	3.141802	3.456766
136	1	0	2.084566	3.746335	3.460707
137	6	0	-5.244449	1.756296	4.162719
138	6	0	-4.637531	0.736592	3.412905
139	6	0	-5.381443	0.223113	2.341925
140	7	0	-6.619023	0.623374	2.036218
141	6	0	-6.528702	2.178221	3.846956
142	6	0	-7.214741	1.578214	2.781359
143	1	0	-4.719266	2.196913	5.004373
144	6	0	-8.607651	1.967029	2.430365
145	6	0	-0.788045	8.795072	2.431856
146	1	0	-1.017021	-4.875437	1.698131
147	1	0	-4.981670	-5.731738	4.427782
148	6	0	5.801400	-6.649972	2.430164
149	6	0	8.120248	3.461287	2.430698
150	1	0	0.514479	-4.252491	3.460968
151	1	0	-4.954359	-0.540333	1.697397
152	1	0	-6.991219	2.971067	4.422031
153	47	0	6.313240	4.458547	0.001359
154	47	0	-2.292810	7.386288	-0.000641
155	6	0	6.123716	-7.646808	3.358736
156	6	0	6.964476	-8.690419	2.975768
157	6	0	7.464170	-8.713651	1.675378
158	6	0	7.109481	-7.676146	0.814477
159	7	0	6.302426	-6.671086	1.178360
160	7	0	8.188353	-4.149698	-1.178105
161	6	0	9.380703	-4.640352	-0.815596
162	6	0	10.474956	-4.688347	-1.677980
163	6	0	10.310046	-4.215791	-2.978288
164	6	0	9.071104	-3.703781	-3.359825
165	1	0	5.752936	-7.598510	4.375670
166	1	0	7.230228	-9.466530	3.686204
167	1	0	8.119095	-9.507881	1.334338
168	1	0	7.481639	-7.644240	-0.205291
169	1	0	9.456415	-5.005941	0.204253
170	1	0	11.422130	-5.092496	-1.338038
171	1	0	11.128705	-4.251966	-3.689749
172	1	0	8.918816	-3.361890	-4.376627
173	6	0	9.167663	3.460233	3.359568
174	6	0	10.420394	3.936264	2.976409
175	6	0	10.597323	4.402907	1.675551
176	6	0	9.501165	4.385946	0.814452
177	7	0	8.295556	3.929993	1.178503
178	7	0	6.483452	6.501424	-1.179377
179	6	0	7.319493	7.483251	-0.817502
180	6	0	7.703420	8.508884	-1.680060
181	6	0	7.202312	8.498491	-2.980076
182	6	0	6.331438	7.479091	-3.360972
183	6	0	-0.464289	9.789473	3.362580
184	6	0	-0.533074	11.128684	2.982487
185	6	0	-0.924345	11.443365	1.682820
186	6	0	-1.245611	10.396922	0.819639
187	7	0	-1.181503	9.108775	1.180784
188	7	0	-4.184286	8.174780	-1.181657
189	6	0	-4.860563	9.273208	-0.820978
190	6	0	-5.717678	9.953797	-1.684385
191	6	0	-5.861971	9.472570	-2.983950
192	6	0	-5.160580	8.329530	-3.363611
193	6	0	-9.452818	2.583767	3.360523
194	6	0	-10.748545	2.930150	2.981306
195	6	0	-11.170232	2.651595	1.682908
196	6	0	-10.274755	2.021411	0.820184

94	6	0	8.102865	3.505940	-2.436703
95	6	0	0.022453	5.529833	-4.167174
96	6	0	0.021377	6.881307	-3.849961
97	6	0	-0.761862	7.343813	-2.782804
98	7	0	-1.482119	6.478610	-2.038347
99	6	0	-0.755404	4.633518	-3.417480
100	6	0	-1.475431	5.178241	-2.345682
101	1	0	0.605070	5.169739	-5.009383
102	1	0	0.629010	7.569689	-4.424620
103	1	0	-2.066857	4.532770	-1.702066
104	6	0	-5.251676	1.731716	-4.168153
105	6	0	-6.536626	2.150610	-3.851219
106	6	0	-7.219200	1.550797	-2.783284
107	7	0	-6.620167	0.598512	-2.037750
108	6	0	-4.640920	0.715008	-3.417302
109	6	0	-5.381943	0.200861	-2.344727
110	1	0	-4.728492	2.172815	-5.010826
111	1	0	-7.002449	2.941403	-4.426520
112	1	0	-4.951647	-0.560404	-1.699758
113	6	0	-0.822969	3.196408	-3.760059
114	6	0	-2.061994	2.545625	-3.753806
115	6	0	-2.219112	1.143836	-4.073202
116	6	0	-1.073441	0.548094	-4.562245
117	6	0	0.402461	2.462779	-4.072201
118	6	0	0.190442	1.189257	-4.561987
119	6	0	1.190729	0.185280	-4.560667
120	6	0	1.783767	2.746397	-3.750991
121	6	0	2.785667	1.769261	-3.757190
122	6	0	2.467043	0.377151	-4.070020
123	6	0	0.545070	-1.076297	-4.560015
124	6	0	1.121404	-2.230847	-4.068718
125	6	0	2.543831	-2.103930	-3.755228
126	6	0	3.163563	-0.849024	-3.748822
127	6	0	-0.854266	-0.852019	-4.560962
128	6	0	-1.774831	-1.756888	-4.070663
129	6	0	-1.214957	-3.070215	-3.755930
130	6	0	0.169949	-3.271943	-3.748094
131	6	0	-3.295362	0.205593	-3.759506
132	6	0	-3.059313	-1.173895	-3.751421
133	1	0	-2.931409	3.126736	-3.462968
134	1	0	4.208243	-0.807643	-3.457078
135	1	0	0.531502	-4.252710	-3.455703
136	1	0	-3.880515	-1.820937	-3.459675
137	6	0	3.228368	-4.489002	-4.161930
138	6	0	3.333377	-3.306574	-3.412728
139	6	0	4.238057	-3.324918	-2.342541
140	7	0	5.007690	-4.373613	-2.036960
141	6	0	4.022725	-5.582651	-3.845918
142	6	0	4.930989	-5.497022	-2.781146
143	1	0	2.543839	-4.539477	-5.002890
144	6	0	5.832332	-6.627646	-2.429305
145	6	0	-4.502593	-7.594662	-2.430717
146	1	0	3.675550	3.360504	-1.695557
147	1	0	7.391487	1.745771	-4.432025
148	6	0	-0.826574	8.787653	-2.428334
149	6	0	-8.611778	1.937792	-2.429213
150	1	0	2.067327	3.752632	-3.458963
151	1	0	4.338941	-2.455675	-1.698421
152	1	0	3.933003	-6.496758	-4.420073
153	47	0	-7.727900	0.070289	-0.000623
154	47	0	-2.457588	-7.334640	0.001526
155	6	0	-0.506100	9.785273	-3.356698
156	6	0	-0.580313	11.123393	-2.973788
157	6	0	-0.973561	11.433780	-1.673680
158	6	0	-1.291342	10.384247	-0.812969
159	7	0	-1.221887	9.097066	-1.176758
160	7	0	-4.223928	8.150623	1.180208
161	6	0	-4.906450	9.244389	0.817331
162	6	0	-5.766632	9.922305	1.679815
163	6	0	-5.907449	9.443229	2.980592
164	6	0	-5.199589	8.304906	3.362458
165	1	0	-0.232589	9.529577	-4.373447
166	1	0	-0.342899	11.908808	-3.684046
167	1	0	-1.042316	12.460948	-1.332731
168	1	0	-1.613073	10.575632	0.206528
169	1	0	-4.755401	9.584577	-0.202933
170	1	0	-6.300471	10.802845	1.339649
171	1	0	-6.550924	9.950619	3.692066
172	1	0	-5.274834	7.938901	4.379484
173	6	0	-9.460647	2.551350	-3.358046
174	6	0	-10.755751	2.896368	-2.975406
175	6	0	-11.172653	2.620108	-1.674955
176	6	0	-10.273424	1.993267	-0.813665
177	7	0	-9.028406	1.659224	-1.177331
178	7	0	-9.057406	-1.492319	1.176486
179	6	0	-10.308498	-1.802753	0.812685
180	6	0	-11.219122	-2.413728	1.673352
181	6	0	-10.807457	-2.698743	2.973595
182	6	0	-9.506211	-2.378054	3.356476
183	6	0	-5.347456	-8.211446	-3.361130
184	6	0	-6.074791	-9.338024	-2.981072
185	6	0	-5.941008	-9.822439	-1.681537
186	6	0	-5.068024	-9.161838	-0.818569
187	7	0	-4.366428	-8.079604	-1.179836
188	7	0	-1.379347	-9.079172	1.180038
189	6	0	-1.471565	-10.365632	0.818741
190	6	0	-1.171509	-11.418999	1.681114
191	6	0	-0.771893	-11.113238	2.980363
192	6	0	-0.674006	-9.775910	3.360683
193	6	0	6.154440	-7.624641	-3.357782
194	6	0	7.001834	-8.663830	-2.977385
195	6	0	7.508193	-8.682665	-1.679498
196	6	0	7.152859	-7.645524	-0.818451

197	7	0	-9.028981	1.686520	1.180469
198	7	0	-9.066379	-1.458303	-1.181387
199	6	0	-10.319161	-1.764612	-0.819817
200	6	0	-11.230725	-2.371086	-1.682677
201	6	0	-10.818013	-2.655978	-2.982576
202	6	0	-9.515010	-2.339674	-3.363093
203	6	0	-5.373903	-8.191862	3.364286
204	6	0	-6.105160	-9.315916	2.984272
205	6	0	-5.975206	-9.799035	1.683886
206	6	0	-5.101946	-9.139867	0.820060
207	7	0	-4.396739	-8.059919	1.181131
208	6	0	-0.709460	-9.771873	-3.358119
209	6	0	-0.811186	-11.108559	-2.976481
210	6	0	-1.213146	-11.412007	-1.677398
211	6	0	-1.511593	-10.356942	-0.816505
212	7	0	-1.415908	-9.071198	-1.179176
213	1	0	9.006595	3.123744	4.376816
214	1	0	11.240486	3.949590	3.687013
215	1	0	11.555357	4.779369	1.334237
216	1	0	9.586426	4.748567	-0.205703
217	1	0	7.691449	7.441867	0.202009
218	1	0	8.381249	9.284292	-1.340460
219	1	0	7.489843	9.265696	-3.691700
220	1	0	5.958462	7.440247	-4.377492
221	1	0	-0.192202	9.530617	4.378917
222	1	0	-0.292994	11.911659	3.694532
223	1	0	-0.988776	12.471500	1.343958
224	1	0	-1.565504	10.591484	-0.199814
225	1	0	-4.707070	9.615224	0.198307
226	1	0	-6.246406	10.838023	-1.345820
227	1	0	-6.502922	9.981962	-3.696267
228	1	0	-5.238146	7.961975	-4.379881
229	1	0	-9.121766	2.765085	4.376107
230	1	0	-11.418471	3.401463	3.693168
231	1	0	-12.168680	2.906042	1.344699
232	1	0	-10.559966	1.774261	-0.198168
233	1	0	-10.596641	-1.513667	0.199742
234	1	0	-12.234358	-2.602735	-1.343418
235	1	0	-11.500230	-3.109188	-3.694533
236	1	0	-9.189803	-2.526218	-4.379647
237	1	0	-5.440547	-7.823527	4.381054
238	1	0	-6.758176	-9.809392	3.696831
239	1	0	-6.527275	-10.668736	1.344945
240	1	0	-4.958757	-9.484432	-0.199848
241	1	0	-0.428738	-9.521006	-4.374088
242	1	0	-0.588160	-11.897993	-3.686935
243	1	0	-1.303014	-12.437819	-1.337299
244	1	0	-1.838603	-10.542444	0.202378
245	1	0	-4.938457	0.668396	-1.698619

[biconvex-(*P*,*P*,*P*)-Ag_5**L**$_2$]$^{5+}$:

Center Number	Atomic Number	Atomic Type	Coordinates (Angstroms) X	Y	Z
1	47	0	6.735981	-3.397967	-0.004521
2	6	0	-4.750080	-3.340322	3.794780
3	6	0	-6.046936	-3.734625	3.495711
4	6	0	-6.781639	-3.030610	2.531291
5	7	0	-6.226638	-1.991133	1.874271
6	6	0	-4.973803	-1.620666	2.159329
7	1	0	-4.182372	-3.865413	4.556587
8	1	0	-6.480161	-4.589769	4.000098
9	1	0	-4.581255	-0.792817	1.576769
10	6	0	-8.178208	-3.409744	2.184596
11	6	0	-8.985151	-4.130610	3.072869
12	6	0	-10.279531	-4.477746	2.689298
13	6	0	-10.736051	-4.097192	1.428948
14	6	0	-9.877258	-3.365491	0.609921
15	7	0	-8.633866	-3.027499	0.974806
16	1	0	-8.625229	-4.394447	4.060263
17	1	0	-10.920926	-5.030551	3.368058
18	1	0	-11.733286	-4.352444	1.087593
19	1	0	-10.190971	-3.036253	-0.376462
20	6	0	-4.175273	-2.250111	3.123542
21	6	0	-4.642730	3.486102	3.797985
22	6	0	-3.079451	4.230381	2.159399
23	7	0	-3.819937	5.306994	1.875041
24	6	0	-5.419313	4.597171	3.499911
25	6	0	-4.979192	5.513176	2.534199
26	1	0	-4.964810	2.784367	4.560980
27	6	0	-5.773402	6.723574	2.189328
28	1	0	-2.171319	4.113409	1.575874
29	1	0	-6.365361	4.745099	4.006303
30	6	0	-6.706095	7.267799	3.080245
31	6	0	-7.437979	8.391085	2.698686
32	6	0	-7.221114	8.942975	1.437597
33	6	0	-6.261710	8.352884	0.615928
34	7	0	-5.554474	7.275216	0.978916
35	1	0	-6.842762	6.844113	4.068162
36	1	0	-8.159957	8.830025	3.379701
37	1	0	-7.773222	9.812379	1.097824
38	1	0	-6.047760	8.753272	-0.370851
39	6	0	-3.429416	3.276529	3.124603
40	6	0	1.885681	5.492919	3.795387
41	6	0	3.074063	4.234751	2.156252
42	7	0	3.871535	5.270052	1.873169
43	6	0	2.705157	6.573235	3.498578
44	6	0	3.712683	6.435909	2.533496
45	1	0	1.118531	5.583785	4.558001
46	6	0	4.622299	7.562460	2.189743

197	7	0	6.339496	-6.644726	-1.179948
198	7	0	8.210224	-4.115011	1.179791
199	6	0	9.405797	-4.598751	0.818402
200	6	0	10.500174	-4.638179	1.680975
201	6	0	10.332089	-4.164407	2.980418
202	6	0	9.089888	-3.659590	3.360802
203	6	0	9.146734	3.513460	-3.369545
204	6	0	10.396782	4.000260	-2.991332
205	6	0	10.574786	4.468778	-1.691295
206	6	0	9.482195	4.442621	-0.825949
207	7	0	8.279061	3.976383	-1.185277
208	6	0	6.290889	7.512125	3.360825
209	6	0	7.157207	8.535733	2.980843
210	6	0	7.663814	8.545816	1.682952
211	6	0	7.289301	7.515922	0.821321
212	7	0	6.457528	6.530218	1.182368
213	1	0	-9.132679	2.731307	-4.374919
214	1	0	-11.428748	3.365164	-3.686043
215	1	0	-12.170361	2.873896	-1.334040
216	1	0	-10.554845	1.748575	0.206341
217	1	0	-10.585429	-1.551240	-0.206881
218	1	0	-12.221329	-2.648709	1.332156
219	1	0	-11.489054	-3.155543	3.683845
220	1	0	-9.181690	-2.564791	4.373228
221	1	0	-5.417342	-7.841954	-4.377280
222	1	0	-6.727849	-9.832430	-3.692953
223	1	0	-6.489973	-10.694122	-1.342635
224	1	0	-4.922234	-9.506765	0.200852
225	1	0	-1.797032	-10.553364	-0.200229
226	1	0	-1.258492	-12.445397	1.342011
227	1	0	-0.547705	-11.901367	3.691903
228	1	0	-0.395240	-9.523231	4.376756
229	1	0	5.778353	-7.579840	-4.372903
230	1	0	7.267512	-9.439904	-3.687891
231	1	0	8.168480	-9.473317	-1.340477
232	1	0	7.529569	-7.610368	0.199549
233	1	0	9.484127	-4.965653	-0.200767
234	1	0	11.449966	-5.036854	1.341862
235	1	0	11.150847	-4.194108	3.692076
236	1	0	8.935285	-3.317015	4.377025
237	1	0	8.984598	3.175289	-4.386059
238	1	0	11.214010	4.020348	-3.705082
239	1	0	11.530878	4.853563	-1.353842
240	1	0	9.568109	4.806404	0.193746
241	1	0	5.913846	7.473725	4.375870
242	1	0	7.437093	9.306485	3.691692
243	1	0	8.338551	9.324359	1.344352
244	1	0	7.665103	7.474446	-0.196782
245	1	0	3.618217	-3.427745	1.695975

[biconvex-(*M*,*M*,*M*)-Ag_5**L**$_2$]$^{5+}$:

Center Number	Atomic Number	Atomic Type	Coordinates (Angstroms) X	Y	Z
1	47	0	5.315542	5.347066	0.000014
2	6	0	1.699296	-5.556006	-3.802854
3	6	0	1.670430	-6.911288	-3.504673
4	6	0	0.775926	-7.390637	-2.537497
5	7	0	-0.036535	-6.539621	-1.876847
6	6	0	0.001003	-5.233543	-2.161395
7	1	0	2.372286	-5.180071	-4.567095
8	1	0	2.345778	-7.589393	-4.012032
9	1	0	-0.662095	-4.602888	-1.576946
10	6	0	0.702474	-8.836355	-2.192285
11	6	0	1.133003	-9.826459	-3.083513
12	6	0	1.061641	-11.165040	-2.701329
13	6	0	0.562746	-11.482146	-1.439455
14	6	0	0.137337	-10.439318	-0.617639
15	7	0	0.201528	-9.152107	-0.981175
16	1	0	1.491970	-9.565331	-4.072017
17	1	0	1.384768	-11.945682	-3.382415
18	1	0	0.496417	-12.509712	-1.099080
19	1	0	-0.270369	-10.636136	0.369703
20	6	0	0.845128	-4.670457	-3.127944
21	6	0	-4.755771	-3.333801	-3.798282
22	6	0	-4.972205	-1.620695	-2.154801
23	7	0	-6.225890	-1.988615	-1.870140
24	6	0	-6.053491	-3.725303	-3.499775
25	6	0	-6.785012	-3.023305	-2.531625
26	1	0	-4.190761	-3.857017	-4.563370
27	6	0	-8.182580	-3.399887	-2.185799
28	1	0	-4.576670	-0.796441	-1.569002
29	1	0	-6.489944	-4.576558	-4.007943
30	6	0	-8.991755	-4.115557	-3.076258
31	6	0	-10.286780	-4.460941	-2.693402
32	6	0	-10.741997	-4.083207	-1.431669
33	6	0	-9.881410	-3.355471	-0.610921
34	7	0	-8.637277	-3.019564	-0.975046
35	1	0	-8.632873	-4.376943	-4.064707
36	1	0	-10.929664	-5.010081	-3.373711
37	1	0	-11.739584	-4.337381	-1.090542
38	1	0	-10.194263	-3.027423	0.376106
39	6	0	-4.176934	-2.248263	-3.123064
40	6	0	-4.640782	3.488013	-3.799845
41	6	0	-3.076204	4.228868	-2.160887
42	7	0	-3.815417	5.306097	-1.875503
43	6	0	-5.415820	4.599972	-3.500998
44	6	0	-4.974386	5.514409	-2.534485
45	1	0	-4.964001	2.787184	-4.563222
46	6	0	-5.767141	6.725197	-2.188036

47	1	0	3.240971	3.335456	1.571088
48	1	0	2.554921	7.518782	4.005212
49	6	0	4.852026	8.618215	3.079950
50	6	0	5.697478	9.659014	2.699282
51	6	0	6.292213	9.620445	1.439692
52	6	0	6.026898	8.525266	0.618693
53	7	0	5.217457	7.521806	0.980845
54	1	0	4.404285	8.619605	4.066713
55	1	0	5.891892	10.481809	3.379643
56	1	0	6.950973	10.412349	1.100465
57	1	0	6.476150	8.443725	-0.366869
58	6	0	2.058903	4.274156	3.121512
59	6	0	1.705688	-5.552315	3.797539
60	6	0	1.678297	-6.907891	3.500090
61	6	0	0.783772	-7.388480	2.533529
62	7	0	-0.029957	-6.538671	1.873100
63	6	0	0.005742	-5.232512	2.157144
64	1	0	2.378934	-5.174940	4.560896
65	1	0	2.354731	-7.585005	4.007377
66	1	0	-0.658599	-4.603074	1.572757
67	6	0	0.711516	-8.834289	2.188403
68	6	0	1.141729	-9.823793	3.080354
69	6	0	1.072005	-11.162551	2.698279
70	6	0	0.574962	-11.480222	1.435786
71	6	0	0.149851	-10.437887	0.613199
72	7	0	0.212448	-9.150562	0.976611
73	1	0	1.499550	-9.561960	4.069112
74	1	0	1.395076	-11.942865	3.379777
75	1	0	0.510228	-12.507866	1.095331
76	1	0	-0.255736	-10.635173	-0.374904
77	6	0	0.849558	-4.668109	3.123033
78	6	0	5.808443	-0.093893	3.796469
79	6	0	7.089387	-0.537649	3.498308
80	6	0	7.270589	-1.536570	2.531562
81	7	0	6.211032	-2.048890	1.871488
82	6	0	4.979597	-1.612477	2.156553
83	1	0	5.657367	0.662713	4.560077
84	1	0	7.942186	-0.102681	4.005135
85	1	0	4.175727	-2.049981	1.572325
86	6	0	8.623883	-2.050385	2.185717
87	6	0	9.698434	-1.945942	3.076976
88	6	0	10.950297	-2.424797	2.694152
89	6	0	11.098652	-2.995404	1.431497
90	6	0	9.975566	-3.078770	0.609648
91	7	0	8.770414	-2.622495	0.973855
92	1	0	9.560423	-1.525004	4.065839
93	1	0	11.792595	-2.357901	3.375143
94	1	0	12.056109	-3.373797	1.090547
95	1	0	10.037606	-3.525293	-0.378508
96	6	0	4.703067	-0.635863	3.122591
97	6	0	3.079003	1.206133	3.420317
98	6	0	3.324238	-0.175425	3.409661
99	6	0	2.244920	-1.133655	3.659823
100	6	0	1.077010	-0.536943	4.082338
101	6	0	1.772297	1.782766	3.658494
102	6	0	0.844347	0.856452	4.081999
103	6	0	-0.552756	1.065679	4.082621
104	6	0	1.194419	3.105419	3.408165
105	6	0	-0.195219	3.299487	3.421375
106	6	0	-1.147194	2.234868	3.660400
107	6	0	-1.183464	-0.198432	4.082997
108	6	0	-2.479255	-0.402320	3.661178
109	6	0	-3.197579	0.832165	3.423029
110	6	0	-2.583861	2.094066	3.411317
111	6	0	-0.176169	-1.188896	4.082793
112	6	0	-0.382659	-2.484127	3.660499
113	6	0	-1.778795	-2.785796	3.422456
114	6	0	-2.789173	-1.812109	3.410881
115	6	0	2.100455	-2.554643	3.421632
116	6	0	0.862172	-3.214535	3.409958
117	1	0	-2.074761	-3.802066	3.183416
118	1	0	-4.255838	0.799382	3.185279
119	1	0	-0.553657	4.295717	3.183696
120	1	0	3.915661	1.854310	3.181176
121	1	0	2.975712	-3.149986	3.182923
122	6	0	5.478902	1.920382	-3.801655
123	6	0	6.554833	2.745395	-3.504087
124	6	0	6.412250	3.750430	-2.537160
125	7	0	5.245502	3.902854	-1.876889
126	6	0	4.214335	3.100550	-2.161370
127	1	0	5.573655	1.154808	-4.565376
128	1	0	7.500851	2.601868	-4.011833
129	1	0	3.313366	3.262819	-1.577473
130	6	0	7.534657	4.664564	-2.191726
131	6	0	8.586023	4.905885	-3.084084
132	6	0	9.623169	5.754976	-2.701445
133	6	0	9.585536	6.341354	-1.437832
134	6	0	8.494841	6.064549	-0.614796
135	7	0	7.494655	5.251928	-0.978962
136	1	0	8.586134	4.464437	-4.073627
137	1	0	10.442438	5.958545	-3.383387
138	1	0	10.375113	7.002023	-1.096905
139	1	0	8.414257	6.506421	0.374133
140	6	0	4.259543	2.086112	-3.126958
141	6	0	-0.134536	5.807131	-3.796847
142	6	0	-1.647936	4.966911	-2.157865
143	7	0	-2.093386	6.194972	-1.872749
144	6	0	-0.587375	7.084726	-3.498153
145	6	0	-1.588145	7.258404	-2.531857
146	1	0	0.623125	5.661805	-4.560499
147	6	0	-2.111596	8.607778	-2.185991
148	1	0	-2.080565	4.159796	-1.574464
149	1	0	-0.158365	7.940732	-4.004656

47	1	0	-2.168307	4.110388	-1.577183
48	1	0	-6.361822	4.749399	-4.007165
49	6	0	-6.699681	7.270740	-3.078235
50	6	0	-7.431207	8.393838	-2.695454
51	6	0	-7.214024	8.944175	-1.433759
52	6	0	-6.254465	8.353030	-0.613005
53	7	0	-5.547621	7.275577	-0.977153
54	1	0	-6.836525	6.848003	-4.066549
55	1	0	-8.153352	8.833627	-3.375733
56	1	0	-7.766170	9.813052	-1.092698
57	1	0	-6.040222	8.752159	0.374135
58	6	0	-3.427609	3.276307	-3.126829
59	6	0	5.806787	-0.095656	-3.798134
60	6	0	7.087701	-0.538338	-3.498739
61	6	0	7.268729	-1.538589	-2.533383
62	7	0	6.208864	-2.053516	-1.875797
63	6	0	4.977279	-1.617716	-2.161702
64	1	0	5.655978	0.662130	-4.560610
65	1	0	7.940808	-0.101664	-4.003578
66	1	0	4.173007	-2.057245	-1.579579
67	6	0	8.622161	-2.050642	-2.186258
68	6	0	9.698610	-1.941261	-3.074628
69	6	0	10.950567	-2.418340	-2.689935
70	6	0	11.097105	-2.992132	-1.428509
71	6	0	9.972187	-3.080248	-0.609642
72	7	0	8.766909	-2.625856	-0.975743
73	1	0	9.561987	-1.517878	-4.062663
74	1	0	11.794369	-2.347653	-3.368683
75	1	0	12.054608	-3.369290	-1.086325
76	1	0	10.032999	-3.529063	0.377566
77	6	0	4.701056	-0.639846	-3.126729
78	6	0	1.883889	5.490432	-3.797668
79	6	0	2.701166	6.571631	-3.498236
80	6	0	3.708182	6.434205	-2.532689
81	7	0	3.869715	5.266940	-1.875226
82	6	0	3.074409	4.230607	-2.161217
83	1	0	1.116528	5.581452	-4.560051
84	1	0	2.549812	7.517877	-4.003318
85	1	0	3.243848	3.329825	-1.579094
86	6	0	4.614531	7.562421	-2.185792
87	6	0	4.844745	8.618647	-3.075281
88	6	0	5.687444	9.660838	-2.692248
89	6	0	6.279005	9.623031	-1.431099
90	6	0	6.013412	8.527398	-0.610871
91	7	0	5.206458	7.522662	-0.975187
92	1	0	4.399558	8.619110	-4.063182
93	1	0	5.882300	10.484052	-3.371983
94	1	0	6.935388	10.416106	-1.090023
95	1	0	6.460011	8.446707	0.375925
96	6	0	2.059058	4.270675	-3.126202
97	6	0	-0.194904	3.296850	-3.426530
98	6	0	1.194734	3.102203	-3.414519
99	6	0	1.771806	1.779421	-3.665666
100	6	0	0.843252	0.853706	-4.089228
101	6	0	-1.147418	2.232909	-3.665986
102	6	0	-0.553771	1.063655	-4.089324
103	6	0	-1.185071	-0.200134	-4.088818
104	6	0	-2.583670	2.092939	-3.414848
105	6	0	-3.197798	0.831331	-3.425294
106	6	0	-2.480131	-0.403227	-3.664229
107	6	0	-0.178344	-1.191229	-4.089131
108	6	0	-0.385377	-2.485798	-3.665014
109	6	0	-1.781140	-2.786497	-3.424006
110	6	0	-2.790699	-1.812195	-3.412086
111	6	0	1.075269	-0.539904	-4.089410
112	6	0	2.242753	-1.137057	-3.666197
113	6	0	2.097718	-2.557962	-3.427806
114	6	0	0.858968	-3.217049	-3.415418
115	6	0	3.078056	1.202276	-3.426596
116	6	0	3.322485	-0.179511	-3.415175
117	1	0	2.972897	-3.153601	-3.189667
118	1	0	-2.077441	-3.802469	-3.184194
119	1	0	-4.255455	0.798779	-3.185139
120	1	0	-0.552778	4.292818	-3.186885
121	1	0	3.914859	1.850360	-3.187249
122	6	0	-0.129278	5.807468	3.796163
123	6	0	-0.581236	7.085066	3.496351
124	6	0	-1.582539	7.258573	2.530642
125	7	0	-2.089373	6.194803	1.873127
126	6	0	-1.644564	4.966542	2.159285
127	1	0	0.628981	5.662317	4.559245
128	1	0	-0.151158	7.941425	4.001366
129	1	0	-2.078001	4.158925	1.577186
130	6	0	-2.105042	8.608030	2.183564
131	6	0	-2.003825	9.685133	3.072089
132	6	0	-2.491278	10.933282	2.688023
133	6	0	-3.067242	11.075456	1.427117
134	6	0	-3.146613	9.950097	0.607935
135	7	0	-2.681927	8.748471	0.973298
136	1	0	-1.578765	9.551789	4.059865
137	1	0	-2.427051	11.777442	3.366973
138	1	0	-3.452636	12.029901	1.085568
139	1	0	-3.597051	10.007556	-0.378781
140	6	0	-0.664920	4.697732	3.124577
141	6	0	-5.560258	1.671233	3.799389
142	6	0	-5.230819	-0.027585	2.159748
143	7	0	-6.536945	-0.071178	1.875997
144	6	0	-6.915404	1.636346	3.501642
145	6	0	-7.391150	0.738635	2.535758
146	1	0	-5.186989	2.346619	4.562835
147	6	0	-8.836607	0.658956	2.191119
148	1	0	-4.597553	-0.688674	1.575892
149	1	0	-7.596212	2.309379	4.008457

150	6	0	-2.011078	9.684108	-3.075546
151	6	0	-2.499466	10.932384	-2.693207
152	6	0	-3.076170	11.075500	-1.432781
153	6	0	-3.155333	9.950856	-0.612605
154	7	0	-2.689352	8.749134	-0.976272
155	1	0	-1.585900	9.549943	-4.063197
156	1	0	-2.435610	11.775779	-3.373139
157	1	0	-3.462412	12.029966	-1.092264
158	1	0	-3.607019	10.008737	0.373515
159	6	0	-0.668768	4.697639	-3.123693
160	6	0	-5.565083	1.669479	-3.797779
161	6	0	-5.234048	-0.028205	-2.157477
162	7	0	-6.539869	-0.072049	-1.873069
163	6	0	-6.920293	1.633724	-3.499800
164	6	0	-7.395044	0.736643	-2.532758
165	1	0	-5.192482	2.344371	-4.561991
166	6	0	-8.840312	0.655433	-2.186769
167	1	0	-4.600568	-0.688567	-1.573108
168	1	0	-7.601756	2.305524	-4.007467
169	6	0	-9.832812	1.079780	-3.078196
170	6	0	-11.171021	1.001143	-2.696159
171	6	0	-11.485414	0.501337	-1.433957
172	6	0	-10.440260	0.082378	-0.611767
173	7	0	-9.153361	0.153697	-0.975161
174	1	0	-9.573654	1.439309	-4.067030
175	1	0	-11.953498	1.319257	-3.377501
176	1	0	-12.512633	0.429339	-1.093690
177	1	0	-10.634824	-0.325347	0.375966
178	6	0	-4.675102	0.819133	-3.123564
179	6	0	3.515055	-4.620336	-3.794970
180	6	0	4.631995	-5.388615	-3.496935
181	6	0	5.545815	-4.940462	-2.532947
182	7	0	5.332149	-3.781705	-1.875447
183	6	0	4.250178	-3.049197	-2.159918
184	1	0	2.814555	-4.948803	-4.556410
185	1	0	4.785873	-6.334464	-4.001871
186	1	0	4.127736	-2.140733	-1.577897
187	6	0	6.762260	-5.725057	-2.187765
188	6	0	7.311560	-6.657263	-3.076147
189	6	0	8.439986	-7.380493	-2.693258
190	6	0	8.991892	-7.155726	-1.433576
191	6	0	8.396656	-6.197200	-0.614699
192	7	0	7.314134	-5.498161	-0.979025
193	1	0	6.887771	-6.800018	-4.063134
194	1	0	8.882690	-8.102201	-3.372114
195	1	0	9.864870	-7.701499	-1.092719
196	1	0	8.796970	-5.977135	0.370738
197	6	0	3.297772	-3.407425	-3.123428
198	6	0	-3.309320	-4.772791	-3.793358
199	6	0	-3.694624	-6.072431	-3.495096
200	6	0	-2.986542	-6.802344	-2.530167
201	7	0	-1.951690	-6.239907	-1.871700
202	6	0	-1.589689	-4.984401	-2.156260
203	1	0	-3.837547	-4.208825	-4.555794
204	1	0	-4.545970	-6.511527	-4.000835
205	1	0	-0.764848	-4.586488	-1.572907
206	6	0	-3.355818	-8.201923	-2.184755
207	6	0	-4.070281	-9.013060	-3.074356
208	6	0	-4.408928	-10.310065	-2.692202
209	6	0	-4.026269	-10.764982	-1.431764
210	6	0	-3.300726	-9.901990	-0.611609
211	7	0	-2.971108	-8.656093	-0.975186
212	1	0	-4.335836	-8.654216	-4.061691
213	1	0	-4.956887	-10.954648	-3.371865
214	1	0	-4.275107	-11.764117	-1.091234
215	1	0	-2.969524	-10.214127	0.374603
216	6	0	-2.223699	-4.190332	-3.121267
217	6	0	-2.775370	-1.797544	-3.420890
218	6	0	-1.795213	-2.801558	-3.409443
219	6	0	-0.387691	-2.482601	-3.660241
220	6	0	-0.192322	-1.185878	-4.083250
221	6	0	-2.483018	-0.399660	-3.660229
222	6	0	-1.189354	-0.185062	-4.083241
223	6	0	-0.545655	1.072416	-4.084131
224	6	0	-3.221660	0.840544	-3.410854
225	6	0	-2.569746	2.083091	-3.423696
226	6	0	-1.149818	2.236547	-3.661834
227	6	0	0.849186	0.848886	-4.084483
228	6	0	1.769517	1.783272	-3.662013
229	6	0	1.184434	3.086150	-3.422831
230	6	0	-0.198846	3.322157	-3.411519
231	6	0	1.067526	-0.546845	-4.083911
232	6	0	2.240675	-1.133230	-3.661549
233	6	0	3.299137	-0.174102	-3.424043
234	6	0	3.096111	1.214313	-3.412786
235	6	0	0.851605	-3.192703	-3.422068
236	6	0	2.109308	-2.570440	-3.410527
237	1	0	4.297645	-0.525841	-3.185826
238	1	0	1.827174	3.927040	-3.183477
239	1	0	-3.170600	2.954770	-3.186018
240	1	0	-3.789696	-2.099927	-3.181829
241	1	0	0.826259	-4.251009	-3.183655
242	47	0	5.323331	5.346870	0.002608
243	47	0	-3.450569	6.712139	0.002817
244	47	0	-7.446949	-1.199314	0.002945
245	47	0	-1.153496	-7.453128	-0.000526

[biconcave-(P,M,P)-Ag_5**L**$_2$]$^{5+}$:

Center Number	Atomic Number	Atomic Type	Coordinates (Angstroms) X	Y	Z

150	6	0	-9.828036	1.086695	3.082212
151	6	0	-11.166431	1.009947	2.700495
152	6	0	-11.481988	0.508859	1.439083
153	6	0	-10.437797	0.086566	0.617410
154	7	0	-9.150767	0.155734	0.980567
155	1	0	-9.567928	1.447720	4.070263
156	1	0	-11.948086	1.330937	3.381426
157	1	0	-12.509368	0.438617	1.098930
158	1	0	-10.633255	-0.322321	-0.369684
159	6	0	-4.671162	0.819976	3.125302
160	6	0	-3.303037	-4.772867	3.799659
161	6	0	-1.588279	-4.983264	2.157141
162	7	0	-1.950110	-6.239062	1.873610
163	6	0	-3.688283	-6.072762	3.502417
164	6	0	-2.982651	-6.802074	2.535211
165	1	0	-3.829114	-4.209497	4.563984
166	6	0	-3.352360	-8.201970	2.191170
167	1	0	-0.765782	-4.584491	1.571119
168	1	0	-4.537655	-6.512775	4.010705
169	6	0	-4.062234	-9.013905	3.083718
170	6	0	-4.401530	-10.311113	2.702879
171	6	0	-4.023880	-10.765622	1.440871
172	6	0	-3.302378	-9.902019	0.617787
173	7	0	-2.972421	-8.655739	0.979933
174	1	0	-4.323499	-8.655396	4.072317
175	1	0	-4.945887	-10.956219	3.384933
176	1	0	-4.273356	-11.764930	1.101333
177	1	0	-2.974564	-10.214232	-0.369539
178	6	0	-2.220062	-4.189646	3.124031
179	6	0	5.483050	1.919313	3.798610
180	6	0	6.558933	2.743419	3.498722
181	6	0	6.415198	3.748544	2.532171
182	7	0	5.247124	3.901836	1.874200
183	6	0	4.215821	3.100237	2.160847
184	1	0	5.578981	1.152958	4.561426
185	1	0	7.506104	2.598679	4.004011
186	1	0	3.313958	3.262994	1.578497
187	6	0	7.537617	4.661520	2.184314
188	6	0	8.592615	4.899055	3.073336
189	6	0	9.629731	5.747388	2.688898
190	6	0	9.588278	6.336889	1.426913
191	6	0	8.494036	6.063683	0.607304
192	7	0	7.494073	5.251603	0.973008
193	1	0	8.595866	4.455237	4.061845
194	1	0	10.451899	5.948037	3.368217
195	1	0	10.377561	6.997239	1.084694
196	1	0	8.410336	6.508190	-0.380173
197	6	0	4.262356	2.086052	3.126708
198	6	0	3.521762	-4.617082	3.801110
199	6	0	4.639058	-5.384619	3.502678
200	6	0	5.550139	-4.937677	2.535527
201	7	0	5.333408	-3.780841	1.875398
202	6	0	4.251324	-3.048771	2.160662
203	1	0	2.823209	-4.944572	4.564765
204	1	0	4.795174	-6.329051	4.009666
205	1	0	4.126541	-2.141918	1.576644
206	6	0	6.766475	-5.722198	2.189470
207	6	0	7.319315	-6.649802	3.080385
208	6	0	8.447132	-7.373983	2.697479
209	6	0	8.994853	-7.154531	1.435034
210	6	0	8.396378	-6.200144	0.613605
211	7	0	7.314450	-5.500198	0.977919
212	1	0	6.898410	-6.788451	4.069227
213	1	0	8.892458	-8.092290	3.378222
214	1	0	9.867055	-7.701289	1.093762
215	1	0	8.793115	-5.984715	-0.374239
216	6	0	3.301527	-3.405977	3.127147
217	6	0	0.855004	-3.191623	3.425188
218	6	0	2.112770	-2.569646	3.415199
219	6	0	2.243802	-1.132521	3.666029
220	6	0	1.070808	-0.546074	4.088739
221	6	0	-0.384182	-2.481563	3.663424
222	6	0	-0.189088	-1.185169	4.087783
223	6	0	-1.186076	-0.184299	4.087520
224	6	0	-1.791352	-2.800630	3.411126
225	6	0	-2.771601	-1.796657	3.422717
226	6	0	-2.479331	-0.399027	3.663153
227	6	0	-0.542314	1.073246	4.088095
228	6	0	-1.146337	2.237087	3.664653
229	6	0	-2.566133	2.083523	3.425817
230	6	0	-3.217886	0.840975	3.413177
231	6	0	0.852578	0.849734	4.088457
232	6	0	1.772985	1.783748	3.665257
233	6	0	1.187995	3.086454	3.424954
234	6	0	-0.195304	3.322409	3.413462
235	6	0	3.302273	-0.173877	3.427289
236	6	0	3.099335	1.214468	3.415047
237	1	0	1.830866	3.927112	3.185022
238	1	0	-3.167200	2.954967	3.187437
239	1	0	-3.785774	-2.098593	3.182703
240	1	0	0.829350	-4.249571	3.185403
241	1	0	4.300468	-0.526125	3.188366
242	47	0	-3.445958	6.706409	0.000028
243	47	0	-7.446841	-1.200887	0.003215
244	47	0	-1.159481	-7.452639	-0.001437
245	47	0	6.730740	-3.403397	-0.000744

[biconcave-(M,P,M)-Ag_5**L**$_2$]$^{5+}$:

Center Number	Atomic Number	Atomic Type	Coordinates (Angstroms) X	Y	Z

1	6	0	5.079549	2.178614	3.543879
2	6	0	6.131271	3.078142	3.658157
3	6	0	6.194467	4.183438	2.796710
4	7	0	5.230657	4.399676	1.879798
5	6	0	4.212476	3.541093	1.789096
6	1	0	5.027251	1.317619	4.202818
7	1	0	6.914764	2.906242	4.386714
8	1	0	3.468044	3.766242	1.031449
9	6	0	7.339805	5.130815	2.833960
10	6	0	8.070135	5.349844	4.006952
11	6	0	9.161021	6.216931	3.980212
12	6	0	9.498859	6.839460	2.780361
13	6	0	8.713361	6.581084	1.658775
14	7	0	7.657683	5.755292	1.680710
15	1	0	7.777551	4.871844	4.934691
16	1	0	9.733977	6.404236	4.882582
17	1	0	10.345692	7.513137	2.710463
18	1	0	8.939282	7.042357	0.702252
19	6	0	4.085024	2.386217	2.576056
20	6	0	-0.508275	5.481203	3.547090
21	6	0	-2.079633	5.081040	1.797948
22	7	0	-2.581086	6.314691	1.893191
23	6	0	-1.037646	6.759403	3.665655
24	6	0	-2.073160	7.162578	2.809596
25	1	0	0.297258	5.163861	4.201811
26	6	0	-2.619398	8.544714	2.852364
27	1	0	-2.527095	4.443670	1.041200
28	1	0	-0.628673	7.450151	4.393533
29	6	0	-2.592343	9.305842	4.025916
30	6	0	-3.081182	10.610867	4.004968
31	6	0	-3.580386	11.125115	2.810121
32	6	0	-3.586163	10.299494	1.687582
33	7	0	-3.124872	9.040977	1.703850
34	1	0	-2.219393	8.879344	4.949958
35	1	0	-3.074574	11.212730	4.907932
36	1	0	-3.961518	12.138187	2.744726
37	1	0	-3.964524	10.657736	0.735061
38	6	0	-1.017680	4.601399	2.580006
39	6	0	-5.380955	1.181974	3.561651
40	6	0	-5.488444	-0.418370	1.796585
41	7	0	-6.817082	-0.511116	1.889650
42	6	0	-6.760631	1.075476	3.678396
43	6	0	-7.465799	0.226783	2.812326
44	1	0	-4.829171	1.841873	4.223711
45	6	0	-8.949541	0.140342	2.848546
46	1	0	-5.021877	-1.034393	1.033793
47	1	0	-7.290319	1.671448	4.412267
48	6	0	-9.668844	0.389671	4.022263
49	6	0	-11.061287	0.335146	3.994032
50	6	0	-11.700550	0.038360	2.792175
51	6	0	-10.913204	-0.211671	1.669864
52	7	0	-9.573775	-0.169123	1.692925
53	1	0	-9.150940	0.598012	4.951376
54	1	0	-11.634953	0.518395	4.896783
55	1	0	-12.781753	-0.004654	2.721287
56	1	0	-11.367520	-0.445910	0.711970
57	6	0	-4.702817	0.434144	2.587127
58	6	0	3.662279	-4.163352	3.554869
59	6	0	4.840811	-4.888961	3.668414
60	6	0	5.911177	-4.611258	2.805384
61	7	0	5.821384	-3.626236	1.889870
62	6	0	4.692335	-2.919634	1.800134
63	1	0	2.827260	-4.377734	4.214471
64	1	0	4.917772	-5.687406	4.396951
65	1	0	4.678857	-2.141222	1.043439
66	6	0	7.161726	-5.414430	2.835571
67	6	0	7.597111	-6.049118	4.003790
68	6	0	8.755827	-6.822970	3.968608
69	6	0	9.447357	-6.948006	2.765459
70	6	0	8.957183	-6.273229	1.649026
71	7	0	7.848426	-5.520683	1.678925
72	1	0	7.055751	-5.921375	4.934128
73	1	0	9.112505	-7.316128	4.867056
74	1	0	10.347339	-7.548074	2.689191
75	1	0	9.462020	-6.342691	0.690267
76	6	0	3.554033	-3.153502	2.586585
77	6	0	-2.808796	-4.772976	3.556860
78	6	0	-3.141069	-6.116988	3.665068
79	6	0	-2.550985	-7.048340	2.797896
80	7	0	-1.639156	-6.659692	1.884706
81	6	0	-1.309742	-5.368745	1.800108
82	1	0	-3.268174	-4.045047	4.218273
83	1	0	-3.878654	-6.436553	4.391807
84	1	0	-0.572883	-5.116191	1.043765
85	6	0	-2.937712	-8.483528	2.820244
86	6	0	-3.407874	-9.097231	3.986124
87	6	0	-3.795274	-10.435468	3.944135
88	6	0	-3.709024	-11.125225	2.736587
89	6	0	-3.216956	-10.447394	1.622781
90	7	0	-2.834530	-9.163266	1.659119
91	1	0	-3.447317	-8.547772	4.919645
92	1	0	-4.155003	-10.929871	4.840688
93	1	0	-4.009325	-12.163987	2.654983
94	1	0	-3.132810	-10.944032	0.660923
95	6	0	-1.880048	-4.358818	2.589926
96	6	0	-2.615381	-2.005945	2.325863
97	6	0	-1.567701	-2.938743	2.319647
98	6	0	-0.193364	-2.518520	2.039572
99	6	0	-0.093328	-1.218776	1.592304
100	6	0	-2.431559	-0.599925	2.036846
101	6	0	-1.163762	-0.294255	1.590978
102	6	0	-0.615366	1.009303	1.588379

1	6	0	-5.273978	-1.627629	-3.543506
2	6	0	-6.414203	-2.411415	-3.660851
3	6	0	-6.594896	-3.504815	-2.800906
4	7	0	-5.661300	-3.821795	-1.881987
5	6	0	-4.558936	-3.074737	-1.787856
6	1	0	-5.130171	-0.776617	-4.201827
7	1	0	-7.173696	-2.157444	-4.390805
8	1	0	-3.844262	-3.377301	-1.028501
9	6	0	-7.833325	-4.326550	-2.841282
10	6	0	-8.581131	-4.466003	-4.015448
11	6	0	-9.756652	-5.214421	-3.991491
12	6	0	-10.159101	-5.800322	-2.793137
13	6	0	-9.352171	-5.627617	-1.670305
14	7	0	-8.216019	-4.916428	-1.689528
15	1	0	-8.239007	-4.019623	-4.941987
16	1	0	-10.344951	-5.339239	-4.894795
17	1	0	-11.071776	-6.381951	-2.725382
18	1	0	-9.626119	-6.064970	-0.715068
19	6	0	-4.308983	-1.939151	-2.573623
20	6	0	-0.068314	-5.499583	-3.544594
21	6	0	1.533784	-5.271564	-1.792593
22	7	0	1.901450	-6.551348	-1.889580
23	6	0	0.322551	-6.826535	-3.665085
24	6	0	1.307899	-7.338965	-2.808273
25	1	0	-0.834570	-5.097563	-4.199992
26	6	0	1.704527	-8.771174	-2.852241
27	1	0	2.044983	-4.687012	-1.033586
28	1	0	-0.156406	-7.468516	-4.394880
29	6	0	1.602293	-9.522990	-4.027664
30	6	0	1.950415	-10.872408	-4.007681
31	6	0	2.387148	-11.438803	-2.811939
32	6	0	2.475235	-10.620556	-1.687449
33	7	0	2.149603	-9.320279	-1.702775
34	1	0	1.280869	-9.057688	-4.952329
35	1	0	1.884305	-11.468502	-4.912076
36	1	0	2.658711	-12.486626	-2.747324
37	1	0	2.809367	-11.018599	-0.734178
38	6	0	0.529971	-4.680445	-2.575070
39	6	0	5.227713	-1.742741	-3.559029
40	6	0	5.506699	-0.159604	-1.797388
41	7	0	6.837548	-0.208928	-1.892005
42	6	0	6.610751	-1.783604	-3.677628
43	6	0	7.403186	-1.013487	-2.813647
44	1	0	4.608177	-2.341825	-4.218997
45	6	0	8.887691	-1.085482	-2.850283
46	1	0	5.109376	0.504175	-1.035447
47	1	0	7.073055	-2.433951	-4.410802
48	6	0	9.576722	-1.409417	-4.023947
49	6	0	10.967156	-1.502196	-3.995441
50	6	0	11.634054	-1.274584	-2.793473
51	6	0	10.877403	-0.943035	-1.671156
52	7	0	9.540960	-0.844058	-1.694515
53	1	0	9.039953	-1.561609	-4.953255
54	1	0	11.518435	-1.744902	-4.898114
55	1	0	12.713744	-1.346039	-2.722473
56	1	0	11.353713	-0.758389	-0.713088
57	6	0	4.633973	-0.925650	-2.585003
58	6	0	-3.198824	4.527989	-3.551729
59	6	0	-4.295106	5.372709	-3.665762
60	6	0	-5.389334	5.208011	-2.803729
61	7	0	-5.403662	4.218570	-1.888688
62	6	0	-4.354412	3.398175	-1.798156
63	1	0	-2.345542	4.654332	-4.210708
64	1	0	-4.287693	6.175117	-4.393964
65	1	0	-4.422936	2.622301	-1.041839
66	6	0	-6.549423	6.137128	-2.834991
67	6	0	-6.915128	6.813066	-4.003988
68	6	0	-7.986665	7.703758	-3.970512
69	6	0	-8.662280	7.901403	-2.768089
70	6	0	-8.246195	7.179998	-1.650734
71	7	0	-7.222238	6.315517	-1.678954
72	1	0	-6.389216	6.628863	-4.933717
73	1	0	-8.289013	8.230831	-4.869630
74	1	0	-9.494585	8.592424	-2.693052
75	1	0	-8.741821	7.302754	-0.692514
76	6	0	-3.197243	3.512243	-2.583576
77	6	0	3.297091	4.453827	-3.554407
78	6	0	3.766387	5.756157	-3.664980
79	6	0	3.277894	6.744459	-2.797732
80	7	0	2.333012	6.453014	-1.881867
81	6	0	1.871857	5.203160	-1.795127
82	1	0	3.676705	3.681832	-4.216405
83	1	0	4.531134	5.996967	-4.393982
84	1	0	1.114149	5.028859	-1.037285
85	6	0	3.810320	8.132197	-2.823210
86	6	0	4.336958	8.693396	-3.991408
87	6	0	4.859989	9.984752	-3.952240
88	6	0	4.849378	10.680611	-2.745142
89	6	0	4.294374	10.057766	-1.628804
90	7	0	3.781871	8.819726	-1.662438
91	1	0	4.316388	8.142110	-4.924474
92	1	0	5.265466	10.438905	-4.850558
93	1	0	5.255171	11.683059	-2.665745
94	1	0	4.265401	10.561024	-0.667150
95	6	0	2.332550	4.138930	-2.585118
96	6	0	2.816642	1.721337	-2.320070
97	6	0	1.873243	2.759536	-2.313824
98	6	0	0.462286	2.486459	-2.033733
99	6	0	0.225722	1.204729	-1.585869
100	6	0	2.485287	0.342505	-2.031163
101	6	0	1.192610	0.172376	-1.584455
102	6	0	0.509653	-1.066125	-1.581652

103	6	0	-3.256266	0.577461	2.314188
104	6	0	-2.692744	1.862085	2.319956
105	6	0	-1.298341	2.121255	2.031956
106	6	0	0.793865	0.890557	1.587697
107	6	0	1.639928	1.884915	2.029036
108	6	0	0.962113	3.131629	2.313389
109	6	0	-0.433881	3.270238	2.307388
110	6	0	1.116706	-0.486452	1.589838
111	6	0	2.323218	-0.982851	2.034053
112	6	0	3.298972	0.047898	2.317527
113	6	0	2.999494	1.418451	2.307256
114	6	0	1.087892	-3.127671	2.325882
115	6	0	2.299066	-2.419736	2.315269
116	1	0	4.311479	-0.237607	2.584323
117	1	0	1.547535	4.006590	2.577091
118	1	0	-3.342789	2.689234	2.586372
119	1	0	-3.602662	-2.368843	2.592968
120	1	0	1.128191	-4.178249	2.595285
121	6	0	-4.864621	-2.587639	-3.564787
122	6	0	-6.221973	-2.857266	-3.680717
123	6	0	-7.129161	-2.230660	-2.813519
124	7	0	-6.703039	-1.346391	-1.889945
125	6	0	-5.398599	-1.077530	-1.797638
126	1	0	-4.155749	-3.073784	-4.227659
127	1	0	-6.571796	-3.573737	-4.414595
128	1	0	-5.114626	-0.360021	-1.033683
129	6	0	-8.581193	-2.547637	-2.848242
130	6	0	-9.208870	-2.979869	-4.021443
131	6	0	-10.564531	-3.302245	-3.991191
132	6	0	-11.258024	-3.189870	-2.787962
133	6	0	-10.565234	-2.738297	-1.666330
134	7	0	-9.263728	-2.418805	-1.691394
135	1	0	-8.655632	-3.039755	-4.951606
136	1	0	-11.069289	-3.632043	-4.893458
137	1	0	-12.310763	-3.439590	-2.715519
138	1	0	-11.064179	-2.636029	-0.707484
139	6	0	-4.412649	-1.685483	-2.589770
140	6	0	-3.989616	3.841752	-3.559853
141	6	0	-2.712737	4.816563	-1.797110
142	7	0	-3.378001	5.970953	-1.881863
143	6	0	-4.671689	5.046495	-3.668295
144	6	0	-4.357624	6.101062	-2.798345
145	1	0	-4.232975	3.018341	-4.224008
146	6	0	-5.116731	7.379011	-2.821723
147	1	0	-1.937871	4.770662	-1.037934
148	1	0	-5.465391	5.156610	-4.397828
149	6	0	-5.731607	7.844183	-3.988974
150	6	0	-6.465244	9.028595	-3.948183
151	6	0	-6.571334	9.715272	-2.740462
152	6	0	-5.918076	9.194176	-1.625146
153	7	0	-5.203887	8.060538	-1.660353
154	1	0	-5.619161	7.305031	-4.922570
155	1	0	-6.942441	9.408375	-4.845789
156	1	0	-7.140487	10.634749	-2.659866
157	1	0	-5.973585	9.694383	-0.663057
158	6	0	-2.986779	3.691647	-2.589852
159	6	0	2.402423	4.999261	-3.554686
160	6	0	3.731512	4.075457	-1.803433
161	7	0	4.628197	5.060426	-1.892023
162	6	0	3.341603	6.015990	-3.666966
163	6	0	4.448081	6.035312	-2.805048
164	1	0	1.539659	4.982212	-4.213207
165	6	0	5.435584	7.146171	-2.833987
166	1	0	3.929198	3.320500	-1.048688
167	1	0	3.199783	6.807198	-4.393672
168	6	0	5.679825	7.878663	-4.000550
169	6	0	6.586021	8.937065	-3.964611
170	6	0	7.221232	9.240839	-2.762304
171	6	0	6.935059	8.455087	-1.647525
172	7	0	6.071295	7.430609	-1.678175
173	1	0	5.190301	7.612344	-4.930270
174	1	0	6.793295	9.511257	-4.861814
175	1	0	7.925418	10.061863	-2.685456
176	1	0	7.405038	8.655577	-0.689496
177	6	0	2.571277	3.995747	-2.588513
178	6	0	0.990067	-5.415272	-3.549302
179	6	0	0.826659	-6.789171	-3.666738
180	6	0	-0.060912	-7.456992	-2.810050
181	7	0	-0.779570	-6.777644	-1.894293
182	6	0	-0.631048	-5.454218	-1.800283
183	1	0	1.679785	-4.892251	-4.204276
184	1	0	1.407698	-7.343977	-4.393869
185	1	0	-1.233902	-4.961401	-1.043492
186	6	0	-0.212275	-8.935428	-2.851150
187	6	0	0.017147	-9.661970	-4.024539
188	6	0	-0.100177	-11.050566	-4.001995
189	6	0	-0.438861	-11.679604	-2.805771
190	6	0	-0.665330	-10.885316	-1.683476
191	7	0	-0.561936	-9.548895	-1.701243
192	1	0	0.258400	-9.151365	-4.949688
193	1	0	0.066953	-11.629026	-4.904809
194	1	0	-0.531369	-12.757958	-2.739096
195	1	0	-0.930292	-11.331759	-0.729911
196	6	0	0.261325	-4.705432	-2.582921
197	6	0	5.479921	-0.726408	-3.543394
198	6	0	6.735771	-1.308101	-3.656232
199	6	0	7.094288	-2.355351	-2.794585
200	7	0	6.223955	-2.823783	-1.878329
201	6	0	5.011535	-2.272339	-1.788867
202	1	0	5.197604	0.088358	-4.202717
203	1	0	7.444365	-0.930884	-4.384098
204	1	0	4.355212	-2.689757	-1.031301
205	6	0	8.453126	-2.957769	-2.830223

103	6	0	3.180873	-0.915325	-2.309498
104	6	0	2.485155	-2.133440	-2.313917
105	6	0	1.071363	-2.244057	-2.025157
106	6	0	-0.879245	-0.799248	-1.581090
107	6	0	-1.825455	-1.698775	-2.022757
108	6	0	-1.283104	-3.009996	-2.307061
109	6	0	0.090394	-3.295253	-2.301034
110	6	0	-1.054820	0.604126	-1.583901
111	6	0	-2.201904	1.224816	-2.029349
112	6	0	-3.280895	0.302976	-2.313123
113	6	0	-3.127945	-1.091450	-2.302242
114	6	0	-0.747438	3.227288	-2.320957
115	6	0	-2.026368	2.650845	-2.311394
116	1	0	-4.257211	0.693932	-2.580942
117	1	0	-1.957510	-3.817883	-2.572108
118	1	0	3.044226	-3.024452	-2.580892
119	1	0	3.836713	1.977671	-2.587534
120	1	0	-0.676613	4.276262	-2.590055
121	6	0	5.106170	2.063105	3.554308
122	6	0	6.484240	2.187507	3.672379
123	6	0	7.321386	1.464558	2.809777
124	7	0	6.805347	0.626087	1.889314
125	6	0	5.479918	0.496567	1.795207
126	1	0	4.451730	2.624884	4.213417
127	1	0	6.906742	2.865864	4.404246
128	1	0	5.123079	-0.191219	1.034498
129	6	0	8.798877	1.625220	2.847231
130	6	0	9.466347	1.989123	4.021631
131	6	0	10.848763	2.164812	3.994549
132	6	0	11.529172	1.978005	2.793114
133	6	0	10.794748	1.602365	1.669972
134	7	0	9.466501	1.423738	1.692005
135	1	0	8.920406	2.108432	4.950391
136	1	0	11.383717	2.439509	4.897877
137	1	0	12.602729	2.113830	2.723128
138	1	0	11.282142	1.446860	0.712324
139	6	0	4.562670	1.209650	2.582156
140	6	0	3.561662	-4.239880	3.555543
141	6	0	2.183308	-5.078738	1.799388
142	7	0	2.721988	-6.297155	1.886804
143	6	0	4.111545	-5.510198	3.666903
144	6	0	3.684681	-6.528108	2.801376
145	1	0	3.893263	-3.444874	4.216116
146	6	0	4.302438	-7.880031	2.827484
147	1	0	1.415030	-4.953420	1.042481
148	1	0	4.890763	-5.701842	4.395169
149	6	0	4.865321	-8.405949	3.995201
150	6	0	5.466704	-9.662784	3.956541
151	6	0	5.496012	-10.359952	2.750524
152	6	0	4.901400	-9.774014	1.634593
153	7	0	4.314039	-8.569750	1.667739
154	1	0	4.812931	-7.855709	4.927636
155	1	0	5.901387	-10.089753	4.854533
156	1	0	5.962310	-11.335768	2.671646
157	1	0	4.901218	-10.279474	0.673641
158	6	0	2.577790	-3.986869	2.587476
159	6	0	-2.921052	-4.718933	3.552057
160	6	0	-4.143227	-3.659421	1.799739
161	7	0	-5.139858	-4.543138	1.888089
162	6	0	-3.963442	-5.629588	3.664210
163	6	0	-5.065212	-5.531246	2.801648
164	1	0	-2.061845	-4.793520	4.211220
165	6	0	-6.166016	-6.529950	2.830705
166	1	0	-4.259103	-2.887895	1.044744
167	1	0	-3.907120	-6.431030	4.391368
168	6	0	-6.491038	-7.228172	3.998590
169	6	0	-7.506070	-8.182709	3.962871
170	6	0	-8.166992	-8.420002	2.759438
171	6	0	-7.794408	-7.673430	1.643250
172	7	0	-6.825195	-6.748144	1.673657
173	1	0	-5.978469	-7.013032	4.929145
174	1	0	-7.776706	-8.728209	4.861100
175	1	0	-8.955502	-9.160431	2.682732
176	1	0	-8.280430	-7.824993	0.684243
177	6	0	-2.981630	-3.703279	2.585691
178	6	0	-0.412075	5.486197	3.545930
179	6	0	-0.102169	6.834306	3.666608
180	6	0	0.851361	7.405302	2.810788
181	7	0	1.492052	6.655113	1.892405
182	6	0	1.202224	5.355524	1.795101
183	1	0	-1.153031	5.038598	4.200844
184	1	0	-0.619741	7.446188	4.395943
185	1	0	1.748541	4.802806	1.036732
186	6	0	1.160736	8.858812	2.855846
187	6	0	1.012082	9.602306	4.031619
188	6	0	1.277821	10.970338	4.012875
189	6	0	1.680447	11.562954	2.817874
190	6	0	1.819065	10.752275	1.693017
191	7	0	1.572917	9.434614	1.707192
192	1	0	0.718534	9.117689	4.955623
193	1	0	1.174867	11.560694	4.917588
194	1	0	1.888009	12.625360	2.754102
195	1	0	2.129301	11.170448	0.740299
196	6	0	0.235386	4.704730	2.576957
197	6	0	-5.373850	1.301114	3.543887
198	6	0	-6.560097	2.013879	3.658358
199	6	0	-6.805255	3.094112	2.797701
200	7	0	-5.890474	3.467577	1.881128
201	6	0	-4.744367	2.789272	1.789861
202	1	0	-5.179893	0.460364	4.202514
203	1	0	-7.304382	1.713847	4.386538
204	1	0	-4.047520	3.135121	1.032439
205	6	0	-8.091703	3.838535	2.834554

206	6	0	9.218895	-2.966762	-4.001027
207	6	0	10.503723	-3.506204	-3.972487
208	6	0	10.993938	-4.018377	-2.773052
209	6	0	10.164386	-3.986726	-1.653877
210	7	0	8.924628	-3.477488	-1.677565
211	1	0	8.810620	-2.582288	-4.928533
212	1	0	11.108676	-3.528027	-4.873145
213	1	0	11.991283	-4.437976	-2.701735
214	1	0	10.503759	-4.373739	-0.697937
215	6	0	4.577426	-1.195097	-2.576602
216	6	0	3.189360	0.844124	-2.319494
217	6	0	3.270733	-0.556368	-2.308910
218	6	0	2.087051	-1.372464	-2.032126
219	6	0	1.003836	-0.643148	-1.591780
220	6	0	1.971537	1.573480	-2.036835
221	6	0	0.943428	0.769870	-1.593533
222	6	0	-0.419217	1.148731	-1.595659
223	6	0	1.560656	2.950705	-2.317433
224	6	0	0.203484	3.306060	-2.326975
225	6	0	-0.866173	2.373884	-2.041274
226	6	0	-1.200616	-0.030158	-1.595119
227	6	0	-2.504166	-0.077393	-2.040002
228	6	0	-3.060408	1.227285	-2.327371
229	6	0	-2.303043	2.408154	-2.320527
230	6	0	-0.321101	-1.137526	-1.593164
231	6	0	-0.678899	-2.392538	-2.036542
232	6	0	-2.091665	-2.518580	-2.324492
233	6	0	-2.980810	-1.433507	-2.317767
234	6	0	1.770560	-2.755738	-2.316645
235	6	0	0.463697	-3.265859	-2.311149
236	1	0	-2.494938	-3.490154	-2.590987
237	1	0	-4.109159	1.310787	-2.593569
238	1	0	-0.040808	4.328920	-2.595287
239	1	0	4.087611	1.392102	-2.585503
240	1	0	2.570408	-3.440411	-2.579855
241	47	0	-1.481947	-8.217231	-0.010629
242	47	0	-3.653048	7.509210	0.014385
243	47	0	6.034930	5.782467	-0.007263
244	47	0	7.365168	-3.948920	0.004142
245	47	0	-8.265632	-1.138860	0.001107

[biconvex-(*P*,*M*,*P*)-$\mathrm{Hg_5}\mathbf{L}_2$]$^{10+}$:

Center Number	Atomic Number	Atomic Type	Coordinates (Angstroms) X	Y	Z
1	80	0	-8.521767	-0.008848	0.000189
2	80	0	-2.646871	8.102187	-0.001723
3	80	0	6.898884	5.017007	-0.002871
4	6	0	5.081911	-2.061621	-3.840216
5	6	0	6.359941	-2.603206	-3.836832
6	6	0	6.728471	-3.520647	-2.840190
7	7	0	5.846616	-3.835682	-1.864542
8	6	0	4.146783	-2.435226	-2.857207
9	6	0	4.604022	-3.322182	-1.876460
10	1	0	4.793841	-1.372814	-4.628414
11	1	0	7.066288	-2.310936	-4.603643
12	1	0	3.949161	-3.633363	-1.067396
13	6	0	-5.320480	-1.317800	-3.845351
14	6	0	-4.782381	-0.464660	-2.864360
15	6	0	-5.672566	-0.014276	-1.883216
16	7	0	-6.979902	-0.328409	-1.869896
17	6	0	-6.672891	-1.630033	-3.840559
18	6	0	-7.509438	-1.102402	-2.844408
19	1	0	-4.683504	-1.707580	-4.633421
20	6	0	-8.974150	-1.383077	-2.828998
21	6	0	-2.899057	4.651495	-3.843965
22	6	0	-3.618797	5.838309	-3.838821
23	6	0	-3.380255	6.797019	-2.841573
24	7	0	-2.478378	6.535951	-1.868162
25	6	0	-1.920741	4.406302	-2.862618
26	6	0	-1.770839	5.392667	-1.881699
27	1	0	-3.070255	3.924821	-4.632267
28	1	0	-4.363856	6.014633	-4.604473
29	1	0	-1.056920	5.261735	-1.073362
30	6	0	3.526298	4.198922	-3.836793
31	6	0	4.433934	5.249057	-3.832712
32	6	0	5.424067	5.314054	-2.839703
33	7	0	5.457205	4.373535	-1.868512
34	6	0	3.598287	3.190269	-2.858133
35	6	0	4.587168	3.348646	-1.881088
36	1	0	2.779518	4.139558	-4.622622
37	1	0	4.369789	6.014338	-4.595975
38	6	0	-1.077913	3.181680	-2.931539
39	6	0	0.323564	3.318258	-2.912955
40	6	0	1.245430	2.207332	-3.107564
41	6	0	0.594955	1.043080	-3.435034
42	6	0	-1.712033	1.864979	-3.110968
43	6	0	-0.805686	0.885986	-3.436496
44	6	0	-1.089107	-0.494558	-3.436464
45	6	0	-3.053509	1.331772	-2.916718
46	6	0	-3.356777	-0.043326	-2.933865
47	6	0	-2.300496	-1.053733	-3.111307
48	6	0	0.136327	-1.190836	-3.434981
49	6	0	0.293504	-2.515217	-3.107576
50	6	0	-0.994021	-3.207499	-2.930996
51	6	0	-2.208204	-2.494176	-2.915782
52	6	0	1.177238	-0.240586	-3.433975
53	6	0	2.485150	-0.499851	-3.105216
54	6	0	2.745571	-1.938147	-2.926224
55	6	0	1.691811	-2.872226	-2.910104

206	6	0	-8.850002	3.931491	4.006574
207	6	0	-10.069003	4.606669	3.979509
208	6	0	-10.503052	5.167519	2.780185
209	6	0	-9.683925	5.044754	1.659593
210	7	0	-8.506524	4.404347	1.681811
211	1	0	-8.483906	3.506339	4.933942
212	1	0	-10.666395	4.694814	4.881166
213	1	0	-11.449310	5.692411	2.709938
214	1	0	-9.981386	5.464795	0.703640
215	6	0	-4.427353	1.671028	2.576304
216	6	0	-3.262993	-0.504015	2.318164
217	6	0	-3.196093	0.897249	2.307092
218	6	0	-1.933322	1.583627	2.027732
219	6	0	-0.933753	0.743689	1.586540
220	6	0	-2.129328	-1.358026	2.034562
221	6	0	-1.022650	-0.667793	1.589427
222	6	0	0.292335	-1.188442	1.590555
223	6	0	-1.866043	-2.770884	2.315233
224	6	0	-0.553965	-3.267513	2.324154
225	6	0	0.607867	-2.453518	2.037167
226	6	0	1.193776	-0.098583	1.588326
227	6	0	2.495362	-0.188688	2.032432
228	6	0	2.911084	-1.544517	2.320867
229	6	0	2.033336	-2.638861	2.315768
230	6	0	0.435992	1.095471	1.585827
231	6	0	0.924800	2.306197	2.027247
232	6	0	2.343293	2.282899	2.313402
233	6	0	3.112611	1.109865	2.308497
234	6	0	-1.472208	2.925938	2.310800
235	6	0	-0.118798	3.295162	2.303358
236	1	0	2.847141	3.206707	2.578814
237	1	0	3.945231	-1.737878	2.587208
238	1	0	-0.418779	-4.310314	2.592733
239	1	0	-4.213666	-0.954114	2.585316
240	1	0	-2.194805	3.691047	2.575689
241	47	0	2.344934	8.017613	0.012597
242	47	0	2.829136	-7.856689	-0.007049
243	47	0	-6.606303	-5.118769	-0.001662
244	47	0	-6.909240	4.702119	-0.003281
245	47	0	8.341211	0.256824	-0.002625

[biconvex-(*M*,*P*,*M*)-$\mathrm{Hg_5}\mathbf{L}_2$]$^{10+}$:

Center Number	Atomic Number	Atomic Type	Coordinates (Angstroms) X	Y	Z
1	80	0	2.651380	-8.100709	0.000388
2	80	0	-6.896429	-5.020173	0.002004
3	80	0	-6.911887	4.998784	-0.001891
4	6	0	0.381561	5.468743	3.839715
5	6	0	0.499620	6.851720	3.833344
6	6	0	1.261748	7.484541	2.838589
7	7	0	1.838952	6.741550	1.867143
8	6	0	1.031074	4.693445	2.861406
9	6	0	1.736237	5.401058	1.881780
10	1	0	-0.187300	4.983142	4.626708
11	1	0	-0.001233	7.433962	4.596600
12	1	0	2.238034	4.872882	1.075922
13	6	0	2.901295	-4.650819	3.843571
14	6	0	1.922991	-4.405822	2.862160
15	6	0	1.773644	-5.392013	1.880990
16	7	0	2.481671	-6.534997	1.867278
17	6	0	3.621552	-5.837317	3.838239
18	6	0	3.383536	-6.795902	2.840737
19	1	0	3.072078	-3.924259	4.632068
20	6	0	4.112091	-8.097344	2.821438
21	6	0	-3.524314	-4.201218	3.836188
22	6	0	-4.431568	-5.251676	3.831961
23	6	0	-5.421673	-5.316898	2.838932
24	7	0	-5.455126	-4.376277	1.867860
25	6	0	-3.596633	-3.192472	2.857639
26	6	0	-4.585430	-3.351094	1.880557
27	1	0	-2.777616	-4.141660	4.622081
28	1	0	-4.367175	-6.017004	4.595155
29	1	0	-4.684640	-2.629101	1.074891
30	6	0	-5.082908	2.058288	3.840369
31	6	0	-6.361243	2.599133	3.836985
32	6	0	-6.730216	3.516580	2.840504
33	7	0	-5.848427	3.832361	1.865048
34	6	0	-4.147889	2.432632	2.857522
35	6	0	-4.605534	3.319558	1.876952
36	1	0	-4.794524	1.369472	4.628444
37	1	0	-7.067488	2.306275	4.603661
38	6	0	-2.692588	-2.012610	2.927204
39	6	0	-3.256033	-0.722084	2.909159
40	6	0	-2.485281	0.498098	3.105433
41	6	0	-1.177287	0.239448	3.434360
42	6	0	-1.244252	-2.208450	3.107477
43	6	0	-0.594378	-1.043943	3.435270
44	6	0	0.806193	-0.886168	3.436820
45	6	0	-0.321843	-3.318882	2.912654
46	6	0	1.079561	-3.181624	2.931332
47	6	0	1.713016	-1.864651	3.111062
48	6	0	1.088936	0.494524	3.436967
49	6	0	2.300027	1.054330	3.111767
50	6	0	3.356808	0.044486	2.934152
51	6	0	3.054223	-1.330754	2.916830
52	6	0	-0.136843	1.190211	3.435526
53	6	0	-0.294663	2.514523	3.108127
54	6	0	0.992509	3.207460	2.931582
55	6	0	2.207037	2.494733	2.916335

56	6	0	2.693693	2.010805	-2.927425
57	6	0	3.256522	0.720003	-2.909239
58	1	0	0.718167	4.320069	-2.768214
59	1	0	-3.116696	-3.072633	-2.773347
60	1	0	1.961152	-3.914699	-2.765541
61	1	0	4.331273	0.654245	-2.764778
62	6	0	-0.384182	-5.469222	-3.838853
63	6	0	-1.033311	-4.693455	-2.860669
64	6	0	-1.738905	-5.400572	-1.880984
65	7	0	-1.842364	-6.740997	-1.866207
66	6	0	-0.503006	-6.852139	-3.832331
67	6	0	-1.265534	-7.484424	-2.837549
68	1	0	0.184962	-4.984020	-4.625888
69	6	0	-1.448639	-8.964676	-2.820370
70	6	0	8.079379	-4.153203	-2.825781
71	1	0	-5.324188	0.622475	-1.074765
72	1	0	-7.073028	-2.282623	-4.606403
73	6	0	-4.108274	8.098762	-2.822442
74	6	0	6.440658	6.405305	-2.823831
75	1	0	-3.884681	2.016637	-2.773825
76	1	0	-2.240452	-4.872024	-1.075214
77	1	0	-0.002465	-7.434753	-4.595512
78	6	0	3.535268	-4.191561	3.836510
79	6	0	4.445209	-5.239693	3.832399
80	6	0	5.435490	-5.302501	2.839392
81	7	0	5.466523	-4.361915	1.868194
82	6	0	3.605017	-3.182745	2.857854
83	6	0	4.594215	-3.338954	1.880784
84	1	0	2.788402	-4.133836	4.622380
85	1	0	4.382755	-6.005107	4.595666
86	1	0	4.691518	-2.616833	1.075001
87	6	0	-0.395823	5.468216	3.838970
88	6	0	-1.043244	4.691101	2.860730
89	6	0	-1.750216	5.396737	1.880968
90	7	0	-1.856476	6.736941	1.866161
91	6	0	-0.517562	6.850881	3.832446
92	6	0	-1.281317	7.481556	2.837589
93	1	0	0.174287	4.984208	4.626040
94	6	0	-1.467529	8.961426	2.820446
95	6	0	-5.323228	1.306251	3.845871
96	6	0	-6.676287	1.615651	3.841136
97	6	0	-7.511728	1.086443	2.844889
98	7	0	-6.980574	0.313740	1.870238
99	6	0	-4.783350	0.454401	2.864732
100	6	0	-5.672579	0.002331	1.883507
101	1	0	-4.687049	1.697267	4.633973
102	1	0	-7.077784	2.267293	4.607074
103	1	0	-5.322863	-0.633503	1.074913
104	6	0	-2.889076	-4.657853	3.844193
105	6	0	-3.606217	-5.846239	3.839083
106	6	0	-3.365679	-6.804385	2.841768
107	7	0	-2.464509	-6.541296	1.868253
108	6	0	-1.911401	-4.410489	2.862751
109	6	0	-1.759471	-5.396461	1.881756
110	1	0	-3.061778	-3.931580	4.632537
111	1	0	-4.350813	-6.024230	4.604803
112	1	0	-1.045942	-5.263919	1.073335
113	6	0	-3.356842	0.036089	2.934182
114	6	0	-3.050676	-1.338362	2.917005
115	6	0	-1.708064	-1.868736	3.111222
116	6	0	-0.803783	-0.887836	3.436776
117	6	0	-2.302699	1.048721	3.111582
118	6	0	-1.090123	0.492113	3.436737
119	6	0	0.133838	1.190988	3.435138
120	6	0	-2.213479	2.489340	2.915980
121	6	0	-1.000811	3.205229	2.931085
122	6	0	0.288179	2.515677	3.107608
123	6	0	1.176755	0.242942	3.434072
124	6	0	2.484061	0.504971	3.105091
125	6	0	2.741430	1.943803	2.926029
126	6	0	1.685699	2.875650	2.909963
127	6	0	0.597193	-1.041962	3.435201
128	6	0	1.250103	-2.204812	3.107573
129	6	0	2.697918	-2.005212	2.927231
130	6	0	3.258005	-0.713227	2.908991
131	6	0	-1.071174	-3.184076	2.931674
132	6	0	0.330585	-3.317683	2.912979
133	1	0	-3.880412	-2.024970	2.774112
134	1	0	1.952816	3.918685	2.765336
135	1	0	4.332590	-0.645178	2.764367
136	1	0	0.727300	-4.318642	2.768117
137	6	0	5.077571	2.072136	3.839858
138	6	0	4.141602	2.443797	2.856911
139	6	0	4.596926	3.331711	1.876145
140	7	0	5.838461	3.847770	1.864129
141	6	0	6.354478	2.616355	3.836372
142	6	0	6.721049	3.534537	2.839692
143	1	0	4.790976	1.382760	4.628096
144	6	0	8.070650	4.169874	2.825181
145	6	0	6.454529	-6.391470	2.823563
146	1	0	-2.250569	4.867128	1.075152
147	1	0	-0.018310	7.434542	4.595669
148	6	0	-8.977021	1.364080	2.829523
149	6	0	-4.090800	-8.107750	2.822693
150	1	0	-3.123224	3.065837	2.773568
151	1	0	3.941361	3.641548	1.067135
152	1	0	7.061464	2.325563	4.603152
153	80	0	-2.630239	-8.107554	0.001561
154	80	0	6.909900	-5.001925	0.002715
155	6	0	-9.650145	1.810727	3.970098
156	6	0	-11.021743	2.073862	3.910659
157	6	0	-11.696299	1.892076	2.704957
158	6	0	-10.974357	1.427248	1.607719

56	6	0	-2.746424	1.936275	2.926567
57	6	0	-1.693124	2.870873	2.910590
58	1	0	-4.330794	-0.656812	2.764547
59	1	0	3.885732	-2.015183	2.773787
60	1	0	3.115269	3.073614	2.773935
61	1	0	-1.962952	3.913228	2.766085
62	6	0	5.319823	1.319902	3.845750
63	6	0	4.782199	0.466559	2.864667
64	6	0	5.672637	0.016752	1.883494
65	7	0	6.979809	0.331594	1.870224
66	6	0	6.672055	1.632887	3.840996
67	6	0	7.508899	1.105806	2.844796
68	1	0	4.682616	1.709276	4.633834
69	6	0	8.973439	1.387365	2.829411
70	6	0	1.443952	8.964908	2.821556
71	1	0	1.059748	-5.261208	1.072608
72	1	0	4.366590	-6.013486	4.603947
73	6	0	-6.437932	-6.408456	2.822959
74	6	0	-8.081515	4.148292	2.826051
75	1	0	-0.715954	-4.320845	2.767622
76	1	0	5.324624	-0.620107	1.074969
77	1	0	7.071819	2.285652	4.606885
78	6	0	2.886574	4.659839	-3.843682
79	6	0	3.603114	5.848582	-3.838402
80	6	0	3.362156	6.806423	-2.840887
81	7	0	2.461153	6.542721	-1.867393
82	6	0	1.909084	4.411799	-2.862216
83	6	0	1.756690	5.397530	-1.881061
84	1	0	3.059584	3.933800	-4.632173
85	1	0	4.347563	6.027084	-4.604143
86	1	0	1.043230	5.264532	-1.072654
87	6	0	-5.076450	-2.074384	-3.840575
88	6	0	-4.140317	-2.445666	-2.857636
89	6	0	-4.595233	-3.333853	-1.876934
90	7	0	-5.836532	-3.850509	-1.864981
91	6	0	-6.353098	-2.619202	-3.837147
92	6	0	-6.719250	-3.537631	-2.840531
93	1	0	-4.790186	-1.384777	-4.628731
94	6	0	-8.068590	-4.173517	-2.826034
95	6	0	0.398605	-5.467557	-3.839783
96	6	0	0.520978	-6.850160	-3.833421
97	6	0	1.285060	-7.480605	-2.838659
98	7	0	1.859923	-6.735823	-1.867195
99	6	0	1.045669	-4.690242	-2.861454
100	6	0	1.753017	-5.395660	-1.881817
101	1	0	-0.171744	-4.983730	-4.626792
102	1	0	0.021968	-7.433954	-4.596701
103	1	0	2.253146	-4.865930	-1.075940
104	6	0	5.323884	-1.303158	-3.845761
105	6	0	6.677084	-1.611926	-3.840992
106	6	0	7.512238	-1.082366	-2.844686
107	7	0	6.980699	-0.309941	-1.870029
108	6	0	4.783564	-0.451622	-2.864586
109	6	0	5.672551	0.000829	-1.883320
110	1	0	4.687925	-1.694410	-4.633924
111	1	0	7.078918	-2.263343	-4.606945
112	1	0	5.322519	0.636481	-1.074717
113	6	0	1.002477	-3.204384	-2.931627
114	6	0	2.214767	-2.487859	-2.916324
115	6	0	2.303253	-1.047164	-3.111731
116	6	0	1.090421	-0.491149	-3.436945
117	6	0	-0.286852	-2.515478	-3.108197
118	6	0	-0.133170	-1.190669	-3.435548
119	6	0	-1.176594	-0.243174	-3.434427
120	6	0	-1.684195	-2.876207	-2.910700
121	6	0	-2.740411	-1.944910	-2.926669
122	6	0	-2.483780	-0.505925	-3.105534
123	6	0	-0.597708	1.042037	-3.435339
124	6	0	-1.251235	2.204492	-3.107542
125	6	0	-2.698954	2.004117	-2.927283
126	6	0	-3.258361	0.711837	-2.909276
127	6	0	0.803354	0.888646	-3.436827
128	6	0	1.707096	1.869967	-3.111040
129	6	0	1.069506	3.184951	-2.931356
130	6	0	-0.332321	3.317815	-2.912719
131	6	0	3.356856	-0.034010	-2.934085
132	6	0	3.049966	1.340278	-2.916770
133	1	0	3.124792	-3.063904	-2.773887
134	1	0	-4.332920	0.643193	-2.764724
135	1	0	-0.729589	4.318536	-2.767729
136	1	0	3.879320	2.027313	-2.773712
137	6	0	-3.537570	4.190071	-3.836333
138	6	0	-3.606677	3.181165	-2.857718
139	6	0	-4.595837	3.336795	-1.880530
140	7	0	-5.468673	4.359307	-1.867776
141	6	0	-4.448058	5.237729	-3.832062
142	6	0	-5.438231	5.299969	-2.838906
143	1	0	-2.790760	4.132770	-4.622286
144	6	0	-6.457797	6.388440	-2.822859
145	6	0	4.086683	8.110113	-2.821604
146	1	0	-3.939555	-3.643390	-1.067902
147	1	0	-7.060231	-2.328652	-4.603886
148	6	0	1.471892	-8.960393	-2.821640
149	6	0	8.977650	-1.359357	-2.829307
150	1	0	-1.950765	-3.919399	-2.766193
151	1	0	-4.692706	2.614558	-1.074798
152	1	0	-4.386107	6.003212	-4.595302
153	80	0	8.521906	0.013240	0.000076
154	80	0	2.626125	8.108879	-0.000472
155	6	0	1.249016	-9.740373	-3.959802
156	6	0	1.421269	-11.126275	-3.898807
157	6	0	1.807631	-11.710168	-2.694013
158	6	0	2.032530	-10.878368	-1.599143

159	7	0	-9.654909	1.165663	1.672102
160	7	0	-9.652459	-1.185801	-1.671630
161	6	0	-10.971368	-1.450079	-1.607202
162	6	0	-11.692341	-1.916638	-2.704342
163	6	0	-11.017396	-2.097313	-3.909993
164	6	0	-9.646344	-1.831364	-3.969479
165	1	0	-9.128575	1.928091	4.912039
166	1	0	-11.551894	2.410937	4.796292
167	1	0	-12.758459	2.093607	2.611061
168	1	0	-11.459732	1.268480	0.650283
169	1	0	-11.457084	-1.292066	-0.649814
170	1	0	-12.754088	-2.120317	-2.610416
171	1	0	-11.546842	-2.435678	-4.795556
172	1	0	-9.124527	-1.947852	-4.911391
173	6	0	-4.728392	-8.608503	3.961189
174	6	0	-5.411734	-9.826299	3.897286
175	6	0	-5.452204	-10.519758	2.689256
176	6	0	-4.781567	-9.978752	1.594309
177	7	0	-4.115839	-8.810207	1.663078
178	7	0	-1.849762	-9.547775	-1.663685
179	6	0	-2.004582	-10.883895	-1.597679
180	6	0	-1.777611	-11.715249	-2.692457
181	6	0	-1.392624	-11.130535	-3.897295
182	6	0	-1.223778	-9.744222	-3.958439
183	6	0	6.730594	-7.153741	3.962087
184	6	0	7.683436	-8.174813	3.901948
185	6	0	8.339740	-8.421119	2.697625
186	6	0	8.034515	-7.615819	1.602393
187	7	0	7.122846	-6.626818	1.667446
188	7	0	8.514122	-4.710289	-1.668392
189	6	0	9.736854	-5.271081	-1.604581
190	6	0	10.593270	-5.317548	-2.702498
191	6	0	10.151718	-4.775924	-3.908072
192	6	0	8.885765	-4.185988	-3.966939
193	6	0	8.877167	4.204139	3.966204
194	6	0	10.141870	4.796738	3.907224
195	6	0	10.582082	5.339500	2.701670
196	6	0	9.725573	5.291435	1.603894
197	7	0	8.504033	4.728061	1.667816
198	7	0	7.108246	6.642305	-1.667627
199	6	0	8.017648	7.633393	-1.602540
200	6	0	8.321212	8.439255	-2.697821
201	6	0	7.665674	8.191311	-3.902225
202	6	0	6.715168	7.168066	-3.962400
203	6	0	-1.244587	9.741388	3.958612
204	6	0	-1.416329	11.127344	3.897502
205	6	0	-1.802241	11.711321	2.692598
206	6	0	-2.027198	10.879560	1.597720
207	7	0	-1.869589	9.543755	1.663701
208	6	0	-4.747285	8.597987	-3.960816
209	6	0	-5.433328	9.814260	-3.896843
210	6	0	-5.475033	10.507737	-2.688865
211	6	0	-4.802889	9.968334	-1.594053
212	7	0	-4.134570	8.801272	-1.662891
213	1	0	-4.675804	-8.079516	4.904856
214	1	0	-5.899646	-10.225524	4.781362
215	1	0	-5.979576	-11.463176	2.592014
216	1	0	-4.783254	-10.486579	0.635332
217	1	0	-2.309077	-11.295945	-0.641125
218	1	0	-1.911094	-12.787989	-2.597446
219	1	0	-1.229683	-11.739819	-4.781175
220	1	0	-0.948462	-9.285362	-4.900073
221	1	0	6.236891	-6.944824	4.903232
222	1	0	7.909803	-8.763000	4.786153
223	1	0	9.079174	-9.209776	2.603409
224	1	0	8.523537	-7.770060	0.646038
225	1	0	10.037992	-5.683849	-0.647250
226	1	0	11.572387	-5.776100	-2.609304
227	1	0	10.778056	-4.813774	-4.794246
228	1	0	8.531340	-3.786145	-4.909047
229	1	0	8.523758	3.803384	4.908306
230	1	0	10.768274	4.835746	4.793300
231	1	0	11.560221	5.800115	2.608389
232	1	0	10.025673	5.705009	0.646582
233	1	0	8.506150	7.788874	-0.646120
234	1	0	9.058829	9.229606	-2.603575
235	1	0	7.890845	8.779912	-4.786461
236	1	0	6.222086	6.957901	-4.903593
237	1	0	-0.968536	9.283053	4.900286
238	1	0	-1.254886	11.736921	4.781455
239	1	0	-1.937939	12.783785	2.597613
240	1	0	-2.332301	11.291020	0.641107
241	1	0	-4.693765	8.069041	-4.904452
242	1	0	-5.922354	10.212318	-4.780829
243	1	0	-6.004481	11.449985	-2.591571
244	1	0	-4.805441	10.476258	-0.635128
245	1	0	4.686078	2.626744	-1.075305

[biconcave-(*M*,*P*,*M*)-Hg_5**L**$_2$]$^{10+}$:

Center Number	Atomic Number	Atomic Type	Coordinates (Angstroms) X	Y	Z
1	80	0	-8.440915	0.913538	-0.000407
2	6	0	3.834494	3.889658	3.736288
3	6	0	5.009959	4.616734	3.876817
4	6	0	6.027411	4.497369	2.916299
5	7	0	5.874864	3.643584	1.878484
6	6	0	4.756867	2.906598	1.773363
7	1	0	3.042474	4.001909	4.470149
8	1	0	5.119079	5.302916	4.707645

159	7	0	1.874425	-9.542628	-1.665008
160	7	0	4.138942	-8.799584	1.661727
161	6	0	4.807805	-9.966322	1.592770
162	6	0	5.479972	-10.505671	2.687599
163	6	0	5.437681	-9.812508	3.895733
164	6	0	4.751061	-8.596565	3.959835
165	1	0	0.972620	-9.282094	-4.901401
166	1	0	1.259870	-11.735854	-4.782766
167	1	0	1.943736	-12.782586	-2.599084
168	1	0	2.338033	-11.289778	-0.642635
169	1	0	4.810764	-10.474053	0.633744
170	1	0	6.009877	-11.447648	2.590170
171	1	0	5.926683	-10.210549	4.779740
172	1	0	4.697065	-8.067881	4.903591
173	6	0	9.651014	-1.805549	-3.969918
174	6	0	11.022715	-2.068150	-3.910460
175	6	0	11.697140	-1.886286	-2.704698
176	6	0	10.974965	-1.421882	-1.607432
177	7	0	9.655417	-1.160819	-1.671832
178	7	0	9.651842	1.190818	1.671967
179	6	0	10.970573	1.455974	1.607571
180	6	0	11.691275	1.922738	2.704805
181	6	0	11.016261	2.102643	3.910531
182	6	0	9.645383	1.835781	3.969987
183	6	0	4.724034	8.611342	-3.960024
184	6	0	5.406884	9.829406	-3.895925
185	6	0	5.447111	10.522658	-2.687771
186	6	0	4.776689	9.981195	-1.592916
187	7	0	4.111429	8.812397	-1.661873
188	7	0	1.844709	9.548373	1.664925
189	6	0	1.998656	10.884596	1.599036
190	6	0	1.771124	11.715717	2.693878
191	6	0	1.386531	11.130650	3.898668
192	6	0	1.218594	9.744218	3.959689
193	6	0	-6.734473	7.150554	-3.961335
194	6	0	-7.687736	8.171224	-3.900997
195	6	0	-8.343849	8.417282	-2.696524
196	6	0	-8.038032	7.612127	-1.601346
197	7	0	-7.125960	6.623514	-1.666589
198	7	0	-8.516395	4.705453	1.668745
199	6	0	-9.739466	5.265473	1.604888
200	6	0	-10.596137	5.311036	2.702653
201	6	0	-10.154466	4.769335	3.908145
202	6	0	-8.888137	4.180201	3.967065
203	6	0	-8.875052	-4.208124	-3.967082
204	6	0	-10.139576	-4.801116	-3.908103
205	6	0	-10.579651	-5.343915	-2.702522
206	6	0	-9.723177	-5.295522	-1.604726
207	7	0	-8.501813	-4.731786	-1.668643
208	6	0	-6.712220	-7.171399	3.961459
209	6	0	-7.662428	-8.194920	3.901209
210	6	0	-8.317916	-8.442931	2.696796
211	6	0	-8.014613	-7.636857	1.601594
212	7	0	-7.105496	-6.645523	1.666748
213	1	0	9.129539	-1.922948	-4.911907
214	1	0	11.553037	-2.404866	-4.796128
215	1	0	12.759373	-2.087410	-2.610761
216	1	0	11.460254	-1.263009	-0.649968
217	1	0	11.456379	1.298509	0.650138
218	1	0	12.752881	2.127150	2.610871
219	1	0	11.545513	2.441111	4.796171
220	1	0	9.123521	1.951662	4.911950
221	1	0	4.671619	8.082522	-4.903793
222	1	0	5.894595	10.228989	-4.779950
223	1	0	5.974102	11.466270	-2.590351
224	1	0	4.778150	10.488895	-0.633870
225	1	0	2.302908	11.296935	0.642530
226	1	0	1.903888	12.788551	2.598931
227	1	0	1.223188	11.739742	4.782606
228	1	0	0.943581	9.285112	4.901290
229	1	0	-6.240918	6.941805	-4.902596
230	1	0	-7.914565	8.759282	-4.785170
231	1	0	-9.083597	9.205625	-2.602131
232	1	0	-8.526922	7.766174	-0.644891
233	1	0	-10.040689	5.678372	0.647637
234	1	0	-11.575538	5.768966	2.609387
235	1	0	-10.780990	4.806497	4.794216
236	1	0	-8.533642	3.780297	4.909121
237	1	0	-8.521734	-3.807317	-4.909195
238	1	0	-10.765937	-4.840379	-4.794199
239	1	0	-11.557637	-5.804808	-2.609210
240	1	0	-10.023194	-5.709125	-0.647401
241	1	0	-6.219214	-6.961154	4.902674
242	1	0	-7.887416	-8.783657	4.785401
243	1	0	-9.055311	-9.233481	2.602464
244	1	0	-8.503150	-7.792346	0.645192
245	1	0	-3.950793	3.631307	1.068009

[biconcave-(*P*,*M*,*P*)-Hg_5**L**$_2$]$^{10+}$:

Center Number	Atomic Number	Atomic Type	Coordinates (Angstroms) X	Y	Z
1	80	0	-8.172238	-2.309656	0.000543
2	6	0	2.104438	5.038853	-3.740767
3	6	0	2.922921	6.152679	-3.880876
4	6	0	3.908267	6.424424	-2.918046
5	7	0	4.083642	5.577294	-1.878307
6	6	0	3.322241	4.475618	-1.773607
7	1	0	1.329943	4.845678	-4.476529
8	1	0	2.769633	6.828389	-4.713349

9	1	0	4.714638	2.218963	0.935841
10	6	0	7.267443	5.319615	2.992279
11	6	0	7.708315	5.873298	4.197001
12	6	0	8.855976	6.671111	4.216399
13	6	0	9.537277	6.908051	3.024009
14	6	0	9.057395	6.313997	1.858769
15	7	0	7.958398	5.535706	1.845698
16	1	0	7.185008	5.670049	5.123620
17	1	0	9.209367	7.098011	5.150161
18	1	0	10.424177	7.532462	2.989999
19	1	0	9.555635	6.475603	0.908853
20	6	0	3.672657	3.008896	2.652790
21	6	0	4.880315	-2.445231	3.738983
22	6	0	4.232024	-3.625960	1.775429
23	7	0	5.278259	-4.461731	1.881343
24	6	0	5.934413	-3.339083	3.880700
25	6	0	6.136207	-4.343440	2.920234
26	1	0	4.742069	-1.657120	4.472630
27	6	0	7.301309	-5.268763	2.997131
28	1	0	3.565489	-3.797910	0.937430
29	1	0	6.619729	-3.231110	4.712420
30	6	0	7.962037	-5.518516	4.202646
31	6	0	9.075697	-6.363142	4.222743
32	6	0	9.513921	-6.935867	3.030222
33	6	0	8.802526	-6.661718	1.864146
34	7	0	7.722379	-5.857498	1.850486
35	1	0	7.605149	-5.085029	5.129201
36	1	0	9.589374	-6.568490	5.157090
37	1	0	10.382261	-7.585867	2.996720
38	1	0	9.111998	-7.083840	0.914012
39	6	0	3.993849	-2.562957	2.654472
40	6	0	-0.821616	-5.395522	3.739761
41	6	0	-2.140699	-5.145297	1.773155
42	7	0	-2.612950	-6.398305	1.879425
43	6	0	-1.346363	-6.674067	3.881454
44	6	0	-2.237482	-7.176892	2.919705
45	1	0	-0.116331	-5.019995	4.474635
46	6	0	-2.757837	-8.570724	2.996317
47	1	0	-2.508203	-4.565248	0.933811
48	1	0	-1.033452	-7.291934	4.714164
49	6	0	-2.793065	-9.275638	4.202166
50	6	0	-3.252756	-10.595610	4.222528
51	6	0	-3.660779	-11.189794	3.029814
52	6	0	-3.617717	-10.429208	1.863344
53	7	0	-3.186024	-9.153613	1.849281
54	1	0	-2.492009	-8.801922	5.128842
55	1	0	-3.290782	-11.147044	5.157147
56	1	0	-4.011089	-12.216337	2.996362
57	1	0	-3.922440	-10.854673	0.913172
58	6	0	-1.205372	-4.589656	2.653931
59	6	0	-2.507392	4.850718	3.742042
60	6	0	-2.834122	6.193672	3.883111
61	6	0	-2.409193	7.123748	2.920616
62	7	0	-1.648283	6.713900	1.880275
63	6	0	-1.293789	5.422573	1.774831
64	1	0	-2.857192	4.132732	4.477300
65	1	0	-3.450069	6.510202	4.715835
66	1	0	-0.655892	5.168785	0.935343
67	6	0	-2.807163	8.557396	2.996982
68	6	0	-3.192474	9.148869	4.202770
69	6	0	-3.596376	10.486947	4.222628
70	6	0	-3.616028	11.206997	3.029617
71	6	0	-3.204024	10.566000	1.863253
72	7	0	-2.803437	9.280290	1.849731
73	1	0	-3.157335	8.589033	5.129678
74	1	0	-3.889426	10.955819	5.157145
75	1	0	-3.936157	12.243346	2.995941
76	1	0	-3.207787	11.088849	0.912821
77	6	0	-1.723780	4.423678	2.656060
78	6	0	-5.387024	-0.887383	3.740451
79	6	0	-6.765396	-0.785255	3.881495
80	6	0	-7.519515	-0.094239	2.919385
81	7	0	-6.895193	0.504672	1.879731
82	6	0	-5.557448	0.444584	1.774133
83	1	0	-4.811596	-1.441449	4.475574
84	1	0	-7.256047	-1.274260	4.714079
85	1	0	-5.120135	0.974086	0.934854
86	6	0	-9.006154	-0.032348	2.994781
87	6	0	-9.688652	-0.218604	4.199676
88	6	0	-11.086129	-0.190975	4.218255
89	6	0	-11.776201	0.013513	3.024900
90	6	0	-11.038420	0.209683	1.859498
91	7	0	-9.691855	0.195028	1.847227
92	1	0	-9.146086	-0.358574	5.126936
93	1	0	-11.623296	-0.326539	5.152124
94	1	0	-12.860736	0.028162	2.990286
95	1	0	-11.536061	0.368421	0.908775
96	6	0	-4.739330	-0.272508	2.655003
97	6	0	-2.780641	-1.763818	2.370463
98	6	0	-3.290777	-0.455775	2.363783
99	6	0	-2.416223	0.685386	2.078973
100	6	0	-1.161554	0.324846	1.628574
101	6	0	-1.397416	-2.084624	2.077849
102	6	0	-0.666742	-1.002785	1.627955
103	6	0	0.748817	-0.942512	1.628017
104	6	0	-0.582559	-3.269135	2.362705
105	6	0	0.819121	-3.188212	2.370415
106	6	0	1.551937	-1.971843	2.077703
107	6	0	1.128845	0.422340	1.628217
108	6	0	2.356105	0.868051	2.077822
109	6	0	3.286535	-0.204688	2.369893
110	6	0	2.930281	-1.562818	2.362785
111	6	0	-0.051793	1.205459	1.628766

9	1	0	3.537733	3.823402	-0.934425
10	6	0	4.750190	7.651205	-2.993154
11	6	0	4.956111	8.327473	-4.198344
12	6	0	5.721344	9.497133	-4.217032
13	6	0	6.259448	9.974375	-3.023361
14	6	0	6.032635	9.245953	-1.857643
15	7	0	5.305372	8.112509	-1.845256
16	1	0	4.550553	7.941109	-5.125725
17	1	0	5.892683	10.023583	-5.151125
18	1	0	6.847439	10.885801	-2.988688
19	1	0	6.430034	9.584367	-0.906716
20	6	0	2.280801	4.163398	-2.655327
21	6	0	5.437903	-0.445069	-3.742432
22	6	0	5.282117	-1.775948	-1.774094
23	7	0	6.565276	-2.158861	-1.880364
24	6	0	6.750217	-0.878569	-3.884147
25	6	0	7.314992	-1.730827	-2.921573
26	1	0	5.013343	0.231191	-4.477904
27	6	0	8.741868	-2.152372	-2.998720
28	1	0	4.730004	-2.182186	-0.933754
29	1	0	7.344180	-0.524118	-4.717583
30	6	0	9.446952	-2.139141	-4.204949
31	6	0	10.795754	-2.505634	-4.225650
32	6	0	11.417619	-2.870232	-3.032976
33	6	0	10.656450	-2.879719	-1.866159
34	7	0	9.353816	-2.538181	-1.851815
35	1	0	8.952951	-1.872593	-5.131603
36	1	0	11.348040	-2.505777	-5.160538
37	1	0	12.466156	-3.147924	-2.999912
38	1	0	11.102476	-3.153270	-0.915936
39	6	0	4.661554	-0.883104	-2.655621
40	6	0	1.254732	-5.310459	-3.741445
41	6	0	-0.059534	-5.573300	-1.773261
42	7	0	-0.026943	-6.911985	-1.879221
43	6	0	1.248073	-6.692525	-3.882930
44	6	0	0.612075	-7.492879	-2.920166
45	1	0	1.766693	-4.697703	-4.476904
46	6	0	0.652693	-8.980192	-2.996938
47	1	0	-0.616809	-5.173557	-0.933160
48	1	0	1.768793	-7.148101	-4.716199
49	6	0	0.883243	-9.646928	-4.203011
50	6	0	0.952329	-11.042939	-4.223234
51	6	0	0.798410	-11.746745	-3.030307
52	6	0	0.553946	-11.025546	-1.863671
53	7	0	0.475502	-9.681160	-1.849772
54	1	0	0.983482	-9.095007	-5.129894
55	1	0	1.122959	-11.568444	-5.157989
56	1	0	0.858945	-12.829730	-2.996923
57	1	0	0.431918	-11.534073	-0.913299
58	6	0	0.598049	-4.707358	-2.654768
59	6	0	-4.143502	3.559889	-3.741354
60	6	0	-4.949395	4.682801	-3.881956
61	6	0	-4.901477	5.705157	-2.920517
62	7	0	-4.040371	5.611044	-1.881752
63	6	0	-3.228078	4.546370	-1.776901
64	1	0	-4.200564	2.762472	-4.475766
65	1	0	-5.640545	4.745186	-4.713544
66	1	0	-2.539679	4.550836	-0.938990
67	6	0	-5.807340	6.885447	-2.996099
68	6	0	-6.387053	7.289754	-4.201377
69	6	0	-7.262219	8.379595	-4.220506
70	6	0	-7.548798	9.039960	-3.027216
71	6	0	-6.926040	8.599521	-1.861393
72	7	0	-6.073575	7.557000	-1.848575
73	1	0	-6.145558	6.783742	-5.128430
74	1	0	-7.710111	8.704874	-5.154648
75	1	0	-8.233227	9.881396	-2.992918
76	1	0	-7.124429	9.082900	-0.910769
77	6	0	-3.254792	3.458225	-2.657080
78	6	0	-4.665460	-2.838291	-3.739945
79	6	0	-5.982161	-3.258565	-3.880727
80	6	0	-6.939942	-2.897940	-2.919178
81	7	0	-6.584843	-2.108332	-1.880065
82	6	0	-5.321565	-1.664002	-1.775019
83	1	0	-3.924391	-3.138232	-4.474329
84	1	0	-6.254665	-3.896572	-4.712451
85	1	0	-5.113486	-1.008263	-0.936699
86	6	0	-8.342153	-3.395480	-2.995261
87	6	0	-8.905097	-3.822325	-4.200735
88	6	0	-10.211760	-4.318559	-4.220364
89	6	0	-10.928952	-4.387202	-3.027443
90	6	0	-10.318432	-3.930501	-1.861418
91	7	0	-9.063716	-3.441355	-1.848110
92	1	0	-8.348950	-3.748924	-5.127609
93	1	0	-10.658901	-4.644446	-5.154656
94	1	0	-11.940517	-4.778639	-2.993603
95	1	0	-10.839939	-3.969756	-0.911059
96	6	0	-4.294736	-2.024598	-2.655378
97	6	0	-1.920404	-2.675492	-2.371375
98	6	0	-2.882701	-1.653085	-2.364604
99	6	0	-2.498134	-0.267551	-2.080340
100	6	0	-1.199740	-0.132677	-1.630016
101	6	0	-0.517589	-2.455773	-2.078917
102	6	0	-0.244485	-1.178975	-1.629415
103	6	0	1.045775	-0.593824	-1.629410
104	6	0	0.681138	-3.249642	-2.363700
105	6	0	1.950886	-2.650347	-2.371289
106	6	0	2.175552	-1.248244	-2.079049
107	6	0	0.887949	0.814164	-1.629817
108	6	0	1.859452	1.686234	-2.079705
109	6	0	3.123449	1.039174	-2.371920
110	6	0	3.300927	-0.353610	-2.364242
111	6	0	-0.499911	1.099164	-1.630269

112	6	0	-0.096399	2.510101	2.078989
113	6	0	1.211295	3.063629	2.370548
114	6	0	2.393042	2.305456	2.362625
115	6	0	-2.538180	2.099935	2.372380
116	6	0	-1.451666	2.989199	2.364896
117	1	0	1.292842	4.109680	2.651720
118	1	0	4.306534	0.040977	2.651184
119	1	0	1.367597	-4.082506	2.651939
120	1	0	-3.461496	-2.562016	2.651870
121	1	0	-3.507359	2.501023	2.654688
122	6	0	-3.488450	-4.198830	-3.742525
123	6	0	-4.096228	-5.440184	-3.883255
124	6	0	-3.880841	-6.439722	-2.920766
125	7	0	-3.049042	-6.203059	-1.880834
126	6	0	-2.425196	-5.018049	-1.775737
127	1	0	-3.675939	-3.422639	-4.477963
128	1	0	-4.765948	-5.617084	-4.715859
129	1	0	-1.747082	-4.907377	-0.936700
130	6	0	-4.578092	-7.754151	-2.996468
131	6	0	-5.081917	-8.249473	-4.201894
132	6	0	-5.764693	-9.469099	-4.220900
133	6	0	-5.939019	-10.167174	-3.027394
134	6	0	-5.398364	-9.629341	-1.861469
135	7	0	-4.730088	-8.460177	-1.848799
136	1	0	-4.926978	-7.710920	-5.129117
137	1	0	-6.151918	-9.864454	-5.155129
138	1	0	-6.474933	-11.110169	-2.993025
139	1	0	-5.514517	-10.138431	-0.910655
140	6	0	-2.631118	-3.949940	-2.656768
141	6	0	2.915213	-4.615831	-3.740058
142	6	0	4.022688	-3.858823	-1.772698
143	7	0	4.956589	-4.818573	-1.878063
144	6	0	3.907377	-5.578110	-3.880730
145	6	0	4.924428	-5.682560	-2.918130
146	1	0	2.119161	-4.553685	-4.475514
147	6	0	5.958679	-6.752189	-2.993725
148	1	0	4.126964	-3.180171	-0.933274
149	1	0	3.868424	-6.269778	-4.713269
150	6	0	6.273428	-7.384817	-4.199105
151	6	0	7.222231	-8.411177	-4.218314
152	6	0	7.832840	-8.792322	-3.024998
153	6	0	7.488858	-8.111670	-1.859073
154	7	0	6.583466	-7.114735	-1.846137
155	1	0	5.808741	-7.071263	-5.126222
156	1	0	7.478062	-8.901930	-5.152525
157	1	0	8.564017	-9.593472	-2.990732
158	1	0	7.937631	-8.379447	-0.908491
159	6	0	2.943831	-3.723693	-2.654236
160	6	0	5.291858	1.343690	-3.740660
161	6	0	4.914504	2.633303	-1.774755
162	7	0	6.116464	3.223570	-1.880322
163	6	0	6.514324	1.988621	-3.881564
164	6	0	6.928508	2.924099	-2.919645
165	1	0	4.986340	0.605112	-4.475253
166	6	0	8.266298	3.575462	-2.995107
167	1	0	4.301555	2.943573	-0.935642
168	1	0	7.160205	1.736433	-4.713624
169	6	0	8.966400	3.677032	-4.200010
170	6	0	10.236513	4.260582	-4.218658
171	6	0	10.787410	4.723972	-3.025400
172	6	0	10.032647	4.609437	-1.859997
173	7	0	8.803990	4.058159	-1.847658
174	1	0	8.524944	3.331658	-5.127166
175	1	0	10.783217	4.350568	-5.152497
176	1	0	11.775868	5.170512	-2.990853
177	1	0	10.425454	4.953807	-0.909312
178	6	0	4.452066	1.648416	-2.655412
179	6	0	-5.071321	2.024650	-3.740012
180	6	0	-6.439620	2.220090	-3.880239
181	6	0	-7.323830	1.704563	-2.918788
182	7	0	-6.841909	0.984482	-1.880252
183	6	0	-5.522210	0.756835	-1.775513
184	1	0	-4.390847	2.444105	-4.474517
185	1	0	-6.814565	2.804212	-4.711695
186	1	0	-5.207622	0.144995	-0.937255
187	6	0	-8.789269	1.962080	-2.994084
188	6	0	-9.415712	2.291128	-4.198890
189	6	0	-10.786653	2.563536	-4.217775
190	6	0	-11.504894	2.510506	-3.024699
191	6	0	-10.826628	2.159772	-1.859405
192	7	0	-9.508117	1.885745	-1.846814
193	1	0	-8.855440	2.312366	-5.125933
194	1	0	-11.281966	2.811878	-5.151599
195	1	0	-12.567394	2.728516	-2.990149
196	1	0	-11.347213	2.110491	-0.908989
197	6	0	-4.569981	1.283533	-2.655943
198	6	0	0.355222	5.448860	-3.740750
199	6	0	0.117128	6.810356	-3.881552
200	6	0	-0.645479	7.492046	-2.919407
201	7	0	-1.179476	6.811494	-1.879692
202	6	0	-0.987476	5.486198	-1.774552
203	1	0	0.963637	4.931113	-4.475754
204	1	0	0.555218	7.347379	-4.713890
205	1	0	-1.471377	4.997836	-0.935849
206	6	0	-0.855133	8.965061	-2.995713
207	6	0	-0.739034	9.661423	-4.201461
208	6	0	-0.905159	11.049237	-4.221259
209	6	0	-1.175742	11.716771	-3.028225
210	6	0	-1.296560	10.964330	-1.861942
211	7	0	-1.148345	9.625849	-1.848500
212	1	0	-0.547104	9.134327	-5.128347
213	1	0	-0.824553	11.596288	-5.155751
214	1	0	-1.297777	12.794550	-2.994507

112	6	0	-1.029076	2.292362	-2.080878
113	6	0	-0.023034	3.294501	-2.373294
114	6	0	1.356427	3.033019	-2.365102
115	6	0	-3.140306	0.998711	-2.373684
116	6	0	-2.465405	2.229918	-2.366353
117	1	0	-0.338175	4.295109	-2.655013
118	1	0	3.977643	1.648177	-2.653668
119	1	0	2.794185	-3.274540	-2.652674
120	1	0	-2.253357	-3.670539	-2.652364
121	1	0	-4.189338	1.007934	-2.655341
122	6	0	-1.664000	-5.202308	3.740010
123	6	0	-1.762519	-6.580897	3.881253
124	6	0	-1.186764	-7.427190	2.919859
125	7	0	-0.502874	-6.896457	1.880672
126	6	0	-0.368440	-5.564138	1.775102
127	1	0	-2.129837	-4.552438	4.474276
128	1	0	-2.318343	-6.995622	4.713215
129	1	0	0.219862	-5.207930	0.936603
130	6	0	-1.340540	-8.907059	2.995908
131	6	0	-1.622842	-9.554743	4.201328
132	6	0	-1.797539	-10.941518	4.220802
133	6	0	-1.695549	-11.654565	3.027786
134	6	0	-1.395636	-10.953534	1.861774
135	7	0	-1.215569	-9.618968	1.848615
136	1	0	-1.682351	-8.997017	5.128258
137	1	0	-2.008923	-11.452907	5.155060
138	1	0	-1.837855	-12.729848	2.993840
139	1	0	-1.311227	-11.469482	0.911315
140	6	0	-0.960508	-4.650775	2.655226
141	6	0	4.432218	-3.194480	3.738718
142	6	0	5.175983	-2.071280	1.775249
143	7	0	6.401709	-2.610722	1.879540
144	6	0	5.712999	-3.714223	3.878696
145	6	0	6.695558	-3.426529	2.917595
146	1	0	3.670618	-3.438006	4.472992
147	6	0	8.055790	-4.029455	2.993039
148	1	0	5.018460	-1.400578	0.937788
149	1	0	5.935808	-4.372223	4.709646
150	6	0	8.584382	-4.499697	4.197908
151	6	0	9.849647	-5.093617	4.217020
152	6	0	10.559910	-5.214573	3.024102
153	6	0	9.986061	-4.711110	1.858693
154	7	0	8.772062	-4.128219	1.845907
155	1	0	8.035141	-4.385787	5.124807
156	1	0	10.270499	-5.453904	5.150891
157	1	0	11.538926	-5.681460	2.989779
158	1	0	10.503354	-4.788321	0.908353
159	6	0	4.124603	-2.353466	2.655309
160	6	0	4.403123	3.225699	3.740956
161	6	0	3.569214	4.279896	1.775497
162	7	0	4.461209	5.278636	1.881526
163	6	0	5.293446	4.282759	3.882504
164	6	0	5.326005	5.305791	2.921170
165	1	0	4.397431	2.426457	4.475586
166	6	0	6.320636	6.412279	2.997768
167	1	0	2.884536	4.337456	0.936484
168	1	0	5.986347	4.291237	4.714891
169	6	0	6.930029	6.769334	4.203340
170	6	0	7.887062	7.788011	4.223395
171	6	0	8.223995	8.425256	3.030689
172	6	0	7.568880	8.035526	1.864536
173	7	0	6.638156	7.062255	1.850850
174	1	0	6.650239	6.282756	5.130027
175	1	0	8.358862	8.076658	5.157805
176	1	0	8.971634	9.211094	2.997078
177	1	0	7.804078	8.503002	0.914342
178	6	0	3.510521	3.192952	2.655610
179	6	0	-5.457141	-0.027558	3.740766
180	6	0	-6.798789	-0.359142	3.883001
181	6	0	-7.427451	-1.166016	2.920859
182	7	0	-6.712997	-1.650716	1.879742
183	6	0	-5.404296	-1.367497	1.773040
184	1	0	-4.981614	0.614463	4.475718
185	1	0	-7.363707	0.040200	4.716321
186	1	0	-4.885174	-1.815512	0.932885
187	6	0	-8.882480	-1.476666	2.998305
188	6	0	-9.584336	-1.408960	4.204583
189	6	0	-10.957273	-1.670917	4.225487
190	6	0	-11.605411	-1.987247	3.033030
191	6	0	-10.847369	-2.055349	1.866120
192	7	0	-9.522405	-1.814643	1.851565
193	1	0	-9.071279	-1.180796	5.131133
194	1	0	-11.507842	-1.628384	5.160422
195	1	0	-12.672136	-2.183849	3.000193
196	1	0	-11.313165	-2.294342	0.916064
197	6	0	-4.716907	-0.524540	2.654086
198	6	0	-1.710330	5.180347	3.740286
199	6	0	-2.440338	6.353856	3.882161
200	6	0	-3.401610	6.702465	2.919721
201	7	0	-3.641396	5.873226	1.878431
202	6	0	-2.967538	4.716108	1.772095
203	1	0	-0.953113	4.926738	4.475674
204	1	0	-2.235298	7.014431	4.715589
205	1	0	-3.232685	4.083841	0.931859
206	6	0	-4.146855	7.990182	2.997181
207	6	0	-4.299388	8.678543	4.203492
208	6	0	-4.972671	9.903395	4.224501
209	6	0	-5.473723	10.422166	3.032052
210	6	0	-5.304356	9.680192	1.865128
211	7	0	-4.666071	8.494388	1.850475
212	1	0	-3.923873	8.261077	5.130035
213	1	0	-5.102274	10.440122	5.159468
214	1	0	-5.990192	11.376007	2.999205

Center Number	Atomic Number	Atomic Type	X	Y	Z
215	1	0	-1.502748	11.444674	-0.911443
216	6	0	-0.192602	4.743435	-2.655375
217	6	0	1.484272	2.940315	-2.372569
218	6	0	0.132493	3.319952	-2.364655
219	6	0	-0.917328	2.337621	-2.079780
220	6	0	-0.435416	1.124204	-1.630216
221	6	0	1.939382	1.595288	-2.079736
222	6	0	0.934320	0.762047	-1.629950
223	6	0	1.013098	-0.652530	-1.629652
224	6	0	3.198225	0.900265	-2.364193
225	6	0	3.255118	-0.502612	-2.370989
226	6	0	2.116296	-1.350966	-2.079044
227	6	0	-0.307983	-1.164524	-1.630168
228	6	0	-0.631113	-2.429504	-2.079916
229	6	0	0.527676	-3.250231	-2.371858
230	6	0	1.844378	-2.762749	-2.363880
231	6	0	-1.203159	-0.066496	-1.630525
232	6	0	-2.505964	-0.149885	-2.080821
233	6	0	-2.928192	-1.505603	-2.373648
234	6	0	-2.057582	-2.607122	-2.365825
235	6	0	-2.337205	2.320644	-2.372170
236	6	0	-3.115774	1.152264	-2.365256
237	1	0	-3.960840	-1.689856	-2.655687
238	1	0	0.383411	-4.289329	-2.653688
239	1	0	4.199025	-0.961040	-2.651684
240	1	0	2.211947	3.696022	-2.654247
241	1	0	-2.831381	3.246262	-2.653008
242	80	0	-3.479586	-7.743799	-0.000377
243	80	0	6.288934	-5.703853	0.002475
244	80	0	7.369680	4.217299	-0.000602
245	80	0	-1.737807	8.311155	0.000253

Ad⊂[biconvex-(*P*,*M*,*P*)-Ag_5**L**$_2$]$^{5+}$:

Center Number	Atomic Number	Atomic Type	Coordinates (Angstroms) X	Y	Z
1	47	0	5.241819	-5.174127	-0.011526
2	47	0	-3.313927	-6.606361	0.037225
3	47	0	-7.313754	1.111085	0.029010
4	6	0	-2.548304	4.919290	-4.336125
5	6	0	-3.142427	6.102118	-3.918076
6	6	0	-2.638187	6.775678	-2.796210
7	7	0	-1.610501	6.255300	-2.091374
8	6	0	-1.451419	4.401479	-3.630877
9	6	0	-1.044951	5.112840	-2.494167
10	1	0	-2.915289	4.408766	-5.221136
11	1	0	-3.993610	6.496850	-4.459151
12	1	0	-0.232359	4.736093	-1.879412
13	6	0	4.852381	-2.570994	-4.401681
14	6	0	3.675672	-2.787139	-3.668864
15	6	0	3.790483	-3.575726	-2.516702
16	7	0	4.931410	-4.148884	-2.119843
17	6	0	6.039457	-3.162933	-3.992923
18	6	0	6.053824	-3.975561	-2.850340
19	1	0	4.828635	-1.960348	-5.298989
20	6	0	7.292304	-4.669993	-2.403784
21	6	0	-0.964718	-5.483859	-4.369549
22	6	0	-1.162865	-6.791257	-3.947531
23	6	0	-1.931163	-7.043306	-2.801854
24	7	0	-2.441073	-6.021355	-2.081654
25	6	0	-1.532752	-4.423461	-3.647478
26	6	0	-2.247354	-4.763711	-2.491745
27	1	0	-0.391043	-5.282575	-5.269014
28	1	0	-0.718814	-7.608858	-4.502057
29	1	0	-2.673209	-3.984008	-1.866481
30	6	0	-5.540209	-0.858866	-4.351972
31	6	0	-6.842972	-1.068057	-3.920715
32	6	0	-7.314726	-0.400751	-2.781066
33	7	0	-6.495198	0.406510	-2.073708
34	6	0	-4.701785	0.014542	-3.642956
35	6	0	-5.240308	0.601796	-2.490528
36	1	0	-5.177498	-1.351935	-5.248628
37	1	0	-7.486572	-1.748478	-4.464674
38	6	0	-1.387529	-3.019914	-4.089123
39	6	0	-2.503018	-2.180764	-4.096058
40	6	0	-2.432109	-0.785637	-4.461499
41	6	0	-1.216356	-0.389221	-4.990525
42	6	0	-0.070340	-2.505761	-4.445118
43	6	0	-0.071182	-1.230808	-4.981951
44	6	0	1.083588	-0.404096	-4.977824
45	6	0	1.235470	-3.008209	-4.087122
46	6	0	2.382450	-2.212659	-4.096859
47	6	0	2.300896	-0.800770	-4.453657
48	6	0	0.653986	0.949050	-4.972437
49	6	0	1.406644	1.980804	-4.439863
50	6	0	2.774844	1.620314	-4.085062
51	6	0	3.181572	0.284593	-4.092262
52	6	0	-0.766566	0.959064	-4.977878
53	6	0	-1.514815	1.988906	-4.435071
54	6	0	-0.750436	3.177606	-4.073259
55	6	0	0.645479	3.152965	-4.078040
56	6	0	-3.323842	0.306806	-4.090545
57	6	0	-2.864587	1.625346	-4.076248
58	1	0	-3.446758	-2.594947	-3.755811
59	1	0	4.189345	0.062612	-3.756185
60	1	0	1.166676	4.041919	-3.737711
61	1	0	-3.547146	2.393070	-3.726244
62	6	0	3.877928	3.864185	-4.361800
63	6	0	3.724352	2.666103	-3.648053
64	6	0	4.525289	2.508175	-2.509081

Center Number	Atomic Number	Atomic Type	X	Y	Z
215	1	0	-5.675566	10.049463	0.915109
216	6	0	-1.953725	4.322791	2.653504
217	6	0	0.276148	3.279006	2.370295
218	6	0	-1.119373	3.124673	2.362248
219	6	0	-1.724829	1.820734	2.077090
220	6	0	-0.823557	0.876084	1.627540
221	6	0	1.201826	2.202174	2.078198
222	6	0	0.582174	1.053112	1.628132
223	6	0	1.184884	-0.229061	1.628775
224	6	0	2.628952	2.029222	2.364140
225	6	0	3.206920	0.749640	2.372145
226	6	0	2.468955	-0.463414	2.079459
227	6	0	0.151664	-1.198492	1.628572
228	6	0	0.325338	-2.492111	2.079278
229	6	0	1.706916	-2.819243	2.372418
230	6	0	2.745346	-1.874295	2.364862
231	6	0	-1.089624	-0.515527	1.627758
232	6	0	-2.266508	-1.080498	2.077879
233	6	0	-2.150834	-2.495618	2.370966
234	6	0	-0.931120	-3.191047	2.364144
235	6	0	-3.035057	1.273363	2.369594
236	6	0	-3.319511	-0.101560	2.362605
237	1	0	-3.039839	-3.052736	2.652373
238	1	0	1.961944	-3.836831	2.654099
239	1	0	4.253430	0.678002	2.654196
240	1	0	0.667567	4.252255	2.652244
241	1	0	-3.839689	1.946677	2.650907
242	80	0	-0.325666	-8.485365	-0.000067
243	80	0	7.969310	-2.929706	-0.001423
244	80	0	5.250409	6.671885	0.002567
245	80	0	-4.720994	7.057690	-0.000443

Ad⊂[biconvex-(*M*,*P*,*M*)-Ag_5**L**$_2$]$^{5+}$:

Center Number	Atomic Number	Atomic Type	Coordinates (Angstroms) X	Y	Z
1	47	0	-5.259720	-5.151373	-0.012936
2	47	0	3.282198	-6.612297	0.036224
3	47	0	7.311132	1.068752	0.029449
4	6	0	2.559943	4.914878	-4.339815
5	6	0	3.158617	6.095753	-3.922958
6	6	0	2.660733	6.769192	-2.798020
7	7	0	1.635614	6.250055	-2.088614
8	6	0	1.464736	4.399268	-3.630268
9	6	0	1.065547	5.109473	-2.490493
10	1	0	2.921853	4.403662	-5.226522
11	1	0	4.008443	6.489264	-4.466983
12	1	0	0.255234	4.733050	-1.872497
13	6	0	-4.866159	-2.547509	-4.400111
14	6	0	-3.689271	-2.768235	-3.668938
15	6	0	-3.805358	-3.556304	-2.516530
16	7	0	-4.947725	-4.125483	-2.117910
17	6	0	-6.054723	-3.135169	-3.989612
18	6	0	-6.070427	-3.948321	-2.847341
19	1	0	-4.841412	-1.936524	-5.297149
20	6	0	-7.311155	-4.638508	-2.400496
21	6	0	0.938758	-5.481872	-4.372408
22	6	0	1.129695	-6.789737	-3.948585
23	6	0	1.895821	-7.044416	-2.801965
24	7	0	2.410964	-6.024358	-2.082934
25	6	0	1.511569	-4.423593	-3.650904
26	6	0	2.223968	-4.766165	-2.494616
27	1	0	0.366566	-5.278565	-5.272359
28	1	0	0.681394	-7.605649	-4.502170
29	1	0	2.653356	-3.987870	-1.870012
30	6	0	5.533415	-0.871324	-4.363108
31	6	0	6.835576	-1.086645	-3.933093
32	6	0	7.309034	-0.427456	-2.789342
33	7	0	6.491924	0.378012	-2.077577
34	6	0	4.697640	0.000537	-3.648921
35	6	0	5.237683	0.579672	-2.493341
36	1	0	5.168987	-1.358428	-5.262355
37	1	0	7.477253	-1.765768	-4.480917
38	6	0	1.372483	-3.019619	-4.093698
39	6	0	2.491406	-2.185288	-4.102179
40	6	0	2.425021	-0.789776	-4.467215
41	6	0	1.210422	-0.388672	-4.995282
42	6	0	0.057073	-2.500317	-4.448850
43	6	0	0.062060	-1.225991	-4.986981
44	6	0	-1.089880	-0.395010	-4.982375
45	6	0	-1.250213	-2.998085	-4.089279
46	6	0	-2.394420	-2.198675	-4.098230
47	6	0	-2.307616	-0.787107	-4.455832
48	6	0	-0.654991	0.956845	-4.975810
49	6	0	-1.403414	1.990681	-4.440914
50	6	0	-2.772494	1.635291	-4.085405
51	6	0	-3.183882	0.300925	-4.092477
52	6	0	0.765533	0.961299	-4.981128
53	6	0	1.517743	1.987569	-4.436970
54	6	0	0.758070	3.178584	-4.072932
55	6	0	-0.637597	3.159269	-4.076799
56	6	0	3.320482	0.298895	-4.094934
57	6	0	2.866124	1.618984	-4.078488
58	1	0	3.434118	-2.602790	-3.763133
59	1	0	-4.191987	0.082134	-3.755284
60	1	0	-1.155171	4.049188	-3.733435
61	1	0	3.551007	2.384162	-3.727206
62	6	0	-3.865640	3.884331	-4.360913
63	6	0	-3.718084	2.684655	-3.648609
64	6	0	-4.520057	2.528951	-2.510053

65	7	0	5.435232	3.403214	-2.110680
66	6	0	4.818579	4.797977	-3.949867
67	6	0	5.613680	4.535449	-2.825059
68	1	0	3.278808	4.048856	-5.248099
69	6	0	6.673398	5.482400	-2.382374
70	6	0	-3.207700	8.074430	-2.344418
71	1	0	2.924031	-3.741288	-1.882826
72	1	0	6.948631	-2.989793	-4.555549
73	6	0	-2.210688	-8.430327	-2.340096
74	6	0	-8.719136	-0.553339	-2.312360
75	1	0	1.336128	-4.033872	-3.746792
76	1	0	4.419419	1.623218	-1.887595
77	1	0	4.931371	5.725262	-4.498022
78	7	0	7.346343	-5.064869	-1.116199
79	6	0	8.440406	-5.695756	-0.671422
80	6	0	9.541128	-5.968520	-1.482418
81	6	0	9.487448	-5.577121	-2.818600
82	6	0	8.349979	-4.921251	-3.286284
83	1	0	8.428966	-5.991376	0.373612
84	1	0	10.407253	-6.479508	-1.076426
85	1	0	10.312824	-5.785894	-3.491573
86	1	0	8.281155	-4.640780	-4.330524
87	7	0	7.094640	5.382226	-1.106041
88	6	0	8.049003	6.211414	-0.665457
89	6	0	8.637284	7.186378	-1.469903
90	6	0	8.218553	7.286848	-2.795053
91	6	0	7.226134	6.424721	-3.258325
92	7	0	-2.967252	8.441020	-1.069811
93	6	0	-3.455797	9.603778	-0.620725
94	6	0	-4.211277	10.466053	-1.414194
95	6	0	-4.452391	10.101657	-2.737265
96	6	0	-3.944270	8.892709	-3.209588
97	7	0	-8.977327	-0.232973	-1.028737
98	6	0	-10.227557	-0.350479	-0.564354
99	6	0	-11.290156	-0.793882	-1.350595
100	6	0	-11.034957	-1.113203	-2.682635
101	6	0	-9.735098	-0.990837	-3.170791
102	6	0	-2.124248	-9.523735	-3.210426
103	6	0	-2.398275	-10.802518	-2.728344
104	6	0	-2.754499	-10.958942	-1.390380
105	6	0	-2.834264	-9.818594	-0.592217
106	7	0	-2.570134	-8.588629	-1.050692
107	1	0	8.349668	6.085128	0.370544
108	1	0	9.405124	7.838123	-1.067703
109	1	0	8.662235	8.017854	-3.463266
110	1	0	6.914957	6.469926	-4.295099
111	1	0	-3.230477	9.848416	0.413224
112	1	0	-4.589204	11.396733	-1.005387
113	1	0	-5.018920	10.750783	-3.397118
114	1	0	-4.095955	8.612928	-4.245215
115	1	0	-10.377612	-0.077799	0.476196
116	1	0	-12.286045	-0.876364	-0.929278
117	1	0	-11.835000	-1.443977	-3.337035
118	1	0	-9.529058	-1.204301	-4.212812
119	1	0	-1.878300	-9.383485	-4.256226
120	1	0	-2.342689	-11.659648	-3.391641
121	1	0	-2.974173	-11.935659	-0.973396
122	1	0	-3.120189	-9.886694	0.453358
123	1	0	-4.625552	1.250774	-1.873101
124	6	0	0.832068	5.514611	4.350398
125	6	0	1.029724	6.820890	3.924037
126	6	0	0.358219	7.291208	2.786260
127	7	0	-0.443182	6.467717	2.077100
128	6	0	-0.033339	4.670747	3.637890
129	6	0	-0.626585	5.209419	2.488499
130	1	0	1.327374	5.153668	5.246480
131	1	0	1.704227	7.468399	4.470713
132	1	0	-1.271696	4.592585	1.869027
133	6	0	-3.808583	-3.938528	4.372551
134	6	0	-2.620034	-3.778891	3.643847
135	6	0	-2.468535	-4.584741	2.507847
136	7	0	-3.361737	-5.503163	2.124533
137	6	0	-4.739647	-4.888664	3.976938
138	6	0	-4.484334	-5.686542	2.852505
139	1	0	-3.987507	-3.336234	5.257849
140	6	0	-5.429198	-6.753682	2.423878
141	6	0	2.635081	-4.856474	4.379177
142	6	0	3.241968	-6.032482	3.960575
143	6	0	4.059680	-6.025960	2.821511
144	7	0	4.224042	-4.894222	2.103337
145	6	0	2.842881	-3.669611	3.660418
146	6	0	3.637883	-3.763559	2.510887
147	1	0	2.019824	-4.848984	5.273622
148	1	0	3.076377	-6.949674	4.512416
149	1	0	3.798950	-2.887985	1.887980
150	6	0	5.512072	0.993864	4.409557
151	6	0	6.812292	1.199371	3.968286
152	6	0	7.043179	1.955947	2.810296
153	7	0	6.007392	2.448330	2.097391
154	6	0	4.438001	1.543256	3.694027
155	6	0	4.757261	2.247423	2.525786
156	1	0	5.327492	0.428538	5.317918
157	1	0	7.640743	0.769397	4.517728
158	1	0	3.965417	2.656357	1.904584
159	6	0	2.254229	-2.385745	4.098966
160	6	0	3.048006	-1.237353	4.126546
161	6	0	2.531710	0.062942	4.486484
162	6	0	1.243721	0.049643	4.990594
163	6	0	0.836342	-2.317774	4.435668
164	6	0	0.425947	-1.111724	4.974964
165	6	0	-0.932074	-0.695283	4.968824
166	6	0	-0.238793	-3.207699	4.065612
167	6	0	-1.579467	-2.818101	4.067444

65	7	0	-5.425433	3.427798	-2.110143
66	6	0	-4.801805	4.822062	-3.947547
67	6	0	-5.598129	4.561848	-2.823023
68	1	0	-3.265327	4.067397	-5.246719
69	6	0	-6.651822	5.514558	-2.378457
70	6	0	3.235536	8.066429	-2.348336
71	1	0	-2.938640	-3.724420	-1.883722
72	1	0	-6.964349	-2.958272	-4.550332
73	6	0	2.166274	-8.432531	-2.337881
74	6	0	8.712731	-0.587232	-2.320741
75	1	0	-1.353138	-4.023253	-3.748154
76	1	0	-4.416477	1.643138	-1.889521
77	1	0	-4.910220	5.750941	-4.493892
78	7	0	-7.366520	-5.032087	-1.112571
79	6	0	-8.462783	-5.658716	-0.667241
80	6	0	-9.564537	-5.928055	-1.477999
81	6	0	-9.509412	-5.538227	-2.814575
82	6	0	-8.369540	-4.886960	-3.282950
83	1	0	-8.452304	-5.953783	0.377963
84	1	0	-10.432563	-6.435403	-1.071495
85	1	0	-10.335532	-5.744798	-3.487318
86	1	0	-8.299441	-4.607987	-4.327495
87	7	0	-7.071033	5.416651	-1.101246
88	6	0	-8.018190	6.252748	-0.658199
89	6	0	-8.601185	7.232223	-1.461042
90	6	0	-8.185313	7.329669	-2.787291
91	6	0	-7.200158	6.460577	-3.253127
92	7	0	3.001918	8.433201	-1.072509
93	6	0	3.495660	9.594562	-0.625249
94	6	0	4.249799	10.455066	-1.421869
95	6	0	4.483674	10.090618	-2.746210
96	6	0	3.970100	8.883242	-3.216642
97	7	0	8.971357	-0.274168	-1.035450
98	6	0	10.220876	-0.398114	-0.570992
99	6	0	11.282467	-0.841113	-1.358911
100	6	0	11.026816	-1.152918	-2.692639
101	6	0	9.727662	-1.023820	-3.180882
102	6	0	2.071871	-9.526819	-3.206289
103	6	0	2.335978	-10.806764	-2.721802
104	6	0	2.690624	-10.963458	-1.383442
105	6	0	2.779109	-9.822271	-0.587376
106	7	0	2.524535	-8.591151	-1.048175
107	1	0	-8.317314	6.128629	0.378490
108	1	0	-9.363136	7.889565	-1.056756
109	1	0	-8.625722	8.063703	-3.454355
110	1	0	-6.891426	6.503281	-4.290742
111	1	0	3.275779	9.839662	0.409754
112	1	0	4.632189	11.384539	-1.014457
113	1	0	5.048867	10.738491	-3.408441
114	1	0	4.116175	8.603533	-4.253086
115	1	0	10.371142	-0.131126	0.470987
116	1	0	12.277916	-0.929052	-0.937656
117	1	0	11.826068	-1.483175	-3.348277
118	1	0	9.521598	-1.231548	-4.224048
119	1	0	1.827052	-9.386620	-4.252353
120	1	0	2.273926	-11.664669	-3.383525
121	1	0	2.902665	-11.941066	-0.964602
122	1	0	3.064371	-9.890761	0.458341
123	1	0	4.624779	1.227243	-1.872535
124	6	0	-0.805324	5.514229	4.352704
125	6	0	-0.995114	6.822573	3.929082
126	6	0	-0.320187	7.291355	2.792728
127	7	0	0.476683	6.464796	2.082186
128	6	0	0.055751	4.666947	3.638927
129	6	0	0.652689	5.204567	2.490968
130	1	0	-1.303022	5.154349	5.247905
131	1	0	-1.665975	7.472823	4.477000
132	1	0	1.294903	4.585345	1.870801
133	6	0	3.794154	-3.956862	4.376985
134	6	0	2.604858	-3.792320	3.650839
135	6	0	2.449629	-4.593696	2.512131
136	7	0	3.340525	-5.512109	2.123617
137	6	0	4.722855	-4.907088	3.975923
138	6	0	4.464073	-5.700218	2.848774
139	1	0	3.975761	-3.357311	5.263591
140	6	0	5.407112	-6.766339	2.413837
141	6	0	-2.656544	-4.848253	4.380706
142	6	0	-3.268000	-6.021518	3.961228
143	6	0	-4.081857	-6.012047	2.819315
144	7	0	-4.237288	-4.880360	2.099198
145	6	0	-2.855888	-3.661102	3.660057
146	6	0	-3.646656	-3.752235	2.507492
147	1	0	-2.044116	-4.842838	5.277113
148	1	0	-3.109143	-6.938822	4.514913
149	1	0	-3.800234	-2.876690	1.882673
150	6	0	-5.505895	1.011317	4.404762
151	6	0	-6.805645	1.223061	3.965150
152	6	0	-7.034232	1.984197	2.809662
153	7	0	-5.997310	2.474937	2.097652
154	6	0	-4.430120	1.558850	3.690118
155	6	0	-4.747525	2.267957	2.524425
156	1	0	-5.322831	0.442399	5.311192
157	1	0	-7.635502	0.794548	4.513672
158	1	0	-3.954733	2.676650	1.904213
159	6	0	-2.263139	-2.379550	4.099945
160	6	0	-3.051796	-1.227696	4.124197
161	6	0	-2.530786	0.070559	4.484944
162	6	0	-1.244323	0.051925	4.992610
163	6	0	-0.845997	-2.317715	4.440869
164	6	0	-0.431520	-1.113116	4.980582
165	6	0	0.928348	-0.702341	4.976165
166	6	0	0.225633	-3.212639	4.073324
167	6	0	1.567854	-2.828471	4.076520

168	6	0	-1.954129	-1.454974	4.427194
169	6	0	-0.952708	0.725200	4.964049
170	6	0	-1.985708	1.465707	4.418010
171	6	0	-3.168236	0.692082	4.055130
172	6	0	-3.130383	-0.703830	4.057666
173	6	0	0.391037	1.185068	4.979997
174	6	0	0.783520	2.405719	4.459909
175	6	0	-0.314416	3.288352	4.079707
176	6	0	-1.629470	2.817837	4.058969
177	6	0	3.037771	1.385562	4.140731
178	6	0	2.186545	2.492584	4.129351
179	1	0	4.080755	-1.330877	3.806020
180	1	0	-4.013971	-1.233244	3.715894
181	1	0	-2.400707	3.494469	3.705322
182	1	0	2.597112	3.439204	3.792891
183	6	0	-4.912961	2.482121	4.333653
184	6	0	-4.400021	1.386052	3.623335
185	6	0	-5.124486	0.976945	2.496133
186	7	0	-6.270689	1.541860	2.103500
187	6	0	-6.101045	3.074319	3.928083
188	6	0	-6.784623	2.569899	2.812429
189	1	0	-4.393991	2.850141	5.213266
190	6	0	-8.086919	3.139837	2.371581
191	6	0	0.499420	8.697909	2.321032
192	1	0	-1.592417	-4.474221	1.875421
193	1	0	-5.659402	-5.006406	4.536667
194	6	0	4.769343	-7.252128	2.365505
195	6	0	8.421359	2.242044	2.326435
196	1	0	-0.005777	-4.211805	3.725824
197	1	0	-4.755808	0.162025	1.879728
198	1	0	-6.491752	3.924581	4.473506
199	6	0	5.031096	-8.314521	3.239133
200	6	0	5.701508	-9.439798	2.762532
201	6	0	6.096378	-9.476558	1.426797
202	6	0	5.812383	-8.371782	0.625126
203	7	0	5.167517	-7.289506	1.078450
204	1	0	4.747426	-8.258303	4.283297
205	1	0	5.918720	-10.268757	3.428391
206	1	0	6.618613	-10.332789	1.014183
207	1	0	6.110308	-8.347117	-0.419023
208	6	0	9.528509	2.167555	3.180361
209	6	0	10.797928	2.447274	2.677265
210	6	0	10.931549	2.797194	1.335184
211	6	0	9.778503	2.865232	0.554308
212	7	0	8.557525	2.595499	1.032853
213	6	0	0.930728	9.715073	3.181194
214	6	0	1.042429	11.016977	2.695948
215	6	0	0.718889	11.272931	1.365071
216	6	0	0.282128	10.208866	0.577065
217	7	0	0.174885	8.956649	1.038531
218	6	0	-8.898933	3.874129	3.244545
219	6	0	-10.111495	4.383065	2.782457
220	6	0	-10.485513	4.145065	1.461502
221	6	0	-9.629138	3.391469	0.659945
222	7	0	-8.463088	2.901765	1.099280
223	6	0	-6.353471	-7.314571	3.313655
224	6	0	-7.214685	-8.313379	2.862506
225	6	0	-7.131291	-8.729868	1.535450
226	6	0	-6.173598	-8.133012	0.716661
227	7	0	-5.345279	-7.172599	1.145613
228	1	0	9.406226	1.926318	4.229483
229	1	0	11.665416	2.400896	3.327683
230	1	0	11.900287	3.021200	0.902184
231	1	0	9.828534	3.146532	-0.493549
232	1	0	1.147431	9.508243	4.222393
233	1	0	1.368170	11.817962	3.351726
234	1	0	0.793069	12.270441	0.946037
235	1	0	0.006312	10.359443	-0.462593
236	1	0	-8.611455	4.023008	4.278476
237	1	0	-10.755958	4.947716	3.448456
238	1	0	-11.419257	4.523712	1.060422
239	1	0	-9.881456	3.168555	-0.372665
240	1	0	-6.385006	-7.004530	4.351313
241	1	0	-7.931818	-8.763611	3.541288
242	1	0	-7.783125	-9.502371	1.142431
243	1	0	-6.061167	-8.431239	-0.321640
244	47	0	6.547776	3.330126	-0.025603
245	47	0	-1.166552	7.288846	-0.016879
246	6	0	1.493052	0.556180	-0.069112
247	6	0	1.065643	0.306957	1.385973
248	6	0	0.555852	1.603182	-0.700587
249	6	0	-0.895410	1.085796	-0.666816
250	6	0	-1.317653	0.834881	0.792317
251	6	0	-0.382836	-0.211423	1.427194
252	6	0	1.398097	-0.764228	-0.857140
253	6	0	-0.473416	-1.532281	0.640854
254	6	0	-0.977107	-0.234796	-1.453099
255	6	0	-0.050656	-1.288786	-0.819662
256	1	0	-0.116887	-2.222862	-1.392445
257	1	0	2.528052	0.927130	-0.095278
258	1	0	1.144570	1.232057	1.966553
259	1	0	1.734702	-0.423685	1.854610
260	1	0	0.856011	1.805734	-1.734373
261	1	0	0.629677	2.551351	-0.147448
262	1	0	-1.559849	1.827370	-1.130794
263	1	0	-1.276488	1.767250	1.370882
264	1	0	-2.357805	0.481228	0.829843
265	1	0	-0.676774	-0.381277	2.463278
266	1	0	1.710926	-0.606685	-1.893924
267	1	0	2.081160	-1.508923	-0.422683
268	1	0	0.175370	-2.286507	1.107678
269	1	0	-1.500188	-1.924158	0.680390
270	1	0	-0.683517	-0.063759	-2.491227

168	6	0	1.947713	-1.466939	4.436274
169	6	0	0.955366	0.717983	4.968663
170	6	0	1.992101	1.453172	4.422300
171	6	0	3.171512	0.673982	4.061410
172	6	0	3.127350	-0.721655	4.066021
173	6	0	-0.386672	1.183688	4.981948
174	6	0	-0.772553	2.405033	4.458780
175	6	0	0.329653	3.282767	4.079776
176	6	0	1.642515	2.806413	4.061096
177	6	0	-3.030592	1.395081	4.136848
178	6	0	-2.174505	2.498354	4.125810
179	1	0	-4.083751	-1.316159	3.799777
180	1	0	4.008249	-1.256036	3.725025
181	1	0	2.417341	3.479060	3.707609
182	1	0	-2.579777	3.446744	3.787950
183	6	0	4.925614	2.456413	4.335247
184	6	0	4.406503	1.361343	3.628010
185	6	0	5.128403	0.944567	2.501940
186	7	0	6.278379	1.500538	2.107892
187	6	0	6.117844	3.039412	3.928230
188	6	0	6.798873	2.526875	2.814724
189	1	0	4.408046	2.830374	5.213155
190	6	0	8.106016	3.086194	2.374726
191	6	0	-0.451926	8.700009	2.330542
192	1	0	1.572961	-4.477776	1.881480
193	1	0	5.643861	-5.027987	4.532972
194	6	0	-4.797160	-7.234841	2.362758
195	6	0	-8.411441	2.276965	2.327449
196	1	0	-0.010801	-4.215866	3.733319
197	1	0	4.753196	0.130468	1.888369
198	1	0	6.513956	3.888924	4.470969
199	6	0	-5.063127	-8.296762	3.235696
200	6	0	-5.739072	-9.418584	2.758801
201	6	0	-6.135315	-9.452440	1.423408
202	6	0	-5.847100	-8.348175	0.622475
203	7	0	-5.196731	-7.269377	1.076044
204	1	0	-4.778534	-8.242656	4.279688
205	1	0	-5.959490	-10.247059	3.424211
206	1	0	-6.661792	-10.305935	1.010516
207	1	0	-6.146258	-8.321032	-0.421258
208	6	0	-9.517543	2.208845	3.183219
209	6	0	-10.786252	2.494834	2.681861
210	6	0	-10.920047	2.844193	1.339640
211	6	0	-9.767835	2.905898	0.556947
212	7	0	-8.547471	2.630398	1.033825
213	6	0	-0.877079	9.718254	3.192539
214	6	0	-0.977877	11.022253	2.710513
215	6	0	-0.650032	11.279217	1.380907
216	6	0	-0.220593	10.213792	0.590774
217	7	0	-0.123827	8.959578	1.049163
218	6	0	8.925061	3.810782	3.249263
219	6	0	10.142627	4.308603	2.788123
220	6	0	10.514299	4.069553	1.466675
221	6	0	9.650328	3.326466	0.663439
222	7	0	8.479505	2.847613	1.101842
223	6	0	6.332489	-7.331793	3.299476
224	6	0	7.192873	-8.328541	2.842093
225	6	0	7.107466	-8.738255	1.513053
226	6	0	6.148258	-8.137484	0.698829
227	7	0	5.320571	-7.179238	1.133761
228	1	0	-9.394380	1.967997	4.232356
229	1	0	-11.653003	2.453653	3.333618
230	1	0	-11.888292	3.072604	0.907852
231	1	0	-9.818132	3.186642	-0.491037
232	1	0	-1.097170	9.510467	4.232827
233	1	0	-1.298456	11.824043	3.367850
234	1	0	-0.715434	12.278457	0.964545
235	1	0	0.057982	10.364889	-0.448073
236	1	0	8.638986	3.960057	4.283545
237	1	0	10.792728	4.865397	3.455254
238	1	0	11.451867	4.439583	1.066461
239	1	0	9.900287	3.103309	-0.369705
240	1	0	6.365239	-7.027062	4.338652
241	1	0	7.910823	-8.782424	3.517574
242	1	0	7.758649	-9.508750	1.115057
243	1	0	6.034105	-8.430753	-0.340691
244	47	0	-6.536527	3.360094	-0.024871
245	47	0	1.202925	7.286426	-0.013174
246	6	0	-1.484619	0.559438	-0.078714
247	6	0	-1.065678	0.311285	1.379174
248	6	0	-0.533759	1.593703	-0.710561
249	6	0	0.911786	1.061402	-0.667266
250	6	0	1.325498	0.810970	0.794669
251	6	0	0.376276	-0.222497	1.429323
252	6	0	-1.398937	-0.764705	-0.862248
253	6	0	0.457184	-1.547309	0.648033
254	6	0	0.984227	-0.262879	-1.448662
255	6	0	0.044026	-1.304621	-0.815297
256	1	0	0.102579	-2.241657	-1.384246
257	1	0	-2.515817	0.940373	-0.108023
258	1	0	-1.137297	1.239696	1.955728
259	1	0	-1.745554	-0.409331	1.847900
260	1	0	-0.825905	1.793921	-1.747105
261	1	0	-0.599876	2.545333	-0.162528
262	1	0	1.585845	1.794846	-1.130405
263	1	0	1.292924	1.745563	1.370108
264	1	0	2.361357	0.445027	0.836289
265	1	0	0.664802	-0.391705	2.467045
266	1	0	-1.704929	-0.606615	-1.901058
267	1	0	-2.092073	-1.500980	-0.429553
268	1	0	-0.200591	-2.293867	1.114683
269	1	0	1.479994	-1.948716	0.693134
270	1	0	0.695688	-0.093284	-2.488497

271	1	0	-2.011956	-0.601096	-1.469611

Ad⊂[biconvex-(*P*,*P*,*P*)-Ag_5**L**$_2$]$^{5+}$:

Center Number	Atomic Number	Atomic Type	Coordinates (Angstroms) X	Y	Z
1	47	0	6.373990	2.686315	0.020863
2	6	0	-0.676767	-5.724138	4.179024
3	6	0	-1.198035	-6.914237	3.690253
4	6	0	-2.085071	-6.892293	2.605324
5	7	0	-2.416287	-5.723498	2.013936
6	6	0	-1.911664	-4.578165	2.489780
7	1	0	0.003253	-5.730002	5.025201
8	1	0	-0.904666	-7.855710	4.138358
9	1	0	-2.201579	-3.674588	1.962050
10	6	0	-2.672581	-8.147705	2.062844
11	6	0	-2.782904	-9.302958	2.846603
12	6	0	-3.324689	-10.457223	2.283188
13	6	0	-3.744150	-10.428420	0.954435
14	6	0	-3.618415	-9.232106	0.249234
15	7	0	-3.098358	-8.121490	0.785355
16	1	0	-2.480198	-9.300218	3.887068
17	1	0	-3.422877	-11.360514	2.876659
18	1	0	-4.165426	-11.305472	0.475285
19	1	0	-3.946856	-9.154242	-0.783052
20	6	0	-1.037306	-4.508175	3.582244
21	6	0	-5.746483	-1.135368	4.227040
22	6	0	-4.986734	0.354324	2.526032
23	7	0	-6.217365	0.479635	2.016077
24	6	0	-7.025897	-1.008801	3.701421
25	6	0	-7.245952	-0.180176	2.592586
26	1	0	-5.568804	-1.769262	5.090301
27	6	0	-8.603140	-0.015034	2.004371
28	1	0	-4.199242	0.890668	2.006740
29	1	0	-7.845502	-1.564975	4.139792
30	6	0	-9.761390	-0.258129	2.753016
31	6	0	-11.007096	-0.105464	2.146300
32	6	0	-11.065121	0.287540	0.810431
33	6	0	-9.865483	0.529320	0.141886
34	7	0	-8.666908	0.383614	0.719217
35	1	0	-9.700173	-0.534454	3.799029
36	1	0	-11.915726	-0.284697	2.712053
37	1	0	-12.013021	0.412081	0.298315
38	1	0	-9.858697	0.854553	-0.894367
39	6	0	-4.682554	-0.436549	3.641540
40	6	0	-2.948448	5.067163	4.156362
41	6	0	-1.289422	4.814101	2.461672
42	7	0	-1.548213	6.026582	1.958329
43	6	0	-3.225936	6.323051	3.633168
44	6	0	-2.497529	6.796987	2.533201
45	1	0	-3.497133	4.699362	5.017898
46	6	0	-2.752301	8.145213	1.956063
47	1	0	-0.534291	4.234464	1.940143
48	1	0	-4.012927	6.925653	4.069703
49	6	0	-3.363447	9.159781	2.703454
50	6	0	-3.593209	10.398984	2.107848
51	6	0	-3.205151	10.595415	0.783859
52	6	0	-2.583885	9.540672	0.116132
53	7	0	-2.362195	8.348548	0.682788
54	1	0	-3.632132	9.002211	3.741259
55	1	0	-4.061263	11.198230	2.673472
56	1	0	-3.370308	11.541773	0.280470
57	1	0	-2.246586	9.650543	-0.910407
58	6	0	-1.948685	4.275605	3.574794
59	6	0	5.243050	-2.328705	4.173788
60	6	0	6.220270	-3.197098	3.707068
61	6	0	5.937409	-4.050881	2.632192
62	7	0	4.729799	-4.014001	2.027431
63	6	0	3.791265	-3.172413	2.478865
64	1	0	5.448614	-1.673342	5.014463
65	1	0	7.201562	-3.200789	4.165567
66	1	0	2.850436	-3.173127	1.937227
67	6	0	6.955780	-5.005270	2.114884
68	6	0	8.017852	-5.447358	2.913569
69	6	0	8.954217	-6.327349	2.373192
70	6	0	8.805806	-6.745153	1.051926
71	6	0	7.708987	-6.274430	0.331062
72	7	0	6.807590	-5.428888	0.844898
73	1	0	8.102526	-5.137195	3.948382
74	1	0	9.780984	-6.684638	2.978666
75	1	0	9.514702	-7.423946	0.590424
76	1	0	7.540058	-6.584813	-0.695920
77	6	0	3.981646	-2.305179	3.562739
78	6	0	3.802524	4.361433	4.102889
79	6	0	4.921550	5.023687	3.616942
80	6	0	5.651133	4.471856	2.554845
81	7	0	5.254427	3.315225	1.980570
82	6	0	4.171797	2.682631	2.449368
83	1	0	3.241655	4.773516	4.936031
84	1	0	5.218519	5.969942	4.052319
85	1	0	3.891194	1.773130	1.929175
86	6	0	6.866865	5.142647	2.018623
87	6	0	7.603159	6.051216	2.789140
88	6	0	8.724800	6.663129	2.231773
89	6	0	9.084603	6.351176	0.922016
90	6	0	8.310179	5.420902	0.229720
91	7	0	7.231667	4.831682	0.759738
92	1	0	7.328923	6.260145	3.816384
93	1	0	9.310405	7.365759	2.815868
94	1	0	9.946460	6.808204	0.448091

271	1	0	2.015598	-0.638972	-1.459968

Ad⊂[biconvex-(*M*,*M*,*M*)-Ag_5**L**$_2$]$^{5+}$:

Center Number	Atomic Number	Atomic Type	Coordinates (Angstroms) X	Y	Z
1	47	0	-6.799189	-1.239459	-0.020614
2	6	0	1.904125	5.432132	-4.181410
3	6	0	2.668321	6.483064	-3.693312
4	6	0	3.529502	6.272742	-2.607755
5	7	0	3.601961	5.060601	-2.015009
6	6	0	2.863750	4.049750	-2.490417
7	1	0	1.241197	5.582984	-5.027713
8	1	0	2.583352	7.465125	-4.142057
9	1	0	2.952962	3.105483	-1.961715
10	6	0	4.369952	7.375525	-2.066159
11	6	0	4.724054	8.479129	-2.852236
12	6	0	5.495155	9.494909	-2.289597
13	6	0	5.894511	9.382515	-0.959103
14	6	0	5.517553	8.241608	-0.251721
15	7	0	4.777176	7.263427	-0.787128
16	1	0	4.430627	8.537437	-3.893687
17	1	0	5.783172	10.355505	-2.884739
18	1	0	6.489219	10.152754	-0.480234
19	1	0	5.818824	8.100498	0.782051
20	6	0	1.994831	4.167971	-3.582880
21	6	0	5.860386	-0.154341	-4.240223
22	6	0	4.799007	-1.425464	-2.524393
23	7	0	5.973416	-1.812579	-2.013856
24	6	0	7.082659	-0.552677	-3.714144
25	6	0	7.119966	-1.399100	-2.597399
26	1	0	5.823225	0.494969	-5.109457
27	6	0	8.411035	-1.849525	-2.009355
28	1	0	3.916507	-1.769209	-1.995962
29	1	0	8.002587	-0.192892	-4.158511
30	6	0	9.592230	-1.867655	-2.761457
31	6	0	10.777823	-2.279649	-2.155027
32	6	0	10.754199	-2.665131	-0.815994
33	6	0	9.532591	-2.638215	-0.144100
34	7	0	8.391656	-2.242425	-0.720956
35	1	0	9.589073	-1.593014	-3.809698
36	1	0	11.701963	-2.304502	-2.723491
37	1	0	11.654738	-2.986449	-0.304073
38	1	0	9.459226	-2.946628	0.894685
39	6	0	4.671053	-0.598350	-3.647537
40	6	0	1.774894	-5.596525	-4.159998
41	6	0	0.217101	-4.979752	-2.462134
42	7	0	0.203027	-6.218338	-1.956284
43	6	0	1.771201	-6.881043	-3.632745
44	6	0	0.958396	-7.180003	-2.530350
45	1	0	2.388336	-5.360679	-5.024021
46	6	0	0.912303	-8.548850	-1.947621
47	1	0	-0.388936	-4.245619	-1.940706
48	1	0	2.405650	-7.643017	-4.068532
49	6	0	1.290997	-9.675745	-2.687979
50	6	0	1.243571	-10.932201	-2.086057
51	6	0	0.817028	-11.032778	-0.763169
52	6	0	0.437249	-9.864872	-0.102835
53	7	0	0.482359	-8.655954	-0.675426
54	1	0	1.591470	-9.585747	-3.725106
55	1	0	1.528915	-11.816948	-2.646056
56	1	0	0.770551	-11.989828	-0.255069
57	1	0	0.079799	-9.893835	0.922346
58	6	0	0.975089	-4.603078	-3.578799
59	6	0	-4.611129	3.402888	-4.177633
60	6	0	-5.378536	4.461545	-3.711329
61	6	0	-4.919253	5.234117	-2.635839
62	7	0	-3.747218	4.939130	-2.031865
63	6	0	-3.012088	3.915117	-2.483197
64	1	0	-4.952764	2.806843	-5.018155
65	1	0	-6.335917	4.676082	-4.170094
66	1	0	-2.093257	3.713955	-1.942128
67	6	0	-5.710949	6.382443	-2.116094
68	6	0	-6.651822	7.044585	-2.914675
69	6	0	-7.382381	8.099890	-2.371011
70	6	0	-7.154338	8.468603	-1.046558
71	6	0	-6.184978	7.771983	-0.326067
72	7	0	-5.481025	6.757623	-0.842976
73	1	0	-6.796620	6.765773	-3.951704
74	1	0	-8.112086	8.628048	-2.976288
75	1	0	-7.705214	9.279391	-0.582398
76	1	0	-5.958981	8.033640	0.703445
77	6	0	-3.384839	3.107987	-3.565720
78	6	0	-4.653486	-3.441295	-4.099299
79	6	0	-5.887897	-3.846173	-3.610062
80	6	0	-6.479469	-3.148473	-2.547980
81	7	0	-5.842707	-2.102143	-1.978011
82	6	0	-4.650488	-1.718136	-2.450392
83	1	0	-4.195745	-3.966151	-4.932057
84	1	0	-6.382360	-4.707274	-4.042690
85	1	0	-4.179487	-0.888347	-1.934812
86	6	0	-7.809553	-3.542976	-2.008911
87	6	0	-8.726126	-4.268421	-2.780072
88	6	0	-9.951565	-4.627332	-2.221006
89	6	0	-10.232183	-4.251463	-0.908726
90	6	0	-9.274018	-3.512487	-0.215794
91	7	0	-8.095331	-3.166553	-0.747402
92	1	0	-8.506230	-4.526728	-3.809177
93	1	0	-10.676186	-5.184887	-2.805685
94	1	0	-11.170775	-4.514744	-0.433345

95	1	0	8.560230	5.131244	-0.786746	95	1	0	-9.453476	-3.181090	0.802826
96	6	0	3.402042	3.149154	3.523315	96	6	0	-4.000605	-2.342374	-3.523324
97	6	0	0.986076	3.132219	4.035718	97	6	0	-1.639204	-2.849360	-4.038439
98	6	0	2.194009	2.430037	3.994791	98	6	0	-2.666211	-1.902062	-3.997385
99	6	0	2.242279	1.012283	4.337502	99	6	0	-2.406348	-0.507819	-4.340943
100	6	0	1.067193	0.515590	4.872171	100	6	0	-1.150876	-0.277910	-4.874703
101	6	0	-0.273347	2.514255	4.379579	101	6	0	-0.276333	-2.520931	-4.385827
102	6	0	-0.153887	1.237528	4.898388	102	6	0	-0.115727	-1.247950	-4.902628
103	6	0	-1.218508	0.300280	4.931443	103	6	0	1.127413	-0.564732	-4.935208
104	6	0	-1.642964	2.912433	4.071115	104	6	0	0.974151	-3.207565	-4.079053
105	6	0	-2.688065	1.986354	4.135633	105	6	0	2.195942	-2.531434	-4.144683
106	6	0	-2.483101	0.592902	4.453350	106	6	0	2.298926	-1.126041	-4.459486
107	6	0	-0.654420	-1.001456	4.913193	107	6	0	0.859519	0.828376	-4.914717
108	6	0	-1.329357	-2.099948	4.411519	108	6	0	1.757436	1.753206	-4.412727
109	6	0	-2.724619	-1.857885	4.128207	109	6	0	3.067684	1.213795	-4.132995
110	6	0	-3.286930	-0.578242	4.122744	110	6	0	3.338599	-0.157661	-4.128379
111	6	0	0.757665	-0.868828	4.883623	111	6	0	-0.547793	1.005901	-4.883840
112	6	0	1.589927	-1.842632	4.361524	112	6	0	-1.148161	2.136524	-4.359762
113	6	0	0.922935	-3.084449	4.050782	113	6	0	-0.227046	3.203111	-4.048227
114	6	0	-0.468003	-3.219960	4.047918	114	6	0	1.160238	3.033019	-4.046699
115	6	0	3.221691	-0.001528	4.024023	115	6	0	-3.142332	0.694569	-4.027664
116	6	0	2.925004	-1.367226	4.013362	116	6	0	-2.555457	1.963052	-4.013917
117	1	0	1.506394	-3.934660	3.712819	117	1	0	-0.611615	4.159924	-3.710076
118	1	0	-3.354882	-2.680081	3.804782	118	1	0	3.862272	1.879337	-3.811411
119	1	0	-3.672844	2.325003	3.830474	119	1	0	3.083482	-3.076653	-3.840683
120	1	0	1.001797	4.167495	3.711117	120	1	0	-1.879650	-3.856238	-3.713549
121	1	0	4.212227	0.294157	3.694565	121	1	0	-4.173968	0.621367	-3.699825
122	6	0	2.092509	5.400299	-4.124402	122	6	0	-3.221603	-4.808462	4.121929
123	6	0	2.142652	6.695156	-3.626472	123	6	0	-3.548726	-6.062722	3.624786
124	6	0	1.316813	7.062406	-2.555073	124	6	0	-2.818257	-6.600508	2.556154
125	7	0	0.497308	6.154871	-1.980397	125	7	0	-1.821822	-5.891367	1.982624
126	6	0	0.457686	4.905066	-2.458908	126	6	0	-1.514271	-4.679098	2.460382
127	1	0	2.715963	5.112361	-4.965200	127	1	0	-3.770011	-4.391921	4.961180
128	1	0	2.828265	7.412475	-4.060580	128	1	0	-4.373570	-6.615095	4.057902
129	1	0	-0.201049	4.217653	-1.938075	129	1	0	-0.721213	-4.150040	1.941488
130	6	0	1.326339	8.447548	-2.009693	130	6	0	-3.120812	-7.952477	2.011593
131	6	0	1.770577	9.533643	-2.774233	131	6	0	-3.792528	-8.916974	2.773074
132	6	0	1.774596	10.807428	-2.208048	132	6	0	-4.065179	-10.161302	2.207021
133	6	0	1.333125	10.965698	-0.895645	133	6	0	-3.658496	-10.412276	0.897774
134	6	0	0.886983	9.836501	-0.209999	134	6	0	-2.975516	-9.406055	0.215533
135	7	0	0.882103	8.611332	-0.748673	135	7	0	-2.711897	-8.209694	0.754310
136	1	0	2.082182	9.399808	-3.803325	136	1	0	-4.075448	-8.717658	3.799840
137	1	0	2.110758	11.661393	-2.787302	137	1	0	-4.580424	-10.922643	2.783782
138	1	0	1.325289	11.937839	-0.414855	138	1	0	-3.855551	-11.364247	0.416940
139	1	0	0.516000	9.910700	0.808054	139	1	0	-2.621335	-9.559598	-0.799650
140	6	0	1.224363	4.464016	-3.546141	140	6	0	-2.170300	-4.082309	3.545514
141	6	0	-4.470234	3.547306	-4.182006	141	6	0	3.584992	-4.430680	4.176289
142	6	0	-4.508578	1.874916	-2.480510	142	6	0	3.990531	-2.799740	2.482615
143	7	0	-5.682594	2.313636	-2.008635	143	7	0	5.043420	-3.480570	2.011873
144	6	0	-5.685514	4.007929	-3.694451	144	6	0	4.673157	-5.141879	3.689569
145	6	0	-6.289577	3.362071	-2.607071	145	6	0	5.406818	-4.637902	2.607040
146	1	0	-4.001418	4.032895	-5.032265	146	1	0	3.019581	-4.806427	5.023468
147	6	0	-7.601148	3.818027	-2.070767	147	6	0	6.592691	-5.362983	2.074069
148	1	0	-4.061315	1.043881	-1.943956	148	1	0	3.736462	-1.889417	1.948367
149	1	0	-6.154749	4.872823	-4.147112	149	1	0	4.942689	-6.089335	4.139974
150	6	0	-8.501264	4.546682	-2.858338	150	6	0	7.309946	-6.272245	2.861506
151	6	0	-9.707996	4.965575	-2.300238	151	6	0	8.402594	-6.937242	2.307368
152	6	0	-9.986796	4.644915	-0.972905	152	6	0	8.753203	-6.676232	0.983945
153	6	0	-9.046761	3.897870	-0.264055	153	6	0	8.000188	-5.741206	0.274921
154	7	0	-7.886355	3.494041	-0.794979	154	7	0	6.949625	-5.101054	0.802095
155	1	0	-8.283561	4.762019	-3.897742	155	1	0	7.043754	-6.441366	3.898185
156	1	0	-10.419843	5.526935	-2.896863	156	1	0	8.973353	-7.641482	2.904096
157	1	0	-10.910758	4.956281	-0.497920	157	1	0	9.592381	-7.175637	0.512103
158	1	0	-9.226142	3.606758	0.766753	158	1	0	8.244916	-5.488747	-0.752621
159	6	0	-3.850546	2.444034	-3.579330	159	6	0	3.221215	-3.216762	3.577765
160	6	0	-4.733572	-3.258263	-4.230750	160	6	0	5.325123	2.156746	4.231680
161	6	0	-3.183036	-3.798930	-2.499785	161	6	0	3.929080	3.021820	2.501113
162	7	0	-3.975945	-4.768656	-2.027211	162	7	0	4.913596	3.796704	2.029573
163	6	0	-5.559555	-4.262227	-3.743840	163	6	0	6.349284	2.958474	3.746051
164	6	0	-5.153824	-5.022335	-2.638463	164	6	0	6.118098	3.789298	2.641304
165	1	0	-5.034794	-2.670455	-5.092300	165	1	0	5.491704	1.517128	5.092899
166	6	0	-6.009029	-6.112308	-2.094326	166	6	0	7.187181	4.670351	2.096813
167	1	0	-2.261166	-3.628480	-1.952943	167	1	0	2.992577	3.056758	1.953568
168	1	0	-6.520791	-4.442531	-4.209347	168	1	0	7.326439	2.925943	4.212177
169	6	0	-6.972807	-6.751062	-2.884420	169	6	0	8.269092	5.084645	2.883580
170	6	0	-7.760163	-7.752850	-2.319016	170	6	0	9.250365	5.896329	2.316502
171	6	0	-7.564293	-8.093002	-0.981749	171	6	0	9.125604	6.275876	0.981216
172	6	0	-6.569658	-7.423190	-0.270138	172	6	0	8.007179	5.837572	0.273136
173	7	0	-5.811616	-6.460004	-0.807967	173	7	0	7.062995	5.057368	0.812664
174	1	0	-7.092313	-6.496078	-3.930787	174	1	0	8.335560	4.806769	3.928795
175	1	0	-8.508596	-8.262290	-2.917440	175	1	0	10.093314	6.231321	2.912317
176	1	0	-8.158976	-8.862015	-0.500863	176	1	0	9.868919	6.901572	0.499278
177	1	0	-6.366620	-7.664981	0.769036	177	1	0	7.855066	6.121651	-0.764088
178	6	0	-3.501822	-3.007533	-3.610893	178	6	0	4.068387	2.179066	3.611342
179	6	0	5.880124	-0.263691	-4.125484	179	6	0	-5.685252	1.548600	4.115576
180	6	0	7.122742	0.082888	-3.612274	180	6	0	-6.972553	1.477661	3.600099
181	6	0	7.207475	0.967495	-2.528114	181	6	0	-7.244675	0.626978	2.519614
182	7	0	6.085813	1.459070	-1.957392	182	7	0	-6.255199	-0.098389	1.954181
183	6	0	4.889928	1.116804	-2.451326	183	6	0	-5.014468	-0.021386	2.449803
184	1	0	5.806374	-0.935540	-4.975029	184	1	0	-5.469275	2.191904	4.962967
185	1	0	8.020275	-0.342210	-4.044354	185	1	0	-7.757706	2.088779	4.027980
186	1	0	4.027125	1.524916	-1.933978	186	1	0	-4.259445	-0.609409	1.937017
187	6	0	8.522495	1.378904	-1.963947	187	6	0	-8.615983	0.504354	1.952882
188	6	0	9.702146	1.291519	-2.713759	188	6	0	-9.751167	0.856535	2.693519
189	6	0	10.909032	1.674121	-2.130531	189	6	0	-11.010294	0.735411	2.107699
190	6	0	10.908249	2.136996	-0.816026	190	6	0	-11.105441	0.262159	0.800335
191	6	0	9.688186	2.213527	-0.145354	191	6	0	-9.928647	-0.087719	0.139175
192	7	0	8.527052	1.846256	-0.700492	192	7	0	-8.717553	0.029015	0.696572
193	1	0	9.683457	0.958877	-3.744748	193	1	0	-9.664303	1.193166	3.719776
194	1	0	11.832088	1.617448	-2.698447	194	1	0	-11.901092	0.999469	2.668523
195	1	0	11.825268	2.439394	-0.322168	195	1	0	-12.064567	0.157305	0.305012
196	1	0	9.632878	2.584659	0.873844	196	1	0	-9.951050	-0.478774	-0.873887
197	6	0	4.716388	0.264863	-3.550326	197	6	0	-4.662844	0.777125	3.546201

198	6	0	1.672510	-5.610498	-4.177614
199	6	0	2.378991	-6.693237	-3.671544
200	6	0	3.221801	-6.519343	-2.565085
201	7	0	3.330047	-5.311631	-1.969547
202	6	0	2.643963	-4.271606	-2.458704
203	1	0	1.026681	-5.732789	-5.041598
204	1	0	2.264073	-7.670394	-4.124414
205	1	0	2.754767	-3.333579	-1.923224
206	6	0	4.005131	-7.653384	-2.002854
207	6	0	4.316650	-8.781720	-2.771639
208	6	0	5.036431	-9.823818	-2.189306
209	6	0	5.429025	-9.712640	-0.856751
210	6	0	5.097159	-8.547106	-0.167113
211	7	0	4.405468	-7.544055	-0.721284
212	1	0	4.030539	-8.840580	-3.815086
213	1	0	5.290891	-10.703774	-2.771303
214	1	0	5.984763	-10.502362	-0.363033
215	1	0	5.396480	-8.405382	0.867223
216	6	0	1.800677	-4.351879	-3.574417
217	6	0	-0.360395	-3.275242	-4.086004
218	6	0	1.032969	-3.178057	-4.056913
219	6	0	1.701965	-1.928085	-4.399738
220	6	0	0.867047	-0.955696	-4.921721
221	6	0	-1.216089	-2.157679	-4.404445
222	6	0	-0.548536	-1.059804	-4.918954
223	6	0	-1.085084	0.253828	-4.912493
224	6	0	-2.614454	-1.912302	-4.072042
225	6	0	-3.136529	-0.616612	-4.092932
226	6	0	-2.335832	0.544838	-4.396082
227	6	0	-0.001613	1.170150	-4.908899
228	6	0	-0.111396	2.444345	-4.380064
229	6	0	-1.463037	2.843220	-4.067987
230	6	0	-2.534564	1.946634	-4.049804
231	6	0	1.205539	0.423040	-4.915924
232	6	0	2.384880	0.917002	-4.386380
233	6	0	2.345526	2.322597	-4.058361
234	6	0	1.161531	3.063659	-4.030070
235	6	0	3.028693	-1.460319	-4.076183
236	6	0	3.367641	-0.105060	-4.043215
237	1	0	3.251010	2.819077	-3.725255
238	1	0	-1.657862	3.858143	-3.737459
239	1	0	-4.165057	-0.488093	-3.771487
240	1	0	-0.802066	-4.211006	-3.758726
241	1	0	3.779153	-2.170801	-3.744713
242	47	0	-0.601720	6.834590	-0.025611
243	47	0	-6.661040	1.561189	-0.007287
244	47	0	-3.599524	-5.885930	-0.002883
245	47	0	4.523060	-5.250773	0.048821
246	6	0	-1.582920	0.476621	0.230954
247	6	0	-0.858918	0.229999	1.563644
248	6	0	-0.845730	1.585587	-0.543182
249	6	0	0.607498	1.147814	-0.807528
250	6	0	1.325905	0.899863	0.529986
251	6	0	0.593189	-0.207916	1.307125
252	6	0	-1.583669	-0.828396	-0.586759
253	6	0	0.592253	-1.509815	0.486161
254	6	0	0.592535	-0.155586	-1.624700
255	6	0	-0.131379	-1.270510	-0.850501
256	1	0	-0.126005	-2.188578	-1.450738
257	1	0	-2.618922	0.791405	0.422048
258	1	0	-0.869524	1.142492	2.165487
259	1	0	-1.382835	-0.542413	2.133985
260	1	0	-1.352328	1.790615	-1.493663
261	1	0	-0.863636	2.517586	0.041628
262	1	0	1.130438	1.926123	-1.376619
263	1	0	1.352362	1.819516	1.129850
264	1	0	2.369689	0.603766	0.345781
265	1	0	1.094403	-0.377165	2.260064
266	1	0	-2.104612	-0.685377	-1.540766
267	1	0	-2.127370	-1.610161	-0.035987
268	1	0	0.100223	-2.310110	1.056002
269	1	0	1.625897	-1.837421	0.297735
270	1	0	0.082406	0.016485	-2.572908
271	1	0	1.617664	-0.464235	-1.858946

Ad⊂[biconvex-(*P*,*M*,*P*)-Hg_5**L**$_2$]$^{10+}$:

Center	Atomic	Atomic	Coordinates (Angstroms)		
Number	Number	Type	X	Y	Z
1	80	0	-7.766594	-0.104048	-0.002629
2	80	0	-2.308527	-7.413111	-0.022238
3	80	0	6.372022	-4.489412	-0.011676
4	6	0	5.064050	2.231131	4.441067
5	6	0	6.318042	2.758582	4.169162
6	6	0	6.529372	3.507253	3.000859
7	7	0	5.512406	3.674307	2.120198
8	6	0	3.995738	2.449518	3.553323
9	6	0	4.292428	3.170112	2.390671
10	1	0	4.902659	1.673515	5.358750
11	1	0	7.129783	2.584440	4.863908
12	1	0	3.521183	3.347853	1.646258
13	6	0	-5.432347	1.069101	4.476639
14	6	0	-4.684049	0.288566	3.577821
15	6	0	-5.330344	-0.090363	2.396184
16	7	0	-6.610994	0.224858	2.117071
17	6	0	-6.753795	1.384382	4.195939
18	6	0	-7.348262	0.932849	3.007353
19	1	0	-4.985501	1.403913	5.407991
20	6	0	-8.777702	1.232729	2.703887
21	6	0	-2.739547	-4.873047	4.481100

198	6	0	-0.413198	5.847246	4.171685
199	6	0	-0.867694	7.056414	3.662823
200	6	0	-1.729796	7.066980	2.557714
201	7	0	-2.097095	5.910252	1.964321
202	6	0	-1.652677	4.747179	2.455767
203	1	0	0.243891	5.828567	5.035657
204	1	0	-0.543477	7.986484	4.113328
205	1	0	-1.964939	3.854274	1.922719
206	6	0	-2.252259	8.342298	1.995131
207	6	0	-2.304263	9.514388	2.759771
208	6	0	-2.789233	10.684758	2.178163
209	6	0	-3.213225	10.655009	0.850861
210	6	0	-3.148842	9.442380	0.165528
211	7	0	-2.682254	8.316555	0.718858
212	1	0	-2.000633	9.514069	3.799981
213	1	0	-2.841296	11.601257	2.757060
214	1	0	-3.592204	11.543722	0.358088
215	1	0	-3.485405	9.363180	-0.864067
216	6	0	-0.812117	4.645084	3.571707
217	6	0	1.062269	3.124244	4.086390
218	6	0	-0.318680	3.333620	4.057931
219	6	0	-1.244373	2.260634	4.402598
220	6	0	-0.642019	1.129062	4.924115
221	6	0	1.652943	1.846675	4.404679
222	6	0	0.762282	0.921281	4.920276
223	6	0	0.999133	-0.477768	4.912964
224	6	0	2.963926	1.302546	4.070911
225	6	0	3.190664	-0.075901	4.090780
226	6	0	2.155773	-1.034306	4.395076
227	6	0	-0.258110	-1.135382	4.908988
228	6	0	-0.428512	-2.403022	4.380602
229	6	0	0.803470	-3.087741	4.069164
230	6	0	2.044604	-2.446078	4.049598
231	6	0	-1.273152	-0.142617	4.915787
232	6	0	-2.531202	-0.367170	4.384323
233	6	0	-2.798882	-1.747497	4.055500
234	6	0	-1.805407	-2.729351	4.029986
235	6	0	-2.640865	2.093494	4.077402
236	6	0	-3.267182	0.844819	4.042141
237	1	0	-3.790237	-2.034596	3.720578
238	1	0	0.771689	-4.120467	3.737592
239	1	0	4.166046	-0.426430	3.768729
240	1	0	1.697863	3.940233	3.757589
241	1	0	-3.217783	2.950517	3.745331
242	47	0	-0.894114	-6.797441	0.030909
243	47	0	6.170629	-2.949064	0.018386
244	47	0	4.789938	4.967207	0.004692
245	47	0	-3.276366	6.104542	-0.054865
246	6	0	1.460282	-0.797182	-0.218244
247	6	0	0.805284	-0.420698	-1.555647
248	6	0	0.519971	-1.742109	0.552006
249	6	0	-0.823185	-1.032487	0.807179
250	6	0	-1.474195	-0.654577	-0.534782
251	6	0	-0.536935	0.288798	-1.308486
252	6	0	1.707220	0.485492	0.597647
253	6	0	-0.291421	1.568019	-0.488906
254	6	0	-0.564139	0.245760	1.623011
255	6	0	0.365393	1.199484	0.852948
256	1	0	0.533173	2.103071	1.452495
257	1	0	2.414945	-1.308689	-0.404332
258	1	0	0.644999	-1.320612	-2.155705
259	1	0	1.470958	0.235484	-2.123394
260	1	0	0.972725	-2.038987	1.505136
261	1	0	0.362164	-2.662123	-0.031145
262	1	0	-1.488313	-1.694524	1.374243
263	1	0	-1.676736	-1.552338	-1.134616
264	1	0	-2.441298	-0.160999	-0.355487
265	1	0	-0.990326	0.550187	-2.264560
266	1	0	2.186974	0.246793	1.554186
267	1	0	2.393468	1.146973	0.048526
268	1	0	0.346764	2.258649	-1.056867
269	1	0	-1.244569	2.087042	-0.307436
270	1	0	-0.104491	-0.018369	2.575797
271	1	0	-1.513369	0.745176	1.847524

Ad⊂[biconvex-(*M*,*P*,*M*)-Hg_5**L**$_2$]$^{10+}$:

Center	Atomic	Atomic	Coordinates (Angstroms)		
Number	Number	Type	X	Y	Z
1	80	0	5.510645	-5.482027	0.000995
2	80	0	-3.529832	-6.919251	-0.017027
3	80	0	-7.698210	1.208547	-0.014779
4	6	0	-2.077437	5.137139	4.441476
5	6	0	-2.605957	6.390486	4.168700
6	6	0	-2.235828	7.071973	2.998713
7	7	0	-1.394893	6.478614	2.116524
8	6	0	-1.161908	4.545648	3.553190
9	6	0	-0.874028	5.265769	2.388069
10	1	0	-2.350846	4.626461	5.359966
11	1	0	-3.307086	6.834271	4.863939
12	1	0	-0.201125	4.852092	1.642042
13	6	0	4.637141	-3.015520	4.480328
14	6	0	3.560132	-3.051027	3.577089
15	6	0	3.763248	-3.772034	2.395312
16	7	0	4.900987	-4.441014	2.120337
17	6	0	5.804448	-3.711818	4.203392
18	6	0	5.919383	-4.449641	3.015025
19	1	0	4.547231	-2.465096	5.412064
20	6	0	7.153977	-5.231993	2.716590
21	6	0	-1.428677	-5.397212	4.474931

22	6	0	-3.445354	-6.030076	4.183918
23	6	0	-3.193312	-6.722176	2.989022
24	7	0	-2.287517	-6.230098	2.108832
25	6	0	-1.762900	-4.391987	3.591625
26	6	0	-1.595900	-5.111668	2.403214
27	1	0	-2.924364	-4.355361	5.417612
28	1	0	-4.192987	-6.389601	4.879389
29	1	0	-0.881643	-4.775242	1.656773
30	6	0	3.732327	-4.147739	4.440322
31	6	0	4.620203	-5.174504	4.153504
32	6	0	5.382172	-5.139002	2.975188
33	7	0	5.211822	-4.118658	2.099211
34	6	0	3.595272	-3.062358	3.557023
35	6	0	4.356233	-3.117103	2.383677
36	1	0	3.164737	-4.170455	5.365793
37	1	0	4.716443	-6.001888	4.844944
38	6	0	-0.950039	-3.193923	3.939520
39	6	0	0.445159	-3.281252	3.929343
40	6	0	1.306776	-2.163334	4.257316
41	6	0	0.641276	-1.062777	4.768463
42	6	0	-1.615094	-1.935209	4.279185
43	6	0	-0.777403	-0.958224	4.788718
44	6	0	-1.115261	0.424398	4.770372
45	6	0	-2.940647	-1.465535	3.929041
46	6	0	-3.290637	-0.112856	3.918019
47	6	0	-2.299660	0.912911	4.245518
48	6	0	0.095300	1.169744	4.734329
49	6	0	0.198384	2.440706	4.197982
50	6	0	-1.082416	3.065788	3.864126
51	6	0	-2.260795	2.314356	3.877372
52	6	0	1.179591	0.252143	4.742020
53	6	0	2.430790	0.545136	4.229489
54	6	0	2.631847	1.954174	3.883289
55	6	0	1.549390	2.838221	3.858481
56	6	0	2.705666	-1.918962	3.900720
57	6	0	3.221224	-0.619322	3.888751
58	1	0	0.889072	-4.224488	3.624163
59	1	0	-3.179340	2.809870	3.576127
60	1	0	1.740188	3.861529	3.548235
61	1	0	4.253890	-0.489545	3.577887
62	6	0	-0.605503	5.460371	4.433585
63	6	0	-1.133682	4.517332	3.533973
64	6	0	-1.717053	5.029251	2.369593
65	7	0	-1.819584	6.347793	2.108481
66	6	0	-0.720755	6.817680	4.172014
67	6	0	-1.358581	7.257207	3.001568
68	1	0	-0.131389	5.128136	5.352240
69	6	0	-1.526451	8.712747	2.721865
70	6	0	7.861148	4.111994	2.708061
71	1	0	-4.799156	-0.665020	1.642638
72	1	0	-7.318008	1.984211	4.898592
73	6	0	-3.918498	-7.985297	2.666916
74	6	0	6.369291	-6.214059	2.667009
75	1	0	-3.698331	-2.184660	3.631207
76	1	0	-2.116015	4.356682	1.615395
77	1	0	-0.312073	7.531568	4.875753
78	7	0	-9.197454	1.103957	1.423015
79	6	0	-10.471505	1.390323	1.096647
80	6	0	-11.404602	1.816334	2.039749
81	6	0	-10.997977	1.925360	3.368587
82	6	0	-9.672419	1.633123	3.701660
83	1	0	-10.744458	1.279809	0.051989
84	1	0	-12.421446	2.043922	1.736687
85	1	0	-11.699855	2.228296	4.139847
86	1	0	-9.363600	1.693395	4.737861
87	7	0	-1.800702	9.089816	1.450695
88	6	0	-1.933174	10.394109	1.145549
89	6	0	-1.806052	11.399655	2.101754
90	6	0	-1.554264	11.028095	3.421472
91	6	0	-1.411203	9.672965	3.732288
92	7	0	8.119190	4.498610	1.436212
93	6	0	9.317285	5.023573	1.118693
94	6	0	10.327445	5.199451	2.062191
95	6	0	10.068515	4.835613	3.382669
96	6	0	8.825278	4.285174	3.706427
97	7	0	6.805784	-6.331076	1.390537
98	6	0	7.675698	-7.303347	1.059642
99	6	0	8.167218	-8.213192	1.993591
100	6	0	7.752183	-8.088762	3.318470
101	6	0	6.843669	-7.081762	3.656119
102	6	0	-4.584332	-8.722423	3.651634
103	6	0	-5.271773	-9.887481	3.300277
104	6	0	-5.285140	-10.292526	1.966522
105	6	0	-4.582685	-9.527912	1.037273
106	7	0	-3.916501	-8.410048	1.381186
107	1	0	-2.140049	10.634019	0.107460
108	1	0	-1.913140	12.440937	1.815597
109	1	0	-1.474634	11.778087	4.202757
110	1	0	-1.241662	9.382868	4.761789
111	1	0	9.468363	5.303101	0.080883
112	1	0	11.282851	5.620364	1.766141
113	1	0	10.818160	4.981027	4.154751
114	1	0	8.614870	4.026859	4.736824
115	1	0	7.978282	-7.353834	0.018538
116	1	0	8.862873	-8.987520	1.686856
117	1	0	8.131033	-8.760142	4.083291
118	1	0	6.541313	-6.969381	4.689866
119	1	0	-4.553144	-8.421843	4.691540
120	1	0	-5.783683	-10.468972	4.061241
121	1	0	-5.816035	-11.184236	1.649317
122	1	0	-4.554453	-9.810049	-0.010390
123	1	0	4.274029	-2.327516	1.641948
124	6	0	3.639749	4.244605	-4.439268

22	6	0	-1.730599	-6.718939	4.180017
23	6	0	-2.399208	-7.040421	2.988473
24	7	0	-2.708309	-6.056551	2.108621
25	6	0	-1.797116	-4.371940	3.586294
26	6	0	-2.423102	-4.772373	2.400802
27	1	0	-0.931747	-5.154393	5.409441
28	1	0	-1.442897	-7.496980	4.875453
29	1	0	-2.703086	-4.033956	1.654597
30	6	0	-5.569410	-0.368227	4.445163
31	6	0	-6.921859	-0.485209	4.158802
32	6	0	-7.442821	0.068360	2.978736
33	7	0	-6.608997	0.678516	2.101236
34	6	0	-4.714315	0.312311	3.560478
35	6	0	-5.297743	0.801017	2.386029
36	1	0	-5.178292	-0.778003	5.371595
37	1	0	-7.568131	-1.009226	4.851245
38	6	0	-1.544994	-2.946125	3.933827
39	6	0	-2.607196	-2.037416	3.928555
40	6	0	-2.446725	-0.636323	4.261062
41	6	0	-1.202232	-0.310888	4.771987
42	6	0	-0.191075	-2.506501	4.273018
43	6	0	-0.111437	-1.224263	4.787958
44	6	0	1.094305	-0.467798	4.771001
45	6	0	1.086959	-3.092919	3.922547
46	6	0	2.280707	-2.367077	3.914238
47	6	0	2.284091	-0.941637	4.244792
48	6	0	0.744965	0.910368	4.736609
49	6	0	1.556071	1.894517	4.200224
50	6	0	2.910547	1.451207	3.865479
51	6	0	3.232532	0.091241	3.878045
52	6	0	-0.672184	1.007355	4.744711
53	6	0	-1.366259	2.089310	4.232472
54	6	0	-0.529288	3.240058	3.885257
55	6	0	0.863125	3.120300	3.859916
56	6	0	-3.279811	0.513358	3.904607
57	6	0	-2.744813	1.804901	3.893365
58	1	0	-3.583364	-2.404316	3.624534
59	1	0	4.237615	-0.192214	3.578896
60	1	0	1.438488	3.986841	3.547158
61	1	0	-3.395903	2.617358	3.583591
62	6	0	4.235521	3.503652	4.432406
63	6	0	3.958047	2.456856	3.535123
64	6	0	4.734241	2.415098	2.371529
65	7	0	5.725355	3.289861	2.108280
66	6	0	5.262833	4.397516	4.168436
67	6	0	6.026330	4.267075	2.997982
68	1	0	3.664698	3.597190	5.351378
69	6	0	7.157277	5.197091	2.713472
70	6	0	-2.764141	8.435737	2.705482
71	1	0	2.984994	-3.810848	1.638259
72	1	0	6.624278	-3.676184	4.909410
73	6	0	-2.763864	-8.451475	2.669930
74	6	0	-8.899659	-0.016480	2.669737
75	1	0	1.129506	-4.136240	3.622826
76	1	0	4.553275	1.651720	1.619952
77	1	0	5.466223	5.196005	4.870508
78	7	0	7.370734	-5.615864	1.436498
79	6	0	8.485840	-6.297024	1.113812
80	6	0	9.448306	-6.642180	2.060419
81	6	0	9.226753	-6.282405	3.388818
82	6	0	8.070663	-5.569207	3.717841
83	1	0	8.608874	-6.564682	0.069180
84	1	0	10.337964	-7.186264	1.760257
85	1	0	9.938209	-6.554637	4.162750
86	1	0	7.886729	-5.312028	4.753549
87	7	0	7.616544	5.270503	1.441846
88	6	0	8.617541	6.115121	1.131560
89	6	0	9.224307	6.932799	2.082763
90	6	0	8.784701	6.848594	3.402896
91	6	0	7.740612	5.974799	3.719098
92	7	0	-2.675917	8.893259	1.434079
93	6	0	-3.163708	10.106934	1.116452
94	6	0	-3.763501	10.939169	2.059380
95	6	0	-3.835610	10.497136	3.379403
96	6	0	-3.333450	9.233660	3.703276
97	7	0	-9.292426	0.195662	1.391437
98	6	0	-10.593037	0.099032	1.058513
99	6	0	-11.580376	-0.208357	1.992447
100	6	0	-11.198141	-0.398282	3.319479
101	6	0	-9.845724	-0.303516	3.659166
102	6	0	-2.793507	-9.443922	3.655164
103	6	0	-3.118950	-10.757862	3.307152
104	6	0	-3.405626	-11.057248	1.976226
105	6	0	-3.382001	-10.019689	1.046415
106	7	0	-3.073609	-8.754669	1.387119
107	1	0	8.933611	6.137038	0.093427
108	1	0	10.024815	7.605584	1.792630
109	1	0	9.248060	7.448434	4.180539
110	1	0	7.417002	5.890639	4.749137
111	1	0	-3.074021	10.412670	0.078881
112	1	0	-4.151388	11.908383	1.763108
113	1	0	-4.271013	11.125037	4.151018
114	1	0	-3.366455	8.901333	4.733398
115	1	0	-10.843850	0.267660	0.016063
116	1	0	-12.618102	-0.283850	1.684189
117	1	0	-11.938690	-0.612555	4.084271
118	1	0	-9.552184	-0.426110	4.694326
119	1	0	-2.595995	-9.207260	4.693300
120	1	0	-3.151726	-11.531280	4.068721
121	1	0	-3.652313	-12.066082	1.661598
122	1	0	-3.609161	-10.201766	0.000781
123	1	0	-4.688681	1.309179	1.643630
124	6	0	0.350183	5.571854	-4.442574

125	6	0	4.497224	5.297141	-4.153218
126	6	0	5.253942	5.288479	-2.970939
127	7	0	5.109543	4.266724	-2.092119
128	6	0	3.529221	3.159569	-3.551656
129	6	0	4.283987	3.240346	-2.376081
130	1	0	3.075151	4.247737	-5.366841
131	1	0	4.573419	6.124112	-4.847614
132	1	0	4.221716	2.451680	-1.631306
133	6	0	-0.443759	-5.495362	-4.466107
134	6	0	-0.992308	-4.559140	-3.572129
135	6	0	-1.557786	-5.076132	-2.400933
136	7	0	-1.625222	-6.394229	-2.128092
137	6	0	-0.522115	-6.853088	-4.191465
138	6	0	-1.142958	-7.299418	-3.014472
139	1	0	0.017628	-5.159334	-5.389900
140	6	0	-1.269206	-8.755995	-2.718638
141	6	0	-5.369663	-1.240728	-4.454809
142	6	0	-6.682829	-1.594958	-4.181358
143	6	0	-7.302218	-1.147251	-3.004130
144	7	0	-6.596956	-0.401087	-2.119340
145	6	0	-4.654969	-0.422935	-3.561475
146	6	0	-5.326117	-0.045919	-2.392883
147	1	0	-4.904036	-1.573767	-5.377540
148	1	0	-7.221082	-2.222211	-4.880541
149	1	0	-4.824905	0.561477	-1.644949
150	6	0	-2.861610	4.776458	-4.463097
151	6	0	-3.603003	5.914557	-4.180806
152	6	0	-3.367983	6.632534	-2.997765
153	7	0	-2.443230	6.183007	-2.114731
154	6	0	-1.864413	4.341425	-3.572213
155	6	0	-1.714885	5.084583	-2.396202
156	1	0	-3.034211	4.238085	-5.390208
157	1	0	-4.365078	6.239217	-4.877781
158	1	0	-0.984427	4.786164	-1.649471
159	6	0	-3.271778	0.017163	-3.894745
160	6	0	-2.956539	1.379096	-3.893028
161	6	0	-1.647181	1.888225	-4.247917
162	6	0	-0.786075	0.939233	-4.770574
163	6	0	-2.254386	-0.979102	-4.235330
164	6	0	-1.085077	-0.451815	-4.755244
165	6	0	0.143878	-1.164848	-4.737581
166	6	0	-2.177105	-2.386099	-3.897633
167	6	0	-0.979120	-3.107356	-3.903414
168	6	0	0.283308	-2.445049	-4.232392
169	6	0	1.201290	-0.216514	-4.735592
170	6	0	2.455532	-0.479235	-4.213754
171	6	0	2.698935	-1.888211	-3.897002
172	6	0	1.647766	-2.809954	-3.906369
173	6	0	0.629410	1.084150	-4.762943
174	6	0	1.265824	2.201256	-4.251164
175	6	0	2.670087	1.992613	-3.892849
176	6	0	3.215226	0.704486	-3.869392
177	6	0	-1.016272	3.165628	-3.911725
178	6	0	0.375126	3.292581	-3.911414
179	1	0	-3.731285	2.075234	-3.584620
180	1	0	1.871769	-3.833606	-3.620353
181	1	0	4.250258	0.600352	-3.556521
182	1	0	0.794160	4.248148	-3.609325
183	6	0	5.139124	-2.109275	-4.452790
184	6	0	4.075685	-2.347557	-3.564490
185	6	0	4.388310	-3.050367	-2.395447
186	7	0	5.620293	-3.521094	-2.118803
187	6	0	6.405594	-2.603137	-4.174711
188	6	0	6.633703	-3.336245	-2.999672
189	1	0	4.965371	-1.562071	-5.374484
190	6	0	7.979661	-3.904711	-2.699313
191	6	0	6.206366	6.393716	-2.660405
192	1	0	-1.967505	-4.406957	-1.650114
193	1	0	-0.097288	-7.561797	-4.890867
194	6	0	-8.722734	-1.490571	-2.706215
195	6	0	-4.130266	7.877478	-2.691656
196	1	0	-3.079609	-2.910870	-3.597536
197	1	0	3.620991	-3.239828	-1.650079
198	1	0	7.214075	-2.414102	-4.869354
199	6	0	-9.598434	-1.926751	-3.705706
200	6	0	-10.916126	-2.257658	-3.377529
201	6	0	-11.333438	-2.150745	-2.051807
202	6	0	-10.419483	-1.688529	-1.106925
203	7	0	-9.153150	-1.365124	-1.428494
204	1	0	-9.281414	-1.984603	-4.739632
205	1	0	-11.603901	-2.588352	-4.150081
206	1	0	-12.344374	-2.407622	-1.752405
207	1	0	-10.701767	-1.578496	-0.064706
208	6	0	-4.816490	8.582088	-3.686003
209	6	0	-5.536370	9.732131	-3.350281
210	6	0	-5.560781	10.154785	-2.022198
211	6	0	-4.837650	9.422469	-1.082737
212	7	0	-4.140506	8.319085	-1.411633
213	6	0	6.660403	7.272371	-3.649406
214	6	0	7.536289	8.307160	-3.309570
215	6	0	7.939444	8.447881	-1.982681
216	6	0	7.469841	7.526544	-1.048719
217	7	0	6.631237	6.527685	-1.381523
218	6	0	8.950503	-4.060827	-3.693972
219	6	0	10.206395	-4.577151	-3.362906
220	6	0	10.470905	-4.924381	-2.039066
221	6	0	9.454138	-4.766705	-1.099414
222	7	0	8.243974	-4.274873	-1.423908
223	6	0	-1.120960	-9.724430	-3.716897
224	6	0	-1.225992	-11.079329	-3.390348
225	6	0	-1.473104	-11.442272	-2.067374
226	6	0	-1.633681	-10.429435	-1.123964
227	7	0	-1.538038	-9.125685	-1.444163

125	6	0	0.470040	6.924365	-4.157663
126	6	0	-0.078060	7.446931	-2.975719
127	7	0	-0.686716	6.614446	-2.095909
128	6	0	-0.326334	4.717668	-3.553786
129	6	0	-0.810520	5.302848	-2.378492
130	1	0	0.755857	5.179648	-5.370342
131	1	0	0.991733	7.569294	-4.853136
132	1	0	-1.316076	4.695164	-1.633095
133	6	0	-3.502808	-4.261491	-4.470388
134	6	0	-2.463765	-3.967515	-3.569945
135	6	0	-2.425655	-4.728171	-2.395900
136	7	0	-3.296209	-5.720781	-2.125763
137	6	0	-4.393000	-5.290556	-4.198614
138	6	0	-4.266373	-6.038311	-3.017624
139	1	0	-3.594059	-3.702287	-5.396745
140	6	0	-5.193143	-7.169514	-2.722627
141	6	0	2.998997	-4.634823	-4.452892
142	6	0	3.700341	-5.800500	-4.180785
143	6	0	4.454573	-5.909955	-3.002004
144	7	0	4.457574	-4.886940	-2.112903
145	6	0	3.047820	-3.552734	-3.556251
146	6	0	3.787353	-3.750153	-2.384856
147	1	0	2.435323	-4.549512	-5.377090
148	1	0	3.655714	-6.623611	-4.882426
149	1	0	3.840846	-2.967769	-1.633524
150	6	0	5.382536	1.421700	-4.458806
151	6	0	6.707660	1.718796	-4.175476
152	6	0	7.039595	2.398480	-2.993003
153	7	0	6.062331	2.724508	-2.112176
154	6	0	4.363867	1.807359	-3.569446
155	6	0	4.774244	2.446006	-2.394301
156	1	0	5.130995	0.915101	-5.385762
157	1	0	7.480452	1.417533	-4.871117
158	1	0	4.041951	2.743453	-1.648985
159	6	0	2.360824	-2.273907	-3.888691
160	6	0	3.083572	-1.077337	-3.888287
161	6	0	2.499393	0.199499	-4.245700
162	6	0	1.221148	0.118016	-4.770106
163	6	0	0.937322	-2.279799	-4.230339
164	6	0	0.466756	-1.088176	-4.754029
165	6	0	-0.911948	-0.743298	-4.739185
166	6	0	-0.098854	-3.234373	-3.890622
167	6	0	-1.460663	-2.916731	-3.898602
168	6	0	-1.904236	-1.563073	-4.232386
169	6	0	-1.009861	0.673987	-4.738361
170	6	0	-2.092202	1.359703	-4.215604
171	6	0	-3.248948	0.519705	-3.898385
172	6	0	-3.137207	-0.873693	-3.907011
173	6	0	0.306895	1.208266	-4.765356
174	6	0	0.628170	2.453174	-4.253009
175	6	0	-0.524090	3.282183	-3.894687
176	6	0	-1.812386	2.737918	-3.870624
177	6	0	2.936489	1.555489	-3.910098
178	6	0	2.026732	2.615655	-3.910844
179	1	0	4.124441	-1.117472	-3.580115
180	1	0	-4.010575	-1.452003	-3.619451
181	1	0	-2.627195	3.384397	-3.557266
182	1	0	2.391049	3.592981	-3.607513
183	6	0	-5.152585	2.062891	-4.452260
184	6	0	-4.556331	1.149640	-3.565011
185	6	0	-5.271248	0.861896	-2.396729
186	7	0	-6.483608	1.381803	-2.120732
187	6	0	-6.406031	2.589412	-4.175280
188	6	0	-7.081990	2.220224	-3.001891
189	1	0	-4.646101	2.336397	-5.373051
190	6	0	-8.445604	2.746527	-2.703862
191	6	0	0.010111	8.903857	-2.667013
192	1	0	-1.672042	-4.529539	-1.639581
193	1	0	-5.186498	-5.506606	-4.902478
194	6	0	5.244241	-7.140568	-2.707520
195	6	0	8.454435	2.756816	-2.684866
196	1	0	0.182212	-4.239464	-3.589299
197	1	0	-4.853494	0.190823	-1.651462
198	1	0	-6.853997	3.288665	-4.869725
199	6	0	5.570829	-8.061628	-3.708163
200	6	0	6.294865	-9.212045	-3.382761
201	6	0	6.675861	-9.423232	-2.058658
202	6	0	6.339604	-8.456801	-1.112762
203	7	0	5.647857	-7.347322	-1.431728
204	1	0	5.297219	-7.885267	-4.741022
205	1	0	6.559036	-9.927027	-4.156220
206	1	0	7.229396	-10.307983	-1.761303
207	1	0	6.624256	-8.571910	-0.071725
208	6	0	9.439858	2.777135	-3.677227
209	6	0	10.757593	3.098986	-3.340206
210	6	0	11.067404	3.390755	-2.012862
211	6	0	10.036657	3.374926	-1.075242
212	7	0	8.767834	3.070264	-1.405227
213	6	0	0.301206	9.848707	-3.656478
214	6	0	0.396483	11.201414	-3.318088
215	6	0	0.203151	11.585235	-1.992092
216	6	0	-0.107001	10.599052	-1.057813
217	7	0	-0.204048	9.298146	-1.389283
218	6	0	-9.248761	3.312936	-3.699075
219	6	0	-10.511788	3.813302	-3.370503
220	6	0	-10.947850	3.742338	-2.048414
221	6	0	-10.110145	3.145767	-1.108163
222	7	0	-8.897350	2.659378	-1.430447
223	6	0	-5.967178	-7.766397	-3.723196
224	6	0	-6.839990	-8.808084	-3.396685
225	6	0	-6.926432	-9.232120	-2.071584
226	6	0	-6.111912	-8.612752	-1.125795
227	7	0	-5.268376	-7.614053	-1.445869

228	1	0	-4.776247	8.268343	-4.721737
229	1	0	-6.064196	10.288813	-4.118875
230	1	0	-6.115822	11.035880	-1.716892
231	1	0	-4.817776	9.719395	-0.038979
232	1	0	6.367706	7.148028	-4.684574
233	1	0	7.899485	8.987299	-4.074251
234	1	0	8.609722	9.243646	-1.674287
235	1	0	7.764927	7.589407	-0.006123
236	1	0	8.736738	-3.815370	-4.726840
237	1	0	10.961677	-4.709053	-4.131926
238	1	0	11.436062	-5.318193	-1.737404
239	1	0	9.609512	-5.033603	-0.058911
240	1	0	-0.954287	-9.442192	-4.749002
241	1	0	-1.120531	-11.835944	-4.162127
242	1	0	-1.551289	-12.482760	-1.769252
243	1	0	-1.838236	-10.662796	-0.083927
244	80	0	-2.515410	7.375345	0.007147
245	80	0	6.246844	4.676578	0.022111
246	6	0	-0.720868	1.271156	-0.612032
247	6	0	-1.881970	0.424928	-0.056421
248	6	0	0.037665	0.441688	-1.656096
249	6	0	0.602801	-0.847598	-1.045722
250	6	0	-1.316635	-0.865357	0.564365
251	6	0	-0.564306	-1.685330	-0.494515
252	6	0	-0.332662	-0.482666	1.674704
253	6	0	0.828445	0.359532	1.129581
254	6	0	1.579478	-0.467790	0.076625
255	1	0	-2.580952	0.186603	-0.869827
256	1	0	-2.439162	0.988095	0.706556
257	1	0	0.854928	1.035420	-2.070657
258	1	0	-0.637946	0.187649	-2.467818
259	1	0	1.126165	-1.413133	-1.818606
260	1	0	-2.132935	-1.461840	0.994773
261	1	0	-1.232532	-1.965550	-1.317529
262	1	0	-0.187324	-2.616951	-0.045268
263	1	0	0.058810	-1.387996	2.140726
264	1	0	-0.859049	0.086833	2.435082
265	1	0	1.501952	0.616708	1.946796
266	1	0	2.000755	-1.367526	0.549756
267	1	0	2.421066	0.102673	-0.343636
268	1	0	-1.119542	2.175989	-1.089865
269	6	0	0.256739	1.647196	0.511995
270	1	0	1.067167	2.271580	0.106326
271	1	0	-0.246159	2.234238	1.290888

228	1	0	9.194235	2.576107	-4.712618
229	1	0	11.525734	3.125303	-4.107339
230	1	0	12.079246	3.635668	-1.706594
231	1	0	10.228011	3.605462	-0.032008
232	1	0	0.425810	9.554104	-4.691043
233	1	0	0.613278	11.940905	-4.083182
234	1	0	0.278276	12.623255	-1.684709
235	1	0	-0.277661	10.851452	-0.016067
236	1	0	-8.920647	3.345444	-4.730585
237	1	0	-11.143645	4.246714	-4.140005
238	1	0	-11.916493	4.128816	-1.748465
239	1	0	-10.410469	3.058541	-0.068814
240	1	0	-5.882048	-7.454362	-4.756711
241	1	0	-7.437655	-9.281251	-4.170078
242	1	0	-7.599061	-10.029808	-1.773451
243	1	0	-6.136295	-8.915858	-0.083873
244	80	0	6.941235	3.532273	0.008243
245	80	0	-1.221189	7.705362	0.015799
246	6	0	1.397030	0.392007	-0.628474
247	6	0	1.624404	-1.014201	-0.043370
248	6	0	0.268734	0.320537	-1.666209
249	6	0	-1.038033	-0.182972	-1.040222
250	6	0	0.316079	-1.513632	0.594425
251	6	0	-0.798204	-1.587777	-0.460896
252	6	0	-0.107189	-0.523363	1.684583
253	6	0	-0.338491	0.880104	1.109268
254	6	0	-1.458260	0.799587	0.061937
255	1	0	1.947026	-1.695157	-0.843056
256	1	0	2.420875	-0.992747	0.714903
257	1	0	0.105464	1.311582	-2.093763
258	1	0	0.561694	-0.347181	-2.470950
259	1	0	-1.811000	-0.230751	-1.809132
260	1	0	0.473988	-2.502277	1.046604
261	1	0	-0.521815	-2.272824	-1.272017
262	1	0	-1.720412	-1.975772	-0.001595
263	1	0	-1.021194	-0.878491	2.161971
264	1	0	0.671698	-0.472981	2.440194
265	1	0	-0.630263	1.555770	1.913384
266	1	0	-2.390402	0.471250	0.546099
267	1	0	-1.653246	1.787987	-0.380172
268	1	0	2.315905	0.741521	-1.117855
269	6	0	0.973933	1.373289	0.476041
270	1	0	0.840401	2.377828	0.046704
271	1	0	1.746350	1.450702	1.252206

Supplementary References

(S1) Huang, F.; Ma, L.; Che, Y.; Jiang, H.; Chen, X.; Wang, Y. Corannulene-based coordination cage with helical bias. *J. Org. Chem.* **2018**, 83, 733−739.

(S2) Piehler, T.; Lützen, A. Enantiomerically Pure C_2-symmetric Dinuclear Silver(I) and Copper(I) Complexes from a Bis(2,2'-bipyridine)-substituted 9,9'-Spirobifluorene Ligand. *J. Chem. Sci.* **2010**, 65, 329−336.

(S3) Maria, N. E.; Lawrence, T. S. Pushing the Ir-Catalyzed C-H Polyborylation of Aromatic Compounds to Maximum Capacity by Exploiting Reversibility. *J. Am. Chem. Soc.* **2012**, 134, 15169−15172.

(S4) Opris, D. M.; Franke, P. A.; Schlüter, D. Shape-Persistent Macrocycles with Bipyridine Units: Progress in Accessibility and Widening of Applicability. *Eur. J. Org. Chem.* **2005**, 16, 822-837.

(S5) Frisch, M. J.; Trucks, G. W.; Schlegel, H. B.; Scuseria, G. E.; Robb, M. A.; Cheeseman, J. R.; Scalmani, G.; Barone, V.; Mennucci, B.; Petersson, G. A.; Nakatsuji, H.; Caricato, M.; Li, X.; Hratchian, H. P.; Izmaylov, A. F.; Bloino, J.; Zheng, G.; Sonnenberg, J. L.; Hada, M.; Ehara, M.; Toyota, K.; Fukuda, R.; Hasegawa, J.; Ishida, M.; Nakajima, T.; Honda, Y.; Kitao, O.; Nakai, H.; Vreven, T.; Mont-gomery, J. A., Jr.; Peralta, J. E.; Ogliaro, F.; Bearpark, M.; Heyd, J. J.; Brothers, E.; Kudin, K. N.; Staroverov, V. N.; Kobayashi, R.; Nor-mand, J.; Raghavachari, K.; Rendell, A.; Burant, J. C.; Iyengar, S. S.; Tomasi, J.; Cossi, M.; Rega, N.; Millam, J. M.; Klene, M.; Knox, J. E.; Cross, J. B.; Bakken, V.; Adamo, C.; Jaramillo, J.; Gomperts, R.; Stratmann, R. E.; Yazyev, O.; Austin, A. J.; Cammi, R.; Pomelli, C.; Ochterski, J. W.; Martin, R. L.; Morokuma, K.; Zakrzewski, V. G.; Voth, G. A.; Salvador, P.; Dannenberg, J. J.; Dapprich, S.; Daniels, A. D.; Farkas, O.; Foresman, J. B.; Ortiz, J. V.; Cioslowski, J.; Fox, D. J. Gaussian 09, revision D.01; Gaussian, Inc.: Wallingford, CT, 2009.

(S6) Grimme, S.; Antony, J.; Ehrlich, S.; Krieg, H. A consistent and accurate *ab initio* parameterization of density functional dispersion correction (DFT-D) for the 94 elements H-Pu. *J. Chem. Phys.* **2010**, 132, 154104- 154118.

(S7) Grimme, S.; Ehrlich, S.; Goerigk, L. Effect of the damping function in dispersion corrected density functional theory. *J. Comput. Chem.* **2011**, 32, 1456–1465.
