# Supplementary material for: Dual-controlled guest release from coordination cages
Source: Commun Chem. 2024 Feb 27;7:43. doi: 10.1038/s42004-024-01128-z (PMC10899651; doi:10.1038/s42004-024-01128-z)
Supplement: Supplementary file 4 — Supplementary Data 1 [file 42004_2024_1128_MOESM4_ESM.pdf]

Supplementary Data

Dual-controlled Guest Release from Coordination Cages

Yuqing Yao,† Chengyuan Shao,† Shuwei Wang, Qiufang Gong, Jia Liu, Hua Jiang,* and Ying Wang*

College of Chemistry, Beijing Normal University, Beijing 100875, China

†Equal contribution

Email: ywang1@bnu.edu.cn and jiangh@bnu.edu.cn

Routine ^{1}H NMR and ^{13}C NMR Spectra of Compounds

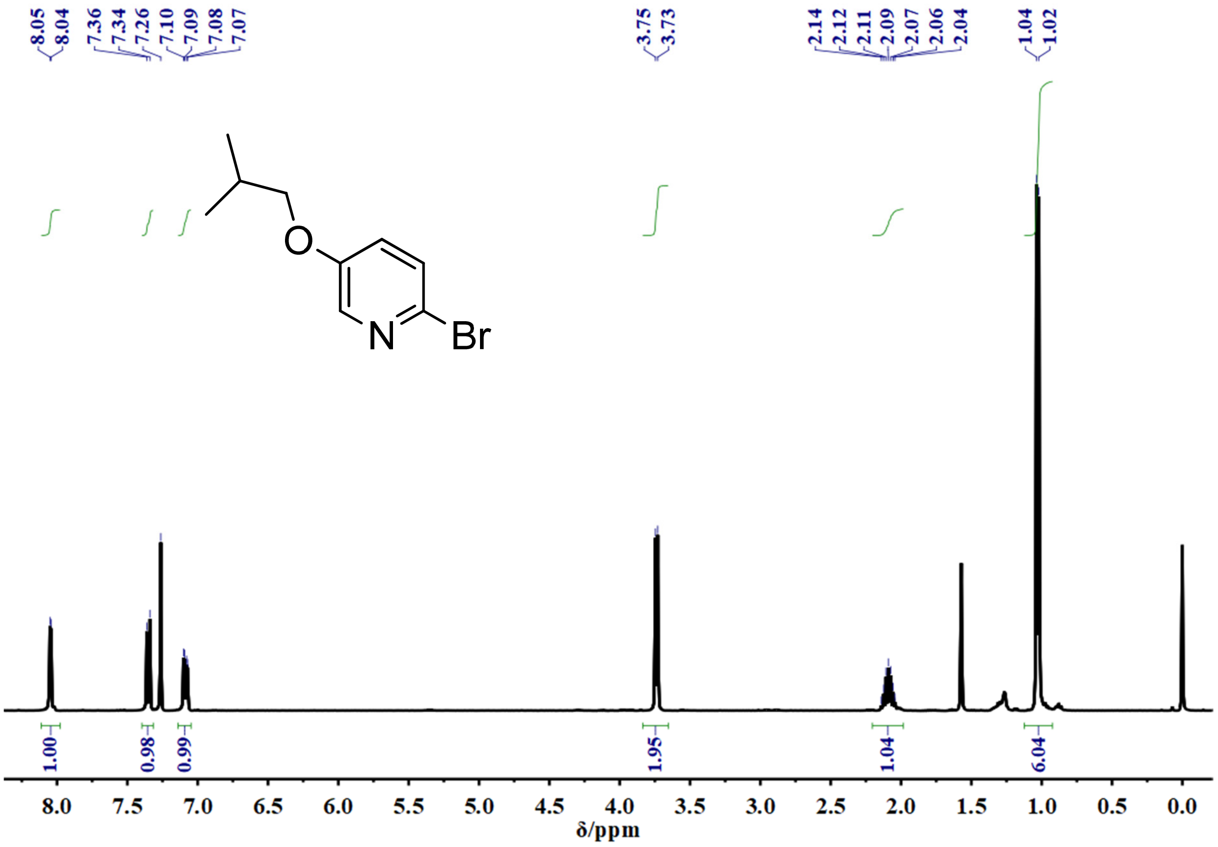

^{1}H NMR spectrum (400 MHz, $CDCl_3$, 298 K) of compound **2**.

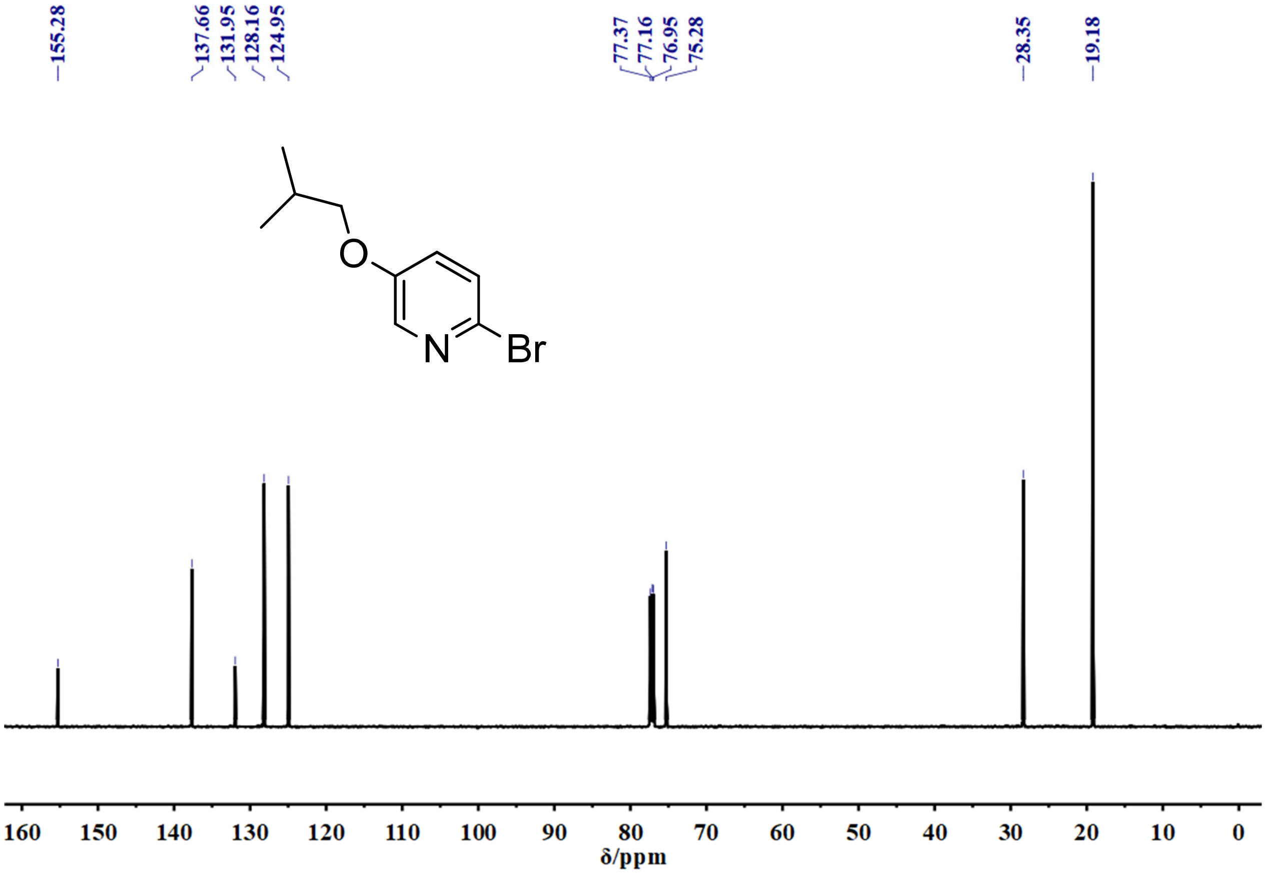

^{13}C NMR spectrum (150 MHz, $CDCl_3$, 298 K) of compound **2**.

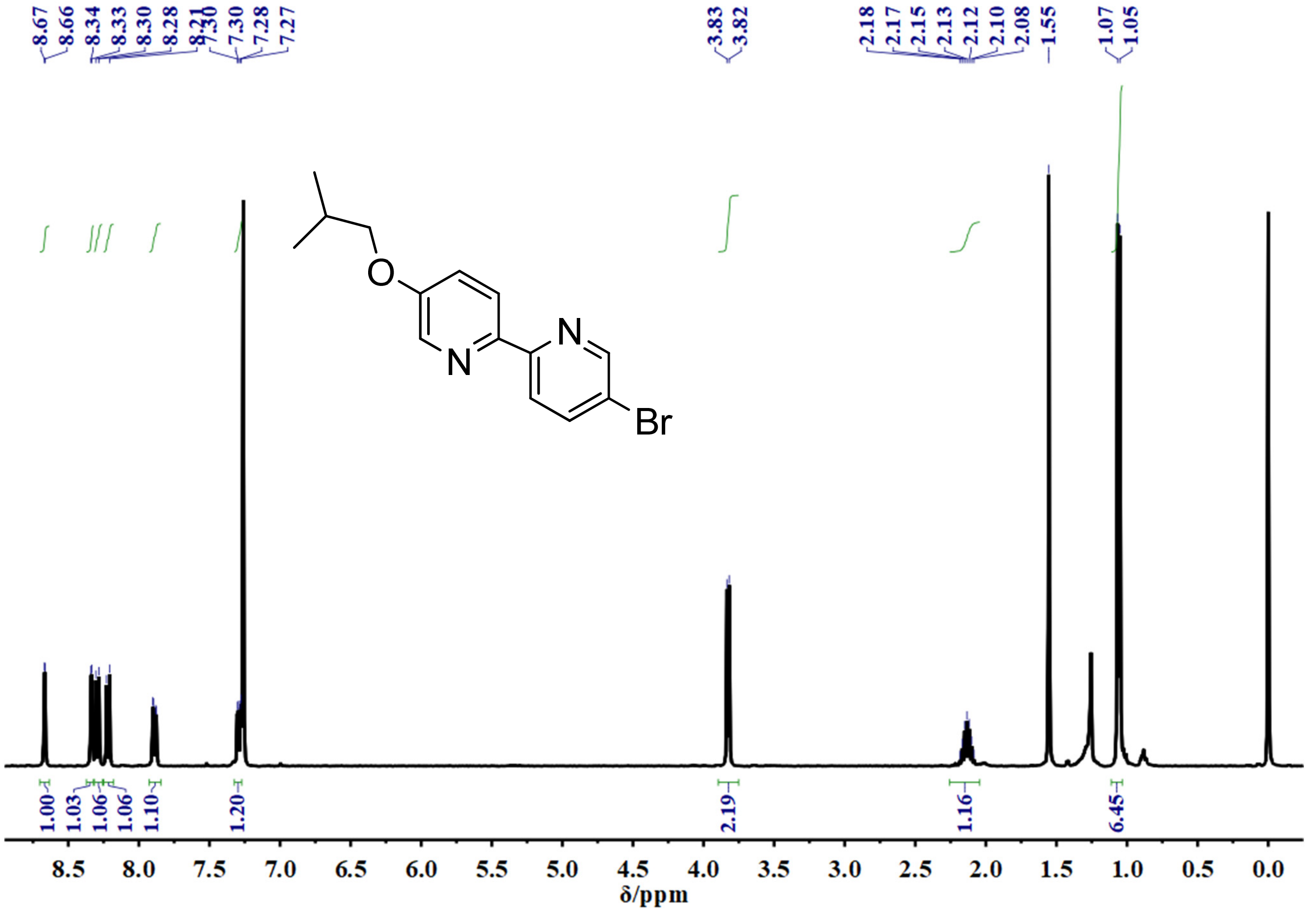

^{1}H NMR spectrum (400 MHz, $CDCl_3$, 298 K) of compound **3**.

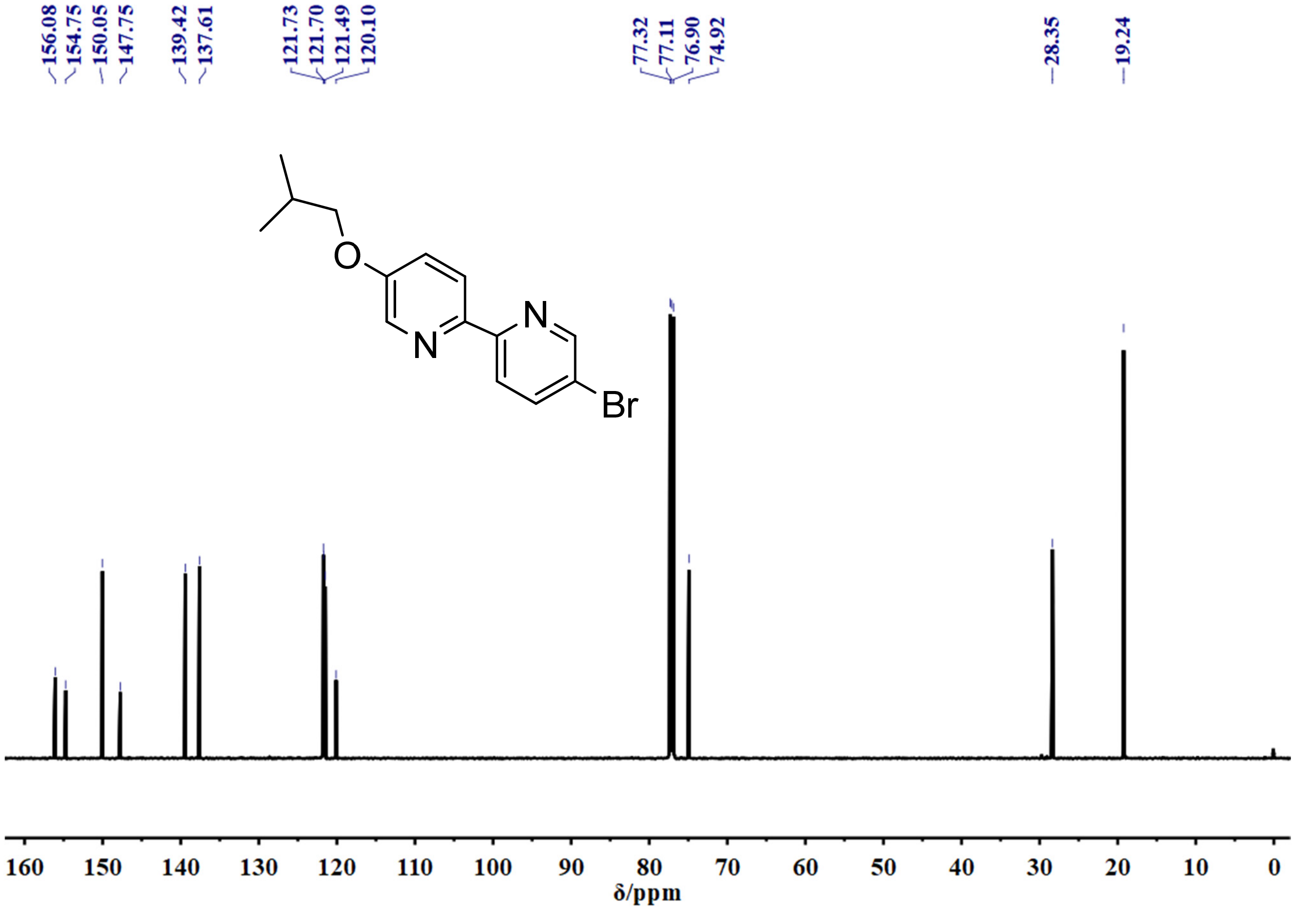

^{13}C NMR spectrum (150 MHz, $CDCl_3$, 298 K) of compound **3**.

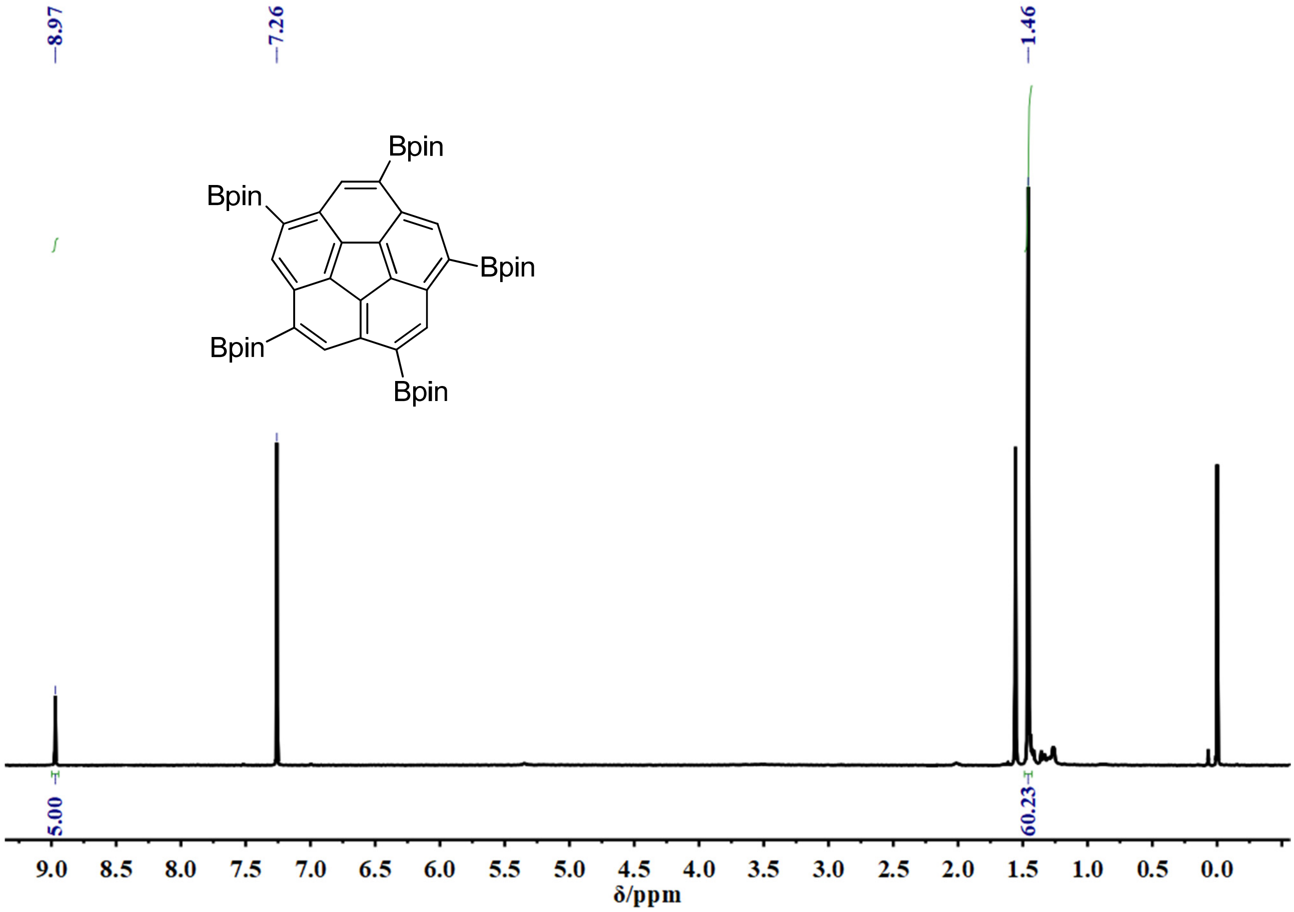

^{1}H NMR spectrum (400 MHz, $CDCl_3$, 298 K) of compound **4**.

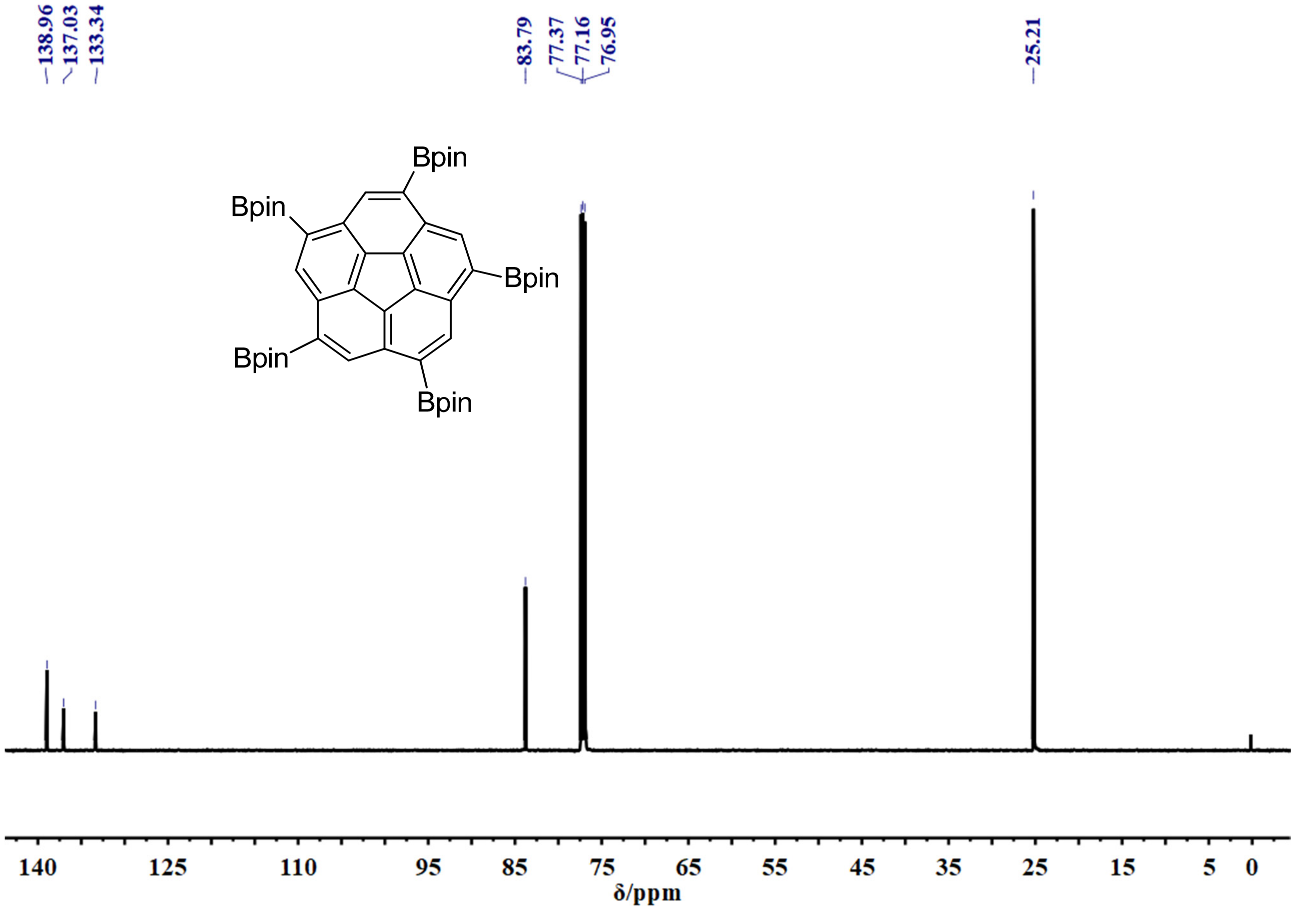

^{13}C NMR spectrum (150 MHz, $CDCl_3$, 298 K) of compound **4**

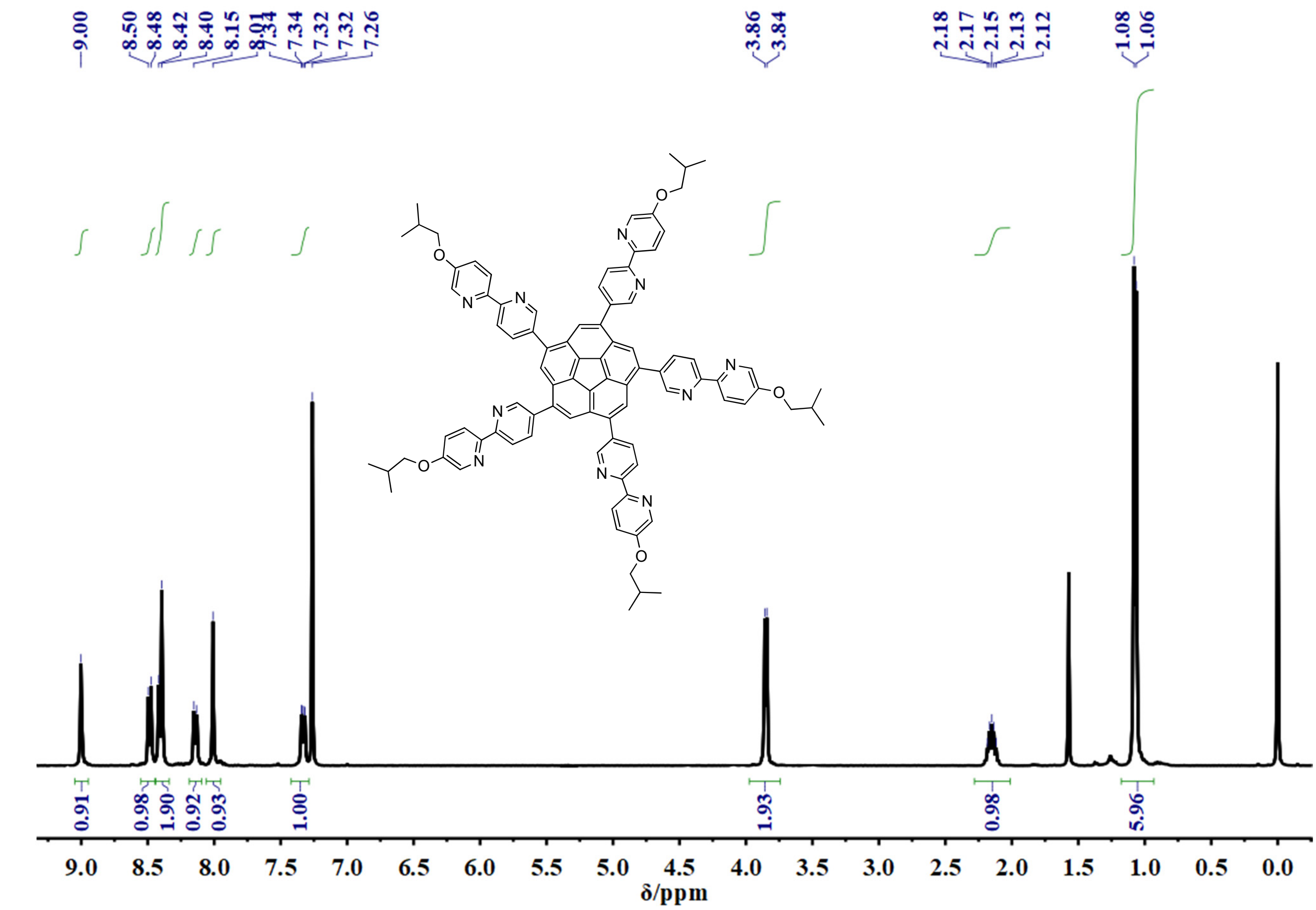

^{1}H NMR spectrum (400 MHz, $CDCl_3$, 298 K) of the ligand **1**.

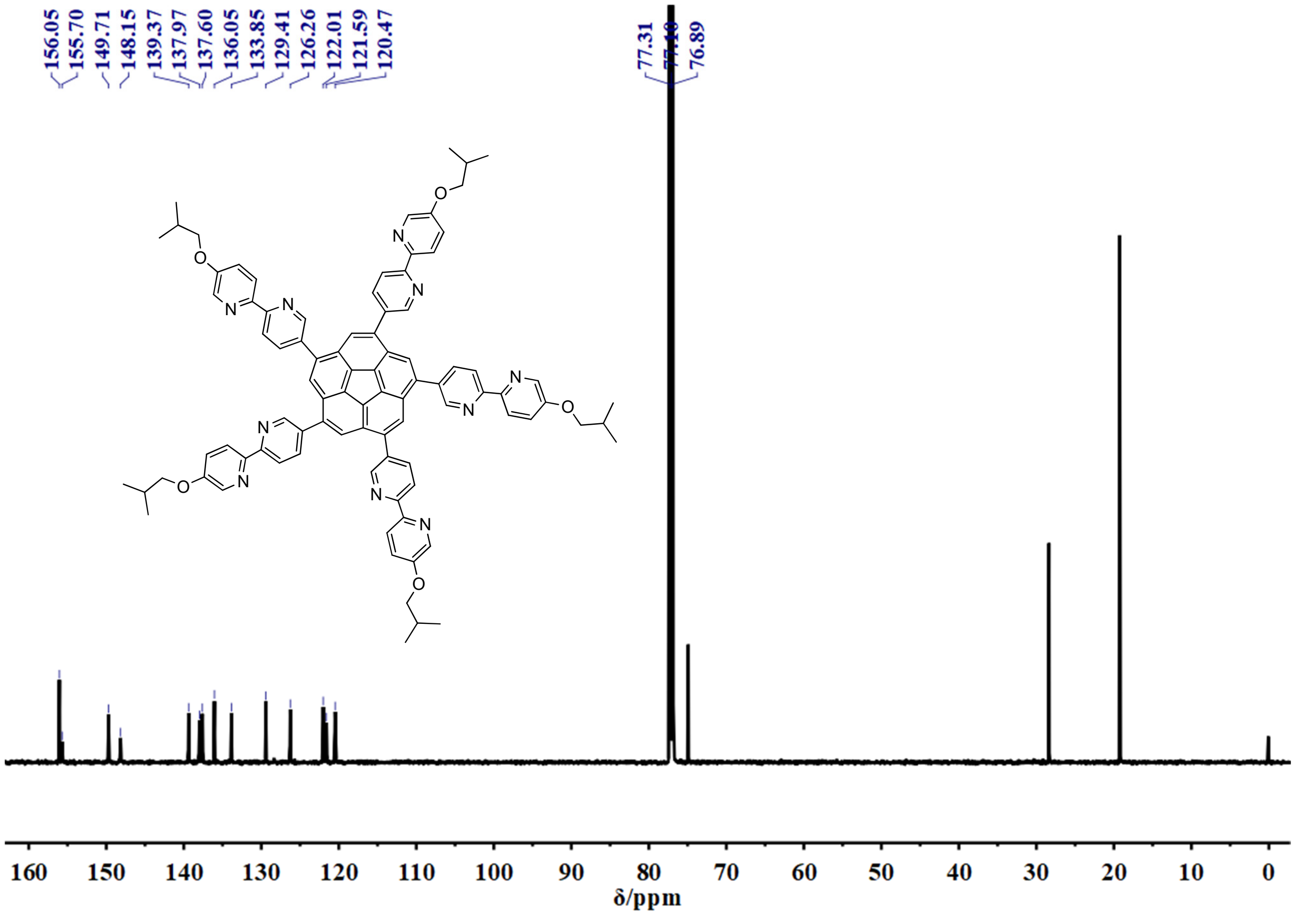

^{13}C NMR spectrum (150 MHz, $CDCl_3$, 298 K) of the ligand **1**.
