# Supplementary material for: Dual-controlled guest release from coordination cages
Source: Commun Chem. 2024 Feb 27;7:43. doi: 10.1038/s42004-024-01128-z (PMC10899651; doi:10.1038/s42004-024-01128-z)
Supplement: Supplementary file 1 — Peer Review File [file 42004_2024_1128_MOESM1_ESM.pdf]

nature portfolio

# Peer Review File

## Dual-controlled guest release from coordination cages

**Open Access** This file is licensed under a Creative Commons Attribution 4.0 International License, which permits use, sharing, adaptation, distribution and reproduction in any medium or format, as long as you give appropriate credit to the original author(s) and the source, provide a link to the Creative Commons license, and indicate if changes were made. In the cases where the authors are anonymous, such as is the case for the reports of anonymous peer reviewers, author attribution should be to 'Anonymous Referee' followed by a clear attribution to the source work. The images or other third party material in this file are included in the article's Creative Commons license, unless indicated otherwise in a credit line to the material. If material is not included in the article's Creative Commons license and your intended use is not permitted by statutory regulation or exceeds the permitted use, you will need to obtain permission directly from the copyright holder. To view a copy of this license, visit http://creativecommons.org/licenses/by/4.0/.

*This manuscript has been previously reviewed at another Nature Portfolio journal. This document only contains reviewer comments and rebuttal letters for versions considered at Communications Chemistry.*

REVIEWERS' COMMENTS:

Reviewer #1 (Remarks to the Author):

The authors almost appropriately revised the manuscript, which was initially submitted to [redacted], according to the reviewers' comments, and the revised version has now been suitable for publication in Communications Chemistry.

**Response to reviews:**

**Reviewer #1 (Remarks to the Author):**

*The authors almost appropriately revised the manuscript, which was initially submitted to [redacted], according to the reviewers' comments, and the revised version has now been suitable for publication in in Communications Chemistry.*

**Answer:** We are very grateful to the reviewer for the positive comments.
